# Network pharmacology study to explore the multiple molecular mechanism of SH003 in the treatment of non-small cell lung cancer

**DOI:** 10.1186/s12906-024-04347-y

**Published:** 2024-02-01

**Authors:** Kangwook Lee, Yu-Jeong Choi, Hae-In Lim, Kwang Jin Cho, Nuri Kang, Seong-Gyu Ko

**Affiliations:** 1 Department of Food and Biotechnology, Korea University, Sejong, 30019 South Korea; 2 Department of Preventive Medicine, College of Korean Medicine, Kyung Hee University, Seoul, 02447 South Korea; 3 Department of Science in Korean Medicine, Graduate School, Kyung Hee University, Seoul, 02447 South Korea; 4 Department of Korean Medicine, Graduate School, Kyung Hee University, Seoul, 02447 South Korea

**Keywords:** Network pharmacology, Non-small cell lung cancer, SH003, Gene ontology, KEGG

## Abstract

**Background:**

Non-small cell lung cancer (NSCLC) is one of the leading causes of human death worldwide. Herbal prescription SH003 has been developed to treat several cancers including NSCLC. Due to the multi-component nature of SH003 with multiple targets and pathways, a network pharmacology study was conducted to analyze its active compounds, potential targets, and pathways for the treatment of NSCLC.

**Methods:**

We systematically identified oral active compounds within SH003, employing ADME criteria-based screening from TM-MC, OASIS, and TCMSP databases. Concurrently, SH003-related and NSCLC-associated targets were amalgamated from various databases. Overlapping targets were deemed anti-NSCLC entities of SH003. Protein–protein interaction networks were constructed using the STRING database, allowing the identification of pivotal proteins through node centrality measures. Empirical validation was pursued through LC–MS analysis of active compounds. Additionally, in vitro experiments, such as MTT cell viability assays and western blot analyses, were conducted to corroborate network pharmacology findings.

**Results:**

We discerned 20 oral active compounds within SH003 and identified 239 core targets shared between SH003 and NSCLC-related genes. Network analyses spotlighted 79 hub genes, including *TP53*, *JUN*, *AKT1*, *STAT3*, and *MAPK3*, crucial in NSCLC treatment. GO and KEGG analyses underscored SH003’s multifaceted anti-NSCLC effects from a genetic perspective. Experimental validations verified SH003’s impact on NSCLC cell viability and the downregulation of hub genes. LC–MS analysis confirmed the presence of four active compounds, namely hispidulin, luteolin, baicalein, and chrysoeriol, among the eight compounds with a median of > 10 degrees in the herb-compounds-targets network in SH003. Previously unidentified targets like *CASP9*, *MAPK9*, and *MCL1* were unveiled, supported by existing NSCLC literature, enhancing the pivotal role of empirical validation in network pharmacology.

**Conclusion:**

Our study pioneers the harmonization of theoretical predictions with practical validations. Empirical validation illuminates specific SH003 compounds within NSCLC, simultaneously uncovering novel targets for NSCLC treatment. This integrated strategy, accentuating empirical validation, establishes a paradigm for in-depth herbal medicine exploration. Furthermore, our network pharmacology study unveils fresh insights into SH003’s multifaceted molecular mechanisms combating NSCLC. Through this approach, we delineate active compounds of SH003 and target pathways, reshaping our understanding of its therapeutic mechanisms in NSCLC treatment.

**Supplementary Information:**

The online version contains supplementary material available at 10.1186/s12906-024-04347-y.

## Background

Cancer is one of the leading causes of human death worldwide [1, 2]. According to the American Cancer Society, lung cancer has been by far the leading cancer for cancer death among both men and women from 1998 to 2021, followed by breast cancer, prostate cancer, colorectal cancer [1, 2]. Based on the histologic features, lung cancers are classified into two types: small cell lung cancers and non-small cell lung cancers (NSCLC) which account for 80 ~ 90% of lung cancers. The absence of readily apparent clinical symptoms and the absence of efficient screening programs frequently result in delayed diagnoses, often occurring in the advanced stages of the disease, thereby constraining treatment possibilities. Diagnosis necessitates the utilization of imaging modalities including X-ray, CT scans, and PET scans, to pinpoint the tumor’s location and the collection of a biopsy specimen for tumor classification and staging. Due to the delayed detection of NSCLC, the prognosis for the majority of NSCLC patients is quite bleak, with a median overall survival of merely one year following diagnosis. The number of incidence and death of NSCLC patients has been continuously increased despite remarkable advances in cancer diagnostics and anti-cancer strategies [3]. Cytotoxic drugs such as paclitaxel, docetaxel, cisplatin, 5-fluorouracil show strong anti-cancer efficacy while severe adverse effects including neutropenia, leukopenia, anorexia, cachexia are still a problem [4, 5]. Meanwhile, targeted therapy and immunotherapy have been developed to overcome these problems but it has faced to acquired resistance, poor therapeutic response, systemic immune dysfunction [6, 7]. Therefore, the improvement of prognosis and quality of life for NSCLC patients remains a challenge in worldwide.

Accumulating studies have reported that herbal medicines and their derivatives are the representative alternative treatment for solving health problems including cancer [8–11]. SH003 is a unique anti-cancer herbal mixture consisting of Astragalus membranaceus (AM), Angelica gigas (AG), and Trichosanthes kirilowii Maximowicz (TK), which is based on traditional Korean medicine theory. SH003 has been reported to have anti-cancer effects against various types of cancer [12–23]. According to our previous references, SH003 exhibits promising anti-cancer effects across various cancers, including breast and lung cancer, along with the ability to overcome drug resistance. It synergizes with conventional chemotherapy, indicating its potential in combination therapies. Furthermore, SH003 displays anti-angiogenic properties, reducing tumor growth and metastasis. It also addresses cancer-related adverse effects, such as chemotherapy-induced peripheral neuropathy and immune suppression, enhancing its therapeutic potential. These findings highlight SH003 as a multi-target herbal mixture with significant implications in cancer treatment and symptom management, offering a novel approach to combatting this complex disease. Our thorough toxicity assessments in Sprague–Dawley rats validate SH003’s safety [18]. Acute toxicity studies indicated a high lethal dose (> 2000 mg/kg), signifying low acute toxicity. In four-week-repeated oral dose studies, SH003 showed no adverse effects on parameters like body weight, hematological values, and clinical signs, affirming its safety. A thirteen-week-repeated oral dose study, followed by a four-week recovery period, revealed no significant differences in organ weights and clinical signs. Liver hypertrophy, observed at thirteen weeks, was reversible, establishing a no-observed-adverse-effect level > 2500 mg/kg for both genders. These results underscore SH003’s low toxicity and its potential for clinical use. In addition, preclinical Good Laboratory Practice toxicity assessments revealed no adverse effects associated with SH003 administration, and human liver microsomes incubated with SH003 and a panel of cytochrome P450 (CYP) substrates, including phenacetin, coumarin, paclitaxel, diclofenac, ( ±)-mephenytoin, dextromethorphan, and midazolam, demonstrated minimal inhibitory impacts on all CYP isozymes, indicative of an absence of herb-drug interactions interaction [18]. Taken together, accumulating evidences have suggested that SH003 would be a promising herbal medicine for the treatment of cancer, both as a single therapy and in combination with other treatments, and for enhancing the quality of life of cancer patients. Safety of SH003 for patients with solid tumors has been demonstrated in a phase I clinical study. The successful completion of Phase 1 clinical trials with a dosage of 4800 mg/day for solid tumor patients without any observed toxicity is an encouraging outcome [24]. A phase II clinical study for wild-type EGFR NSCLC is currently underway [25]. Additionally, a phase I/II basket trial for combination therapy of SH003 and docetaxel is also in progress [26]. In light of this context, we hypothesized that SH003 would be a novel strategy for improvement of prognosis and and quality of life for NSCLC patients.

Network pharmacological analysis, originally developed by Hopkins, represents a cutting-edge multidisciplinary approach that seamlessly integrates principles from systems biology, network analysis, and pharmacology [27]. This innovative methodology stands as a beacon in modern drug discovery, serving a dual purpose of enhancing clinical efficacy while unraveling the complexities of side effects and toxicity [27]. At its core, network pharmacology hinges upon the creation and examination of intricate networks linking herbs to compounds, compounds to genes, and genes to diseases. These networks are meticulously constructed using data from web databases, enabling researchers to delve deep into the labyrinthine interactions among various components. This holistic approach illuminates the potential synergistic effects and elucidates the underlying mechanisms at play within target pathways. Recent scientific endeavors have increasingly harnessed the power of network pharmacology, employing it as a formidable tool to identify the primary active compounds within herbal medicines and shed light on their intricate interactions with the molecular pathways underpinning various diseases [28–36]. Despite the fact that SH003 has multiple components, targets and pathways, previous studies on its efficacy and mechanism of action in cancer treatment have focused on single pathways or targets. Therefore, it is important to consider that network pharmacological analysis is a valuable and necessary approach for comprehending the synergistic interactions between the numerous compounds of SH003 and their disease targets. Notably, Lee et al. has reported the therapeutic mechanism of SH003 for breast cancer at system level by network pharmacological analysis [29]. This study reported that targets of active compounds in SH003 are functionally enriched in multiple pathways in breast cancer including TNF signaling pathway, estrogen signaling pathway, PI3K-Akt signaling pathway and MAPK signaling pathway. It could be helpful for understanding the fine details of SH003-targets interaction and for investigating potential therapeutic targets of SH003 on breast cancer in clinical trials. Through this systematic and integrative lens, we aim to elucidate the intricate workings of SH003, shedding light on its multi-target potential and advancing our understanding of its therapeutic mechanisms.

**Fig. 1:**
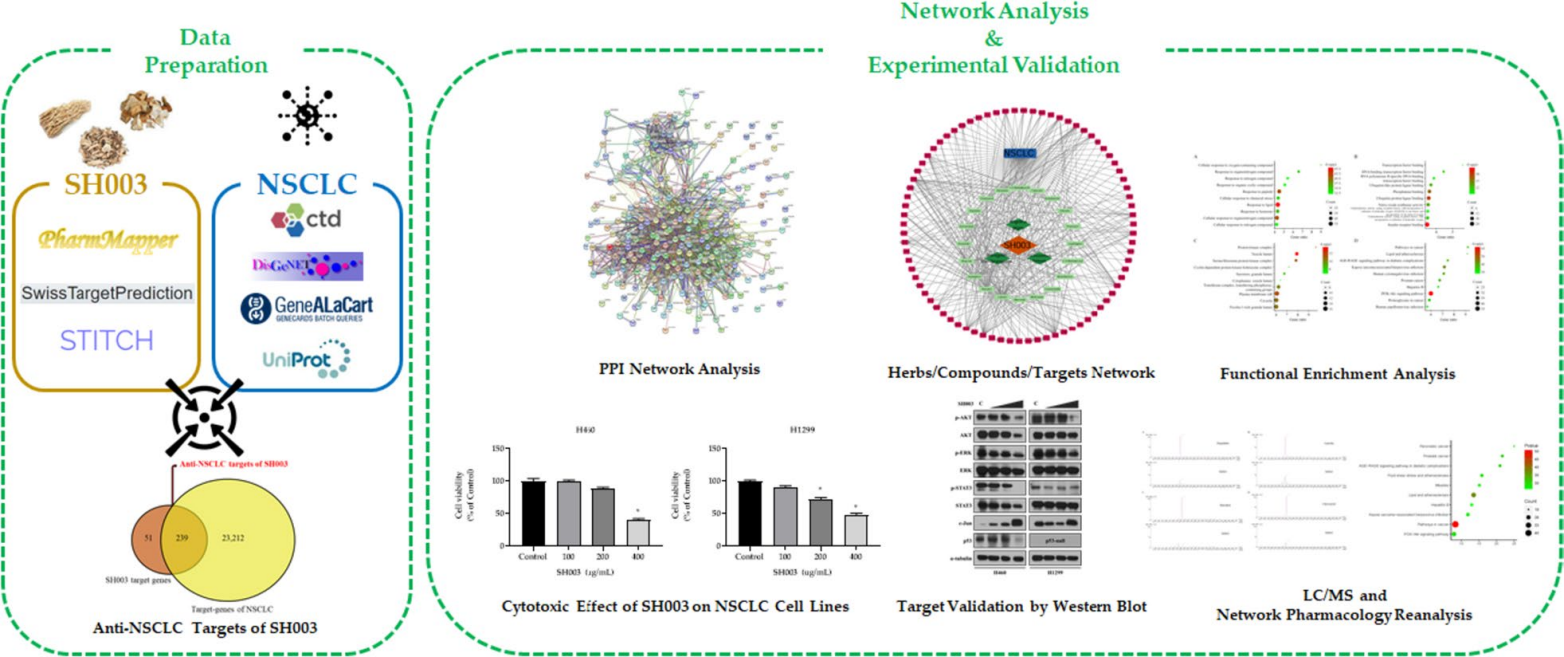
The flowchart for exploring the molecular mechanism of SH003 against NSCLC

In this study, we employed network pharmacology to investigate the active compounds of SH003 with multi-targets and multi-pathways against NSCLC, providing a reference for further anti-cancer research on SH003 (Fig. 1). First, the active compounds of SH003 were screened from TM-MC, OASIS and TCMSP database and target proteins of each component were collected from PharmMapper, SwissTargetPrediction and STITCH database. The intersecting target genes of NSCLC were collected from CTD, DisGeNET and GeneCards and the genes common to both SH003 network and NSCLC-related genes were determined as anti-NSCLC targets of SH003. Protein–protein interaction (PPI) network and pharmacological networks of the selected NSCLC targets of SH003 were constructed. Gene ontology (GO) and Kyoto Encyclopedia for Genes and Genomes (KEGG) pathway analysis for the selected NSCLC targets of SH003 were conducted. In addition, the inhibitory effect of SH003 on viability of NSCLC cell lines was measured by MTT assay. Western blot analysis was performed to verify the results of functional enrichment analysis. Consequently, the present study represents deeper insights about active compounds and multiple molecular mechanisms of SH003 in NSCLC using network pharmacology and in vitro experiments.

## Materials and methods

### The collection and screen of potential active compounds in SH003

The chemical components of AM, AG and TK were obtained from web databases including TM-MC (https://informatics.kiom.re.kr/compound/), OASIS (https://oasis.kiom.re.kr/) and TCMSP (https://tcmsp-e.com/index.php) (Table S1). Further selection of potential active compounds of SH003 was performed on the basis of absorption, distribution, metabolism and excretion (ADME) profiles, which were obtained from Traditional Chinese Medicine Systems Pharmacology (TCMSP, https://old.tcmsp-e.com/tcmsp.php) database [37]. TCMSP is a unique database including drug-target-disease networks as well as pharmacokinetic properties for phytochemicals involving molecular weight (MW), ALogP, H-bond donor (Hdon), H-bond acceptor (Hacc), oral bioavailability (OB), Caco-2 permeability (Caco-2), drug-likeness (DL), rotatable bonds (RBN) and etc. In this study, the pharmacokinetic parameters of each compound consisting of MW, ALogP, Hdon, Hacc, OB, DL, Caco-2 and RBN were investigated. According to the Lipinski’s rule of five, orally active compounds follows criteria bellow: MW < 500 da, ALogP < 5, Hdon < 5, Hacc < 10 [38]. OB means that the rate and extent to which the active ingredient or active moiety is absorbed from a drug product and becomes available at the site of action [39]. DL is a qualitative parameter to assess whether molecular properties of a compound are suitable for drug design based on the similarity with conventional drugs [40]. Caco-2 is commonly used as an efficient tool to evaluate the ability of drug intake in the gut [41]. RBN refers to molecular flexibility [42]. In addition to the Lipinski’s rule of five, compounds with OB ≥ 30%, DL ≥ 0.18, Caco-2 > -0.4 and RBN ≤ 10 were classified as pharmaceutically active compounds.

### Target prediction of active compounds in SH003

Possible targets of SH003 active compounds were collected from three well known databases, namely, PharmMapper (http://lilab-ecust.cn/pharmmapper), SwissTargetPrediction (http://swisstargetprediction.ch) and STITCH (http://stitch.embl.de). The parameters of PharmMapper were set as normalized fit score > 0.7. The cut-off of SwissTargetPrediction was set as probability > 0.7. According to STITCH database, compound-target interactions were screened with a species limited to ‘Homo sapiens’ and the high confidence score > 0.7. The name of collected targets was standardized by using UniProt (https://www.uniprot.org/) [43] database. Target genes of active compounds in SH003 was presented in Table S2.

### NSCLC-related targets

The information of NSCLC-related genes was obtained from public databases including CTD (http://ctdbase.org) [44], DisGeNET (http://www.disgenet.org) [45] and GeneCards (http://www.genecards.org) [46] database by using keywords ‘non-small cell lung cancer’ with the species limited to ‘Homo sapiens’. The name of NSCLC-targets was matched by using UniProt (https://www.uniprot.org/) [43].

### Protein–protein interaction (PPI) network analysis

A Venn diagram was drawn to obtain overlapping targets between SH003-related targets and NSCLC-related targets. Using the Search Toll for the Retrieval of Interacting Genes/Proteins (STRING 11.0, http://string-db.org), we constructed a PPI network of overlapping targets between SH003 compounds and NSCLC with a species limited to ‘Homo sapiens’ and the high confidence score > 0.7 [47]. Topological analysis and screening the hub genes in the PPI network were performed using Cytoscape software [48]. Three topological measures in Cytoscape including Degree, Betweenness Centrality and Closeness Centrality were computed for each node to find hub genes [49–55]. Degree corresponds to the number of neighbors of a node. Betweenness Centrality quantifies the number of times a node acts as a bridge along the shortest path between two other nodes. Closeness Centrality indicates the importance of a node in the PPI network by measuring how close a given node is to the other nodes. The cut-off to filter the core targets was set up according to the median value from results of each topological analysis, followed by the selection of common genes in the three methods as key targets. Using the core targets based on PPI analysis results, we constructed herb-compound-target network and further visualized and analyzed by using Cytoscape [48].

### GO and KEGG pathway enrichment analysis

GO [56] and KEGG [57–59] pathway enrichment analysis were conducted by using ClueGO 2.5.8 [60], which is Cytoscape plug-in which analyses GO and pathway annotation networks. GO analysis was applied to analyze the functions including biological process (BP), molecular function (MF) and cellular component (CC). Load marker lists of genes were limited to ‘Homo sapiens [9606]’. The bubble chart of GO and KEGG enrichment analysis was constructed using an online R package (http://www.ehbio.com/ImageGP/index.php/Home). X-axis and Y-axis represent % Associated Genes and description, respectively. The significance threshold for terms and pathways is set at *p* < 0.001.

### Preparation of SH003

SH003 was prepared by Hanpoong Pharm and Foods Company (Jeonju, South Korea). In brief, AM (333 g), AG (333 g) and TK (333 g) were mixed and extracted with 10 times volume of 30% ethanol for 3 h at about 90–100 °C. The extract was dried for 16 h at 60 °C with reduced pressure (40 Torr). The powder form of dried SH003 was stored at -20 °C until use.

### Cell culture

Human NSCLC cell lines, H460 and H1299, were purchased from Korean Cell Line Bank (KCLB, Seoul, South Korea). Cell lines were grown in RPMI1640 medium (WelGENE Inc., Daegu, South Korea) supplemented with 10% fetal bovine serum (FBS; JR Scientific, Woodland, CA, USA) and 1% penicillin/streptomycin solution (WelGENE, Daegu, Korea). Cell lines were maintained at 37 °C in a humidified atmosphere with 5% CO_2_.

### Cell viability assay

Cell viability was measured using the MTT assay (M5655, Sigma-Aldrich, MO, USA). A total of 5 × 10^3^ cells per well were seeded into 96-wells plate. After 24 h, cell lines were treated with SH003 (100, 200 and 400 μg/mL) for 24 h. Following treatment, the medium was carefully suctioned and 100 uL of MTT working solution in the complete medium (0.5 mg/mL) was added into each well. After 1 h incubation, the supernatant was suctioned and MTT formazan crystals were dissolved by adding 100 uL of dimethyl sulfoxide (DMSO). The absorbance was measured at 570 nm by using spectrophotometer (Molecular Devices, CA, USA).

### Western blot analysis

To validate the effect of SH003 on hub targets determined by network pharmacology analysis, we performed the western blot. Following treatment of SH003 on H460 and H1299, whole proteins were extracted using ice-cold radioimmunoprecipitation assay buffer (R2002, Biosesang, Seongnam, South Korea) containing protease and phosphatase inhibitors. Protein concentration was quantified by Bradford assay (#5000006, Bio-Rad, Hercules, CA, USA) and equal amounts of proteins were separated on 10% SDS-PAGE. The separated proteins on gel were transferred to PVDF membrane (IPVH00010, Merck Millipore Ltd., MA, USA) with 90 – 120 V for 60 ~ 90 min, followed by blocking with tris buffered saline with Tween-20 (TBS-T) containing 5% bovine serum albumin (BSA) at room temperature for 1 h. The blocked membrane was incubated with anti-p53 (1:1,000, #2527, 453 µg/mL, Cell Signaling Technology), anti-c-Jun (1:1,000, #9165, 48 µg/mL, Cell Signaling Technology), anti-AKT (1:1,000, #9272, 31 µg/mL, Cell Signaling Technology), anti-phospho-AKT (1:1,000, #9271, 10 µg/mL, Cell Signaling Technology), anti-STAT3 (1:1,000, #4904, 24 µg/mL, Cell Signaling Technology), anti-phospho-STAT3 (1:1,000, #9145, 100 µg/mL, Cell Signaling Technology), anti-ERK (1:1,000, sc-1647, 200 µg/mL, Santa Cruz Biotechnology), anti-phospho-ERK (1:1,000, sc-7383, 200 µg/mL, Santa Cruz Biotechnology) and anti-alpha-tubulin (1:3,000, #3873, 358 µg/mL, Cell Signaling Technology) antibodies at 4 °C for 16-24 h. Horseradish peroxidase-conjugated secondary IgG antibodies (#7074 for anti-rabbit, 77 µg/mL; #7076 for anti-mice, 184 µg/mL) were purchased from Cell Signaling Technology and incubated with the membrane at room temperature for 1 h. Immobilon Western chemiluminescent HRP substrate (WBKLS0500, Merck Millipore Ltd., MA, USA) was used for detection of horseradish peroxidase signal. The same PVDF membrane was used to blot each band in the western blot results, following the manufacturer’s protocol for antibody stripping with Restore™ PLUS Western Blot Stripping Buffer (#46430–500 mL, Thermo Scientific, Rockford IL, USA).

### LC–MS analysis

Liquid chromatography-mass spectrometry (LC–MS) analysis were performed to validate whether the active compounds found by network pharmacology are actually existed in SH003. LC–MS analysis was conducted using a Waters TQD instrument. The reference component (four active compounds, 1.0 mg each) was weighed, dissolved in 1.0 mL of methanol to prepare a solution at a concentration of 1.0 mg/mL, then diluted. SH003 powder (10.0 mg) was weighed, sonicated in 1 mL of methanol for 10 min, and filtered through a 0.22 μm syringe filter. Chromatographic separation was achieved on an InfinityLab Poroshell 120 EC-C18 column (100 × 2.1 mm, 2.7 µm, Agilent) with a gradient elution profile using mobile phases A (0.1% Formic acid, 5 mM Ammonium Formate in Water) and B (0.1% Formic acid, 5 mM Ammonium Formate in Methanol). The gradient program commenced with 99% A at 0 min, transitioned to 20% A at 3 min, maintained this composition until 4 min, shifted to 1% A at 5 min, increased to 8% A at 8 min, returned to 99% A at 8.5 min, and persisted until the end of the 12-min analysis. The column temperature was maintained at 40 ℃, and the flow rate was set at 0.5 mL/min. Subsequent network pharmacology analysis was performed for the compounds validated via chromatography analysis.

### Statistics

The statistical test used for enrichment was based on the Benjamini–Hochberg method for multiple test correction, and only terms and pathways with *p* < 0.001 were considered significant. Statistical analysis for in vitro study was performed using PRISM 8.0.2 (GraphPad, San Diego, CA, USA). The normality of MTT data was assessed using the Shapiro–Wilk test. The differences of means between the groups were analyzed by two tailed unpaired Student’s t-test with Welch’s correction. *P* value < 0.05 means statistically significant difference.

## Results

### Active compounds screening based on ADME criteria

The ingredients of AM, AG and TK were obtained from TM-MC, OASIS and TCMSP web database. After deleting the duplicates and uninformed components, 965 compounds were collected (Table S1). According to the pharmacokinetic data from TCMSP database, total 20 compounds were selected as oral active compounds of SH003 (15 compounds in AM, one compound in AG and four compounds in TK), which indicating that active compounds of SH003 were mostly concentrated on AM (Table 1). There were no common active compounds in AM, AG and TK.

**Table 1:** The selected active compounds of SH003

Source	Compound name	PubChem CID	MW	ALogP	Hdon	Hacc	OB (%)	Caco-2	DL	RBN
*Astragalus membranaceus*	3'-O-Methylorobol	5,319,744	300.26	2.05	3	6	57.41	0.45	0.27	2
	Baicalein	5,281,605	270.24	2.33	3	5	33.52	0.63	0.21	1
	Calycosin	5,280,448	284.26	2.32	2	5	47.75	0.52	0.24	2
	Formononetin	5,280,378	268.26	2.58	1	4	69.67	0.78	0.21	2
	Hesperetin	72,281	302.28	2.28	3	6	70.31	0.37	0.27	2
	Hispidulin	5,281,628	300.26	2.32	3	6	30.97	0.48	0.27	2
	Isorhamnetin	5,281,654	316.26	1.76	4	7	49.60	0.31	0.31	2
	Kaempferol	5,280,863	286.24	1.77	4	6	41.88	0.26	0.24	1
	Kumatakenin	5,318,869	314.29	2.09	2	6	50.83	0.61	0.29	3
	Liquiritigenin	114,829	256.25	2.57	2	4	32.76	0.51	0.18	1
	Medicarpin	336,327	270.28	2.66	1	4	49.22	1.00	0.34	1
	Mosloflavone	471,722	298.29	2.84	1	5	34.04	0.86	0.26	3
	Odoratin	13,965,473	314.29	2.3	2	6	49.95	0.42	0.30	3
	Pratensein	5,281,803	300.26	1.37	2	6	39.06	0.39	0.28	2
	Wogonin	5,281,703	284.26	2.59	2	5	30.68	0.79	0.23	2
*Angelica gigas*	Marmesin	334,704	246.26	2.03	1	4	50.28	0.52	0.18	1
*Trichosanthes Kirilowii Maximowicz*	Chrysoeriol	5,280,666	300.26	2.32	3	6	35.85	0.39	0.27	2
	Diosmetin	5,281,612	300.26	2.32	3	6	31.14	0.46	0.27	2
	Luteolin	5,280,445	286.24	2.07	4	6	36.16	0.19	0.25	1
	7-O-Methylluteolin	5,318,214	300.26	2.32	3	6	36.47	0.52	0.27	2

### Screening of potential targets of SH003 in NSCLC

**Fig. 2:**
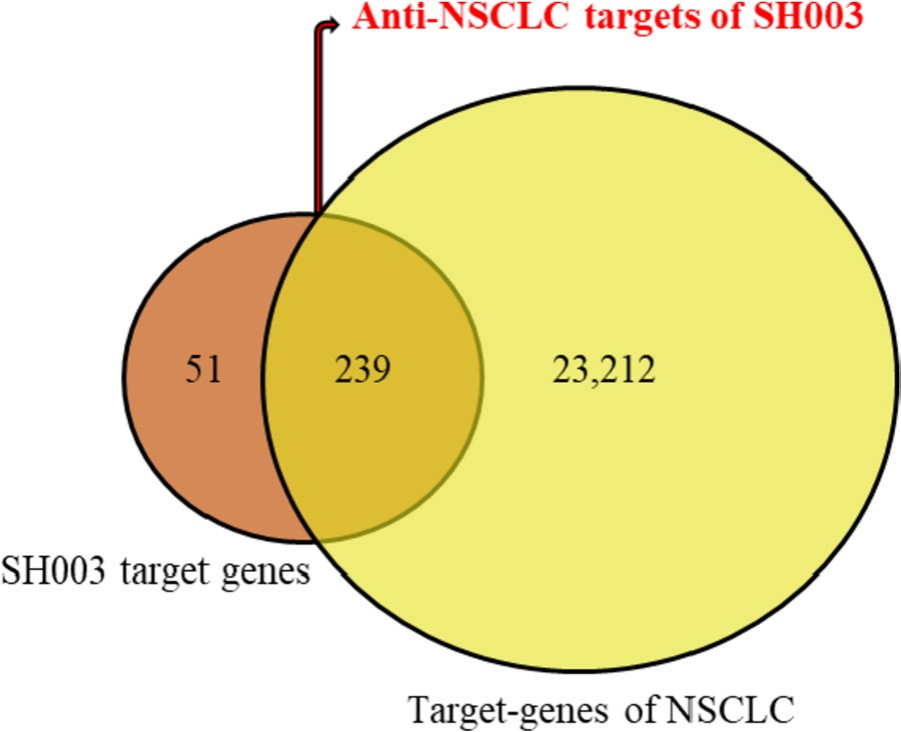
Venn diagram of the potential anti-NSCLC targets of SH003

The targets of each active ingredient were collected from PharmMapper, SwissTargetPrediction and STITCH. The sum of SH003-related targets from 20 active compounds was 290 genes (Table S2). Next, targets of NSCLC were retrieved from CTD, DisGeNET and GeneCards. From 23,088, 3,926 and 949 genes gathered from CTD, DisGeNET and GeneCards, respectively, total 23,451 genes were identified as targets of NSCLC (Table S3). As shown in the Venn diagram in Fig. 2, 239 intersecting genes were obtained as the potential anti-NSCLC targets of SH003.

### PPI network of target genes for SH003 against NSCLC

In total, 239 target genes were conducted to STRING analysis with the species limited to ‘Homo sapiens’ and the high confidence score > 0.7. According to the results of the STRING analysis, PPI networks contained 239 nodes and 1,720 edges (Fig. S2). The interaction network between 239 targets was analyzed in Cytoscape. The median value for Degree, Betweenness Centrality and Closeness Centrality was 20, 0.00156 and 0.38889, respectively. Finally, we found 79 targets as key targets of SH003 against NSCLC. A list of 79 key targets with the detailed information was shown in Table 2. *TP53*, *JUN*, *AKT1*, *STAT3* and *MAPK3* were found to be the top five key targets of the PPI network with the higher Degree, Betweenness Centrality and Closeness Centrality (Table 2).

**Table 2:** The 79 core targets in PPI network of SH003 in NSCLC

No	Name	Degree	Betweenness Centrality	Closeness Centrality
1	TP53	162	0.10566	0.54523
2	JUN	138	0.07476	0.53846
3	AKT1	138	0.05411	0.53056
4	STAT3	130	0.03249	0.51544
5	MAPK3	130	0.04915	0.52670
6	HSP90AA1	118	0.04699	0.51300
7	MAPK1	118	0.03560	0.51059
8	SRC	116	0.03600	0.50820
9	MYC	110	0.02469	0.49543
10	IL6	100	0.03790	0.49206
11	EGFR	100	0.04443	0.50115
12	CASP3	96	0.02424	0.48984
13	RELA	94	0.02887	0.49206
14	KRAS	92	0.01626	0.46667
15	ESR1	92	0.04012	0.49543
16	VEGFA	84	0.01507	0.48009
17	CCND1	84	0.00941	0.46269
18	MAPK8	78	0.01431	0.47903
19	IL1B	76	0.02354	0.46767
20	MAPK14	74	0.00708	0.45975
21	INS	74	0.02426	0.47797
22	FOS	72	0.01245	0.45877
23	NFKBIA	72	0.01884	0.48009
24	NFKB1	68	0.00743	0.45021
25	MTOR	66	0.00600	0.45493
26	FN1	64	0.01797	0.42970
27	STAT1	62	0.00371	0.45975
28	IGF1	62	0.00898	0.44286
29	CDK1	58	0.01181	0.43927
30	CDK2	58	0.00870	0.43313
31	FOXO1	58	0.00980	0.45114
32	PPARG	58	0.01106	0.46567
33	ERBB2	56	0.01331	0.46467
34	IRS1	56	0.00310	0.44016
35	TLR4	54	0.00806	0.44835
36	MMP9	54	0.01797	0.45781
37	PTGS2	54	0.05750	0.47797
38	BCL2L1	54	0.00259	0.43056
39	CDH1	54	0.01497	0.43662
40	RB1	52	0.00502	0.44650
41	PRKCD	50	0.00157	0.42801
42	NOTCH1	50	0.00650	0.44376
43	MAP2K1	50	0.00621	0.43487
44	RXRA	50	0.05300	0.46368
45	PTK2	48	0.00349	0.42970
46	PRKCA	48	0.01431	0.44467
47	CDK4	48	0.00353	0.42136
48	CASP8	48	0.00297	0.43141
49	GSK3B	48	0.00250	0.43227
50	CCL2	48	0.01312	0.42717
51	IGF1R	46	0.00169	0.43400
52	CHUK	46	0.00306	0.42633
53	CCNB1	46	0.00326	0.40185
54	APP	46	0.02875	0.43056
55	NOS3	44	0.01364	0.44106
56	SMAD2	42	0.00729	0.43662
57	PDPK1	40	0.00211	0.41333
58	NOS2	40	0.00419	0.44650
59	CCNA2	40	0.00202	0.39599
60	CDK6	38	0.00225	0.41333
61	H2AFX	38	0.00236	0.40789
62	CDK5	38	0.00274	0.41333
63	ESR2	38	0.00615	0.44742
64	ATM	38	0.00176	0.41176
65	MMP2	38	0.00342	0.41098
66	PPARA	38	0.03269	0.45877
67	NFATC1	36	0.00232	0.40561
68	HMOX1	36	0.01348	0.43141
69	PARP1	34	0.01929	0.41333
70	CAT	34	0.01341	0.43056
71	EZH2	30	0.00241	0.43487
72	HSPA8	30	0.00507	0.43662
73	NFE2L2	28	0.00473	0.42300
74	KEAP1	26	0.00424	0.42136
75	AHR	26	0.01433	0.42218
76	AURKA	24	0.00195	0.39599
77	GSTP1	24	0.02367	0.40789
78	NR1I2	24	0.02153	0.43056
79	HMGB1	22	0.00158	0.41021

### Screening of main active compounds for SH003 against NSCLC

**Fig. 3:**
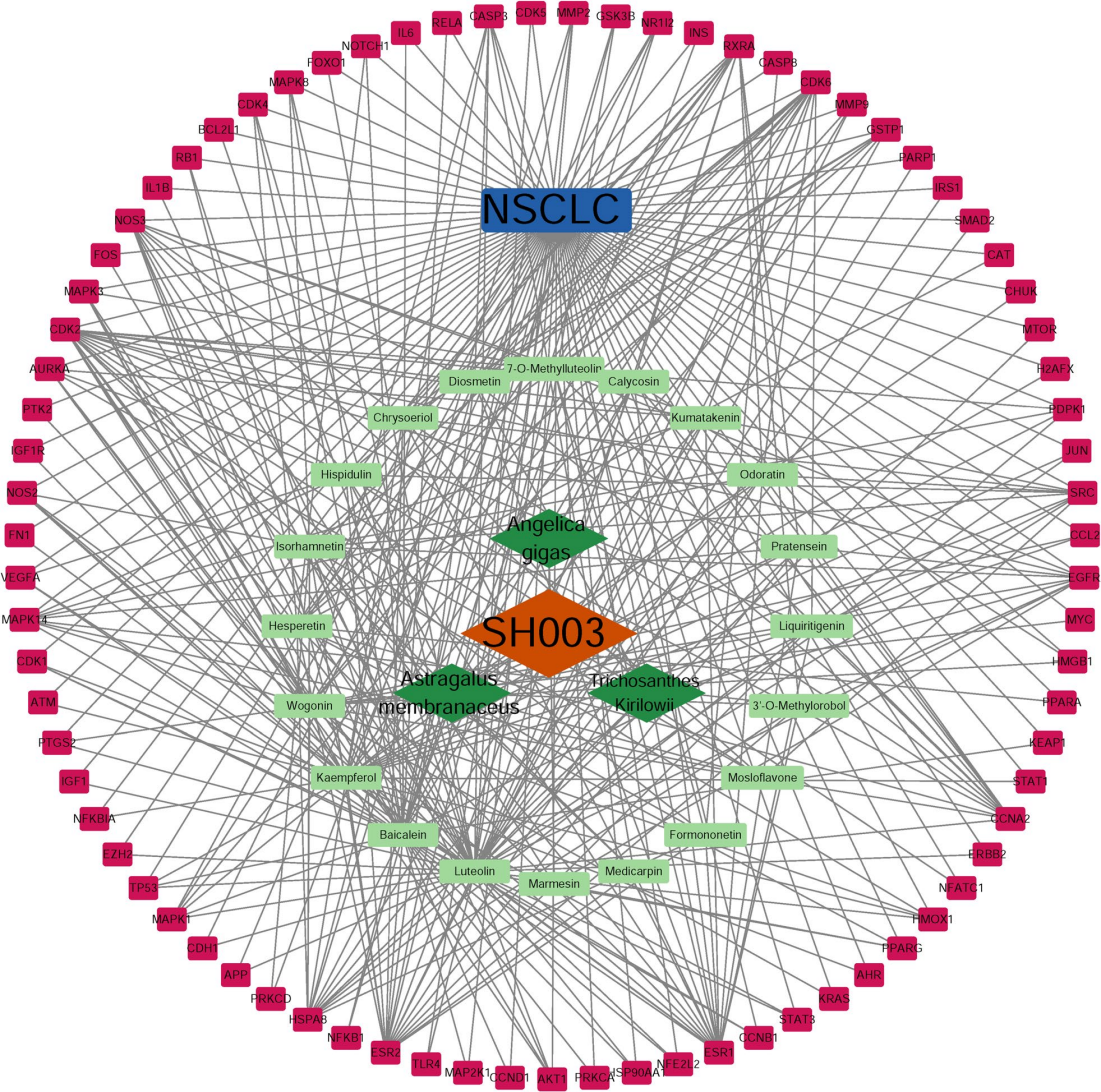
Construction of Herb-Compound-Target network of SH003 against NSCLC. The blue rectangles green diamonds, light green rectangles and purple rectangles indicate disease, herbs, compounds and disease target genes, respectively (NDEx DOI: https://doi.org/10.18119/N9KP5V)

The network of herb-compounds-targets was constructed by using Cytoscape and further analysis was performed to elucidate the main active compounds of SH003 for targeting NSCLC. As shown in Fig. 3, the blue rectangles, green diamonds, light green rectangles and purple rectangles indicate disease, herbs, compounds and disease target genes, respectively. The gray line showed the connection of disease-target genes, SH003-herbs, herbs-compounds and compounds-target genes. Active compounds were ranked for their degree and listed in Table 3. In our approach, a total of 8 active compounds was found in the network of compounds-targets to have the median of > 10 degrees, which include luteolin, baicalein, kaempferol, wogonin, hesperetin, isorhamnetin, hispidulin, and Chrysoeriol. We suggest that these selected phytochemicals may be active compounds of SH003 for NSCLC treatment (Table 3).

**Table 3:** Degree value of SH003 active compounds

No	Name	Degree
1	Luteolin	48
2	Baicalein	34
3	Kaempferol	31
4	Wogonin	21
5	Hesperetin	15
6	Isorhamnetin	14
7	Hispidulin	12
8	Chrysoeriol	11
9	Diosmetin	10
10	Calycosin	10
11	7-O-Methylluteolin	10
12	Kumatakenin	9
13	Odoratin	9
14	Pratensein	8
15	Liquiritigenin	7
16	Mosloflavone	6
17	Formononetin	6
18	3'-O-Methylorobol	6
19	Medicarpin	4
20	Marmesin	3

### GO gene enrichment and KEGG pathway analysis

**Fig. 4:**
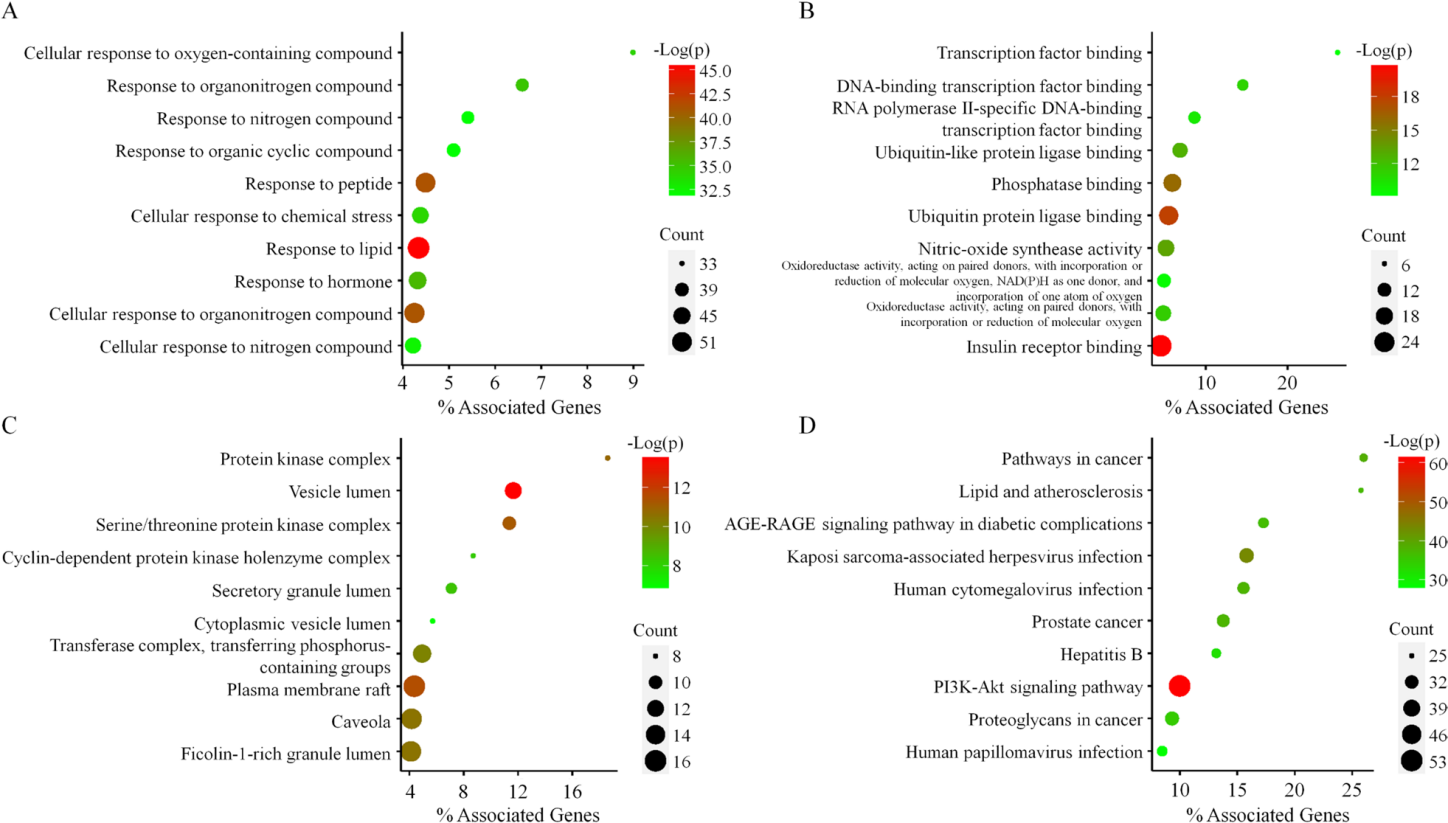
GO and KEGG analysis. **A** Biological Process **B** Molecular Function **C** Cellular Component **D** KEGG. The enriched GO and KEGG terms are depicted on the Y-axis, while the X-axis denotes “% Associated Genes”, representing the percentage of genes within the specified GO or KEGG term. Dot size indicates the number of genes associated with specific terms, and the color of the dots corresponds to the –Log(*P* value)

To verify the biological characteristics of the selected 79 targets in NSCLC, we further performed the GO enrichment analysis by using ClueGO 2.5.8, based on BP, MF and CC (Table S4) with *p* value < 0.001. In the Fig. 4, the GO analysis results represented the PPI network-related functions. The BP results of GO analysis included ‘cellular response to oxygen-containing compound’, ‘response to organonitrogen compound’, ‘response to nitrogen compound’, ‘response to organic cyclic compound’ and ‘response to peptide’ (Fig. 4A). The MF results included ‘transcription factor binding’, ‘DNA-binding transcription factor binding’, ‘RNA polymerase II-specific DNA-binding transcription factor binding’, ‘ubiquitin-like protein ligase binding’ and ‘phosphatase binding’ (Fig. 4B). The CC results contained ‘protein kinase complex’, ‘vesicle lumen’, ‘serine/threonine protein kinase complex’, ‘cyclin-dependent protein kinase holoenzyme complex’, ‘secretory granule lumen’ (Fig. 4C). The results from GO analysis suggest that SH003 could treat NSCLC from a genetic perspective with multiple synergies.

To further identify the target pathways of SH003 against NSCLC, KEGG pathway annotation of 79 key targets was performed. Total 145 pathways significantly correlated with target genes were collected (*p* < 0.001). The enriched pathways identified according to the –log10 (*p* value corrected with Benjamini-Hochberg) were presented in Fig. 4D. The detailed results of KEGG analysis were presented in Table S4. The top 20 enriched KEGG pathways were listed in Table 4. From the results of KEGG analysis, the top enriched pathways included ‘Pathways in cancer’, ‘Lipid and atherosclerosis’, ‘PI3K-Akt signaling pathway’, ‘Human cytomegalovirus infection’, ‘Kaposi sarcoma-associated herpesvirus infection’, ‘Hepatitis B’, ‘Hepatitis B’, ‘Proteoglycans in cancer’, ‘AGE-RAGE signaling pathway in diabetic complications’, ‘Prostate cancer’, ‘MAPK signaling pathway’, ‘Cellular senescence’ and so on, suggesting that the enriched pathways from KEGG are likely to be related to the anticancer mechanism of SH003 in NSCLC.

**Table 4:** Top 20 enriched KEGG pathways with 79 key targets of SH003 in NSCLC

ID	Pathway Name	Term *P*Value Corrected with Benjamini-Hochberg	Gene Count
KEGG:05200	Pathways in cancer	5.04E-62	53
KEGG:05417	Lipid and atherosclerosis	3.60E-44	34
KEGG:04933	PI3K-Akt signaling pathway	1.95E-35	33
KEGG:05167	Human cytomegalovirus infection	2.62E-38	31
KEGG:05163	Kaposi sarcoma-associated herpesvirus infection	1.46E-38	30
KEGG:05215	Hepatitis B	2.65E-37	28
KEGG:05161	Human papillomavirus infection	1.01E-28	28
KEGG:04151	Proteoglycans in cancer	9.96E-33	27
KEGG:05212	AGE-RAGE signaling pathway in diabetic complications	2.55E-39	26
KEGG:05205	Prostate cancer	9.13E-38	25
KEGG:04218	MAPK signaling pathway	1.12E-25	25
KEGG:05160	Cellular senescence	1.08E-30	24
KEGG:05162	Hepatitis C	1.22E-30	24
KEGG:04068	Human immunodeficiency virus 1 infection	1.70E-27	24
KEGG:05165	Human T-cell leukemia virus 1 infection	3.42E-27	24
KEGG:05418	Measles	3.77E-30	23
KEGG:05224	MicroRNAs in cancer	2.72E-22	23
KEGG:05219	Pancreatic cancer	1.74E-34	22
KEGG:05170	FoxO signaling pathway	5.74E-29	22
KEGG:04926	Fluid shear stress and atherosclerosis	2.17E-28	22

### Cytotoxic effect of SH003 on NSCLC cell lines

**Fig. 5:**
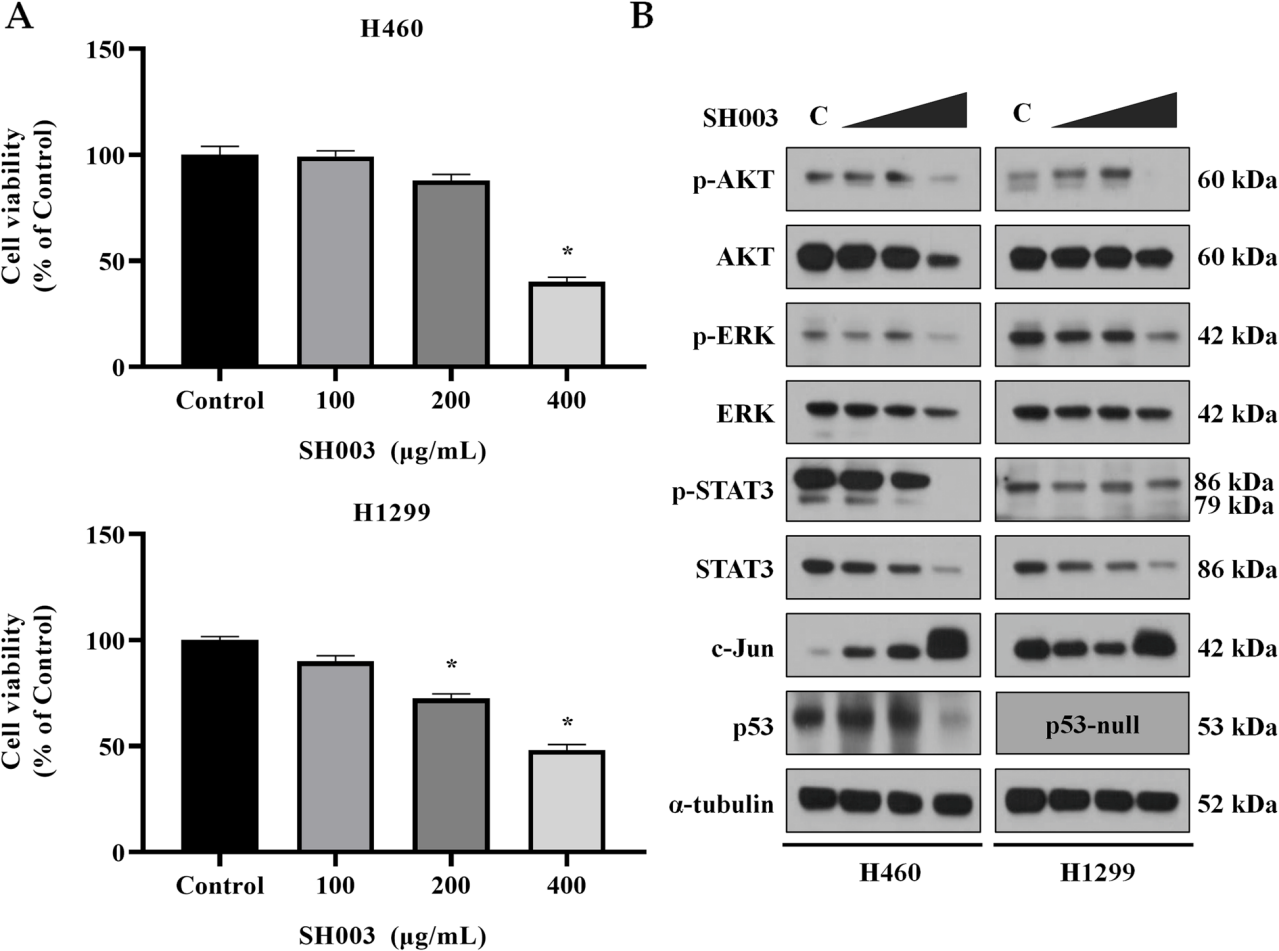
In vitro validation of the network pharmacology analysis. **A** Cell viability of H460 and H1299 cell lines after SH003 treatment for 24 h was measured using the MTT assay. The data are shown as the means ± SEM, three individual experiments, *p < 0.05 compared to Control. **B** The representative western blot image of key targets in H460 and H1299 cell lines after SH003 treatments (C: Control, 0.1% DMSO; SH003: 100, 200, 400 μg/mL). Experiments were performed in triplicate

We examined the cytotoxic effect of SH003 on H460 and H1299 cell lines, which have different p53 status [61]. SH003 treatment dose-dependently inhibited the viability of H460 and H1299 cell lines (Fig. 5A). The IC50 values for H460 and H1299 were 378.3 μg/mL and 383.6 μg/mL, respectively.

### Target validation by western blot analysis

We performed western blot analysis to validate the regulatory effect of SH003 on top five hub targets. SH003 treatment differentially regulated the expression of the hub targets including p53, c-Jun, STAT3, ERK and AKT, suggesting that these expression patterns affected by SH003 appeared to be unique to H460 and H1299 cell lines (Fig. 5B and Fig. S1).

### The validation of active compounds in SH003

**Fig. 6:**
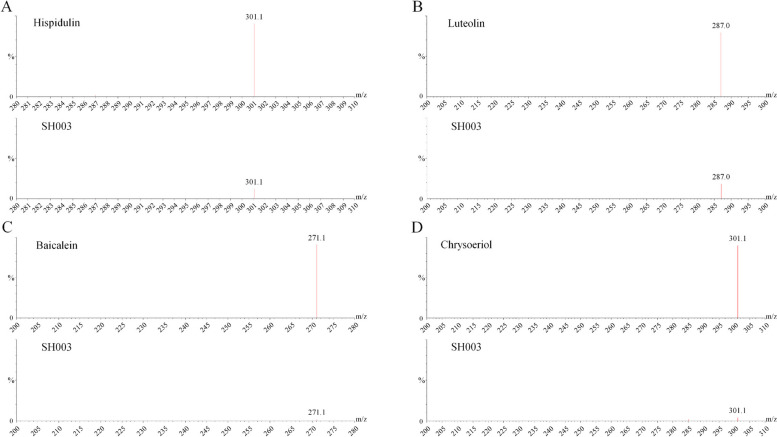
The mass spectra of four active components in SH003. **A** hispidulin **B** Luteolin **C** Baicalein **D** Chrysoeriol

We conducted LC–MS experiments to validate the eight active compounds of SH003 identified through current network pharmacology analysis. MS/MS confirmation was achieved for the standard compounds Chrysoriol (301.1 > 286.0 m/z), Isorhamnetin (317.0 > 153.0 m/z), Hispidulin (301.1 > 286.0 m/z), Kaempferol (287.0 > 89.0 m/z), Luteolin (287.0 > 121.0 m/z), Wogonin (285.1 > 270.0 m/z), Bicalein (271.1 > 123.0 m/z), and Hesperetin (301.0 > 164.0 m/z). Notably, our findings revealed that only four compounds—hispidulin, luteolin, baicalein, and chrysoeriol—were identified in the SH003 extract at matching MS and retention time (RT), while the others were not detected (refer to Fig. 6 and Fig. S3).

### Network pharmacology analysis for actually existing active compounds in SH003

**Fig. 7:**
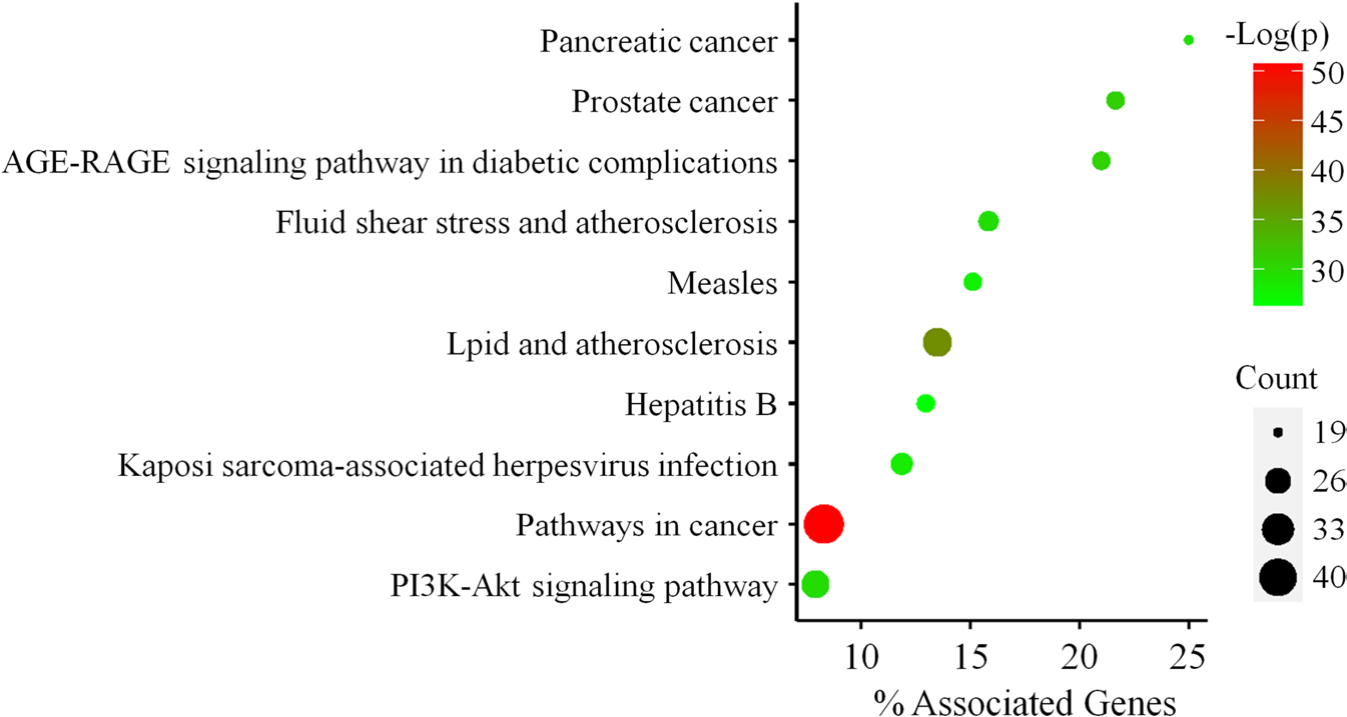
Analysis of the 64 key targets associated with the 4 validated active compounds of SH003 using KEGG. The enriched KEGG terms are depicted on the Y-axis, while the X-axis denotes “% Associated Genes”, representing the percentage of genes within the specified KEGG term. Dot size indicates the number of genes associated with specific terms, and the color of the dots corresponds to the -Log(*P* value)

Following the validation of the four active compounds in SH003, additional network pharmacological analysis was conducted. This involved PPI analysis to identify genuine key targets, followed by GO and KEGG analysis. Utilizing Cytoscape to analyze the interaction network, we identified 64 key targets related to the four SH003 compounds (Table 5). The results of the GO analysis encompassed terms such as “cellular response to chemical stress,” “cellular response to nitrogen compound,” and “response to oxidative stress” (Table S5). Furthermore, the KEGG analysis highlighted significant pathways including “Pathways in cancer,” “Lipid and atherosclerosis,” and “PI3K-Akt signaling pathway” (Fig. 7). The detailed results of GO and KEGG analysis were presented in Table S5.

**Table 5:** The 64 key targets in the PPI network for the validated four active compounds from SH003 in NSCLC

No	Name	Degree	Betweenness Centrality	Closeness Centrality
1	TP53	148	0.15135	0.59316
2	AKT1	114	0.06646	0.56934
3	JUN	108	0.05402	0.55714
4	IL6	104	0.05594	0.53793
5	STAT3	102	0.04319	0.54545
6	EGFR	100	0.04684	0.55319
7	ESR1	88	0.07330	0.54355
8	MAPK3	86	0.02919	0.52000
9	CASP3	84	0.02055	0.50814
10	IL1B	82	0.03133	0.51656
11	SRC	82	0.01311	0.51656
12	NFKB1	82	0.02687	0.52349
13	CCND1	82	0.03380	0.52525
14	HSP90AA1	82	0.03791	0.50000
15	FN1	78	0.04886	0.49057
16	MAPK1	76	0.02645	0.51148
17	MAPK8	72	0.01777	0.50649
18	INS	70	0.04570	0.50000
19	FOS	62	0.01344	0.48750
20	MAPK14	60	0.00913	0.48148
21	PPARG	60	0.02096	0.49057
22	ERBB2	58	0.03464	0.50323
23	MMP9	58	0.00992	0.48297
24	MTOR	58	0.00824	0.48148
25	PTGS2	58	0.03742	0.48148
26	GSK3B	56	0.00732	0.47273
27	FOXO1	56	0.01969	0.48750
28	IGF1	56	0.00684	0.47130
29	RELA	56	0.02063	0.48447
30	TLR4	52	0.01024	0.47706
31	BCL2L1	52	0.00407	0.46847
32	PARP1	48	0.02031	0.46018
33	CDK2	46	0.00983	0.44193
34	IRS1	44	0.00280	0.46988
35	CASP9	44	0.00367	0.45748
36	APP	44	0.04140	0.45882
37	CCNB1	42	0.00851	0.43575
38	CDH1	42	0.00204	0.45748
39	CDK1	42	0.00827	0.43454
40	MAPK9	42	0.00432	0.44571
41	NFE2L2	40	0.01331	0.46018
42	MMP2	38	0.00419	0.44193
43	CDK4	38	0.00434	0.44068
44	MCL1	34	0.01074	0.43454
45	MAP2K1	34	0.00177	0.44068
46	SMAD2	32	0.00761	0.45087
47	KDR	32	0.00333	0.43697
48	PTK2	32	0.00252	0.44571
49	ESR2	32	0.00523	0.45087
50	HMOX1	30	0.00648	0.44068
51	CCNA2	30	0.00192	0.41270
52	MMP3	28	0.00523	0.40206
53	AURKA	28	0.00435	0.43094
54	CYP1A1	28	0.04861	0.39898
55	NOS3	26	0.00416	0.42507
56	CYP19A1	26	0.06300	0.42276
57	HSPA8	26	0.01106	0.44699
58	CHEK1	26	0.00177	0.40206
59	AGT	24	0.00746	0.42391
60	CDK5	22	0.00507	0.42049
61	CDK6	22	0.00184	0.41711
62	KEAP1	20	0.00489	0.43213
63	GSTP1	20	0.02364	0.41935
64	NQO1	18	0.01164	0.43213

## Discussion

NSCLC remains a leading cause of cancer-related deaths globally [2]. Herbal medicines are being considered as potential treatments for NSCLC due to their cytotoxic effects on cancer cells, regulatory effects on the tumor microenvironment, and minimal side effects [8–11]. A novel herbal prescription SH003 has been developed for treatment of several cancers [12–21, 24–26, 62]. To date, a network pharmacological analysis has widely been used to understand the therapeutic mechanisms of traditional herbal medicine in several diseases including cancer [28–36]. This systemic methodology is beneficial for the identification of the relationship between herbal ingredients and disease, based on the multi-targeted therapy [27]. As SH003 contains multiple components with multiple targets and pathways, this study provides the first insight into the potential active compounds and molecular targets of SH003 for the treatment of NSCLC using a network pharmacological approach.

Total 965 compounds in SH003 were screened for their ADME criteria and thus 15 compounds of AM, one compound of AG and four compounds of TK were identified for further analysis. After collecting 23,451 disease targets from web databases, the study ultimately identified 79 key targets by considering their median value of topological parameters such as degree, betweenness centrality and closeness centrality. The network analysis of 79 key targets of SH003 in NSCLC treatment revealed *TP53*, *JUN*, *AKT1*, *STAT3*, and *MAPK3* as the top five key targets with the higher value of degree, betweenness centrality, closeness centrality. The functional enrichment analysis showed that the 79 key targets were enriched in various signaling pathways, including the ‘PI3K-Akt signaling pathway’, which was further verified through in vitro experiments to confirm the modulatory effect of SH003 on these targets and pathways. Furthermore, MTT assay substantiated prior research findings regarding SH003's anti-NSCLC properties [23].

The top five key genes identified from the PPI analysis, namely *TP53*, *JUN*, *AKT1*, *STAT3*, and *MAPK3*, are crucial targets not only in NSCLC but also in various other cancers [63–67]. Among them, STAT3 plays a crucial role in tumor proliferation, differentiation, survival, immunosuppression, angiogenesis and tumorigenesis [68–70]. It has been reported that abnormal expression of STAT3 has significant correlation with poor overall survival of cancer patients [71–79]. Notably, SH003 suppresses tumor growth and metastasis of triple-negative breast cancer MDA-MB-231 cell lines by down-regulation of STAT3-IL6 signaling loop [12]. SH003 also induces autophagy via inhibiting STAT3 activation while sustained activation of STAT3 weakens SH003-induced autophagy in breast cancer cell [18]. Moreover, SH003 promotes autophagy-mediated cell death of gastric cancer cells via activating ATF4 and inhibiting G9a under hypoxia [20]. A recent study from our group has demonstrated that SH003 effectively inhibits the growth of NSCLC cell lines by suppressing STAT3 activation [21]. We also revealed that SH003 prevented docetaxel-induced peripheral neuropathy with inhibition of phospho-STAT3 at the sciatic nerves and spinal cords (L4 – L6), which is one of the readouts for chemotherapy-induced peripheral neuropathy [62]. Therefore, our findings in the present study are consistent with the results from previous studies on SH003. Next, our results further indicate that SH003 treatment downregulates p53 expression while upregulating c-jun expression. Accumulating studies have supported that p53 and c-JUN are closely associated and these proteins have double-edged sword roles in cancer [80, 81]. DNA damage triggers p53 expression, resulting in promoting apoptosis in damaged cells [67, 82]. In cancer, suppression of p53 by AP-1 contributes to cell proliferation, drug resistance and metastasis [67, 80–82]. In contrast, some researchers have reported the opposite results that c-jun mediates apoptotic death of cancer cells through alternative pathways including p73 stabilization-induced apoptosis [83–86]. Previous study demonstrated that SH003 induces p73-dependent apoptosis in triple-negative breast cancer cells [13]. Therefore, the results suggest that SH003 has multiple targets for NSCLC treatment, and further investigation is needed to understand the diverse and heterogeneous functions of key proteins in different NSCLC cancer cell lines.

Numerous studies have shown that the activation of the PI3K-Akt pathway plays a role in various cellular processes, such as cancer cell proliferation, differentiation, metastasis, and drug resistance [66]. The present study identified the PI3K-Akt signaling pathway as one of the top enriched target pathways of SH003 for NSCLC treatment using KEGG pathway analysis. Our previous studies demonstrated that SH003 treatment induces apoptotic and autophagic cell death of cancer cell lines by targeting PI3K-Akt signaling pathway and their downstream signaling including STAT3 and mTOR [12, 14, 17, 20]. Moreover, recent study demonstrated that SH003 inhibits the growth of NSCLC cell lines via inhibition of EGFR and STAT3 [21]. Taken together, our data supported the possibility that PI3K-Akt may be a crucial target of SH003 for NSCLC treatment and Therefore, our data suggest that the PI3K-Akt pathway may be a crucial target of SH003 for NSCLC treatment and further investigations are necessary to confirm the pharmacological mechanisms of SH003 and its key compounds.

The literatures were reviewed for eight key active compounds, namely luteolin, baicalein, kaempferol, wogonin, hesperetin, isorhamnetin, hispidulin and chrysoeriol. Anti-NSCLC effect of these active compounds has been well documented. Luteolin-mediated ER stress, apoptotic and autophagic cell death are associated with multiple targets including EGFR, LIMK1, DR5, PDK1, NF-кB, MEK/ERK, STAT3/IL-6 and PI3K/Akt signaling pathway [87–98]. Moreover, luteolin suppresses the migration and invasion of NSCLC cell lines via suppressing several targets such as PI3K/AKT/NF-кB, EGFR and so on [90, 99–102]. Recently, Jiang et al. demonstrated that luteolin improves anti-tumor immunity in KRAS-mutant lung cancer by suppressing PD-L1 expression [103]. Baicalein induces apoptosis and autophagy in NSCLC via targeting PI3K/Akt/NF-кB pathway, AMPK signaling pathway, Notch signaling pathway and so on [104–107]. Kaempferol inhibits the growth, migration and invasion of NSCLC cell lines by regulation of STAT3, MEK/MAPK and PI3K/Akt signaling pathway [92, 108–112]. Besides, wogonin [113–118], hesperetin [119–123], isorhamnetin [124–127], hispidulin [128] and chrysoeriol [129] exhibit anti-NSCLC effect with a diverse mode of action while more studies are necessary to provide a deeper insight into the anti-NSCLC effect and molecular mechanism of each compound.

In addition to the aforementioned KEGG pathways, our results revealed other cancer-related pathways such as ‘Proteoglycans in cancer’, ‘MAPK signaling pathway’, ‘MicroRNAs in cancer’, ‘FoxO signaling pathway’, Relaxin signaling pathway’, HIF-1 signaling pathway’, ‘IL-17 signaling pathway’, ‘TNF signaling pathway’, ‘Apoptosis’, ‘Prolactin signaling pathway’,, ‘PD-L1 expression and PD-1 checkpoint pathway’ and so on (Table S4). These multiple pathways play a crucial role in cancer development and progression, indicating that SH003's anti-NSCLC effect can be achieved by targeting multiple signaling pathways simultaneously. Furthermore, the KEGG results suggest that SH003 may have potential for treating various types of cancers, such as prostate cancer, pancreatic cancer, breast cancer, small cell lung cancer, gastric cancer, bladder cancer, leukemia, colorectal cancer, glioma, melanoma, among others (Table S4). While our previous study reported the anti-cancer effect of SH003 against breast cancer, NSCLC, prostate cancer and gastric cancer [12, 13, 15–21], the present study hypothesized that the indication of SH003 can be expanded to various cancers while further experimental validation for anti-cancer effect and molecular mechanisms is needed.

Immune checkpoint inhibitors have emerged as a primary therapeutic option for NSCLC patients with PD-L1 positivity and EGFR/ALK wild-type [130–134]. Despite their potential benefits in harnessing the body’s immune system to fight cancer, low response rates and systemic adverse effects remain a concern [135–138]. Our study conducted KEGG analysis and revealed the potential effect of SH003 on the PD-L1/PD-1 pathway (Table S4). A previous study by Han et al. reported that SH003 improves immunosuppression by activating macrophages, splenocytes, and NK cells [139]. Additionally, our preliminary data showed that SH003 reduces the expression of PD-L1 in NSCLC cell lines without interfering with the binding of PD-L1 to PD-1 (unpublished data). Based on these findings, we propose that SH003 could serve as a promising therapeutic option for improving the response rate of current immunotherapy and enhancing the quality of life for NSCLC patients.

In our present study, LC–MS analysis definitively confirmed the presence of luteolin, baicalein, hispidulin, and chrysoeriol within SH003, signifying their potential as vital anti-cancer components. While theoretical models hinted at the anti-cancer effects of all eight compounds through intricate molecular interactions, our experimental validation pinpointed only these four validated oral active compounds as the true anti-cancer agents in SH003. Acknowledging the existing gaps in research on the therapeutic efficacy and mechanisms of these compounds in NSCLC, we stress the urgency of further research to unveil the synergistic molecular pathways through which luteolin, baicalein, hispidulin, and chrysoeriol exert their anti-NSCLC effects. Additionally, conducting in vivo experiments is essential to enhance our comprehension of their efficacy and safety, bridging the gap between laboratory discoveries and potential clinical applications. Our study delved into diverse network pharmacology approaches, considering experimental validation and predictions from literature or structure-based methods. A common challenge lies in validating active compounds identified through network pharmacology, particularly regarding their presence in the actual plant extract, significantly influenced by extraction conditions. Our focus was to identify the genuine active compounds within SH003 using LC–MS analysis. Out of the initial eight compounds identified through network pharmacology, only four were experimentally validated, underscoring the potential variability in multi-pathways and multi-targets depending on different databases and extraction methods. Revisiting our network pharmacology analysis, we uncovered new key targets and pathway enrichment patterns. Previously unexplored targets such as *CASP9*, *MAPK9*, *MCL1*, and others, absent from the initial list of 79 targets, were discovered and supported by recent publications [140–144]. The PI3K-AKT pathway’s significance in NSCLC was reaffirmed, and differences in GO terms and KEGG pathways enrichment patterns, including ‘response to xenobiotic stimulus’ and ‘Alcoholic liver disease’, hinted at SH003’s extensive therapeutic potential. We anticipate our findings to be a pivotal reference for researchers in the field, planning further experimental validations of these new targets, expanding our exploration into novel therapeutic avenues.

## Conclusion

In summary, our study successfully validated the theoretical predictions derived from online databases through comprehensive analyses. Initially, database-based network pharmacology against NSCLC identified 79 key targets, multiple pathways, and eight active compounds within SH003. Subsequent rigorous LC–MS analysis revealed the presence of only four active compounds in SH003, emphasizing the possible impact of extraction methods on the composition of herbal formulations. Further network pharmacology analyses specifically focusing on these four compounds uncovered 64 key targets and intricate pathways related to NSCLC. This study underscores the critical need to confirm the presence of active compounds identified through network pharmacology in actual herbal formulations, aligning theoretical predictions with empirical evidence. We believe our findings will serve as a valuable reference, highlighting the importance of integrating theoretical predictions with experimental validation for researchers engaged in network pharmacology studies. Finally, we recommend the application of network pharmacology for the systematic analysis of natural products, as it can provide a comprehensive understanding of their therapeutic potential and multiple mechanisms of action. Our study demonstrates the utility of this approach in identifying promising compounds for further investigation as potential anti-cancer agents.

### Supplementary Information

**Additional file 1:** **Figure S1.** Expression of key targets in H460 and H1299 cell lines after SH003 treatments (C: Control, 0.1% DMSO; SH003: 100, 200, 400 ug/mL). We performed three independent experiments. The western blot bands were blotted using the same PVDF membrane for each experiment, following the manufacturer’s protocol for repeated antibody stripping with RestoreTM PLUS Western Blot Stripping Buffer. **Figure S2.** Protein-protein interaction network of identified anti-NSCLC targets of SH003. **Figure S3.** The mass spectra of the four components not detected in SH003: (A) Kaempferol, (B) Wogonin, (C) Isorhamnetin, and (D) Hesperetin. **Table S1.** Compounds in SH003. **Table S2.** Potential targets of SH003. **Table S3.** NSCLC-related genes. **Table S4.** GO Terms and KEGG pathways associated with the 79 identified key targets. **Table S5.** GO Terms and KEGG pathways associated with the 64 identified key targets.

## Data Availability

The datasets generated and/or analysed during the current study are available in the following repository: [TM-MC (https://informatics.kiom.re.kr/compound/), OASIS (https://oasis.kiom.re.kr/), TCMSP (https://tcmsp-e.com/index.php), PharmMapper (http://lilab-ecust.cn/pharmmapper), SwissTargetPrediction (http://swisstargetprediction.ch), STITCH (http://stitch.embl.de), UniProt (https://www.uniprot.org/), CTD (http://ctdbase.org),, DisGeNET (http://www.disgenet.org), GeneCards (http://www.genecards.org), STRING 11.0 (http://string-db.org)].

## Abbreviations

NSCLC: Non-Small Cell Lung Cancers
AM: Astragalus Membranaceus
AG: Angelica Gigas
TK: Trichosanthes Kirilowii Maximowicz
PPI: Protein–Protein Interaction
GO: Gene Ontology
KEGG: Kyoto Encyclopedia for Genes and Genomes
ADME: Absorption, Distribution, Metabolism and Excretion
TCMSP: Traditional Chinese Medicine Systems Pharmacology
MW: Molecular weight
Hdon: H-bond donor
Hacc: H-bond acceptor
OB: Oral bioavailability
Caco-2: Caco-2 permeability
DL: Drug-likeness
RBN: Rotatable bonds
STRING: Search Toll for the Retrieval of Interacting Genes/Proteins
BP: Biological Process
MF: Molecular Function
CC: Cellular Component
