# Supplementary material for: Network pharmacology study to explore the multiple molecular mechanism of SH003 in the treatment of non-small cell lung cancer
Source: BMC Complement Med Ther. 2024 Feb 1;24:70. doi: 10.1186/s12906-024-04347-y (PMC10832243; doi:10.1186/s12906-024-04347-y)
Supplement: Supplementary file 1 — Additional file 1: Figure S1. Expression of key targets in H460 and H1299 cell lines after SH003 treatments (C: Control, 0.1% DMSO; SH003: 100, 200, 400 ug/mL). We performed three independent experiments. The western blot bands were blotted using the same PVDF membrane for each experiment, following the manufacturer’s protocol for repeated antibody stripping with RestoreTM PLUS Western Blot Stripping Buffer. Figure S2. Protein-protein interaction network of identified anti-NSCLC targets of SH003. Figure S3. The mass spectra of the four components not detected in SH003: (A) Kaempferol, (B) Wogonin, (C) Isorhamnetin, and (D) Hesperetin. Table S1. Compounds in SH003. Table S2. Potential targets of SH003. Table S3. NSCLC-related genes. Table S4. GO Terms and KEGG pathways associated with the 79 identified key targets. Table S5. GO Terms and KEGG pathways associated with the 64 identified key targets. [file 12906_2024_4347_MOESM1_ESM.pdf]

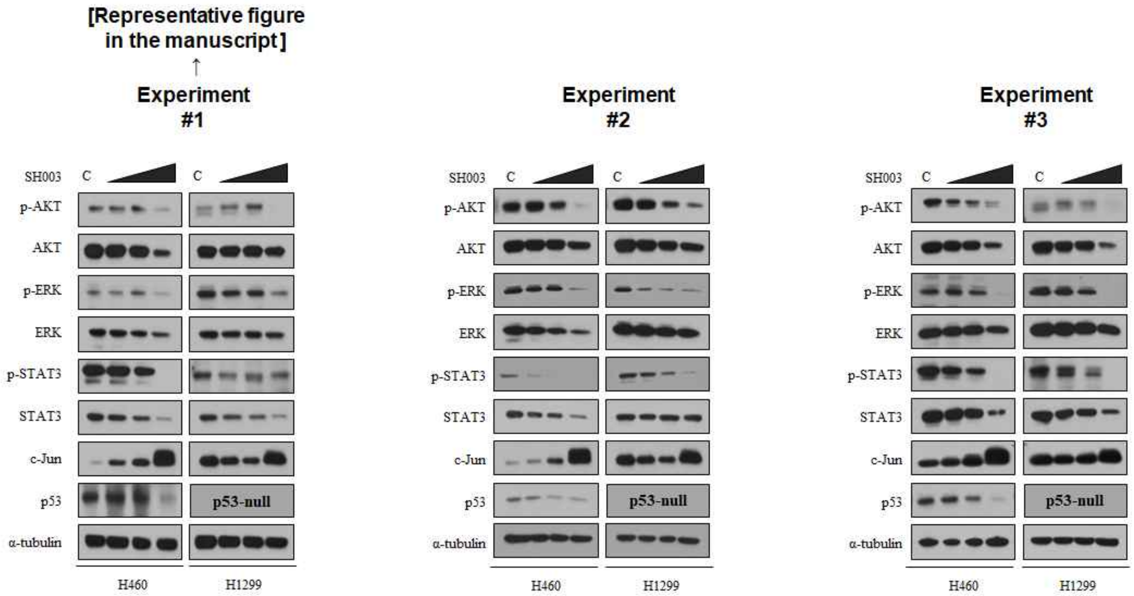

Figure S1. Expression of key targets in H460 and H1299 cell lines after SH003 treatments (C: Control, 0.1% DMSO; SH003: 100, 200, 400 ug/mL). We performed three independent experiments. The western blot bands were blotted using the same PVDF membrane for each experiment, following the manufacturer's protocol for repeated antibody stripping with Restore^{TM} PLUS Western Blot Stripping Buffer.

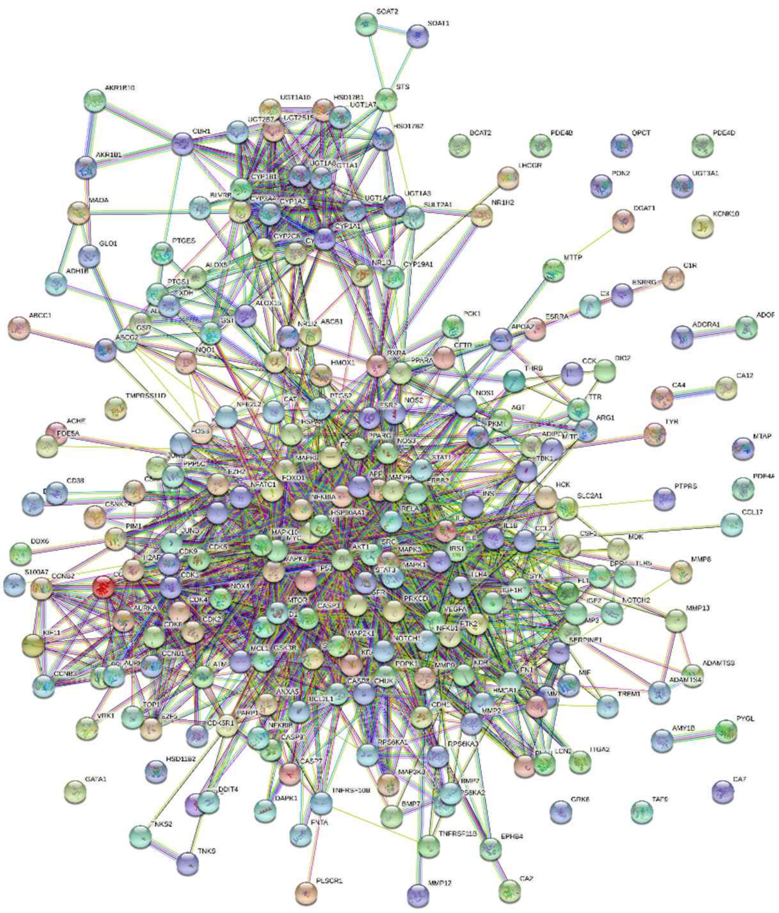

Figure S2. Protein-protein interaction network of identified anti-NSCLC targets of SH003

Figure S3

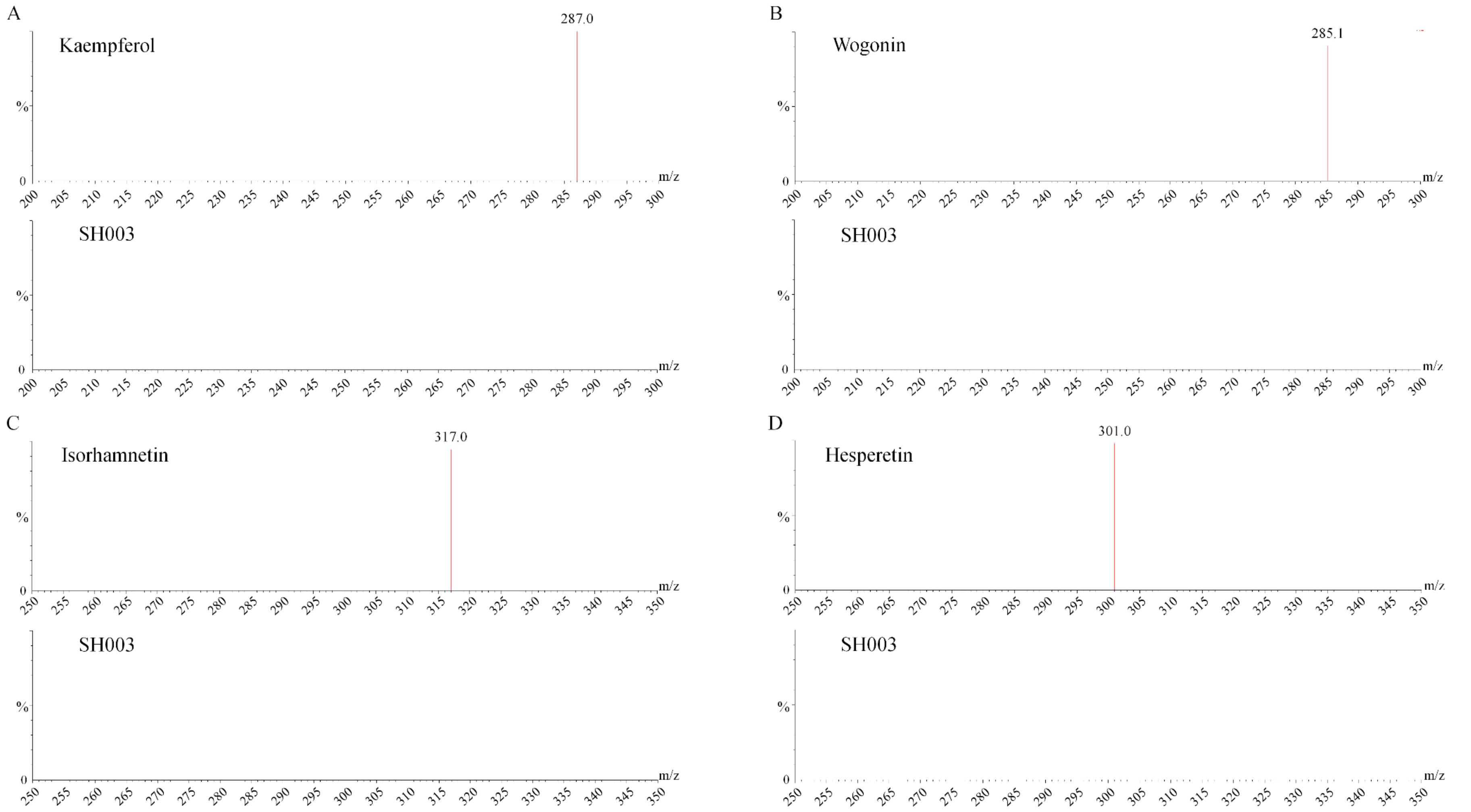

Figure S3. the mass spectra of the four components not detected in SH003: (A) Kaempferol, (B) Wogonin, (C) Isorhamnetin, and (D) Hesperetin

Table S1. Compounds in SH003

No.	Scientific name	Compound name
1	Astragalus membranaceus	(-)-methylinissolin
2	Astragalus membranaceus	(-)-methylinissolin 3-O-β-D-(6'-acetyl)-glucoside
3	Astragalus membranaceus	(-)-methylinissolin 3-O-β-D-glucoside
4	Astragalus membranaceus	(-)-methylinissolin 3-O-β-D-{6'-[(E)-but-2-enoyl]}-glucoside
5	Astragalus membranaceus	(-)-methylinissolin-3-O-β-D-glucoside
6	Astragalus membranaceus	(-)-methylnissolin
7	Astragalus membranaceus	(-)-mucronulatol
8	Astragalus membranaceus	(-)-β-sitosterol
9	Astragalus membranaceus	(3R) 8,2'-dihydroxy-7,4'-dimethoxyisoflavan
10	Astragalus membranaceus	(3R)-(-)-7,2'-dihydroxy-3',4'-dimethyl isoflavan-7-O-β-D-glucopyranoside
11	Astragalus membranaceus	(3R)-2',3'-dihydroxy-7,4'-dimethoxyisoflavan
12	Astragalus membranaceus	(3R)-2'-hydroxy-3',4'-dimethoxyisoflavan-7-O-β-D-glucoside
13	Astragalus membranaceus	(3R)-2'-hydroxy-3',4'-dimethyl-isoflavan-7-O-β-D-glucopyranoside
14	Astragalus membranaceus	(3R)-2'-hydroxy-7,3',4'-trimethoxyisoflavan
15	Astragalus membranaceus	(3R)-3',8-dihydroxyvestitol
16	Astragalus membranaceus	(3R)-7,2'-dihydroxy-3',4'-dimethoxyisoflavan
17	Astragalus membranaceus	(3R)-7,2'-dihydroxy-3',4'-dimethoxyisoflavan-7-O-β-D-glucopyranoside
18	Astragalus membranaceus	(3R)-7,2'-dihydroxy-3',4'-dimethoxyisoflavan-7-O-β-D-glucoside
19	Astragalus membranaceus	(3R)-7,2'-dihydroxy-3',4'-dimethoxyisoflavone-7-O-β-D-glycoside
20	Astragalus membranaceus	(3R)-7,2'-dihydroxy-3',4'-dimethoxyisoflavan-7-O-β-D-glucoside
21	Astragalus membranaceus	(3R)-7,2'-dihydroxy-3',4'-dimethoxyisoflavone
22	Astragalus membranaceus	(3R)-7,2-dihydroxy-3,4-dimethoxyisoflavan-7-O-β-D-glucopyranoside
23	Astragalus membranaceus	(3R)-7,2-dihydroxy-3,4-dimethoxyisoflavone
24	Astragalus membranaceus	(3R)-8,2'-dihydroxy-7,4'-dimenthoxyisoflavan
25	Astragalus membranaceus	(3R)-8,2'-dihydroxy-7,4'-dimethoxyisoflavan
26	Astragalus membranaceus	(6aR,11aR)-10-hydroxy-3,9-dimethoxypterocarpan
27	Astragalus membranaceus	
28	Astragalus membranaceus	(6aR,11aR)-3-hydroxy-9,10-dimethoxypterocarpan-3-O-β-D-glucoside
29	Astragalus membranaceus	(6aR,11aR)-3-hydroxy-9,10-dimethoxypterocarpan-3-O-β-D-glucoside-6''-O-malonate
30	Astragalus membranaceus	(6aR,11aR)-3-hydroxy-9,10-dimethoxypterocarpan-3-O-β-D-glucuronide
31	Astragalus membranaceus	(6aR,11aR)-3-hydroxy-9,10-dimethoxypterocarpan-3-O-β-D-sambubioside
32	Astragalus membranaceus	(6aR,11aR)-9,10-di-methoxypterocarpan-3-O-β-D-glucopyranoside
33	Astragalus membranaceus	(6aR,11aR)-9,10-dimethoxypterocarpan-3-O-β-D-glucopyranoside
34	Astragalus membranaceus	(6aR,11aR)-9,10-dimethoxypterocarpan-3-O-β-D-glucopyranoside-6''-acetate

35	Astragalus membranaceus	(6aR,11aR)-9,10-dimethoxypterocarpan-3-O-β-D-glucoside
36	Astragalus membranaceus	(6αR,11αR) 3-hydroxy-9,10-dimethoxypterocarpan-3-O-β-D-glucoside
37	Astragalus membranaceus	(6αR,11αR)-3-hydroxy-9,10-dimethoxypterocarpan
38	Astragalus membranaceus	(6αR,11αR)-9,10-dimethoxypterocarpan
39	Astragalus membranaceus	(6αR,11αR)-9,10-dimethoxypterocarpan-3-O-β-D-glucopyranoside
40	Astragalus membranaceus	(6αR,11αR)-9,10-dimethoxypterocarpan-3-O-β-D-glucoside
41	Astragalus membranaceus	(R)-3-(5-hydroxy-2,3,4-trimethoxyphenyl)-chroman-7-ol
42	Astragalus membranaceus	1,2-benzendicarboxylic acid diisononylester
43	Astragalus membranaceus	1,4-β-D-galactanase
44	Astragalus membranaceus	10-dihydroxy-9-methoxypterocarpan-3-O-glycoside
45	Astragalus membranaceus	10-dihydroxy-9-methoxypterocarpan-3-O-glycoside-6'-O-malonate
46	Astragalus membranaceus	10-hydroxy-3,9-dimethoxypterocarpan
47	Astragalus membranaceus	2',4'-dimethoxy-3'-hydroxyisoflavan 6-O-β-D-glucoside
48	Astragalus membranaceus	2'-dihydroxy-3',4'-dimethoxyisoflavan-7-O-β-D-glucopyranoside
49	Astragalus membranaceus	2'-hydroxy-3',4'-dimethoxy isoflavone-7-O-glycoside
50	Astragalus membranaceus	2'-hydroxy-7,3',4'-trimethoxyisoflavan
51	Astragalus membranaceus	2,3,4,5-tetrahydroxypentanal
52	Astragalus membranaceus	2-epilentiginosine
53	Astragalus membranaceus	3',6-dimethoxy-4'-hydroxyisoflavone-7-O-β-D-glucopyranoside
54	Astragalus membranaceus	3,10-dihydroxy-9-methoxypterocarpan
55	Astragalus membranaceus	3,9-dimethoxypterocarpan-10-O-β-D-glucopyranoside
56	Astragalus membranaceus	3-O-β-D-galactopyranosyl-β-sitosterol
57	Astragalus membranaceus	3-O-β-D-xylopyranosyl-6-O-β-D-glucopyranosyl-cycloastragenol
58	Astragalus membranaceus	3-hydro-9,10-dimethoxypterocarpan
59	Astragalus membranaceus	3-hydro-9,10-dimethoxypterocarpan-pentosyl-hexoside
60	Astragalus membranaceus	3-hydro-9-methoxypterocarpan
61	Astragalus membranaceus	3-hydro-9-methoxypterocarpan hexoside
62	Astragalus membranaceus	3-hydro-9-methoxypterocarpan hexosyl-hexoside
63	Astragalus membranaceus	3-hydro-9-methoxypterocarpan malonyl-glucoside
64	Astragalus membranaceus	3-hydroxy-9,10-dimethoxypterocarpan
65	Astragalus membranaceus	3-hydroxyflavanone
66	Astragalus membranaceus	4'7-dihydroxyisoflavone
67	Astragalus membranaceus	3'-O-Methylorobol
68	Astragalus membranaceus	5,7-dihydroxy-4'-methoxy-isoflavone
69	Astragalus membranaceus	5-hydroxy-4'-methoxyisoflavone-7-O-β-D-glycoside-6"-O-malonate
70	Astragalus membranaceus	6"-O-acetyl-(3R)-7,2'-dihydroxy-3',4'-dimethoxyisoflavan-7-O-β-D-glucopyranoside

71	Astragalus membranaceus	6''-O-acetyl-(6aR,11aR)-3-hydroxy-9,10-dimethoxypterocarpan-3-O-β-D-glucopyranoside
72	Astragalus membranaceus	6''-O-acetyl-ononin
73	Astragalus membranaceus	6''-O-acetylcalycosin-7-O-β-D-glucopyranoside
74	Astragalus membranaceus	6''-O-acetylononin
75	Astragalus membranaceus	6''-acetylononin
76	Astragalus membranaceus	6,4'-dimethoxyisoflavone-7-O-glucoside
77	Astragalus membranaceus	6,4'-dimethoxyisoflavone-7-O-glycoside
78	Astragalus membranaceus	6,4'-dimethoxyisoflavone-7-O-β-D-glycoside
79	Astragalus membranaceus	7,2''-dihydroxy-3'',4''-dimethoxyisoflavane
80	Astragalus membranaceus	7,2',3'-trihydroxy-4'-methoxyisoflavan
81	Astragalus membranaceus	7,2'-dihydroxy-3',4',5'-trimethoxyisoflavan-7-O-β-D-glucuronide
82	Astragalus membranaceus	7,2'-dihydroxy-3',4',6'-trimethoxyisoflavan
83	Astragalus membranaceus	7,2'-dihydroxy-3',4'-dimethoxyisoflavan
84	Astragalus membranaceus	7,2'-dihydroxy-3',4'-dimethoxyisoflavan-2'-O-β-D-glucuronide
85	Astragalus membranaceus	7,2'-dihydroxy-3',4'-dimethoxyisoflavan-7-O-β-D-glucoside
86	Astragalus membranaceus	7,2'-dihydroxy-3',4'-dimethoxyisoflavan-7-O-β-D-glucoside-6''-O-malonate
87	Astragalus membranaceus	7,2'-dihydroxy-3',4'-dimethoxyisoflavan-7-O-β-D-glucuronide
88	Astragalus membranaceus	7,2'-dihydroxy-3',4'-dimethoxyisoflavane
89	Astragalus membranaceus	7,2'-dihydroxy-3',4'-dimethoxyisoflavone 7-O-glucoside
90	Astragalus membranaceus	7,3'-dihydroxy-2',4'-dimethoxyisoflavan-7-O-β-D-glucoside
91	Astragalus membranaceus	7,3'-dihydroxy-2',4'-dimethoxyisoflavan-7-O-β-D-glucosyl-3'-O-β-D-glucuronide
92	Astragalus membranaceus	7,3'-dihydroxy-4'-methoxyisoflavone
93	Astragalus membranaceus	7,3'-dihydroxy-6,4'-dimethoxyisoflavone
94	Astragalus membranaceus	7,8-dihydroxy-3',4'-dimethoxyisoflavan
95	Astragalus membranaceus	7-hydroxy-4'-methoxyisoflavone
96	Astragalus membranaceus	7-hydroxy-6,4'-dimethoxyisoflavon
97	Astragalus membranaceus	7-hydroxy-6,4'-dimethoxyisoflavone
98	Astragalus membranaceus	7-hydroxycoumarin
99	Astragalus membranaceus	8,3'-dihydroxy-7,4'-dimethoxy-isoflavone
100	Astragalus membranaceus	8-(dimethylaminomethyl)-7-hydroxy-2-methylisoflavone hydrochloride
101	Astragalus membranaceus	8-methoxyvestitol
102	Astragalus membranaceus	9,10-diMP-3-O-acetyl-glucoside
103	Astragalus membranaceus	9,10-dimethoxy-6a,11a-dihydro-6H-[1]benzofuro[3,2-c]chromen-3-ol
104	Astragalus membranaceus	9,10-dimethoxypterocarpan 3-O-β-D-glucoside
105	Astragalus membranaceus	9,10-dimethoxypterocarpan-3-O-acetyl-glucoside
106	Astragalus membranaceus	9,10-dimethoxypterocarpan-3-O-glucoside

107	Astragalus membranaceus	9,10-dimethoxypterocarpan-3-O-glycoside
108	Astragalus membranaceus	9,10-dimethoxypterocarpan-3-O-malonyl-glucoside
109	Astragalus membranaceus	9,10-dimethoxypterocarpan-3-O-β-D-glucopyranoside
110	Astragalus membranaceus	9,10-dimethoxypterocarpan-3-O-β-D-glucoside
111	Astragalus membranaceus	9,10-dimethoxypterocarpan-3-O-β-D-glycoside
112	Astragalus membranaceus	9-methoxy-nissolin-3-O-β-D-glucoside
113	Astragalus membranaceus	9-methoxynissolin
114	Astragalus membranaceus	9Z,12Z-octadecadienoic acid
115	Astragalus membranaceus	Isoastragaloside II
116	Astragalus membranaceus	Isomucronulatol
117	Astragalus membranaceus	L-phenylalanine
118	Astragalus membranaceus	L-tryptophan
119	Astragalus membranaceus	acetylastragaloside I
120	Astragalus membranaceus	aciphyllene
121	Astragalus membranaceus	adenosine
122	Astragalus membranaceus	afromosin
123	Astragalus membranaceus	afrormosin
124	Astragalus membranaceus	afrormosin-7-O-glycoside-6''-O-malonate
125	Astragalus membranaceus	afrormosin-7-O-β-D-glycoside
126	Astragalus membranaceus	afrormosin-7-O-β-D-glycoside-6''-O-malonate
127	Astragalus membranaceus	agroastragaloside I
128	Astragalus membranaceus	agroastragaloside III
129	Astragalus membranaceus	agroastragaloside IV
130	Astragalus membranaceus	alexandroside I
131	Astragalus membranaceus	ammopiptanoside A
132	Astragalus membranaceus	apigenin
133	Astragalus membranaceus	apigenin-hexoside
134	Astragalus membranaceus	artemisic acid
135	Astragalus membranaceus	asparagine
136	Astragalus membranaceus	astraciceran
137	Astragalus membranaceus	astragalin
138	Astragalus membranaceus	astragaloside
139	Astragalus membranaceus	astragaloside A
140	Astragalus membranaceus	astragaloside I
141	Astragalus membranaceus	astragaloside I isomer
142	Astragalus membranaceus	astragaloside II

143	Astragalus membranaceus	astragaloside II isomer
144	Astragalus membranaceus	astragaloside III
145	Astragalus membranaceus	astragaloside IV
146	Astragalus membranaceus	astragaloside IV isomer
147	Astragalus membranaceus	astragaloside V
148	Astragalus membranaceus	astragaloside VI
149	Astragalus membranaceus	astragaloside VII
150	Astragalus membranaceus	astragaloside VIII
151	Astragalus membranaceus	astragaloside IV
152	Astragalus membranaceus	astragaluquinone
153	Astragalus membranaceus	astragenol
154	Astragalus membranaceus	astragloside IV
155	Astragalus membranaceus	astraisoflavan-7-O-β-D-glucoside
156	Astragalus membranaceus	astraisoflavan-7-O-β-D-glycoside-6''-O-malonate
157	Astragalus membranaceus	astraisoflavanglucoside-6'-O-malonate
158	Astragalus membranaceus	astraisoflavanglycoside
159	Astragalus membranaceus	astraisoflavanglycoside-6''-O-malonate
160	Astragalus membranaceus	astraisoflavanglycoside-6''-O-malonate isomer
161	Astragalus membranaceus	astramembranin II
162	Astragalus membranaceus	astramembrannin II
163	Astragalus membranaceus	astraperocarpan-3-O-glycoside
164	Astragalus membranaceus	astraperocarpan-3-O-glycoside-6'-O-malonate
165	Astragalus membranaceus	astraperocarpan-3-O-glycoside-6'-O-malonate isomer
166	Astragalus membranaceus	astrapterocarpan
167	Astragalus membranaceus	astrapterocarpan-3-O-β-D-glycoside-6''-O-malonate
168	Astragalus membranaceus	astrapterocarpanglucoside-6''-O-malonate
169	Astragalus membranaceus	astrapterocarpanglucoside-6'-O-malonate
170	Astragalus membranaceus	azelaic acid
171	Astragalus membranaceus	baicalein
172	Astragalus membranaceus	biochanin A
173	Astragalus membranaceus	caffeic acid
174	Astragalus membranaceus	calycosin
175	Astragalus membranaceus	calycosin 7-O-glucoside
176	Astragalus membranaceus	calycosin 7-O-β-D-(6''-acetyl)-glucoside
177	Astragalus membranaceus	calycosin 7-O-β-D-{6''-[(E)-but-2-enoyl]}-glucoside
178	Astragalus membranaceus	calycosin sulfate

179	Astragalus membranaceus	calycosin-7-O-glucoside
180	Astragalus membranaceus	calycosin-7-O-glycoside
181	Astragalus membranaceus	calycosin-7-O-glycoside-6''-O-malonate
182	Astragalus membranaceus	calycosin-7-O-glycoside-6''-O-malonate isomer
183	Astragalus membranaceus	calycosin-7-O-β-D-6''-O-acetyl-glucopyranoside
184	Astragalus membranaceus	calycosin-7-O-β-D-glucopyranoside
185	Astragalus membranaceus	calycosin-7-O-β-D-glucopyranoside-6''-acetate
186	Astragalus membranaceus	calycosin-7-O-β-D-glucoside
187	Astragalus membranaceus	calycosin-7-O-β-D-glucoside-6''-O-acetate
188	Astragalus membranaceus	calycosin-7-O-β-D-glucoside-6''-O-malonate
189	Astragalus membranaceus	calycosin-7-O-β-D-glucoside-6''-O-malonate isomer
190	Astragalus membranaceus	calycosin-7-O-β-D-glucuronide
191	Astragalus membranaceus	calycosin-7-O-β-D-glycoside
192	Astragalus membranaceus	calycosin-7-O-β-D-glycoside-6''-O-acetate
193	Astragalus membranaceus	calycosin-7-O-β-D-glycoside-6''-O-malonate
194	Astragalus membranaceus	calycosin-7-glucoside
195	Astragalus membranaceus	calycosine-O-β-D-glucoside
196	Astragalus membranaceus	campanulin
197	Astragalus membranaceus	carotene
198	Astragalus membranaceus	cellulase
199	Astragalus membranaceus	chlorogenic acid
200	Astragalus membranaceus	complanaruside
201	Astragalus membranaceus	complanatuside
202	Astragalus membranaceus	coumaric acid
203	Astragalus membranaceus	cycloastragenol
204	Astragalus membranaceus	cycloastragenol-3-O-β-D-(3'',4''-di-O-acetyl) xylopyranosyl-6-O-β-D-glucopyranoside
205	Astragalus membranaceus	cyclocanthoside E
206	Astragalus membranaceus	cyclocephaloside II
207	Astragalus membranaceus	cyclogaleginoside A
208	Astragalus membranaceus	cycloorbigenin 3-O-β-d-xylopyranoside
209	Astragalus membranaceus	daidzein
210	Astragalus membranaceus	daidzein-7-O-β-D-glucuronide
211	Astragalus membranaceus	daucosterol
212	Astragalus membranaceus	dihydroxy-dimethoxy dihydroisoflavone-hexoside
213	Astragalus membranaceus	dihydroxy-dimethoxyisoflavan
214	Astragalus membranaceus	dihydroxy-dimethoxyisoflavone

215	Astragalus membranaceus	dihydroxy-octadecadienoic acid
216	Astragalus membranaceus	dihydroxy-trimethoxy dihydroisoflavone
217	Astragalus membranaceus	dihydroxy-trimethoxy dihydroisoflavone-hexoside
218	Astragalus membranaceus	dihydroxy-trimethoxy dihydroisoflavone-pentose
219	Astragalus membranaceus	dihydroxyflavone
220	Astragalus membranaceus	diosmetin-7-O-β-D-glucopyranoside
221	Astragalus membranaceus	eremophiloside J
222	Astragalus membranaceus	ferulic acid
223	Astragalus membranaceus	formononetin
224	Astragalus membranaceus	formononetin 7-O-glucoside
225	Astragalus membranaceus	formononetin 7-O-glucoside-6''-O-acetate
226	Astragalus membranaceus	formononetin 7-O-glucoside-6''-O-malonate
227	Astragalus membranaceus	formononetin 7-O-β-D-glucopyranoside
228	Astragalus membranaceus	formononetin 7-O-β-D-glucopyranoside-6''-acetate
229	Astragalus membranaceus	formononetin 7-O-β-D-glucoside
230	Astragalus membranaceus	formononetin 7-O-β-D-glucoside-6''-O-malonate
231	Astragalus membranaceus	formononetin 7-O-β-D-glucuronide
232	Astragalus membranaceus	formononetin 7-O-β-D-glycoside
233	Astragalus membranaceus	formononetin 7-O-β-D-glycoside-6''-O-malonate
234	Astragalus membranaceus	formononetin-7-O-glycoside
235	Astragalus membranaceus	formononetin-7-O-glycoside-6''-O-malonate
236	Astragalus membranaceus	formononetin-7-O-β-D-glucoside
237	Astragalus membranaceus	formononetin-7-O-β-D-glucoside-6''-O-malonate
238	Astragalus membranaceus	formononetin-7-O-β-D-glycoside
239	Astragalus membranaceus	formononetin-7-O-β-D-glycoside-6''-O-acetate
240	Astragalus membranaceus	formononetin-7-O-β-D-glycoside-6''-O-malonate
241	Astragalus membranaceus	galactomannan
242	Astragalus membranaceus	genistein
243	Astragalus membranaceus	hederagenin
244	Astragalus membranaceus	hesperetin
245	Astragalus membranaceus	hesperidin
246	Astragalus membranaceus	hexadecanoic acid
247	Astragalus membranaceus	hispidulin
248	Astragalus membranaceus	hydroxy-octadecatrienoic acid
249	Astragalus membranaceus	hyperoside
250	Astragalus membranaceus	isoamylase

251	Astragalus membranaceus	isoastragaloside I
252	Astragalus membranaceus	isoastragaloside II
253	Astragalus membranaceus	isoastragaloside IV
254	Astragalus membranaceus	isoliquiritigenin
255	Astragalus membranaceus	isomucronulatol
256	Astragalus membranaceus	isomucronulatol 7-O-glucoside
257	Astragalus membranaceus	isomucronulatol-7-O-glycoside-6''-O-acetyl
258	Astragalus membranaceus	isomucronulatol-7-O-glycoside-glycoside
259	Astragalus membranaceus	isomucronulatol-7-O-β-D-glucopyranoside
260	Astragalus membranaceus	isomucronulatol-7-O-β-D-glucoside
261	Astragalus membranaceus	isomucronulatol-acetyl-glucoside
262	Astragalus membranaceus	isomucronulatol-hexosyl-hexoside
263	Astragalus membranaceus	isoquercitrin
264	Astragalus membranaceus	isorhamnetin
265	Astragalus membranaceus	isorhamnetin 3-O-rutinoside
266	Astragalus membranaceus	isorhamnetin-3-O-neohesperidoside
267	Astragalus membranaceus	isorhamnetin-3-O-β-D-glucoside
268	Astragalus membranaceus	isorhamnetin-3-β-D-glucoside
269	Astragalus membranaceus	isorhamnetin-O-hexoside
270	Astragalus membranaceus	isorhamnetin-O-hexoside-hexoside derivative
271	Astragalus membranaceus	kaempferol
272	Astragalus membranaceus	kaempferol 4'-methylether-3-β-D-glucoside
273	Astragalus membranaceus	kumatakenin
274	Astragalus membranaceus	leucoside
275	Astragalus membranaceus	licoagroside D
276	Astragalus membranaceus	linolenic acid
277	Astragalus membranaceus	liquiritigenin
278	Astragalus membranaceus	lupeol
279	Astragalus membranaceus	malonyl astragaloside I
280	Astragalus membranaceus	malonyl astragaloside II isomer
281	Astragalus membranaceus	malonyl astragaloside IV isomer
282	Astragalus membranaceus	malonylastragaloside II isomer
283	Astragalus membranaceus	mangiferin
284	Astragalus membranaceus	medicarpin
285	Astragalus membranaceus	methoxypterocarpan
286	Astragalus membranaceus	methylnissolin 3-glucoside

287	Astragalus membranaceus	methylnissolin 7-O-glucoside
288	Astragalus membranaceus	methylnissolin-3-O-glucoside
289	Astragalus membranaceus	mosloflavone
290	Astragalus membranaceus	naringenin chalcone
291	Astragalus membranaceus	naringin
292	Astragalus membranaceus	octadeca-9,12-dienoic acid
293	Astragalus membranaceus	odoratin
294	Astragalus membranaceus	odoratin-7-O-glucoside
295	Astragalus membranaceus	odoratin-7-O-glucoside-6''-O-malonate
296	Astragalus membranaceus	odoratin-7-O-glycoside
297	Astragalus membranaceus	odoratin-7-O-glycoside-6''-O-malonate
298	Astragalus membranaceus	odoratin-7-O-β-D-glucopyranoside
299	Astragalus membranaceus	odoratin-7-O-β-D-glycoside
300	Astragalus membranaceus	odoration
301	Astragalus membranaceus	ononin
302	Astragalus membranaceus	patchouli alcohol
303	Astragalus membranaceus	pendulone
304	Astragalus membranaceus	pinitol
305	Astragalus membranaceus	pogostone
306	Astragalus membranaceus	pratensein
307	Astragalus membranaceus	pratensein-7-O-glucoside
308	Astragalus membranaceus	pratensein-7-O-glucoside-6''-O-malonate
309	Astragalus membranaceus	pratensein-7-O-β-D-glucopyranoside
310	Astragalus membranaceus	pratensein-7-O-β-D-glucoside
311	Astragalus membranaceus	pratensein-7-O-β-D-glycoside
312	Astragalus membranaceus	protocatechuic acid
313	Astragalus membranaceus	quercetin
314	Astragalus membranaceus	quercetin-O-hexoside-pentoside
315	Astragalus membranaceus	quercetin-O-malonyl-hexoside
316	Astragalus membranaceus	quercetin-O-rutinoside-hexoside
317	Astragalus membranaceus	rhamnocitrin
318	Astragalus membranaceus	rhamnocitrin-O-hexoside
319	Astragalus membranaceus	rhamnocitrin-O-malonyl-glucoside-rhamnoside
320	Astragalus membranaceus	rhamnocitrin-O-malonyl-hexoside
321	Astragalus membranaceus	rhamnocitrin-hexoside
322	Astragalus membranaceus	rhamnocitrin-hexosyl-acetate

323	Astragalus membranaceus	rhamnocitrin-hexosyl-malonate
324	Astragalus membranaceus	rutin
325	Astragalus membranaceus	saccharose
326	Astragalus membranaceus	seychellene
327	Astragalus membranaceus	sissotrin
328	Astragalus membranaceus	smyrnovinine
329	Astragalus membranaceus	sophoraflavoside II
330	Astragalus membranaceus	sophoricoside
331	Astragalus membranaceus	soyasaponin I
332	Astragalus membranaceus	soyasaponin II
333	Astragalus membranaceus	stigmast-4-en-6β-ol-3-one
334	Astragalus membranaceus	sucrose
335	Astragalus membranaceus	trans-caryophyllene
336	Astragalus membranaceus	trigonoside I
337	Astragalus membranaceus	trihydroxy-dimethoxy dihydroisoflavone-hexoside
338	Astragalus membranaceus	trihydroxy-dimethoxyisoflavan-hexoside
339	Astragalus membranaceus	trihydroxy-methoxyisoflavan-hexosyl-hexoside
340	Astragalus membranaceus	trihydroxy-octadecadienoic acid
341	Astragalus membranaceus	triterpene saponin
342	Astragalus membranaceus	trojanoside I
343	Astragalus membranaceus	vanillic acid
344	Astragalus membranaceus	vesticarpan
345	Astragalus membranaceus	wogonin
346	Astragalus membranaceus	α-amylase
347	Astragalus membranaceus	α-guaiene
348	Astragalus membranaceus	α-patchoulene
349	Astragalus membranaceus	β-D-glucopyranoside, (3β,6α,16β,20R,24S)-3-[(3,4-di-O-acetyl-β-D-xylopyranosyl) oxy]-20,24-epoxy-16,25-dihydroxy-9,19-cyclolanostan-6-yl
350	Astragalus membranaceus	β-elemene
351	Astragalus membranaceus	β-patchoulene
352	Astragalus membranaceus	β-sitosterol
353	Astragalus membranaceus	δ-guaiene
354	Angelica gigas	(+)-cuparene
355	Angelica gigas	(+)-spathulenol
356	Angelica gigas	(+)-β-funebrene
357	Angelica gigas	(+/-)-3'-hydroxy-3',4'-dihydroxanthyletin
358	Angelica gigas	(-)-isoledene

359	Angelica gigas	(-)-spathulenol
360	Angelica gigas	(-)-trans-pinocarvyl acetate
361	Angelica gigas	(3'Z)-(3R,8S,3a'R,6'S)-3,3a':8,6'-biligustilide
362	Angelica gigas	(3E,5Z)-1,3,5-undecatriene
363	Angelica gigas	(3Z,3Z')-6.8',7.3'-diligustilide
364	Angelica gigas	(E)-2-nonen-4-yne
365	Angelica gigas	(E)-2-nonenal
366	Angelica gigas	(E)-2-octenal
367	Angelica gigas	(E)-3-butylidene phthalide
368	Angelica gigas	(E)-6,7-cis-dihydroxy-ligustilide
369	Angelica gigas	(E)-carveol
370	Angelica gigas	(E)-ferulic acid
371	Angelica gigas	(E)-ligustilide
372	Angelica gigas	(E)-solanone
373	Angelica gigas	(E)-β-caryophyllene
374	Angelica gigas	(E)-β-ocimene
375	Angelica gigas	(E)-ρ-2-menthen-1-ol
376	Angelica gigas	(E)-ρ-mentha-2,8-dien-1ol
377	Angelica gigas	(E,E)-2,4-decadienal
378	Angelica gigas	(E,E)-α-farnesene
379	Angelica gigas	(E,Z)-2,4-decadienal
380	Angelica gigas	(R)-1-hexen-3-ol
381	Angelica gigas	(R,E)-1-phenylnon-2-en-1-ol
382	Angelica gigas	(Z)-2-nonen-4-yne
383	Angelica gigas	(Z)-3-butylidene phthalide
384	Angelica gigas	(Z)-3-butylidene-3,4-dihydro phthalide
385	Angelica gigas	(Z)-6,7-cis-dihydroxyligustilide
386	Angelica gigas	(Z)-6,7-epoxyligustilide
387	Angelica gigas	(Z)-butylidenephthalide
388	Angelica gigas	(Z)-carveol
389	Angelica gigas	(Z)-ligustilide
390	Angelica gigas	(Z)-piperitol
391	Angelica gigas	(Z)-β-farnesene
392	Angelica gigas	(Z)-β-ocimene
393	Angelica gigas	1,10-didenhydroaristolane
394	Angelica gigas	1,3,5-undecatriene

395	Angelica gigas	1,3,6-octatriene, 3,7-dimethyl-, (E)-
396	Angelica gigas	1,3,8-menthatriene
397	Angelica gigas	1,3-benzodioxole, 5-(2-propenyl)-
398	Angelica gigas	1,3-cyclohexadiene, 1,5,5,6-tetramethyl-
399	Angelica gigas	1,3-isobenzofurandione
400	Angelica gigas	1,4-cyclohexadiene-1,2-dicarboxylic anhydride
401	Angelica gigas	1,4-β-D-galactanase
402	Angelica gigas	1,7-diepi-β-cedrene
403	Angelica gigas	1,9-dioxa-4,6-diazacycloundecane-5-thione
404	Angelica gigas	1-(3H)-isobenzofuranone, 3-butylidene-
405	Angelica gigas	1-(but-3-enyl) indan-1-ol
406	Angelica gigas	1-acetyl-β-carboline
407	Angelica gigas	1-butanone, 1-phenyl-
408	Angelica gigas	1-heptadecanol
409	Angelica gigas	1-hexadecanol
410	Angelica gigas	1-octadecanol
411	Angelica gigas	1-pentadecanol
412	Angelica gigas	1-pentanone, 1-phenyl-
413	Angelica gigas	1-phenyl-1-pentanone
414	Angelica gigas	1-phenyl-1-propanol-(1)
415	Angelica gigas	1-tetradecene
416	Angelica gigas	10-angeloylbutylphthalide
417	Angelica gigas	11-angeloylsenkyunolide F
418	Angelica gigas	11-hexadecen-1-ol, (Z)-
419	Angelica gigas	19-methylene-5,10-secocholestan-3,5-dione
420	Angelica gigas	1H-pyrrole-2-carboxaldehyde
421	Angelica gigas	2'-hydroxyvalerophenone
422	Angelica gigas	2(3H)-furanone, 5-hexyldihydro-
423	Angelica gigas	2(3H)-furanone, dihydro-5-pentyl-
424	Angelica gigas	2,3,6-trimethylbenzaldehyde
425	Angelica gigas	2,3-dehydro-1,8-cineole
426	Angelica gigas	2,4,6-trimethyl heptane
427	Angelica gigas	2,4,6-trimethyl-1,3,6-heptatriene
428	Angelica gigas	2,4-decadienal
429	Angelica gigas	2-(1-oxypentyl)-benzoic acid methyl ester
430	Angelica gigas	2-acetylpyrrole

431	Angelica gigas	2-carene
432	Angelica gigas	2-cyclopenten-1-one
433	Angelica gigas	2-decenal, (Z)-
434	Angelica gigas	2-furancarboxaldehyde
435	Angelica gigas	2-furanmethanol
436	Angelica gigas	2-furanmethanol, acetate
437	Angelica gigas	2-heptanone
438	Angelica gigas	2-hexenal
439	Angelica gigas	2-hydroxycyclopentadecanone
440	Angelica gigas	2-isopropenyl-2,3-dihydrofuro [3,2-g]chromen-7-one
441	Angelica gigas	2-isopropylpsoralen
442	Angelica gigas	2-methoxy-4-vinylphenol
443	Angelica gigas	2-methoxyphenol
444	Angelica gigas	2-methyl butanoic acid
445	Angelica gigas	2-methyl decane
446	Angelica gigas	2-methyl nonane
447	Angelica gigas	2-methyl octane
448	Angelica gigas	2-methyl-2-butenal
449	Angelica gigas	2-methyl-2-cyclopentenone
450	Angelica gigas	2-methyl-3-buten-2-ol
451	Angelica gigas	2-methyl-propanol
452	Angelica gigas	2-methylbutanal
453	Angelica gigas	2-methylbutyl hexanoate
454	Angelica gigas	2-methylpropyl isobutyrate
455	Angelica gigas	2-nonanone
456	Angelica gigas	2-pentadecanol
457	Angelica gigas	2-pentyl furan
458	Angelica gigas	2-santalene
459	Angelica gigas	2-undecanone
460	Angelica gigas	3'(R)-O-β-D-glucopyranosyl-3',4'-dihydroxanthyletin
461	Angelica gigas	3'(S)-O-β-D-glucopyranosyl-3',4'-dihydroxanthyletin
462	Angelica gigas	3,3'Z-6.7',7.6'-diligustilide
463	Angelica gigas	3,5-dimethoxytoluene
464	Angelica gigas	3,5-dimethyl styrene
465	Angelica gigas	3,5-dimethylbenzaldehyde
466	Angelica gigas	3,7-dimethyl-1,3,6-octatriene

467	Angelica gigas	3,9-dihydroxyligustilide
468	Angelica gigas	3-N-butylphthalide
469	Angelica gigas	3-butylidene-4,5-dihydro-2(1,3H)-1-isobenzofuranol
470	Angelica gigas	3-butylidene-7-hydroxyphthalide
471	Angelica gigas	3-isopropylbenzaldehyde
472	Angelica gigas	3-menthene
473	Angelica gigas	3-methyl butanal
474	Angelica gigas	3-methyl nonane
475	Angelica gigas	3-methyl-2-butenoic aicd, 3-tridecyl ester
476	Angelica gigas	3-methyl-2-butenol
477	Angelica gigas	3-methylbutanal
478	Angelica gigas	3-n-butyl phthalide
479	Angelica gigas	3-n-butylphthalide
480	Angelica gigas	3a.7'a,7a.3'a-diligustilide
481	Angelica gigas	4-aminobutyric acid
482	Angelica gigas	4-decene
483	Angelica gigas	4-ethenyl-2-methoxyphenol
484	Angelica gigas	4-hydroxy-3-butylphthalide
485	Angelica gigas	4-hydroxy-4-methyl-2-pentanone
486	Angelica gigas	4-methyl pentanol
487	Angelica gigas	4-methyl-1-penten-3-one
488	Angelica gigas	4-octanone
489	Angelica gigas	4-pyridinol
490	Angelica gigas	4-tetradecanol
491	Angelica gigas	4-vinyl guaiacol
492	Angelica gigas	5-methylfurfural
493	Angelica gigas	5-tetradecene, (E)-
494	Angelica gigas	5-undecen-3-yne
495	Angelica gigas	5-undecene
496	Angelica gigas	5-undecene, (E)-
497	Angelica gigas	6-butyl-1,4-cycloheptadiene
498	Angelica gigas	6-dodecen-1-al
499	Angelica gigas	6-methoxycoumarin
500	Angelica gigas	6-methyl-5-hepten-2-one
501	Angelica gigas	6-undecanol
502	Angelica gigas	6-undecanone

503	Angelica gigas	7-demethylsuberosine
504	Angelica gigas	7-hexadecene, (Z)-
505	Angelica gigas	7-hydroxy-6-(2R-hydroxy-3-methylbut-3-enyl)coumarin
506	Angelica gigas	8-heptadecene
507	Angelica gigas	9,12,15-octadecatrienoic acid, (Z,Z,Z)-
508	Angelica gigas	9,12-octadecadienoic acid
509	Angelica gigas	9,12-octadecadienoic acid (Z,Z)-
510	Angelica gigas	9,12-octadecadienoic acid, methyl ester
511	Angelica gigas	9-octadecanoic acid
512	Angelica gigas	9-octadecenamide, (Z)-
513	Angelica gigas	9H-fluorene
514	Angelica gigas	D-carvone
515	Angelica gigas	D-limonene
516	Angelica gigas	D-nerolidol
517	Angelica gigas	E,E'-3.3',8.8'-diligustilide
518	Angelica gigas	E,E'-3.3',8.8'-isodiligustilide
519	Angelica gigas	E-2-hexadecacen-1-ol
520	Angelica gigas	E-6,7-dihydroxydihydroligustilide
521	Angelica gigas	E-butylidenephthalide
522	Angelica gigas	E-coniferin
523	Angelica gigas	E-ligustilide
524	Angelica gigas	E-ligustulide
525	Angelica gigas	O-phthalic acid
526	Angelica gigas	Z,Z'-3,3',8,8'-diligustilide
527	Angelica gigas	Z,Z'-3.3',8.8'-diligustilide
528	Angelica gigas	Z,Z'-3.3'8.8'-diligustilide
529	Angelica gigas	Z,Z'-6,6',7,3'a-diligustilide
530	Angelica gigas	Z,Z'-6,6',7,3'a-diligustilide 8-epimer
531	Angelica gigas	Z,Z'-6,8',7,3'-diligustilide
532	Angelica gigas	Z,Z'-6.8',7.3'-diligustilide
533	Angelica gigas	Z,Z'-6.8'7.3'-diligustilide
534	Angelica gigas	Z,Z-3,3'-8'8'-diligudtilide
535	Angelica gigas	Z-3',8',3'a,7'a-tetrahydro-6,3',7,7'a-diligustilide-8'-one
536	Angelica gigas	Z-6,7-epoxyligustilide
537	Angelica gigas	Z-butylidenephthalide
538	Angelica gigas	Z-ligustilide

539	Angelica gigas	Z-ligustilide dimer E-232
540	Angelica gigas	Z/E-butylidenephthalide
541	Angelica gigas	[6-hydroxymethyl-2,3-dimethylphenyl] methanol
542	Angelica gigas	acetic acid
543	Angelica gigas	acetic acid, butyl ester
544	Angelica gigas	acetoacetic acid ester 3[10]-caren-4-ol
545	Angelica gigas	acetol acetate
546	Angelica gigas	acetoxyacetone
547	Angelica gigas	acoradiene
548	Angelica gigas	agarospirol
549	Angelica gigas	alanine
550	Angelica gigas	alloocimene
551	Angelica gigas	angelate
552	Angelica gigas	angelicide
553	Angelica gigas	angelicin
554	Angelica gigas	angeliferulate
555	Angelica gigas	anisic acid
556	Angelica gigas	ansapirolide
557	Angelica gigas	ansaspirolide
558	Angelica gigas	aromadendrene
559	Angelica gigas	aromadendrene, dehydro-
560	Angelica gigas	asparagine
561	Angelica gigas	asparaginic acid
562	Angelica gigas	azelaic acid
563	Angelica gigas	baicalin
564	Angelica gigas	benzaldehyde, 2,4,5-trimethyl-
565	Angelica gigas	benzaldehyde, 2,4,6-trimethyl-
566	Angelica gigas	benzaldehyde, 2,5-dimethyl-
567	Angelica gigas	benzene, 1,2,3-trimethyl-
568	Angelica gigas	benzene, 1,2-dimethoxy-4-(2-propenyl)-
569	Angelica gigas	benzene, pentyl-
570	Angelica gigas	benzeneacetaldehyde
571	Angelica gigas	benzeneacetic acid, α-oxo-, methyl ester
572	Angelica gigas	benzenemethanol, α, α, 4-trimethyl-
573	Angelica gigas	benzoic acid, 2-propenyl ester
574	Angelica gigas	bergamiol

575	Angelica gigas	bergapten
576	Angelica gigas	bicyclogermacrene
577	Angelica gigas	bisabolene
578	Angelica gigas	bomeol
579	Angelica gigas	bornyl acetate
580	Angelica gigas	bulnesol
581	Angelica gigas	butanal
582	Angelica gigas	butanoic acid
583	Angelica gigas	butanol
584	Angelica gigas	butyl heptanoate
585	Angelica gigas	butyl hexanoate
586	Angelica gigas	butyl octanoate
587	Angelica gigas	butyl phthalide
588	Angelica gigas	butylidene dihydrophthalide
589	Angelica gigas	butylidenephalide
590	Angelica gigas	butylidenephthalide
591	Angelica gigas	butylphthalide
592	Angelica gigas	butyrolactone
593	Angelica gigas	buylidene dihydro-phthalide
594	Angelica gigas	caffeic acid
595	Angelica gigas	camphene
596	Angelica gigas	camphenone, 6-
597	Angelica gigas	caryophyllene
598	Angelica gigas	cedrene
599	Angelica gigas	cedrol
600	Angelica gigas	chlorogenic acid
601	Angelica gigas	cis,cis-linoleic acid
602	Angelica gigas	cis-4-hydroxy-3-methylundecanoic acid lactone
603	Angelica gigas	cis-7-tetradecen-1-ol
604	Angelica gigas	cis-alloocimene
605	Angelica gigas	cis-carveol
606	Angelica gigas	cis-ocimene
607	Angelica gigas	cis-β-ocimene
608	Angelica gigas	citric acid
609	Angelica gigas	columbianetin
610	Angelica gigas	columbianetin O-β-D-glucopyranoside

611	Angelica gigas	coniferyl ferulate
612	Angelica gigas	copaene
613	Angelica gigas	cuminol
614	Angelica gigas	cuparene
615	Angelica gigas	cyclopentadecanone
616	Angelica gigas	cyclosativene
617	Angelica gigas	cystine
618	Angelica gigas	decahydro-1,1,7-trimethyl-4-methylene-[1a.a] 1H-cycloprop[e] azulene
619	Angelica gigas	decanal
620	Angelica gigas	decane
621	Angelica gigas	decursin
622	Angelica gigas	decursinol
623	Angelica gigas	decursinol angelate
624	Angelica gigas	dehydroaromadendrene
625	Angelica gigas	demethylsuberosin
626	Angelica gigas	dibenzofuran
627	Angelica gigas	dibutyl phthalate
628	Angelica gigas	diels-alder dimer of ligustilide
629	Angelica gigas	diisobutyl phthalate
630	Angelica gigas	dodecanal
631	Angelica gigas	dodecane
632	Angelica gigas	dodecanol
633	Angelica gigas	elemol
634	Angelica gigas	elixene
635	Angelica gigas	esculetin
636	Angelica gigas	ethanol
637	Angelica gigas	ethanone, 1-(3,4-dimethylphenyl)-
638	Angelica gigas	ethyl acetate
639	Angelica gigas	ethyl dl-mandelate
640	Angelica gigas	eudesmol
641	Angelica gigas	falcarindiol
642	Angelica gigas	falcarinol
643	Angelica gigas	ferulic acid
644	Angelica gigas	folic acid
645	Angelica gigas	folinic acid
646	Angelica gigas	fructose

647	Angelica gigas	fumaric acid
648	Angelica gigas	furan, 2-methyl-
649	Angelica gigas	furan, 2-pentyl-
650	Angelica gigas	furfural
651	Angelica gigas	galacturonic acid
652	Angelica gigas	gelispirolide
653	Angelica gigas	germacrene
654	Angelica gigas	globulol
655	Angelica gigas	glucose
656	Angelica gigas	glycine
657	Angelica gigas	guaiacol
658	Angelica gigas	guaiacylglycerol
659	Angelica gigas	guaiol
660	Angelica gigas	guaiyl acetate
661	Angelica gigas	heptadecane, 2,6,10,14-tetramethyl-
662	Angelica gigas	heptanal
663	Angelica gigas	heptane
664	Angelica gigas	hexadecane
665	Angelica gigas	hexadecanoic acid
666	Angelica gigas	hexadecanoic acid, methyl ester
667	Angelica gigas	hexadecanol
668	Angelica gigas	hexahydrofarnesyl acetone
669	Angelica gigas	hexanal
670	Angelica gigas	hexyl acetate
671	Angelica gigas	histidine
672	Angelica gigas	homosenkyunolide H
673	Angelica gigas	homosenkyunolide I
674	Angelica gigas	hypoxanthine-9-β-D-ribofuranoside
675	Angelica gigas	imperatorin
676	Angelica gigas	indole
677	Angelica gigas	inositol
678	Angelica gigas	isobutanal
679	Angelica gigas	isoeugenol
680	Angelica gigas	isoimperatorin
681	Angelica gigas	isoleucine
682	Angelica gigas	isophorone

683	Angelica gigas	isopimpinelline
684	Angelica gigas	isopropyl hexanoate
685	Angelica gigas	isopulegone
686	Angelica gigas	isospathulenol
687	Angelica gigas	isotokinolide B
688	Angelica gigas	ledene
689	Angelica gigas	ledene oxide-(II)
690	Angelica gigas	levistilide
691	Angelica gigas	levistilide A
692	Angelica gigas	levistolide A
693	Angelica gigas	ligustilide
694	Angelica gigas	limonene
695	Angelica gigas	limonene oxide
696	Angelica gigas	linalool
697	Angelica gigas	linoleic acid
698	Angelica gigas	linoleic acid, methyl ester
699	Angelica gigas	linolenic acid
700	Angelica gigas	lomatin
701	Angelica gigas	m-ethylphenol
702	Angelica gigas	magnolol
703	Angelica gigas	malic acid
704	Angelica gigas	maltol
705	Angelica gigas	marmesin
706	Angelica gigas	menthene-3-ol
707	Angelica gigas	methionine
708	Angelica gigas	methoxsalen
709	Angelica gigas	methyl hexadecanoate
710	Angelica gigas	methyl hexanoate
711	Angelica gigas	methyl linoleate
712	Angelica gigas	methyl palmitate
713	Angelica gigas	methylphthalimide
714	Angelica gigas	mono(2-ethylhexyl) phthalate
715	Angelica gigas	muskolactone
716	Angelica gigas	muurola-4,11-diene
717	Angelica gigas	myrcene
718	Angelica gigas	myrtanol

719	Angelica gigas	myrtanyl acetate
720	Angelica gigas	myrtenal
721	Angelica gigas	myrtenol
722	Angelica gigas	n-butylidenephthalide
723	Angelica gigas	n-butylphthalide
724	Angelica gigas	n-decanoic acid
725	Angelica gigas	n-hexadecanoic acid
726	Angelica gigas	n-pentylbenzene
727	Angelica gigas	n-tridecan-1-ol
728	Angelica gigas	naphthalene
729	Angelica gigas	naphthalene, 1,3-dimethyl-
730	Angelica gigas	naphthalene, 2-methyl-
731	Angelica gigas	neo-allo-ocimene
732	Angelica gigas	neocnidilide
733	Angelica gigas	neoligustilide
734	Angelica gigas	nicotinic acid
735	Angelica gigas	nodakenetin
736	Angelica gigas	nodakenin
737	Angelica gigas	nonacosane
738	Angelica gigas	nonane
739	Angelica gigas	o-cresol
740	Angelica gigas	o-cymene
741	Angelica gigas	ocimene
742	Angelica gigas	octadecane
743	Angelica gigas	octadecenoic acid
744	Angelica gigas	octahydro-3,8,8-trimethyl-6-methylene-1H,3a,7-methanoazulene
745	Angelica gigas	octanal
746	Angelica gigas	octane
747	Angelica gigas	octanoic acid
748	Angelica gigas	oleic acid, methyl ester
749	Angelica gigas	ornithine
750	Angelica gigas	osthole
751	Angelica gigas	oxirane, tetradecyl-
752	Angelica gigas	p-cimene
753	Angelica gigas	p-cymene
754	Angelica gigas	p-hydroxybenzoic acid

755	Angelica gigas	p-hydroxyphenethyl trans-ferulate
756	Angelica gigas	p-hydroxyphenylethanol ferulate
757	Angelica gigas	p-menth-1-en-9-ol
758	Angelica gigas	p-xylene
759	Angelica gigas	palmitic acid
760	Angelica gigas	palmitic acid methyl ester
761	Angelica gigas	palustrol
762	Angelica gigas	pentadecanal
763	Angelica gigas	pentadecane
764	Angelica gigas	pentadecanoic acid
765	Angelica gigas	pentadecanoic acid, methyl ester
766	Angelica gigas	pentanol
767	Angelica gigas	pentyl benzene
768	Angelica gigas	pentyl isohexanoate
769	Angelica gigas	pentylbenzene
770	Angelica gigas	peucedanone
771	Angelica gigas	phenol, 2-methoxy-4-propyl-
772	Angelica gigas	phenol, 4-ethyl-2-methoxy-
773	Angelica gigas	phenol, 4-pentyl-
774	Angelica gigas	phenylalanine
775	Angelica gigas	phosphoric acid
776	Angelica gigas	phthalic acid
777	Angelica gigas	phthalide
778	Angelica gigas	phthalide dimer
779	Angelica gigas	pinene oxide
780	Angelica gigas	pinocarveol
781	Angelica gigas	pipecolic acid
782	Angelica gigas	proline
783	Angelica gigas	propanedioic acid
784	Angelica gigas	propanoic acid
785	Angelica gigas	propyl-2-methyl-2-butenoic acid ester
786	Angelica gigas	protocatechuic acid
787	Angelica gigas	psoralen
788	Angelica gigas	quinic acid
789	Angelica gigas	r-terpinene
790	Angelica gigas	riligustilide

791	Angelica gigas	rosefuran
792	Angelica gigas	rosifoliol
793	Angelica gigas	sabinene
794	Angelica gigas	safranal
795	Angelica gigas	sedannoic acid lactone
796	Angelica gigas	sedecanoic acid lactone
797	Angelica gigas	sendanenolide A
798	Angelica gigas	senkyunolide
799	Angelica gigas	senkyunolide A
800	Angelica gigas	senkyunolide D
801	Angelica gigas	senkyunolide E
802	Angelica gigas	senkyunolide F
803	Angelica gigas	senkyunolide H
804	Angelica gigas	senkyunolide H-7-acetate
805	Angelica gigas	senkyunolide I
806	Angelica gigas	senkyunolide O
807	Angelica gigas	senkyunolide P
808	Angelica gigas	serine
809	Angelica gigas	seselin
810	Angelica gigas	sinaspirolide
811	Angelica gigas	spathulenol
812	Angelica gigas	sphatulenol
813	Angelica gigas	spinasterol
814	Angelica gigas	squalene
815	Angelica gigas	stearic acid
816	Angelica gigas	stigmasterol
817	Angelica gigas	succinic acid
818	Angelica gigas	sulphur dioxide
819	Angelica gigas	terpinen-4-ol
820	Angelica gigas	terpinolene
821	Angelica gigas	tetradecane
822	Angelica gigas	tetradecanol
823	Angelica gigas	threonine
824	Angelica gigas	thujopsene
825	Angelica gigas	tokinolide B
826	Angelica gigas	trans-13-octadecenoic acid

827	Angelica gigas	trans-alloocimene
828	Angelica gigas	trans-caryophyllene
829	Angelica gigas	trans-sabinol
830	Angelica gigas	trans-α-bergamotol
831	Angelica gigas	trans-β-farnesene
832	Angelica gigas	tricyclene
833	Angelica gigas	tridecane
834	Angelica gigas	umbelliferone
835	Angelica gigas	undecane
836	Angelica gigas	undecanol-3
837	Angelica gigas	valine
838	Angelica gigas	vanillic acid
839	Angelica gigas	verbenol
840	Angelica gigas	verbenone
841	Angelica gigas	viridiflorol
842	Angelica gigas	widdrene
843	Angelica gigas	xanthotoxin
844	Angelica gigas	α-alaskene
845	Angelica gigas	α-campholene aldehyde
846	Angelica gigas	α-cedrene
847	Angelica gigas	α-copaene
848	Angelica gigas	α-cuprenene
849	Angelica gigas	α-elemene
850	Angelica gigas	α-eudesmol
851	Angelica gigas	α-farnesene
852	Angelica gigas	α-fenchene
853	Angelica gigas	α-limonene
854	Angelica gigas	α-muurolene
855	Angelica gigas	α-myrcene
856	Angelica gigas	α-phellandrene
857	Angelica gigas	α-phellandrene-8-ol
858	Angelica gigas	α-pinene
859	Angelica gigas	α-pinene, (-)-
860	Angelica gigas	α-terpinene
861	Angelica gigas	α-terpineol
862	Angelica gigas	α-terpinolene

863	Angelica gigas	α-thujene
864	Angelica gigas	β-barbatene
865	Angelica gigas	β-bisabolene
866	Angelica gigas	β-caryophyllene oxide
867	Angelica gigas	β-cedrene
868	Angelica gigas	β-chamigrene
869	Angelica gigas	β-cis-farnesene
870	Angelica gigas	β-citronellol
871	Angelica gigas	β-cubebene
872	Angelica gigas	β-cuprenene
873	Angelica gigas	β-elemene
874	Angelica gigas	β-eudesmol
875	Angelica gigas	β-farnesene
876	Angelica gigas	β-funebrene
877	Angelica gigas	β-himachalene
878	Angelica gigas	β-myrcene
879	Angelica gigas	β-ocimene
880	Angelica gigas	β-phellandren-8-ol
881	Angelica gigas	β-phellandrene
882	Angelica gigas	β-pinene
883	Angelica gigas	β-sesquiphellandrene
884	Angelica gigas	β-sitosterol
885	Angelica gigas	γ-aminobutyric acid
886	Angelica gigas	γ-eudesmol
887	Angelica gigas	γ-gurjunene
888	Angelica gigas	γ-muurolene
889	Angelica gigas	γ-sitosterol
890	Angelica gigas	γ-terpinene
891	Angelica gigas	δ-3-carene
892	Angelica gigas	δ-cadinene
893	Angelica gigas	δ-guaiene
894	Angelica gigas	δ-limonene
895	Angelica gigas	ρ-cymen-8-ol
896	Angelica gigas	ρ-cymene
897	Trichosanthes Kirilowii Maximowicz	23,24-dihydrocucurbitacin B
898	Trichosanthes Kirilowii	4'-hydroxyscutellarin

	Maximowicz	
899	Trichosanthes Kirilowii Maximowicz	5-ethoxymethyl-1-carboxyl propyl-1H-pyrrole-2-carbaldehyde
900	Trichosanthes Kirilowii Maximowicz	5-hydroxymethyl-2-furfural
901	Trichosanthes Kirilowii Maximowicz	adenosine
902	Trichosanthes Kirilowii Maximowicz	apigenin
903	Trichosanthes Kirilowii Maximowicz	apigenin-7-O-glucuronide
904	Trichosanthes Kirilowii Maximowicz	chrysoeriol
905	Trichosanthes Kirilowii Maximowicz	cucurbitacin B
906	Trichosanthes Kirilowii Maximowicz	cucurbitacin D
907	Trichosanthes Kirilowii Maximowicz	cucurbitacin E
908	Trichosanthes Kirilowii Maximowicz	isoquercitrin
909	Trichosanthes Kirilowii Maximowicz	karasurin
910	Trichosanthes Kirilowii Maximowicz	luteolin
911	Trichosanthes Kirilowii Maximowicz	luteoloside
912	Trichosanthes Kirilowii Maximowicz	quercetin
913	Trichosanthes Kirilowii Maximowicz	rutin
914	Trichosanthes Kirilowii Maximowicz	stigmast-7-en-3β-ol
915	Trichosanthes Kirilowii Maximowicz	tangeretin
916	Trichosanthes Kirilowii Maximowicz	trichomislin
917	Trichosanthes Kirilowii Maximowicz	trichosanthin
918	Trichosanthes Kirilowii Maximowicz	vanillic acid
919	Trichosanthes Kirilowii Maximowicz	α-spinasterol
920	Trichosanthes Kirilowii Maximowicz	β-D-glucopyranosyl-a-spinasterol

921	Trichosanthes Kirilowii Maximowicz	(-)-pinoresinol C
922	Trichosanthes Kirilowii Maximowicz	(-)Pinoresinol
923	Trichosanthes Kirilowii Maximowicz	(-)Secoisolariciresinol
924	Trichosanthes Kirilowii Maximowicz	Anthricin
925	Trichosanthes Kirilowii Maximowicz	arvenin I
926	Trichosanthes Kirilowii Maximowicz	β-sitosterol
927	Trichosanthes Kirilowii Maximowicz	Britanin
928	Trichosanthes Kirilowii Maximowicz	cerotic acid
929	Trichosanthes Kirilowii Maximowicz	chrysoeriol-7- O-β-D-glucopyranoside
930	Trichosanthes Kirilowii Maximowicz	delta 7-stigmastenol
931	Trichosanthes Kirilowii Maximowicz	delta 7-stigmastenone-3
932	Trichosanthes Kirilowii Maximowicz	delta 7-stigmatenol-3-beta-D-glucopyranoside
933	Trichosanthes Kirilowii Maximowicz	dibutyl phthalate
934	Trichosanthes Kirilowii Maximowicz	diosmetin
935	Trichosanthes Kirilowii Maximowicz	diosmetin-7-O-β-D-glucopyranoside
936	Trichosanthes Kirilowii Maximowicz	ehletianol C
937	Trichosanthes Kirilowii Maximowicz	fluranthene
938	Trichosanthes Kirilowii Maximowicz	isoquercitrin
939	Trichosanthes Kirilowii Maximowicz	L-(-)-alpha-monopalmitin
940	Trichosanthes Kirilowii Maximowicz	lauric acid
941	Trichosanthes Kirilowii Maximowicz	ligballinol
942	Trichosanthes Kirilowii Maximowicz	lignoceric acid
943	Trichosanthes Kirilowii Maximowicz	linoleic acid

944	Trichosanthes Kirilowii Maximowicz	linolenic acid
945	Trichosanthes Kirilowii Maximowicz	luteolin 7-O-β-D-glucopyranoside
946	Trichosanthes Kirilowii Maximowicz	melissic acid
947	Trichosanthes Kirilowii Maximowicz	methyl hexadecanoate
948	Trichosanthes Kirilowii Maximowicz	montanic acid
949	Trichosanthes Kirilowii Maximowicz	myristic acid
950	Trichosanthes Kirilowii Maximowicz	palmitic acid
951	Trichosanthes Kirilowii Maximowicz	phenanthrene
952	Trichosanthes Kirilowii Maximowicz	Praeruptorin A
953	Trichosanthes Kirilowii Maximowicz	Quercitrin
954	Trichosanthes Kirilowii Maximowicz	stigmasterol
955	Trichosanthes Kirilowii Maximowicz	TKP
956	Trichosanthes Kirilowii Maximowicz	Tomentosin
957	Trichosanthes Kirilowii Maximowicz	trichobenzolignan
958	Trichosanthes Kirilowii Maximowicz	trichosanthin
959	Trichosanthes Kirilowii Maximowicz	1-O-acetylbritannilactone
960	Trichosanthes Kirilowii Maximowicz	1,5-di-O-caffeoylquinic acid
961	Trichosanthes Kirilowii Maximowicz	10α-cucurbita-5,24-dien-3β-ol
962	Trichosanthes Kirilowii Maximowicz	3-methyl phenanthrene
963	Trichosanthes Kirilowii Maximowicz	3,29-dibenzoyl rarounitriol
964	Trichosanthes Kirilowii Maximowicz	4′,6-Dihydroxy-4-methoxyisoaurone
965	Trichosanthes Kirilowii Maximowicz	7-O-Methylluteolin

Table S2. Potential targets of SH003

No.	Gene name
1	ABCB1
2	ABCC1
3	ABCG2
4	ACHE
5	ADA17
6	ADAMTS3
7	ADAMTS4
8	ADH1B
9	ADIPOQ
10	ADORA1
11	ADORA2A
12	AGT
13	AHR
14	AK1C2
15	AK1C3
16	AKR1B1
17	AKR1B10
18	AKT1
19	ALBU
20	ALDR
21	ALOX12
22	ALOX15
23	ALOX5
24	AMPM1
25	AMY1
26	ANDR
27	ANGI
28	ANXA5
29	AOFB
30	APOA2
31	APP
32	ARG1
33	ATM
34	AURKA

35	AURKB
36	BACE1
37	BCAT2
38	BCL2L1
39	BLVRB
40	BMP2
41	BMP7
42	BRAF1
43	C1R
44	C3
45	CA12
46	CA2
47	CA4
48	CA7
49	CAH1
50	CAH12
51	CAH2
52	CASP3
53	CASP7
54	CASP8
55	CASP9
56	CAT
57	CATB
58	CATD
59	CATS
60	CBR1
61	CCK
62	CCL17
63	CCL2
64	CCNA2
65	CCNB1
66	CCNB2
67	CCNB3
68	CCND1
69	CD38
70	CD5R1

71	CDH1
72	CDK
73	CDK1
74	CDK2
75	CDK4
76	CDK5
77	CDK5R1
78	CDK6
79	CDK9
80	CFAB
81	CFAD
82	CFTR
83	CHK1
84	CHLE
85	CHUK
86	CSF2
87	CSNK2A1
88	CSNK2A2
89	CYP19A1
90	CYP1A1
91	CYP1A2
92	CYP1B1
93	CYP2B6
94	CYP2C8
95	CYP3A4
96	DAPK1
97	DCAM
98	DDIT4
99	DDX6
100	DGAT1
101	DHB11
102	DHI1
103	DHSO
104	DIO2
105	DPP4
106	DUS6

107	DYR
108	E2F5
109	EGFR
110	EPHB4
111	ERBB2
112	ERG7
113	ESR1
114	ESR2
115	ESRRA
116	ESRRG
117	EST1
118	EZH2
119	FGFR1
120	FLT3
121	FN1
122	FNTA
123	FOS
124	FOSB
125	FOXO1
126	GATA1
127	GLCM
128	GLO1
129	GRK6
130	GSK3B
131	GSR
132	GSTP1
133	H2AFX
134	HCK
135	HMGB1
136	HMOX1
137	HS90A
138	HSD11B2
139	HSD17B1
140	HSD17B2
141	HSP90AA1
142	HSPA8

143	IGF1
144	IGF1R
145	IGF2
146	IL1B
147	IL2
148	IL6
149	INS
150	IRS1
151	ITGA2
152	JUN
153	JUNB
154	JUND
155	KC1G2
156	KCNK10
157	KDM4E
158	KDR
159	KEAP1
160	KIF11
161	KRAS
162	LCN2
163	LEG7
164	LHCGR
165	LKHA4
166	LYAM3
167	MAOA
168	MAP2K1
169	MAP3K8
170	MAPK1
171	MAPK10
172	MAPK14
173	MAPK3
174	MAPK8
175	MAPK9
176	MCL1
177	MCR
178	MDK

179	MIF
180	MITF
181	MK08
182	MK10
183	MMP1
184	MMP12
185	MMP13
186	MMP2
187	MMP3
188	MMP8
189	MMP9
190	MTAP
191	MTOR
192	MTTP
193	MYC
194	NFATC1
195	NFE2L2
196	NFKB1
197	NFKBIA
198	NFKBIB
199	NGAL
200	NOS1
201	NOS2
202	NOS3
203	NOTCH1
204	NOTCH2
205	NOX4
206	NQO1
207	NR1H2
208	NR1I2
209	NR1I3
210	PARP1
211	PCK1
212	PDE4A
213	PDE4B
214	PDE4D

215	PDE5A
216	PDPK1
217	PH4H
218	PIM1
219	PK3CG
220	PKM
221	PLAU
222	PLSCR1
223	PNPH
224	PON2
225	PPARA
226	PPARG
227	PPIA
228	PPP5C
229	PRGR
230	PRKCA
231	PRKCD
232	PTGES
233	PTGS1
234	PTGS2
235	PTK2
236	PTN1
237	PTPRS
238	PYGL
239	QPCT
240	RB1
241	RELA
242	RPS6KA1
243	RPS6KA2
244	RPS6KA3
245	RXRA
246	S100A7
247	SEPR
248	SERPINE1
249	SHBG
250	SLC2A1

251	SMAD2
252	SOAT1
253	SOAT2
254	SRC
255	STAT1
256	STAT3
257	STK6
258	STS
259	SULT2A1
260	SYK
261	TAF9
262	TBK1
263	THRB
264	TLR4
265	TLR5
266	TMPRSS11D
267	TNFRSF10B
268	TNFRSF11B
269	TNKS
270	TNKS2
271	TOP1
272	TP53
273	TREM1
274	TTHY
275	TTR
276	TYR
277	UGT1A1
278	UGT1A10
279	UGT1A3
280	UGT1A7
281	UGT1A8
282	UGT1A9
283	UGT2B15
284	UGT2B7
285	UGT3A1
286	USP8

287	VEGFA
288	VRK1
289	VTDB
290	XDH

Table S3. NSCLC-related genes

CTD (23,088)	Disgenet (3,926)	Genecards (949)	Total (23,452)
A1BG	A1CF	ABCB1	A1BG
A1BG-AS1	AAMP	ABCC1	A1BG-AS1
A1CF	ABCA1	ABCC2	A1CF
A2M	ABCA3	ABCC3	A2M
A2M-AS1	ABCA4	ABCG2	A2M-AS1
A2ML1	ABCB1	ACHE	A2ML1
A2MP1	ABCB9	ACTA2	A2MP1
A3GALT2	ABCC1	ACTA2-AS1	A3GALT2
A4GALT	ABCC10	ACTR3B	A4GALT
AAA1	ABCC11	ACY1	AAA1
AAAS	ABCC2	ADAM12	AAAS
AACS	ABCC3	ADAM15	AACS
AACSP1	ABCC4	ADAMTS9-AS2	AACSP1
AADAC	ABCC5	AFAP1-AS1	AADAC
AADACL2	ABCC8	AGAP2-AS1	AADACL2
AADACP1	ABCE1	AIMP2	AADAT
AADAT	ABCG1	AKR1B10	AAGAB
AAGAB	ABCG2	AKR1C1	AAK1
AAK1	ABCG4	AKT1	AAMDC
AAMDC	ABL1	AKT2	AAMP
AAMP	ACACA	AKT3	AANAT
AANAT	ACE	ALB	AAR2
AAR2	ACE2	ALDH1A1	AARD
AARD	ACHE	ALDOA	AARS1
AARS1	ACKR3	ALK	AARS2
AARS2	ACLY	ANGPT1	AARSD1
AARSD1	ACOT11	ANGPT2	AASDH
AASDH	ACOT8	ANPEP	AASDHPPT
AASDHPPT	ACSS1	ANXA1	AASS
AASS	ACSS2	ANXA5	AATBC
AATBC	ACTB	AOC3	AATF
AATF	ACTG1	APAF1	AATK
AATK	ACTN4	APC	AAY
AAY	ACVRL1	APEX1	ABAT

ABAT	ACY1	API5	ABCA1
ABCA1	ADA	AQP3	ABCA10
ABCA10	ADA2	ARAF	ABCA11P
ABCA11P	ADAM10	AREG	ABCA12
ABCA12	ADAM15	ARHGAP27P1	ABCA13
ABCA13	ADAM17	ARHGAP5	ABCA14
ABCA14	ADAM19	ASAP1-IT1	ABCA17P
ABCA17P	ADAM22	ASCL1	ABCA1A
ABCA1A	ADAM23	ATM	ABCA2
ABCA2	ADAM28	AURKA	ABCA3
ABCA3	ADAM9	AURKB	ABCA4
ABCA4	ADAMTS1	AXL	ABCA5
ABCA5	ADAMTS13	B2M	ABCA6
ABCA6	ADAMTS5	BAD	ABCA7
ABCA7	ADAMTS8	BAG1	ABCA8
ABCA8	ADARB1	BAK1	ABCA8A
ABCA8A	ADCYAP1	BANCR	ABCA8B
ABCA8B	ADCYAP1R1	BAX	ABCA9
ABCA9	ADD1	BCAR4	ABCB1
ABCB1	ADD3	BCL2	ABCB10
ABCB10	ADGRA2	BCL2L1	ABCB11
ABCB11	ADGRF1	BCL2L11	ABCB1A
ABCB1A	ADGRG6	BCL6	ABCB1B
ABCB1B	ADH1A	BCRP3	ABCB4
ABCB4	ADH1B	BCYRN1	ABCB5
ABCB5	ADH1C	BECN1	ABCB6
ABCB6	ADH7	BID	ABCB6A
ABCB6A	ADIPOQ	BIRC2	ABCB7
ABCB7	ADIPOR1	BIRC3	ABCB8
ABCB8	ADIPOR2	BIRC5	ABCB9
ABCB9	ADM	BIRC7	ABCC1
ABCC1	ADRA1A	BLACAT1	ABCC10
ABCC10	ADRA2B	BMS1P20	ABCC11
ABCC11	AFAP1	BPIFA1	ABCC12
ABCC12	AFAP1-AS1	BRAF	ABCC13
ABCC13	AFAP1L1	BRCA1	ABCC2

ABCC2	AGAP2	BRCA2	ABCC3
ABCC3	AGAP2-AS1	BRD3OS	ABCC4
ABCC4	AGER	BRDT	ABCC5
ABCC5	AGFG1	BRS3	ABCC6
ABCC6	AGL	BSG	ABCC6P1
ABCC6P1	AGO2	C14orf132	ABCC6P2
ABCC6P2	AGR2	C20orf85	ABCC8
ABCC8	AGT	CA9	ABCC9
ABCC9	AHR	CACNA1G-AS1	ABCD1
ABCD1	AHSA1	CACNA2D2	ABCD2
ABCD2	AHSG	CADM1	ABCD3
ABCD3	AICDA	CALB2	ABCD4
ABCD4	AIM2	CASC2	ABCE1
ABCE1	AIMP2	CASC8	ABCF1
ABCF1	AKAP4	CASC9	ABCF2
ABCF2	AKIP1	CASP1	ABCF3
ABCF3	AKR1A1	CASP2	ABCG1
ABCG1	AKR1B10	CASP3	ABCG2
ABCG2	AKR1C1	CASP7	ABCG3L1
ABCG3L1	AKR1C2	CASP8	ABCG3L2
ABCG3L2	AKR1C3	CASP9	ABCG4
ABCG4	AKT1	CAV1	ABCG5
ABCG5	AKT1S1	CAVIN3	ABCG8
ABCG8	AKT2	CBR1	ABHD1
ABHD1	AKT3	CCAT1	ABHD10
ABHD10	ALB	CCAT2	ABHD11
ABHD11	ALCAM	CCKBR	ABHD11-AS1
ABHD11-AS1	ALDH1A1	CCNA1	ABHD12
ABHD12	ALDH1A2	CCNA2	ABHD12B
ABHD12B	ALDH1A3	CCNB1	ABHD13
ABHD13	ALDH1B1	CCND1	ABHD14A
ABHD14A	ALDH3A1	CCND3	ABHD14A-ACY1
ABHD14A-ACY1	ALDH3A2	CCNE1	ABHD14B
ABHD14B	ALDH7A1	CCNE2	ABHD15
ABHD15	ALG1	CCR3	ABHD16A
ABHD16A	ALG13	CCR6	ABHD16B

ABHD16B	ALK	CCR7	ABHD17A
ABHD17A	ALKBH3	CD22	ABHD17AP4
ABHD17AP4	ALKBH5	CD226	ABHD17B
ABHD17B	ALOX15	CD24	ABHD17C
ABHD17C	ALOX15B	CD274	ABHD18
ABHD18	ALOX5	CD40	ABHD2
ABHD2	ALPK1	CD44	ABHD3
ABHD3	ALPK2	CD63	ABHD4
ABHD4	AMACR	CD9	ABHD5
ABHD5	AMELX	CDA	ABHD6
ABHD6	AMFR	CDC25A	ABHD8
ABHD8	AMMECR1	CDC25C	ABI1
ABI1	AMPH	CDC42	ABI2
ABI2	ANAPC10	CDCP1	ABI3
ABI3	ANCR	CDH1	ABI3BP
ABI3BP	ANGPT1	CDH10	ABITRAM
ABITRAM	ANGPT2	CDH13	ABL
ABL	ANGPTL2	CDH2	ABL1
ABL1	ANGPTL4	CDK1	ABL2
ABL2	ANGPTL5	CDK12	ABLIM1
ABLIM1	ANIB1	CDK2	ABLIM2
ABLIM2	ANK1	CDK4	ABLIM3
ABLIM3	ANKRD22	CDK5	ABO
ABO	ANKRD36B	CDK6	ABR
ABR	ANKRD6	CDKN1A	ABRA
ABRA	ANKRD7	CDKN1B	ABRACL
ABRACL	ANLN	CDKN1C	ABRAXAS1
ABRAXAS1	ANO1	CDKN2A	ABRAXAS2
ABRAXAS2	ANP32B	CDKN2A-DT	ABT1
ABT1	ANPEP	CDKN2B	ABTB1
ABTB1	ANXA1	CDKN2B-AS1	ABTB2
ABTB2	ANXA10	CDKN2C	ABTB3
ABTB3	ANXA2	CDX2	ABU-11
ABU-11	ANXA4	CEACAM5	ABU-15
ABU-15	ANXA5	CEACAM7	ABU-2
ABU-2	APAF1	CFLAR	ABU-3

ABU-3	APC	CHEK1	ABU-5
ABU-5	APC2	CHEK2	ABU-6
ABU-6	APEX1	CHGA	ABU-7
ABU-7	API5	CHIAP2	ABU-8
ABU-8	APIP	CHRNA3	ACAA1
ACAA1	APLN	CHRNA5	ACAA1A
ACAA1A	APOA1	CHRNB4	ACAA1B
ACAA1B	APOBEC3B	CHUK	ACAA2
ACAA2	APOE	CLDN7	ACACA
ACACA	APOM	COL18A1	ACACB
ACACB	APP	COL4A2	ACAD10
ACAD10	APPBP2	COL4A3	ACAD11
ACAD11	APRT	COTL1	ACAD8
ACAD8	AQP1	CPS1-IT1	ACAD9
ACAD9	AQP3	CRABP2	ACADL
ACADL	AQP4	CREB1	ACADM
ACADM	AQP5	CREBBP	ACADS
ACADS	AR	CSF2	ACADSB
ACADSB	ARAF	CSF3	ACADVL
ACADVL	ARAP2	CSNK1A1	ACAN
ACAN	AREG	CT83	ACAP1
ACAP1	ARG1	CTAG1B	ACAP2
ACAP2	ARHGAP1	CTLA4	ACAP3
ACAP3	ARHGAP10	CTNNB1	ACAT1
ACAT1	ARHGAP24	CTNND1	ACAT2
ACAT2	ARHGAP27P1	CTSB	ACAT3
ACAT3	ARHGAP5	CTSL	ACBD3
ACBD3	ARHGEF19	CUL3	ACBD4
ACBD4	ARHGEF2	CUL5	ACBD5
ACBD5	ARHGEF39	CXCL12	ACBD6
ACBD6	ARHGEF5	CXCL8	ACBD7
ACBD7	ARHGEF7	CXCR2	ACC
ACC	ARID2	CXCR4	ACCOAS
ACCOAS	ARIH1	CXCR5	ACCS
ACCS	ARL4C	CYCS	ACCSL
ACCSL	ARMC8	CYP1A1	ACD

ACD	ARMCX3	CYP1B1	ACE
ACE	ARMH1	CYP2A13	ACE2
ACE2	ARNT2	CYP2A6	ACER2
ACER2	ARR3	CYP2D6	ACER3
ACER3	ARRB1	CYP2E1	ACHE
ACHE	ARSF	CYP3A4	ACIN1
ACIN1	ARTN	CYTOR	ACIN1A
ACIN1A	ASAP1-IT1	DANCR	ACKR1
ACKR1	ASAP3	DAPK1	ACKR2
ACKR2	ASCC1	DCK	ACKR3
ACKR3	ASCL1	DDB2	ACKR4
ACKR4	ASH1L	DDIAS	ACLY
ACLY	ASIP	DDR2	ACLYA
ACLYA	ASNS	DDX5	ACMSD
ACMSD	ASPA	DGCR5	ACNAT1
ACNAT1	ASPM	DHDH	ACNAT2
ACNAT2	ASS1	DHFR	ACO1
ACO1	ATD	DIABLO	ACO2
ACO2	ATF1	DLEC1	ACOD1
ACOD1	ATF2	DLGAP2	ACOD1LB.L
ACOD1LB.L	ATF3	DLK1	ACOT1
ACOT1	ATF4	DLX6-AS1	ACOT11
ACOT11	ATF6	DMBT1	ACOT12
ACOT12	ATG10	DMTF1	ACOT13
ACOT13	ATG12	DNMT1	ACOT2
ACOT2	ATG16L1	DNMT3A	ACOT3
ACOT3	ATG16L2	DNMT3B	ACOT4
ACOT4	ATG2B	DPYD	ACOT6
ACOT6	ATG3	DPYSL5	ACOT7
ACOT7	ATG5	DRAIC	ACOT8
ACOT8	ATG7	DROSHA	ACOT9
ACOT9	ATM	DUS2	ACOX1
ACOX1	ATN1	DUSP1	ACOX2
ACOX2	ATOX1	DYRK1B	ACOX3
ACOX3	ATP2C1	E2F1	ACOXL
ACOXL	ATP5F1A	E2F2	ACP1

ACP1	ATP6V1E1	E2F3	ACP2
ACP2	ATP7A	E2F5	ACP3
ACP3	ATP7B	ECT2	ACP4
ACP4	ATP8A1	EFNB3	ACP5
ACP5	ATR	EGF	ACP5B
ACP5B	ATRAID	EGFR	ACP6
ACP6	ATRNL1	EGFR-AS1	ACP7
ACP7	ATRX	EGR1	ACPP
ACPP	ATXN7	EIF2AK2	ACR
ACR	AURKA	EIF4E	ACR-11
ACR-11	AURKB	ELAVL4	ACR-14
ACR-14	AVEN	EML4	ACR-15
ACR-15	AVP	ENO1	ACR-16
ACR-16	AVPR2	ENO2	ACR-17
ACR-17	AXIN1	ENPP2	ACR-2
ACR-2	AXL	ENSG00000266919	ACR-20
ACR-20	AZI2	EP300	ACR-21
ACR-21	AZIN1	EPB41L3	ACR-3
ACR-3	AZIN2	EPB41L4A-DT	ACR-5
ACR-5	B2M	EPCAM	ACR-6
ACR-6	B3GALNT1	EPHA2	ACR-7
ACR-7	B3GAT1	EPHA5	ACRBP
ACRBP	B3GNT3	EPHA7	ACS-2
ACS-2	BAAT	EPHX1	ACSBG1
ACSBG1	BABAM2	ERBB2	ACSBG2
ACSBG2	BABAM2-AS1	ERBB3	ACSF2
ACSF2	BACE1	ERBB4	ACSF3
ACSF3	BAD	ERCC1	ACSL
ACSL	BAG1	ERCC2	ACSL1
ACSL1	BAG3	ERCC4	ACSL1B
ACSL1B	BAG4	ERCC6	ACSL3
ACSL3	BAG6	ESR1	ACSL4
ACSL4	BAGE	ESR2	ACSL5
ACSL5	BAK1	EXO1	ACSL6
ACSL6	BAMBI	EZH2	ACSM1
ACSM1	BANCR	F2RL3	ACSM2A

ACSM2A	BAP1	FABP4	ACSM2B
ACSM2B	BARD1	FAR2P1	ACSM3
ACSM3	BARHL2	FAS	ACSM5
ACSM5	BARX2	FASLG	ACSS1
ACSS1	BATF	FASN	ACSS2
ACSS2	BATF2	FAT1	ACSS3
ACSS3	BAX	FBXO5	ACTA1
ACTA1	BAZ1A	FBXW7	ACTA1A
ACTA1A	BBC3	FENDRR	ACTA2
ACTA2	BCAR1	FEZF1-AS1	ACTA2-AS1
ACTA2-AS1	BCAR4	FGA	ACTB
ACTB	BCAT2	FGF2	ACTBL2
ACTBL2	BCL10	FGFR1	ACTC1
ACTC1	BCL11A	FGFR2	ACTC1A
ACTC1A	BCL2	FGFR3	ACTC1B
ACTC1B	BCL2A1	FGFR4	ACTG1
ACTG1	BCL2L1	FGL1	ACTG2
ACTG2	BCL2L11	FHIT	ACTL10
ACTL10	BCL2L2	FLT1	ACTL6A
ACTL6A	BCL3	FLT4	ACTL6B
ACTL6B	BCL6	FN1	ACTL7A
ACTL7A	BCL9	FOS	ACTL7B
ACTL7B	BCR	FOXD2-AS1	ACTL8
ACTL8	BCS1L	FOXK2	ACTL9
ACTL9	BCYRN1	FOXM1	ACTN1
ACTN1	BDNF	FOXO3	ACTN2
ACTN2	BECN1	FOXP3	ACTN3
ACTN3	BHLHE22	FSCN1	ACTN3B
ACTN3B	BHLHE40	GACAT2	ACTN4
ACTN4	BICD2	GADD45A	ACTR10
ACTR10	BID	GADD45B	ACTR1A
ACTR1A	BIN1	GADD45G	ACTR1B
ACTR1B	BIRC2	GAPDH	ACTR2
ACTR2	BIRC3	GART	ACTR3
ACTR3	BIRC5	GAS5	ACTR3B
ACTR3B	BIRC6	GAS5-AS1	ACTR3C

ACTR3C	BIRC7	GAS6-AS1	ACTR5
ACTR5	BLACAT1	GATA2	ACTR6
ACTR6	BLID	GDF15	ACTR8
ACTR8	BMF	GGPS1	ACTRT1
ACTRT1	BMI1	GHET1	ACTRT2
ACTRT2	BMP1	GIMAP6	ACTRT3
ACTRT3	BMP2	GJA1	ACVR1
ACVR1	BMP4	GJB5	ACVR1B
ACVR1B	BMP6	GLI1	ACVR1C
ACVR1C	BMP7	GNAS	ACVR2A
ACVR2A	BMS1	GNAS-AS1	ACVR2B
ACVR2B	BMX	GPC3	ACVR2B-AS1
ACVR2B-AS1	BNIP3	GPRC5A	ACVRL1
ACVRL1	BOK	GPSM2	ACY1
ACY1	BPI	GRP	ACY3
ACY3	BPIFA1	GRPR	ACYP1
ACYP1	BPTF	GSTM1	ACYP2
ACYP2	BRAF	GSTP1	ADA
ADA	BRCA1	GUCY1B2	ADA2
ADA2	BRCA2	H1-0	ADA2A
ADA2A	BRD2	H19	ADAD1
ADAD1	BRD4	H2AC18	ADAD2
ADAD2	BRD7	HAGLR	ADAL
ADAL	BRF2	HAVCR2	ADAM1
ADAM1	BRINP1	HDAC1	ADAM10
ADAM10	BRK1	HDAC9	ADAM11
ADAM11	BRMS1	HDGF	ADAM12
ADAM12	BRMS1L	HGF	ADAM15
ADAM15	BRS3	HIF1A	ADAM17
ADAM17	BSG	HIF1A-AS1	ADAM17A
ADAM17A	BTBD7	HJURP	ADAM18
ADAM18	BTF3P11	HLA-G	ADAM19
ADAM19	BTG1	HMGA2	ADAM1A
ADAM1A	BTG2	HMGB1	ADAM2
ADAM2	BTG3	HNF1A-AS1	ADAM20
ADAM20	BTK	HNRNPA2B1	ADAM20P1

ADAM20P1	BUB1	HOPX	ADAM21
ADAM21	BZW1	HOTAIR	ADAM21P1
ADAM21P1	C10orf90	HOTTIP	ADAM22
ADAM22	C12orf54	HOXA11-AS	ADAM23
ADAM23	C15orf48	HOXB9	ADAM24
ADAM24	C20orf181	HOXC13	ADAM25
ADAM25	C20orf85	HPGD	ADAM26A
ADAM26A	C3	HRAS	ADAM28
ADAM28	C4BPA	HSD17B1	ADAM29
ADAM29	C4BPB	HSF1	ADAM32
ADAM32	C5AR1	HSP90AA1	ADAM33
ADAM33	CA1	HSPA4	ADAM34
ADAM34	CA11	HSPA5	ADAM3A
ADAM3A	CA4	HSPA8	ADAM4
ADAM4	CA8	HSPB1	ADAM5
ADAM5	CA9	ICOSLG	ADAM6A
ADAM6A	CABLES1	ID1	ADAM6B
ADAM6B	CACNA1A	IDH1	ADAM7
ADAM7	CACNA1B	IDH2	ADAM8
ADAM8	CACNA1G-AS1	IFI27	ADAM9
ADAM9	CACNA2D2	IFNAR1	ADAMDEC1
ADAMDEC1	CACUL1	IGF1	ADAMTS1
ADAMTS1	CADM1	IGF1R	ADAMTS10
ADAMTS10	CALB1	IGF2	ADAMTS12
ADAMTS12	CALCA	IGF2-AS	ADAMTS13
ADAMTS13	CALCR	IGF2BP2-AS1	ADAMTS14
ADAMTS14	CALM1	IGFBP3	ADAMTS15
ADAMTS15	CALM2	IKBKB	ADAMTS16
ADAMTS16	CALM3	IL10	ADAMTS16-DT
ADAMTS16-DT	CALR	IL1B	ADAMTS17
ADAMTS17	CAMK1	IL2	ADAMTS18
ADAMTS18	CAMK2D	IL24	ADAMTS19
ADAMTS19	CAMP	IL2RA	ADAMTS2
ADAMTS2	CAND1	IL3	ADAMTS20
ADAMTS20	CAP1	IL4	ADAMTS3
ADAMTS3	CAPN2	IL4R	ADAMTS4

ADAMTS4	CAPNS1	IL6	ADAMTS5
ADAMTS5	CARD10	ILK	ADAMTS6
ADAMTS6	CARD16	ING1	ADAMTS7
ADAMTS7	CARD18	IQANK1	ADAMTS7P1
ADAMTS7P1	CARM1	IRAIN	ADAMTS8
ADAMTS8	CASC11	IREB2	ADAMTS9
ADAMTS9	CASC15	IRF1	ADAMTS9-AS2
ADAMTS9-AS2	CASC19	IRS1	ADAMTSL1
ADAMTSL1	CASC2	IST1	ADAMTSL2
ADAMTSL2	CASC8	ITGA11	ADAMTSL3
ADAMTSL3	CASP1	ITGA2	ADAMTSL4
ADAMTSL4	CASP10	ITGA2B	ADAMTSL5
ADAMTSL5	CASP2	ITGA3	ADAP1
ADAP1	CASP3	ITGA5	ADAP2
ADAP2	CASP4	ITGA6	ADAR
ADAR	CASP7	ITGAE	ADARB1
ADARB1	CASP8	ITGAV	ADARB2
ADARB2	CASP9	ITGB1	ADAT1
ADAT1	CASR	JAK2	ADAT2
ADAT2	CASS4	JAK3	ADAT3
ADAT3	CASZ1	JUN	ADCK1
ADCK1	CAT	JUP	ADCK2
ADCK2	CAV1	KCNMB2-AS1	ADCK3
ADCK3	CAVIN1	KCNQ1OT1	ADCK5
ADCK5	CAVIN2	KDM4C	ADCY1
ADCY1	CBFA2T2	KDR	ADCY10
ADCY10	CBL	KEAP1	ADCY10P1
ADCY10P1	CBLB	KIT	ADCY2
ADCY2	CBLC	KLF6	ADCY3
ADCY3	CBLL1	KMT2D	ADCY4
ADCY4	CBLL2	KRAS	ADCY5
ADCY5	CBR1	KRT18	ADCY6
ADCY6	CBX3	KRT19	ADCY7
ADCY7	CBX4	KRT20	ADCY8
ADCY8	CBX5	KRT7	ADCY9
ADCY9	CBX7	KRT8	ADCYAP1

Table S3

ADCYAP1	CCAT1	LAMA1	ADCYAP1B
ADCYAP1B	CCDC106	LAMA4	ADCYAP1R1
ADCYAP1R1	CCDC54	LAMA5	ADD1
ADD1	CCDC6	LAMB3	ADD2
ADD2	CCDC8	LAMC1	ADD3
ADD3	CCDC85B	LAMC2	ADD3-AS1
ADD3-AS1	CCDC88A	LCAL1	ADGB
ADGB	CCEPR	LGALS1	ADGRA1
ADGRA1	CCK	LIMD1	ADGRA2
ADGRA2	CCL17	LINC00115	ADGRA3
ADGRA3	CCL18	LINC00210	ADGRB1
ADGRB1	CCL19	LINC00261	ADGRB2
ADGRB2	CCL2	LINC00312	ADGRB3
ADGRB3	CCL20	LINC00313	ADGRD1
ADGRD1	CCL21	LINC00319	ADGRD2
ADGRD2	CCL22	LINC00342	ADGRE1
ADGRE1	CCL25	LINC00460	ADGRE2
ADGRE2	CCL4	LINC00472	ADGRE4
ADGRE4	CCL5	LINC00473	ADGRE5
ADGRE5	CCL7	LINC00511	ADGRF1
ADGRF1	CCL8	LINC00635	ADGRF2
ADGRF2	CCN1	LINC00673	ADGRF3
ADGRF3	CCN2	LINC00707	ADGRF4
ADGRF4	CCN4	LINC00857	ADGRF5
ADGRF5	CCN6	LINC00858	ADGRG1
ADGRG1	CCNA2	LINC00880	ADGRG2
ADGRG2	CCNB1	LINC00941	ADGRG3
ADGRG3	CCNB2	LINC00968	ADGRG5
ADGRG5	CCND1	LINC01116	ADGRG6
ADGRG6	CCND2	LINC01133	ADGRG7
ADGRG7	CCND3	LINC01186	ADGRL1
ADGRL1	CCNE1	LINC01433	ADGRL2
ADGRL2	CCNE2	LINC01502	ADGRL3
ADGRL3	CCNH	LINC01511	ADGRL4
ADGRL4	CCNJ	LINC01512	ADGRV1
ADGRV1	CCNY	LINC01513	ADH1

ADH1	CCR1	LINC01589	ADH1A
ADH1A	CCR2	LINC01600	ADH1B
ADH1B	CCR4	LINC01627	ADH1C
ADH1C	CCR6	LINC01628	ADH4
ADH4	CCR7	LINC01852	ADH5
ADH5	CCR9	LINC01969	ADH5P4
ADH5P4	CCT4	LINC02042	ADH6
ADH6	CCT5	LINC02412	ADH6A
ADH6A	CD14	LINC02633	ADH6-PS1
ADH6-PS1	CD151	LINC-ROR	ADH7
ADH7	CD163	LNCR3	ADH8A
ADH8A	CD177	LNCRNA-ATB	ADH8B
ADH8B	CD19	LOC105371114	ADHFE1
ADHFE1	CD1D	LOC730101	ADI1
ADI1	CD200	LOX	ADIG
ADIG	CD24	LRP1B	ADIPOQ
ADIPOQ	CD27	LRRC56	ADIPOR1
ADIPOR1	CD274	LUADT1	ADIPOR2
ADIPOR2	CD276	LUCAT1	ADIRF
ADIRF	CD28	LY6K	ADK
ADK	CD300A	MAFA-AS1	ADM
ADM	CD33	MAGEA1	ADM2
ADM2	CD34	MAGEA3	ADM5
ADM5	CD36	MAGEA4	ADNP
ADNP	CD38	MAGEC2	ADNP2
ADNP2	CD3EAP	MALAT1	ADO
ADO	CD40	MALINC1	ADORA1
ADORA1	CD40LG	MAP2K1	ADORA2A
ADORA2A	CD44	MAP2K2	ADORA2A-AS1
ADORA2A-AS1	CD47	MAP2K4	ADORA2B
ADORA2B	CD55	MAP3K2	ADORA3
ADORA3	CD59	MAP3K8	ADPGK
ADPGK	CD63	MAPK1	ADPGK-AS1
ADPGK-AS1	CD68	MAPK14	ADPRH
ADPRH	CD69	MAPK3	ADPRHL1
ADPRHL1	CD70	MAPK8	ADPRM

ADPRM	CD74	MAX	ADPRS
ADPRS	CD80	MCL1	ADRA1A
ADRA1A	CD82	MCM3	ADRA1B
ADRA1B	CD83	MDM2	ADRA1D
ADRA1D	CD86	MED12	ADRA2A
ADRA2A	CD9	MED19	ADRA2B
ADRA2B	CD93	MEG3	ADRA2C
ADRA2C	CD99	MET	ADRB1
ADRB1	CDA	METTL13	ADRB2
ADRB2	CDC25A	MGMT	ADRB2B
ADRB2B	CDC25B	MIAT	ADRB3
ADRB3	CDC25C	MIF	ADRM1
ADRM1	CDC42	MIR100	ADSL
ADSL	CDC45	MIR101-1	ADSS
ADSS	CDCA3	MIR106A	ADSS1
ADSS1	CDCA5	MIR106B	ADSS2
ADSS2	CDH1	MIR107	ADSSL1
ADSSL1	CDH13	MIR124-1	ADTRP
ADTRP	CDH2	MIR124-3	AEBP1
AEBP1	CDH3	MIR125A	AEBP2
AEBP2	CDH5	MIR125B1	AEN
AEN	CDK1	MIR125B2	AFAP1
AFAP1	CDK14	MIR126	AFAP1-AS1
AFAP1-AS1	CDK15	MIR128-2	AFAP1L1
AFAP1L1	CDK16	MIR129-1	AFAP1L2
AFAP1L2	CDK2	MIR130A	AFDN
AFDN	CDK4	MIR133A1	AFDN-DT
AFDN-DT	CDK5	MIR133B	AFF1
AFF1	CDK6	MIR137	AFF2
AFF2	CDK8	MIR138-1	AFF3
AFF3	CDK9	MIR139	AFF4
AFF4	CDKN1A	MIR140	AFG1L
AFG1L	CDKN1B	MIR141	AFG3L1
AFG3L1	CDKN1C	MIR142	AFG3L1P
AFG3L1P	CDKN2A	MIR143	AFG3L2
AFG3L2	CDKN2B	MIR144	AFM

AFM	CDKN2B-AS1	MIR145	AFMID
AFMID	CDKN2C	MIR146A	AFP
AFP	CDKN3	MIR146B	AFP4
AFP4	CDR1-AS	MIR148A	AFTPH
AFTPH	CDR3	MIR149	AFTPH-DT
AFTPH-DT	CDX2	MIR150	AGA
AGA	CEACAM1	MIR154	AGAP1
AGAP1	CEACAM3	MIR155	AGAP1-IT1
AGAP1-IT1	CEACAM5	MIR15A	AGAP2
AGAP2	CEACAM7	MIR15B	AGAP2-AS1
AGAP3	CEBPA	MIR17	AGAP3
AGAP4	CEBPB	MIR17HG	AGAP4
AGAP6	CELF1	MIR181A1	AGAP6
AGAP7P	CELF2	MIR181C	AGAP7P
AGBL1	CELIAC2	MIR182	AGBL1
AGBL2	CEMIP	MIR183	AGBL2
AGBL3	CENPE	MIR185	AGBL3
AGBL4	CENPU	MIR186	AGBL4
AGBL5	CEP55	MIR18A	AGBL5
AGER	CEP72	MIR191	AGER
AGFG1	CERNA2	MIR192	AGFG1
AGFG1A	CERS6	MIR193A	AGFG1A
AGFG2	CERT1	MIR195	AGFG2
AGGF1	CETN1	MIR196A1	AGGF1
AGK	CFAP97	MIR197	AGK
AGL	CFH	MIR198	AGL
AGMAT	CFHR1	MIR199A1	AGMAT
AGMO	CFI	MIR199B	AGMO
AGO1	CFL1	MIR19A	AGO1
AGO2	CFLAR	MIR200A	AGO2
AGO3	CFTR	MIR200B	AGO3
AGO4	CGA	MIR200C	AGO4
AGPAT1	CHAF1A	MIR203A	AGPAT1
AGPAT2	CHAF1B	MIR204	AGPAT2
AGPAT3	CHAMP1	MIR205	AGPAT3
AGPAT4	CHCHD2	MIR20A	AGPAT4

AGPAT4-IT1	CHD1L	MIR21	AGPAT4-IT1
AGPAT5	CHD5	MIR210	AGPAT5
AGPAT9	CHEK1	MIR211	AGPAT9
AGPS	CHEK2	MIR212	AGPS
AGR2	CHFR	MIR214	AGR2
AGR3	CHGA	MIR216A	AGR3
AGRN	CHI3L1	MIR218-1	AGRN
AGRP	CHKA	MIR218-2	AGRP
AGT	CHKB	MIR219A1	AGT
AGTPBP1	CHL1	MIR22	AGTPBP1
AGTR1	CHMP2B	MIR221	AGTR1
AGTR1A	CHMP4C	MIR222	AGTR1A
AGTR1B	CHODL	MIR223	AGTR1B
AGTR2	CHPF	MIR224	AGTR2
AGTRAP	CHPT1	MIR22HG	AGTRAP
AGXT	CHRM3	MIR23A	AGXT
AGXT2	CHRNA3	MIR24-1	AGXT2
AHCTF1	CHRNA4	MIR24-2	AHCTF1
AHCY	CHRNA5	MIR25	AHCY
AHCYL1	CHRNA9	MIR26A1	AHCYL1
AHCYL2	CHRNB3	MIR27A	AHCYL2
AHDC1	CHRNB4	MIR27B	AHDC1
AHI1	CHUK	MIR296	AHI1
AHI1-DT	CIAPIN1	MIR29A	AHI1-DT
AHNAK	CIB1	MIR29B1	AHNAK
AHNAK2	CIB2	MIR29B2	AHNAK2
AHR	CILK1	MIR29C	AHR
AHR2	CIP2A	MIR30A	AHR2
AHRR	CISD3	MIR30B	AHRR
AHRRA	CIT	MIR30C1	AHRRA
AHRRB	CKAP4	MIR30D	AHRRB
AHSA1	CKAP5	MIR30E	AHSA1
AHSA1B	CKLF	MIR31	AHSA1B
AHSA2	CKS1B	MIR31HG	AHSA2
AHSA2P	CKS1BP7	MIR32	AHSA2P
AHSG	CLDN1	MIR324	AHSG

AHSP	CLDN18	MIR330	AHSP
AICDA	CLDN2	MIR335	AICDA
AIDA.L	CLDN4	MIR338	AIDA.L
AIF1	CLDN5	MIR339	AIF1
AIF1L	CLDN6	MIR33A	AIF1L
AIFM1	CLDN7	MIR340	AIFM1
AIFM2	CLEC2D	MIR345	AIFM2
AIFM3	CLEC4M	MIR34A	AIFM3
AIFM4	CLOCK	MIR34B	AIFM4
AIG1	CLPTM1	MIR34C	AIG1
AIM2	CLPTM1L	MIR361	AIM2
AIMP1	CLSTN1	MIR365A	AIMP1
AIMP2	CLU	MIR372	AIMP2
AIP	CMA1	MIR375	AIP
AIPL1	CMKLR1	MIR377	AIPL1
AIRE	CMPK1	MIR423	AIRE
AIRN	CMTM1	MIR424	AIRN
AJAP1	CMTM6	MIR425	AJAP1
AJM1	CMTM7	MIR429	AJM1
AJUBA	CMYA5	MIR4435-2HG	AJUBA
AK1	CNDP2	MIR451A	AK1
AK1.L	CNKSR3	MIR455	AK1.L
AK1.S	CNOT3	MIR483	AK1.S
AK2	CNPY2	MIR486-1	AK2
AK3	CNR1	MIR497	AK3
AK4	CNTN1	MIR499A	AK4
AK5	CNTN2	MIR503	AK5
AK6	COIL	MIR511	AK6
AK7	COL10A1	MIR574	AK7
AK8	COL11A1	MIR9-1	AK8
AK9	COL11A2	MIR93	AK9
AKAIN1	COL17A1	MIR95	AKAIN1
AKAP1	COL18A1	MIR96	AKAP1
AKAP10	COL1A1	MIR98	AKAP10
AKAP11	COL1A2	MIR99A	AKAP11
AKAP12	COL4A3	MIRLET7A1	AKAP12

AKAP13	COL7A1	MIRLET7A2	AKAP13
AKAP14	COMETT	MIRLET7A3	AKAP14
AKAP17A	COMMD3	MIRLET7B	AKAP17A
AKAP3	COMMD3-BMI1	MIRLET7C	AKAP3
AKAP4	COMMD4	MIRLET7D	AKAP4
AKAP5	COMMD5	MIRLET7E	AKAP5
AKAP6	COMMD6	MIRLET7F1	AKAP6
AKAP7	COMMD8	MIRLET7G	AKAP7
AKAP8	COMMD9	MKI67	AKAP8
AKAP8L	COMT	MLH1	AKAP8L
AKAP9	COPD	MMP1	AKAP9
AKIP1	COPS5	MMP12	AKIP1
AKIRIN1	CORO1C	MMP13	AKIRIN1
AKIRIN2	COTL1	MMP14	AKIRIN2
AKNA	COX1	MMP2	AKNA
AKP3	COX17	MMP3	AKP3
AKR1A1	COX2	MMP7	AKR1A1
AKR1B1	COX5A	MMP9	AKR1B1
AKR1B10	COX7A1	MSH3	AKR1B10
AKR1B15	COX8A	MSLN	AKR1B15
AKR1B3	CP	MTOR	AKR1B3
AKR1B7	CPA4	MUC1	AKR1B7
AKR1B8	CPEB1	MUC16	AKR1B8
AKR1C1	CPEB4	MUC20-OT1	AKR1C1
AKR1C13	CPNE1	MUC3A	AKR1C13
AKR1C14	CPNE3	MUC4	AKR1C14
AKR1C18	CPOX	MUC5AC	AKR1C18
AKR1C19	CPQ	MUC5B	AKR1C19
AKR1C2	CPS1	MUC5B-AS1	AKR1C2
AKR1C20	CPSF3	MVP	AKR1C20
AKR1C3	CR1	MXRA5	AKR1C3
AKR1C4	CRABP2	MYB	AKR1C4
AKR1C6	CRACD	MYC	AKR1C6
AKR1C8	CRAMP1	MYCL	AKR1C8
AKR1CL	CREB1	MYCN	AKR1CL
AKR1D1	CREBBP	MYEF2	AKR1D1

AKR1E1	CREBZF	MYO18B	AKR1E1
AKR1E2	CRISP2	NAPSA	AKR1E2
AKR7A2	CRISPLD2	NAT2	AKR7A2
AKR7A2P1	CRK	NBN	AKR7A2P1
AKR7A3	CRKL	NCAM1	AKR7A3
AKR7A5	CRLS1	NCOR1	AKR7A5
AKT1	CRMP1	NEAT1	AKT1
AKT1S1	CRNDE	NECTIN4	AKT1S1
AKT2	CRP	NEXN-AS1	AKT2
AKT3	CRTAP	NFE2L2	AKT3
AKTIP	CRTC2	NFKB1	AKTIP
AKTS1	CRYAB	NFKBIA	AKTS1
ALAD	CRYBG3	NKILA	ALAD
ALAS1	CRYZ	NKX2-1	ALAS1
ALAS2	CSF1	NMB	ALAS2
ALB	CSF1R	NMBR	ALB
ALB1	CSF2	NME1	ALB1
ALCAM	CSF3	NMRAL2P	ALCAM
ALDH	CSH1	NNMT	ALDH
ALDH1	CSH2	NOD2	ALDH1
ALDH16A1	CSK	NOP2	ALDH16A1
ALDH18A1	CSNK2A2	NOS2	ALDH18A1
ALDH1A1	CSNK2A3	NOTCH1	ALDH1A1
ALDH1A2	CST1	NOVA1	ALDH1A2
ALDH1A3	CST6	NPRL2	ALDH1A3
ALDH1A3-AS1	CSTA	NPTN-IT1	ALDH1A3-AS1
ALDH1A7	CSTF2	NQO1	ALDH1A7
ALDH1B1	CT45A2	NRAS	ALDH1B1
ALDH1L1	CT83	NRG1	ALDH1L1
ALDH1L2	CTAG1A	NRP1	ALDH1L2
ALDH2	CTAG1B	NSD1	ALDH2
ALDH2.2	CTBP2	NSD3	ALDH2.2
ALDH3A1	CTC1	OGG1	ALDH3A1
ALDH3A2	CTDSPL	PANDAR	ALDH3A2
ALDH3B1	CTHRC1	PARP1	ALDH3B1
ALDH3B2	CTLA4	PAX9	ALDH3B2

ALDH4A1	CTNNB1	PCAT1	ALDH4A1
ALDH5A1	CTNND1	PCAT29	ALDH5A1
ALDH6A1	CTNND2	PCAT6	ALDH6A1
ALDH7A1	CTSB	PCAT7	ALDH7A1
ALDH8A1	CTSD	PCBP2-OT1	ALDH8A1
ALDH9A1	CTSL	PCLAF	ALDH9A1
ALDH9A1A.1	CTSS	PCNA	ALDH9A1A.1
ALDOA	CTTN	PDCD1	ALDOA
ALDOAB	CUL3	PDCD1LG2	ALDOAB
ALDOB	CUL4A	PDCD4	ALDOB
ALDOC	CUL4B	PDGFB	ALDOC
ALDOCA	CUL7	PDGFRA	ALDOCA
ALG1	CUX1	PDGFRB	ALG1
ALG10	CX3CL1	PDK1	ALG10
ALG10B	CX3CR1	PDPN	ALG10B
ALG11	CXADR	PEBP1	ALG11
ALG12	CXADRP1	PECAM1	ALG12
ALG13	CXCL1	PGBD3	ALG13
ALG14	CXCL10	PIK3C3	ALG14
ALG1L	CXCL11	PIK3CA	ALG1L
ALG1L9P	CXCL12	PIK3CB	ALG1L9P
ALG2	CXCL13	PIK3CG	ALG2
ALG3	CXCL14	PIK3R1	ALG3
ALG6	CXCL16	PIK3R2	ALG6
ALG8	CXCL5	PIK3R3	ALG8
ALG9	CXCL6	PIP	ALG9
ALK	CXCL8	PLA2G1B	ALK
ALKAL2	CXCL9	PLAC9P1	ALKAL2
ALKBH1	CXCR1	PLAG1	ALKBH1
ALKBH2	CXCR2	PLAU	ALKBH2
ALKBH3	CXCR3	PLAUR	ALKBH3
ALKBH4	CXCR4	PLCG1	ALKBH4
ALKBH5	CXCR5	PLK1	ALKBH5
ALKBH6	CXCR6	POLK	ALKBH6
ALKBH7	CYB561D2	POSTN	ALKBH7
ALKBH8	CYBB	POU5F1	ALKBH8

ALMS1	CYGB	PPARG	ALMS1
ALMS1-IT1	CYLD	PPIEL	ALMS1-IT1
ALMS1P1	CYP17A1	PPP2R1A	ALMS1P1
ALOX12	CYP19A1	PPP2R1B	ALOX12
ALOX12B	CYP1A1	PPP2R2A	ALOX12B
ALOX12E	CYP1A2	PPP6C	ALOX12E
ALOX12P2	CYP1B1	PRAL	ALOX12P2
ALOX15	CYP24A1	PRDM14	ALOX15
ALOX15B	CYP27A1	PRDX1	ALOX15B
ALOX5	CYP27B1	PRKAA1	ALOX5
ALOX5AP	CYP2A13	PRKCA	ALOX5AP
ALOXE3	CYP2A6	PRKCB	ALOXE3
ALPG	CYP2B6	PRKCI	ALPG
ALPI	CYP2C19	PRKCZ-AS1	ALPI
ALPK1	CYP2C8	PRKN	ALPK1
ALPK2	CYP2C9	PROM1	ALPK2
ALPK3	CYP2D6	PTEN	ALPK3
ALPL	CYP2E1	PTGER4	ALPL
ALPP	CYP2R1	PTGS2	ALPP
ALPPL2	CYP3A4	PTHLH	ALPPL2
ALS2	CYP3A5	PTK2	ALS2
ALS2CL	CYP3A51P	PTK2B	ALS2CL
ALT	CYP4F3	PTPN11	ALT
ALX1	CYTIP	PTPN13	ALX1
ALX3	CYTOR	PTPRC	ALX3
ALX4	DAB1	PTPRG	ALX4
ALYREF	DAB2	PTRH2	ALYREF
AMACR	DAB2IP	PVT1	AMACR
AMBN	DACH1	PXN	AMBN
AMBP	DANCR	RAC1	AMBP
AMBRA1	DAND5	RACK1	AMBRA1
AMD	DAP	RAD51	AMD
AMD1	DAPK1	RAF1	AMD1
AMDHD1	DAPK3	RALBP1	AMDHD1
AMDHD2	DBN1	RARA	AMDHD2
AMELX	DCAF1	RARB	AMELX

AMER1	DCBLD1	RARS1	AMER1
AMER2	DCC	RASA1	AMER2
AMFR	DCDC2	RASSF1	AMFR
AMH	DCK	RASSF5	AMH
AMHR2	DCLK1	RB1	AMHR2
AMIGO1	DCN	RBM38	AMIGO1
AMIGO2	DCR	RBM5	AMIGO2
AMIGO3	DCTN4	RBM6	AMIGO3
AMMECR1	DCTN6	RCVRN	AMMECR1
AMMECR1L	DCUN1D1	RECK	AMMECR1L
AMN	DDB1	RELA	AMN
AMN1	DDB2	RET	AMN1
AMOT	DDH2	RGMB-AS1	AMOT
AMOTL1	DDIAS	RHOA	AMOTL1
AMOTL2	DDIT3	RHOB	AMOTL2
AMPD1	DDIT4	RIOX1	AMPD1
AMPD2	DDR1	RIOX2	AMPD2
AMPD3	DDR2	RMRP	AMPD3
AMPH	DDT	RNH1	AMPH
AMT	DDX17	RNY1	AMT
AMTN	DDX23	RNY3	AMTN
AMY1	DDX3X	ROBO1	AMY1
AMY1A	DDX43	ROCK1	AMY1A
AMY2A	DDX5	ROS1	AMY2A
AMY2A5	DDX51	RPLP0P2	AMY2A5
AMY2B	DDX53	RPS6KB1	AMY2B
AMZ1	DDX56	RPSA	AMZ1
AMZ2	DDX58	RRM1	AMZ2
AMZ2P1	DECR1	RRM2	AMZ2P1
ANAPC1	DEFB1	RUNX3	ANAPC1
ANAPC10	DEK	RXRA	ANAPC10
ANAPC11	DENND2D	RXRB	ANAPC11
ANAPC13	DENR	RXRG	ANAPC13
ANAPC15	DEPDC1B	S100A2	ANAPC15
ANAPC16	DERL1	S100A4	ANAPC16
ANAPC2	DES	SAA1	ANAPC2

ANAPC4	DEUP1	SAT1	ANAPC4
ANAPC5	DGCR5	SBF2-AS1	ANAPC5
ANAPC7	DHCR24	SCGB1A1	ANAPC7
ANCE	DHDDS	SCGB2A2	ANCE
AND1	DHDH	SCUBE3	ANCR
AND2	DHFR	SEMA3B	AND1
ANG	DHX15	SEMA3F	AND2
ANG5	DHX29	SERPINA3	ANG
ANGEL1	DHX9	SERPINB3	ANG5
ANGEL2	DIABLO	SERPINB5	ANGEL1
ANGPT1	DICER1	SETD2	ANGEL2
ANGPT2	DIP2A	SEZ6L	ANGPT1
ANGPT4	DISC1	SEZ6L2	ANGPT2
ANGPTL1	DISP1	SFTA1P	ANGPT4
ANGPTL2	DKK1	SFTPA1	ANGPTL1
ANGPTL3	DKK3	SFTPA2	ANGPTL2
ANGPTL4	DKK4	SFTPB	ANGPTL3
ANGPTL5	DLC1	SFTPC	ANGPTL4
ANGPTL6	DLEC1	SFTPD	ANGPTL5
ANGPTL7	DLEU1	SGO1-AS1	ANGPTL6
ANGPTL8	DLEU2	SHC1	ANGPTL7
ANK	DLG3	SIRT3	ANGPTL8
ANK1	DLGAP2	SIX1	ANIB1
ANK2	DLGAP5	SKP1	ANK
ANK3	DLK1	SKP2	ANK1
ANKAR	DLL1	SLC16A1-AS1	ANK2
ANKDD1A	DLL3	SLC19A1	ANK3
ANKEF1	DLL4	SLC22A18	ANKAR
ANKFN1	DLX6	SLC2A1	ANKDD1A
ANKFY1	DLX6-AS1	SLFN11	ANKEF1
ANKH	DMBT1	SLMAP	ANKFN1
ANKHD1	DMRT1	SMAD2	ANKFY1
ANKIB1	DMTF1	SMAD3	ANKH
ANKK1	DNAH8	SMAD4	ANKHD1
ANKLE1	DNAJA3	SMARCA4	ANKIB1
ANKLE2	DNAJB1	SMIM31	ANKK1

ANKMY1	DNAJB4	SNAI1	ANKLE1
ANKMY2	DNASE2	SNAI2	ANKLE2
ANKRA2	DNER	SNHG1	ANKMY1
ANKRD1	DNM1L	SNHG12	ANKMY2
ANKRD10	DNM1P50	SNHG15	ANKRA2
ANKRD10-IT1	DNMT1	SNHG16	ANKRD1
ANKRD11	DNMT3A	SNHG20	ANKRD10
ANKRD12	DNMT3B	SNHG7	ANKRD10-IT1
ANKRD13A	DNTT	SOD2	ANKRD11
ANKRD13B	DNTTIP1	SOS1	ANKRD12
ANKRD13C	DOCK3	SOX2	ANKRD13A
ANKRD13D	DOK4	SOX2-OT	ANKRD13B
ANKRD16	DPP10	SOX30	ANKRD13C
ANKRD17	DPP4	SP1	ANKRD13D
ANKRD18A	DPP9	SPAAR	ANKRD16
ANKRD18B	DPPA4	SPAG9	ANKRD17
ANKRD18DP	DPYD	SPARC	ANKRD18A
ANKRD19P	DPYSL2	SPP1	ANKRD18B
ANKRD1B	DRAM2	SPRY4-IT1	ANKRD18DP
ANKRD2	DRD2	SRC	ANKRD19P
ANKRD2.L	DRD4	SRPK1	ANKRD1B
ANKRD20A1	DROSHA	SSTR2	ANKRD2
ANKRD20A11P	DSC2	STAG3L2	ANKRD2.L
ANKRD20A12P	DSC3	STAT1	ANKRD20A1
ANKRD20A2P	DSCAM-AS1	STAT3	ANKRD20A11P
ANKRD20A3P	DSG2	STK11	ANKRD20A12P
ANKRD20A4P	DSG3	STK4	ANKRD20A2P
ANKRD20A5P	DSP	STMN1	ANKRD20A3P
ANKRD20A8P	DSPP	SYP	ANKRD20A4P
ANKRD20A9P	DSTN	TATDN1	ANKRD20A5P
ANKRD22	DTD1	TCF7	ANKRD20A8P
ANKRD23	DTX1	TCIM	ANKRD20A9P
ANKRD24	DTX3	TERC	ANKRD22
ANKRD26	DUS2	TERT	ANKRD23
ANKRD26P1	DUSP1	TFAP2A	ANKRD24
ANKRD26P3	DUSP2	TFPI2	ANKRD26

ANKRD27	DUSP3	TGFA	ANKRD26P1
ANKRD28	DUSP4	TGFB1	ANKRD26P3
ANKRD29	DUSP6	TGFBI	ANKRD27
ANKRD30A	DUT	TGFBR1	ANKRD28
ANKRD30B	DUXAP10	TGFBR2	ANKRD29
ANKRD30BL	DUXAP8	THBS1	ANKRD30A
ANKRD30BP2	DUXAP9	THY1	ANKRD30B
ANKRD33	DVL1	TIMELESS	ANKRD30BL
ANKRD33B	DVL1P1	TIMP1	ANKRD30BP2
ANKRD34A	DVL3	TIMP2	ANKRD33
ANKRD34B	DYNLL1	TIMP3	ANKRD33B
ANKRD34C	DYNLL2	TINCR	ANKRD34A
ANKRD35	DYNLRB1	TK1	ANKRD34B
ANKRD36	DYRK1A	TKT	ANKRD34C
ANKRD36B	DYRK1B	TLCD3A	ANKRD35
ANKRD36BP1	DYRK2	TLE1	ANKRD36
ANKRD36BP2	E2F1	TLR4	ANKRD36B
ANKRD36C	E2F2	TLR9	ANKRD36BP1
ANKRD37	E2F3	TNF	ANKRD36BP2
ANKRD39	E2F5	TNFRSF10A	ANKRD36C
ANKRD40	E2F6	TNFRSF10B	ANKRD37
ANKRD42	E2F7	TNFRSF10D	ANKRD39
ANKRD44	E2F8	TNFSF10	ANKRD40
ANKRD44-AS1	EBAG9	TOB1	ANKRD42
ANKRD45	EBI3	TOP1	ANKRD44
ANKRD46	EBNA1BP2	TOP2A	ANKRD44-AS1
ANKRD49	ECM1	TP53	ANKRD45
ANKRD50	ECSCR	TP53BP2	ANKRD46
ANKRD52	ECT2	TP53COR1	ANKRD49
ANKRD53	EDARADD	TP53TG1	ANKRD50
ANKRD54	EDIL3	TP63	ANKRD52
ANKRD55	EDN1	TP73	ANKRD53
ANKRD6	EDNRA	TP73-AS1	ANKRD54
ANKRD60	EDNRB	TRAF1	ANKRD55
ANKRD63	EEF1A2	TRAF2	ANKRD6
ANKRD65	EEF1B2P2	TRAF3	ANKRD60

ANKRD66	EEF1E1	TRAF4	ANKRD63
ANKRD9	EEF2K	TRAF6	ANKRD65
ANKS1	EFEMP1	TRIP13	ANKRD66
ANKS1A	EFEMP2	TRPM2-AS	ANKRD7
ANKS1B	EFNA1	TTK	ANKRD9
ANKS3	EFNA3	TUBA4B	ANKS1
ANKS4B	EFNB3	TUBB	ANKS1A
ANKS6	EGF	TUG1	ANKS1B
ANKUB1	EGFL7	TUSC1	ANKS3
ANKZF1	EGFR	TUSC2	ANKS4B
ANLN	EGFR-AS1	TUSC7	ANKS6
ANO1	EGLN2	TUT1	ANKUB1
ANO10	EGLN3	TWIST1	ANKZF1
ANO2	EGR1	TXN	ANLN
ANO3	EGR2	TYMP	ANO1
ANO4	EGR3	TYMS	ANO10
ANO5	EGR4	U2AF1	ANO2
ANO6	EHD1	UBA7	ANO3
ANO7	EHMT2	UBE2K	ANO4
ANO8	EI24	UCA1	ANO5
ANO9	EIF2AK2	UCHL1	ANO6
ANO9A	EIF2AK3	UGT1A1	ANO7
ANOS1	EIF2S1	USF2	ANO8
ANP32A	EIF2S2	USP33	ANO9
ANP32A-IT1	EIF2S3	USP8	ANO9A
ANP32B	EIF3A	VDAC1	ANOS1
ANP32C	EIF3B	VEGFA	ANP32A
ANP32D	EIF3C	VEGFC	ANP32A-IT1
ANP32E	EIF3D	VEGFD	ANP32B
ANPEP	EIF3E	WEE1	ANP32C
ANTKMT	EIF3H	WIF1	ANP32D
ANTXR1	EIF3J	WWP2	ANP32E
ANTXR2	EIF4A1	XIAP	ANPEP
ANTXRL	EIF4A2	XIST	ANTKMT
ANXA1	EIF4A3	XPA	ANTXR1
ANXA10	EIF4E	XRCC1	ANTXR2

ANXA11	EIF4E2	XRCC3	ANTXRL
ANXA13	EIF4EBP1	XRCC5	ANXA1
ANXA1A	EIF4G1	XRCC6	ANXA10
ANXA2	EIF5A	YAP1	ANXA11
ANXA2P1	EIF5A2	YBX1	ANXA13
ANXA2P2	EIF6	YWHAE	ANXA1A
ANXA2P3	ELAC2	YY1	ANXA2
ANXA2R	ELANE	ZEB1	ANXA2P1
ANXA3	ELAVL1	ZEB2	ANXA2P2
ANXA4	ELAVL2	ZEB2-AS1	ANXA2P3
ANXA5	ELF3	ZFAS1	ANXA2R
ANXA5B	ELK1	ZNF295-AS1	ANXA3
ANXA6	ELMO3	ZNF461	ANXA4
ANXA7	ELN	ZNF793	ANXA5
ANXA8	ELP1	ZNRD1ASP	ANXA5B
ANXA8L1	ELP3		ANXA6
ANXA9	EMD		ANXA7
AOAH	EML4		ANXA8
AOC1	EMP1		ANXA8L1
AOC2	EMP3		ANXA9
AOC3	EN2		AOAH
AOPEP	ENO1		AOC1
AOX1	ENO2		AOC2
AOX3	ENPEP		AOC3
AP1AR	ENTPD1		AOPEP
AP1B1	EOMES		AOX1
AP1G1	EP300		AOX3
AP1G2	EPAS1		AP1AR
AP1M1	EPB41L3		AP1B1
AP1M2	EPB41L4A-DT		AP1G1
AP1S1	EPCAM		AP1G2
AP1S2	EPG5		AP1M1
AP1S3	EPHA2		AP1M2
AP2A1	EPHA7		AP1S1
AP2A2	EPHB2		AP1S2
AP2B1	EPHB3		AP1S3

AP2M1	EPHB4	AP2A1
AP2S1	EPHB6	AP2A2
AP3B1	EPHX1	AP2B1
AP3B2	EPN3	AP2M1
AP3D1	EPO	AP2S1
AP3M1	EPOR	AP3B1
AP3M2	EPS15	AP3B2
AP3S1	EPS8	AP3D1
AP3S2	ERAP1	AP3M1
AP4B1	ERBB2	AP3M2
AP4E1	ERBB3	AP3S1
AP4M1	ERBB4	AP3S2
AP4S1	ERCC1	AP4B1
AP5B1	ERCC2	AP4E1
AP5M1	ERCC4	AP4M1
AP5S1	ERCC5	AP4S1
AP5Z1	EREG	AP5B1
APAF1	ERG	AP5M1
APBA1	ERGIC3	AP5S1
APBA2	ERN1	AP5Z1
APBA3	ERO1A	APAF1
APBB1	ERRFI1	APBA1
APBB1IP	ERVK-10	APBA2
APBB2	ERVK-18	APBA3
APBB3	ERVK-19	APBB1
APC	ERVK-21	APBB1IP
APC2	ERVK-24	APBB2
APCDD1	ERVK-25	APBB3
APCDD1L	ERVK-7	APC
APCS	ERVK-8	APC2
APEG3	ERVK-9	APCDD1
APEH	ERVW-1	APCDD1L
APELA	ESD	APCS
APEX1	ESM1	APEG3
APEX2	ESPL1	APEH
APH1A	ESR1	APELA

APH1B	ESR2	APEX1
API5	ESRP1	APEX2
APIP	ESRRA	APH1A
APLF	ESRRB	APH1B
APLN	ESRRG	API5
APLNR	ESYT1	APIP
APLP1	ETFA	APLF
APLP2	ETS1	APLN
APMAP	ETS2	APLNR
APOA1	ETV4	APLP1
APOA2	EXO1	APLP2
APOA4	EXTL3	APMAP
APOA4B.3	EYA2	APOA1
APOA5	EYA4	APOA2
APOB	EZH1	APOA4
APOBEC1	EZH2	APOA4B.3
APOBEC3A	EZR	APOA5
APOBEC3B	F11R	APOB
APOBEC3C	F2R	APOBEC1
APOBEC3D	F3	APOBEC3A
APOBEC3F	FAAH	APOBEC3B
APOBEC3G	FADD	APOBEC3C
APOBEC3H	FAF1	APOBEC3D
APOBEC4	FAIM2	APOBEC3F
APOBR	FALEC	APOBEC3G
APOC1	FAM107A	APOBEC3H
APOC2	FAM13A	APOBEC4
APOC3	FAM201A	APOBR
APOC4	FAM83A	APOC1
APOD	FAM83A-AS1	APOC2
APOE	FAM83B	APOC3
APOF	FAM83F	APOC4
APOH	FAM98A	APOD
APOL1	FANCG	APOE
APOL10B	FAP	APOF
APOL2	FARP2	APOH

APOL3	FAS	APOL1
APOL4	FAS-AS1	APOL10B
APOL5	FASLG	APOL2
APOL6	FASN	APOL3
APOL7C	FASTK	APOL4
APOL8	FBLIM1	APOL5
APOL9A	FBLN5	APOL6
APOLD1	FBN2	APOL7C
APOM	FBP1	APOL8
APON	FBRS	APOL9A
APOO	FBXL19	APOLD1
APOOL	FBXL19-AS1	APOM
APOOP5	FBXL3	APON
APOV1	FBXO17	APOO
APP	FBXO6	APOOL
APPBP2	FBXO7	APOOP5
APPL1	FBXO8	APOV1
APPL2	FBXW11	APP
APRG1	FBXW4	APPBP2
APRT	FBXW5	APPL1
APTR	FBXW7	APPL2
APTX	FCGBP	APRG1
AQP1	FCGR3A	APRT
AQP10	FCGR3B	APTR
AQP11	FCGRT	APTX
AQP12A	FCHSD2	AQP1
AQP12B	FDX1	AQP10
AQP1A.1	FEN1	AQP11
AQP2	FENDRR	AQP12A
AQP3	FER	AQP12B
AQP3A	FEZF1	AQP1A.1
AQP4	FEZF1-AS1	AQP2
AQP4-AS1	FEZF2	AQP3
AQP5	FGF13	AQP3A
AQP5-AS1	FGF18	AQP4
AQP6	FGF2	AQP4-AS1

AQP7	FGF5	AQP5
AQP7P1	FGF9	AQP5-AS1
AQP7P2	FGFR1	AQP6
AQP8	FGFR1OP	AQP7
AQP8A.1	FGFR2	AQP7P1
AQP9	FGFR3	AQP7P2
AQR	FGFR4	AQP8
AR	FGG	AQP8A.1
AR.L	FHIT	AQP9
ARAF	FHL1	AQR
ARAP1	FKBP3	AR
ARAP2	FLI1	AR.L
ARAP3	FLNA	ARAF
ARC	FLOT1	ARAP1
ARCN1	FLOT2	ARAP2
AREG	FLT1	ARAP3
AREL1	FLT3	ARC
ARF1	FLT4	ARCN1
ARF1P1	FLVCR1	AREG
ARF3	FLVCR1-DT	AREL1
ARF4	FMNL1	ARF1
ARF5	FN1	ARF1P1
ARF6	FNDC5	ARF3
ARFGAP1	FOLH1	ARF4
ARFGAP2	FOLR1	ARF5
ARFGAP3	FOLR2	ARF6
ARFGEF1	FOS	ARFGAP1
ARFGEF2	FOSB	ARFGAP2
ARFGEF3	FOSL1	ARFGAP3
ARFIP1	FOSL2	ARFGEF1
ARFIP2	FOXA1	ARFGEF2
ARFRP1	FOXA2	ARFGEF3
ARG1	FOXC1	ARFIP1
ARG2	FOXC2	ARFIP2
ARGK-1	FOXC2-AS1	ARFRP1
ARGLU1	FOXD1	ARG1

ARHGAP1	FOXD2	ARG2
ARHGAP10	FOXD2-AS1	ARGK-1
ARHGAP11A	FOXD3	ARGLU1
ARHGAP12	FOXE1	ARHGAP1
ARHGAP15	FOXF2	ARHGAP10
ARHGAP17	FOXG1	ARHGAP11A
ARHGAP18	FOXJ2	ARHGAP12
ARHGAP19	FOXJ3	ARHGAP15
ARHGAP20	FOXK2	ARHGAP17
ARHGAP21	FOXM1	ARHGAP18
ARHGAP22	FOXN1	ARHGAP19
ARHGAP23	FOXO1	ARHGAP20
ARHGAP24	FOXO3	ARHGAP21
ARHGAP25	FOXO4	ARHGAP22
ARHGAP26	FOXP1	ARHGAP23
ARHGAP27	FOXP2	ARHGAP24
ARHGAP27P1	FOXP3	ARHGAP25
ARHGAP28	FOXP4	ARHGAP26
ARHGAP29	FOXP4-AS1	ARHGAP27
ARHGAP29B	FOXQ1	ARHGAP27P1
ARHGAP30	FOXR2	ARHGAP28
ARHGAP31	FPR2	ARHGAP29
ARHGAP31-AS1	FRAT1	ARHGAP29B
ARHGAP32	FRK	ARHGAP30
ARHGAP33	FRMD3	ARHGAP31
ARHGAP35	FRS2	ARHGAP31-AS1
ARHGAP36	FRS3	ARHGAP32
ARHGAP39	FRZB	ARHGAP33
ARHGAP4	FSCN1	ARHGAP35
ARHGAP40	FSD1	ARHGAP36
ARHGAP42	FSD1L	ARHGAP39
ARHGAP44	FSIP1	ARHGAP4
ARHGAP45	FSTL1	ARHGAP40
ARHGAP5	FTO	ARHGAP42
ARHGAP5-AS1	FURIN	ARHGAP44
ARHGAP6	FUS	ARHGAP45

ARHGAP8	FUT4	ARHGAP5
ARHGAP9	FUT8	ARHGAP5-AS1
ARHGDIA	FUZ	ARHGAP6
ARHGDIB	FXR1	ARHGAP8
ARHGDIG	FZD1	ARHGAP9
ARHGEF1	FZD4	ARHGDIA
ARHGEF10	FZD5	ARHGDIB
ARHGEF10L	FZD8	ARHGDIG
ARHGEF11	FZD9	ARHGEF1
ARHGEF12	G0S2	ARHGEF10
ARHGEF15	G3BP1	ARHGEF10L
ARHGEF16	G6PD	ARHGEF11
ARHGEF17	GAB1	ARHGEF12
ARHGEF18	GAB2	ARHGEF15
ARHGEF19	GABPA	ARHGEF16
ARHGEF2	GABPB1-IT1	ARHGEF17
ARHGEF25	GACAT3	ARHGEF18
ARHGEF26	GADD45A	ARHGEF19
ARHGEF26-AS1	GADD45B	ARHGEF2
ARHGEF28	GADD45G	ARHGEF25
ARHGEF3	GADL1	ARHGEF26
ARHGEF33	GAGE1	ARHGEF26-AS1
ARHGEF35	GALC	ARHGEF28
ARHGEF37	GALNS	ARHGEF3
ARHGEF38	GALNT14	ARHGEF33
ARHGEF38-IT1	GAP43	ARHGEF35
ARHGEF39	GAPDH	ARHGEF37
ARHGEF3-AS1	GAPLINC	ARHGEF38
ARHGEF4	GART	ARHGEF38-IT1
ARHGEF40	GAS5	ARHGEF39
ARHGEF5	GAS5-AS1	ARHGEF3-AS1
ARHGEF6	GAS6	ARHGEF4
ARHGEF7	GAS6-AS1	ARHGEF40
ARHGEF7-IT1	GASAL1	ARHGEF5
ARHGEF9	GAST	ARHGEF6
ARHGEF9A	GATA2	ARHGEF7

ARID1A	GATA2-AS1	ARHGEF7-IT1
ARID1B	GATA3	ARHGEF9
ARID2	GATA4	ARHGEF9A
ARID3A	GATA6	ARID1A
ARID3B	GCA	ARID1B
ARID3C	GCLC	ARID2
ARID4A	GCLM	ARID3A
ARID4B	GCNT3	ARID3B
ARID5A	GCSAM	ARID3C
ARID5B	GDE1	ARID4A
ARIH1	GDF10	ARID4B
ARIH2	GDF15	ARID5A
ARIH2OS	GDF5	ARID5B
ARL1	GDI2	ARIH1
ARL10	GDNF	ARIH2
ARL11	GEM	ARIH2OS
ARL13A	GEMIN2	ARL1
ARL13B	GFAP	ARL10
ARL14	GFPT2	ARL11
ARL14EP	GFRA3	ARL13A
ARL14EPL	GHET1	ARL13B
ARL15	GHRH	ARL14
ARL16	GHSR	ARL14EP
ARL17A	GINS1	ARL14EPL
ARL2	GINS2	ARL15
ARL2BP	GINS4	ARL16
ARL3	GIPR	ARL17A
ARL4A	GJA1	ARL2
ARL4C	GJB1	ARL2BP
ARL4D	GJB2	ARL3
ARL5A	GK5	ARL4A
ARL5B	GLDC	ARL4C
ARL6	GLI1	ARL4D
ARL6IP1	GLI2	ARL5A
ARL6IP4	GLI3	ARL5B
ARL6IP5	GLIPR1	ARL6

ARL6IP5A	GLRA1	ARL6IP1
ARL6IP5B	GLS	ARL6IP4
ARL6IP6	GLUL	ARL6IP5
ARL8A	GMEB1	ARL6IP5A
ARL8B	GML	ARL6IP5B
ARL9	GNA12	ARL6IP6
ARMC1	GNA15	ARL8A
ARMC10	GNAS	ARL8B
ARMC2	GNAS-AS1	ARL9
ARMC3	GOLM1	ARMC1
ARMC5	GOLPH3	ARMC10
ARMC6	GORASP1	ARMC2
ARMC7	GOT1	ARMC3
ARMC8	GPBAR1	ARMC5
ARMC9	GPC3	ARMC6
ARMCX1	GPC5	ARMC7
ARMCX2	GPER1	ARMC8
ARMCX3	GPKOW	ARMC9
ARMCX4	GPR151	ARMCX1
ARMCX5	GPR166P	ARMCX2
ARMCX6	GPR35	ARMCX3
ARMH1	GPR42	ARMCX4
ARMH3	GPR55	ARMCX5
ARMH4	GPRC5A	ARMCX6
ARMT1	GPRC6A	ARMH1
ARNT	GPT	ARMH3
ARNT2	GPX1	ARMH4
ARNTL	GPX3	ARMT1
ARNTL2	GPX4	ARNT
ARP5	GRAP2	ARNT2
ARPC1A	GRB2	ARNTL
ARPC1B	GREB1	ARNTL2
ARPC2	GRHL2	ARP5
ARPC3	GRIA2	ARPC1A
ARPC4	GRIA3	ARPC1B
ARPC5	GRIK2	ARPC2

ARPC5L	GRIN2B	ARPC3
ARPIN	GRK5	ARPC4
ARPIN-AP3S2	GRM1	ARPC5
ARPP19	GRN	ARPC5L
ARPP21	GRP	ARPIN
ARR3	GRPR	ARPIN-AP3S2
ARRB1	GSDMD	ARPP19
ARRB2	GSK3B	ARPP21
ARRDC1	GSN	ARR3
ARRDC1-AS1	GSPT1	ARRB1
ARRDC2	GSR	ARRB2
ARRDC3	GSTK1	ARRDC1
ARRDC3-AS1	GSTM1	ARRDC1-AS1
ARRDC4	GSTM2	ARRDC2
ARRDC5	GSTP1	ARRDC3
ARSA	GSTT1	ARRDC3-AS1
ARSB	GSTT2	ARRDC4
ARSD	GTF2B	ARRDC5
ARSG	GTF2H1	ARSA
ARSI	GULP1	ARSB
ARSJ	GYPA	ARSD
ARSK	GZMA	ARSF
ARSL	GZMB	ARSG
ART1	H2AW	ARSI
ART2A	H2AX	ARSJ
ART3	H2AZ1	ARSK
ART4	H3-4	ARSL
ART5	H3P10	ART1
ARTN	H3P12	ART2A
ARV1	H3P23	ART3
ARVCF	H3P28	ART4
ARX	H3P40	ART5
ARXES1	H3P41	ARTN
ARXES2	H3P9	ARV1
AS3MT	HABP2	ARVCF
ASAH1	HAGH	ARX

ASAH2	HAGLR	ARXES1
ASAH2B	HAND2-AS1	ARXES2
ASAP1	HAS2	AS3MT
ASAP1-IT1	HAS3	ASAH1
ASAP1-IT2	HAVCR1	ASAH2
ASAP2	HAVCR2	ASAH2B
ASAP3	HBE1	ASAP1
ASB1	HBEGF	ASAP1-IT1
ASB10	HBP1	ASAP1-IT2
ASB11	HDAC1	ASAP2
ASB12	HDAC2	ASAP3
ASB13	HDAC3	ASB1
ASB13A.2	HDAC4	ASB10
ASB14	HDAC5	ASB11
ASB15	HDAC6	ASB12
ASB15-AS1	HDAC7	ASB13
ASB16	HDAC9	ASB13A.2
ASB16-AS1	HDGF	ASB14
ASB18	HEATR1	ASB15
ASB2	HEATR6	ASB15-AS1
ASB3	HEIH	ASB16
ASB4	HELLS	ASB16-AS1
ASB5	HEPACAM	ASB18
ASB6	HERC2	ASB2
ASB7	HERC4	ASB3
ASB8	HERC5	ASB4
ASB9	HES1	ASB5
ASCC2	HES3	ASB6
ASCC3	HES5	ASB7
ASCL1	HEY2	ASB8
ASCL1A	HEYL	ASB9
ASCL1B	HGF	ASCC1
ASCL2	HHIP	ASCC2
ASCL3	HHLA2	ASCC3
ASCL5	HIC1	ASCL1
ASF1A	HIF1A	ASCL1A

Table S3

ASF1B	HIF1A-AS1	ASCL1B
ASGR1	HIF3A	ASCL2
ASGR2	HINT1	ASCL3
ASH1L	HINT2	ASCL5
ASH1L-AS1	HIP1	ASF1A
ASH2L	HIPK2	ASF1B
ASIC1	HIPK3	ASGR1
ASIC2	HIVEP1	ASGR2
ASIC3	HJURP	ASH1L
ASIC4	HK1	ASH1L-AS1
ASIC4B	HK2	ASH2L
ASL	HLA-A	ASIC1
ASL1	HLA-C	ASIC2
ASMT	HLA-DMB	ASIC3
ASMTL	HLA-DOA	ASIC4
ASMTL-AS1	HLA-DRA	ASIC4B
ASNA-1	HLA-DRB1	ASIP
ASNS	HLA-G	ASL
ASNSD1	HLTF	ASL1
ASP-14	HMGA1	ASMT
ASPA	HMGA2	ASMTL
ASPDH	HMGB1	ASMTL-AS1
ASPG	HMGB2	ASNA-1
ASPH	HMGB3	ASNS
ASPHD1	HMGB4	ASNSD1
ASPHD2	HMGN5	ASP-14
ASPM	HMMR	ASPA
ASPN	HMOX1	ASPDH
ASPRV1	HNF1A	ASPG
ASPSCR1	HNF1A-AS1	ASPH
ASRGL1	HNF4A	ASPHD1
ASS1	HNRNPA1	ASPHD2
ASTE1	HNRNPA1P10	ASPM
ASTL	HNRNPA2B1	ASPN
ASTL3A.1.L	HNRNPAB	ASPRV1
ASTN1	HNRNPC	ASPSCR1

ASTN2	HNRNPD	ASRGL1
ASUN	HNRNPDL	ASS1
ASXL1	HNRNPK	ASTE1
ASXL2	HOOK1	ASTL
ASXL3	HOPX	ASTL3A.1.L
ASZ1	HORMAD1	ASTN1
ATAD1	HORMAD2	ASTN2
ATAD2	HOTAIR	ASUN
ATAD2B	HOTTIP	ASXL1
ATAD3A	HOXA@	ASXL2
ATAD3B	HOXA1	ASXL3
ATAD3C	HOXA10	ASZ1
ATAD5	HOXA11	ATAD1
ATAD5A	HOXA11-AS	ATAD2
ATAT1	HOXA13	ATAD2B
ATCAY	HOXA3	ATAD3A
ATE1	HOXA4	ATAD3B
ATE1-AS1	HOXA5	ATAD3C
ATF1	HOXA9	ATAD5
ATF2	HOXA-AS2	ATAD5A
ATF3	HOXA-AS3	ATAT1
ATF4	HOXB2	ATCAY
ATF4A	HOXB5	ATD
ATF5	HOXB7	ATE1
ATF5.2.S	HOXB9	ATE1-AS1
ATF5B	HOXC11	ATF1
ATF6	HOXC6	ATF2
ATF6B	HOXC8	ATF3
ATF7	HOXD10	ATF4
ATF7IP	HOXD13	ATF4A
ATF7IP2	HOXD8	ATF5
ATG10	HP	ATF5.2.S
ATG101	HPC4	ATF5B
ATG12	HPD	ATF6
ATG13	HPGD	ATF6B
ATG14	HPGDS	ATF7

ATG16	HPP1	ATF7IP
ATG16L1	HPR	ATF7IP2
ATG16L2	HPRT1	ATG10
ATG2A	HPS5	ATG101
ATG2B	HPSE	ATG12
ATG3	HRAS	ATG13
ATG4A	HRG	ATG14
ATG4B	HRH4	ATG16
ATG4C	HS3ST2	ATG16L1
ATG4D	HS3ST3B1	ATG16L2
ATG5	HSD11B2	ATG2A
ATG7	HSD17B1	ATG2B
ATG9A	HSD17B6	ATG3
ATG9B	HSP90AA1	ATG4A
ATIC	HSP90AB1	ATG4B
ATL1	HSP90B1	ATG4C
ATL2	HSPA12B	ATG4D
ATL3	HSPA14	ATG5
ATM	HSPA1A	ATG7
ATMIN	HSPA1B	ATG9A
ATN1	HSPA2	ATG9B
ATOH1	HSPA4	ATIC
ATOH7	HSPA5	ATL1
ATOH8	HSPA9	ATL2
ATOX1	HSPB1	ATL3
ATP10A	HSPB2	ATM
ATP10B	HSPB3	ATMIN
ATP10D	HSPD1	ATN1
ATP11A	HTATIP2	ATOH1
ATP11AUN	HTC2	ATOH7
ATP11B	HTRA2	ATOH8
ATP11C	HTRA3	ATOX1
ATP12A	HULC	ATP10A
ATP13A1	HUWE1	ATP10B
ATP13A2	HYAL1	ATP10D
ATP13A3	IARS1	ATP11A

ATP13A3-DT	IARS2	ATP11AUN
ATP13A4	IATPR	ATP11B
ATP13A5	ICAM1	ATP11C
ATP1A1	ICOS	ATP12A
ATP1A1A.2	ID1	ATP13A1
ATP1A1A.3	IDH1	ATP13A2
ATP1A1A.4	IDH2	ATP13A3
ATP1A1-AS1	IDO1	ATP13A3-DT
ATP1A1OS	IFI27	ATP13A4
ATP1A2	IFI44	ATP13A5
ATP1A2A	IFIT2	ATP1A1
ATP1A3	IFITM1	ATP1A1A.2
ATP1A3A	IFN1@	ATP1A1A.3
ATP1A4	IFNA1	ATP1A1A.4
ATP1B1	IFNA13	ATP1A1-AS1
ATP1B1B	IFNA17	ATP1A1OS
ATP1B2	IFNA2	ATP1A2
ATP1B2A	IFNB1	ATP1A2A
ATP1B3	IFNG	ATP1A3
ATP1B3A	IFNGR1	ATP1A3A
ATP1B4	IFNL1	ATP1A4
ATP23	IFRD1	ATP1B1
ATP2A1	IGF1	ATP1B1B
ATP2A1L	IGF1R	ATP1B2
ATP2A2	IGF2	ATP1B2A
ATP2A2A	IGF2-AS	ATP1B3
ATP2A2B	IGF2BP1	ATP1B3A
ATP2A3	IGF2BP2	ATP1B4
ATP2B1	IGF2BP3	ATP23
ATP2B1-AS1	IGF2R	ATP2A1
ATP2B2	IGFBP1	ATP2A1L
ATP2B3	IGFBP2	ATP2A2
ATP2B4	IGFBP3	ATP2A2A
ATP2C1	IGFBP4	ATP2A2B
ATP2C2	IGFBP6	ATP2A3
ATP2C2-AS1	IGFBP7	ATP2B1

ATP4A	IGKC	ATP2B1-AS1
ATP4B	IKBKB	ATP2B2
ATP5A1	IKBKE	ATP2B3
ATP5B	IL10	ATP2B4
ATP5C1	IL11	ATP2C1
ATP5D	IL12A	ATP2C2
ATP5E	IL12A-AS1	ATP2C2-AS1
ATP5F1A	IL12B	ATP4A
ATP5F1B	IL12RB2	ATP4B
ATP5F1C	IL13	ATP5A1
ATP5F1D	IL15	ATP5B
ATP5F1E	IL16	ATP5C1
ATP5G1	IL17A	ATP5D
ATP5G2	IL17B	ATP5E
ATP5G3	IL17D	ATP5F1A
ATP5I	IL17F	ATP5F1B
ATP5IF1	IL17RA	ATP5F1C
ATP5J	IL17RB	ATP5F1D
ATP5J2	IL17RC	ATP5F1E
ATP5K	IL18	ATP5G1
ATP5L	IL18R1	ATP5G2
ATP5MC1	IL1A	ATP5G3
ATP5MC2	IL1B	ATP5I
ATP5MC3	IL1F10	ATP5IF1
ATP5MD	IL1RN	ATP5J
ATP5ME	IL2	ATP5J2
ATP5MF	IL20	ATP5K
ATP5MFP3	IL20RA	ATP5L
ATP5MF-PTCD1	IL20RB	ATP5MC1
ATP5MG	IL21	ATP5MC2
ATP5MGL	IL21R	ATP5MC3
ATP5MJ	IL22	ATP5MD
ATP5MK	IL23A	ATP5ME
ATP5O	IL23R	ATP5MF
ATP5PB	IL24	ATP5MFP3
ATP5PD	IL25	ATP5MF-PTCD1

ATP5PF	IL27	ATP5MG
ATP5PO	IL2RA	ATP5MGL
ATP6	IL31	ATP5MJ
ATP6AP1	IL33	ATP5MK
ATP6AP1-DT	IL37	ATP5O
ATP6AP1L	IL4	ATP5PB
ATP6AP2	IL6	ATP5PD
ATP6V0A1	IL6R	ATP5PF
ATP6V0A2	IL6ST	ATP5PO
ATP6V0A4	IL7	ATP6
ATP6V0B	IL7R	ATP6AP1
ATP6V0C	IL9	ATP6AP1-DT
ATP6V0CP1	ILF2	ATP6AP1L
ATP6V0D1	ILK	ATP6AP2
ATP6V0D1-DT	ILRUN	ATP6V0A1
ATP6V0D2	IMP3	ATP6V0A2
ATP6V0E1	IMPACT	ATP6V0A4
ATP6V0E2	ING1	ATP6V0B
ATP6V0E2-AS1	ING2	ATP6V0C
ATP6V1A	ING4	ATP6V0CP1
ATP6V1AB	INHBA	ATP6V0D1
ATP6V1B1	INHBC	ATP6V0D1-DT
ATP6V1B2	INO80	ATP6V0D2
ATP6V1BA	INO80D	ATP6V0E1
ATP6V1C1	INPP4A	ATP6V0E2
ATP6V1C1B	INPP5D	ATP6V0E2-AS1
ATP6V1C2	INPPL1	ATP6V1A
ATP6V1D	INS-IGF2	ATP6V1AB
ATP6V1E1	INSL4	ATP6V1B1
ATP6V1E2	INSM1	ATP6V1B2
ATP6V1F	INSR	ATP6V1BA
ATP6V1G1	INTS6	ATP6V1C1
ATP6V1G2	INVS	ATP6V1C1B
ATP6V1H	IPO8	ATP6V1C2
ATP7A	IQGAP1	ATP6V1D
ATP7B	IRAIN	ATP6V1E1

ATP8	IRAK2	ATP6V1E2
ATP8A1	IREB2	ATP6V1F
ATP8A2	IRF1	ATP6V1G1
ATP8B1	IRF3	ATP6V1G2
ATP8B2	IRF4	ATP6V1H
ATP8B3	IRF5	ATP7A
ATP8B4	IRF7	ATP7B
ATP8B5P	IRF8	ATP8
ATP9A	IRS1	ATP8A1
ATP9B	IRS2	ATP8A2
ATPAF1	IRX5	ATP8B1
ATPAF2	ISG15	ATP8B2
ATPIF1	ISG20	ATP8B3
ATPSCKMT	ITCH	ATP8B4
ATR	ITGA1	ATP8B5P
ATRAID	ITGA11	ATP9A
ATRIP	ITGA2	ATP9B
ATRN	ITGA2B	ATPAF1
ATRNL1	ITGA5	ATPAF2
ATRX	ITGA7	ATPIF1
ATTB	ITGA9	ATPSCKMT
ATXN1	ITGAE	ATR
ATXN10	ITGAM	ATRAID
ATXN1L	ITGAV	ATRIP
ATXN2	ITGB1	ATRN
ATXN2L	ITGB2	ATRNL1
ATXN3	ITGB3	ATRX
ATXN3L	ITGB4	ATTB
ATXN7	ITGBL1	ATXN1
ATXN7L1	ITIH5	ATXN10
ATXN7L2	ITPR3	ATXN1L
ATXN7L2A	ITPRID2	ATXN2
ATXN7L3	JAG1	ATXN2L
ATXN7L3B	JAK1	ATXN3
AUH	JAK2	ATXN3L
AUNIP	JAK3	ATXN7

AUP1	JAM2	ATXN7L1
AURKA	JAM3	ATXN7L2
AURKAIP1	JMJD6	ATXN7L2A
AURKB	JPT2	ATXN7L3
AURKC	JPX	ATXN7L3B
AUTS2	JUN	AUH
AVEN	JUNB	AUNIP
AVIL	JUND	AUP1
AVL9	KARS1	AURKA
AVP	KAT2A	AURKAIP1
AVPI1	KAT5	AURKB
AVPR1A	KAT8	AURKC
AVPR1B	KCNA5	AUTS2
AVPR2	KCNH4	AVEN
AXDND1	KCNH8	AVIL
AXIN1	KCNJ3	AVL9
AXIN2	KCNK3	AVP
AXL	KCNN4	AVPI1
AXUD1	KCNQ1	AVPR1A
AZGP1	KCNQ1OT1	AVPR1B
AZI2	KCTD20	AVPR2
AZIN1	KDM1A	AXDND1
AZIN2	KDM2A	AXIN1
AZU1	KDM4A	AXIN2
B0334.6	KDM4B	AXL
B2M	KDM5A	AXUD1
B3GALNT1	KDM5B	AZGP1
B3GALNT2	KDM6B	AZI2
B3GALT1	KDM7A-DT	AZIN1
B3GALT2	KDR	AZIN2
B3GALT4	KEAP1	AZU1
B3GALT5	KHDRBS1	B0334.6
B3GALT5-AS1	KHK	B2M
B3GALT6	KHSRP	B3GALNT1
B3GAT1	KIAA1217	B3GALNT2
B3GAT2	KIAA1522	B3GALT1

B3GAT3	KIDINS220	B3GALT2
B3GLCT	KIF11	B3GALT4
B3GNT2	KIF14	B3GALT5
B3GNT3	KIF16B	B3GALT5-AS1
B3GNT4	KIF20A	B3GALT6
B3GNT5	KIF22	B3GAT1
B3GNT6	KIF2C	B3GAT2
B3GNT7	KIF3A	B3GAT3
B3GNT8	KIF4A	B3GLCT
B3GNT9	KIF5B	B3GNT2
B3GNTL1	KIFC1	B3GNT3
B4GALNT1	KIN	B3GNT4
B4GALNT2	KIR2DL1	B3GNT5
B4GALNT3	KIR2DL2	B3GNT6
B4GALNT4	KIR2DL4	B3GNT7
B4GALT1	KIR2DS1	B3GNT8
B4GALT2	KIR3DL1	B3GNT9
B4GALT3	KIR3DL2	B3GNTL1
B4GALT4	KISS1	B4GALNT1
B4GALT5	KISS1R	B4GALNT2
B4GALT6	KIT	B4GALNT3
B4GALT7	KITLG	B4GALNT4
B4GAT1	KL	B4GALT1
B9D1	KLB	B4GALT2
B9D2	KLC2	B4GALT3
BAALC	KLC3	B4GALT4
BAALC-AS2	KLF17	B4GALT5
BAAT	KLF2	B4GALT6
BABAM1	KLF4	B4GALT7
BABAM2	KLF5	B4GAT1
BACE1	KLF6	B9D1
BACE2	KLF7	B9D2
BACH1	KLF8	BAALC
BACH1A	KLF9	BAALC-AS2
BACH2	KLHL1	BAAT
BAD	KLK10	BABAM1

BAG1	KLK11	BABAM2
BAG2	KLK13	BABAM2-AS1
BAG3	KLK14	BACE1
BAG4	KLK5	BACE2
BAG5	KLK8	BACH1
BAG6	KLLN	BACH1A
BAGE	KLRB1	BACH2
BAGE2	KLRC4-KLRK1	BAD
BAGE3	KLRD1	BAG1
BAGE4	KLRK1	BAG2
BAGE5	KMT2A	BAG3
BAHCC1	KMT2B	BAG4
BAHD1	KMT2D	BAG5
BAIAP2	KMT5A	BAG6
BAIAP2-DT	KNG1	BAGE
BAIAP2L1	KPNA2	BAGE2
BAIAP2L2	KRAS	BAGE3
BAIAP3	KRT14	BAGE4
BAK1	KRT16	BAGE5
BAMBI	KRT17	BAHCC1
BANCR	KRT18	BAHD1
BANF1	KRT19	BAIAP2
BANF2	KRT20	BAIAP2-DT
BANK1	KRT5	BAIAP2L1
BANP	KRT6B	BAIAP2L2
BAP1	KRT7	BAIAP3
BARD1	KRT8	BAK1
BARHL1	KRT81	BAMBI
BARHL2	KRT8P3	BANCR
BARX1	L1CAM	BANF1
BARX2	L1TD1	BANF2
BASP1	LAG3	BANK1
BATF	LAMB3	BANP
BATF2	LAMC2	BAP1
BATF3	LAMP1	BARD1
BATH-36	LAMTOR1	BARHL1

BAX	LAMTOR5	BARHL2
BAXA	LANCL1	BARX1
BAXB	LAPTM4B	BARX2
BAZ1A	LARP1	BASP1
BAZ1B	LARS1	BATF
BAZ2A	LARS2	BATF2
BAZ2B	LASP1	BATF3
BBC3	LAT	BATH-36
BBIP1	LATS1	BAX
BBLN	LATS2	BAXA
BBOF1	LBX2-AS1	BAXB
BBOX1	LCK	BAZ1A
BBS1	LDHA	BAZ1B
BBS10	LDHB	BAZ2A
BBS12	LDHC	BAZ2B
BBS2	LDOC1	BBC3
BBS4	LECT2	BBIP1
BBS5	LEF1	BBLN
BBS7	LEMD3	BBOF1
BBS9	LEP	BBOX1
BBX	LEPQTL1	BBS1
BCAM	LEPR	BBS10
BCAN	LETM1	BBS12
BCAP29	LGALS1	BBS2
BCAP31	LGALS3	BBS4
BCAR1	LGALS9	BBS5
BCAR3	LGI1	BBS7
BCAS1	LGI3	BBS9
BCAS2	LGR4	BBX
BCAS3	LGR5	BCAM
BCAS4	LGR6	BCAN
BCAT1	LHX2	BCAP29
BCAT2	LHX3	BCAP31
BCCIP	LHX6	BCAR1
BCDIN3D	LIG1	BCAR3
BCHE	LIG4	BCAR4

BCKDHA	LILRB1	BCAS1
BCKDHB	LILRB2	BCAS2
BCKDK	LIMCH1	BCAS3
BCL10	LIMD2	BCAS4
BCL11A	LIME1	BCAT1
BCL11B	LIMK1	BCAT2
BCL2	LIMK2	BCCIP
BCL2A	LIN28A	BCDIN3D
BCL2A1	LIN28B	BCHE
BCL2A1A	LINC00173	BCKDHA
BCL2A1B	LINC00221	BCKDHB
BCL2A1C	LINC00243	BCKDK
BCL2A1D	LINC00261	BCL10
BCL2B	LINC00312	BCL11A
BCL2L1	LINC00319	BCL11B
BCL2L10	LINC00328	BCL2
BCL2L11	LINC00337	BCL2A
BCL2L12	LINC00339	BCL2A1
BCL2L13	LINC00342	BCL2A1A
BCL2L14	LINC00346	BCL2A1B
BCL2L15	LINC00426	BCL2A1C
BCL2L2	LINC00460	BCL2A1D
BCL3	LINC00472	BCL2B
BCL6	LINC00473	BCL2L1
BCL6B	LINC00511	BCL2L10
BCL7A	LINC00589	BCL2L11
BCL7B	LINC00630	BCL2L12
BCL7C	LINC00641	BCL2L13
BCL9	LINC00665	BCL2L14
BCL9L	LINC00668	BCL2L15
BCLAF1	LINC00673	BCL2L2
BCLAF3	LINC00702	BCL3
BCLX	LINC00858	BCL6
BCO1	LINC00958	BCL6B
BCO2	LINC00963	BCL7A
BCO2A	LINC00968	BCL7B

BCOR	LINC01088	BCL7C
BCORL1	LINC01116	BCL9
BCR	LINC01123	BCL9L
BCRP5	LINC01133	BCLAF1
BCRP7	LINC01193	BCLAF3
BCS1L	LINC01194	BCLX
BCYRN1	LINC01234	BCO1
BDH1	LINC01288	BCO2
BDH2	LINC01354	BCO2A
BDKRB1	LINC01433	BCOR
BDKRB2	LINC01436	BCORL1
BDNF	LINC01614	BCR
BDNF-AS	LINC01638	BCRP3
BDP1	LINC01672	BCRP5
BEAN1	LINC01852	BCRP7
BECN1	LINC02418	BCS1L
BEGAIN	LINC02602	BCYRN1
BEND3	LINC02605	BDH1
BEND3P3	LINC-PINT	BDH2
BEND4	LINC-ROR	BDKRB1
BEND5	LIPC	BDKRB2
BEND6	LLGL1	BDNF
BEND7	LMLN	BDNF-AS
BEST1	LMNB2	BDP1
BEST3	LMO1	BEAN1
BEST4	LMO7	BECN1
BET1	LNCNEF	BEGAIN
BET1L	LNCRNA-ATB	BEND3
BETAGGT-I	LOC110806263	BEND3P3
BEX1	LOC730101	BEND4
BEX2	LONP1	BEND5
BEX3	LOX	BEND6
BEX4	LOXL1	BEND7
BEX5	LOXL1-AS1	BEST1
BFAR	LOXL2	BEST3
BFSP1	LPAR2	BEST4

BFSP2	LPAR3	BET1
BFSP2-AS1	LPCAT1	BET1L
BGLAP	LPIN3	BETAGGT-I
BGN	LPL	BEX1
BHLHA15	LRG1	BEX2
BHLHA9	LRIG1	BEX3
BHLHB9	LRIG2	BEX4
BHLHE22	LRP1	BEX5
BHLHE23	LRP12	BFAR
BHLHE40	LRP1B	BFSP1
BHLHE41	LRP5	BFSP2
BHMT	LRP6	BFSP2-AS1
BHMT2	LRPPRC	BGLAP
BICC1	LRRC59	BGN
BICD1	LSINCT5	BHLHA15
BICD2	LSM2	BHLHA9
BICDL1	LTA	BHLHB9
BICDL2	LTBR	BHLHE22
BICRA	LTF	BHLHE23
BICRAL	LTO1	BHLHE40
BID	LUCAT1	BHLHE41
BIDA	LY6K	BHMT
BIK	LYN	BHMT2
BIN1	LYPD3	BICC1
BIN2	LYPD5	BICD1
BIN3	LYPLA1	BICD2
BIN3-IT1	LYVE1	BICDL1
BIRC2	LZTS2	BICDL2
BIRC3	LZTS3	BICRA
BIRC5	MACC1	BICRAL
BIRC5.S	MACIR	BID
BIRC6	MAD2L1	BIDA
BIRC7	MAFG-DT	BIK
BIRC8	MAFK	BIN1
BIVM	MAGEA1	BIN2
BIVM-ERCC5	MAGEA10	BIN3

BLACAT1	MAGEA3	BIN3-IT1
BLCAP	MAGEA4	BIRC2
BLF	MAGEC1	BIRC3
BLK	MAGEC2	BIRC5
BLM	MAGED1	BIRC5.S
BLMH	MAGED4	BIRC6
BLNK	MAGED4B	BIRC7
BLOC1S1	MAGI2	BIRC8
BLOC1S2	MAGI2-AS3	BIVM
BLOC1S3	MAK16	BIVM-ERCC5
BLOC1S4	MALAT1	BLACAT1
BLOC1S5	MAOA	BLCAP
BLOC1S5-TXNDC5	MAOB	BLF
BLOC1S6	MAP1LC3A	BLID
BLTP1	MAP1LC3B	BLK
BLTP2	MAP2K1	BLM
BLTP3A	MAP2K2	BLMH
BLTP3B	MAP2K3	BLNK
BLVRA	MAP2K4	BLOC1S1
BLVRB	MAP2K7	BLOC1S2
BLZF1	MAP3K10	BLOC1S3
BMERB1	MAP3K11	BLOC1S4
BMF	MAP3K14	BLOC1S5
BMI1	MAP3K2	BLOC1S5-TXNDC5
BMI1A	MAP3K3	BLOC1S6
BMM	MAP3K7	BLTP1
BMP1	MAP3K8	BLTP2
BMP10	MAP4K1	BLTP3A
BMP15	MAP4K3	BLTP3B
BMP2	MAP4K4	BLVRA
BMP2K	MAPK1	BLVRB
BMP3	MAPK14	BLZF1
BMP4	MAPK3	BMERB1
BMP5	MAPK7	BMF
BMP6	MAPK8	BMI1
BMP7	MAPK8IP2	BMI1A

BMP8A	MAPK9	BMM
BMP8B	MAPKAP1	BMP1
BMPER	MAPKAPK2	BMP10
BMPR1A	MAPRE1	BMP15
BMPR1AP1	MAPT	BMP2
BMPR1B	MARCHF1	BMP2K
BMPR2	MARCHF3	BMP3
BMS1	MARCHF8	BMP4
BMS1P1	MARCKSL1	BMP5
BMS1P18	MARCO	BMP6
BMS1P2	MARK1	BMP7
BMS1P20	MARK2	BMP8A
BMS1P4	MARS1	BMP8B
BMT2	MARVELD1	BMPER
BMX	MASTL	BMPR1A
BMYC	MB	BMPR1AP1
BNC1	MBD2	BMPR1B
BNC2	MBD3	BMPR2
BNIP1	MBD4	BMS1
BNIP2	MBD5	BMS1P1
BNIP3	MBL2	BMS1P18
BNIP3L	MBNL1-AS1	BMS1P2
BNIP5	MCAM	BMS1P20
BNIPL	MCAT	BMS1P4
BNL	MCC	BMT2
BOC	MCCD1P1	BMX
BOD1	MCL1	BMYC
BOD1L	MCM2	BNC1
BOD1L1	MCM4	BNC2
BOK	MCM6	BNIP1
BOLA1	MCM7	BNIP2
BOLA2	MCOLN1	BNIP3
BOLA2B	MCPH1	BNIP3L
BOLA2-PS4	MCRS1	BNIP5
BOLA3	MCTS1	BNIPL
BOLA3-DT	MDFI	BNL

BOLL	MDH1	BOC
BOP1	MDH2	BOD1
BORA	MDK	BOD1L
BORCS5	MDM2	BOD1L1
BORCS6	MDM4	BOK
BORCS7	ME1	BOLA1
BORCS7-ASMT	ME2	BOLA2
BORCS8	MECOM	BOLA2B
BORCS8-MEF2B	MECP2	BOLA2-PS4
BPESC1	MED1	BOLA3
BPGM	MED12	BOLA3-DT
BPHL	MED19	BOLL
BPI	MED23	BOP1
BPIFA1	MED28	BORA
BPIFA2	MEF2D	BORCS5
BPIFA5	MEG3	BORCS6
BPIFB1	MEN1	BORCS7
BPIFB2	MEOX1	BORCS7-ASMT
BPIFB3	MEOX2	BORCS8
BPIFB4	MERTK	BORCS8-MEF2B
BPIFC	MESP1	BPESC1
BPNT1	MEST	BPGM
BPNT2	MET	BPHL
BPTF	METAP2	BPI
BPY2	METTL3	BPIFA1
BRAF	MFAP1	BPIFA2
BRAP	MFF	BPIFA5
BRAT1	MFN2	BPIFB1
BRCA1	MGA	BPIFB2
BRCA2	MGMT	BPIFB3
BRCC3	MIAT	BPIFB4
BRD1	MICA	BPIFC
BRD2	MICB	BPNT1
BRD3	MICOS10-NBL1	BPNT2
BRD3OS	MIF	BPTF
BRD4	MIF-AS1	BPY2

BRD7	MIIP	BRAF
BRD8	MINCR	BRAP
BRD9	MINDY3	BRAT1
BRDT	MIR100	BRCA1
BRE	MIR105-1	BRCA2
BRF1	MIR106A	BRCC3
BRF1A	MIR106B	BRD1
BRF2	MIR107	BRD2
BRI3	MIR10A	BRD3
BRI3BP	MIR10B	BRD3OS
BRICD5	MIR1179	BRD4
BRINP1	MIR1182	BRD7
BRINP2	MIR1183	BRD8
BRINP3	MIR1197	BRD9
BRIP1	MIR1-2	BRDT
BRIX1	MIR1204	BRE
BRME1	MIR122	BRF1
BRMS1	MIR1238	BRF1A
BRMS1L	MIR124-1	BRF2
BROX	MIR124-2	BRI3
BRPF1	MIR124-3	BRI3BP
BRPF3	MIR1244-1	BRICD5
BRS3	MIR1246	BRINP1
BRSK1	MIR1253	BRINP2
BRSK2	MIR1256	BRINP3
BRWD1	MIR1258	BRIP1
BRWD1-AS2	MIR125A	BRIX1
BRWD3	MIR125B1	BRK1
BSCL2	MIR125B2	BRME1
BSDC1	MIR126	BRMS1
BSG	MIR1260B	BRMS1L
BSN	MIR1271	BROX
BSN-DT	MIR1275	BRPF1
BSPRY	MIR128-2	BRPF3
BST1	MIR1287	BRS3
BST2	MIR1290	BRSK1

BSX	MIR129-1	BRSK2
BTAF1	MIR129-2	BRWD1
BTBD1	MIR1297	BRWD1-AS2
BTBD10	MIR1298	BRWD3
BTBD11	MIR1304	BSCL2
BTBD16	MIR1305	BSDC1
BTBD17	MIR130A	BSG
BTBD18	MIR130B	BSN
BTBD19	MIR132	BSN-DT
BTBD2	MIR133B	BSPRY
BTBD3	MIR134	BST1
BTBD35F20	MIR135B	BST2
BTBD6	MIR136	BSX
BTBD7	MIR137	BTAF1
BTBD8	MIR139	BTBD1
BTBD9	MIR140	BTBD10
BTC	MIR141	BTBD11
BTD	MIR142	BTBD16
BTF3	MIR143	BTBD17
BTF3L4	MIR144	BTBD18
BTF3P11	MIR145	BTBD19
BTG1	MIR146A	BTBD2
BTG2	MIR146B	BTBD3
BTG2-DT	MIR147A	BTBD35F20
BTG3	MIR147B	BTBD6
BTG4	MIR148A	BTBD7
BTG5.2.S	MIR148B	BTBD8
BTK	MIR149	BTBD9
BTLA	MIR150	BTC
BTN1A1	MIR152	BTD
BTN2A1	MIR154	BTF3
BTN2A2	MIR155	BTF3L4
BTN2A3P	MIR15A	BTF3P11
BTN3A1	MIR15B	BTG1
BTN3A2	MIR16-1	BTG2
BTN3A3	MIR17	BTG2-DT

BTNL1	MIR17HG	BTG3
BTNL10P	MIR181C	BTG4
BTNL3	MIR181D	BTG5.2.S
BTNL5-PS	MIR182	BTK
BTNL8	MIR183	BTLA
BTNL9	MIR184	BTN1A1
BTR29	MIR185	BTN2A1
BTRC	MIR186	BTN2A2
BUB1	MIR187	BTN2A3P
BUB1B	MIR188	BTN3A1
BUB3	MIR18A	BTN3A2
BUD13	MIR1908	BTN3A3
BUD23	MIR190B	BTNL1
BUD31	MIR191	BTNL10P
BVES	MIR1914	BTNL3
BXDC2	MIR192	BTNL5-PS
BYSL	MIR193A	BTNL8
BZW1	MIR193B	BTNL9
BZW1-AS1	MIR195	BTR29
BZW1B	MIR196A1	BTRC
BZW1P2	MIR196A2	BUB1
BZW2	MIR196B	BUB1B
C02D5.4	MIR197	BUB3
C04G2.3	MIR1976	BUD13
C10ORF105	MIR198	BUD23
C10ORF120	MIR199B	BUD31
C10ORF143	MIR19A	BVES
C10ORF55	MIR19B1	BXDC2
C10ORF67	MIR200A	BYSL
C10ORF71	MIR200B	BZW1
C10ORF82	MIR200C	BZW1-AS1
C10ORF88	MIR202	BZW1B
C10ORF95	MIR203A	BZW1P2
C10ORF95-AS1	MIR204	BZW2
C11ORF1	MIR205	C02D5.4
C11ORF16	MIR206	C04G2.3

C11ORF21	MIR208A	C10ORF105
C11ORF24	MIR20A	C10ORF120
C11ORF40	MIR20B	C10ORF143
C11ORF42	MIR21	C10ORF55
C11ORF52	MIR210	C10ORF67
C11ORF54	MIR210HG	C10ORF71
C11ORF58	MIR211	C10ORF82
C11ORF65	MIR212	C10ORF88
C11ORF68	MIR214	C10orf90
C11ORF71	MIR215	C10ORF95
C11ORF80	MIR216A	C10ORF95-AS1
C11ORF86	MIR216B	C11ORF1
C11ORF87	MIR217	C11ORF16
C11ORF91	MIR219A1	C11ORF21
C11ORF94	MIR22	C11ORF24
C11ORF96	MIR221	C11ORF40
C11ORF97	MIR222	C11ORF42
C12ORF29	MIR223	C11ORF52
C12ORF4	MIR224	C11ORF54
C12ORF50	MIR23A	C11ORF58
C12ORF54	MIR23B	C11ORF65
C12ORF56	MIR25	C11ORF68
C12ORF57	MIR26A1	C11ORF71
C12ORF60	MIR26B	C11ORF80
C12ORF73	MIR27A	C11ORF86
C12ORF75	MIR27B	C11ORF87
C12ORF76	MIR296	C11ORF91
C13A2.12	MIR299	C11ORF94
C14A6.8	MIR29A	C11ORF96
C14ORF119	MIR29B1	C11ORF97
C14ORF132	MIR29B2	C12ORF29
C14ORF156	MIR29C	C12ORF4
C14ORF180	MIR300	C12ORF50
C14ORF28	MIR301A	C12ORF54
C14ORF39	MIR301B	C12ORF56
C14ORF93	MIR302A	C12ORF57

C15ORF32	MIR302B	C12ORF60
C15ORF39	MIR30A	C12ORF73
C15ORF40	MIR30B	C12ORF75
C15ORF48	MIR30C1	C12ORF76
C15ORF61	MIR30C2	C13A2.12
C15ORF62	MIR30D	C14A6.8
C16C8.14	MIR30E	C14ORF119
C16C8.4	MIR31	C14ORF132
C16ORF46	MIR3120	C14ORF156
C16ORF54	MIR3127	C14ORF180
C16ORF72	MIR3163	C14ORF28
C16ORF74	MIR3188	C14ORF39
C16ORF82	MIR31HG	C14ORF93
C16ORF86	MIR32	C15ORF32
C16ORF87	MIR320A	C15ORF39
C16ORF89	MIR324	C15ORF40
C16ORF90	MIR325	C15ORF48
C16ORF91	MIR326	C15ORF61
C16ORF92	MIR328	C15ORF62
C16ORF95	MIR330	C16C8.14
C16ORF96	MIR331	C16C8.4
C17H12.6	MIR335	C16ORF46
C17H12.8	MIR337	C16ORF54
C17ORF100	MIR338	C16ORF72
C17ORF107	MIR339	C16ORF74
C17ORF113	MIR33A	C16ORF82
C17ORF49	MIR33B	C16ORF86
C17ORF50	MIR340	C16ORF87
C17ORF58	MIR342	C16ORF89
C17ORF64	MIR345	C16ORF90
C17ORF67	MIR346	C16ORF91
C17ORF75	MIR34A	C16ORF92
C17ORF80	MIR34B	C16ORF95
C17ORF97	MIR34C	C16ORF96
C17ORF98	MIR361	C17H12.6
C17ORF99	MIR3619	C17H12.8

C18H18ORF32	MIR362	C17ORF100
C18H9.6	MIR363	C17ORF107
C18ORF21	MIR365A	C17ORF113
C18ORF25	MIR3666	C17ORF49
C18ORF32	MIR367	C17ORF50
C18ORF54	MIR369	C17ORF58
C19ORF12	MIR370	C17ORF64
C19ORF18	MIR372	C17ORF67
C19ORF25	MIR373	C17ORF75
C19ORF33	MIR374A	C17ORF80
C19ORF38	MIR375	C17ORF97
C19ORF47	MIR376C	C17ORF98
C19ORF48	MIR377	C17ORF99
C19ORF53	MIR378A	C18H18ORF32
C19ORF54	MIR379	C18H9.6
C19ORF67	MIR381	C18ORF21
C19ORF73	MIR382	C18ORF25
C19ORF81	MIR383	C18ORF32
C1D	MIR384	C18ORF54
C1GALT1	MIR3940	C19ORF12
C1GALT1C1	MIR409	C19ORF18
C1GALT1C1L	MIR410	C19ORF25
C1H9ORF85	MIR411	C19ORF33
C1ORF100	MIR421	C19ORF38
C1ORF105	MIR422A	C19ORF47
C1ORF109	MIR424	C19ORF48
C1ORF112	MIR425	C19ORF53
C1ORF115	MIR4257	C19ORF54
C1ORF116	MIR4262	C19ORF67
C1ORF122	MIR4270	C19ORF73
C1ORF131	MIR4286	C19ORF81
C1ORF141	MIR429	C1D
C1ORF159	MIR4293	C1GALT1
C1ORF162	MIR4299	C1GALT1C1
C1ORF174	MIR4301	C1GALT1C1L
C1ORF198	MIR4317	C1H9ORF85

C1ORF21	MIR4319	C1ORF100
C1ORF210	MIR433	C1ORF105
C1ORF216	MIR4435-2HG	C1ORF109
C1ORF226	MIR4443	C1ORF112
C1ORF228	MIR4458	C1ORF115
C1ORF35	MIR4465	C1ORF116
C1ORF43	MIR448	C1ORF122
C1ORF50	MIR449A	C1ORF131
C1ORF52	MIR449B	C1ORF141
C1ORF53	MIR449C	C1ORF159
C1ORF54	MIR4500	C1ORF162
C1ORF56	MIR451A	C1ORF174
C1ORF68	MIR452	C1ORF198
C1ORF74	MIR454	C1ORF21
C1ORF87	MIR455	C1ORF210
C1ORF94	MIR4677	C1ORF216
C1QA	MIR4735	C1ORF226
C1QB	MIR4782	C1ORF228
C1QBP	MIR483	C1ORF35
C1QC	MIR484	C1ORF43
C1QL1	MIR485	C1ORF50
C1QL2	MIR486-1	C1ORF52
C1QL3	MIR488	C1ORF53
C1QL3A	MIR489	C1ORF54
C1QL3B	MIR491	C1ORF56
C1QL4	MIR493	C1ORF68
C1QTNF1	MIR494	C1ORF74
C1QTNF12	MIR495	C1ORF87
C1QTNF1-AS1	MIR496	C1ORF94
C1QTNF2	MIR497	C1QA
C1QTNF3	MIR498	C1QB
C1QTNF4	MIR499A	C1QBP
C1QTNF5	MIR500A	C1QC
C1QTNF6	MIR501	C1QL1
C1QTNF7	MIR502	C1QL2
C1QTNF8	MIR503	C1QL3

C1QTNF9	MIR504	C1QL3A
C1R	MIR505	C1QL3B
C1RL	MIR506	C1QL4
C1RL-AS1	MIR507	C1QTNF1
C1S	MIR509-1	C1QTNF12
C2	MIR510	C1QTNF1-AS1
C20ORF141	MIR5100	C1QTNF2
C20ORF144	MIR5195	C1QTNF3
C20ORF173	MIR519D	C1QTNF4
C20ORF181	MIR520A	C1QTNF5
C20ORF203	MIR520E	C1QTNF6
C20ORF204	MIR520F	C1QTNF7
C20ORF27	MIR522	C1QTNF8
C20ORF85	MIR526B	C1QTNF9
C20ORF96	MIR527	C1R
C21ORF58	MIR539	C1RL
C21ORF62	MIR541	C1RL-AS1
C21ORF62-AS1	MIR542	C1S
C21ORF91	MIR543	C2
C22ORF15	MIR545	C20ORF141
C22ORF23	MIR548L	C20ORF144
C22ORF39	MIR561	C20ORF173
C22ORF42	MIR5702	C20ORF181
C22ORF46	MIR574	C20ORF203
C24H12.2	MIR575	C20ORF204
C2CD2	MIR576	C20ORF27
C2CD2L	MIR577	C20ORF85
C2CD3	MIR582	C20ORF96
C2CD4A	MIR584	C21ORF58
C2CD4B	MIR585	C21ORF62
C2CD4C	MIR589	C21ORF62-AS1
C2CD5	MIR590	C21ORF91
C2ORF15	MIR592	C22ORF15
C2ORF16	MIR593	C22ORF23
C2ORF27A	MIR598	C22ORF39
C2ORF42	MIR599	C22ORF42

C2ORF49	MIR600	C22ORF46
C2ORF50	MIR605	C24H12.2
C2ORF66	MIR608	C2CD2
C2ORF68	MIR612	C2CD2L
C2ORF69	MIR613	C2CD3
C2ORF72	MIR615	C2CD4A
C2ORF73	MIR616	C2CD4B
C2ORF74	MIR621	C2CD4C
C2ORF76	MIR625	C2CD5
C2ORF80	MIR628	C2ORF15
C2ORF81	MIR629	C2ORF16
C2ORF88	MIR630	C2ORF27A
C2ORF92	MIR635	C2ORF42
C3	MIR638	C2ORF49
C32H11.3	MIR641	C2ORF50
C32H11.4	MIR642B	C2ORF66
C32H11.9	MIR647	C2ORF68
C35B1.5	MIR650	C2ORF69
C39F7.5	MIR6507	C2ORF72
C3AR1	MIR652	C2ORF73
C3ORF14	MIR653	C2ORF74
C3ORF18	MIR655	C2ORF76
C3ORF20	MIR660	C2ORF80
C3ORF22	MIR661	C2ORF81
C3ORF33	MIR663A	C2ORF88
C3ORF36	MIR665	C2ORF92
C3ORF38	MIR671	C3
C3ORF49	MIR675	C32H11.3
C3ORF52	MIR6754	C32H11.4
C3ORF56	MIR708	C32H11.9
C3ORF62	MIR7-1	C35B1.5
C3ORF70	MIR718	C39F7.5
C3ORF80	MIR7-2	C3AR1
C3P1	MIR7-3	C3ORF14
C43D7.7	MIR744	C3ORF18
C47E8.3	MIR758	C3ORF20

C49F5.6	MIR760	C3ORF22
C49G7.12	MIR761	C3ORF33
C4A	MIR762	C3ORF36
C4B	MIR769	C3ORF38
C4BP	MIR770	C3ORF49
C4BPA	MIR802	C3ORF52
C4BPB	MIR873	C3ORF56
C4ORF17	MIR874	C3ORF62
C4ORF19	MIR875	C3ORF70
C4ORF3	MIR877	C3ORF80
C4ORF33	MIR889	C3P1
C4ORF36	MIR92B	C43D7.7
C4ORF46	MIR93	C47E8.3
C4ORF47	MIR9-3	C49F5.6
C4ORF48	MIR935	C49G7.12
C4ORF50	MIR936	C4A
C4ORF54	MIR939	C4B
C5	MIR940	C4BP
C5AR1	MIR942	C4BPA
C5AR2	MIR944	C4BPB
C5ORF15	MIR95	C4ORF17
C5ORF22	MIR96	C4ORF19
C5ORF24	MIR98	C4ORF3
C5ORF34	MIR99A	C4ORF33
C5ORF46	MIR99B	C4ORF36
C5ORF47	MIRLET7A2	C4ORF46
C5ORF49	MIRLET7B	C4ORF47
C5ORF58	MIRLET7C	C4ORF48
C5ORF63	MIRLET7E	C4ORF50
C5ORF64	MIRLET7G	C4ORF54
C6	MKI67	C5
C6ORF118	MKNK2	C5AR1
C6ORF120	MKS1	C5AR2
C6ORF132	MLC1	C5ORF15
C6ORF136	MLH1	C5ORF22
C6ORF141	MLKL	C5ORF24

C6ORF15	MLRL	C5ORF34
C6ORF163	MLXIP	C5ORF46
C6ORF201	MMD	C5ORF47
C6ORF47	MME	C5ORF49
C6ORF52	MMP1	C5ORF58
C6ORF58	MMP10	C5ORF63
C6ORF62	MMP11	C5ORF64
C6ORF89	MMP12	C6
C7	MMP13	C6ORF118
C77080	MMP14	C6ORF120
C78859	MMP15	C6ORF132
C79685	MMP16	C6ORF136
C7ORF25	MMP19	C6ORF141
C7ORF31	MMP2	C6ORF15
C7ORF49	MMP26	C6ORF163
C7ORF50	MMP3	C6ORF201
C7ORF57	MMP7	C6ORF47
C80142	MMP9	C6ORF52
C8A	MMRN1	C6ORF58
C8B	MMS19	C6ORF62
C8G	MMUT	C6ORF89
C8ORF33	MNS16A	C7
C8ORF34	MNX1-AS1	C77080
C8ORF34-AS1	MOB1A	C78859
C8ORF44	MOB4	C79685
C8ORF48	MOK	C7ORF25
C8ORF58	MORC2	C7ORF31
C8ORF74	MOS	C7ORF49
C8ORF76	MOV10L1	C7ORF50
C8ORF82	MPG	C7ORF57
C8ORF88	MPHOSPH8	C80142
C9	MPO	C8A
C9ORF131	MPRIP	C8B
C9ORF152	MPST	C8G
C9ORF163	MPZL3	C8ORF33
C9ORF24	MRC1	C8ORF34

C9ORF40	MRGPRD	C8ORF34-AS1
C9ORF43	MRGPRX1	C8ORF44
C9ORF50	MRGPRX3	C8ORF48
C9ORF64	MRGPRX4	C8ORF58
C9ORF72	MRM2	C8ORF74
C9ORF78	MRPL28	C8ORF76
C9ORF85	MRPL41	C8ORF82
CA1	MRPL58	C8ORF88
CA10	MS4A1	C9
CA10A	MSH2	C9ORF131
CA11	MSH3	C9ORF152
CA12	MSI1	C9ORF163
CA13	MSLN	C9ORF24
CA14	MSMB	C9ORF40
CA2	MSR1	C9ORF43
CA3	MSRA	C9ORF50
CA4	MST1	C9ORF64
CA4A	MST1R	C9ORF72
CA5A	MSTO1	C9ORF78
CA5B	MT1A	C9ORF85
CA5BP1	MT1B	CA1
CA7	MT1E	CA10
CA8	MT1F	CA10A
CA9	MT1G	CA11
CAAP1	MT1H	CA12
CAB39	MT1IP	CA13
CAB39L	MT1JP	CA14
CAB39L1	MT1L	CA2
CABCOCO1	MT1M	CA3
CABIN1	MT1X	CA4
CABLES1	MT3	CA4A
CABLES2	MT4	CA5A
CABP1	MTA1	CA5B
CABP2	MTA2	CA5BP1
CABP4	MTA3	CA7
CABP7	MTAP	CA8

CABS1	MTBP	CA9
CABYR	MTCO2P12	CAAP1
CACFD1	MTDH	CAB39
CACHD1	MTERF1	CAB39L
CACNA1A	MTERF2	CAB39L1
CACNA1B	MTFMT	CABCOCO1
CACNA1C	MTHFD1	CABIN1
CACNA1D	MTHFD2	CABLES1
CACNA1E	MTHFR	CABLES2
CACNA1F	MTHFS	CABP1
CACNA1G	MTNR1A	CABP2
CACNA1H	MTOR	CABP4
CACNA1I	MTR	CABP7
CACNA1S	MTRR	CABS1
CACNA2D1	MTSS1	CABYR
CACNA2D2	MTUS1	CACFD1
CACNA2D3	MUC1	CACHD1
CACNA2D4	MUC16	CACNA1A
CACNB1	MUC2	CACNA1B
CACNB2	MUC4	CACNA1C
CACNB3	MUC5AC	CACNA1D
CACNB4	MUC5B	CACNA1E
CACNG1	MUL1	CACNA1F
CACNG2	MUS81	CACNA1G
CACNG3	MUTYH	CACNA1G-AS1
CACNG4	MVP	CACNA1H
CACNG5	MXI1	CACNA1I
CACNG6	MXRA5	CACNA1S
CACNG7	MYB	CACNA2D1
CACNG8	MYBBP1A	CACNA2D2
CACTIN	MYBL2	CACNA2D3
CACUL1	MYC	CACNA2D4
CACYBP	MYCBP	CACNB1
CAD	MYCL	CACNB2
CADM1	MYCN	CACNB3
CADM2	MYD88	CACNB4

CADM3	MYDGF	CACNG1
CADM4	MYEOV	CACNG2
CADPS	MYH11	CACNG3
CADPS2	MYH9	CACNG4
CAGE1	MYL9	CACNG5
CAHM	MYLIP	CACNG6
CALB1	MYLK	CACNG7
CALB2	MYO10	CACNG8
CALCA	MYO6	CACTIN
CALCB	MYOZ2	CACUL1
CALCOCO1	MZB1	CACYBP
CALCOCO2	NAA25	CAD
CALCR	NAA50	CADM1
CALCRL	NAE1	CADM2
CALD1	NAIF1	CADM3
CALHM1	NAMPT	CADM4
CALHM2	NANOG	CADPS
CALHM3	NANOS3	CADPS2
CALHM4	NAP1L1	CAGE1
CALHM5	NAPSA	CAHM
CALHM6	NAT1	CALB1
CALM1	NAT2	CALB2
CALM1A	NBAT1	CALCA
CALM1B	NBL1	CALCB
CALM2	NBN	CALCOCO1
CALM2B	NBR2	CALCOCO2
CALM3	NCALD	CALCR
CALM4	NCAM1	CALCRL
CALML3	NCBP1	CALD1
CALML4	NCKAP1	CALHM1
CALML5	NCL	CALHM2
CALML6	NCOA3	CALHM3
CALN1	NCOA4	CALHM4
CALR	NCOR1	CALHM5
CALR3	NCR1	CALHM6
CALU	NCR3	CALM1

CALY	NDC1	CALM1A
CAMK1	NDC80	CALM1B
CAMK1D	NDRG1	CALM2
CAMK1G	NDRG3	CALM2B
CAMK1GA	NDUFA4L2	CALM3
CAMK2A	NDUFS1	CALM4
CAMK2B	NDUFS8	CALML3
CAMK2D	NEAT1	CALML4
CAMK2D2	NEBL	CALML5
CAMK2G	NECAB3	CALML6
CAMK2N1	NECTIN4	CALN1
CAMK2N2	NEDD4	CALR
CAMK4	NEDD4L	CALR3
CAMKK1	NEDD9	CALU
CAMKK1B	NEFL	CALY
CAMKK2	NEIL1	CAMK1
CAMKMT	NEK2	CAMK1D
CAMKV	NEK4	CAMK1G
CAML	NEMF	CAMK1GA
CAMLG	NES	CAMK2A
CAMP	NET1	CAMK2B
CAMSAP1	NEU1	CAMK2D
CAMSAP2	NEU3	CAMK2D2
CAMSAP3	NEURL1	CAMK2G
CAMTA1	NEUROD1	CAMK2N1
CAMTA2	NF2	CAMK2N2
CAND1	NFASC	CAMK4
CAND2	NFAT5	CAMKK1
CANT1	NFATC1	CAMKK1B
CANX	NFE2	CAMKK2
CAP1	NFE2L2	CAMKMT
CAP2	NFKB1	CAMKV
CAPG	NFKB2	CAML
CAPN1	NFKBIA	CAMLG
CAPN10	NGB	CAMP
CAPN11	NGF	CAMSAP1

CAPN12	NHLH1	CAMSAP2
CAPN13	NID1	CAMSAP3
CAPN14	NID2	CAMTA1
CAPN15	NIPBL	CAMTA2
CAPN2	NIPSNAP2	CAND1
CAPN3	NIT1	CAND2
CAPN5	NKD1	CANT1
CAPN6	NKILA	CANX
CAPN7	NKTR	CAP1
CAPN8	NKX2-1	CAP2
CAPN8.3.L	NKX2-8	CAPG
CAPN9	NLK	CAPN1
CAPNS1	NLRP1	CAPN10
CAPNS2	NLRP2	CAPN11
CAPRIN1	NLRP3	CAPN12
CAPRIN2	NM	CAPN13
CAPS	NMB	CAPN14
CAPS2	NMBR	CAPN15
CAPSL	NME1	CAPN2
CAPZA1	NME1-NME2	CAPN3
CAPZA2	NME2	CAPN5
CAPZB	NME4	CAPN6
CAR1	NMRAL2P	CAPN7
CAR11	NMU	CAPN8
CAR12	NNAT	CAPN8.3.L
CAR13	NNMT	CAPN9
CAR14	NNT	CAPNS1
CAR2	NNT-AS1	CAPNS2
CAR3	NOB1	CAPRIN1
CAR4	NOCT	CAPRIN2
CAR5B	NOD2	CAPS
CAR8	NOL3	CAPS2
CAR9	NOLC1	CAPSL
CARD10	NORAD	CAPZA1
CARD11	NOS1	CAPZA2
CARD14	NOS2	CAPZB

CARD16	NOS3	CAR1
CARD18	NOTCH1	CAR11
CARD19	NOTCH2	CAR12
CARD6	NOTCH3	CAR13
CARD8	NOTCH4	CAR14
CARD8-AS1	NOVA1	CAR2
CARD9	NOX4	CAR3
CARF	NPAS2	CAR4
CARHSP1	NPM1	CAR5B
CARM1	NPRL2	CAR8
CARM1L	NQO1	CAR9
CARMIL1	NR0B2	CARD10
CARMIL2	NR1H2	CARD11
CARMIL3	NR1H3	CARD14
CARMN	NR1I2	CARD16
CARNMT1	NR1I3	CARD18
CARNS1	NR2C2	CARD19
CARS	NR2C2AP	CARD6
CARS1	NR2F2-AS1	CARD8
CARS2	NR2F6	CARD8-AS1
CARTPT	NR3C1	CARD9
CASC1	NR4A1	CARF
CASC15	NR4A3	CARHSP1
CASC16	NR5A2	CARM1
CASC2	NRARP	CARM1L
CASC3	NRAS	CARMIL1
CASC9	NRG1	CARMIL2
CASD1	NRN1	CARMIL3
CASK	NRP1	CARMN
CASKIN1	NRSN2	CARNMT1
CASKIN2	NSD3	CARNS1
CASP	NT5C2	CARS
CASP1	NT5E	CARS1
CASP10	NTF3	CARS2
CASP12	NTHL1	CARTPT
CASP14	NTN1	CASC1

CASP16P	NTRK1	CASC11
CASP2	NTRK2	CASC15
CASP3	NTRK3	CASC16
CASP3A	NTS	CASC19
CASP4	NTSR1	CASC2
CASP4LP	NUAK1	CASC3
CASP5	NUDT1	CASC8
CASP6	NUF2	CASC9
CASP7	NUMB	CASD1
CASP8	NUP62	CASK
CASP8AP2	NUP98	CASKIN1
CASP9	NUPR1	CASKIN2
CASQ1	NUS1	CASP
CASQ2	NUS1P3	CASP1
CASR	NUSAP1	CASP10
CASS4	NXT1	CASP12
CAST	OAT	CASP14
CASTOR1	OAZ1	CASP16P
CASTOR2	OBP2A	CASP2
CASTOR3P	OCA2	CASP3
CASZ1	ODC1	CASP3A
CAT	OGFRP1	CASP4
CAT2	OGG1	CASP4LP
CATIP	OGN	CASP5
CATP-3	OLFM4	CASP6
CATP-4	OLIG1	CASP7
CATSPER1	ONECUT2	CASP8
CATSPER2	OPCML	CASP8AP2
CATSPER3	OPLAH	CASP9
CATSPER4	OPN1LW	CASQ1
CATSPERB	OPRK1	CASQ2
CATSPERD	OPRL1	CASR
CATSPERE	OPRM1	CASS4
CATSPERG	OR2J3	CAST
CATSPERG2	OR3A4P	CASTOR1
CAV1	ORAI1	CASTOR2

CAV2	ORAI3	CASTOR3P
CAV3	ORM1	CASZ1
CAVIN1	OSMR	CAT
CAVIN1.L	OSR1	CAT2
CAVIN2	OTUB2	CATIP
CAVIN2.S	OTUD3	CATP-3
CAVIN3	OTUD4	CATP-4
CAVIN4.S	OTUD6B	CATSPER1
CAVIN4A	OTUD7B	CATSPER2
CAVIN4B	OXER1	CATSPER3
CAX2	OXTR	CATSPER4
CBARP	P2RX5	CATSPERB
CBFA2T2	P4HB	CATSPERD
CBFA2T3	PABPN1	CATSPERE
CBFB	PADI1	CATSPERG
CBL	PADI4	CATSPERG2
CBLB	PAEP	CAV1
CBLC	PAFAH1B1	CAV2
CBLIF	PAG1	CAV3
CBLL1	PAGE5	CAVIN1
CBLN1	PAGR1	CAVIN1.L
CBLN2	PAH	CAVIN2
CBLN4	PAK1	CAVIN2.S
CBR1	PAK2	CAVIN3
CBR1L	PAK4	CAVIN4.S
CBR2	PALB2	CAVIN4A
CBR3	PANDAR	CAVIN4B
CBR4	PAOX	CAX2
CBS	PAPPA	CBARP
CBWD1	PAPSS1	CBFA2T2
CBX1	PAQR3	CBFA2T3
CBX2	PAQR4	CBFB
CBX3	PARD6A	CBL
CBX4	PARK7	CBLB
CBX5	PARN	CBLC
CBX6	PARP1	CBLIF

CBX7	PART1	CBLL1
CBX8	PAWR	CBLL2
CBY1	PAX5	CBLN1
CBY2	PAX6	CBLN2
CBY3	PAX8	CBLN4
CC2D1A	PBK	CBR1
CC2D1B	PBX1	CBR1L
CC2D2A	PBX2	CBR2
CC2D2B	PC	CBR3
CCAR1	PCAP	CBR4
CCAR2	PCAT1	CBS
CCBE1	PCAT19	CBWD1
CCDC102A	PCAT6	CBX1
CCDC102B	PCAT7	CBX2
CCDC103	PCBP4	CBX3
CCDC105	PCDH10	CBX4
CCDC106	PCDH20	CBX5
CCDC107	PCDH7	CBX6
CCDC110	PCDHB13	CBX7
CCDC112	PCK2	CBX8
CCDC113	PCLAF	CBY1
CCDC115	PCNA	CBY2
CCDC116	PCNA-AS1	CBY3
CCDC117	PCNX3	CC2D1A
CCDC12	PCSK4	CC2D1B
CCDC120	PCSK9	CC2D2A
CCDC121	PDB1	CC2D2B
CCDC122	PDC	CCAR1
CCDC124	PDCD1	CCAR2
CCDC125	PDCD10	CCAT1
CCDC126	PDCD1LG2	CCAT2
CCDC127	PDCD2	CCBE1
CCDC13	PDCD4	CCDC102A
CCDC130	PDCD6	CCDC102B
CCDC134	PDCD6IP	CCDC103
CCDC136	PDE10A	CCDC105

CCDC137	PDE3A	CCDC106
CCDC138	PDE4D	CCDC107
CCDC13-AS1	PDE5A	CCDC110
CCDC14	PDGFRA	CCDC112
CCDC140	PDGFRB	CCDC113
CCDC141	PDIA2	CCDC115
CCDC144A	PDIA3	CCDC116
CCDC144BP	PDIA3P1	CCDC117
CCDC144CP	PDIA6	CCDC12
CCDC144NL	PDIK1L	CCDC120
CCDC146	PDK1	CCDC121
CCDC148	PDLIM7	CCDC122
CCDC149	PDPK1	CCDC124
CCDC15	PDPN	CCDC125
CCDC150	PDSS2	CCDC126
CCDC152	PDXK	CCDC127
CCDC153	PDYN	CCDC13
CCDC154	PEA15	CCDC130
CCDC157	PEBP1	CCDC134
CCDC158	PEBP4	CCDC136
CCDC159	PECAM1	CCDC137
CCDC160	PELI3	CCDC138
CCDC162	PELP1	CCDC13-AS1
CCDC162P	PER1	CCDC14
CCDC163	PER2	CCDC140
CCDC166	PER3	CCDC141
CCDC167	PEX19	CCDC144A
CCDC169	PFKFB3	CCDC144BP
CCDC17	PFN2	CCDC144CP
CCDC170	PGAM1	CCDC144NL
CCDC171	PGAM5	CCDC146
CCDC172	PGF	CCDC148
CCDC174	PGK1	CCDC149
CCDC175	PGR	CCDC15
CCDC177	PHACTR2	CCDC150
CCDC178	PHB	CCDC152

CCDC18	PHF20	CCDC153
CCDC180	PHF5A	CCDC154
CCDC181	PHF8	CCDC157
CCDC184	PHGDH	CCDC158
CCDC185	PHLDA2	CCDC159
CCDC186	PHLPP1	CCDC160
CCDC188	PHLPP2	CCDC162
CCDC18-AS1	PHRF1	CCDC162P
CCDC190	PHTF2	CCDC163
CCDC191	PIAS1	CCDC166
CCDC192	PIAS3	CCDC167
CCDC196	PIAS4	CCDC169
CCDC197	PICART1	CCDC17
CCDC198	PIDD1	CCDC170
CCDC200	PIEZO2	CCDC171
CCDC22	PIGR	CCDC172
CCDC24	PIK3C2B	CCDC174
CCDC25	PIK3C3	CCDC175
CCDC26	PIK3CA	CCDC177
CCDC27	PIK3CB	CCDC178
CCDC28A	PIK3CD	CCDC18
CCDC28A-AS1	PIK3CG	CCDC180
CCDC28B	PIK3R1	CCDC181
CCDC3	PIK3R2	CCDC184
CCDC30	PIK3R3	CCDC185
CCDC32	PIM1	CCDC186
CCDC32.L	PIN1	CCDC188
CCDC33	PINK1	CCDC18-AS1
CCDC34	PINX1	CCDC190
CCDC37	PITX1	CCDC191
CCDC38	PITX2	CCDC192
CCDC39	PIWIL1	CCDC196
CCDC40	PIWIL4	CCDC197
CCDC42	PJA2	CCDC198
CCDC43	PKD1	CCDC200
CCDC47	PKM	CCDC22

CCDC50	PKMYT1	CCDC24
CCDC51	PKNOX1	CCDC25
CCDC54	PKP2	CCDC26
CCDC56	PKP3	CCDC27
CCDC57	PKP4	CCDC28A
CCDC59	PLA2G10	CCDC28A-AS1
CCDC6	PLA2G1B	CCDC28B
CCDC60	PLA2G2A	CCDC3
CCDC61	PLA2G4A	CCDC30
CCDC62	PLA2G6	CCDC32
CCDC63	PLAAT4	CCDC32.L
CCDC65	PLAC1	CCDC33
CCDC66	PLAC8	CCDC34
CCDC68	PLAU	CCDC37
CCDC69	PLAUR	CCDC38
CCDC7	PLB1	CCDC39
CCDC70	PLBD1	CCDC40
CCDC71	PLCB3	CCDC42
CCDC71L	PLCB4	CCDC43
CCDC73	PLCE1	CCDC47
CCDC74A	PLCH1	CCDC50
CCDC74B	PLEK2	CCDC51
CCDC77	PLF	CCDC54
CCDC78	PLG	CCDC56
CCDC8	PLK1	CCDC57
CCDC80	PLOD2	CCDC59
CCDC81	PLP2	CCDC6
CCDC82	PLSCR4	CCDC60
CCDC83	PMAIP1	CCDC61
CCDC84	PMCH	CCDC62
CCDC85A	PML	CCDC63
CCDC85B	PNO1	CCDC65
CCDC85C	PNOC	CCDC66
CCDC86	POGLUT1	CCDC68
CCDC87	POLA2	CCDC69
CCDC88A	POLDIP2	CCDC7

CCDC88B	POLE	CCDC70
CCDC88C	POLK	CCDC71
CCDC89	POLR2A	CCDC71L
CCDC9	POMC	CCDC73
CCDC90B	PON1	CCDC74A
CCDC91	POSTN	CCDC74B
CCDC92	POTEF	CCDC77
CCDC93	POU2F1	CCDC78
CCDC96	POU3F1	CCDC8
CCDC97	POU5F1	CCDC80
CCDC9B	POU5F1P3	CCDC81
CCER1	POU5F1P4	CCDC82
CCER2	PPA1	CCDC83
CCHCR1	PPARA	CCDC84
CCK	PPARD	CCDC85A
CCKAR	PPARG	CCDC85B
CCKBR	PPARGC1A	CCDC85C
CCL1	PPBP	CCDC86
CCL11	PPDPF	CCDC87
CCL12	PPIA	CCDC88A
CCL13	PPIB	CCDC88B
CCL14	PPIG	CCDC88C
CCL15	PPIP5K1	CCDC89
CCL15-CCL14	PPM1D	CCDC9
CCL16	PPP1R11	CCDC90B
CCL17	PPP1R12C	CCDC91
CCL18	PPP1R13B	CCDC92
CCL19	PPP1R13L	CCDC93
CCL2	PPP1R2C	CCDC96
CCL20	PPP2R2A	CCDC97
CCL21	PPP5C	CCDC9B
CCL21B	PPT1	CCEPR
CCL22	PRAME	CCER1
CCL23	PRAP1	CCER2
CCL24	PRC1	CCHCR1
CCL25	PRCC	CCK

CCL26	PRDM1	CCKAR
CCL27	PRDM14	CCKBR
CCL27A	PRDX1	CCL1
CCL28	PRDX2	CCL11
CCL3	PRDX5	CCL12
CCL3L1	PRDX6	CCL13
CCL3L3	PRG1	CCL14
CCL4	PRG2	CCL15
CCL5	PRIMA1	CCL15-CCL14
CCL6	PRKAA1	CCL16
CCL7	PRKAA2	CCL17
CCL8	PRKAB1	CCL18
CCL9	PRKACG	CCL19
CCM2	PRKAR1A	CCL2
CCM2L	PRKCA	CCL20
CCN1	PRKCB	CCL21
CCN2	PRKCD	CCL21B
CCN2.L	PRKCI	CCL22
CCN2A	PRKD1	CCL23
CCN3	PRKDC	CCL24
CCN4	PRKN	CCL25
CCN5	PRL	CCL26
CCNA1	PRMT1	CCL27
CCNA2	PRMT5	CCL27A
CCNB1	PRMT7	CCL28
CCNB1IP1	PRNCR1	CCL3
CCNB2	PROM1	CCL3L1
CCNB3	PROP1	CCL3L3
CCNC	PROS1	CCL4
CCND1	PRPF31	CCL5
CCND2	PRPF38B	CCL6
CCND2A	PRPF40A	CCL7
CCND3	PRR11	CCL8
CCNDBP1	PRR13	CCL9
CCNE1	PRRT2	CCM2
CCNE2	PRRX1	CCM2L

CCNF	PRRX2	CCN1
CCNG1	PRSS3	CCN2
CCNG2	PRSS50	CCN2.L
CCNH	PRSS55	CCN2A
CCNI	PRSS8	CCN3
CCNI2	PRTN3	CCN4
CCNJ	PSAT1	CCN5
CCNJL	PSCA	CCN6
CCNK	PSG1	CCNA1
CCNL1	PSG2	CCNA2
CCNL2	PSMA4	CCNB1
CCNO	PSMB6	CCNB1IP1
CCNP	PSMD10	CCNB2
CCNQ	PSMD4	CCNB3
CCNQP1	PSMD7	CCNC
CCNT1	PSMD8	CCND1
CCNT2	PSMD9	CCND2
CCNY	PSME3	CCND2A
CCNYL1	PSMG1	CCND3
CCP110	PSPH	CCNDBP1
CCP84AE	PTAFR	CCNE1
CCPG1	PTBP1	CCNE2
CCR1	PTCH1	CCNF
CCR10	PTEN	CCNG1
CCR2	PTGDR	CCNG2
CCR3	PTGER2	CCNH
CCR4	PTGER4	CCNI
CCR5	PTGES	CCNI2
CCR6	PTGES3	CCNJ
CCR7	PTGIS	CCNJL
CCR8	PTGR1	CCNK
CCR9	PTGS1	CCNL1
CCRL2	PTGS2	CCNL2
CCS	PTHLH	CCNO
CCSAP	PTK2	CCNP
CCSER1	PTK2B	CCNQ

CCSER2	PTK7	CCNQP1
CCT2	PTMA	CCNT1
CCT3	PTMAP4	CCNT2
CCT4	PTN	CCNY
CCT5	PTOV1	CCNYL1
CCT6A	PTP4A3	CCP110
CCT6B	PTPA	CCP84AE
CCT6P1	PTPN1	CCPG1
CCT6P3	PTPN11	CCR1
CCT7	PTPN13	CCR10
CCT8	PTPN3	CCR2
CCT8L1	PTPN4	CCR3
CCT8L2	PTPN6	CCR4
CCZ1	PTPRA	CCR5
CCZ1B	PTPRC	CCR6
CD101	PTPRD	CCR7
CD109	PTPRF	CCR8
CD14	PTPRH	CCR9
CD151	PTPRK	CCRL2
CD160	PTPRT	CCS
CD163	PTRH2	CCSAP
CD163L1	PTTG1	CCSER1
CD164	PTTG1IP	CCSER2
CD164L2	PTTG2	CCT2
CD177	PTTG3P	CCT3
CD180	PUF60	CCT4
CD19	PUM1	CCT5
CD1A	PVT1	CCT6A
CD1B	PWAR1	CCT6B
CD1C	PXN	CCT6P1
CD1D	PXN-AS1	CCT6P3
CD1D1	PYCARD	CCT7
CD1D2	PYCR1	CCT8
CD1E	PYGB	CCT8L1
CD2	QPCT	CCT8L2
CD200	RAB11A	CCZ1

CD200R1	RAB11FIP2	CCZ1B
CD200R1L	RAB14	CD101
CD200R3	RAB17	CD109
CD207	RAB18	CD14
CD209	RAB22A	CD151
CD209B	RAB25	CD160
CD209C	RAB26	CD163
CD209D	RAB27A	CD163L1
CD209F	RAB27B	CD164
CD209G	RAB37	CD164L2
CD22	RAB38	CD177
CD226	RAB4B-EGLN2	CD180
CD24	RAB5A	CD19
CD244	RAB8A	CD1A
CD247	RABEP2	CD1B
CD248	RABEPK	CD1C
CD24A	RABGEF1	CD1D
CD27	RABL3	CD1D1
CD274	RAC1	CD1D2
CD276	RAC2	CD1E
CD27-AS1	RACGAP1	CD2
CD28	RACK1	CD200
CD2AP	RAD17	CD200R1
CD2BP2	RAD18	CD200R1L
CD300A	RAD23B	CD200R3
CD300C	RAD50	CD207
CD300C2	RAD51	CD209
CD300LB	RAD51AP1	CD209B
CD300LF	RAD51B	CD209C
CD300LG	RAD52	CD209D
CD302	RAD9A	CD209F
CD320	RAF1	CD209G
CD33	RAG2	CD22
CD34	RALA	CD226
CD36	RALBP1	CD24
CD37	RALGPS2	CD244

CD38	RANBP9	CD247
CD3D	RAP1A	CD248
CD3E	RAP1GDS1	CD24A
CD3EAP	RAP2B	CD27
CD3G	RARA	CD274
CD3Z	RARB	CD276
CD4	RARRES2	CD27-AS1
CD40	RASA1	CD28
CD40LG	RASD1	CD2AP
CD44	RASGRF1	CD2BP2
CD46	RASGRF2	CD300A
CD47	RASIP1	CD300C
CD48	RASSF1	CD300C2
CD5	RASSF10	CD300LB
CD52	RASSF2	CD300LF
CD53	RASSF3	CD300LG
CD55	RASSF4	CD302
CD58	RASSF5	CD320
CD59	RASSF7	CD33
CD59A	RASSF8	CD34
CD5L	RB1	CD36
CD6	RBAK	CD37
CD63	RBM14	CD38
CD63-AS1	RBM14-RBM4	CD3D
CD68	RBM19	CD3E
CD69	RBM23	CD3EAP
CD7	RBM3	CD3G
CD70	RBM38	CD3Z
CD72	RBM39	CD4
CD74	RBM45	CD40
CD79A	RBM5	CD40LG
CD79B	RBM7	CD44
CD80	RBM8A	CD46
CD81	RBMS3	CD47
CD81.L	RBMX	CD48
CD82	RBP2	CD5

CD83	RBPJ	CD52
CD84	RBX1	CD53
CD86	RCCD1	CD55
CD8A	RCN1	CD58
CD8B	RDH10	CD59
CD9	RDM1	CD59A
CD93	RECK	CD5L
CD96	RECQL	CD6
CD99	REG1A	CD63
CD99L2	RELA	CD63-AS1
CD99P1	REN	CD68
CDA	REST	CD69
CDADC1	RET	CD7
CDAN1	REV3L	CD70
CDC123	RFC1	CD72
CDC14A	RFPL3	CD74
CDC14B	RFWD3	CD79A
CDC14C	RFX5	CD79B
CDC16	RGCC	CD80
CDC20	RGMB	CD81
CDC20B	RGMB-AS1	CD81.L
CDC23	RGN	CD82
CDC25A	RGS17	CD83
CDC25B	RGS3	CD84
CDC25C	RGS4	CD86
CDC26	RGS6	CD8A
CDC27	RHEB	CD8B
CDC34	RHOB	CD9
CDC37	RHOH	CD93
CDC37L1	RHOJ	CD96
CDC40	RHOV	CD99
CDC42	RHPN1-AS1	CD99L2
CDC42BPA	RICTOR	CD99P1
CDC42BPB	RIF1	CDA
CDC42BPG	RIN1	CDADC1
CDC42EP1	RING1	CDAN1

CDC42EP2	RIOK2	CDC123
CDC42EP3	RIPK1	CDC14A
CDC42EP4	RIPK3	CDC14B
CDC42EP5	RITA1	CDC14C
CDC42L2	RMDN1	CDC16
CDC42SE1	RMDN2	CDC20
CDC42SE2	RMDN3	CDC20B
CDC45	RMRP	CDC23
CDC5L	RNASE3	CDC25A
CDC6	RND3	CDC25B
CDC7	RNF111	CDC25C
CDC73	RNF146	CDC26
CDCA2	RNF187	CDC27
CDCA3	RNF19A	CDC34
CDCA4	RNF25	CDC37
CDCA5	RNF38	CDC37L1
CDCA7	RNF8	CDC40
CDCA7L	RNPS1	CDC42
CDCA8	RNU1-1	CDC42BPA
CDCP1	ROBO1	CDC42BPB
CDCP2	ROBO3	CDC42BPG
CDH1	ROBO4	CDC42EP1
CDH10	ROCK1	CDC42EP2
CDH11	ROCK2	CDC42EP3
CDH12	ROMO1	CDC42EP4
CDH13	ROPN1L	CDC42EP5
CDH15	ROR1	CDC42L2
CDH16	ROR2	CDC42SE1
CDH17	RORA	CDC42SE2
CDH18	RORB	CDC45
CDH19	RORC	CDC5L
CDH2	ROS1	CDC6
CDH20	RPA1	CDC7
CDH22	RPAIN	CDC73
CDH23	RPE65	CDCA2
CDH24	RPL13A	CDCA3

CDH26	RPL14	CDCA4
CDH3	RPL17	CDCA5
CDH4	RPL17-C18orf32	CDCA7
CDH5	RPL19	CDCA7L
CDH6	RPL22	CDCA8
CDH7	RPL32	CDCP1
CDH8	RPL34	CDCP2
CDH9	RPL36A	CDH1
CDHR1	RPLP0	CDH10
CDHR2	RPN2	CDH11
CDHR3	RPS15A	CDH12
CDHR4	RPS27A	CDH13
CDHR5	RPS3	CDH15
CDIN1	RPS6	CDH16
CDIP1	RPS6KA1	CDH17
CDIPT	RPS6KB1	CDH18
CDIPTOSP	RPS6KB2	CDH19
CDK1	RPSA	CDH2
CDK10	RRAD	CDH20
CDK11A	RRM1	CDH22
CDK11B	RRM2	CDH23
CDK12	RRM2B	CDH24
CDK13	RSF1	CDH26
CDK14	RSS	CDH3
CDK15	RTL10	CDH4
CDK16	RTN4	CDH5
CDK17	RTN4R	CDH6
CDK18	RTRAF	CDH7
CDK19	RUNX1	CDH8
CDK2	RUNX2	CDH9
CDK20	RUNX3	CDHR1
CDK2AP1	RXRG	CDHR2
CDK2AP2	RYBP	CDHR3
CDK3	S100A1	CDHR4
CDK4	S100A11	CDHR5
CDK5	S100A12	CDIN1

CDK5R1	S100A13	CDIP1
CDK5R2	S100A14	CDIPT
CDK5RAP1	S100A2	CDIPTOSP
CDK5RAP2	S100A4	CDK1
CDK5RAP3	S100A6	CDK10
CDK6	S100A7	CDK11A
CDK7	S100A7A	CDK11B
CDK8	S100A8	CDK12
CDK9	S100A9	CDK13
CDKAL1	S100B	CDK14
CDKL1	SAA1	CDK15
CDKL2	SAA2	CDK16
CDKL3	SAGE1	CDK17
CDKL4	SAI1	CDK18
CDKL5	SALL4	CDK19
CDKN1A	SAMD9	CDK2
CDKN1B	SARDH	CDK20
CDKN1C	SARNP	CDK2AP1
CDKN2A	SART3	CDK2AP2
CDKN2AIP	SAT1	CDK3
CDKN2AIPNL	SATB1	CDK4
CDKN2B	SATB2	CDK5
CDKN2B-AS1	SAXO1	CDK5R1
CDKN2C	SBF2-AS1	CDK5R2
CDKN2D	SBNO1	CDK5RAP1
CDKN3	SBNO2	CDK5RAP2
CDNF	SCAF1	CDK5RAP3
CDO1	SCAF11	CDK6
CDON	SCD	CDK7
CDPF1	SCGB1A1	CDK8
CDR1	SCGB3A1	CDK9
CDR-1	SCLC1	CDKAL1
CDR2	SCO2	CDKL1
CDR2L	SCUBE2	CDKL2
CDR-4	SCYL1	CDKL3
CDRT15	SDC4	CDKL4

CDRT15P1	SDF4	CDKL5
CDRT15P3	SDHA	CDKN1A
CDRT4	SDHAF2	CDKN1B
CDS1	SDHC	CDKN1C
CDS2	SEA	CDKN2A
CDSN	SEC14L2	CDKN2A-DT
CDT1	SEC31A	CDKN2AIP
CDV3	SEC61G	CDKN2AIPNL
CDX1	SEC62	CDKN2B
CDX2	SEL1L	CDKN2B-AS1
CDX4	SELE	CDKN2C
CDY2A	SELENOF	CDKN2D
CDYL	SELENOP	CDKN3
CDYL2	SELENOS	CDNF
CEACAM1	SEMA3A	CDO1
CEACAM10	SEMA3B	CDON
CEACAM12	SEMA4B	CDPF1
CEACAM16	SEMA4C	CDR1
CEACAM19	SEMA4D	CDR-1
CEACAM3	SEMA5A	CDR1-AS
CEACAM5	SEMA6A	CDR2
CEACAM6	SENP1	CDR2L
CEACAM7	SEPTIN7	CDR3
CEACAM8	SERBP1	CDR-4
CEACAM9	SERINC1	CDRT15
CEBP1	SERINC2	CDRT15P1
CEBPA	SERPINA1	CDRT15P3
CEBPA-DT	SERPINA3	CDRT4
CEBPB	SERPINB2	CDS1
CEBPD	SERPINB3	CDS2
CEBPE	SERPINB4	CDSN
CEBPG	SERPINB5	CDT1
CEBPZ	SERPINE1	CDV3
CEBPZOS	SERPINF1	CDX1
CECA2	SESN2	CDX2
CECR2	SESN3	CDX4

CECR3	SET	CDY2A
CECR7	SETD2	CDYL
CED-13	SETD5	CDYL2
CED-3	SETD7	CEACAM1
CED-4	SETDB1	CEACAM10
CED-9	SEZ6L	CEACAM12
CEH-14	SF3A3	CEACAM16
CEL	SF3B3	CEACAM19
CELA1	SFN	CEACAM3
CELA2A	SFRP1	CEACAM5
CELA3B	SFRP2	CEACAM6
CELF1	SFRP5	CEACAM7
CELF2	SFTA1P	CEACAM8
CELF2-AS1	SFTPA1	CEACAM9
CELF3	SFTPA2	CEBP1
CELF3A	SFTPB	CEBPA
CELF4	SFTPC	CEBPA-DT
CELF5	SFTPD	CEBPB
CELF6	SGK1	CEBPD
CELP	SGMS1	CEBPE
CELSR1	SGO1	CEBPG
CELSR1A	SGSM3	CEBPZ
CELSR2	SH2B1	CEBPZOS
CELSR3	SH3GL2	CECA2
CEMIP	SHC1	CECR2
CEMIP2	SHCBP1	CECR3
CEMP1	SHISA3	CECR7
CENATAC	SHMT2	CED-13
CEND1	SHOC2	CED-3
CENPA	SHOX2	CED-4
CENPB	SHQ1	CED-9
CENPBD1P	SHTN1	CEH-14
CENPC	SIAH2	CEL
CENPC1	SIGLEC9	CELA1
CENPE	SIK1	CELA2A
CENPF	SIK1B	CELA3B

CENPH	SIK3	CELF1
CENPI	SIM2	CELF2
CENPJ	SIN3A	CELF2-AS1
CENPK	SIPA1	CELF3
CENPL	SIRT1	CELF3A
CENPM	SIRT2	CELF4
CENPN	SIRT3	CELF5
CENPO	SIRT4	CELF6
CENPP	SIRT5	CELIAC2
CENPQ	SIRT6	CELP
CENPS	SIRT7	CELSR1
CENPT	SIVA1	CELSR1A
CENPU	SIX1	CELSR2
CENPV	SIX2	CELSR3
CENPVL1	SIX4	CEMIP
CENPW	SIX6	CEMIP2
CENPX	SKAP2	CEMP1
CEP-1	SKIL	CENATAC
CEP104	SKP2	CEND1
CEP112	SLAMF7	CENPA
CEP120	SLC10A2	CENPB
CEP126	SLC11A1	CENPBD1P
CEP128	SLC11A2	CENPC
CEP131	SLC12A2	CENPC1
CEP135	SLC12A3	CENPE
CEP152	SLC12A9	CENPF
CEP162	SLC14A2	CENPH
CEP170	SLC15A1	CENPI
CEP170B	SLC16A1	CENPJ
CEP170P1	SLC16A3	CENPK
CEP19	SLC16A4	CENPL
CEP192	SLC16A7	CENPM
CEP20	SLC17A5	CENPN
CEP250	SLC19A1	CENPO
CEP290	SLC1A2	CENPP
CEP295	SLC1A5	CENPQ

CEP295NL	SLC22A1	CENPS
CEP350	SLC22A10	CENPT
CEP41	SLC22A16	CENPU
CEP43	SLC22A18	CENPV
CEP44	SLC25A1	CENPVL1
CEP55	SLC25A11	CENPW
CEP57	SLC25A16	CENPX
CEP57L1	SLC25A5	CEP-1
CEP63	SLC26A2	CEP104
CEP68	SLC28A1	CEP112
CEP70	SLC29A1	CEP120
CEP72	SLC29A3	CEP126
CEP76	SLC2A1	CEP128
CEP78	SLC2A3	CEP131
CEP83	SLC2A4RG	CEP135
CEP85	SLC2A5	CEP152
CEP85L	SLC31A1	CEP162
CEP89	SLC34A2	CEP170
CEP95	SLC35F2	CEP170B
CEP97	SLC38A3	CEP170P1
CEPT1	SLC38A7	CEP19
CER1	SLC39A4	CEP192
CERCAM	SLC39A6	CEP20
CERK	SLC3A2	CEP250
CERKL	SLC44A4	CEP290
CEROX1	SLC46A1	CEP295
CERS1	SLC4A7	CEP295NL
CERS2	SLC52A2	CEP350
CERS3	SLC5A1	CEP41
CERS4	SLC5A2	CEP43
CERS5	SLC5A5	CEP44
CERS5.L	SLC6A2	CEP55
CERS6	SLC6A3	CEP57
CERS6-AS1	SLC6A5	CEP57L1
CERT1	SLC6A8	CEP63
CES1	SLC7A11	CEP68

CES1B	SLC7A5	CEP70
CES1C	SLC9A3R2	CEP72
CES1D	SLCO1B1	CEP76
CES1E	SLCO1B3	CEP78
CES1F	SLCO6A1	CEP83
CES1G	SLIT2	CEP85
CES1P1	SLPI	CEP85L
CES2	SMAD1	CEP89
CES2A	SMAD2	CEP95
CES2B	SMAD3	CEP97
CES2C	SMAD4	CEPT1
CES2E	SMAD5	CER1
CES2G	SMAD6	CERCAM
CES2H	SMAD7	CERK
CES3	SMARCA1	CERKL
CES3A	SMARCA2	CERNA2
CES3B	SMARCA4	CEROX1
CES4A	SMARCA5	CERS1
CESL1	SMARCD1	CERS2
CETN1	SMARCE1	CERS3
CETN2	SMN1	CERS4
CETN3	SMN2	CERS5
CETP	SMO	CERS5.L
CFAP100	SMPD1	CERS6
CFAP100-DT	SMPX	CERS6-AS1
CFAP107	SMR3B	CERT1
CFAP119	SMS	CES1
CFAP126	SMUG1	CES1B
CFAP141	SMYD2	CES1C
CFAP157	SNAI1	CES1D
CFAP161	SNAI2	CES1E
CFAP20	SNAI3	CES1F
CFAP206	SNAP47	CES1G
CFAP20DC	SNCA	CES1P1
CFAP210	SND1	CES2
CFAP221	SNHG1	CES2A

CFAP251	SNHG12	CES2B
CFAP276	SNHG14	CES2C
CFAP298	SNHG15	CES2E
CFAP299	SNHG16	CES2G
CFAP300	SNHG17	CES2H
CFAP36	SNHG20	CES3
CFAP410	SNHG3	CES3A
CFAP418	SNHG7	CES3B
CFAP43	SNHG8	CES4A
CFAP44	SNIP1	CESL1
CFAP45	SNORA3A	CETN1
CFAP46	SNORA71A	CETN2
CFAP47	SNORA78	CETN3
CFAP52	SNORA80E	CETP
CFAP53	SNORD118	CFAP100
CFAP54	SNORD138	CFAP100-DT
CFAP57	SNORD14B	CFAP107
CFAP58	SNORD14C	CFAP119
CFAP61	SNORD14D	CFAP126
CFAP65	SNORD14E	CFAP141
CFAP69	SNORD15A	CFAP157
CFAP70	SNORD35B	CFAP161
CFAP73	SNORD66	CFAP20
CFAP74	SNORD76	CFAP206
CFAP77	SNORD78	CFAP20DC
CFAP91	SNRPB	CFAP210
CFAP92	SNRPD3	CFAP221
CFAP95	SNX1	CFAP251
CFAP97	SOAT1	CFAP276
CFAP97D1	SOCS1	CFAP298
CFAP99	SOCS2	CFAP299
CFB	SOCS3	CFAP300
CFC1	SOCS6	CFAP36
CFD	SOD1	CFAP410
CFDP1	SOD2	CFAP418
CFH	SOS1	CFAP43

CFHR1	SOS2	CFAP44
CFHR2	SOSTDC1	CFAP45
CFHR3	SOX1	CFAP46
CFHR5	SOX10	CFAP47
CFI	SOX13	CFAP52
CFL1	SOX17	CFAP53
CFL1P1	SOX18	CFAP54
CFL2	SOX2	CFAP57
CFLAR	SOX2-OT	CFAP58
CFLAR-AS1	SOX30	CFAP61
CFP	SOX4	CFAP65
CFTR	SOX5	CFAP69
CGA	SOX6	CFAP70
CGAS	SOX7	CFAP73
CGB1	SOX8	CFAP74
CGB2	SOX9	CFAP77
CGB3	SP1	CFAP91
CGB5	SPA17	CFAP92
CGB7	SPAAR	CFAP95
CGB8	SPAG5	CFAP97
CGGBP1	SPAG6	CFAP97D1
CGN	SPAG9	CFAP99
CGNL1	SPARC	CFB
CGREF1	SPARCL1	CFC1
CGRRF1	SPATA2	CFD
CH25H	SPC24	CFDP1
CHAC1	SPC25	CFH
CHAC2	SPEN	CFHR1
CHAD	SPG7	CFHR2
CHADL	SPHK1	CFHR3
CHAF1A	SPHK2	CFHR5
CHAF1B	SPIN1	CFI
CHAMP1	SPINK1	CFL1
CHASERR	SPINT1	CFL1P1
CHAT	SPINT2	CFL2
CHCHD1	SPN	CFLAR

CHCHD10	SPNS2	CFLAR-AS1
CHCHD2	SPOCK1	CFP
CHCHD3	SPOP	CFTR
CHCHD4	SPP1	CGA
CHCHD5	SPRY2	CGAS
CHCHD6	SPRY4	CGB1
CHCHD7	SPRY4-IT1	CGB2
CHD1	SPTB	CGB3
CHD1L	SPZ1	CGB5
CHD2	SQSTM1	CGB7
CHD3	SRA1	CGB8
CHD4	SRC	CGGBP1
CHD5	SRCIN1	CGN
CHD6	SREBF2	CGNL1
CHD7	SRF	CGREF1
CHD8	SRGN	CGRRF1
CHD9	SRL	CH25H
CHDH	SRPK1	CHAC1
CHEK1	SRPK2	CHAC2
CHEK2	SRRM2	CHAD
CHEK2P2	SRSF1	CHADL
CHERP	SRSF2	CHAF1A
CHFR	SRSF5	CHAF1B
CHGA	SRY	CHAMP1
CHGB	SSBP1	CHASERR
CHI3L1	SSR1	CHAT
CHI3L2	SSRP1	CHCHD1
CHI3L3	SST	CHCHD10
CHI3L4	SSTR2	CHCHD2
CHIA	SSTR4	CHCHD3
CHIAP2	ST13	CHCHD4
CHIC1	ST6GAL1	CHCHD5
CHIC2	ST8SIA2	CHCHD6
CHID1	STARD13	CHCHD7
CHIL1	STARD8	CHD1
CHIL3	STAT1	CHD1L

CHIL4	STAT2	CHD2
CHIT1	STAT3	CHD3
CHKA	STAT5A	CHD4
CHKB	STAT5B	CHD5
CHKB-CPT1B	STAT6	CHD6
CHKB-DT	STC1	CHD7
CHL1	STEAP1	CHD8
CHL1-AS1	STIM1	CHD9
CHL1-AS2	STIP1	CHDH
CHM	STK11	CHEK1
CHML	STK33	CHEK2
CHMP1A	STK39	CHEK2P2
CHMP1B	STMN1	CHERP
CHMP2A	STMN3	CHFR
CHMP2B	STOM	CHGA
CHMP3	STOML2	CHGB
CHMP4A	STPG4	CHI3L1
CHMP4B	STRN4	CHI3L2
CHMP4C	STS	CHI3L3
CHMP5	STUB1	CHI3L4
CHMP6	STXBP5	CHIA
CHMP7	STXBP5-AS1	CHIAP2
CHN1	STYK1	CHIC1
CHN2	SUB1	CHIC2
CHODL	SULF2	CHID1
CHORDC1	SUMO1	CHIL1
CHP	SUMO1P3	CHIL3
CHP1	SUSD6	CHIL4
CHP2	SUV39H1	CHIT1
CHPF	SYBU	CHKA
CHPF2	SYCE1L	CHKB
CHPT1	SYCP3	CHKB-CPT1B
CHRAC1	SYF2	CHKB-DT
CHRD	SYK	CHL1
CHRDL1	SYP	CHL1-AS1
CHRDL2	SYT1	CHL1-AS2

CHRFAM7A	SYT7	CHM
CHRM1	SYTL2	CHML
CHRM2	SYVN1	CHMP1A
CHRM3	TAB1	CHMP1B
CHRM4	TAB3	CHMP2A
CHRM5	TAC1	CHMP2B
CHRNA1	TACC3	CHMP3
CHRNA1.2.L	TACSTD2	CHMP4A
CHRNA10	TADA3	CHMP4B
CHRNA2	TAM	CHMP4C
CHRNA3	TAP1	CHMP5
CHRNA4	TAPBP	CHMP6
CHRNA5	TARBP1	CHMP7
CHRNA6	TARBP2	CHN1
CHRNA7	TARDBP	CHN2
CHRNA9	TAT	CHODL
CHRNB1	TATDN1	CHORDC1
CHRNB2	TAZ	CHP
CHRNB3	TBC1D7	CHP1
CHRNB4	TBC1D9	CHP2
CHRND	TBILA	CHPF
CHRNE	TBK1	CHPF2
CHRNG	TBL1XR1	CHPT1
CHROMR	TBP	CHRAC1
CHST1	TBPL1	CHRD
CHST10	TBX1	CHRDL1
CHST11	TBX2	CHRDL2
CHST12	TBX21	CHRFAM7A
CHST13	TBX3	CHRM1
CHST14	TBX5	CHRM2
CHST15	TCEAL7	CHRM3
CHST2	TCF19	CHRM4
CHST3	TCF21	CHRM5
CHST3A	TCF4	CHRNA1
CHST4	TCF7	CHRNA1.2.L
CHST5	TCF7L2	CHRNA10

CHST6	TCHP	CHRNA2
CHST7	TCN1	CHRNA3
CHST8	TCTN1	CHRNA4
CHST9	TDP1	CHRNA5
CHSY1	TDP2	CHRNA6
CHSY3	TDRG1	CHRNA7
CHTF18	TEAD1	CHRNA9
CHTF8	TEK	CHRNB1
CHTOP	TENT5A	CHRNB2
CHUK	TERC	CHRNB3
CHURC1	TERF1	CHRNB4
CIA30	TERF2	CHRND
CIAO1	TERF2IP	CHRNE
CIAO2A	TERT	CHRNG
CIAO2B	TES	CHROMR
CIAO3	TESC	CHST1
CIAPIN1	TESMIN	CHST10
CIART	TET1	CHST11
CIB1	TET2	CHST12
CIB2	TF	CHST13
CIB3	TFAM	CHST14
CIBAR1	TFAP2B	CHST15
CIBAR2	TFAP2C	CHST2
CIC	TFDP1	CHST3
CIDEA	TFE3	CHST3A
CIDEB	TFEB	CHST4
CIDEC	TFG	CHST5
CIDECP1	TFIP11	CHST6
CIITA	TFPI	CHST7
CILK1	TFPI2	CHST8
CILP	TFRC	CHST9
CILP2	TG	CHSY1
CINP	TGFA	CHSY3
CIP2A	TGFB1	CHTF18
CIPC	TGFB2	CHTF8
CIR	TGFB3	CHTOP

CIR1	TGFBI	CHUK
CIRBP	TGFBR1	CHURC1
CIRBP-AS1	TGFBR2	CIA30
CISD1	TGFBR3	CIAO1
CISD2	TGIF1	CIAO2A
CISD3	TGIF2	CIAO2B
CISH	TGM2	CIAO3
CIT	TGM5	CIAPIN1
CITED1	THAS	CIART
CITED2	THBD	CIB1
CITED4	THBS1	CIB2
CIZ1	THBS2	CIB3
CK137956	THOP1	CIBAR1
CKAP2	THUMPD3-AS1	CIBAR2
CKAP2L	THY1	CIC
CKAP4	TIA1	CIDEA
CKAP5	TIAM1	CIDEB
CKB	TIAM2	CIDEC
CKBB	TICAM2	CIDECP1
CKLF	TIE1	CIITA
CKM	TIGAR	CILK1
CKMA	TIMD4	CILP
CKMB	TIMELESS	CILP2
CKMT1	TIMM50	CINP
CKMT1A	TIMM8A	CIP2A
CKMT1B	TIMP1	CIPC
CKMT1B.L	TIMP2	CIR
CKMT2	TIMP3	CIR1
CKMT2-AS1	TINAGL1	CIRBP
CKMT2B	TINCR	CIRBP-AS1
CKS1B	TJP1	CISD1
CKS2	TK1	CISD2
CLASP1	TLK1	CISD3
CLASP2	TLR1	CISH
CLASRP	TLR2	CIT
CLBA1	TLR3	CITED1

CLC	TLR4	CITED2
CLCA1	TLR5	CITED4
CLCA2	TLR9	CIZ1
CLCA3	TM4SF1	CK137956
CLCA3P	TM4SF4	CKAP2
CLCA4	TM4SF5	CKAP2L
CLCA4A	TM7SF2	CKAP4
CLCC1	TMED7	CKAP5
CLCF1	TMED7-TICAM2	CKB
CLCN1	TMEFF2	CKBB
CLCN2	TMEM100	CKLF
CLCN3	TMEM106B	CKM
CLCN4	TMEM132D	CKMA
CLCN5	TMEM135	CKMB
CLCN6	TMEM14A	CKMT1
CLCN7	TMEM158	CKMT1A
CLCNKA	TMEM17	CKMT1B
CLCNKB	TMEM88	CKMT1B.L
CLDN1	TMEM97	CKMT2
CLDN10	TMPO	CKMT2-AS1
CLDN11	TMPO-AS1	CKMT2B
CLDN12	TMPRSS11D	CKS1B
CLDN14	TMPRSS13	CKS1BP7
CLDN15	TMPRSS4	CKS2
CLDN16	TMSB10	CLASP1
CLDN17	TMSB4X	CLASP2
CLDN18	TMTC3	CLASRP
CLDN19	TMX2-CTNND1	CLBA1
CLDN2	TNC	CLC
CLDN20	TNF	CLCA1
CLDN22	TNFAIP1	CLCA2
CLDN23	TNFAIP3	CLCA3
CLDN25	TNFAIP8	CLCA3P
CLDN3	TNFAIP8L3	CLCA4
CLDN34C1	TNFRSF10B	CLCA4A
CLDN3C	TNFRSF10C	CLCC1

CLDN3D	TNFRSF10D	CLCF1
CLDN4	TNFRSF11A	CLCN1
CLDN5	TNFRSF11B	CLCN2
CLDN6	TNFRSF12A	CLCN3
CLDN6.1.L	TNFRSF13C	CLCN4
CLDN7	TNFRSF17	CLCN5
CLDN7A	TNFRSF18	CLCN6
CLDN8	TNFRSF1B	CLCN7
CLDN9	TNFRSF4	CLCNKA
CLDND1	TNFRSF9	CLCNKB
CLDND1A	TNFSF10	CLDN1
CLDND2	TNFSF11	CLDN10
CLEC10A	TNFSF12	CLDN11
CLEC11A	TNFSF13	CLDN12
CLEC-123	TNFSF14	CLDN14
CLEC12A	TNFSF4	CLDN15
CLEC12B	TNFSF9	CLDN16
CLEC-143	TNK2	CLDN17
CLEC14A	TNK2-AS1	CLDN18
CLEC16A	TNKS	CLDN19
CLEC17A	TNKS2	CLDN2
CLEC18A	TNNC1	CLDN20
CLEC18B	TNNI1	CLDN22
CLEC18C	TNNI3	CLDN23
CLEC19A	TOB1	CLDN25
CLEC1A	TOB1-AS1	CLDN3
CLEC1B	TOP1	CLDN34C1
CLEC2B	TOP2A	CLDN3C
CLEC2D	TOP2B	CLDN3D
CLEC2H	TOP3A	CLDN4
CLEC2L	TOP3B	CLDN5
CLEC-3	TOPBP1	CLDN6
CLEC3B	TOPORS	CLDN6.1.L
CLEC4A	TP53	CLDN7
CLEC4A2	TP53AIP1	CLDN7A
CLEC4A3	TP53BP1	CLDN8

CLEC4B1	TP53BP2	CLDN9
CLEC4B2	TP53COR1	CLDND1
CLEC4D	TP53I3	CLDND1A
CLEC4E	TP53TG1	CLDND2
CLEC4F	TP63	CLEC10A
CLEC4G	TP73	CLEC11A
CLEC4GP1	TP73-AS1	CLEC-123
CLEC4M	TPD52L1	CLEC12A
CLEC4N	TPM3	CLEC12B
CLEC-5	TPO	CLEC-143
CLEC5A	TPPP2	CLEC14A
CLEC-60	TPPP3	CLEC16A
CLEC-66	TPX2	CLEC17A
CLEC-74	TRA2B	CLEC18A
CLEC7A	TRAF1	CLEC18B
CLEC9A	TRAF2	CLEC18C
CLECL1P	TRAF4	CLEC19A
CLGN	TRAF6	CLEC1A
CLHC1	TRAP1	CLEC1B
CLIC1	TRAT1	CLEC2B
CLIC2	TRBV20OR9-2	CLEC2D
CLIC3	TREH	CLEC2H
CLIC4	TREM1	CLEC2L
CLIC5	TRERF1	CLEC-3
CLIC6	TRE-TTC3-1	CLEC3B
CLINT1	TRIB1	CLEC4A
CLIP1	TRIB3	CLEC4A2
CLIP1-AS1	TRIM13	CLEC4A3
CLIP2	TRIM14	CLEC4B1
CLIP3	TRIM15	CLEC4B2
CLIP4	TRIM16	CLEC4D
CLK1	TRIM16L	CLEC4E
CLK2	TRIM22	CLEC4F
CLK2P1	TRIM24	CLEC4G
CLK3	TRIM25	CLEC4GP1
CLK4	TRIM27	CLEC4M

CLK4A	TRIM28	CLEC4N
CLLU1	TRIM29	CLEC-5
CLMN	TRIM32	CLEC5A
CLMP	TRIM33	CLEC-60
CLN3	TRIM36	CLEC-66
CLN5	TRIM37	CLEC-74
CLN6	TRIM4	CLEC7A
CLN8	TRIM44	CLEC9A
CLNK	TRIM45	CLECL1P
CLOCK	TRIM46	CLGN
CLP1	TRIM47	CLHC1
CLPB	TRIM54	CLIC1
CLPP	TRIM59	CLIC2
CLPS	TRIM65	CLIC3
CLPSL2	TRIM66	CLIC4
CLPTM1	TRIM67	CLIC5
CLPTM1L	TRIM71	CLIC6
CLPX	TRIM9	CLINT1
CLRN1	TRIP10	CLIP1
CLRN1-AS1	TRMO	CLIP1-AS1
CLRN2	TRMT11	CLIP2
CLRN3	TRPC1	CLIP3
CLSPN	TRPC6	CLIP4
CLSTN1	TRPM2-AS	CLK1
CLSTN2	TRPV3	CLK2
CLSTN3	TRPV5	CLK2P1
CLTA	TRPV6	CLK3
CLTB	TSC2	CLK4
CLTC	TSC22D1	CLK4A
CLTCL1	TSHZ1	CLLU1
CLTRN	TSIX	CLMN
CLU	TSN	CLMP
CLUAP1	TSPAN1	CLN3
CLUH	TSPAN12	CLN5
CLUHP3	TSPAN14	CLN6
CLUL1	TSPAN18	CLN8

CLVS1	TSPAN33	CLNK
CLVS2	TSPAN7	CLOCK
CLXN	TSPAN8	CLP1
CLYBL	TTF1	CLPB
CMA1	TTK	CLPP
CMAH	TTN	CLPS
CMAHP	TTTY15	CLPSL2
CMAS	TUBA1B	CLPTM1
CMASA	TUBA3C	CLPTM1L
CMBL	TUBA3D	CLPX
CMC1	TUBA4A	CLRN1
CMC2	TUBA4B	CLRN1-AS1
CMC4	TUBB	CLRN2
CMET	TUBB2A	CLRN3
CMIP	TUBB3	CLSPN
CMKLR1	TUBB4B	CLSTN1
CMKLR2	TUG1	CLSTN2
CML	TUSC2	CLSTN3
CMLC1	TUSC3	CLTA
CMN	TWF1	CLTB
CMPK1	TWIST1	CLTC
CMPK2	TWSG1	CLTCL1
CMSS1	TXN	CLTRN
CMTM1	TXNIP	CLU
CMTM2	TXNRD1	CLUAP1
CMTM3	TXNRD2	CLUH
CMTM4	TYMP	CLUHP3
CMTM5	TYMS	CLUL1
CMTM6	TYR	CLVS1
CMTM7	U2AF2	CLVS2
CMTM8	UBA2	CLXN
CMTR1	UBA52	CLYBL
CMTR2	UBB	CMA1
CMYA5	UBC	CMAH
CNB-1	UBE2C	CMAHP
CNBD1	UBE2F	CMAS

CNBD2	UBE2I	CMASA
CNBP	UBE2L3	CMBL
CNC-11	UBE2N	CMC1
CNDP1	UBE3A	CMC2
CNDP2	UBE3C	CMC4
CNEP1R1	UBR5	CMET
CNFN	UCA1	CMIP
CNFN.1.S	UCHL1	CMKLR1
CNGA1	UCHL5	CMKLR2
CNGA3	UCN	CML
CNGA4	UCN3	CMLC1
CNGB1	UCP1	CMN
CNGB3	UCP2	CMPK1
CNIH1	UCP3	CMPK2
CNIH2	UGCG	CMSS1
CNIH3	UGP2	CMTM1
CNIH4	UGT1A	CMTM2
CNK	UGT1A1	CMTM3
CNKSR1	UGT1A7	CMTM4
CNKSR2	UGT1A9	CMTM5
CNKSR3	UHRF1	CMTM6
CNMD	UHRF2	CMTM7
CNMD.L	UIMC1	CMTM8
CNN1	ULBP1	CMTR1
CNN2	ULK1	CMTR2
CNN3	ULK2	CMYA5
CNNM1	UMPS	CNB-1
CNNM2	UNG	CNBD1
CNNM3	UQCRFS1	CNBD2
CNNM4	URGCP	CNBP
CNO	USE1	CNC-11
CNOT1	USF1	CNDP1
CNOT10	USP1	CNDP2
CNOT11	USP10	CNEP1R1
CNOT2	USP13	CNFN
CNOT3	USP14	CNFN.1.S

CNOT4	USP15	CNGA1
CNOT6	USP17L2	CNGA3
CNOT6L	USP17L24	CNGA4
CNOT7	USP17L25	CNGB1
CNOT8	USP17L26	CNGB3
CNOT9	USP17L27	CNIH1
CNP	USP17L28	CNIH2
CNP1	USP17L29	CNIH3
CNPPD1	USP17L30	CNIH4
CNPY1	USP17L9P	CNK
CNPY2	USP22	CNKSR1
CNPY3	USP25	CNKSR2
CNPY4	USP27X	CNKSR3
CNR1	USP28	CNMD
CNR2	USP44	CNMD.L
CNRIP1	USP49	CNN1
CNST	USP5	CNN2
CNTD1	USP7	CNN3
CNTF	USP8	CNNM1
CNTFR	USP9X	CNNM2
CNTFR-AS1	UTS2R	CNNM3
CNTLN	UVRAG	CNNM4
CNTN1	VASH2	CNO
CNTN2	VAV2	CNOT1
CNTN3	VCAM1	CNOT10
CNTN4	VCL	CNOT11
CNTN5	VCP	CNOT2
CNTN6	VDAC1	CNOT3
CNTNAP1	VDR	CNOT4
CNTNAP2	VEGFA	CNOT6
CNTNAP3	VEGFB	CNOT6L
CNTNAP3B	VEGFC	CNOT7
CNTNAP3P2	VEGFD	CNOT8
CNTNAP5	VHL	CNOT9
CNTNAP5B	VIM	CNP
CNTRL	VIP	CNP1

CNTROB	VN1R17P	CNPPD1
COA1	VPS51	CNPY1
COA3	VPS9D1-AS1	CNPY2
COA4	VSIG1	CNPY3
COA5	VSIG4	CNPY4
COA6	VSIR	CNR1
COA7	VSNL1	CNR2
COA8	VSX1	CNRIP1
COASY	VTCN1	CNST
COBL	VTI1A	CNTD1
COBLL1	VTN	CNTF
COCH	VWCE	CNTFR
COG1	VWF	CNTFR-AS1
COG2	WDR1	CNTLN
COG3	WEE1	CNTN1
COG4	WIF1	CNTN2
COG5	WLS	CNTN3
COG6	WNK1	CNTN4
COG7	WNK2	CNTN5
COG8	WNT1	CNTN6
COIL	WNT2B	CNTNAP1
COL10A1	WNT3	CNTNAP2
COL11A1	WNT3A	CNTNAP3
COL11A2	WNT5A	CNTNAP3B
COL12A1	WNT7A	CNTNAP3P2
COL13A1	WRAP53	CNTNAP5
COL14A1	WSPAR	CNTNAP5B
COL15A1	WT1	CNTRL
COL16A1	WT1-AS	CNTROB
COL17A1	WTIP	COA1
COL18A1	WWC3	COA3
COL18A1-AS1	WWOX	COA4
COL19A1	WWTR1	COA5
COL1A1	XAB2	COA6
COL1A1A	XAF1	COA7
COL1A1B	XAGE1A	COA8

COL1A2	XAGE1B	COASY
COL20A1	XBP1	COBL
COL21A1	XDH	COBLL1
COL22A1	XIAP	COCH
COL23A1	XIST	COG1
COL24A1	XK	COG2
COL25A1	XKR6	COG3
COL26A1	XPA	COG4
COL27A1	XPC	COG5
COL28A1	XPO1	COG6
COL2A1	XPO5	COG7
COL2A1A	XPO6	COG8
COL2A1B	XPR1	COIL
COL3A1	XRCC1	COL10A1
COL4A1	XRCC2	COL11A1
COL4A2	XRCC3	COL11A2
COL4A3	XRCC4	COL12A1
COL4A4	XRCC5	COL13A1
COL4A5	XRCC6	COL14A1
COL4A6	XRCC6P5	COL15A1
COL5A1	XXYLT1	COL16A1
COL5A2	YAF2	COL17A1
COL5A2A	YAP1	COL18A1
COL5A3	YBX1	COL18A1-AS1
COL6A1	YEATS2	COL19A1
COL6A2	YEATS4	COL1A1
COL6A3	YES1	COL1A1A
COL6A4P2	YKT6	COL1A1B
COL6A5	YOD1	COL1A2
COL6A6	YPEL1	COL20A1
COL7A1	YPEL5	COL21A1
COL8A1	YTHDF1	COL22A1
COL8A2	YWHAG	COL23A1
COL9A1	YWHAZ	COL24A1
COL9A2	YY1	COL25A1
COL9A3	ZBED9	COL26A1

COLCA1	ZBTB16	COL27A1
COLEC10	ZBTB20	COL28A1
COLEC11	ZBTB5	COL2A1
COLEC12	ZBTB7A	COL2A1A
COLGALT1	ZCCHC9	COL2A1B
COLGALT2	ZDHHC5	COL3A1
COLQ	ZEB1	COL4A1
COLT	ZEB1-AS1	COL4A2
COMMD1	ZEB2	COL4A3
COMMD10	ZEB2-AS1	COL4A4
COMMD2	ZFAS1	COL4A5
COMMD3	ZFHX3	COL4A6
COMMD3-BMI1	ZFP36	COL5A1
COMMD4	ZFR	COL5A2
COMMD5	ZFX	COL5A2A
COMMD6	ZHX2	COL5A3
COMMD7	ZMYND10	COL6A1
COMMD8	ZNF124	COL6A2
COMMD9	ZNF197	COL6A3
COMP	ZNF205-AS1	COL6A4P2
COMT	ZNF264	COL6A5
COMT-4	ZNF326	COL6A6
COMTD1	ZNF331	COL7A1
COP1	ZNF367	COL8A1
COPA	ZNF395	COL8A2
COPB1	ZNF398	COL9A1
COPB2	ZNF462	COL9A2
COPB2-DT	ZNF503	COL9A3
COPE	ZNF654	COLCA1
COPG1	ZNF668	COLEC10
COPG2	ZNF677	COLEC11
COPG2IT1	ZNF703	COLEC12
COPRS	ZNF71	COLGALT1
COPS2	ZNF746	COLGALT2
COPS3	ZNRD2	COLQ
COPS4	ZNRF2	COLT

COPS5	ZPR1	COMETT
COPS6	ZSWIM5	COMMD1
COPS7A	ZYG11A	COMMD10
COPS7B	ZYX	COMMD2
COPS8		COMMD3
COPS9		COMMD3-BMI1
COPZ1		COMMD4
COPZ2		COMMD5
COQ10A		COMMD6
COQ10B		COMMD7
COQ2		COMMD8
COQ3		COMMD9
COQ4		COMP
COQ5		COMT
COQ6		COMT-4
COQ7		COMTD1
COQ8A		COP1
COQ8B		COPA
COQ9		COPB1
CORIN		COPB2
CORO1A		COPB2-DT
CORO1B		COPD
CORO1C		COPE
CORO2A		COPG1
CORO2B		COPG2
CORO6		COPG2IT1
CORO7		COPRS
CORT		COPS2
COSMOC		COPS3
COTL1		COPS4
COX1		COPS5
COX10		COPS6
COX10-DT		COPS7A
COX11		COPS7B
COX14		COPS8
COX15		COPS9

COX16	COPZ1
COX17	COPZ2
COX18	COQ10A
COX19	COQ10B
COX2	COQ2
COX20	COQ3
COX3	COQ4
COX4I1	COQ5
COX4I2	COQ6
COX5A	COQ7
COX5AA	COQ8A
COX5B	COQ8B
COX6A1	COQ9
COX6A2	CORIN
COX6B1	CORO1A
COX6B2	CORO1B
COX6C	CORO1C
COX6C-PS1	CORO2A
COX7A1	CORO2B
COX7A2	CORO6
COX7A2A	CORO7
COX7A2L	CORT
COX7B	COSMOC
COX7B2	COTL1
COX7BP1	COX1
COX7C	COX10
COX8A	COX10-DT
COX8B	COX11
COX8C	COX14
CP	COX15
CP110	COX16
CPA1	COX17
CPA2	COX18
CPA3	COX19
CPA4	COX2
CPA5	COX20

CPAMD8	COX3
CPB1	COX4I1
CPB2	COX4I2
CPD	COX5A
CPE	COX5AA
CPEB1	COX5B
CPEB2	COX6A1
CPEB3	COX6A2
CPEB4	COX6B1
CPED1	COX6B2
CPHX1	COX6C
CPLANE1	COX6C-PS1
CPLANE2	COX7A1
CPLX1	COX7A2
CPLX2	COX7A2A
CPLX3	COX7A2L
CPLX4	COX7B
CPM	COX7B2
CPN1	COX7BP1
CPN2	COX7C
CPNE1	COX8A
CPNE2	COX8B
CPNE3	COX8C
CPNE4	CP
CPNE5	CP110
CPNE6	CPA1
CPNE7	CPA2
CPNE8	CPA3
CPNE9	CPA4
CPO	CPA5
CPOX	CPAMD8
CPPED1	CPB1
CPQ	CPB2
CPR-4	CPD
CPR-5	CPE
CPR67B	CPEB1

CPS1
CPS1-IT1
CPSF1
CPSF2
CPSF3L
CPSF4
CPSF4L
CPSF6
CPSF7
CPT1
CPT1A
CPT1B
CPT1C
CPT2
CPTP
CPVL
CPXCR1
CPXM1
CPXM2
CPZ
CR1
CR1L
CR2
CRABP1
CRABP2
CRACD
CRACDL
CRACR2A
CRACR2B
CRADD
CRAMP1
CRAT
CRAT37
CRB1
CRB2
CRB3

CPEB2
CPEB3
CPEB4
CPED1
CPHX1
CPLANE1
CPLANE2
CPLX1
CPLX2
CPLX3
CPLX4
CPM
CPN1
CPN2
CPNE1
CPNE2
CPNE3
CPNE4
CPNE5
CPNE6
CPNE7
CPNE8
CPNE9
CPO
CPOX
CPPED1
CPQ
CPR-4
CPR-5
CPR67B
CPS1
CPS1-IT1
CPSF1
CPSF2
CPSF3
CPSF3L

CRBN	CPSF4
CRCP	CPSF4L
CRCT1	CPSF6
CREB1	CPSF7
CREB3	CPT1
CREB3L1	CPT1A
CREB3L2	CPT1B
CREB3L3	CPT1C
CREB3L4	CPT2
CREB5	CPTP
CREBBP	CPVL
CREBL2	CPXCR1
CREBRF	CPXM1
CREBZF	CPXM2
CREG1	CPZ
CREG2	CR1
CRELD1	CR1L
CRELD2	CR2
CREM	CRABP1
CRFB15	CRABP2
CRH	CRACD
CRHBP	CRACDL
CRHR1	CRACR2A
CRHR2	CRACR2B
CRIM1	CRADD
CRIP1	CRAMP1
CRIP2	CRAT
CRIP3	CRAT37
CRIPT	CRB1
CRISP2	CRB2
CRISP3	CRB3
CRISPLD1	CRBN
CRISPLD1A	CRCP
CRISPLD2	CRCT1
CRK	CREB1
CRKL	CREB3

CRLF1	CREB3L1
CRLF2	CREB3L2
CRLF3	CREB3L3
CRLS1	CREB3L4
CRMA	CREB5
CRMP	CREBBP
CRMP1	CREBL2
CRNDE	CREBRF
CRNKL1	CREBZF
CRNN	CREG1
CROCC	CREG2
CROCC2	CRELD1
CROCCP2	CRELD2
CROCCP3	CREM
CROP	CRFB15
CROT	CRH
CRP	CRHBP
CRP6	CRHR1
CRPPA	CRHR2
CRTAC1	CRIM1
CRTAM	CRIP1
CRTAP	CRIP2
CRTC1	CRIP3
CRTC2	CRIPT
CRTC3	CRISP2
CRX	CRISP3
CRX.S	CRISPLD1
CRXOS	CRISPLD1A
CRXOS1	CRISPLD2
CRY1	CRK
CRY2	CRKL
CRY4	CRLF1
CRYAA	CRLF2
CRYAB	CRLF3
CRYBA1	CRLS1
CRYBA2	CRMA

CRYBA2A	CRMP
CRYBA4	CRMP1
CRYBB1	CRNDE
CRYBB1L1	CRNKL1
CRYBB1L2	CRNN
CRYBB2	CROCC
CRYBB2P1	CROCC2
CRYBB3	CROCCP2
CRYBG1	CROCCP3
CRYBG2	CROP
CRYBG3	CROT
CRY-DASH	CRP
CRYGA	CRP6
CRYGB	CRPPA
CRYGC	CRTAC1
CRYGD	CRTAM
CRYGE	CRTAP
CRYGEP	CRTC1
CRYGF	CRTC2
CRYGMX	CRTC3
CRYGN	CRX
CRYGS	CRX.S
CRYL1	CRXOS
CRYM	CRXOS1
CRYS	CRY1
CRYZ	CRY2
CRYZL1	CRY4
CRYZL2P	CRYAA
CS	CRYAB
CSAD	CRYBA1
CSAG1	CRYBA2
CSAG2	CRYBA2A
CSDC2	CRYBA4
CSDE1	CRYBB1
CSE1L	CRYBB1L1
CSF1	CRYBB1L2

CSF1R	CRYBB2
CSF2	CRYBB2P1
CSF2RA	CRYBB3
CSF2RB	CRYBG1
CSF2RB2	CRYBG2
CSF3	CRYBG3
CSF3R	CRY-DASH
CSGALNACT1	CRYGA
CSGALNACT2	CRYGB
CSH1	CRYGC
CSH2	CRYGD
CSHL1	CRYGE
CSK	CRYGEP
CSKMT	CRYGF
CSMD1	CRYGMX
CSMD2	CRYGN
CSMD3	CRYGS
CSN1S1	CRYL1
CSN1S2AP	CRYM
CSN1S2B	CRYS
CSN2	CRYZ
CSNK1A1	CRYZL1
CSNK1A1P1	CRYZL2P
CSNK1D	CS
CSNK1E	CSAD
CSNK1G1	CSAG1
CSNK1G2	CSAG2
CSNK1G2-AS1	CSDC2
CSNK1G3	CSDE1
CSNK2A1	CSE1L
CSNK2A2	CSF1
CSNK2A3	CSF1R
CSNK2B	CSF2
CSNKA2IP	CSF2RA
CSPG4	CSF2RB
CSPG4B	CSF2RB2

CSPG4P1Y	CSF3
CSPG4P2Y	CSF3R
CSPG5	CSGALNACT1
CSPG5B	CSGALNACT2
CSPP1	CSH1
CSPRS	CSH2
CSRNP1	CSHL1
CSRNP2	CSK
CSRNP3	CSKMT
CSRP1	CSMD1
CSRP2	CSMD2
CSRP3	CSMD3
CST1	CSN1S1
CST10	CSN1S2AP
CST11	CSN1S2B
CST2	CSN2
CST3	CSNK1A1
CST4	CSNK1A1P1
CST5	CSNK1D
CST6	CSNK1E
CST7	CSNK1G1
CST8	CSNK1G2
CST9L	CSNK1G2-AS1
CSTA	CSNK1G3
CSTB	CSNK2A1
CSTF1	CSNK2A2
CSTF2	CSNK2A3
CSTF2T	CSNK2B
CSTF3	CSNKA2IP
CSTL1	CSPG4
CSTPP1	CSPG4B
CT45A1	CSPG4P1Y
CT45A2	CSPG4P2Y
CT45A3	CSPG5
CT45A5	CSPG5B
CT45A6	CSPP1

CT47B1	CSPRS
CT55	CSRNP1
CT62	CSRNP2
CTAG1A	CSRNP3
CTAG1B	CSRP1
CTAG2	CSRP2
CTAGE4	CSRP3
CTAGE6	CST1
CTAGE7P	CST10
CTBP1	CST11
CTBP1-DT	CST2
CTBP2	CST3
CTBS	CST4
CTC1	CST5
CTCF	CST6
CTCFL	CST7
CTCFLOS	CST8
CTDNEP1	CST9L
CTDP1	CSTA
CTDSP1	CSTB
CTDSP2	CSTF1
CTDSPL	CSTF2
CTDSPL2	CSTF2T
CTDSPLB	CSTF3
CTF1	CSTL1
CTGF	CSTPP1
CTH	CT45A1
CTHRC1	CT45A2
CTIF	CT45A3
CTLA2A	CT45A5
CTLA2B	CT45A6
CTLA4	CT47B1
CTNNA1	CT55
CTNNA2	CT62
CTNNAL1	CT83
CTNNB1	CTAG1A

CTNNBIP1	CTAG1B
CTNNBL1	CTAG2
CTNND1	CTAGE4
CTNND2	CTAGE6
CTNS	CTAGE7P
CTPS	CTBP1
CTPS1	CTBP1-DT
CTPS2	CTBP2
CTR9	CTBS
CTRB1	CTC1
CTRB2	CTCF
CTRC	CTCFL
CTRL	CTCFLOS
CTSA	CTDNEP1
CTSB	CTDP1
CTSBB	CTDSP1
CTSC	CTDSP2
CTSD	CTDSPL
CTSE	CTDSPL2
CTSF	CTDSPLB
CTSG	CTF1
CTSH	CTGF
CTSJ	CTH
CTSK	CTHRC1
CTSL	CTIF
CTSL.1	CTLA2A
CTSLL3	CTLA2B
CTSLP8	CTLA4
CTSM	CTNNA1
CTSO	CTNNA2
CTSQ	CTNNAL1
CTSS	CTNNB1
CTSV	CTNNBIP1
CTSW	CTNNBL1
CTSZ	CTNND1
CTTN	CTNND2

CTTNBP2	CTNS
CTTNBP2NL	CTPS
CTU1	CTPS1
CTU2	CTPS2
CTXN1	CTR9
CTXN3	CTRB1
CTXND1	CTRB2
CUBN	CTRC
CUEDC1	CTRL
CUEDC2	CTSA
CUL1	CTSB
CUL2	CTSBB
CUL3	CTSC
CUL4A	CTSD
CUL4B	CTSE
CUL5	CTSF
CUL7	CTSG
CUL9	CTSH
CUTA	CTSJ
CUTALP	CTSK
CUTC	CTSL
CUX1	CTSL.1
CUX2	CTSLL3
CUZD1	CTSLP8
CWC15	CTSM
CWC22	CTSO
CWC25	CTSQ
CWC27	CTSS
CWF19L1	CTSV
CWF19L2	CTSW
CWH43	CTSZ
CX3CL1	CTTN
CX3CR1	CTTNBP2
CXADR	CTTNBP2NL
CXCL1	CTU1
CXCL10	CTU2

CXCL11	CTXN1
CXCL12	CTXN3
CXCL12A	CTXND1
CXCL13	CUBN
CXCL14	CUEDC1
CXCL15	CUEDC2
CXCL16	CUL1
CXCL17	CUL2
CXCL2	CUL3
CXCL3	CUL4A
CXCL5	CUL4B
CXCL6	CUL5
CXCL8	CUL7
CXCL9	CUL9
CXCR1	CUTA
CXCR2	CUTALP
CXCR2P1	CUTC
CXCR3	CUX1
CXCR4	CUX2
CXCR5	CUZD1
CXCR6	CWC15
CXCR7	CWC22
CXORF38	CWC25
CXORF49	CWC27
CXORF49B	CWF19L1
CXORF51A	CWF19L2
CXORF58	CWH43
CXXC1	CX3CL1
CXXC4	CX3CR1
CXXC5	CXADR
CYAT1	CXADRP1
CYB561	CXCL1
CYB561A3	CXCL10
CYB561D1	CXCL11
CYB561D2	CXCL12
CYB5A	CXCL12A

CYB5B	CXCL13
CYB5D1	CXCL14
CYB5D2	CXCL15
CYB5R1	CXCL16
CYB5R2	CXCL17
CYB5R3	CXCL2
CYB5R4	CXCL3
CYB5RL	CXCL5
CYBA	CXCL6
CYBASC3	CXCL8
CYBB	CXCL9
CYBC1	CXCR1
CYBRD1	CXCR2
CYC1	CXCR2P1
CYCE	CXCR3
CYCL	CXCR4
CYCS	CXCR5
CYCSB	CXCR6
CYFIP1	CXCR7
CYFIP2	CXORF38
CYGB	CXORF49
CYGB1	CXORF49B
CYHR1	CXORF51A
CYLC1	CXORF58
CYLD	CXXC1
CYM	CXXC4
CYORF15A	CXXC5
CYP11A1	CYAT1
CYP11B1	CYB561
CYP11B2	CYB561A3
CYP-13A6	CYB561D1
CYP17A1	CYB561D2
CYP19A1	CYB5A
CYP19A1A	CYB5B
CYP19A1B	CYB5D1
CYP1A	CYB5D2

CYP1A1	CYB5R1
CYP1A2	CYB5R2
CYP1B1	CYB5R3
CYP1B1-AS1	CYB5R4
CYP1C1	CYB5RL
CYP1C2	CYBA
CYP20A1	CYBASC3
CYP21A2	CYBB
CYP24A1	CYBC1
CYP26A1	CYBRD1
CYP26B1	CYC1
CYP26C1	CYCE
CYP27A1	CYCL
CYP27B1	CYCS
CYP27C1	CYCSB
CYP28D1	CYFIP1
CYP2A1	CYFIP2
CYP2A12	CYGB
CYP2A13	CYGB1
CYP2A2	CYHR1
CYP2A3	CYLC1
CYP2A4	CYLD
CYP2A5	CYM
CYP2A6	CYORF15A
CYP2A7	CYP11A1
CYP2AA11	CYP11B1
CYP2AA12	CYP11B2
CYP2AA3	CYP-13A6
CYP2AE1	CYP17A1
CYP2B1	CYP19A1
CYP2B10	CYP19A1A
CYP2B12	CYP19A1B
CYP2B13	CYP1A
CYP2B15	CYP1A1
CYP2B19	CYP1A2
CYP2B2	CYP1B1

CYP2B3	CYP1B1-AS1
CYP2B6	CYP1C1
CYP2B7P	CYP1C2
CYP2B9	CYP20A1
CYP2C	CYP21A2
CYP2C1	CYP24A1
CYP2C11	CYP26A1
CYP2C12	CYP26B1
CYP2C13	CYP26C1
CYP2C18	CYP27A1
CYP2C19	CYP27B1
CYP2C23	CYP27C1
CYP2C29	CYP28D1
CYP2C37	CYP2A1
CYP2C38	CYP2A12
CYP2C39	CYP2A13
CYP2C40	CYP2A2
CYP2C50	CYP2A3
CYP2C54	CYP2A4
CYP2C55	CYP2A5
CYP2C65	CYP2A6
CYP2C66	CYP2A7
CYP2C68	CYP2AA11
CYP2C6V1	CYP2AA12
CYP2C7	CYP2AA3
CYP2C70	CYP2AE1
CYP2C8	CYP2B1
CYP2C9	CYP2B10
CYP2C93	CYP2B12
CYP2D1	CYP2B13
CYP2D10	CYP2B15
CYP2D11	CYP2B19
CYP2D12	CYP2B2
CYP2D22	CYP2B3
CYP2D26	CYP2B6
CYP2D3	CYP2B7P

CYP2D4	CYP2B9
CYP2D6	CYP2C
CYP2D7	CYP2C1
CYP2D9	CYP2C11
CYP2E1	CYP2C12
CYP2F1	CYP2C13
CYP2F2	CYP2C18
CYP2G1	CYP2C19
CYP2J11	CYP2C23
CYP2J13	CYP2C29
CYP2J2	CYP2C37
CYP2J3	CYP2C38
CYP2J4	CYP2C39
CYP2J5	CYP2C40
CYP2J9	CYP2C50
CYP2K18	CYP2C54
CYP2K19	CYP2C55
CYP2K20	CYP2C65
CYP2K21	CYP2C66
CYP2R1	CYP2C68
CYP2S1	CYP2C6V1
CYP2T1	CYP2C7
CYP2T1P	CYP2C70
CYP2U1	CYP2C8
CYP2W1	CYP2C9
CYP313A1	CYP2C93
CYP-35C1	CYP2D1
CYP39A1	CYP2D10
CYP3A11	CYP2D11
CYP3A13	CYP2D12
CYP3A18	CYP2D22
CYP3A2	CYP2D26
CYP3A23-3A1	CYP2D3
CYP3A25	CYP2D4
CYP3A4	CYP2D6
CYP3A41A	CYP2D7

CYP3A43	CYP2D9
CYP3A44	CYP2E1
CYP3A5	CYP2F1
CYP3A62	CYP2F2
CYP3A65	CYP2G1
CYP3A7	CYP2J11
CYP3A9	CYP2J13
CYP46A1	CYP2J2
CYP4A1	CYP2J3
CYP4A10	CYP2J4
CYP4A11	CYP2J5
CYP4A12A	CYP2J9
CYP4A14	CYP2K18
CYP4A2	CYP2K19
CYP4A22	CYP2K20
CYP4A3	CYP2K21
CYP4A31	CYP2R1
CYP4A32	CYP2S1
CYP4A8	CYP2T1
CYP4AC1	CYP2T1P
CYP4B1	CYP2U1
CYP4D21	CYP2W1
CYP4E2	CYP313A1
CYP4F1	CYP-35C1
CYP4F11	CYP39A1
CYP4F12	CYP3A11
CYP4F15	CYP3A13
CYP4F16	CYP3A18
CYP4F18	CYP3A2
CYP4F2	CYP3A23-3A1
CYP4F22	CYP3A25
CYP4F29P	CYP3A4
CYP4F3	CYP3A41A
CYP4F30P	CYP3A43
CYP4F35P	CYP3A44
CYP4F37	CYP3A5

CYP4F39	CYP3A51P
CYP4F40	CYP3A62
CYP4F6	CYP3A65
CYP4F8	CYP3A7
CYP4V2	CYP3A9
CYP4V3	CYP46A1
CYP4V7	CYP4A1
CYP4X1	CYP4A10
CYP4Z1	CYP4A11
CYP4Z2P	CYP4A12A
CYP51	CYP4A14
CYP51A1	CYP4A2
CYP51A1P2	CYP4A22
CYP6A17	CYP4A3
CYP6A23	CYP4A31
CYP6G1	CYP4A32
CYP7A1	CYP4A8
CYP7B1	CYP4AC1
CYP8B1	CYP4B1
CYP9F2	CYP4D21
CYPT1	CYP4E2
CYR61	CYP4F1
CYREN	CYP4F11
CYRIA	CYP4F12
CYRIB	CYP4F15
CYS1	CYP4F16
CYSLTR1	CYP4F18
CYSLTR2	CYP4F2
CYSRT1	CYP4F22
CYSTM1	CYP4F29P
CYT1	CYP4F3
CYTB	CYP4F30P
CYT-B5-R	CYP4F35P
CYTH1	CYP4F37
CYTH2	CYP4F39
CYTH3	CYP4F40

CYTH4	CYP4F6
CYTIP	CYP4F8
CYTL1	CYP4V2
CYTOR	CYP4V3
CYYR1	CYP4V7
CZIB	CYP4X1
D17H6S56E-5	CYP4Z1
D1ERTD692	CYP4Z2P
D2ERTD127E	CYP51
D2HGDH	CYP51A1
D3ERTD751E	CYP51A1P2
D4ERTD298E	CYP6A17
D5ERTD579E	CYP6A23
D6ERTD474E	CYP6G1
D6ERTD527E	CYP7A1
D6WSU163E	CYP7B1
DAAM1	CYP8B1
DAAM1B	CYP9F2
DAAM2	CYPT1
DAB1	CYR61
DAB2	CYREN
DAB2IP	CYRIA
DACH1	CYRIB
DACH2	CYS1
DACT1	CYSLTR1
DACT2	CYSLTR2
DACT3	CYSRT1
DACT3-AS1	CYSTM1
DAD1	CYT1
DAF-16	CYTB
DAG1	CYT-B5-R
DAGLA	CYTH1
DAGLB	CYTH2
DAK	CYTH3
DALRD3	CYTH4
DANCR	CYTIP

DAND5	CYTL1
DAO	CYTOR
DAO.1	CYYR1
DAOA-AS1	CZIB
DAP	D17H6S56E-5
DAP3	D1ERTD692
DAPK1	D2ERTD127E
DAPK2	D2HGDH
DAPK3	D3ERTD751E
DAPL1	D4ERTD298E
DAPP1	D5ERTD579E
DARC	D6ERTD474E
DARMIN.L	D6ERTD527E
DARS	D6WSU163E
DARS1	DAAM1
DARS2	DAAM1B
DAW1	DAAM2
DAXX	DAB1
DAZ2	DAB2
DAZ3	DAB2IP
DAZ4	DACH1
DAZAP1	DACH2
DAZAP2	DACT1
DAZL	DACT2
DBF4	DACT3
DBF4B	DACT3-AS1
DBH	DAD1
DBH-AS1	DAF-16
DBI	DAG1
DBI.S	DAGLA
DBIL5P	DAGLB
DBN1	DAK
DBNDD1	DALRD3
DBNDD2	DANCR
DBNL	DAND5
DBNLB	DAO

DBP	DAO.1
DBR1	DAOA-AS1
DBT	DAP
DBT-1	DAP3
DBX2	DAPK1
DCAF1	DAPK2
DCAF10	DAPK3
DCAF11	DAPL1
DCAF12	DAPP1
DCAF12L1	DARC
DCAF12L2	DARMIN.L
DCAF13	DARS
DCAF15	DARS1
DCAF16	DARS2
DCAF17	DAW1
DCAF4	DAXX
DCAF4L1	DAZ2
DCAF5	DAZ3
DCAF6	DAZ4
DCAF7	DAZAP1
DCAF8	DAZAP2
DCAF8L1	DAZL
DCAF8L2	DBF4
DCAKD	DBF4B
DCANP1	DBH
DCBLD1	DBH-AS1
DCBLD2	DBI
DCC	DBI.S
DCD	DBIL5P
DCDC1	DBN1
DCDC2	DBNDD1
DCDC2A	DBNDD2
DCDC2B	DBNL
DCHS1	DBNLB
DCK	DBP
DCLK1	DBR1

DCLK2	DBT
DCLK3	DBT-1
DCLRE1A	DBX2
DCLRE1B	DCAF1
DCLRE1C	DCAF10
DCN	DCAF11
DCP1A	DCAF12
DCP1B	DCAF12L1
DCP2	DCAF12L2
DCPP1	DCAF13
DCPP2	DCAF15
DCPP3	DCAF16
DCPS	DCAF17
DCST1	DCAF4
DCST1-AS1	DCAF4L1
DCST2	DCAF5
DCSTAMP	DCAF6
DCT	DCAF7
DCTD	DCAF8
DCTN1	DCAF8L1
DCTN1A	DCAF8L2
DCTN1-AS1	DCAKD
DCTN2	DCANP1
DCTN3	DCBLD1
DCTN4	DCBLD2
DCTN5	DCC
DCTN6	DCD
DCTPP1	DCDC1
DCUN1D1	DCDC2
DCUN1D2	DCDC2A
DCUN1D2A	DCDC2B
DCUN1D3	DCHS1
DCUN1D4	DCK
DCUN1D5	DCLK1
DCX	DCLK2
DCXR	DCLK3

DDA1	DCLRE1A
DDAH1	DCLRE1B
DDAH2	DCLRE1C
DDB1	DCN
DDB2	DCP1A
DDC	DCP1B
DDEFL1	DCP2
DDHD1	DCPP1
DDHD2	DCPP2
DDI2	DCPP3
DDIAS	DCPS
DDIT3	DCR
DDIT4	DCST1
DDIT4L	DCST1-AS1
DDN	DCST2
DDO	DCSTAMP
DDOST	DCT
DDR1	DCTD
DDR2	DCTN1
DDRGK1	DCTN1A
DDT	DCTN1-AS1
DDTL	DCTN2
DDX1	DCTN3
DDX10	DCTN4
DDX11	DCTN5
DDX11L2	DCTN6
DDX12P	DCTPP1
DDX17	DCUN1D1
DDX18	DCUN1D2
DDX19A	DCUN1D2A
DDX19B	DCUN1D3
DDX20	DCUN1D4
DDX21	DCUN1D5
DDX23	DCX
DDX24	DCXR
DDX25	DDA1

DDX27	DDAH1
DDX28	DDAH2
DDX31	DDB1
DDX39A	DDB2
DDX39B	DDC
DDX3X	DDEFL1
DDX3Y	DDH2
DDX4	DDHD1
DDX42	DDHD2
DDX43	DDI2
DDX46	DDIAS
DDX47	DDIT3
DDX49	DDIT4
DDX5	DDIT4L
DDX50	DDN
DDX51	DDO
DDX52	DDOST
DDX53	DDR1
DDX54	DDR2
DDX55	DDRGK1
DDX56	DDT
DDX58	DDTL
DDX59	DDX1
DDX59-AS1	DDX10
DDX6	DDX11
DDX60	DDX11L2
DDX60L	DDX12P
DEAF1	DDX17
DECAY	DDX18
DECR1	DDX19A
DECR2	DDX19B
DEDD	DDX20
DEDD2	DDX21
DEF	DDX23
DEF6	DDX24
DEF8	DDX25

DEFA1	DDX27
DEFA10P	DDX28
DEFA1B	DDX31
DEFA29	DDX39A
DEFA3	DDX39B
DEFA4	DDX3X
DEFA5	DDX3Y
DEFB1	DDX4
DEFB103A	DDX42
DEFB103B	DDX43
DEFB108B	DDX46
DEFB109A	DDX47
DEFB109B	DDX49
DEFB113	DDX5
DEFB114	DDX50
DEFB115	DDX51
DEFB123	DDX52
DEFB124	DDX53
DEFB125	DDX54
DEFB129	DDX55
DEFB13	DDX56
DEFB132	DDX58
DEFB136	DDX59
DEFB15	DDX59-AS1
DEFB24	DDX6
DEFB3	DDX60
DEFB4	DDX60L
DEFB42	DEAF1
DEFB4A	DECAY
DEFB5	DECR1
DEFB9	DECR2
DEGS1	DEDD
DEGS2	DEDD2
DEK	DEF
DELE1	DEF6
DELEC1	DEF8

DENN2B	DEFA1
DENND10	DEFA10P
DENND10P1	DEFA1B
DENND11	DEFA29
DENND1A	DEFA3
DENND1B	DEFA4
DENND1C	DEFA5
DENND2A	DEFB1
DENND2B	DEFB103A
DENND2C	DEFB103B
DENND2D	DEFB108B
DENND3	DEFB109A
DENND4A	DEFB109B
DENND4B	DEFB113
DENND4C	DEFB114
DENND5A	DEFB115
DENND5B	DEFB123
DENND6A	DEFB124
DENND6A-DT	DEFB125
DENND6B	DEFB129
DENR	DEFB13
DEPDC1	DEFB132
DEPDC1B	DEFB136
DEPDC4	DEFB15
DEPDC5	DEFB24
DEPDC7	DEFB3
DEPDC7.S	DEFB4
DEPP1	DEFB42
DEPTOR	DEFB4A
DER	DEFB5
DERA	DEFB9
DERL1	DEGS1
DERL2	DEGS2
DERL3	DEK
DES	DELE1
DESI1	DELEC1

DESI1A	DENN2B
DESI2	DENND10
DESMA	DENND10P1
DET1	DENND11
DEUP1	DENND1A
DEXI	DENND1B
DFFA	DENND1C
DFFB	DENND2A
DGAT1	DENND2B
DGAT2	DENND2C
DGAT2L6	DENND2D
DGCR2	DENND3
DGCR5	DENND4A
DGCR6	DENND4B
DGCR6L	DENND4C
DGCR8	DENND5A
DGKA	DENND5B
DGKB	DENND6A
DGKD	DENND6A-DT
DGKE	DENND6B
DGKG	DENR
DGKH	DEPDC1
DGKI	DEPDC1B
DGKK	DEPDC4
DGKQ	DEPDC5
DGKZ	DEPDC7
DGLUCY	DEPDC7.S
DGUOK	DEPP1
DHCR24	DEPTOR
DHCR7	DER
DHDDS	DERA
DHDH	DERL1
DHDH.1	DERL2
DHFR	DERL3
DHFR2	DES
DHH	DESI1

DHODH	DESI1A
DHPS	DESI2
DHRS1	DESMA
DHRS11	DET1
DHRS12	DEUP1
DHRS13	DEXI
DHRS2	DFFA
DHRS3	DFFB
DHRS3B	DGAT1
DHRS4	DGAT2
DHRS4-AS1	DGAT2L6
DHRS4L1	DGCR2
DHRS4L2	DGCR5
DHRS7	DGCR6
DHRS7B	DGCR6L
DHRS7C	DGCR8
DHRS9	DGKA
DHRSX	DGKB
DHTKD1	DGKD
DHX15	DGKE
DHX16	DGKG
DHX29	DGKH
DHX30	DGKI
DHX32	DGKK
DHX32A	DGKQ
DHX33	DGKZ
DHX34	DGLUCY
DHX35	DGUOK
DHX36	DHCR24
DHX37	DHCR7
DHX38	DHDDS
DHX40	DHDH
DHX57	DHDH.1
DHX58	DHFR
DHX8	DHFR2
DHX9	DHH

DIABLO	DHODH
DIAPH1	DHPS
DIAPH2	DHRS1
DIAPH2-AS1	DHRS11
DIAPH3	DHRS12
DICER1	DHRS13
DICER1-AS1	DHRS2
DIDO1	DHRS3
DIMT1	DHRS3B
DIMT1L	DHRS4
DIO1	DHRS4-AS1
DIO2	DHRS4L1
DIO3	DHRS4L2
DIO3OS	DHRS7
DIP2A	DHRS7B
DIP2B	DHRS7C
DIP2C	DHRS9
DIP2C-AS1	DHRSX
DIPA	DHTKD1
DIPK1A	DHX15
DIPK1B	DHX16
DIPK1C	DHX29
DIPK2A	DHX30
DIPK2B	DHX32
DIRAS1	DHX32A
DIRAS2	DHX33
DIRAS3	DHX34
DIRC1	DHX35
DIRC3	DHX36
DIS3	DHX37
DIS3L	DHX38
DIS3L2	DHX40
DISC1	DHX57
DISP1	DHX58
DISP2	DHX8
DISP3	DHX9

DIXDC1	DIABLO
DKC1	DIAPH1
DKK1	DIAPH2
DKK2	DIAPH2-AS1
DKK3	DIAPH3
DKK4	DICER1
DKKL1	DICER1-AS1
DLA	DIDO1
DLAT	DIMT1
DLC1	DIMT1L
DLD	DIO1
DLEC1	DIO2
DLEU1	DIO3
DLEU2	DIO3OS
DLEU2L	DIP2A
DLEU7	DIP2B
DLG1	DIP2C
DLG2	DIP2C-AS1
DLG3	DIPA
DLG4	DIPK1A
DLG5	DIPK1B
DLG5-AS1	DIPK1C
DLGAP1	DIPK2A
DLGAP1-AS1	DIPK2B
DLGAP1-AS2	DIRAS1
DLGAP2	DIRAS2
DLGAP3	DIRAS3
DLGAP4	DIRC1
DLGAP5	DIRC3
DLK1	DIS3
DLK2	DIS3L
DLL1	DIS3L2
DLL3	DISC1
DLL4	DISP1
DLST	DISP2
DLSTP1	DISP3

DLX1	DIXDC1
DLX2	DKC1
DLX3	DKK1
DLX4	DKK2
DLX5	DKK3
DLX6	DKK4
DLX6-AS1	DKKL1
DM1-AS	DLA
DMAC1	DLAT
DMAC2	DLC1
DMAC2L	DLD
DMAP1	DLEC1
DMBT1	DLEU1
DMBT1L1	DLEU2
DMBX1	DLEU2L
DMC1	DLEU7
DMD	DLG1
DMGDH	DLG2
DMKN	DLG3
DMP1	DLG4
DMPK	DLG5
DMRT1	DLG5-AS1
DMRT2	DLGAP1
DMRT3	DLGAP1-AS1
DMRTA1	DLGAP1-AS2
DMRTA2	DLGAP2
DMRTB1	DLGAP3
DMRTC1	DLGAP4
DMRTC1B	DLGAP5
DMRTC2	DLK1
DMTF1	DLK2
DMTN	DLL1
DMWD	DLL3
DMXL1	DLL4
DMXL2	DLST
DNA2	DLSTP1

DNAAF1	DLX1
DNAAF10	DLX2
DNAAF11	DLX3
DNAAF2	DLX4
DNAAF3	DLX5
DNAAF4	DLX6
DNAAF5	DLX6-AS1
DNAAF6	DM1-AS
DNAAF8	DMAC1
DNAAF9	DMAC2
DNAH1	DMAC2L
DNAH10	DMAP1
DNAH11	DMBT1
DNAH12	DMBT1L1
DNAH14	DMBX1
DNAH17	DMC1
DNAH17-AS1	DMD
DNAH2	DMGDH
DNAH3	DMKN
DNAH5	DMP1
DNAH6	DMPK
DNAH7	DMRT1
DNAH8	DMRT2
DNAH9	DMRT3
DNAI1	DMRTA1
DNAI2	DMRTA2
DNAI3	DMRTB1
DNAI4	DMRTC1
DNAI7	DMRTC1B
DNAJA	DMRTC2
DNAJA1	DMTF1
DNAJA1P5	DMTN
DNAJA2	DMWD
DNAJA3	DMXL1
DNAJA4	DMXL2
DNAJB1	DNA2

DNAJB11	DNAAF1
DNAJB12	DNAAF10
DNAJB13	DNAAF11
DNAJB14	DNAAF2
DNAJB1A	DNAAF3
DNAJB1B	DNAAF4
DNAJB2	DNAAF5
DNAJB3	DNAAF6
DNAJB4	DNAAF8
DNAJB5	DNAAF9
DNAJB6	DNAH1
DNAJB6A	DNAH10
DNAJB7	DNAH11
DNAJB8	DNAH12
DNAJB9	DNAH14
DNAJC1	DNAH17
DNAJC10	DNAH17-AS1
DNAJC11	DNAH2
DNAJC12	DNAH3
DNAJC13	DNAH5
DNAJC14	DNAH6
DNAJC15	DNAH7
DNAJC16	DNAH8
DNAJC17	DNAH9
DNAJC18	DNAI1
DNAJC19	DNAI2
DNAJC2	DNAI3
DNAJC21	DNAI4
DNAJC22	DNAI7
DNAJC24	DNAJA
DNAJC25	DNAJA1
DNAJC25-GNG10	DNAJA1P5
DNAJC27	DNAJA2
DNAJC28	DNAJA3
DNAJC3	DNAJA4
DNAJC30	DNAJB1

DNAJC3-DT	DNAJB11
DNAJC4	DNAJB12
DNAJC5	DNAJB13
DNAJC5G	DNAJB14
DNAJC6	DNAJB1A
DNAJC7	DNAJB1B
DNAJC8	DNAJB2
DNAJC9	DNAJB3
DNAJC9-AS1	DNAJB4
DNAL1	DNAJB5
DNAL4	DNAJB6
DNALI1	DNAJB6A
DNASE1	DNAJB7
DNASE1L1	DNAJB8
DNASE1L2	DNAJB9
DNASE1L3	DNAJC1
DNASE2	DNAJC10
DNASE2A	DNAJC11
DNASE2B	DNAJC12
DND1	DNAJC13
DNER	DNAJC14
DNHD1	DNAJC15
DNLZ	DNAJC16
DNM1	DNAJC17
DNM1L	DNAJC18
DNM2	DNAJC19
DNM3	DNAJC2
DNM3OS	DNAJC21
DNMBP	DNAJC22
DNMT1	DNAJC24
DNMT2	DNAJC25
DNMT3A	DNAJC25-GNG10
DNMT3AOS	DNAJC27
DNMT3B	DNAJC28
DNMT3L	DNAJC3
DNPEP	DNAJC30

DNPH1	DNAJC3-DT
DNTTIP1	DNAJC4
DNTTIP2	DNAJC5
DOC2A	DNAJC5G
DOC2B	DNAJC6
DOCK1	DNAJC7
DOCK10	DNAJC8
DOCK11	DNAJC9
DOCK2	DNAJC9-AS1
DOCK3	DNAL1
DOCK4	DNAL4
DOCK5	DNALI1
DOCK6	DNASE1
DOCK7	DNASE1L1
DOCK8	DNASE1L2
DOCK8-AS1	DNASE1L3
DOCK9	DNASE2
DOD-17	DNASE2A
DOD-24	DNASE2B
DOHH	DND1
DOK1	DNER
DOK2	DNHD1
DOK3	DNLZ
DOK4	DNM1
DOK5	DNM1L
DOK6	DNM1P50
DOK7	DNM2
DOLK	DNM3
DOLPP1	DNM3OS
DONSON	DNMBP
DOP1A	DNMT1
DOP1B	DNMT2
DOT1L	DNMT3A
DP1	DNMT3AOS
DPAGT1	DNMT3B
DPCD	DNMT3L

DPEP1	DNPEP
DPEP2	DNPH1
DPEP3	DNTT
DPF1	DNTTIP1
DPF2	DNTTIP2
DPF3	DOC2A
DPH1	DOC2B
DPH2	DOCK1
DPH3	DOCK10
DPH5	DOCK11
DPH6	DOCK2
DPH7	DOCK3
DPM1	DOCK4
DPM2	DOCK5
DPM3	DOCK6
DPP10	DOCK7
DPP10-AS1	DOCK8
DPP3	DOCK8-AS1
DPP4	DOCK9
DPP6	DOD-17
DPP7	DOD-24
DPP8	DOHH
DPP9	DOK1
DPP9-AS1	DOK2
DPPA2	DOK3
DPPA3	DOK4
DPPA3P2	DOK5
DPPA4	DOK6
DPPA5	DOK7
DPT	DOLK
DPY19L1	DOLPP1
DPY19L1P1	DONSON
DPY19L2	DOP1A
DPY19L2P2	DOP1B
DPY19L2P3	DOT1L
DPY19L3	DP1

DPY19L4	DPAGT1
DPY30	DPCD
DPYD	DPEP1
DPYS	DPEP2
DPYSL2	DPEP3
DPYSL3	DPF1
DPYSL4	DPF2
DPYSL5	DPF3
DQX1	DPH1
DR1	DPH2
DRAIC	DPH3
DRAM1	DPH5
DRAM2	DPH6
DRAP1	DPH7
DRAXIN	DPM1
DRC1	DPM2
DRC3	DPM3
DRC7	DPP10
DRD1	DPP10-AS1
DRD2	DPP3
DRD3	DPP4
DRD4	DPP6
DRD5	DPP7
DRD-50	DPP8
DRG1	DPP9
DRICH1	DPP9-AS1
DRL	DPPA2
DRO	DPPA3
DROSHA	DPPA3P2
DRP2	DPPA4
DRS	DPPA5
DSC1	DPT
DSC2	DPY19L1
DSC3	DPY19L1P1
DSCAM	DPY19L2
DSCAM-AS1	DPY19L2P2

DSCAML1	DPY19L2P3
DSCC1	DPY19L3
DSCR10	DPY19L4
DSCR8	DPY30
DSE	DPYD
DSEL	DPYS
DSG1	DPYSL2
DSG2	DPYSL3
DSG2-AS1	DPYSL4
DSG3	DPYSL5
DSG4	DQX1
DSN1	DR1
DSP	DRAIC
DSPP	DRAM1
DST	DRAM2
DSTN	DRAP1
DSTNP2	DRAXIN
DSTYK	DRC1
DTD1	DRC3
DTD2	DRC7
DTHD1	DRD1
DTL	DRD2
DTNA	DRD3
DTNB	DRD4
DTNBP1	DRD5
DTR	DRD-50
DTWD1	DRG1
DTWD2	DRICH1
DTX1	DRL
DTX2	DRO
DTX2P1-UPK3BP1-PMS2P11	DROSHA
DTX3	DRP2
DTX3L	DRS
DTX4	DSC1
DTYMK	DSC2
DUBR	DSC3

DULLARD	DSCAM
DUOX1	DSCAM-AS1
DUOX2	DSCAML1
DUOXA1	DSCC1
DUOXA2	DSCR10
DUS1L	DSCR8
DUS2	DSE
DUS3L	DSEL
DUS4L	DSG1
DUSP1	DSG2
DUSP10	DSG2-AS1
DUSP11	DSG3
DUSP12	DSG4
DUSP13	DSN1
DUSP14	DSP
DUSP15	DSPP
DUSP16	DST
DUSP18	DSTN
DUSP19	DSTNP2
DUSP2	DSTYK
DUSP21	DTD1
DUSP22	DTD2
DUSP22B	DTHD1
DUSP23	DTL
DUSP26	DTNA
DUSP27	DTNB
DUSP28	DTNBP1
DUSP3	DTR
DUSP4	DTWD1
DUSP5	DTWD2
DUSP5P1	DTX1
DUSP6	DTX2
DUSP7	DTX2P1-UPK3BP1-PMS2P11
DUSP8	DTX3
DUSP9	DTX3L
DUT	DTX4

DUX4	DTYMK
DUXA	DUBR
DUXBL1	DULLARD
DVL1	DUOX1
DVL2	DUOX2
DVL3	DUOXA1
DXO	DUOXA2
DYDC1	DUS1L
DYDC2	DUS2
DYM	DUS3L
DYNAP	DUS4L
DYNC1H1	DUSP1
DYNC1I1	DUSP10
DYNC1I2	DUSP11
DYNC1LI1	DUSP12
DYNC1LI2	DUSP13
DYNC2H1	DUSP14
DYNC2I1	DUSP15
DYNC2I2	DUSP16
DYNC2LI1	DUSP18
DYNLL1	DUSP19
DYNLL2	DUSP2
DYNLRB1	DUSP21
DYNLRB2	DUSP22
DYNLT1	DUSP22B
DYNLT1F	DUSP23
DYNLT2	DUSP26
DYNLT2A1	DUSP27
DYNLT2B	DUSP28
DYNLT3	DUSP3
DYNLT4	DUSP4
DYNLT5	DUSP5
DYRK1A	DUSP5P1
DYRK1B	DUSP6
DYRK2	DUSP7
DYRK3	DUSP8

DYRK4	DUSP9
DYSF	DUT
DZANK1	DUX4
DZIP1	DUXA
DZIP1L	DUXAP10
DZIP3	DUXAP8
E03H4.8	DUXAP9
E2F1	DUXBL1
E2F2	DVL1
E2F3	DVL1P1
E2F4	DVL2
E2F5	DVL3
E2F6	DXO
E2F7	DYDC1
E2F8	DYDC2
E4F1	DYM
EAF1	DYNAP
EAF2	DYNC1H1
EAR1	DYNC1I1
EAR11	DYNC1I2
EAR3	DYNC1LI1
EAR6	DYNC1LI2
EARS2	DYNC2H1
EAT-3	DYNC2I1
EBAG9	DYNC2I2
EBF1	DYNC2LI1
EBF2	DYNLL1
EBF3	DYNLL2
EBF4	DYNLRB1
EBI3	DYNLRB2
EBLN2	DYNLT1
EBLN3P	DYNLT1F
EBNA1BP2	DYNLT2
EBP	DYNLT2A1
EBPIII	DYNLT2B
EBPL	DYNLT3

ECD	DYNLT4
ECE1	DYNLT5
ECE2	DYRK1A
ECEL1	DYRK1B
ECEL1P2	DYRK2
ECH1	DYRK3
ECHDC1	DYRK4
ECHDC2	DYSF
ECHDC3	DZANK1
ECHS1	DZIP1
ECI1	DZIP1L
ECI2	DZIP3
ECI3	E03H4.8
ECM1	E2F1
ECM2	E2F2
ECPAS	E2F3
ECRG4	E2F4
ECSCR	E2F5
ECSIT	E2F6
ECT2	E2F7
ECT2L	E2F8
EDA	E4F1
EDA2R	EAF1
EDAR	EAF2
EDARADD	EAR1
EDC3	EAR11
EDC4	EAR3
EDDM13	EAR6
EDDM3A	EARS2
EDDM3B	EAT-3
EDEM1	EBAG9
EDEM2	EBF1
EDEM3	EBF2
EDF1	EBF3
EDIL3	EBF4
EDN1	EBI3

EDN2
EDN3
EDNRA
EDNRB
EDNRB-AS1
EDRF1
EDRF1-DT
EEA1
EED
EEF1A1
EEF1A1O.L
EEF1A1P24
EEF1A2
EEF1AKMT1
EEF1AKMT2
EEF1AKMT3
EEF1ALPHA1
EEF1B2
EEF1D
EEF1DA
EEF1DB
EEF1E1
EEF1E1-BLOC1S5
EEF1G
EEF2
EEF2K
EEF2KMT
EEFSEC
EEPD1
EFCAB1
EFCAB10
EFCAB11
EFCAB12
EFCAB13
EFCAB14
EFCAB2

EBLN2
EBLN3P
EBNA1BP2
EBP
EBPIII
EBPL
ECD
ECE1
ECE2
ECEL1
ECEL1P2
ECH1
ECHDC1
ECHDC2
ECHDC3
ECHS1
ECI1
ECI2
ECI3
ECM1
ECM2
ECPAS
ECRG4
ECSCR
ECSIT
ECT2
ECT2L
EDA
EDA2R
EDAR
EDARADD
EDC3
EDC4
EDDM13
EDDM3A
EDDM3B

EFCAB3	EDEM1
EFCAB5	EDEM2
EFCAB6	EDEM3
EFCAB7	EDF1
EFCC1	EDIL3
EFEMP1	EDN1
EFEMP2	EDN2
EFEMP2A	EDN3
EFHB	EDNRA
EFHC1	EDNRB
EFHC2	EDNRB-AS1
EFHD1	EDRF1
EFHD2	EDRF1-DT
EFL1	EEA1
EFL1P1	EED
EFNA1	EEF1A1
EFNA1A	EEF1A1O.L
EFNA2	EEF1A1P24
EFNA3	EEF1A2
EFNA4	EEF1AKMT1
EFNA5	EEF1AKMT2
EFNB1	EEF1AKMT3
EFNB2	EEF1ALPHA1
EFNB2A	EEF1B2
EFNB3	EEF1B2P2
EFR3A	EEF1D
EFR3B	EEF1DA
EFS	EEF1DB
EFTUD2	EEF1E1
EGF	EEF1E1-BLOC1S5
EGFEM1	EEF1G
EGFEM1P	EEF2
EGFL6	EEF2K
EGFL7	EEF2KMT
EGFL8	EEFSEC
EGFLAM	EEPD1

EGFR	EFCAB1
EGL-1	EFCAB10
EGL-10	EFCAB11
EGL-44	EFCAB12
EGLN1	EFCAB13
EGLN2	EFCAB14
EGLN3	EFCAB2
EGOT	EFCAB3
EGR	EFCAB5
EGR1	EFCAB6
EGR2	EFCAB7
EGR3	EFCC1
EGR4	EFEMP1
EHBP1	EFEMP2
EHBP1L1	EFEMP2A
EHBP1L1B	EFHB
EHD1	EFHC1
EHD1A	EFHC2
EHD1B	EFHD1
EHD2	EFHD2
EHD3	EFL1
EHD4	EFL1P1
EHF	EFNA1
EHHADH	EFNA1A
EHMT1	EFNA2
EHMT2	EFNA3
EI24	EFNA4
EID1	EFNA5
EID2	EFNB1
EID2B	EFNB2
EID3	EFNB2A
EIF1	EFNB3
EIF1A	EFR3A
EIF1AD	EFR3B
EIF1AX	EFS
EIF1AX-AS1	EFTUD2

EIF1AY	EGF
EIF1B	EGFEM1
EIF2A	EGFEM1P
EIF2AK1	EGFL6
EIF2AK2	EGFL7
EIF2AK3	EGFL8
EIF2AK4	EGFLAM
EIF2B1	EGFR
EIF2B2	EGFR-AS1
EIF2B3	EGL-1
EIF2B4	EGL-10
EIF2B5	EGL-44
EIF2D	EGLN1
EIF2S1	EGLN2
EIF2S2	EGLN3
EIF2S3	EGOT
EIF2S3X	EGR
EIF3A	EGR1
EIF3B	EGR2
EIF3C	EGR3
EIF3CL	EGR4
EIF3D	EHBP1
EIF3E	EHBP1L1
EIF3F	EHBP1L1B
EIF3G	EHD1
EIF3H	EHD1A
EIF3HB	EHD1B
EIF3I	EHD2
EIF3J	EHD3
EIF3J-DT	EHD4
EIF3K	EHF
EIF3L	EHHADH
EIF3M	EHMT1
EIF4A1	EHMT2
EIF4A2	EI24
EIF4A2P4	EID1

EIF4A3
EIF4B
EIF4E
EIF4E1B
EIF4E2
EIF4E3
EIF4EBP1
EIF4EBP2
EIF4EBP3
EIF4EBP3L
EIF4ENIF1
EIF4G
EIF4G1
EIF4G2
EIF4G2B
EIF4G3
EIF4H
EIF5
EIF5A
EIF5A2
EIF5AL1
EIF5B
EIF6
EIPR1
ELAC1
ELAC2
ELANE
ELAPOR1
ELAPOR2
ELAVL1
ELAVL2
ELAVL3
ELAVL4
ELF1
ELF2
ELF3

EID2
EID2B
EID3
EIF1
EIF1A
EIF1AD
EIF1AX
EIF1AX-AS1
EIF1AY
EIF1B
EIF2A
EIF2AK1
EIF2AK2
EIF2AK3
EIF2AK4
EIF2B1
EIF2B2
EIF2B3
EIF2B4
EIF2B5
EIF2D
EIF2S1
EIF2S2
EIF2S3
EIF2S3X
EIF3A
EIF3B
EIF3C
EIF3CL
EIF3D
EIF3E
EIF3F
EIF3G
EIF3H
EIF3HB
EIF3I

ELF4	EIF3J
ELF5	EIF3J-DT
ELFN1	EIF3K
ELFN2	EIF3L
ELK1	EIF3M
ELK2AP	EIF4A1
ELK3	EIF4A2
ELK4	EIF4A2P4
ELL	EIF4A3
ELL2	EIF4B
ELL3	EIF4E
ELMO1	EIF4E1B
ELMO2	EIF4E2
ELMO3	EIF4E3
ELMOD1	EIF4EBP1
ELMOD2	EIF4EBP2
ELMOD3	EIF4EBP3
ELN	EIF4EBP3L
ELNB	EIF4ENIF1
ELO-2	EIF4G
ELOA	EIF4G1
ELOA2	EIF4G2
ELOA3BP	EIF4G2B
ELOA3P	EIF4G3
ELOA-AS1	EIF4H
ELOB	EIF5
ELOC	EIF5A
ELOCP29	EIF5A2
ELOF1	EIF5AL1
ELOVL1	EIF5B
ELOVL2	EIF6
ELOVL2-AS1	EIPR1
ELOVL3	ELAC1
ELOVL4	ELAC2
ELOVL4B	ELANE
ELOVL5	ELAPOR1

ELOVL6	ELAPOR2
ELOVL7	ELAVL1
ELP1	ELAVL2
ELP2	ELAVL3
ELP3	ELAVL4
ELP4	ELF1
ELP5	ELF2
ELP6	ELF3
EMB	ELF4
EMBP1	ELF5
EMC1	ELFN1
EMC10	ELFN2
EMC2	ELK1
EMC3	ELK2AP
EMC4	ELK3
EMC6	ELK4
EMC7	ELL
EMC8	ELL2
EMC9	ELL3
EMCN	ELMO1
EMD	ELMO2
EME1	ELMO3
EME2	ELMOD1
EMG1	ELMOD2
EMID1	ELMOD3
EMILIN1	ELN
EMILIN2	ELNB
EMILIN3	ELO-2
EML1	ELOA
EML2	ELOA2
EML2-AS1	ELOA3BP
EML3	ELOA3P
EML4	ELOA-AS1
EML5	ELOB
EML6	ELOC
EMP1	ELOCP29

EMP2	ELOF1
EMP3	ELOVL1
EMSY	ELOVL2
EMX1	ELOVL2-AS1
EMX2	ELOVL3
EMX2OS	ELOVL4
EN1	ELOVL4B
EN1B	ELOVL5
EN2	ELOVL6
EN2B	ELOVL7
ENAH	ELP1
ENC1	ELP2
ENDOD1	ELP3
ENDOG	ELP4
ENDOU	ELP5
ENDOUL.L	ELP6
ENDOV	EMB
ENG	EMBP1
ENGASE	EMC1
ENHO	EMC10
ENKD1	EMC2
ENKUR	EMC3
ENO1	EMC4
ENO1-AS1	EMC6
ENO1B	EMC7
ENO2	EMC8
ENO3	EMC9
ENO4	EMCN
ENOPH1	EMD
ENOSF1	EME1
ENOX1	EME2
ENOX2	EMG1
ENPEP	EMID1
ENPP1	EMILIN1
ENPP2	EMILIN2
ENPP3	EMILIN3

ENPP4	EML1
ENPP5	EML2
ENPP6	EML2-AS1
ENPP7	EML3
ENSA	EML4
ENTHD1	EML5
ENTPD1	EML6
ENTPD2	EMP1
ENTPD3	EMP2
ENTPD3-AS1	EMP3
ENTPD4	EMSY
ENTPD5	EMX1
ENTPD6	EMX2
ENTPD7	EMX2OS
ENTPD8	EN1
ENTR1	EN1B
ENTREP1	EN2
ENTREP2	EN2B
ENTREP3	ENAH
ENY2	ENC1
EOGT	ENDOD1
EOLA1	ENDOG
EOLA1-DT	ENDOU
EOLA2	ENDOUL.L
EOLA2-DT	ENDOV
EOMES	ENG
EOMESA	ENGASE
EP300	ENHO
EP300-AS1	ENKD1
EP400	ENKUR
EPAS1	ENO1
EPB41	ENO1-AS1
EPB41L1	ENO1B
EPB41L2	ENO2
EPB41L3	ENO3
EPB41L4A	ENO4

EPB41L4A-AS1	ENOPH1
EPB41L4A-DT	ENOSF1
EPB41L4B	ENOX1
EPB41L5	ENOX2
EPB42	ENPEP
EPC1	ENPP1
EPC2	ENPP2
EPCAM	ENPP3
EPCAM-DT	ENPP4
EPD	ENPP5
EPDR1	ENPP6
EPG5	ENPP7
EPGN	ENSA
EPHA1	ENSG00000266919
EPHA10	ENTHD1
EPHA2	ENTPD1
EPHA3	ENTPD2
EPHA4	ENTPD3
EPHA4A	ENTPD3-AS1
EPHA5	ENTPD4
EPHA5-AS1	ENTPD5
EPHA6	ENTPD6
EPHA7	ENTPD7
EPHA8	ENTPD8
EPHB1	ENTR1
EPHB2	ENTREP1
EPHB3	ENTREP2
EPHB3A	ENTREP3
EPHB4	ENY2
EPHB6	EOGT
EPHX1	EOLA1
EPHX2	EOLA1-DT
EPHX3	EOLA2
EPHX4	EOLA2-DT
EPHX5	EOMES
EPIST	EOMESA

EPM2A	EP300
EPM2AIP1	EP300-AS1
EPN1	EP400
EPN2	EPAS1
EPN2-AS1	EPB41
EPN3	EPB41L1
EPO	EPB41L2
EPOP	EPB41L3
EPOR	EPB41L4A
EPPIN	EPB41L4A-AS1
EPPK1	EPB41L4A-DT
EPRS	EPB41L4B
EPRS1	EPB41L5
EPS15	EPB42
EPS15L1	EPC1
EPS15L1A	EPC2
EPS8	EPCAM
EPS8L1	EPCAM-DT
EPS8L2	EPD
EPS8L3	EPDR1
EPSTI1	EPG5
EPX	EPGN
EPYC	EPHA1
EQTN	EPHA10
ERAL1	EPHA2
ERAP1	EPHA3
ERAP2	EPHA4
ERAS	EPHA4A
ERBB2	EPHA5
ERBB3	EPHA5-AS1
ERBB4	EPHA6
ERBIN	EPHA7
ERC1	EPHA8
ERC2	EPHB1
ERC2-IT1	EPHB2
ERCC1	EPHB3

ERCC2	EPHB3A
ERCC3	EPHB4
ERCC4	EPHB6
ERCC5	EPHX1
ERCC6	EPHX2
ERCC6L	EPHX3
ERCC6L2	EPHX4
ERCC6L2-AS1	EPHX5
ERCC8	EPIST
ERDR1	EPM2A
EREG	EPM2AIP1
ERF	EPN1
ERFE	EPN2
ERG	EPN2-AS1
ERG28	EPN3
ERGIC1	EPO
ERGIC2	EPOP
ERGIC3	EPOR
ERH	EPPIN
ERI1	EPPK1
ERI2	EPRS
ERICH1	EPRS1
ERICH3	EPS15
ERICH4	EPS15L1
ERICH5	EPS15L1A
ERICH6	EPS8
ERICH6-AS1	EPS8L1
ERLEC1	EPS8L2
ERLEC1P1	EPS8L3
ERLIN1	EPSTI1
ERLIN2	EPX
ERMAP	EPYC
ERMARD	EQTN
ERMN	ERAL1
ERMP1	ERAP1
ERN1	ERAP2

ERN2	ERAS
ERO1A	ERBB2
ERO1B	ERBB3
ERP27	ERBB4
ERP29	ERBIN
ERP44	ERC1
ERRFI1	ERC2
ERV3-1	ERC2-IT1
ERV9-1	ERCC1
ERVFRD-1	ERCC2
ERVH48-1	ERCC3
ERVH-6	ERCC4
ERVK-6	ERCC5
ERVMER34-1	ERCC6
ERVMER61-1	ERCC6L
ERVW-1	ERCC6L2
ES2	ERCC6L2-AS1
ESAM	ERCC8
ESCO1	ERDR1
ESCO2	EREG
ESD	ERF
ESF1	ERFE
ESM1	ERG
ESP38	ERG28
ESPL1	ERGIC1
ESPN	ERGIC2
ESPNL	ERGIC3
ESPNP	ERH
ESR1	ERI1
ESR2	ERI2
ESRG	ERICH1
ESRP1	ERICH3
ESRP2	ERICH4
ESRRA	ERICH5
ESRRB	ERICH6
ESRRG	ERICH6-AS1

ESS2	ERLEC1
ESX1	ERLEC1P1
ESYT1	ERLIN1
ESYT2	ERLIN2
ESYT2.S	ERMAP
ESYT3	ERMARD
ETAA1	ERMN
ETD	ERMP1
ETF1	ERN1
ETFA	ERN2
ETFB	ERO1A
ETFBKMT	ERO1B
ETFDH	ERP27
ETFRF1	ERP29
ETHE1	ERP44
ETL4	ERRFI1
ETNK1	ERV3-1
ETNK2	ERV9-1
ETNPPL	ERVFRD-1
ETS1	ERVH48-1
ETS2	ERVH-6
ETV1	ERVK-10
ETV2	ERVK-18
ETV3	ERVK-19
ETV4	ERVK-21
ETV5	ERVK-24
ETV5-AS1	ERVK-25
ETV5B	ERVK-6
ETV6	ERVK-7
ETV7	ERVK-8
EVA1A	ERVK-9
EVA1B	ERVMER34-1
EVA1C	ERVMER61-1
EVC	ERVW-1
EVC2	ES2
EVI1	ESAM

EVI2A	ESCO1
EVI2B	ESCO2
EVI5	ESD
EVI5L	ESF1
EVL	ESM1
EVPL	ESP38
EVPLL	ESPL1
EVX1	ESPN
EWSAT1	ESPNL
EWSR1	ESPNP
EXD2	ESR1
EXD3	ESR2
EXO1	ESRG
EXO5	ESRP1
EXO70	ESRP2
EXOC1	ESRRA
EXOC2	ESRRB
EXOC3	ESRRG
EXOC3-AS1	ESS2
EXOC3L	ESX1
EXOC3L1	ESYT1
EXOC3L2	ESYT2
EXOC3L4	ESYT2.S
EXOC4	ESYT3
EXOC5	ETAA1
EXOC6	ETD
EXOC6B	ETF1
EXOC7	ETFA
EXOC8	ETFB
EXOG	ETFBKMT
EXOSC1	ETFDH
EXOSC10	ETFRF1
EXOSC10-AS1	ETHE1
EXOSC2	ETL4
EXOSC3	ETNK1
EXOSC4	ETNK2

EXOSC5	ETNPPL
EXOSC6	ETS1
EXOSC7	ETS2
EXOSC8	ETV1
EXOSC9	ETV2
EXPH5	ETV3
EXT1	ETV4
EXT2	ETV5
EXTL1	ETV5-AS1
EXTL2	ETV5B
EXTL3	ETV6
EXTL3-AS1	ETV7
EYA1	EVA1A
EYA2	EVA1B
EYA3	EVA1C
EYA4	EVC
EYS	EVC2
EZH1	EVI1
EZH2	EVI2A
EZHIP	EVI2B
EZR	EVI5
F01D5.2	EVI5L
F01D5.3	EVL
F07C6.6	EVPL
F09C8.1	EVPLL
F10	EVX1
F11	EWSAT1
F11R	EWSR1
F12	EXD2
F13A1	EXD3
F13A1B	EXO1
F13B	EXO5
F17B5.8	EXO70
F17C11.15	EXOC1
F17E9.5	EXOC2
F2	EXOC3

F2R	EXOC3-AS1
F2RL1	EXOC3L
F2RL2	EXOC3L1
F2RL3	EXOC3L2
F3	EXOC3L4
F35E12.6	EXOC4
F36G9.15	EXOC5
F39B2.14	EXOC6
F39B2.3	EXOC6B
F43D9.8	EXOC7
F46A8.9	EXOC8
F46C3.7	EXOG
F5	EXOSC1
F53G12.9	EXOSC10
F55G11.2	EXOSC10-AS1
F55G11.4	EXOSC2
F55G11.6	EXOSC3
F56A4.2	EXOSC4
F56D5.3	EXOSC5
F7	EXOSC6
F8	EXOSC7
F8A	EXOSC8
F8A1	EXOSC9
F8A2	EXPH5
F8A3	EXT1
F9	EXT2
FA2H	EXTL1
FAAH	EXTL2
FAAP100	EXTL3
FAAP20	EXTL3-AS1
FAAP24	EYA1
FABP1	EYA2
FABP10A	EYA3
FABP1B.1	EYA4
FABP2	EYS
FABP3	EZH1

FABP3P2	EZH2
FABP4	EZHIP
FABP4A	EZR
FABP5	F01D5.2
FABP5L2	F01D5.3
FABP5P3	F07C6.6
FABP6	F09C8.1
FABP7	F10
FABP7A	F11
FABP9	F11R
FADD	F12
FADS1	F13A1
FADS2	F13A1B
FADS3	F13B
FAF1	F17B5.8
FAF2	F17C11.15
FAH	F17E9.5
FAHD1	F2
FAHD2A	F2R
FAHD2B	F2RL1
FAHD2CP	F2RL2
FAIM	F2RL3
FAIM2	F3
FAM102A	F35E12.6
FAM102B	F36G9.15
FAM104A	F39B2.14
FAM104B	F39B2.3
FAM105A	F43D9.8
FAM106A	F46A8.9
FAM106C	F46C3.7
FAM107A	F5
FAM107B	F53G12.9
FAM110A	F55G11.2
FAM110B	F55G11.4
FAM110C	F55G11.6
FAM111A	F56A4.2

FAM111A-DT	F56D5.3
FAM111B	F7
FAM114A1	F8
FAM114A2	F8A
FAM117A	F8A1
FAM117B	F8A2
FAM118A	F8A3
FAM118B	F9
FAM120A	FA2H
FAM120A2P	FAAH
FAM120AOS	FAAP100
FAM120B	FAAP20
FAM120C	FAAP24
FAM122A	FABP1
FAM122B	FABP10A
FAM124A	FABP1B.1
FAM124B	FABP2
FAM126A	FABP3
FAM126B	FABP3P2
FAM127A	FABP4
FAM127C	FABP4A
FAM129B	FABP5
FAM131A	FABP5L2
FAM131B	FABP5P3
FAM131C	FABP6
FAM133A	FABP7
FAM133B	FABP7A
FAM133CP	FABP9
FAM135A	FADD
FAM135B	FADS1
FAM136A	FADS2
FAM138D	FADS3
FAM13A	FAF1
FAM13A-AS1	FAF2
FAM13B	FAH
FAM13C	FAHD1

FAM149A	FAHD2A
FAM149B	FAHD2B
FAM149B1	FAHD2CP
FAM151A	FAIM
FAM153A	FAIM2
FAM153B	FALEC
FAM153CP	FAM102A
FAM161A	FAM102B
FAM161B	FAM104A
FAM162A	FAM104B
FAM162B	FAM105A
FAM163A	FAM106A
FAM163B	FAM106C
FAM166A	FAM107A
FAM166B	FAM107B
FAM166C	FAM110A
FAM167A	FAM110B
FAM167B	FAM110C
FAM168A	FAM111A
FAM168B	FAM111A-DT
FAM169A	FAM111B
FAM170A	FAM114A1
FAM170B	FAM114A2
FAM171A1	FAM117A
FAM171A2	FAM117B
FAM171B	FAM118A
FAM172A	FAM118B
FAM174A	FAM120A
FAM174B	FAM120A2P
FAM174C	FAM120AOS
FAM177A	FAM120B
FAM177A1	FAM120C
FAM177B	FAM122A
FAM178B	FAM122B
FAM180A	FAM124A
FAM180B	FAM124B

FAM181A	FAM126A
FAM181A-AS1	FAM126B
FAM181B	FAM127A
FAM182A	FAM127C
FAM182B	FAM129B
FAM183A	FAM131A
FAM183B	FAM131B
FAM184A	FAM131C
FAM184B	FAM133A
FAM185A	FAM133B
FAM186A	FAM133CP
FAM186B	FAM135A
FAM187B	FAM135B
FAM193A	FAM136A
FAM193B	FAM138D
FAM199X	FAM13A
FAM200A	FAM13A-AS1
FAM200B	FAM13B
FAM201A	FAM13C
FAM204A	FAM149A
FAM205A	FAM149B
FAM205BP	FAM149B1
FAM209A	FAM151A
FAM209B	FAM153A
FAM20A	FAM153B
FAM20B	FAM153CP
FAM20C	FAM161A
FAM210A	FAM161B
FAM210B	FAM162A
FAM214A	FAM162B
FAM214B	FAM163A
FAM215A	FAM163B
FAM216A	FAM166A
FAM216B	FAM166B
FAM217A	FAM166C
FAM217B	FAM167A

FAM218A	FAM167B
FAM219A	FAM168A
FAM219B	FAM168B
FAM21A	FAM169A
FAM21EP	FAM170A
FAM220A	FAM170B
FAM221A	FAM171A1
FAM221B	FAM171A2
FAM222A	FAM171B
FAM222A-AS1	FAM172A
FAM222B	FAM174A
FAM223A	FAM174B
FAM223B	FAM174C
FAM224A	FAM177A
FAM224B	FAM177A1
FAM225A	FAM177B
FAM226B	FAM178B
FAM227A	FAM180A
FAM227B	FAM180B
FAM228B	FAM181A
FAM229A	FAM181A-AS1
FAM229B	FAM181B
FAM230B	FAM182A
FAM234A	FAM182B
FAM234B	FAM183A
FAM238A	FAM183B
FAM238B	FAM184A
FAM238C	FAM184B
FAM240B	FAM185A
FAM241A	FAM186A
FAM241B	FAM186B
FAM24A	FAM187B
FAM24B	FAM193A
FAM25A	FAM193B
FAM25BP	FAM199X
FAM25C	FAM200A

FAM25G	FAM200B
FAM27B	FAM201A
FAM27C	FAM204A
FAM27E3	FAM205A
FAM27E5	FAM205BP
FAM29A	FAM209A
FAM30A	FAM209B
FAM32A	FAM20A
FAM3A	FAM20B
FAM3B	FAM20C
FAM3C	FAM210A
FAM3D	FAM210B
FAM41AY1	FAM214A
FAM41C	FAM214B
FAM43A	FAM215A
FAM43B	FAM216A
FAM46A	FAM216B
FAM46B	FAM217A
FAM47A	FAM217B
FAM47B	FAM218A
FAM47C	FAM219A
FAM47E	FAM219B
FAM47E-STBD1	FAM21A
FAM50A	FAM21EP
FAM50B	FAM220A
FAM53A	FAM221A
FAM53B	FAM221B
FAM53C	FAM222A
FAM63A	FAM222A-AS1
FAM63B	FAM222B
FAM65A	FAM223A
FAM65B	FAM223B
FAM65C	FAM224A
FAM66A	FAM224B
FAM66C	FAM225A
FAM66D	FAM226B

FAM72A	FAM227A
FAM72B	FAM227B
FAM72D	FAM228B
FAM76A	FAM229A
FAM76B	FAM229B
FAM78A	FAM230B
FAM78B	FAM234A
FAM81A	FAM234B
FAM81B	FAM238A
FAM83A	FAM238B
FAM83A-AS1	FAM238C
FAM83B	FAM240B
FAM83C	FAM241A
FAM83C-AS1	FAM241B
FAM83D	FAM24A
FAM83E	FAM24B
FAM83F	FAM25A
FAM83G	FAM25BP
FAM83H	FAM25C
FAM86B1	FAM25G
FAM86B3P	FAM27B
FAM86C1P	FAM27C
FAM86C2P	FAM27E3
FAM86DP	FAM27E5
FAM86EP	FAM29A
FAM86FP	FAM30A
FAM86HP	FAM32A
FAM86JP	FAM3A
FAM87B	FAM3B
FAM89A	FAM3C
FAM89B	FAM3D
FAM8A1	FAM41AY1
FAM90A1	FAM41C
FAM90A20P	FAM43A
FAM91A1	FAM43B
FAM98A	FAM46A

FAM98B	FAM46B
FAM98C	FAM47A
FAM9B	FAM47B
FAM9C	FAM47C
FAN1	FAM47E
FANCA	FAM47E-STBD1
FANCB	FAM50A
FANCC	FAM50B
FANCD2	FAM53A
FANCD2OS	FAM53B
FANCE	FAM53C
FANCF	FAM63A
FANCG	FAM63B
FANCI	FAM65A
FANCL	FAM65B
FANCM	FAM65C
FANK1	FAM66A
FAP	FAM66C
FAR1	FAM66D
FAR2	FAM72A
FARP1	FAM72B
FARP2	FAM72D
FARS2	FAM76A
FARSA	FAM76B
FARSB	FAM78A
FAS	FAM78B
FAS2	FAM81A
FASL	FAM81B
FASLG	FAM83A
FASN	FAM83A-AS1
FASN1	FAM83B
FASTK	FAM83C
FASTKD1	FAM83C-AS1
FASTKD2	FAM83D
FASTKD5	FAM83E
FAT1	FAM83F

FAT2	FAM83G
FAT3	FAM83H
FAT4	FAM86B1
FATE1	FAM86B3P
FAU	FAM86C1P
FAXC	FAM86C2P
FAXDC2	FAM86DP
FBF1	FAM86EP
FBH1	FAM86FP
FBL	FAM86HP
FBLIM1	FAM86JP
FBLL1	FAM87B
FBLN1	FAM89A
FBLN2	FAM89B
FBLN5	FAM8A1
FBLN7	FAM90A1
FBN1	FAM90A20P
FBN2	FAM91A1
FBN3	FAM98A
FBP	FAM98B
FBP1	FAM98C
FBP1B	FAM9B
FBP2	FAM9C
FBRS	FAN1
FBRSL1	FANCA
FBXA-163	FANCB
FBXA-2	FANCC
FBXA-217	FANCD2
FBXA-95	FANCD2OS
FBXL12	FANCE
FBXL13	FANCF
FBXL14	FANCG
FBXL15	FANCI
FBXL16	FANCL
FBXL17	FANCM
FBXL18	FANK1

FBXL19	FAP
FBXL19-AS1	FAR1
FBXL2	FAR2
FBXL20	FAR2P1
FBXL21	FARP1
FBXL21P	FARP2
FBXL3	FARS2
FBXL4	FARSA
FBXL5	FARSB
FBXL6	FAS
FBXL7	FAS2
FBXL8	FAS-AS1
FBXO10	FASL
FBXO11	FASLG
FBXO15	FASN
FBXO16	FASN1
FBXO17	FASTK
FBXO2	FASTKD1
FBXO21	FASTKD2
FBXO22	FASTKD5
FBXO24	FAT1
FBXO25	FAT2
FBXO27	FAT3
FBXO28	FAT4
FBXO3	FATE1
FBXO30	FAU
FBXO31	FAXC
FBXO32	FAXDC2
FBXO33	FBF1
FBXO34	FBH1
FBXO36	FBL
FBXO38	FBLIM1
FBXO39	FBLL1
FBXO40	FBLN1
FBXO41	FBLN2
FBXO42	FBLN5

FBXO43	FBLN7
FBXO44	FBN1
FBXO45	FBN2
FBXO46	FBN3
FBXO47	FBP
FBXO48	FBP1
FBXO5	FBP1B
FBXO6	FBP2
FBXO7	FBRS
FBXO8	FBRSL1
FBXO9	FBXA-163
FBXW10	FBXA-2
FBXW10B	FBXA-217
FBXW11	FBXA-95
FBXW12	FBXL12
FBXW2	FBXL13
FBXW4	FBXL14
FBXW4P1	FBXL15
FBXW5	FBXL16
FBXW7	FBXL17
FBXW9	FBXL18
FCAR	FBXL19
FCER1A	FBXL19-AS1
FCER1G	FBXL2
FCER2	FBXL20
FCER2A	FBXL21
FCF1	FBXL21P
FCGBP	FBXL3
FCGR1	FBXL4
FCGR1A	FBXL5
FCGR1BP	FBXL6
FCGR2A	FBXL7
FCGR2B	FBXL8
FCGR2C	FBXO10
FCGR3	FBXO11
FCGR3A	FBXO15

FCGR3B	FBXO16
FCGRT	FBXO17
FCHO1	FBXO2
FCHO2	FBXO21
FCHSD1	FBXO22
FCHSD2	FBXO24
FCMR	FBXO25
FCN1	FBXO27
FCN2	FBXO28
FCN3	FBXO3
FCNA	FBXO30
FCNB	FBXO31
FCRL1	FBXO32
FCRL2	FBXO33
FCRL3	FBXO34
FCRL5	FBXO36
FCRLA	FBXO38
FCRLB	FBXO39
FCRLS	FBXO40
FCSK	FBXO41
FDCSP	FBXO42
FDFT1	FBXO43
FDPS	FBXO44
FDPSP2	FBXO45
FDX1	FBXO46
FDX2	FBXO47
FDXACB1	FBXO48
FDXR	FBXO5
FECH	FBXO6
FEM1A	FBXO7
FEM1B	FBXO8
FEM1C	FBXO9
FEN1	FBXW10
FENDRR	FBXW10B
FER	FBXW11
FER1HCH	FBXW12

FER1L4	FBXW2
FER1L5	FBXW4
FER1L6	FBXW4P1
FER1L6-AS2	FBXW5
FERD3L	FBXW7
FERMT1	FBXW9
FERMT2	FCAR
FERMT3	FCER1A
FES	FCER1G
FETUB	FCER2
FEZ1	FCER2A
FEZ2	FCF1
FEZF1	FCGBP
FEZF2	FCGR1
FFAR1	FCGR1A
FFAR2	FCGR1BP
FFAR3	FCGR2A
FGA	FCGR2B
FGB	FCGR2C
FGD1	FCGR3
FGD2	FCGR3A
FGD3	FCGR3B
FGD4	FCGRT
FGD5	FCHO1
FGD5-AS1	FCHO2
FGD6	FCHSD1
FGF1	FCHSD2
FGF10	FCMR
FGF11	FCN1
FGF12	FCN2
FGF13	FCN3
FGF13-AS1	FCNA
FGF14	FCNB
FGF14-AS2	FCRL1
FGF15	FCRL2
FGF16	FCRL3

FGF17	FCRL5
FGF18	FCRLA
FGF19	FCRLB
FGF2	FCRLS
FGF20	FCSK
FGF21	FDCSP
FGF22	FDFT1
FGF23	FDPS
FGF3	FDPSP2
FGF4	FDX1
FGF5	FDX2
FGF6	FDXACB1
FGF7	FDXR
FGF8	FECH
FGF9	FEM1A
FGFBP1	FEM1B
FGFBP2	FEM1C
FGFBP3	FEN1
FGFR1	FENDRR
FGFR1OP2	FER
FGFR2	FER1HCH
FGFR3	FER1L4
FGFR4	FER1L5
FGFRL1	FER1L6
FGG	FER1L6-AS2
FGGY	FERD3L
FGL1	FERMT1
FGL2	FERMT2
FGR	FERMT3
FH	FES
FH1	FETUB
FHAD1	FEZ1
FHDC1	FEZ2
FHIP1A	FEZF1
FHIP1B	FEZF1-AS1
FHIP2A	FEZF2

FHIP2B	FFAR1
FHIT	FFAR2
FHL1	FFAR3
FHL2	FGA
FHL3	FGB
FHL5	FGD1
FHOD1	FGD2
FHOD3	FGD3
FIBCD1	FGD4
FIBIN	FGD5
FIBP	FGD5-AS1
FICD	FGD6
FIG4	FGF1
FIGN	FGF10
FIGNL1	FGF11
FIGNL2	FGF12
FILIP1	FGF13
FILIP1L	FGF13-AS1
FIP1L1	FGF14
FIS1	FGF14-AS2
FITM1	FGF15
FITM2	FGF16
FIZ1	FGF17
FJX1	FGF18
FKBP10	FGF19
FKBP11	FGF2
FKBP14	FGF20
FKBP15	FGF21
FKBP1A	FGF22
FKBP1AB	FGF23
FKBP1B	FGF3
FKBP2	FGF4
FKBP3	FGF5
FKBP4	FGF6
FKBP5	FGF7
FKBP6	FGF8

FKBP7	FGF9
FKBP8	FGFBP1
FKBP9	FGFBP2
FKBP9P1	FGFBP3
FKBPL	FGFR1
FKRP	FGFR1OP
FKSG49	FGFR1OP2
FKTN	FGFR2
FLACC1	FGFR3
FLAD1	FGFR4
FLCN	FGFRL1
FLG	FGG
FLG2	FGGY
FLG-AS1	FGL1
FLI1	FGL2
FLII	FGR
FLNA	FH
FLNB	FH1
FLNB-AS1	FHAD1
FLNC	FHDC1
FLOT1	FHIP1A
FLOT2	FHIP1B
FLOT2B	FHIP2A
FLRT1	FHIP2B
FLRT2	FHIT
FLRT3	FHL1
FLT1	FHL2
FLT3	FHL3
FLT3LG	FHL5
FLT4	FHOD1
FLVCR1	FHOD3
FLVCR1-DT	FIBCD1
FLVCR2	FIBIN
FLVCR2B	FIBP
FLYWCH1	FICD
FLYWCH2	FIG4

FMC1	FIGN
FMN1	FIGNL1
FMN2	FIGNL2
FMNL1	FILIP1
FMNL2	FILIP1L
FMNL3	FIP1L1
FMO1	FIS1
FMO2	FITM1
FMO3	FITM2
FMO4	FIZ1
FMO5	FJX1
FMO6P	FKBP10
FMOD	FKBP11
FMR1	FKBP14
FMR1NB	FKBP15
FN1	FKBP1A
FN3K	FKBP1AB
FN3KRP	FKBP1B
FNBP1	FKBP2
FNBP1L	FKBP3
FNBP4	FKBP4
FNDC1	FKBP5
FNDC10	FKBP6
FNDC11	FKBP7
FNDC3A	FKBP8
FNDC3B	FKBP9
FNDC3C1	FKBP9P1
FNDC4	FKBPL
FNDC5	FKRP
FNDC8	FKSG49
FNDC9	FKTN
FNIP1	FLACC1
FNIP2	FLAD1
FNTA	FLCN
FNTB	FLG
FOCAD	FLG2

FOCAD-AS1	FLG-AS1
FOLH1	FLI1
FOLR1	FLII
FOLR2	FLNA
FOLR3	FLNB
FOLT-1	FLNB-AS1
FOS	FLNC
FOSAB	FLOT1
FOSB	FLOT2
FOSL1	FLOT2B
FOSL2	FLRT1
FOXA1	FLRT2
FOXA2	FLRT3
FOXA3	FLT1
FOXB1	FLT3
FOXB1A	FLT3LG
FOXC1	FLT4
FOXC2	FLVCR1
FOXD1	FLVCR1-DT
FOXD2	FLVCR2
FOXD2-AS1	FLVCR2B
FOXD3	FLYWCH1
FOXD4	FLYWCH2
FOXD4L1	FMC1
FOXD4L3	FMN1
FOXD4L6	FMN2
FOXE1	FMNL1
FOXE3	FMNL2
FOXF1	FMNL3
FOXF2	FMO1
FOXG1	FMO2
FOXG1A	FMO3
FOXH1	FMO4
FOXI1	FMO5
FOXI2	FMO6P
FOXJ1	FMOD

FOXJ2	FMR1
FOXJ3	FMR1NB
FOXK1	FN1
FOXK2	FN3K
FOXL1	FN3KRP
FOXL2	FNBP1
FOXL2NB	FNBP1L
FOXM1	FNBP4
FOXN1	FNDC1
FOXN2	FNDC10
FOXN3	FNDC11
FOXN3-AS1	FNDC3A
FOXN3-AS2	FNDC3B
FOXN4	FNDC3C1
FOXO1	FNDC4
FOXO3	FNDC5
FOXO3A	FNDC8
FOXO3B	FNDC9
FOXO4	FNIP1
FOXO6	FNIP2
FOXP1	FNTA
FOXP1-IT1	FNTB
FOXP2	FOCAD
FOXP3	FOCAD-AS1
FOXP4	FOLH1
FOXQ1	FOLR1
FOXRED1	FOLR2
FOXRED2	FOLR3
FOXS1	FOLT-1
FPGS	FOS
FPGT	FOSAB
FPGT-TNNI3K	FOSB
FPR1	FOSL1
FPR2	FOSL2
FPR3	FOXA1
FPR-RS4	FOXA2

FRA10AC1	FOXA3
FRAS1	FOXB1
FRAT1	FOXB1A
FRAT2	FOXC1
FREM1	FOXC2
FREM2	FOXC2-AS1
FRG1	FOXD1
FRG2C	FOXD2
FRK	FOXD2-AS1
FRMD1	FOXD3
FRMD3	FOXD4
FRMD4A	FOXD4L1
FRMD4B	FOXD4L3
FRMD5	FOXD4L6
FRMD6	FOXE1
FRMD6-AS1	FOXE3
FRMD7	FOXF1
FRMD8	FOXF2
FRMPD1	FOXG1
FRMPD2	FOXG1A
FRMPD4	FOXH1
FRRS1	FOXI1
FRRS1L	FOXI2
FRS2	FOXJ1
FRS3	FOXJ2
FRY	FOXJ3
FRYL	FOXK1
FRZB	FOXK2
FSBP	FOXL1
FSCB	FOXL2
FSCN1	FOXL2NB
FSCN2	FOXM1
FSD1	FOXN1
FSD1L	FOXN2
FSD2	FOXN3
FSHB	FOXN3-AS1

FSHR	FOXN3-AS2
FSIP1	FOXN4
FSIP2-AS2	FOXO1
FST	FOXO3
FSTA	FOXO3A
FSTL1	FOXO3B
FSTL3	FOXO4
FSTL4	FOXO6
FSTL5	FOXP1
FTCD	FOXP1-IT1
FTH1	FOXP2
FTH1A	FOXP3
FTH1B	FOXP4
FTH1P1	FOXP4-AS1
FTH1P11	FOXQ1
FTH1P12	FOXR2
FTH1P16	FOXRED1
FTH1P19	FOXRED2
FTH1P2	FOXS1
FTH1P20	FPGS
FTH1P3	FPGT
FTH1P7	FPGT-TNNI3K
FTHL17	FPR1
FTL	FPR2
FTL1	FPR3
FTLP3	FPR-RS4
FTMT	FRA10AC1
FTO	FRAS1
FTO-IT1	FRAT1
FTSJ1	FRAT2
FTSJ3	FREM1
FTX	FREM2
FUBP1	FRG1
FUBP3	FRG2C
FUCA1	FRK
FUCA2	FRMD1

FUNDC1	FRMD3
FUNDC2	FRMD4A
FUNDC2P2	FRMD4B
FUOM	FRMD5
FURIN	FRMD6
FUS	FRMD6-AS1
FUT1	FRMD7
FUT10	FRMD8
FUT11	FRMPD1
FUT2	FRMPD2
FUT3	FRMPD4
FUT4	FRRS1
FUT5	FRRS1L
FUT6	FRS2
FUT7	FRS3
FUT8	FRY
FUT8-AS1	FRYL
FUT9	FRZB
FUZ	FSBP
FXN	FSCB
FXR1	FSCN1
FXR2	FSCN2
FXYD1	FSD1
FXYD2	FSD1L
FXYD3	FSD2
FXYD4	FSHB
FXYD5	FSHR
FXYD6	FSIP1
FXYD7	FSIP2-AS2
FYB	FST
FYB1	FSTA
FYB2	FSTL1
FYCO1	FSTL3
FYN	FSTL4
FYTTD1	FSTL5
FZD1	FTCD

FZD10	FTH1
FZD10-AS1	FTH1A
FZD2	FTH1B
FZD3	FTH1P1
FZD4	FTH1P11
FZD5	FTH1P12
FZD6	FTH1P16
FZD7	FTH1P19
FZD8	FTH1P2
FZD9	FTH1P20
FZO-1	FTH1P3
FZR1	FTH1P7
G0S2	FTHL17
G2E3	FTL
G3BP1	FTL1
G3BP2	FTLP3
G6PC	FTMT
G6PC1	FTO
G6PC2	FTO-IT1
G6PC3	FTSJ1
G6PD	FTSJ3
G6PD2	FTX
G6PDX	FUBP1
GAA	FUBP3
GAB1	FUCA1
GAB2	FUCA2
GAB3	FUNDC1
GABARAP	FUNDC2
GABARAPL1	FUNDC2P2
GABARAPL2	FUOM
GABARAPL3	FURIN
GABBR1	FUS
GABBR2	FUT1
GABPA	FUT10
GABPB1	FUT11
GABPB1-AS1	FUT2

GABPB2	FUT3
GABRA1	FUT4
GABRA2	FUT5
GABRA3	FUT6
GABRA4	FUT7
GABRA5	FUT8
GABRA6	FUT8-AS1
GABRB1	FUT9
GABRB2	FUZ
GABRB3	FXN
GABRD	FXR1
GABRE	FXR2
GABRG1	FXYD1
GABRG2	FXYD2
GABRG3	FXYD3
GABRP	FXYD4
GABRQ	FXYD5
GABRR1	FXYD6
GABRR2	FXYD7
GAD1	FYB
GAD2	FYB1
GADD45A	FYB2
GADD45AA	FYCO1
GADD45B	FYN
GADD45BA	FYTTD1
GADD45G	FZD1
GADD45GIP1	FZD10
GAGE12B	FZD10-AS1
GAGE4	FZD2
GAGE5	FZD3
GAGE7	FZD4
GAK	FZD5
GAL	FZD6
GAL3	FZD7
GAL3ST1	FZD8
GAL3ST2	FZD9

GAL3ST3	FZO-1
GAL3ST4	FZR1
GALC	G0S2
GALCB	G2E3
GALE	G3BP1
GALK1	G3BP2
GALK2	G6PC
GALM	G6PC1
GALNS	G6PC2
GALNT1	G6PC3
GALNT10	G6PD
GALNT11	G6PD2
GALNT12	G6PDX
GALNT13	GAA
GALNT14	GAB1
GALNT15	GAB2
GALNT16	GAB3
GALNT17	GABARAP
GALNT18	GABARAPL1
GALNT2	GABARAPL2
GALNT3	GABARAPL3
GALNT4	GABBR1
GALNT5	GABBR2
GALNT6	GABPA
GALNT7	GABPB1
GALNT9	GABPB1-AS1
GALNTL5	GABPB1-IT1
GALNTL6	GABPB2
GALP	GABRA1
GALR1	GABRA2
GALR2	GABRA3
GALR3	GABRA4
GALT	GABRA5
GAMT	GABRA6
GAN	GABRB1
GANAB	GABRB2

GANC	GABRB3
GAP43	GABRD
GAP43.L	GABRE
GAPDH	GABRG1
GAPDHS	GABRG2
GAPT	GABRG3
GAPVD1	GABRP
GAR1	GABRQ
GAREM	GABRR1
GAREM1	GABRR2
GAREM2	GACAT2
GAREML	GACAT3
GARIN1B	GAD1
GARIN2	GAD2
GARIN3	GADD45A
GARIN4	GADD45AA
GARIN5A	GADD45B
GARIN5B	GADD45BA
GARIN6	GADD45G
GARNL3	GADD45GIP1
GARRE1	GADL1
GARS	GAGE1
GARS1	GAGE12B
GART	GAGE4
GAS1	GAGE5
GAS-1	GAGE7
GAS1RR	GAK
GAS2	GAL
GAS2L1	GAL3
GAS2L1P2	GAL3ST1
GAS2L3	GAL3ST2
GAS5	GAL3ST3
GAS5-AS1	GAL3ST4
GAS6	GALC
GAS6-AS1	GALCB
GAS7	GALE

GAS8	GALK1
GAS8-AS1	GALK2
GASK1A	GALM
GASK1B	GALNS
GASK1B-AS1	GALNT1
GAST	GALNT10
GATA1	GALNT11
GATA1A	GALNT12
GATA2	GALNT13
GATA2A	GALNT14
GATA2-AS1	GALNT15
GATA3	GALNT16
GATA4	GALNT17
GATA5	GALNT18
GATA6	GALNT2
GATA6-AS1	GALNT3
GATAD1	GALNT4
GATAD2A	GALNT5
GATAD2B	GALNT6
GATB	GALNT7
GATC	GALNT9
GATD1	GALNTL5
GATD3	GALNTL6
GATM	GALP
GATS	GALR1
GBA1	GALR2
GBA2	GALR3
GBA3	GALT
GBAP1	GAMT
GBE1	GAN
GBF1	GANAB
GBGT1	GANC
GBP	GAP43
GBP1	GAP43.L
GBP10	GAPDH
GBP2	GAPDHS

GBP3	GAPLINC
GBP4	GAPT
GBP5	GAPVD1
GBP6	GAR1
GBP7	GAREM
GBX1	GAREM1
GBX2	GAREM2
GBX2.1.S	GAREML
GBX2.2.L	GARIN1B
GC	GARIN2
GCA	GARIN3
GCAT	GARIN4
GCC1	GARIN5A
GCC2	GARIN5B
GCDH	GARIN6
GCFC2	GARNL3
GCG	GARRE1
GCGR	GARS
GCH1	GARS1
GCH2	GART
GCHFR	GAS1
GCK	GAS-1
GCKR	GAS1RR
GCLC	GAS2
GCLM	GAS2L1
GCM1	GAS2L1P2
GCM2	GAS2L3
GCN1	GAS5
GCNA	GAS5-AS1
GCNT1	GAS6
GCNT2	GAS6-AS1
GCNT3	GAS7
GCNT4	GAS8
GCOM1	GAS8-AS1
GCSAM	GASAL1
GCSAML	GASK1A

GCSH	GASK1B
GCSHB	GASK1B-AS1
GCSHP3	GAST
GDA	GATA1
GDAP1	GATA1A
GDAP10	GATA2
GDAP1L1	GATA2A
GDAP2	GATA2-AS1
GDE1	GATA3
GDF1	GATA4
GDF10	GATA5
GDF11	GATA6
GDF15	GATA6-AS1
GDF2	GATAD1
GDF3	GATAD2A
GDF5	GATAD2B
GDF5-AS1	GATB
GDF6	GATC
GDF7	GATD1
GDF9	GATD3
GDI1	GATM
GDI2	GATS
GDNF	GBA1
GDPD1	GBA2
GDPD2	GBA3
GDPD3	GBAP1
GDPD4	GBE1
GDPD5	GBF1
GDPGP1	GBGT1
GEM	GBP
GEMIN2	GBP1
GEMIN4	GBP10
GEMIN5	GBP2
GEMIN6	GBP3
GEMIN7	GBP4
GEMIN8	GBP5

GEMIN8P4	GBP6
GEN1	GBP7
GET1	GBX1
GET3	GBX2
GET4	GBX2.1.S
GFAP	GBX2.2.L
GFER	GC
GFI1	GCA
GFM1	GCAT
GFM2	GCC1
GFOD1	GCC2
GFOD1-AS1	GCDH
GFOD2	GCFC2
GFPT1	GCG
GFPT2	GCGR
GFRA1	GCH1
GFRA2	GCH2
GFRA3	GCHFR
GFRA4	GCK
GFRAL	GCKR
GFUS	GCLC
GGA1	GCLM
GGA2	GCM1
GGA3	GCM2
GGACT	GCN1
GGCT	GCNA
GGCTB	GCNT1
GGCX	GCNT2
GGH	GCNT3
GGN	GCNT4
GGNBP2	GCOM1
GGPS1	GCSAM
GGT1	GCSAML
GGT2P	GCSH
GGT3P	GCSHB
GGT5	GCSHP3

GGT6	GDA
GGT7	GDAP1
GGT8P	GDAP10
GGTA1	GDAP1L1
GGTLC1	GDAP2
GGTLC2	GDE1
GGTLC3	GDF1
GH1	GDF10
GH2	GDF11
GHDC	GDF15
GHET1	GDF2
GHITM	GDF3
GHR	GDF5
GHRH	GDF5-AS1
GHRHR	GDF6
GHRL	GDF7
GID4	GDF9
GID8	GDI1
GIGYF1	GDI2
GIGYF2	GDNF
GIMAP1	GDPD1
GIMAP2	GDPD2
GIMAP3	GDPD3
GIMAP4	GDPD4
GIMAP5	GDPD5
GIMAP6	GDPGP1
GIMAP7	GEM
GIMAP8	GEMIN2
GIN1	GEMIN4
GINM1	GEMIN5
GINS1	GEMIN6
GINS2	GEMIN7
GINS3	GEMIN8
GINS4	GEMIN8P4
GIP	GEN1
GIPC1	GET1

GIPC2	GET3
GIPR	GET4
GIT1	GFAP
GIT2	GFER
GJA1	GFI1
GJA10	GFM1
GJA1B	GFM2
GJA3	GFOD1
GJA4	GFOD1-AS1
GJA5	GFOD2
GJA8	GFPT1
GJA9B	GFPT2
GJB1	GFRA1
GJB2	GFRA2
GJB3	GFRA3
GJB4	GFRA4
GJB5	GFRAL
GJB6	GFUS
GJB7	GGA1
GJB8	GGA2
GJC1	GGA3
GJC2	GGACT
GJC3	GGCT
GJD2	GGCTB
GJD3	GGCX
GJD4	GGH
GJE1	GGN
GK	GGNBP2
GK2	GGPS1
GK5	GGT1
GK6P	GGT2P
GKAP1	GGT3P
GKN1	GGT5
GKN3	GGT6
GLA	GGT7
GLAZ	GGT8P

GLB1	GGTA1
GLB1L	GGTLC1
GLB1L2	GGTLC2
GLB1L3	GGTLC3
GLCCI1	GH1
GLCCI1-DT	GH2
GLCE	GHDC
GLDC	GHET1
GLDN	GHITM
GLE1	GHR
GLG1	GHRH
GLI1	GHRHR
GLI2	GHRL
GLI3	GHSR
GLI4	GID4
GLIDR	GID8
GLIPR1	GIGYF1
GLIPR1L1	GIGYF2
GLIPR1L2	GIMAP1
GLIPR2	GIMAP2
GLIS1	GIMAP3
GLIS2	GIMAP4
GLIS3	GIMAP5
GLIS3-AS1	GIMAP6
GLMN	GIMAP7
GLMP	GIMAP8
GLO1	GIN1
GLOD4	GINM1
GLOD5	GINS1
GLP1R	GINS2
GLP2R	GINS3
GLRA1	GINS4
GLRA2	GIP
GLRA3	GIPC1
GLRB	GIPC2
GLRX	GIPR

GLRX2	GIT1
GLRX3	GIT2
GLRX5	GJA1
GLRXP3	GJA10
GLS	GJA1B
GLS2	GJA3
GLT1D1	GJA4
GLT28D2	GJA5
GLT6D1	GJA8
GLT8D1	GJA9B
GLT8D2	GJB1
GLTP	GJB2
GLTPD2	GJB3
GLTPP1	GJB4
GLUD1	GJB5
GLUD2	GJB6
GLUL	GJB7
GLYAT	GJB8
GLYATL1	GJC1
GLYATL2	GJC2
GLYCAM1	GJC3
GLYCTK	GJD2
GLYP	GJD3
GLYR1	GJD4
GM2A	GJE1
GMCL1	GK
GMCL2	GK2
GMD-2	GK5
GMDS	GK6P
GMDS-DT	GKAP1
GMEB1	GKN1
GMEB2	GKN3
GMFB	GLA
GMFG	GLAZ
GMIP	GLB1
GML	GLB1L

GML2	GLB1L2
GMNC	GLB1L3
GMNN	GLCCI1
GMPPA	GLCCI1-DT
GMPPB	GLCE
GMPR	GLDC
GMPR2	GLDN
GMPS	GLE1
GNA11	GLG1
GNA11B	GLI1
GNA12	GLI2
GNA13	GLI3
GNA14	GLI4
GNA15	GLIDR
GNAI1	GLIPR1
GNAI2	GLIPR1L1
GNAI3	GLIPR1L2
GNAL	GLIPR2
GNAO1	GLIS1
GNAO1.S	GLIS2
GNAQ	GLIS3
GNAS	GLIS3-AS1
GNAS-AS1	GLMN
GNAT1	GLMP
GNAT2	GLO1
GNAT3	GLOD4
GNAZ	GLOD5
GNB1	GLP1R
GNB1L	GLP2R
GNB2	GLRA1
GNB3	GLRA2
GNB4	GLRA3
GNB5	GLRB
GNE	GLRX
GNG10	GLRX2
GNG11	GLRX3

GNG12	GLRX5
GNG12-AS1	GLRXP3
GNG13	GLS
GNG14	GLS2
GNG2	GLT1D1
GNG3	GLT28D2
GNG4	GLT6D1
GNG5	GLT8D1
GNG7	GLT8D2
GNG8	GLTP
GNGT1	GLTPD2
GNGT2	GLTPP1
GNL1	GLUD1
GNL2	GLUD2
GNL3	GLUL
GNL3L	GLYAT
GNL3LP1	GLYATL1
GNLY	GLYATL2
GNMT	GLYCAM1
GNPAT	GLYCTK
GNPDA1	GLYP
GNPDA2	GLYR1
GNPNAT1	GM2A
GNPTAB	GMCL1
GNPTG	GMCL2
GNRH1	GMD-2
GNRH2	GMDS
GNRHR	GMDS-DT
GNRHR2	GMEB1
GNS	GMEB2
GOLGA1	GMFB
GOLGA2	GMFG
GOLGA2P11	GMIP
GOLGA2P5	GML
GOLGA3	GML2
GOLGA4	GMNC

GOLGA5	GMNN
GOLGA6B	GMPPA
GOLGA6C	GMPPB
GOLGA6L6	GMPR
GOLGA7	GMPR2
GOLGA7B	GMPS
GOLGA7B-DT	GNA11
GOLGA8A	GNA11B
GOLGA8B	GNA12
GOLGA8H	GNA13
GOLGA8IP	GNA14
GOLGA8N	GNA15
GOLGA8O	GNAI1
GOLGA8R	GNAI2
GOLGA8S	GNAI3
GOLGB1	GNAL
GOLIM4	GNAO1
GOLM1	GNAO1.S
GOLM2	GNAQ
GOLPH3	GNAS
GOLPH3L	GNAS-AS1
GOLT1A	GNAT1
GOLT1B	GNAT2
GON4L	GNAT3
GON7	GNAZ
GOPC	GNB1
GORAB	GNB1L
GORASP1	GNB2
GORASP2	GNB3
GOSR1	GNB4
GOSR2	GNB5
GOT1	GNE
GOT1L1	GNG10
GOT2	GNG11
GOT2P1	GNG12
GP1BA	GNG12-AS1

GP1BB
GP2
GP5
GP6
GPA33
GPAA1
GPALPP1
GPAM
GPANK1
GPAT2
GPAT3
GPAT4
GPATCH1
GPATCH11
GPATCH2
GPATCH2L
GPATCH3
GPATCH4
GPATCH8
GPBAR1
GPBP1
GPBP1L1
GPC1
GPC2
GPC3
GPC4
GPC5
GPC6
GPCPD1
GPD1
GPD1L
GPD2
GPDH1
GPER1
GPHB5
GPHN

GNG13
GNG14
GNG2
GNG3
GNG4
GNG5
GNG7
GNG8
GNGT1
GNGT2
GNL1
GNL2
GNL3
GNL3L
GNL3LP1
GNLY
GNMT
GNPAT
GNPDA1
GNPDA2
GNPNAT1
GNPTAB
GNPTG
GNRH1
GNRH2
GNRHR
GNRHR2
GNS
GOLGA1
GOLGA2
GOLGA2P11
GOLGA2P5
GOLGA3
GOLGA4
GOLGA5
GOLGA6B

GPI	GOLGA6C
GPIA	GOLGA6L6
GPIHBP1	GOLGA7
GPKOW	GOLGA7B
GPLD1	GOLGA7B-DT
GPM6A	GOLGA8A
GPM6AA	GOLGA8B
GPM6B	GOLGA8H
GPN1	GOLGA8IP
GPN2	GOLGA8N
GPN3	GOLGA8O
GPNMB	GOLGA8R
GPR1	GOLGA8S
GPR107	GOLGB1
GPR108	GOLIM4
GPR119	GOLM1
GPR12	GOLM2
GPR132	GOLPH3
GPR135	GOLPH3L
GPR137	GOLT1A
GPR137B	GOLT1B
GPR137C	GON4L
GPR139	GON7
GPR141	GOPC
GPR142	GORAB
GPR143	GORASP1
GPR146	GORASP2
GPR148	GOSR1
GPR149	GOSR2
GPR15	GOT1
GPR150	GOT1L1
GPR152	GOT2
GPR153	GOT2P1
GPR155	GP1BA
GPR156	GP1BB
GPR157	GP2

GPR158	GP5
GPR158-AS1	GP6
GPR15LG	GPA33
GPR160	GPAA1
GPR161	GPALPP1
GPR162	GPAM
GPR17	GPANK1
GPR171	GPAT2
GPR173	GPAT3
GPR174	GPAT4
GPR176	GPATCH1
GPR179	GPATCH11
GPR18	GPATCH2
GPR180	GPATCH2L
GPR182	GPATCH3
GPR183	GPATCH4
GPR19	GPATCH8
GPR199P	GPBAR1
GPR20	GPBP1
GPR21	GPBP1L1
GPR22	GPC1
GPR25	GPC2
GPR26	GPC3
GPR27	GPC4
GPR3	GPC5
GPR31	GPC6
GPR32	GPCPD1
GPR33	GPD1
GPR34	GPD1L
GPR35	GPD2
GPR37	GPDH1
GPR37L1	GPER1
GPR39	GPHB5
GPR4	GPHN
GPR42	GPI
GPR45	GPIA

GPR50	GPIHBP1
GPR55	GPKOW
GPR6	GPLD1
GPR62	GPM6A
GPR63	GPM6AA
GPR65	GPM6B
GPR68	GPN1
GPR75	GPN2
GPR75-ASB3	GPN3
GPR78	GPNMB
GPR83	GPR1
GPR84	GPR107
GPR85	GPR108
GPR87	GPR119
GPR88	GPR12
GPR89	GPR132
GPR89A	GPR135
GPR89B	GPR137
GPRASP1	GPR137B
GPRASP2	GPR137C
GPRC5A	GPR139
GPRC5B	GPR141
GPRC5C	GPR142
GPRC5D	GPR143
GPRIN1	GPR146
GPRIN2	GPR148
GPRIN3	GPR149
GPS1	GPR15
GPS2	GPR150
GPSM1	GPR151
GPSM2	GPR152
GPSM3	GPR153
GPT	GPR155
GPT2	GPR156
GPX1	GPR157
GPX1A	GPR158

GPX1B	GPR158-AS1
GPX2	GPR15LG
GPX3	GPR160
GPX4	GPR161
GPX5	GPR162
GPX6	GPR166P
GPX7	GPR17
GPX8	GPR171
GRAMD1A	GPR173
GRAMD1B	GPR174
GRAMD1C	GPR176
GRAMD2A	GPR179
GRAMD2B	GPR18
GRAMD3	GPR180
GRAMD4	GPR182
GRAP	GPR183
GRAP2	GPR19
GRASLND	GPR199P
GRB10	GPR20
GRB14	GPR21
GRB2	GPR22
GRB7	GPR25
GREB1	GPR26
GREB1L	GPR27
GREM1	GPR3
GREM2	GPR31
GRHL1	GPR32
GRHL2	GPR33
GRHL3	GPR34
GRHPR	GPR35
GRIA1	GPR37
GRIA2	GPR37L1
GRIA3	GPR39
GRIA4	GPR4
GRID1	GPR42
GRID2	GPR45

GRID2IP	GPR50
GRIK1	GPR55
GRIK2	GPR6
GRIK3	GPR62
GRIK4	GPR63
GRIK5	GPR65
GRIN1	GPR68
GRIN2A	GPR75
GRIN2B	GPR75-ASB3
GRIN2C	GPR78
GRIN2D	GPR83
GRIN3A	GPR84
GRIN3B	GPR85
GRINA	GPR87
GRIP1	GPR88
GRIP2	GPR89
GRIPAP1	GPR89A
GRK1	GPR89B
GRK2	GPRASP1
GRK3	GPRASP2
GRK4	GPRC5A
GRK5	GPRC5B
GRK6	GPRC5C
GRK7	GPRC5D
GRM1	GPRC6A
GRM2	GPRIN1
GRM3	GPRIN2
GRM4	GPRIN3
GRM5	GPS1
GRM6	GPS2
GRM7	GPSM1
GRM8	GPSM2
GRN	GPSM3
GRP	GPT
GRPEL1	GPT2
GRPEL2	GPX1

GRPR	GPX1A
GRTP1	GPX1B
GRWD1	GPX2
GSAP	GPX3
GSC	GPX4
GSC2	GPX5
GSDMA	GPX6
GSDMB	GPX7
GSDMC	GPX8
GSDMC3	GRAMD1A
GSDMD	GRAMD1B
GSDME	GRAMD1C
GSDMEB	GRAMD2A
GSE1	GRAMD2B
GSEC	GRAMD3
GSG1	GRAMD4
GSG1L	GRAP
GSK3A	GRAP2
GSK3B	GRASLND
GSN	GRB10
GSN-AS1	GRB14
GSP-4	GRB2
GSPT1	GRB7
GSPT2	GREB1
GSR	GREB1L
GSS	GREM1
GST-10	GREM2
GST-12	GRHL1
GST-13	GRHL2
GST-30	GRHL3
GST-38	GRHPR
GST-39	GRIA1
GST-4	GRIA2
GST-5	GRIA3
GST-6	GRIA4
GST-7	GRID1

GSTA	GRID2
GSTA1	GRID2IP
GSTA2	GRIK1
GSTA3	GRIK2
GSTA4	GRIK3
GSTA5	GRIK4
GSTCD	GRIK5
GSTD1	GRIN1
GSTD2	GRIN2A
GSTE1	GRIN2B
GSTE3	GRIN2C
GSTE9	GRIN2D
GSTK1	GRIN3A
GSTM1	GRIN3B
GSTM2	GRINA
GSTM3	GRIP1
GSTM4	GRIP2
GSTM5	GRIPAP1
GSTM6	GRK1
GSTM7	GRK2
GSTO1	GRK3
GSTO2	GRK4
GSTP1	GRK5
GSTP2	GRK6
GSTP3	GRK7
GSTT1	GRM1
GSTT1A	GRM2
GSTT1B	GRM3
GSTT2	GRM4
GSTT2B	GRM5
GSTT3	GRM6
GSTT4	GRM7
GSTTP2	GRM8
GSTZ1	GRN
GSX1	GRP
GSX2	GRPEL1

GT(ROSA)26SOR	GRPEL2
GTDC1	GRPR
GTF2A1	GRTP1
GTF2A1L	GRWD1
GTF2A2	GSAP
GTF2B	GSC
GTF2E1	GSC2
GTF2E2	GSDMA
GTF2F1	GSDMB
GTF2F2	GSDMC
GTF2H1	GSDMC3
GTF2H2B	GSDMD
GTF2H2C_2	GSDME
GTF2H3	GSDMEB
GTF2H4	GSE1
GTF2H5	GSEC
GTF2I	GSG1
GTF2I-AS1	GSG1L
GTF2IRD1	GSK3A
GTF2IRD2	GSK3B
GTF2IRD2B	GSN
GTF3A	GSN-AS1
GTF3C1	GSP-4
GTF3C2	GSPT1
GTF3C2-AS1	GSPT2
GTF3C3	GSR
GTF3C4	GSS
GTF3C5	GST-10
GTF3C6	GST-12
GTPBP1	GST-13
GTPBP10	GST-30
GTPBP2	GST-38
GTPBP3	GST-39
GTPBP4	GST-4
GTPBP6	GST-5
GTPBP8	GST-6

GTSE1	GST-7
GTSE1-DT	GSTA
GTSF1	GSTA1
GTSF1L	GSTA2
GUCA1A	GSTA3
GUCA1B	GSTA4
GUCA1C	GSTA5
GUCA1D	GSTCD
GUCA2A	GSTD1
GUCD1	GSTD2
GUCY1A1	GSTE1
GUCY1A2	GSTE3
GUCY1A3	GSTE9
GUCY1B1	GSTK1
GUCY1B2	GSTM1
GUCY2C	GSTM2
GUCY2D	GSTM3
GUCY2EP	GSTM4
GUCY2F	GSTM5
GUCY2G	GSTM6
GUF1	GSTM7
GUK1	GSTO1
GULO	GSTO2
GULP1	GSTP1
GUSB	GSTP2
GUSBP1	GSTP3
GUSBP11	GSTT1
GUSBP14	GSTT1A
GUSBP2	GSTT1B
GUSBP3	GSTT2
GUSBP4	GSTT2B
GVIN1	GSTT3
GVINP1	GSTT4
GVQW3	GSTTP2
GXIVSPLA2	GSTZ1
GXYLT1	GSX1

GXYLT2	GSX2
GYG	GT(ROSA)26SOR
GYG1	GTDC1
GYG2	GTF2A1
GYLTL1B	GTF2A1L
GYPA	GTF2A2
GYPB	GTF2B
GYPC	GTF2E1
GYS1	GTF2E2
GYS2	GTF2F1
GZF1	GTF2F2
GZMA	GTF2H1
GZMB	GTF2H2B
GZMD	GTF2H2C_2
GZMH	GTF2H3
GZMK	GTF2H4
GZMM	GTF2H5
GZMN	GTF2I
H1-0	GTF2I-AS1
H1-0.L	GTF2IRD1
H1-1	GTF2IRD2
H1-10	GTF2IRD2B
H1-10.S	GTF3A
H1-10-AS1	GTF3C1
H1-2	GTF3C2
H13	GTF3C2-AS1
H1-3	GTF3C3
H1-4	GTF3C4
H1-5	GTF3C5
H1-6	GTF3C6
H1-8	GTPBP1
H19	GTPBP10
H1-9P	GTPBP2
H1F0	GTPBP3
H1F1	GTPBP4
H1F10	GTPBP6

H1F2	GTPBP8
H1F6	GTSE1
H1F8	GTSE1-DT
H2-AA	GTSF1
H2AB1	GTSF1L
H2-AB1	GUCA1A
H2AB2	GUCA1B
H2AB3	GUCA1C
H2AC1	GUCA1D
H2AC11	GUCA2A
H2AC12	GUCD1
H2AC13	GUCY1A1
H2AC14	GUCY1A2
H2AC15	GUCY1A3
H2AC17	GUCY1B1
H2AC18	GUCY1B2
H2AC19	GUCY2C
H2AC20	GUCY2D
H2AC21	GUCY2EP
H2AC23	GUCY2F
H2AC25	GUCY2G
H2AC4	GUF1
H2AC6	GUK1
H2AC7	GULO
H2AC8	GULP1
H2AFX	GUSB
H2AJ	GUSBP1
H2AJ.L	GUSBP11
H2AX	GUSBP14
H2AZ1	GUSBP2
H2AZ2	GUSBP3
H2B	GUSBP4
H2BC1	GVIN1
H2BC10	GVINP1
H2BC11	GVQW3
H2BC12	GXIVSPLA2

H2BC12L	GXYLT1
H2BC13	GXYLT2
H2BC14	GYG
H2BC15	GYG1
H2BC17	GYG2
H2BC18	GYLTL1B
H2BC20P	GYPA
H2BC21	GYPB
H2BC26	GYPC
H2BC3	GYS1
H2BC4	GYS2
H2BC5	GZF1
H2BC6	GZMA
H2BC7	GZMB
H2BC8	GZMD
H2BC9	GZMH
H2-BL	GZMK
H2BP1	GZMM
H2BU2	GZMN
H2BW1	H1-0
H2BW2	H1-0.L
H2BW4P	H1-1
H2-D1	H1-10
H2-DMA	H1-10.S
H2-DMB1	H1-10-AS1
H2-EA	H1-2
H2-EB1	H13
H2-EB2	H1-3
H2-K1	H1-4
H2-K2	H1-5
H2-KE6	H1-6
H2-M10.1	H1-8
H2-M10.4	H19
H2-M2	H1-9P
H2-M3	H1F0
H2-OA	H1F1

H2-OB	H1F10
H2-Q1	H1F2
H2-Q10	H1F6
H2-Q2	H1F8
H2-Q6	H2-AA
H2-Q7	H2AB1
H2-Q8	H2-AB1
H2-T10	H2AB2
H2-T23	H2AB3
H2-T24	H2AC1
H2-T3	H2AC11
H3-3A	H2AC12
H3-3B	H2AC13
H3-4	H2AC14
H3-7	H2AC15
H3C1	H2AC17
H3C10	H2AC18
H3C11	H2AC19
H3C12	H2AC20
H3C13	H2AC21
H3C14	H2AC23
H3C14.L	H2AC25
H3C15	H2AC4
H3C2	H2AC6
H3C3	H2AC7
H3C4	H2AC8
H3C6	H2AFX
H3C7	H2AJ
H3C8	H2AJ.L
H3C8.S	H2AW
H3F3A	H2AX
H3F3B	H2AZ1
H3F3C	H2AZ2
H3F4	H2B
H3P6	H2BC1
H4C1	H2BC10

H4C11	H2BC11
H4C12	H2BC12
H4C13	H2BC12L
H4C14	H2BC13
H4C15	H2BC14
H4C16	H2BC15
H4C2	H2BC17
H4C3	H2BC18
H4C4	H2BC20P
H4C5	H2BC21
H4C6	H2BC26
H4C8	H2BC3
H4C9	H2BC4
H4F16	H2BC5
H60A	H2BC6
H6PD	H2BC7
HAAO	H2BC8
HABP2	H2BC9
HABP4	H2-BL
HACD1	H2BP1
HACD2	H2BU2
HACD3	H2BW1
HACD4	H2BW2
HACE1	H2BW4P
HACL1	H2-D1
HADH	H2-DMA
HADHA	H2-DMB1
HADHB	H2-EA
HAGH	H2-EB1
HAGHL	H2-EB2
HAGLR	H2-K1
HAL	H2-K2
HAMP	H2-KE6
HAMP2	H2-M10.1
HAND1	H2-M10.4
HAND2	H2-M2

HAO1	H2-M3
HAO2	H2-OA
HAP1	H2-OB
HAPLN1	H2-Q1
HAPLN1A	H2-Q10
HAPLN2	H2-Q2
HAPLN3	H2-Q6
HAPLN4	H2-Q7
HAR1A	H2-Q8
HAR1B	H2-T10
HARBI1	H2-T23
HARS	H2-T24
HARS1	H2-T3
HARS2	H3-3A
HAS1	H3-3B
HAS2	H3-4
HAS2-AS1	H3-7
HAS3	H3C1
HASPIN	H3C10
HAS-RS.S	H3C11
HAT1	H3C12
HAUS1	H3C13
HAUS2	H3C14
HAUS3	H3C14.L
HAUS4	H3C15
HAUS5	H3C2
HAUS6	H3C3
HAUS7	H3C4
HAUS8	H3C6
HAVCR1	H3C7
HAVCR1P1	H3C8
HAVCR2	H3C8.S
HAX1	H3F3A
HBA	H3F3B
HBA1	H3F3C
HBA2	H3F4

HBA4	H3P10
HBA-A1	H3P12
HBA-A2	H3P23
HBAE3	H3P28
HBA-X	H3P40
HBB	H3P41
HBB-B1	H3P6
HBB-B2	H3P9
HBB-BH1	H4C1
HBB-BS	H4C11
HBBE1.1	H4C12
HBBE2	H4C13
HBBE3	H4C14
HBB-Y	H4C15
HBD	H4C16
HBE1	H4C2
HBEGF	H4C3
HBG1	H4C4
HBG2	H4C5
HBM	H4C6
HBP1	H4C8
HBQ1	H4C9
HBQ1B	H4F16
HBS1L	H60A
HBZ	H6PD
HC	HAAO
HCAR1	HABP2
HCAR2	HABP4
HCAR3	HACD1
HCCAT5	HACD2
HCCS	HACD3
HCFC1	HACD4
HCFC1A	HACE1
HCFC1B	HACL1
HCFC1R1	HADH
HCFC2	HADHA

HCG11	HADHB
HCG14	HAGH
HCG18	HAGHL
HCG22	HAGLR
HCG23	HAL
HCG25	HAMP
HCG27	HAMP2
HCG4	HAND1
HCK	HAND2
HCLS1	HAND2-AS1
HCN1	HAO1
HCN2	HAO2
HCN3	HAP1
HCN4	HAPLN1
HCP5	HAPLN1A
HCRT	HAPLN2
HCRTR1	HAPLN3
HCRTR2	HAPLN4
HCST	HAR1A
HDA-1	HAR1B
HDAC1	HARBI1
HDAC10	HARS
HDAC11	HARS1
HDAC2	HARS2
HDAC3	HAS1
HDAC4	HAS2
HDAC4-AS1	HAS2-AS1
HDAC5	HAS3
HDAC6	HASPIN
HDAC7	HAS-RS.S
HDAC8	HAT1
HDAC9	HAUS1
HDAC9B	HAUS2
HDC	HAUS3
HDDC2	HAUS4
HDDC3	HAUS5

HDGF
HDGFL1
HDGFL2
HDGFL3
HDHD2
HDHD3
HDHD5
HDLBP
HDR
HDX
HE1.3
HEATR1
HEATR2
HEATR3
HEATR4
HEATR5A
HEATR5B
HEATR6
HEBP1
HEBP2
HECA
HECTD1
HECTD2
HECTD2-AS1
HECTD3
HECTD4
HECW1
HECW2
HECW2A
HEG1
HELB
HELLS
HELQ
HELT
HELZ
HELZ2
HAUS6
HAUS7
HAUS8
HAVCR1
HAVCR1P1
HAVCR2
HAX1
HBA
HBA1
HBA2
HBA4
HBA-A1
HBA-A2
HBAE3
HBA-X
HBB
HBB-B1
HBB-B2
HBB-BH1
HBB-BS
HBBE1.1
HBBE2
HBBE3
HBB-Y
HBD
HBE1
HBEGF
HBG1
HBG2
HBM
HBP1
HBQ1
HBQ1B
HBS1L
HBZ
HC

HEMGN	HCAR1
HEMK1	HCAR2
HENMT1	HCAR3
HEPACAM	HCCAT5
HEPACAM2	HCCS
HEPH	HCFC1
HER15.2	HCFC1A
HER2	HCFC1B
HER3	HCFC1R1
HER4.5	HCFC2
HER8A	HCG11
HER9	HCG14
HERC1	HCG18
HERC2	HCG22
HERC2P2	HCG23
HERC2P4	HCG25
HERC2P9	HCG27
HERC3	HCG4
HERC4	HCK
HERC5	HCLS1
HERC6	HCN1
HERPUD1	HCN2
HERPUD2	HCN3
HES1	HCN4
HES2	HCP5
HES3	HCRT
HES4	HCRTR1
HES5	HCRTR2
HES6	HCST
HES7	HDA-1
HES7.1.L	HDAC1
HESX1	HDAC10
HEXA	HDAC11
HEXA-AS1	HDAC2
HEXB	HDAC3
HEXD	HDAC4

HEXDC	HDAC4-AS1
HEXIM1	HDAC5
HEXIM2	HDAC6
HEXO1	HDAC7
HEY1	HDAC8
HEY2	HDAC9
HEYL	HDAC9B
HFE	HDC
HFM1	HDDC2
HGC6.3	HDDC3
HGD	HDGF
HGF	HDGFL1
HGFAC	HDGFL2
HGH1	HDGFL3
HGS	HDHD2
HGSNAT	HDHD3
HHAT	HDHD5
HHATL	HDLBP
HHATL-AS1	HDR
HHATLB	HDX
HHEX	HE1.3
HHIP	HEATR1
HHIP-AS1	HEATR2
HHIPL1	HEATR3
HHIPL2	HEATR4
HHLA2	HEATR5A
HHLA3	HEATR5B
HIBADH	HEATR6
HIBCH	HEBP1
HIC1	HEBP2
HIC2	HECA
HID1	HECTD1
HIF1A	HECTD2
HIF1A-AS2	HECTD2-AS1
HIF1AN	HECTD3
HIF3A	HECTD4

HIGD1A	HECW1
HIGD1B	HECW2
HIGD1C	HECW2A
HIGD2A	HEG1
HIKESHI	HEIH
HILPDA	HELB
HINFP	HELLS
HINT1	HELQ
HINT2	HELT
HINT3	HELZ
HIP1	HELZ2
HIP1R	HEMGN
HIPK1	HEMK1
HIPK1-AS1	HENMT1
HIPK2	HEPACAM
HIPK3	HEPACAM2
HIPK3A	HEPH
HIPK4	HER15.2
HIRA	HER2
HIRIP3	HER3
HIS-26	HER4.5
HIS-38	HER8A
HIS-64	HER9
HIS-67	HERC1
HIST1H2AC	HERC2
HIST1H2AF	HERC2P2
HIST1H2BC	HERC2P4
HIST1H2BD	HERC2P9
HIST1H2BG	HERC3
HIST1H2BQ	HERC4
HIST1H3H	HERC5
HIST1H4B	HERC6
HIST2H2AA2	HERPUD1
HIST2H2L	HERPUD2
HIST3H2A	HES1
HIST3H2BA	HES2

HIVEP1	HES3
HIVEP2	HES4
HIVEP3	HES5
HJURP	HES6
HJV	HES7
HK	HES7.1.L
HK1	HESX1
HK2	HEXA
HK2P1	HEXA-AS1
HK3	HEXB
HKDC1	HEXD
HLA-A	HEXDC
HLA-B	HEXIM1
HLA-C	HEXIM2
HLA-DMA	HEXO1
HLA-DMB	HEY1
HLA-DOA	HEY2
HLA-DOB	HEYL
HLA-DPA1	HFE
HLA-DPA3	HFM1
HLA-DPB1	HGC6.3
HLA-DPB2	HGD
HLA-DQA1	HGF
HLA-DQA2	HGFAC
HLA-DQB1	HGH1
HLA-DQB2	HGS
HLA-DRA	HGSNAT
HLA-DRB1	HHAT
HLA-DRB4	HHATL
HLA-DRB5	HHATL-AS1
HLA-DRB6	HHATLB
HLA-E	HHEX
HLA-F	HHIP
HLA-F-AS1	HHIP-AS1
HLA-G	HHIPL1
HLA-H	HHIPL2

HLA-J	HHLA2
HLA-V	HHLA3
HLCS	HIBADH
HLF	HIBCH
HLH-14	HIC1
HLTF	HIC2
HLX	HID1
HM13	HIF1A
HMBOX1	HIF1A-AS1
HMBS	HIF1A-AS2
HMCES	HIF1AN
HMCN1	HIF3A
HMCN2	HIGD1A
HMG1	HIGD1B
HMG-1.2	HIGD1C
HMG20A	HIGD2A
HMG20B	HIKESHI
HMGA1	HILPDA
HMGA1B	HINFP
HMGA2	HINT1
HMGA2-PS1	HINT2
HMGB1	HINT3
HMGB1A	HIP1
HMGB1P1	HIP1R
HMGB1P4	HIPK1
HMGB2	HIPK1-AS1
HMGB3	HIPK2
HMGB3A	HIPK3
HMGB3P1	HIPK3A
HMGCL	HIPK4
HMGCLL1	HIRA
HMGCR	HIRIP3
HMGCRA	HIS-26
HMGCS1	HIS-38
HMGCS2	HIS-64
HMGN1	HIS-67

HMGN2	HIST1H2AC
HMGN3	HIST1H2AF
HMGN3-AS1	HIST1H2BC
HMGN5	HIST1H2BD
HMGN5B	HIST1H2BG
HMGN6	HIST1H2BQ
HMGXB3	HIST1H3H
HMGXB4	HIST1H4B
HMHB1	HIST2H2AA2
HMMR	HIST2H2L
HMOX1	HIST3H2A
HMOX1A	HIST3H2BA
HMOX2	HIVEP1
HMSD	HIVEP2
HMX1	HIVEP3
HMX2	HJURP
HMX3	HJV
HN	HK
HN1	HK1
HN1L	HK2
HNF1A	HK2P1
HNF1A-AS1	HK3
HNF1B	HKDC1
HNF4A	HLA-A
HNF4G	HLA-B
HNMT	HLA-C
HNRNPA0	HLA-DMA
HNRNPA1	HLA-DMB
HNRNPA1L2	HLA-DOA
HNRNPA1P12	HLA-DOB
HNRNPA1P7	HLA-DPA1
HNRNPA2B1	HLA-DPA3
HNRNPA3	HLA-DPB1
HNRNPAB	HLA-DPB2
HNRNPC	HLA-DQA1
HNRNPCL1	HLA-DQA2

HNRNPD	HLA-DQB1
HNRNPDL	HLA-DQB2
HNRNPF	HLA-DRA
HNRNPH1	HLA-DRB1
HNRNPH2	HLA-DRB4
HNRNPH3	HLA-DRB5
HNRNPK	HLA-DRB6
HNRNPL	HLA-E
HNRNPLL	HLA-F
HNRNPM	HLA-F-AS1
HNRNPR	HLA-G
HNRNPU	HLA-H
HNRNPUL1	HLA-J
HNRNPUL2	HLA-V
HOATZ	HLCS
HOGA1	HLF
HOMER1	HLH-14
HOMER2	HLTF
HOMER3	HLX
HOMEZ	HM13
HOOK1	HMBOX1
HOOK2	HMBS
HOOK3	HMCES
HOPX	HMCN1
HORMAD1	HMCN2
HORMAD2	HMG1
HOTAIR	HMG-1.2
HOTAIRM1	HMG20A
HOTTIP	HMG20B
HOXA1	HMGA1
HOXA10	HMGA1B
HOXA10B	HMGA2
HOXA11	HMGA2-PS1
HOXA11-AS	HMGB1
HOXA11OS	HMGB1A
HOXA13	HMGB1P1

HOXA2	HMGB1P4
HOXA3	HMGB2
HOXA5	HMGB3
HOXA6	HMGB3A
HOXA7	HMGB3P1
HOXA9	HMGB4
HOXA-AS2	HMGCL
HOXB1	HMGCLL1
HOXB13	HMGCR
HOXB1A	HMGCRA
HOXB2	HMGCS1
HOXB3	HMGCS2
HOXB4	HMGN1
HOXB5	HMGN2
HOXB5B	HMGN3
HOXB6	HMGN3-AS1
HOXB7	HMGN5
HOXB8	HMGN5B
HOXB8A	HMGN6
HOXB9	HMGXB3
HOXB-AS1	HMGXB4
HOXB-AS3	HMHB1
HOXC10	HMMR
HOXC11	HMOX1
HOXC13	HMOX1A
HOXC13A	HMOX2
HOXC4	HMSD
HOXC4A	HMX1
HOXC5	HMX2
HOXC6	HMX3
HOXC8	HN
HOXC9	HN1
HOXC9A	HN1L
HOXD1	HNF1A
HOXD10	HNF1A-AS1
HOXD11	HNF1B

HOXD12	HNF4A
HOXD13	HNF4G
HOXD3	HNMT
HOXD4	HNRNPA0
HOXD8	HNRNPA1
HOXD9	HNRNPA1L2
HP	HNRNPA1P10
HP1BP3	HNRNPA1P12
HPCA	HNRNPA1P7
HPCAL1	HNRNPA2B1
HPCAL4	HNRNPA3
HPD	HNRNPAB
HPDL	HNRNPC
HPF1	HNRNPCL1
HPGD	HNRNPD
HPGDS	HNRNPDL
HPN	HNRNPF
HPN-AS1	HNRNPH1
HPR	HNRNPH2
HPRT	HNRNPH3
HPRT1	HNRNPK
HPS1	HNRNPL
HPS3	HNRNPLL
HPS4	HNRNPM
HPS5	HNRNPR
HPS6	HNRNPU
HPSE	HNRNPUL1
HPSE2	HNRNPUL2
HPX	HOATZ
HPYR1	HOGA1
HR	HOMER1
HRAS	HOMER2
HRC	HOMER3
HRCT1	HOMEZ
HRG	HOOK1
HRH1	HOOK2

HRH2	HOOK3
HRH3	HOPX
HRH4	HORMAD1
HRK	HORMAD2
HRNR	HOTAIR
HROB	HOTAIRM1
HS1BP3	HOTTIP
HS2ST1	HOXA@
HS3ST1	HOXA1
HS3ST2	HOXA10
HS3ST3A1	HOXA10B
HS3ST3B1	HOXA11
HS3ST4	HOXA11-AS
HS3ST5	HOXA11OS
HS3ST6	HOXA13
HS6ST1	HOXA2
HS6ST2	HOXA3
HS6ST3	HOXA4
HSBP1	HOXA5
HSBP1L1	HOXA6
HSCB	HOXA7
HSD11B1	HOXA9
HSD11B1-AS1	HOXA-AS2
HSD11B1L	HOXA-AS3
HSD11B2	HOXB1
HSD17B1	HOXB13
HSD17B10	HOXB1A
HSD17B11	HOXB2
HSD17B12	HOXB3
HSD17B13	HOXB4
HSD17B14	HOXB5
HSD17B2	HOXB5B
HSD17B3	HOXB6
HSD17B4	HOXB7
HSD17B6	HOXB8
HSD17B7	HOXB8A

HSD17B7P2	HOXB9
HSD17B8	HOXB-AS1
HSD3B	HOXB-AS3
HSD3B1	HOXC10
HSD3B2	HOXC11
HSD3B3	HOXC13
HSD3B4	HOXC13A
HSD3B5	HOXC4
HSD3B6	HOXC4A
HSD3B7	HOXC5
HSDL1	HOXC6
HSDL2	HOXC8
HSF1	HOXC9
HSF2	HOXC9A
HSF2BP	HOXD1
HSF4	HOXD10
HSFX2	HOXD11
HSH2D	HOXD12
HSP-16.1	HOXD13
HSP-16.2	HOXD3
HSP22	HOXD4
HSP23	HOXD8
HSP27	HOXD9
HSP47	HP
HSP68	HP1BP3
HSP70	HPC4
HSP70.3	HPCA
HSP70BBB	HPCAL1
HSP70BC	HPCAL4
HSP70L	HPD
HSP83	HPDL
HSP90AA1	HPF1
HSP90AA1.1	HPGD
HSP90AA2P	HPGDS
HSP90AB1	HPN
HSP90AB2P	HPN-AS1

HSP90AB5P	HPP1
HSP90B1	HPR
HSP90B2P	HPRT
HSPA12A	HPRT1
HSPA12B	HPS1
HSPA13	HPS3
HSPA14	HPS4
HSPA1A	HPS5
HSPA1B	HPS6
HSPA1L	HPSE
HSPA2	HPSE2
HSPA4	HPX
HSPA4L	HPYR1
HSPA5	HR
HSPA6	HRAS
HSPA7	HRC
HSPA8	HRCT1
HSPA9	HRG
HSPB1	HRH1
HSPB11	HRH2
HSPB2	HRH3
HSPB3	HRH4
HSPB6	HRK
HSPB7	HRNR
HSPB8	HROB
HSPB9	HS1BP3
HSPBAP1	HS2ST1
HSPBP1	HS3ST1
HSPD1	HS3ST2
HSPD1P5	HS3ST3A1
HSPD1P6	HS3ST3B1
HSPE1	HS3ST4
HSPE1P9	HS3ST5
HSPG2	HS3ST6
HSPH1	HS6ST1
HTATIP2	HS6ST2

HTATSF1	HS6ST3
HTN1	HSBP1
HTN3	HSBP1L1
HTR1A	HSCB
HTR1B	HSD11B1
HTR1D	HSD11B1-AS1
HTR1E	HSD11B1L
HTR1F	HSD11B2
HTR2A	HSD17B1
HTR2B	HSD17B10
HTR2C	HSD17B11
HTR3A	HSD17B12
HTR3B	HSD17B13
HTR3E	HSD17B14
HTR4	HSD17B2
HTR5A	HSD17B3
HTR5B	HSD17B4
HTR6	HSD17B6
HTR7	HSD17B7
HTR7P1	HSD17B7P2
HTRA1	HSD17B8
HTRA1B	HSD3B
HTRA2	HSD3B1
HTRA3	HSD3B2
HTT	HSD3B3
HUNK	HSD3B4
HUS1	HSD3B5
HUS1B	HSD3B6
HUWE1	HSD3B7
HVCN1	HSDL1
HYAL1	HSDL2
HYAL2	HSF1
HYAL3	HSF2
HYAL6P	HSF2BP
HYDIN	HSF4
HYI	HSFX2

HYKK	HSH2D
HYLS1	HSP-16.1
HYMAI	HSP-16.2
HYOU1	HSP22
HYPK	HSP23
IAH1	HSP27
IAPP	HSP47
IARS1	HSP68
IARS2	HSP70
IBA57	HSP70.3
IBSP	HSP70BBB
IBTK	HSP70BC
ICA1	HSP70L
ICA1L	HSP83
ICAL1	HSP90AA1
ICAM1	HSP90AA1.1
ICAM2	HSP90AA2P
ICAM3	HSP90AB1
ICAM4	HSP90AB2P
ICAM5	HSP90AB5P
ICE1	HSP90B1
ICE2	HSP90B2P
ICMT	HSPA12A
ICMT-DT	HSPA12B
ICN2	HSPA13
ICOS	HSPA14
ICOSL	HSPA1A
ICOSLG	HSPA1B
ID1	HSPA1L
ID2	HSPA2
ID2B	HSPA4
ID3	HSPA4L
ID4	HSPA5
IDE	HSPA6
IDGF1	HSPA7
IDGF2	HSPA8

IDH1	HSPA9
IDH1-AS1	HSPB1
IDH2	HSPB11
IDH3A	HSPB2
IDH3B	HSPB3
IDH3G	HSPB6
IDI1	HSPB7
IDI2	HSPB8
IDI2-AS1	HSPB9
IDNK	HSPBAP1
IDO1	HSPBP1
IDO2	HSPD1
IDS	HSPD1P5
IDSP1	HSPD1P6
IDUA	HSPE1
IER2	HSPE1P9
IER3	HSPG2
IER3IP1	HSPH1
IER5	HTATIP2
IER5L	HTATSF1
IFFO2	HTC2
IFI16	HTN1
IFI202B	HTN3
IFI203	HTR1A
IFI204	HTR1B
IFI205	HTR1D
IFI27	HTR1E
IFI27L1	HTR1F
IFI27L2	HTR2A
IFI27L2B	HTR2B
IFI30	HTR2C
IFI35	HTR3A
IFI44	HTR3B
IFI44L	HTR3E
IFI47	HTR4
IFI6	HTR5A

IFIH1	HTR5B
IFIT1	HTR6
IFIT2	HTR7
IFIT3	HTR7P1
IFIT5	HTRA1
IFITM1	HTRA1B
IFITM10	HTRA2
IFITM2	HTRA3
IFITM3	HTT
IFITM4P	HULC
IFITM5	HUNK
IFITM6	HUS1
IFITM8P	HUS1B
IFNA	HUWE1
IFNA1	HVCN1
IFNA14	HYAL1
IFNA2	HYAL2
IFNA21	HYAL3
IFNA9	HYAL6P
IFNAR1	HYDIN
IFNAR2	HYI
IFNB1	HYKK
IFNE	HYLS1
IFNG	HYMAI
IFNGR1	HYOU1
IFNGR2	HYPK
IFNK	IAH1
IFNL1	IAPP
IFNL2	IARS1
IFNL3	IARS2
IFNLR1	IATPR
IFNZ	IBA57
IFRD1	IBSP
IFRD2	IBTK
IFRG15	ICA1
IFT122	ICA1L

IFT140	ICAL1
IFT172	ICAM1
IFT20	ICAM2
IFT22	ICAM3
IFT27	ICAM4
IFT43	ICAM5
IFT46	ICE1
IFT52	ICE2
IFT57	ICMT
IFT74	ICMT-DT
IFT80	ICN2
IFT81	ICOS
IFT88	ICOSL
IFTAP	ICOSLG
IGBP1	ID1
IGD	ID2
IGDCC3	ID2B
IGDCC4	ID3
IGF1	ID4
IGF1R	IDE
IGF1RA	IDGF1
IGF2	IDGF2
IGF2-AS	IDH1
IGF2BP1	IDH1-AS1
IGF2BP2	IDH2
IGF2BP3	IDH3A
IGF2R	IDH3B
IGF3	IDH3G
IGFALS	IDI1
IGFBP1	IDI2
IGFBP1A	IDI2-AS1
IGFBP1B	IDNK
IGFBP2	IDO1
IGFBP2A	IDO2
IGFBP3	IDS
IGFBP4	IDSP1

IGFBP5	IDUA
IGFBP5B	IER2
IGFBP6	IER3
IGFBP7	IER3IP1
IGFBP7-AS1	IER5
IGFBPL1	IER5L
IGFL1	IFFO2
IGFL2	IFI16
IGFL2-AS1	IFI202B
IGFL3	IFI203
IGFLR1	IFI204
IGFN1	IFI205
IGFN1.1	IFI27
IGFN1.3	IFI27L1
IGG-2A	IFI27L2
IGH	IFI27L2B
IGH-6	IFI30
IGHA	IFI35
IGHA1	IFI44
IGHG	IFI44L
IGHG1	IFI47
IGHG2A	IFI6
IGHG2B	IFIH1
IGHG2C	IFIT1
IGHG3	IFIT2
IGHM	IFIT3
IGHMBP2	IFIT5
IGHV1-18	IFITM1
IGHV1-69	IFITM10
IGHV1-9	IFITM2
IGH-V3609N	IFITM3
IGHV3-8	IFITM4P
IGHV3-9	IFITM5
IGHV6-3	IFITM6
IGHV9-2	IFITM8P
IGH-VJ558	IFN1@

IGIP	IFNA
IGK	IFNA1
IGKC	IFNA13
IGK-V1	IFNA14
IGKV10-94	IFNA17
IGKV1-117	IFNA2
IGKV1-135	IFNA21
IGKV12-98	IFNA9
IGKV1-5	IFNAR1
IGKV15-103	IFNAR2
IGK-V28	IFNB1
IGKV3-10	IFNE
IGKV4-72	IFNG
IGKV4-86	IFNGR1
IGKV5-48	IFNGR2
IGKV6-14	IFNK
IGKV8-24	IFNL1
IGKV8-30	IFNL2
IGL	IFNL3
IGLC1	IFNLR1
IGLC2	IFNZ
IGLC3	IFRD1
IGLL1	IFRD2
IGLL5	IFRG15
IGLON5	IFT122
IGLV1	IFT140
IGLV1-47	IFT172
IGLV3-21	IFT20
IGLV3-25	IFT22
IGSF1	IFT27
IGSF10	IFT43
IGSF11	IFT46
IGSF21	IFT52
IGSF3	IFT57
IGSF5	IFT74
IGSF6	IFT80

IGSF8	IFT81
IGSF9	IFT88
IGSF9B	IFTAP
IGTP	IGBP1
IHH	IGD
IHO1	IGDCC3
IIGP1	IGDCC4
IK	IGF1
IKBIP	IGF1R
IKBKB	IGF1RA
IKBKE	IGF2
IKBKG	IGF2-AS
IKZF1	IGF2BP1
IKZF2	IGF2BP2
IKZF3	IGF2BP2-AS1
IKZF4	IGF2BP3
IKZF5	IGF2R
IL10	IGF3
IL10RA	IGFALS
IL10RB	IGFBP1
IL10RB-DT	IGFBP1A
IL11	IGFBP1B
IL11RA	IGFBP2
IL11RA1	IGFBP2A
IL11RA2	IGFBP3
IL12A	IGFBP4
IL12A-AS1	IGFBP5
IL12B	IGFBP5B
IL12RB1	IGFBP6
IL12RB2	IGFBP7
IL13	IGFBP7-AS1
IL13RA1	IGFBPL1
IL13RA2	IGFL1
IL15	IGFL2
IL15RA	IGFL2-AS1
IL16	IGFL3

IL17A	IGFLR1
IL17B	IGFN1
IL17C	IGFN1.1
IL17D	IGFN1.3
IL17F	IGG-2A
IL17RA	IGH
IL17RB	IGH-6
IL17RC	IGHA
IL17RD	IGHA1
IL17RE	IGHG
IL17REL	IGHG1
IL18	IGHG2A
IL18BP	IGHG2B
IL18R1	IGHG2C
IL18RAP	IGHG3
IL19	IGHM
IL1A	IGHMBP2
IL1B	IGHV1-18
IL1F10	IGHV1-69
IL1R1	IGHV1-9
IL1R2	IGH-V3609N
IL1RAP	IGHV3-8
IL1RAPL1	IGHV3-9
IL1RAPL2	IGHV6-3
IL1RL1	IGHV9-2
IL1RL2	IGH-VJ558
IL1RN	IGIP
IL2	IGK
IL20	IGKC
IL20RA	IGK-V1
IL20RB	IGKV10-94
IL21R	IGKV1-117
IL22	IGKV1-135
IL22RA1	IGKV12-98
IL22RA2	IGKV1-5
IL23A	IGKV15-103

IL23R	IGK-V28
IL24	IGKV3-10
IL25	IGKV4-72
IL26	IGKV4-86
IL27	IGKV5-48
IL27RA	IGKV6-14
IL2RA	IGKV8-24
IL2RB	IGKV8-30
IL2RG	IGL
IL3	IGLC1
IL31	IGLC2
IL31RA	IGLC3
IL32	IGLL1
IL33	IGLL5
IL34	IGLON5
IL36A	IGLV1
IL36B	IGLV1-47
IL36G	IGLV3-21
IL36RN	IGLV3-25
IL37	IGSF1
IL3RA	IGSF10
IL4	IGSF11
IL4I1	IGSF21
IL4R	IGSF3
IL4RA	IGSF5
IL5	IGSF6
IL5RA	IGSF8
IL6	IGSF9
IL6R	IGSF9B
IL6RA	IGTP
IL6R-AS1	IHH
IL6ST	IHO1
IL7	IIGP1
IL7R	IK
IL8L2	IKBIP
IL9	IKBKB

IL9R	IKBKE
ILDR1	IKBKG
ILDR2	IKZF1
ILF2	IKZF2
ILF3	IKZF3
ILF3-DT	IKZF4
ILK	IKZF5
ILKAP	IL10
ILP3	IL10RA
ILP5	IL10RB
ILRUN	IL10RB-DT
ILVBL	IL11
ILYS-5	IL11RA
IMMP1L	IL11RA1
IMMP2L	IL11RA2
IMMT	IL12A
IMP3	IL12A-AS1
IMP4	IL12B
IMPA1	IL12RB1
IMPA2	IL12RB2
IMPACT	IL13
IMPDH1	IL13RA1
IMPDH1B	IL13RA2
IMPDH2	IL15
IMPG1	IL15RA
IMPG2	IL16
IMPL2	IL17A
INA	IL17B
INAA	IL17C
INAB	IL17D
INAFM1	IL17F
INAFM2	IL17RA
INAVA	IL17RB
INCA1	IL17RC
INCENP	IL17RD
INCENP.S	IL17RE

INDO	IL17REL
INE1	IL18
INF2	IL18BP
ING1	IL18R1
ING2	IL18RAP
ING3	IL19
ING4	IL1A
ING5	IL1B
ING5B	IL1F10
INHA	IL1R1
INHBA	IL1R2
INHBA-AS1	IL1RAP
INHBB	IL1RAPL1
INHBC	IL1RAPL2
INHBE	IL1RL1
INHCA	IL1RL2
INIP	IL1RN
INKA1	IL2
INKA1A	IL20
INKA2	IL20RA
INKA2-AS1	IL20RB
INMT	IL21
INO80	IL21R
INO80B	IL22
INO80B-WBP1	IL22RA1
INO80C	IL22RA2
INO80D	IL23A
INO80E	IL23R
INPP1	IL24
INPP4A	IL25
INPP4B	IL26
INPP5A	IL27
INPP5B	IL27RA
INPP5D	IL2RA
INPP5E	IL2RB
INPP5F	IL2RG

INPP5J	IL3
INPP5K	IL31
INPPL1	IL31RA
INS	IL32
INS1	IL33
INS2	IL34
INSC	IL36A
INSIG1	IL36B
INSIG2	IL36G
INS-IGF2	IL36RN
INSL3	IL37
INSL4	IL3RA
INSL5	IL4
INSL6	IL4I1
INSM1	IL4R
INSM1B	IL4RA
INSM2	IL5
INSR	IL5RA
INSRR	IL6
INSYN1	IL6R
INSYN2A	IL6RA
INSYN2B	IL6R-AS1
INTS1	IL6ST
INTS10	IL7
INTS11	IL7R
INTS12	IL8L2
INTS13	IL9
INTS14	IL9R
INTS15	ILDR1
INTS2	ILDR2
INTS3	ILF2
INTS4	ILF3
INTS4P1	ILF3-DT
INTS4P2	ILK
INTS5	ILKAP
INTS6	ILP3

INTS6-AS1	ILP5
INTS6L	ILRUN
INTS7	ILVBL
INTS8	ILYS-5
INTS9	IMMP1L
INTU	IMMP2L
INVS	IMMT
IP6K1	IMP3
IP6K2	IMP4
IP6K3	IMPA1
IPCEF1	IMPA2
IPF1	IMPACT
IPMK	IMPDH1
IPO11	IMPDH1B
IPO13	IMPDH2
IPO4	IMPG1
IPO5	IMPG2
IPO5P1	IMPL2
IPO7	INA
IPO8	INAA
IPO9	INAB
IPP	INAFM1
IPPK	INAFM2
IPW	INAVA
IQANK1	INCA1
IQCA	INCENP
IQCA1	INCENP.S
IQCB1	INDO
IQCC	INE1
IQCD	INF2
IQCE	ING1
IQCF1	ING2
IQCF3	ING3
IQCF5	ING4
IQCF6	ING5
IQCG	ING5B

IQCH	INHA
IQCH-AS1	INHBA
IQCJ-SCHIP1	INHBA-AS1
IQCK	INHBB
IQCN	INHBC
IQGAP1	INHBE
IQGAP2	INHCA
IQGAP3	INIP
IQSEC1	INKA1
IQSEC2	INKA1A
IQSEC3	INKA2
IQUB	INKA2-AS1
IRAG1	INMT
IRAG2	INO80
IRAK1	INO80B
IRAK1BP1	INO80B-WBP1
IRAK2	INO80C
IRAK3	INO80D
IRAK4	INO80E
IRBP	INPP1
IREB2	INPP4A
IRF1	INPP4B
IRF2	INPP5A
IRF2BP1	INPP5B
IRF2BP2	INPP5D
IRF2BP2B	INPP5E
IRF2BPL	INPP5F
IRF3	INPP5J
IRF4	INPP5K
IRF5	INPPL1
IRF6	INS
IRF7	INS1
IRF8	INS2
IRF9	INSC
IRG-1	INSIG1
IRG-5	INSIG2

IRG-6	INS-IGF2
IRGC	INSL3
IRGC1	INSL4
IRGM	INSL5
IRGM1	INSL6
IRGM2	INSM1
IRGQ	INSM1B
IRIS	INSM2
IRS1	INSR
IRS2	INSRR
IRS3	INSYN1
IRS4	INSYN2A
IRX1	INSYN2B
IRX2	INTS1
IRX2-DT	INTS10
IRX3	INTS11
IRX4	INTS12
IRX5	INTS13
IRX6	INTS14
ISCA1	INTS15
ISCA2	INTS2
ISCU	INTS3
ISG15	INTS4
ISG20	INTS4P1
ISG20L2	INTS4P2
ISL1	INTS5
ISL2	INTS6
ISLR	INTS6-AS1
ISLR2	INTS6L
ISM1	INTS7
ISM2	INTS8
ISOC1	INTS9
ISOC2	INTU
ISOC2B	INVS
IST1	IP6K1
ISW-1	IP6K2

ISY1	IP6K3
ISY1-RAB43	IPCEF1
ISYNA1	IPF1
ITCH	IPMK
ITFG1	IPO11
ITFG2	IPO13
ITGA1	IPO4
ITGA11	IPO5
ITGA2	IPO5P1
ITGA2B	IPO7
ITGA3	IPO8
ITGA4	IPO9
ITGA5	IPP
ITGA6	IPPK
ITGA7	IPW
ITGA8	IQANK1
ITGA9	IQCA
ITGA9-AS1	IQCA1
ITGAD	IQCB1
ITGAE	IQCC
ITGAL	IQCD
ITGAM	IQCE
ITGAV	IQCF1
ITGAX	IQCF3
ITGB1	IQCF5
ITGB1BP1	IQCF6
ITGB1BP2	IQCG
ITGB1BP3	IQCH
ITGB2	IQCH-AS1
ITGB2-AS1	IQCJ-SCHIP1
ITGB3	IQCK
ITGB3BP	IQCN
ITGB4	IQGAP1
ITGB5	IQGAP2
ITGB6	IQGAP3
ITGB7	IQSEC1

ITGB8
ITGBL1
ITIH1
ITIH2
ITIH3
ITIH4
ITIH5
ITIH6
ITK
ITLN1
ITLN2
ITLN3
ITM2A
ITM2B
ITM2C
ITM2CB
ITPA
ITPK1
ITPK1-AS1
ITPKA
ITPKB
ITPKC
ITPR1
ITPR1A
ITPR2
ITPR3
ITPRID2
ITPRIP
ITPRIPL1
ITPRIPL2
ITSN1
ITSN2
IVD
IVL
IVNS1ABP
IWS1
IQSEC2
IQSEC3
IQUB
IRAG1
IRAG2
IRAIN
IRAK1
IRAK1BP1
IRAK2
IRAK3
IRAK4
IRBP
IREB2
IRF1
IRF2
IRF2BP1
IRF2BP2
IRF2BP2B
IRF2BPL
IRF3
IRF4
IRF5
IRF6
IRF7
IRF8
IRF9
IRG-1
IRG-5
IRG-6
IRGC
IRGC1
IRGM
IRGM1
IRGM2
IRGQ
IRIS

IYD	IRS1
IZUMO1R	IRS2
IZUMO2	IRS3
IZUMO4	IRS4
JADE1	IRX1
JADE2	IRX2
JADE3	IRX2-DT
JAFRAC1	IRX3
JAG1	IRX4
JAG1B	IRX5
JAG2	IRX6
JAGN1	ISCA1
JAK1	ISCA2
JAK2	ISCU
JAK3	ISG15
JAKMIP1	ISG20
JAKMIP2	ISG20L2
JAKMIP2-AS1	ISL1
JAKMIP3	ISL2
JAM2	ISLR
JAM3	ISLR2
JAML	ISM1
JARID2	ISM2
JARID2-AS1	ISOC1
JAZF1	ISOC2
JCAD	ISOC2B
JCHAIN	IST1
JDP2	ISW-1
JHEH1	ISY1
JHEH2	ISY1-RAB43
JHY	ISYNA1
JKAMP	ITCH
JMJD1C	ITFG1
JMJD1C-AS1	ITFG2
JMJD2C	ITGA1
JMJD4	ITGA11

JMJD6	ITGA2
JMJD7	ITGA2B
JMJD7-PLA2G4B	ITGA3
JMJD8	ITGA4
JMY	ITGA5
JOSD1	ITGA6
JOSD2	ITGA7
JPH1	ITGA8
JPH2	ITGA9
JPH3	ITGA9-AS1
JPH4	ITGAD
JPT1	ITGAE
JPT2	ITGAL
JPX	ITGAM
JRK	ITGAV
JRKL	ITGAX
JSRP1	ITGB1
JTB	ITGB1BP1
JUB	ITGB1BP2
JUN	ITGB1BP3
JUNB	ITGB2
JUNBB	ITGB2-AS1
JUND	ITGB3
JUP	ITGB3BP
JUPB	ITGB4
K07C5.10	ITGB5
KAAG1	ITGB6
KAL1	ITGB7
KALRN	ITGB8
KANK1	ITGBL1
KANK2	ITIH1
KANK3	ITIH2
KANK4	ITIH3
KANSL1	ITIH4
KANSL1-AS1	ITIH5
KANSL1L	ITIH6

KANSL2	ITK
KANSL3	ITLN1
KARS1	ITLN2
KASH5	ITLN3
KAT14	ITM2A
KAT2A	ITM2B
KAT2B	ITM2C
KAT5	ITM2CB
KAT6A	ITPA
KAT6B	ITPK1
KAT7	ITPK1-AS1
KAT8	ITPKA
KATNA1	ITPKB
KATNAL1	ITPKC
KATNAL2	ITPR1
KATNB1	ITPR1A
KATNBL1	ITPR2
KATNBL1P6	ITPR3
KATNIP	ITPRID2
KAZALD1	ITPRIP
KAZN	ITPRIPL1
KBTBD11	ITPRIPL2
KBTBD12	ITSN1
KBTBD2	ITSN2
KBTBD3	IVD
KBTBD4	IVL
KBTBD6	IVNS1ABP
KBTBD7	IWS1
KBTBD8	IYD
KC6	IZUMO1R
KCMF1	IZUMO2
KCNA1	IZUMO4
KCNA10	JADE1
KCNA2	JADE2
KCNA3	JADE3
KCNA4	JAFRAC1

KCNA5	JAG1
KCNA6	JAG1B
KCNA7	JAG2
KCNAB1	JAGN1
KCNAB2	JAK1
KCNAB3	JAK2
KCNB1	JAK3
KCNC1	JAKMIP1
KCNC3	JAKMIP2
KCNC4	JAKMIP2-AS1
KCND1	JAKMIP3
KCND2	JAM2
KCND3	JAM3
KCNE1	JAML
KCNE1L	JARID2
KCNE2	JARID2-AS1
KCNE3	JAZF1
KCNE4	JCAD
KCNE5	JCHAIN
KCNE5.L	JDP2
KCNF1	JHEH1
KCNG1	JHEH2
KCNG2	JHY
KCNG3	JKAMP
KCNG4	JMJD1C
KCNH1	JMJD1C-AS1
KCNH2	JMJD2C
KCNH3	JMJD4
KCNH4	JMJD6
KCNH5	JMJD7
KCNH7	JMJD7-PLA2G4B
KCNH8	JMJD8
KCNIP1	JMY
KCNIP2	JOSD1
KCNIP3	JOSD2
KCNIP4	JPH1

KCNJ1	JPH2
KCNJ10	JPH3
KCNJ11	JPH4
KCNJ12	JPT1
KCNJ13	JPT2
KCNJ14	JPX
KCNJ15	JRK
KCNJ16	JRKL
KCNJ2	JSRP1
KCNJ2-AS1	JTB
KCNJ3	JUB
KCNJ4	JUN
KCNJ5	JUNB
KCNJ5-AS1	JUNBB
KCNJ6	JUND
KCNJ8	JUP
KCNJ9	JUPB
KCNK1	K07C5.10
KCNK10	KAAG1
KCNK12	KAL1
KCNK13	KALRN
KCNK15	KANK1
KCNK16	KANK2
KCNK18	KANK3
KCNK2	KANK4
KCNK3	KANSL1
KCNK4	KANSL1-AS1
KCNK5	KANSL1L
KCNK6	KANSL2
KCNK7	KANSL3
KCNK9	KARS1
KCNMA1	KASH5
KCNMB1	KAT14
KCNMB2	KAT2A
KCNMB3	KAT2B
KCNMB4	KAT5

KCNN1	KAT6A
KCNN2	KAT6B
KCNN3	KAT7
KCNN4	KAT8
KCNQ1	KATNA1
KCNQ1DN	KATNAL1
KCNQ1OT1	KATNAL2
KCNQ2	KATNB1
KCNQ3	KATNBL1
KCNQ4	KATNBL1P6
KCNQ5	KATNIP
KCNRG	KAZALD1
KCNS1	KAZN
KCNS2	KBTBD11
KCNS3	KBTBD12
KCNT1	KBTBD2
KCNT2	KBTBD3
KCNU1	KBTBD4
KCNV1	KBTBD6
KCNV2	KBTBD7
KCP	KBTBD8
KCTD1	KC6
KCTD10	KCMF1
KCTD11	KCNA1
KCTD12	KCNA10
KCTD12B	KCNA2
KCTD13	KCNA3
KCTD14	KCNA4
KCTD15	KCNA5
KCTD15B	KCNA6
KCTD16	KCNA7
KCTD17	KCNAB1
KCTD18	KCNAB2
KCTD2	KCNAB3
KCTD20	KCNB1
KCTD21	KCNC1

KCTD3	KCNC3
KCTD4	KCNC4
KCTD5	KCND1
KCTD6	KCND2
KCTD7	KCND3
KCTD8	KCNE1
KCTD9	KCNE1L
KCTD9A	KCNE2
KCTD9P2	KCNE3
KDELR1	KCNE4
KDELR2	KCNE5
KDELR3	KCNE5.L
KDF1	KCNF1
KDM1A	KCNG1
KDM1B	KCNG2
KDM2A	KCNG3
KDM2B	KCNG4
KDM3A	KCNH1
KDM3B	KCNH2
KDM4A	KCNH3
KDM4B	KCNH4
KDM4C	KCNH5
KDM4D	KCNH7
KDM5A	KCNH8
KDM5B	KCNIP1
KDM5C	KCNIP2
KDM5D	KCNIP3
KDM6A	KCNIP4
KDM6B	KCNJ1
KDM6BB	KCNJ10
KDM7A	KCNJ11
KDM7AA	KCNJ12
KDM7A-DT	KCNJ13
KDM8	KCNJ14
KDR	KCNJ15
KDRL	KCNJ16

KDSR	KCNJ2
KEAP1	KCNJ2-AS1
KEAP1A	KCNJ3
KEAP1B	KCNJ4
KEG1	KCNJ5
KEL	KCNJ5-AS1
KERA	KCNJ6
KHDC1	KCNJ8
KHDC1L	KCNJ9
KHDC3	KCNK1
KHDC3L	KCNK10
KHDC4	KCNK12
KHDRBS1	KCNK13
KHDRBS1B	KCNK15
KHDRBS2	KCNK16
KHDRBS3	KCNK18
KHK	KCNK2
KHNYN	KCNK3
KHSRP	KCNK4
KIAA0040	KCNK5
KIAA0087	KCNK6
KIAA0232	KCNK7
KIAA0319	KCNK9
KIAA0319L	KCNMA1
KIAA0408	KCNMB1
KIAA0513	KCNMB2
KIAA0586	KCNMB2-AS1
KIAA0753	KCNMB3
KIAA0825	KCNMB4
KIAA0930	KCNN1
KIAA1143	KCNN2
KIAA1191	KCNN3
KIAA1210	KCNN4
KIAA1217	KCNQ1
KIAA1328	KCNQ1DN
KIAA1522	KCNQ1OT1

KIAA1549	KCNQ2
KIAA1549L	KCNQ3
KIAA1586	KCNQ4
KIAA1614	KCNQ5
KIAA1644	KCNRG
KIAA1671	KCNS1
KIAA1671-AS1	KCNS2
KIAA1755	KCNS3
KIAA1919	KCNT1
KIAA1958	KCNT2
KIAA2012	KCNU1
KIAA2026	KCNV1
KICS2	KCNV2
KIDINS220	KCP
KIF11	KCTD1
KIF12	KCTD10
KIF13A	KCTD11
KIF13B	KCTD12
KIF14	KCTD12B
KIF15	KCTD13
KIF16B	KCTD14
KIF17	KCTD15
KIF18A	KCTD15B
KIF18B	KCTD16
KIF19	KCTD17
KIF19A	KCTD18
KIF1A	KCTD2
KIF1B	KCTD20
KIF1C	KCTD21
KIF20A	KCTD3
KIF20B	KCTD4
KIF21A	KCTD5
KIF21B	KCTD6
KIF22	KCTD7
KIF23	KCTD8
KIF23-AS1	KCTD9

KIF24	KCTD9A
KIF25	KCTD9P2
KIF25-AS1	KDELR1
KIF26A	KDELR2
KIF26B	KDELR3
KIF26B-AS1	KDF1
KIF27	KDM1A
KIF28P	KDM1B
KIF2A	KDM2A
KIF2B	KDM2B
KIF2C	KDM3A
KIF3A	KDM3B
KIF3B	KDM4A
KIF3C	KDM4B
KIF4	KDM4C
KIF4A	KDM4D
KIF5A	KDM5A
KIF5B	KDM5B
KIF5C	KDM5C
KIF6	KDM5D
KIF7	KDM6A
KIF9	KDM6B
KIFAP3	KDM6BB
KIFBP	KDM7A
KIFC1	KDM7AA
KIFC2	KDM7A-DT
KIFC3	KDM8
KIN	KDR
KIR2DL1	KDRL
KIR2DL3	KDSR
KIR2DS2	KEAP1
KIR2DS3	KEAP1A
KIR2DS4	KEAP1B
KIR3DL1	KEG1
KIR3DL2	KEL
KIR3DL3	KERA

KIR3DS1	KHDC1
KIR3DX1	KHDC1L
KIRREL1	KHDC3
KIRREL2	KHDC3L
KIRREL3	KHDC4
KIRREL3-AS3	KHDRBS1
KIS	KHDRBS1B
KISS1	KHDRBS2
KISS1R	KHDRBS3
KIT	KHK
KITL	KHNYN
KITLG	KHSRP
KIZ	KIAA0040
KL	KIAA0087
KLB	KIAA0232
KLC1	KIAA0319
KLC2	KIAA0319L
KLC3	KIAA0408
KLF1	KIAA0513
KLF10	KIAA0586
KLF11	KIAA0753
KLF11A	KIAA0825
KLF12	KIAA0930
KLF13	KIAA1143
KLF14	KIAA1191
KLF15	KIAA1210
KLF16	KIAA1217
KLF17	KIAA1328
KLF2	KIAA1522
KLF3	KIAA1549
KLF3-AS1	KIAA1549L
KLF4	KIAA1586
KLF5	KIAA1614
KLF6	KIAA1644
KLF7	KIAA1671
KLF8	KIAA1671-AS1

KLF9	KIAA1755
KLHDC1	KIAA1919
KLHDC10	KIAA1958
KLHDC2	KIAA2012
KLHDC3	KIAA2026
KLHDC4	KICS2
KLHDC7A	KIDINS220
KLHDC7B	KIF11
KLHDC8A	KIF12
KLHDC8B	KIF13A
KLHDC9	KIF13B
KLHL1	KIF14
KLHL10	KIF15
KLHL11	KIF16B
KLHL12	KIF17
KLHL13	KIF18A
KLHL14	KIF18B
KLHL15	KIF19
KLHL17	KIF19A
KLHL18	KIF1A
KLHL2	KIF1B
KLHL20	KIF1C
KLHL21	KIF20A
KLHL22	KIF20B
KLHL23	KIF21A
KLHL24	KIF21B
KLHL24B	KIF22
KLHL25	KIF23
KLHL26	KIF23-AS1
KLHL28	KIF24
KLHL29	KIF25
KLHL3	KIF25-AS1
KLHL30	KIF26A
KLHL31	KIF26B
KLHL32	KIF26B-AS1
KLHL33	KIF27

KLHL34	KIF28P
KLHL35	KIF2A
KLHL36	KIF2B
KLHL38	KIF2C
KLHL38B	KIF3A
KLHL4	KIF3B
KLHL40	KIF3C
KLHL41	KIF4
KLHL41B	KIF4A
KLHL42	KIF5A
KLHL5	KIF5B
KLHL6	KIF5C
KLHL7	KIF6
KLHL7-DT	KIF7
KLHL8	KIF9
KLHL9	KIFAP3
KLK1	KIFBP
KLK10	KIFC1
KLK11	KIFC2
KLK12	KIFC3
KLK13	KIN
KLK15	KIR2DL1
KLK1B1	KIR2DL2
KLK1B21	KIR2DL3
KLK1B27	KIR2DL4
KLK1B4	KIR2DS1
KLK1B5	KIR2DS2
KLK1C6	KIR2DS3
KLK1C9	KIR2DS4
KLK2	KIR3DL1
KLK3	KIR3DL2
KLK4	KIR3DL3
KLK5	KIR3DS1
KLK6	KIR3DX1
KLK7	KIRREL1
KLK8	KIRREL2

KLK9	KIRREL3
KLKB1	KIRREL3-AS3
KLKP1	KIS
KLLN	KISS1
KLRA1	KISS1R
KLRA14-PS	KIT
KLRA16	KITL
KLRA18	KITLG
KLRA2	KIZ
KLRA22	KL
KLRA3	KLB
KLRA7	KLC1
KLRB1	KLC2
KLRB1A	KLC3
KLRB1B	KLF1
KLRB1C	KLF10
KLRB1F	KLF11
KLRC1	KLF11A
KLRC2	KLF12
KLRC3	KLF13
KLRC4	KLF14
KLRD1	KLF15
KLRE1	KLF16
KLRF1	KLF17
KLRG1	KLF2
KLRG2	KLF3
KLRK1	KLF3-AS1
KLRK1-AS1	KLF4
KMO	KLF5
KMT2A	KLF6
KMT2B	KLF7
KMT2C	KLF8
KMT2D	KLF9
KMT2E	KLHDC1
KMT5A	KLHDC10
KMT5B	KLHDC2

KMT5B.L	KLHDC3
KMT5C	KLHDC4
KNCN	KLHDC7A
KNDC1	KLHDC7B
KNG1	KLHDC8A
KNG2	KLHDC8B
KNL1	KLHDC9
KNOP1	KLHL1
KNSTRN	KLHL10
KNTC1	KLHL11
KPNA1	KLHL12
KPNA2	KLHL13
KPNA3	KLHL14
KPNA4	KLHL15
KPNA5	KLHL17
KPNA6	KLHL18
KPNA7	KLHL2
KPNB1	KLHL20
KPNB3	KLHL21
KPRP	KLHL22
KPTN	KLHL23
KRAS	KLHL24
KRBA1	KLHL24B
KRBA2	KLHL25
KRBOX1	KLHL26
KRBOX4	KLHL28
KRBOX5	KLHL29
KRCC1	KLHL3
KREMEN1	KLHL30
KREMEN2	KLHL31
KRI1	KLHL32
KRIT1	KLHL33
KRR1	KLHL34
KRT1	KLHL35
KRT10	KLHL36
KRT10-AS1	KLHL38

KRT1-19D	KLHL38B
KRT12	KLHL4
KRT13	KLHL40
KRT14	KLHL41
KRT15	KLHL41B
KRT16	KLHL42
KRT16P2	KLHL5
KRT16P3	KLHL6
KRT16P6	KLHL7
KRT17	KLHL7-DT
KRT17P1	KLHL8
KRT17P2	KLHL9
KRT17P3	KLK1
KRT17P5	KLK10
KRT18	KLK11
KRT18P19	KLK12
KRT18P44	KLK13
KRT18P50	KLK14
KRT19	KLK15
KRT2	KLK1B1
KRT20	KLK1B21
KRT222	KLK1B27
KRT23	KLK1B4
KRT24	KLK1B5
KRT25	KLK1C6
KRT27	KLK1C9
KRT28	KLK2
KRT3	KLK3
KRT32	KLK4
KRT33A	KLK5
KRT34	KLK6
KRT35	KLK7
KRT36	KLK8
KRT38	KLK9
KRT39	KLKB1
KRT4	KLKP1

KRT40	KLLN
KRT42	KLRA1
KRT42P	KLRA14-PS
KRT5	KLRA16
KRT62	KLRA18
KRT6A	KLRA2
KRT6B	KLRA22
KRT6C	KLRA3
KRT7	KLRA7
KRT71	KLRB1
KRT73	KLRB1A
KRT74	KLRB1B
KRT75	KLRB1C
KRT76	KLRB1F
KRT77	KLRC1
KRT78	KLRC2
KRT79	KLRC3
KRT7-AS	KLRC4
KRT8	KLRC4-KLRK1
KRT80	KLRD1
KRT81	KLRE1
KRT82	KLRF1
KRT83	KLRG1
KRT84	KLRG2
KRT85	KLRK1
KRT86	KLRK1-AS1
KRT87P	KMO
KRT8P12	KMT2A
KRT8P41	KMT2B
KRT8P47	KMT2C
KRT9	KMT2D
KRT90	KMT2E
KRTAP10-1	KMT5A
KRTAP10-10	KMT5B
KRTAP10-11	KMT5B.L
KRTAP10-12	KMT5C

KRTAP10-2	KNCN
KRTAP10-3	KNDC1
KRTAP10-4	KNG1
KRTAP10-5	KNG2
KRTAP10-6	KNL1
KRTAP10-7	KNOP1
KRTAP10-8	KNSTRN
KRTAP1-1	KNTC1
KRTAP12-2	KPNA1
KRTAP12-3	KPNA2
KRTAP12-4	KPNA3
KRTAP1-3	KPNA4
KRTAP13-3	KPNA5
KRTAP13-4	KPNA6
KRTAP14	KPNA7
KRTAP1-4	KPNB1
KRTAP17-1	KPNB3
KRTAP19-1	KPRP
KRTAP19-3	KPTN
KRTAP19-4	KRAS
KRTAP19-5	KRBA1
KRTAP20-4	KRBA2
KRTAP2-1	KRBOX1
KRTAP21-1	KRBOX4
KRTAP22-1	KRBOX5
KRTAP2-3	KRCC1
KRTAP2-4	KREMEN1
KRTAP26-1	KREMEN2
KRTAP27-1	KRI1
KRTAP3-1	KRIT1
KRTAP4-1	KRR1
KRTAP4-11	KRT1
KRTAP4-12	KRT10
KRTAP4-2	KRT10-AS1
KRTAP4-3	KRT1-19D
KRTAP4-4	KRT12

KRTAP4-5	KRT13
KRTAP4-7	KRT14
KRTAP4-8	KRT15
KRTAP4-9	KRT16
KRTAP5-1	KRT16P2
KRTAP5-10	KRT16P3
KRTAP5-11	KRT16P6
KRTAP5-4	KRT17
KRTAP5-5	KRT17P1
KRTAP5-6	KRT17P2
KRTAP5-7	KRT17P3
KRTAP5-9	KRT17P5
KRTAP5-AS1	KRT18
KRTAP6-1	KRT18P19
KRTAP6-2	KRT18P44
KRTAP6-3	KRT18P50
KRTAP7-1	KRT19
KRTAP9-3	KRT2
KRTAP9-8	KRT20
KRTCAP2	KRT222
KRTCAP3	KRT23
KRTDAP	KRT24
KSR1	KRT25
KSR2	KRT27
KTI12	KRT28
KTN1	KRT3
KTN1-AS1	KRT32
KU80	KRT33A
KXD1	KRT34
KY	KRT35
KYAT1	KRT36
KYAT3	KRT38
KYNU	KRT39
L1CAM	KRT4
L1TD1	KRT40
L2HGDH	KRT42

L3HYPDH	KRT42P
L3MBTL1	KRT5
L3MBTL2	KRT62
L3MBTL3	KRT6A
L3MBTL4	KRT6B
LACC1	KRT6C
LACTB	KRT7
LACTB2	KRT71
LAD1	KRT73
LAG3	KRT74
LAGE3	KRT75
LAIR1	KRT76
LAMA1	KRT77
LAMA2	KRT78
LAMA3	KRT79
LAMA4	KRT7-AS
LAMA5	KRT8
LAMA5-AS1	KRT80
LAMB1	KRT81
LAMB2	KRT82
LAMB2P1	KRT83
LAMB3	KRT84
LAMB4	KRT85
LAMC1	KRT86
LAMC2	KRT87P
LAMC3	KRT8P12
LAMP1	KRT8P3
LAMP2	KRT8P41
LAMP3	KRT8P47
LAMP5	KRT9
LAMTOR1	KRT90
LAMTOR2	KRTAP10-1
LAMTOR3	KRTAP10-10
LAMTOR4	KRTAP10-11
LAMTOR5	KRTAP10-12
LANCL1	KRTAP10-2

LANCL2	KRTAP10-3
LANCL3	KRTAP10-4
LAP3	KRTAP10-5
LAPTM4A	KRTAP10-6
LAPTM4A.S	KRTAP10-7
LAPTM4B	KRTAP10-8
LAPTM5	KRTAP1-1
LARGE	KRTAP12-2
LARGE1	KRTAP12-3
LARGE2	KRTAP12-4
LARP1	KRTAP1-3
LARP1B	KRTAP13-3
LARP4	KRTAP13-4
LARP4B	KRTAP14
LARP6	KRTAP1-4
LARP7	KRTAP17-1
LARS1	KRTAP19-1
LAS1L	KRTAP19-3
LASP1	KRTAP19-4
LASS6	KRTAP19-5
LAT	KRTAP20-4
LAT2	KRTAP2-1
LATS1	KRTAP21-1
LATS2	KRTAP22-1
LAX1	KRTAP2-3
LAYN	KRTAP2-4
LBH	KRTAP26-1
LBHD1	KRTAP27-1
LBP	KRTAP3-1
LBR	KRTAP4-1
LBX1	KRTAP4-11
LBX2	KRTAP4-12
LCA5	KRTAP4-2
LCA5L	KRTAP4-3
LCAL1	KRTAP4-4
LCAT	KRTAP4-5

LCE1A	KRTAP4-7
LCE1B	KRTAP4-8
LCE1C	KRTAP4-9
LCE1D	KRTAP5-1
LCE1E	KRTAP5-10
LCE1F	KRTAP5-11
LCE1I	KRTAP5-4
LCE1M	KRTAP5-5
LCE2A	KRTAP5-6
LCE2B	KRTAP5-7
LCE2C	KRTAP5-9
LCE2D	KRTAP5-AS1
LCE3A	KRTAP6-1
LCE3D	KRTAP6-2
LCE3E	KRTAP6-3
LCE4A	KRTAP7-1
LCE5A	KRTAP9-3
LCE6A	KRTAP9-8
LCK	KRTCAP2
LCLAT1	KRTCAP3
LCMT1	KRTDAP
LCMT2	KSR1
LCN1	KSR2
LCN10	KTI12
LCN12	KTN1
LCN15	KTN1-AS1
LCN2	KU80
LCN6	KXD1
LCN8	KY
LCN9	KYAT1
LCNL1	KYAT3
LCOR	KYNU
LCORL	L1CAM
LCP1	L1TD1
LCP2	L2HGDH
LCT	L3HYPDH

LCTL	L3MBTL1
LDAF1	L3MBTL2
LDAH	L3MBTL3
LDB1	L3MBTL4
LDB2	LACC1
LDB3	LACTB
LDB3A	LACTB2
LDB3B	LAD1
LDHA	LAG3
LDHAL6A	LAGE3
LDHB	LAIR1
LDHC	LAMA1
LDHD	LAMA2
LDLR	LAMA3
LDLRAD1	LAMA4
LDLRAD2	LAMA5
LDLRAD3	LAMA5-AS1
LDLRAD4	LAMB1
LDLRAD4-AS1	LAMB2
LDLRAP1	LAMB2P1
LDLRAP1.S	LAMB3
LDLRAP1A	LAMB4
LDOC1	LAMC1
LEAP2	LAMC2
LECT2	LAMC3
LEF1	LAMP1
LEF1-AS1	LAMP2
LEFTY1	LAMP3
LEFTY2	LAMP5
LEKR1	LAMTOR1
LELP1	LAMTOR2
LEMD1	LAMTOR3
LEMD2	LAMTOR4
LEMD3	LAMTOR5
LENG1	LANCL1
LENG8	LANCL2

LENG8-AS1	LANCL3
LENG9	LAP3
LEO1	LAPTM4A
LEP	LAPTM4A.S
LEPA	LAPTM4B
LEPR	LAPTM5
LEPROT	LARGE
LEPROTL1	LARGE1
LETM1	LARGE2
LETM2	LARP1
LETMD1	LARP1B
LEV-1	LARP4
LEV-8	LARP4B
LEXM	LARP6
LFNG	LARP7
LGALS1	LARS1
LGALS12	LARS2
LGALS2	LAS1L
LGALS2B	LASP1
LGALS3	LASS6
LGALS3BP	LAT
LGALS4	LAT2
LGALS5	LATS1
LGALS7	LATS2
LGALS7B	LAX1
LGALS8	LAYN
LGALS9	LBH
LGALS9B	LBHD1
LGALS9C	LBP
LGALS9L1	LBR
LGALSL	LBX1
LGI1	LBX2
LGI1A	LBX2-AS1
LGI2	LCA5
LGI3	LCA5L
LGI4	LCAL1

LGMN	LCAT
LGR4	LCE1A
LGR5	LCE1B
LGR6	LCE1C
LGSN	LCE1D
LHB	LCE1E
LHCGR	LCE1F
LHFPL1	LCE1I
LHFPL2	LCE1M
LHFPL3	LCE2A
LHFPL4	LCE2B
LHFPL5	LCE2C
LHFPL6	LCE2D
LHFPL7	LCE3A
LHPP	LCE3D
LHX1	LCE3E
LHX2	LCE4A
LHX3	LCE5A
LHX4	LCE6A
LHX4-AS1	LCK
LHX5	LCLAT1
LHX6	LCMT1
LHX8	LCMT2
LHX8A	LCN1
LHX9	LCN10
LIAS	LCN12
LIF	LCN15
LIF-AS2	LCN2
LIFR	LCN6
LIG1	LCN8
LIG3	LCN9
LIG4	LCNL1
LILRA2	LCOR
LILRA3	LCORL
LILRA4	LCP1
LILRA5	LCP2

LILRA6	LCT
LILRB1	LCTL
LILRB2	LDAF1
LILRB3	LDAH
LILRB3A	LDB1
LILRB3L	LDB2
LILRB4	LDB3
LILRB4A	LDB3A
LILRC2	LDB3B
LIM2	LDHA
LIM2.3	LDHAL6A
LIM2.4	LDHB
LIMA1	LDHC
LIMCH1	LDHD
LIMD1	LDLR
LIMD2	LDLRAD1
LIME1	LDLRAD2
LIMK1	LDLRAD3
LIMK2	LDLRAD4
LIMS1	LDLRAD4-AS1
LIMS2	LDLRAP1
LIMS3	LDLRAP1.S
LIMS3-LOC440895	LDLRAP1A
LIN28A	LDOC1
LIN28B	LEAP2
LIN-29	LECT2
LIN37	LEF1
LIN52	LEF1-AS1
LIN54	LEFTY1
LIN7A	LEFTY2
LIN7B	LEKR1
LIN7C	LELP1
LIN9	LEMD1
LINC00029	LEMD2
LINC00032	LEMD3
LINC00051	LENG1

LINC00052	LENG8
LINC00092	LENG8-AS1
LINC00102	LENG9
LINC00106	LEO1
LINC00111	LEP
LINC00115	LEPA
LINC00158	LEPQTL1
LINC00173	LEPR
LINC00174	LEPROT
LINC00176	LEPROTL1
LINC00189	LETM1
LINC00200	LETM2
LINC00205	LETMD1
LINC00207	LEV-1
LINC00235	LEV-8
LINC00240	LEXM
LINC00242	LFNG
LINC00260	LGALS1
LINC00261	LGALS12
LINC00265	LGALS2
LINC00268	LGALS2B
LINC00294	LGALS3
LINC00299	LGALS3BP
LINC00301	LGALS4
LINC00304	LGALS5
LINC00305	LGALS7
LINC00309	LGALS7B
LINC00310	LGALS8
LINC00311	LGALS9
LINC00312	LGALS9B
LINC00313	LGALS9C
LINC00315	LGALS9L1
LINC00319	LGALSL
LINC00323	LGI1
LINC00324	LGI1A
LINC00326	LGI2

LINC00327	LGI3
LINC00334	LGI4
LINC00336	LGMN
LINC00339	LGR4
LINC00342	LGR5
LINC00365	LGR6
LINC00452	LGSN
LINC00458	LHB
LINC00460	LHCGR
LINC00461	LHFPL1
LINC00467	LHFPL2
LINC00472	LHFPL3
LINC00473	LHFPL4
LINC00474	LHFPL5
LINC00477	LHFPL6
LINC00479	LHFPL7
LINC00482	LHPP
LINC00486	LHX1
LINC00491	LHX2
LINC00511	LHX3
LINC00515	LHX4
LINC00520	LHX4-AS1
LINC00523	LHX5
LINC00525	LHX6
LINC00526	LHX8
LINC00528	LHX8A
LINC00537	LHX9
LINC00540	LIAS
LINC00545	LIF
LINC00551	LIF-AS2
LINC00552	LIFR
LINC00570	LIG1
LINC00574	LIG3
LINC00588	LIG4
LINC00589	LILRA2
LINC00597	LILRA3

LINC00602	LILRA4
LINC00606	LILRA5
LINC00607	LILRA6
LINC00612	LILRB1
LINC00616	LILRB2
LINC00622	LILRB3
LINC00623	LILRB3A
LINC00624	LILRB3L
LINC00630	LILRB4
LINC00632	LILRB4A
LINC00638	LILRC2
LINC00641	LIM2
LINC00645	LIM2.3
LINC00648	LIM2.4
LINC00652	LIMA1
LINC00654	LIMCH1
LINC00656	LIMD1
LINC00661	LIMD2
LINC00662	LIME1
LINC00663	LIMK1
LINC00664	LIMK2
LINC00665	LIMS1
LINC00667	LIMS2
LINC00671	LIMS3
LINC00672	LIMS3-LOC440895
LINC00673	LIN28A
LINC00689	LIN28B
LINC00691	LIN-29
LINC00696	LIN37
LINC00698	LIN52
LINC00703	LIN54
LINC00705	LIN7A
LINC00707	LIN7B
LINC00839	LIN7C
LINC00840	LIN9
LINC00842	LINC00029

LINC00844	LINC00032
LINC00847	LINC00051
LINC00857	LINC00052
LINC00858	LINC00092
LINC00862	LINC00102
LINC00863	LINC00106
LINC00865	LINC00111
LINC00869	LINC00115
LINC00880	LINC00158
LINC00885	LINC00173
LINC00886	LINC00174
LINC00887	LINC00176
LINC00888	LINC00189
LINC00896	LINC00200
LINC00900	LINC00205
LINC00908	LINC00207
LINC00917	LINC00210
LINC00923	LINC00221
LINC00926	LINC00235
LINC00937	LINC00240
LINC00938	LINC00242
LINC00939	LINC00243
LINC00941	LINC00260
LINC00942	LINC00261
LINC00944	LINC00265
LINC00954	LINC00268
LINC00957	LINC00294
LINC00958	LINC00299
LINC00960	LINC00301
LINC00963	LINC00304
LINC00964	LINC00305
LINC00968	LINC00309
LINC00987	LINC00310
LINC00992	LINC00311
LINC00994	LINC00312
LINC00997	LINC00313

LINC01000	LINC00315
LINC01002	LINC00319
LINC01003	LINC00323
LINC01004	LINC00324
LINC01011	LINC00326
LINC01014	LINC00327
LINC01018	LINC00328
LINC01060	LINC00334
LINC01088	LINC00336
LINC01089	LINC00337
LINC01091	LINC00339
LINC01093	LINC00342
LINC01094	LINC00346
LINC01101	LINC00365
LINC01102	LINC00426
LINC01107	LINC00452
LINC01119	LINC00458
LINC01121	LINC00460
LINC01128	LINC00461
LINC01132	LINC00467
LINC01133	LINC00472
LINC01134	LINC00473
LINC01138	LINC00474
LINC01139	LINC00477
LINC01140	LINC00479
LINC01142	LINC00482
LINC01164	LINC00486
LINC01191	LINC00491
LINC01193	LINC00511
LINC01204	LINC00515
LINC01208	LINC00520
LINC01210	LINC00523
LINC01234	LINC00525
LINC01238	LINC00526
LINC01239	LINC00528
LINC01250	LINC00537

LINC01252	LINC00540
LINC01257	LINC00545
LINC01267	LINC00551
LINC01269	LINC00552
LINC01270	LINC00570
LINC01271	LINC00574
LINC01273	LINC00588
LINC01275	LINC00589
LINC01278	LINC00597
LINC01303	LINC00602
LINC01312	LINC00606
LINC01315	LINC00607
LINC01341	LINC00612
LINC01342	LINC00616
LINC01392	LINC00622
LINC01393	LINC00623
LINC01402	LINC00624
LINC01405	LINC00630
LINC01409	LINC00632
LINC01410	LINC00635
LINC01436	LINC00638
LINC01448	LINC00641
LINC01460	LINC00645
LINC01465	LINC00648
LINC01474	LINC00652
LINC01484	LINC00654
LINC01503	LINC00656
LINC01504	LINC00661
LINC01515	LINC00662
LINC01523	LINC00663
LINC01547	LINC00664
LINC01549	LINC00665
LINC01550	LINC00667
LINC01551	LINC00668
LINC01554	LINC00671
LINC01555	LINC00672

LINC01558	LINC00673
LINC01559	LINC00689
LINC01560	LINC00691
LINC01564	LINC00696
LINC01565	LINC00698
LINC01569	LINC00702
LINC01587	LINC00703
LINC01588	LINC00705
LINC01589	LINC00707
LINC01600	LINC00839
LINC01605	LINC00840
LINC01619	LINC00842
LINC01620	LINC00844
LINC01629	LINC00847
LINC01634	LINC00857
LINC01637	LINC00858
LINC01671	LINC00862
LINC01679	LINC00863
LINC01711	LINC00865
LINC01722	LINC00869
LINC01732	LINC00880
LINC01772	LINC00885
LINC01777	LINC00886
LINC01783	LINC00887
LINC01814	LINC00888
LINC01820	LINC00896
LINC01836	LINC00900
LINC01843	LINC00908
LINC01881	LINC00917
LINC01896	LINC00923
LINC01907	LINC00926
LINC01911	LINC00937
LINC01933	LINC00938
LINC01948	LINC00939
LINC01963	LINC00941
LINC01970	LINC00942

LINC01982	LINC00944
LINC01990	LINC00954
LINC01996	LINC00957
LINC02021	LINC00958
LINC02029	LINC00960
LINC02035	LINC00963
LINC02042	LINC00964
LINC02043	LINC00968
LINC02056	LINC00987
LINC02057	LINC00992
LINC02069	LINC00994
LINC02076	LINC00997
LINC02104	LINC01000
LINC02125	LINC01002
LINC02145	LINC01003
LINC02148	LINC01004
LINC02150	LINC01011
LINC02159	LINC01014
LINC02175	LINC01018
LINC02178	LINC01060
LINC02182	LINC01088
LINC02203	LINC01089
LINC02210	LINC01091
LINC02245	LINC01093
LINC02249	LINC01094
LINC02265	LINC01101
LINC02289	LINC01102
LINC02290	LINC01107
LINC02292	LINC01116
LINC02331	LINC01119
LINC02345	LINC01121
LINC02370	LINC01123
LINC02381	LINC01128
LINC02387	LINC01132
LINC02395	LINC01133
LINC02449	LINC01134

LINC02532
LINC02541
LINC02560
LINC02561
LINC02579
LINC02582
LINC02601
LINC02603
LINC02610
LINC02614
LINC02615
LINC02649
LINC02681
LINC02688
LINC02693
LINC02696
LINC02709
LINC02731
LINC02763
LINC02777
LINC02783
LINC02870
LINC02875
LINC02877
LINC02889
LINC02894
LINC02901
LINC02904
LINC02905
LINC02907
LINC02908
LINC02909
LINC02910
LINC02961
LINC03011
LINC03040

LINC01138
LINC01139
LINC01140
LINC01142
LINC01164
LINC01186
LINC01191
LINC01193
LINC01194
LINC01204
LINC01208
LINC01210
LINC01234
LINC01238
LINC01239
LINC01250
LINC01252
LINC01257
LINC01267
LINC01269
LINC01270
LINC01271
LINC01273
LINC01275
LINC01278
LINC01288
LINC01303
LINC01312
LINC01315
LINC01341
LINC01342
LINC01354
LINC01392
LINC01393
LINC01402
LINC01405

LINC03042	LINC01409
LINC-72	LINC01410
LINC-PINT	LINC01433
LINCR	LINC01436
LINGO1	LINC01448
LINGO2	LINC01460
LINGO3	LINC01465
LINGO4	LINC01474
LINS1	LINC01484
LIP1	LINC01502
LIPA	LINC01503
LIPC	LINC01504
LIPE	LINC01511
LIPE-AS1	LINC01512
LIPF	LINC01513
LIPG	LINC01515
LIPH	LINC01523
LIPJ	LINC01547
LIPL-3	LINC01549
LIPN	LINC01550
LIPT1	LINC01551
LIPT2	LINC01554
LITAF	LINC01555
LIX1	LINC01558
LIX1L	LINC01559
LIX1L-AS1	LINC01560
LKAAEAR1	LINC01564
LL22NC03-63E9.3	LINC01565
LLCFC1	LINC01569
LLGL1	LINC01587
LLGL2	LINC01588
LLPH	LINC01589
LMAN1	LINC01600
LMAN1L	LINC01605
LMAN2	LINC01614
LMAN2L	LINC01619

LMAN2LA	LINC01620
LMBR1	LINC01627
LMBR1L	LINC01628
LMBRD1	LINC01629
LMBRD2	LINC01634
LMBRD2B	LINC01637
LMCD1	LINC01638
LMF1	LINC01671
LMF2	LINC01672
LMLN	LINC01679
LMNA	LINC01711
LMNB1	LINC01722
LMNB2	LINC01732
LMNTD1	LINC01772
LMNTD2	LINC01777
LMNTD2-AS1	LINC01783
LMO1	LINC01814
LMO2	LINC01820
LMO3	LINC01836
LMO4	LINC01843
LMO7	LINC01852
LMO7-AS1	LINC01881
LMO7DN	LINC01896
LMO7DN-IT1	LINC01907
LMOD1	LINC01911
LMOD2	LINC01933
LMOD3	LINC01948
LMTK2	LINC01963
LMTK3	LINC01969
LMX1A	LINC01970
LMX1B	LINC01982
LNCRI	LINC01990
LNP1	LINC01996
LNPEP	LINC02021
LNPK	LINC02029
LNPK.L	LINC02035

LNX1	LINC02042
LNX2	LINC02043
LOK	LINC02056
LONP1	LINC02057
LONP2	LINC02069
LONRF1	LINC02076
LONRF2	LINC02104
LONRF3	LINC02125
LORICRIN	LINC02145
LOX	LINC02148
LOXL1	LINC02150
LOXL1-AS1	LINC02159
LOXL2	LINC02175
LOXL3	LINC02178
LOXL3B	LINC02182
LOXL4	LINC02203
LOXL5B	LINC02210
LPA	LINC02245
LPAL2	LINC02249
LPAR1	LINC02265
LPAR2	LINC02289
LPAR2B	LINC02290
LPAR3	LINC02292
LPAR4	LINC02331
LPAR5	LINC02345
LPAR6	LINC02370
LPCAT1	LINC02381
LPCAT2	LINC02387
LPCAT2B	LINC02395
LPCAT3	LINC02412
LPCAT4	LINC02418
LPGAT1	LINC02449
LPIN1	LINC02532
LPIN2	LINC02541
LPIN3	LINC02560
LPL	LINC02561

LPO	LINC02579
LPP	LINC02582
LPP-AS2	LINC02601
LPPR1	LINC02602
LPXN	LINC02603
LRAT	LINC02605
LRATD1	LINC02610
LRATD2	LINC02614
LRBA	LINC02615
LRCH1	LINC02633
LRCH2	LINC02649
LRCH3	LINC02681
LRCH4	LINC02688
LRCOL1	LINC02693
LRE3	LINC02696
LRFN1	LINC02709
LRFN2	LINC02731
LRFN3	LINC02763
LRFN4	LINC02777
LRFN5	LINC02783
LRG1	LINC02870
LRGUK	LINC02875
LRIF1	LINC02877
LRIG1	LINC02889
LRIG2	LINC02894
LRIG3	LINC02901
LRIT1	LINC02904
LRIT2	LINC02905
LRIT3	LINC02907
LRMDA	LINC02908
LRP1	LINC02909
LRP10	LINC02910
LRP11	LINC02961
LRP12	LINC03011
LRP1B	LINC03040
LRP2	LINC03042

LRP2BP	LINC-72
LRP3	LINC-PINT
LRP4	LINCR
LRP5	LINC-ROR
LRP6	LINGO1
LRP8	LINGO2
LRPAP1	LINGO3
LRPPRC	LINGO4
LRR1	LINS1
LRRC1	LIP1
LRRC10	LIPA
LRRC10B	LIPC
LRRC14	LIPE
LRRC14B	LIPE-AS1
LRRC15	LIPF
LRRC17	LIPG
LRRC18	LIPH
LRRC19	LIPJ
LRRC2	LIPL-3
LRRC20	LIPN
LRRC23	LIPT1
LRRC24	LIPT2
LRRC25	LITAF
LRRC26	LIX1
LRRC27	LIX1L
LRRC28	LIX1L-AS1
LRRC29	LKAAEAR1
LRRC3	LL22NC03-63E9.3
LRRC30A	LLCFC1
LRRC31	LLGL1
LRRC32	LLGL2
LRRC34	LLPH
LRRC36	LMAN1
LRRC37A	LMAN1L
LRRC37A11P	LMAN2
LRRC37A16P	LMAN2L

LRRC37A2	LMAN2LA
LRRC37A3	LMBR1
LRRC37A4P	LMBR1L
LRRC37A5P	LMBRD1
LRRC37B	LMBRD2
LRRC37BP1	LMBRD2B
LRRC39	LMCD1
LRRC3B	LMF1
LRRC4	LMF2
LRRC40	LMLN
LRRC41	LMNA
LRRC42	LMNB1
LRRC43	LMNB2
LRRC45	LMNTD1
LRRC46	LMNTD2
LRRC47	LMNTD2-AS1
LRRC49	LMO1
LRRC4B	LMO2
LRRC4C	LMO3
LRRC51	LMO4
LRRC52	LMO7
LRRC53	LMO7-AS1
LRRC55	LMO7DN
LRRC56	LMO7DN-IT1
LRRC57	LMOD1
LRRC58	LMOD2
LRRC59	LMOD3
LRRC61	LMTK2
LRRC63	LMTK3
LRRC66	LMX1A
LRRC69	LMX1B
LRRC7	LNCNEF
LRRC70	LNCR3
LRRC71	LNCRI
LRRC73	LNCRNA-ATB
LRRC74B	LNP1

LRRC75A	LNPEP
LRRC75B	LNPK
LRRC77P	LNPK.L
LRRC8A	LNX1
LRRC8B	LNX2
LRRC8C	LOC105371114
LRRC8D	LOC110806263
LRRC8DB	LOC730101
LRRC8E	LOK
LRRC9	LONP1
LRRCC1	LONP2
LRRFIP1	LONRF1
LRRFIP1A	LONRF2
LRRFIP2	LONRF3
LRRIQ1	LORICRIN
LRRIQ3	LOX
LRRK1	LOXL1
LRRK2	LOXL1-AS1
LRRN1	LOXL2
LRRN2	LOXL3
LRRN3	LOXL3B
LRRN4	LOXL4
LRRN4CL	LOXL5B
LRRTM1	LPA
LRRTM2	LPAL2
LRRTM4	LPAR1
LRSAM1	LPAR2
LRTM1	LPAR2B
LRTM2	LPAR3
LRTOMT	LPAR4
LRWD1	LPAR5
LSAMP	LPAR6
LSG1	LPCAT1
LSM1	LPCAT2
LSM10	LPCAT2B
LSM11	LPCAT3

LSM12	LPCAT4
LSM14A	LPGAT1
LSM14B	LPIN1
LSM2	LPIN2
LSM3	LPIN3
LSM4	LPL
LSM5	LPO
LSM6	LPP
LSM7	LPP-AS2
LSM8	LPPR1
LSMEM1	LPXN
LSMEM2	LRAT
LSP1	LRATD1
LSP1P3	LRATD2
LSR	LRBA
LSS	LRCH1
LST1	LRCH2
LTA	LRCH3
LTA4H	LRCH4
LTB	LRCOL1
LTB4DH	LRE3
LTB4R	LRFN1
LTB4R1	LRFN2
LTB4R2	LRFN3
LTBP1	LRFN4
LTBP2	LRFN5
LTBP3	LRG1
LTBP4	LRGUK
LTBR	LRIF1
LTC4S	LRIG1
LTF	LRIG2
LTK	LRIG3
LTN1	LRIT1
LTV1	LRIT2
LUC7L	LRIT3
LUC7L2	LRMDA

LUC7L3	LRP1
LUM	LRP10
LURAP1	LRP11
LURAP1L	LRP12
LUZP1	LRP1B
LUZP6	LRP2
LXN	LRP2BP
LY49I4	LRP3
LY6A	LRP4
LY6AL	LRP5
LY6B	LRP6
LY6C	LRP8
LY6C2	LRPAP1
LY6D	LRPPRC
LY6E	LRR1
LY6F	LRRC1
LY6G	LRRC10
LY6G5B	LRRC10B
LY6G5C	LRRC14
LY6G6C	LRRC14B
LY6G6D	LRRC15
LY6H	LRRC17
LY6I	LRRC18
LY6K	LRRC19
LY75	LRRC2
LY86	LRRC20
LY86-AS1	LRRC23
LY9	LRRC24
LY96	LRRC25
LYAR	LRRC26
LYC2	LRRC27
LYG1	LRRC28
LYG2	LRRC29
LYL1	LRRC3
LYN	LRRC30A
LYNX1	LRRC31

LYPD1	LRRC32
LYPD2	LRRC34
LYPD3	LRRC36
LYPD5	LRRC37A
LYPD6	LRRC37A11P
LYPD6B	LRRC37A16P
LYPLA1	LRRC37A2
LYPLA2	LRRC37A3
LYPLA2P1	LRRC37A4P
LYPLAL1	LRRC37A5P
LYRM1	LRRC37B
LYRM2	LRRC37BP1
LYRM4	LRRC39
LYRM7	LRRC3B
LYRM9	LRRC4
LYS-4	LRRC40
LYSMD1	LRRC41
LYSMD2	LRRC42
LYSMD3	LRRC43
LYSMD4	LRRC45
LYST	LRRC46
LYVE1	LRRC47
LYZ	LRRC49
LYZ1	LRRC4B
LYZ2	LRRC4C
LYZL4	LRRC51
LYZL6	LRRC52
LZIC	LRRC53
LZTFL1	LRRC55
LZTR1	LRRC56
LZTS1	LRRC57
LZTS2	LRRC58
LZTS3	LRRC59
M03E7.3	LRRC61
M110.8	LRRC63
M162.9	LRRC66

M6PR	LRRC69
MAB21L1	LRRC7
MAB21L2	LRRC70
MAB21L3	LRRC71
MAB21L4	LRRC73
MACC1	LRRC74B
MACF1	LRRC75A
MACIR	LRRC75B
MACO1	LRRC77P
MACO1A	LRRC8A
MACROD1	LRRC8B
MACROD2	LRRC8C
MACROH2A1	LRRC8D
MACROH2A2	LRRC8DB
MAD1L1	LRRC8E
MAD2L1	LRRC9
MAD2L1BP	LRRCC1
MAD2L2	LRRFIP1
MADCAM1	LRRFIP1A
MADD	LRRFIP2
MAEA	LRRIQ1
MAEL	LRRIQ3
MAF	LRRK1
MAF1	LRRK2
MAFA	LRRN1
MAFB	LRRN2
MAFF	LRRN3
MAFG	LRRN4
MAFK	LRRN4CL
MAG	LRRTM1
MAGEA1	LRRTM2
MAGEA10	LRRTM4
MAGEA11	LRSAM1
MAGEA12	LRTM1
MAGEA2	LRTM2
MAGEA2B	LRTOMT

MAGEA3	LRWD1
MAGEA4	LSAMP
MAGEA6	LSG1
MAGEA8	LSINCT5
MAGEA9	LSM1
MAGEA9B	LSM10
MAGEB1	LSM11
MAGEB10	LSM12
MAGEB2	LSM14A
MAGEB6	LSM14B
MAGEB6B	LSM2
MAGEC1	LSM3
MAGED1	LSM4
MAGED2	LSM5
MAGED4	LSM6
MAGED4B	LSM7
MAGEE1	LSM8
MAGEE2	LSMEM1
MAGEF1	LSMEM2
MAGEH1	LSP1
MAGEL2	LSP1P3
MAGI1	LSR
MAGI2	LSS
MAGI2-AS3	LST1
MAGI3	LTA
MAGIX	LTA4H
MAGMAS-PS1	LTB
MAGOH	LTB4DH
MAGOHB	LTB4R
MAGT1	LTB4R1
MAILR	LTB4R2
MAIP1	LTBP1
MAJIN	LTBP2
MAK	LTBP3
MAK10	LTBP4
MAK16	LTBR

MAL	LTC4S
MAL2	LTF
MALAT1	LTK
MALINC1	LTN1
MALL	LTO1
MALSU1	LTV1
MALT1	LUADT1
MALT1-AS1	LUC7L
MAMDC2	LUC7L2
MAMDC4	LUC7L3
MAML1	LUCAT1
MAML2	LUM
MAML3	LURAP1
MAMLD1	LURAP1L
MAMSTR	LUZP1
MAN1A1	LUZP6
MAN1A2	LXN
MAN1B1	LY49I4
MAN1C1	LY6A
MAN2A1	LY6AL
MAN2A2	LY6B
MAN2B1	LY6C
MAN2B2	LY6C2
MAN2C1	LY6D
MANBA	LY6E
MANBAL	LY6F
MANCR	LY6G
MANEA	LY6G5B
MANEAL	LY6G5C
MANF	LY6G6C
MANSC1	LY6G6D
MAO	LY6H
MAOA	LY6I
MAOB	LY6K
MAP1	LY75
MAP10	LY86

MAP1A	LY86-AS1
MAP1B	LY9
MAP1LC3A	LY96
MAP1LC3B	LYAR
MAP1LC3B2	LYC2
MAP1LC3BP1	LYG1
MAP1LC3C	LYG2
MAP1S	LYL1
MAP2	LYN
MAP2K1	LYNX1
MAP2K2	LYPD1
MAP2K3	LYPD2
MAP2K4	LYPD3
MAP2K4P1	LYPD5
MAP2K5	LYPD6
MAP2K6	LYPD6B
MAP2K7	LYPLA1
MAP3K1	LYPLA2
MAP3K10	LYPLA2P1
MAP3K11	LYPLAL1
MAP3K12	LYRM1
MAP3K13	LYRM2
MAP3K14	LYRM4
MAP3K14-AS1	LYRM7
MAP3K15	LYRM9
MAP3K19	LYS-4
MAP3K2	LYSMD1
MAP3K20	LYSMD2
MAP3K21	LYSMD3
MAP3K2-DT	LYSMD4
MAP3K3	LYST
MAP3K4	LYVE1
MAP3K5	LYZ
MAP3K6	LYZ1
MAP3K7	LYZ2
MAP3K7CL	LYZL4

MAP3K8	LYZL6
MAP3K9	LZIC
MAP3K9-DT	LZTFL1
MAP4	LZTR1
MAP4K1	LZTS1
MAP4K2	LZTS2
MAP4K3	LZTS3
MAP4K4	M03E7.3
MAP4K5	M110.8
MAP6	M162.9
MAP6D1	M6PR
MAP7	MAB21L1
MAP7D1	MAB21L2
MAP7D2	MAB21L3
MAP7D3	MAB21L4
MAP9	MACC1
MAPK1	MACF1
MAPK10	MACIR
MAPK11	MACO1
MAPK12	MACO1A
MAPK13	MACROD1
MAPK14	MACROD2
MAPK15	MACROH2A1
MAPK1IP1L	MACROH2A2
MAPK3	MAD1L1
MAPK4	MAD2L1
MAPK6	MAD2L1BP
MAPK6-DT	MAD2L2
MAPK7	MADCAM1
MAPK8	MADD
MAPK8B	MAEA
MAPK8IP1	MAEL
MAPK8IP1P2	MAF
MAPK8IP2	MAF1
MAPK8IP3	MAFA
MAPK9	MAFA-AS1

MAPKAP1	MAFB
MAPKAPK2	MAFF
MAPKAPK3	MAFG
MAPKAPK5	MAFG-DT
MAPKAPK5-AS1	MAFK
MAPKBP1	MAG
MAPRE1	MAGEA1
MAPRE2	MAGEA10
MAPRE3	MAGEA11
MAPT	MAGEA12
MAPT-AS1	MAGEA2
MARCHF1	MAGEA2B
MARCHF10	MAGEA3
MARCHF11	MAGEA4
MARCHF2	MAGEA6
MARCHF3	MAGEA8
MARCHF4	MAGEA9
MARCHF5	MAGEA9B
MARCHF6	MAGEB1
MARCHF7	MAGEB10
MARCHF8	MAGEB2
MARCHF9	MAGEB6
MARCKS	MAGEB6B
MARCKSL1	MAGEC1
MARCO	MAGEC2
MARF1	MAGED1
MARK1	MAGED2
MARK2	MAGED4
MARK3	MAGED4B
MARK4	MAGEE1
MARS1	MAGEE2
MARS2	MAGEF1
MARVELD1	MAGEH1
MARVELD2	MAGEL2
MARVELD3	MAGI1
MAS1	MAGI2

MAS1L	MAGI2-AS3
MASP1	MAGI3
MASP2	MAGIX
MAST1	MAGMAS-PS1
MAST2	MAGOH
MAST3	MAGOHB
MAST4	MAGT1
MASTL	MAILR
MAT1A	MAIP1
MAT2A	MAJIN
MAT2B	MAK
MATCAP1	MAK10
MATCAP2	MAK16
MATH-43	MAL
MATK	MAL2
MATN1	MALAT1
MATN2	MALINC1
MATN3	MALL
MATN4	MALSU1
MATR3	MALT1
MAU2	MALT1-AS1
MAVS	MAMDC2
MAX	MAMDC4
MAZ	MAML1
MB	MAML2
MB21D2	MAML3
MBD1	MAMLD1
MBD2	MAMSTR
MBD3	MAN1A1
MBD3B	MAN1A2
MBD3L2	MAN1B1
MBD3L3	MAN1C1
MBD3L4	MAN2A1
MBD3L5	MAN2A2
MBD4	MAN2B1
MBD5	MAN2B2

MBD6	MAN2C1
MBIP	MANBA
MBL1	MANBAL
MBL1P	MANCR
MBL2	MANEA
MBLAC1	MANEAL
MBLAC2	MANF
MBNL1	MANSC1
MBNL1-AS1	MAO
MBNL2	MAOA
MBNL3	MAOB
MBOAT1	MAP1
MBOAT2	MAP10
MBOAT7	MAP1A
MBP	MAP1B
MBPA	MAP1LC3A
MBPB	MAP1LC3B
MBTD1	MAP1LC3B2
MBTPS1	MAP1LC3BP1
MBTPS2	MAP1LC3C
MC1R	MAP1S
MC2R	MAP2
MC4R	MAP2K1
MC5R	MAP2K2
MC5RA	MAP2K3
MCAM	MAP2K4
MCAT	MAP2K4P1
MCC	MAP2K5
MCCC1	MAP2K6
MCCC2	MAP2K7
MCEE	MAP3K1
MCEMP1	MAP3K10
MCF2	MAP3K11
MCF2L	MAP3K12
MCF2L2	MAP3K13
MCF2L-AS1	MAP3K14

MCFD2	MAP3K14-AS1
MCHR1	MAP3K15
MCHR2	MAP3K19
MCIDAS	MAP3K2
MCL1	MAP3K20
MCL1A	MAP3K21
MCL1B	MAP3K2-DT
MCM10	MAP3K3
MCM2	MAP3K4
MCM3	MAP3K5
MCM3AP	MAP3K6
MCM3AP-AS1	MAP3K7
MCM4	MAP3K7CL
MCM4.S	MAP3K8
MCM5	MAP3K9
MCM5.S	MAP3K9-DT
MCM6	MAP4
MCM7	MAP4K1
MCM7.S	MAP4K2
MCM8	MAP4K3
MCM9	MAP4K4
MCMBP	MAP4K5
MCMDC2	MAP6
MCOLN1	MAP6D1
MCOLN2	MAP7
MCOLN3	MAP7D1
MCPH1	MAP7D2
MCPT1	MAP7D3
MCPT10	MAP9
MCPT2	MAPK1
MCPT4	MAPK10
MCPT8	MAPK11
MCPT9	MAPK12
MCRIP1	MAPK13
MCRIP2	MAPK14
MCRS1	MAPK15

MCTP1	MAPK1IP1L
MCTP2	MAPK3
MCTS1	MAPK4
MCTS2	MAPK6
MCU	MAPK6-DT
MCUB	MAPK7
MCUR1	MAPK8
MDC1	MAPK8B
MDFI	MAPK8IP1
MDFIC	MAPK8IP1P2
MDGA1	MAPK8IP2
MDGA2	MAPK8IP3
MDH1	MAPK9
MDH1AA	MAPKAP1
MDH1B	MAPKAPK2
MDH2	MAPKAPK3
MDK	MAPKAPK5
MDK-PS1	MAPKAPK5-AS1
MDM1	MAPKBP1
MDM2	MAPRE1
MDM4	MAPRE2
MDN1	MAPRE3
MDP1	MAPT
MDR49	MAPT-AS1
MDR50	MARCHF1
MDR65	MARCHF10
MDS2	MARCHF11
ME1	MARCHF2
ME2	MARCHF3
ME3	MARCHF4
MEA1	MARCHF5
MEAF6	MARCHF6
MEAK7	MARCHF7
MECOM	MARCHF8
MECP2	MARCHF9
MECR	MARCKS

MED1	MARCKSL1
MED10	MARCO
MED11	MARF1
MED12	MARK1
MED12L	MARK2
MED13	MARK3
MED13L	MARK4
MED14	MARS1
MED14OS	MARS2
MED15	MARVELD1
MED16	MARVELD2
MED17	MARVELD3
MED18	MAS1
MED19	MAS1L
MED20	MASP1
MED21	MASP2
MED22	MAST1
MED23	MAST2
MED24	MAST3
MED25	MAST4
MED26	MASTL
MED27	MAT1A
MED28	MAT2A
MED29	MAT2B
MED30	MATCAP1
MED31	MATCAP2
MED4	MATH-43
MED6	MATK
MED7	MATN1
MED8	MATN2
MED9	MATN3
MEDAG	MATN4
MEF2	MATR3
MEF2A	MAU2
MEF2B	MAVS
MEF2C	MAX

MEF2CA	MAZ
MEF2D	MB
MEFV	MB21D2
MEG3	MBD1
MEG8	MBD2
MEGF10	MBD3
MEGF11	MBD3B
MEGF6	MBD3L2
MEGF8	MBD3L3
MEGF9	MBD3L4
MEI1	MBD3L5
MEIG1	MBD4
MEIOB	MBD5
MEIOC	MBD6
MEIS1	MBIP
MEIS1-AS3	MBL1
MEIS2	MBL1P
MEIS3P1	MBL2
MELK	MBLAC1
MELTF	MBLAC2
MELTF-AS1	MBNL1
MEMO1	MBNL1-AS1
MEN	MBNL2
MEN1	MBNL3
MEOX1	MBOAT1
MEOX2	MBOAT2
MEP1A	MBOAT7
MEP1A.1	MBP
MEP1A.2	MBPA
MEP1B	MBPB
MEPCE	MBTD1
MEPE	MBTPS1
MERTK	MBTPS2
MESD	MC1R
MESP1	MC2R
MESP2	MC4R

MESPA.S	MC5R
MESPBA	MC5RA
MEST	MCAM
MET	MCAT
METAP1	MCC
METAP1D	MCCC1
METAP2	MCCC2
METRN	MCCD1P1
METRNL	MCEE
METTL1	MCEMP1
METTL10	MCF2
METTL11A	MCF2L
METTL13	MCF2L2
METTL14	MCF2L-AS1
METTL15	MCFD2
METTL16	MCHR1
METTL17	MCHR2
METTL18	MCIDAS
METTL21A	MCL1
METTL21E	MCL1A
METTL22	MCL1B
METTL23	MCM10
METTL24	MCM2
METTL25	MCM3
METTL25B	MCM3AP
METTL26	MCM3AP-AS1
METTL27	MCM4
METTL2B	MCM4.S
METTL3	MCM5
METTL4	MCM5.S
METTL5	MCM6
METTL6	MCM7
METTL7A	MCM7.S
METTL7A1	MCM8
METTL7A2	MCM9
METTL7B	MCMBP

METTL8	MCMDC2
METTL9	MCOLN1
MEV-1	MCOLN2
MEX3A	MCOLN3
MEX3B	MCPH1
MEX3C	MCPT1
MEX3D	MCPT10
MFAP1	MCPT2
MFAP2	MCPT4
MFAP3	MCPT8
MFAP3L	MCPT9
MFAP4	MCRIP1
MFAP5	MCRIP2
MFF	MCRS1
MFGE8	MCTP1
MFHAS1	MCTP2
MFN1	MCTS1
MFN2	MCTS2
MFNG	MCU
MFSD1	MCUB
MFSD10	MCUR1
MFSD11	MDC1
MFSD12	MDFI
MFSD12A	MDFIC
MFSD14A	MDGA1
MFSD14B	MDGA2
MFSD14CP	MDH1
MFSD2	MDH1AA
MFSD2A	MDH1B
MFSD2B	MDH2
MFSD3	MDK
MFSD4A	MDK-PS1
MFSD4B	MDM1
MFSD5	MDM2
MFSD6	MDM4
MFSD6B	MDN1

MFSD6L	MDP1
MFSD8	MDR49
MFSD9	MDR50
MGA	MDR65
MGAA	MDS2
MGAM	ME1
MGAM2	ME2
MGARP	ME3
MGAT1	MEA1
MGAT2	MEAF6
MGAT3	MEAK7
MGAT4A	MECOM
MGAT4B	MECP2
MGAT4C	MECR
MGAT4D	MED1
MGAT5	MED10
MGAT5B	MED11
MGL2	MED12
MGLL	MED12L
MGME1	MED13
MGMT	MED13L
MGP	MED14
MGRN1	MED14OS
MGST1	MED15
MGST2	MED16
MGST3	MED17
MHC1LAA	MED18
MHC1ZBA	MED19
MHENCR	MED20
MIA	MED21
MIA2	MED22
MIA2-AS1	MED23
MIA3	MED24
MIAT	MED25
MIB1	MED26
MIB2	MED27

MIBP	MED28
MICA	MED29
MICAL1	MED30
MICAL2	MED31
MICAL3	MED4
MICALL1	MED6
MICALL2	MED7
MICB	MED8
MICOS10	MED9
MICOS10P1	MEDAG
MICOS13	MEF2
MICU1	MEF2A
MICU2	MEF2B
MICU3	MEF2C
MID1	MEF2CA
MID1IP1	MEF2D
MID2	MEFV
MIDEAS	MEG3
MIDN	MEG8
MIEF1	MEGF10
MIEF2	MEGF11
MIEN1	MEGF6
MIER1	MEGF8
MIER1A	MEGF9
MIER2	MEI1
MIER3	MEIG1
MIF	MEIOB
MIF4GD	MEIOC
MIGA1	MEIS1
MIIP	MEIS1-AS3
MILIP	MEIS2
MILR1	MEIS3P1
MIMT1	MELK
MINAR1	MELTF
MINCR	MELTF-AS1
MINDY1	MEMO1

MINDY2	MEN
MINDY3	MEN1
MINDY4	MEOX1
MINK1	MEOX2
MINPP1	MEP1A
MIOS	MEP1A.1
MIOX	MEP1A.2
MIP	MEP1B
MIPB	MEPCE
MIPEP	MEPE
MIPEPP3	MERTK
MIPOL1	MESD
MIR1	MESP1
MIR100	MESP2
MIR100HG	MESPA.S
MIR101-1	MESPBA
MIR101A	MEST
MIR101B	MET
MIR101C	METAP1
MIR103A1	METAP1D
MIR103A2	METAP2
MIR105-1	METRN
MIR106A	METRNL
MIR106B	METTL1
MIR107	METTL10
MIR10A	METTL11A
MIR10B	METTL13
MIR1-1	METTL14
MIR1180	METTL15
MIR1181	METTL16
MIR1194	METTL17
MIR1-1HG	METTL18
MIR1202	METTL21A
MIR1204	METTL21E
MIR1207	METTL22
MIR1208	METTL23

MIR122	METTL24
MIR1224	METTL25
MIR1225	METTL25B
MIR1228	METTL26
MIR1231	METTL27
MIR124-1HG	METTL2B
MIR124-2HG	METTL3
MIR124-3	METTL4
MIR1246	METTL5
MIR124A-1	METTL6
MIR1251	METTL7A
MIR125A	METTL7A1
MIR125B2	METTL7A2
MIR126	METTL7B
MIR1260A	METTL8
MIR1260B	METTL9
MIR1268A	MEV-1
MIR1268B	MEX3A
MIR126A	MEX3B
MIR127	MEX3C
MIR1275	MEX3D
MIR128-1	MFAP1
MIR1282	MFAP2
MIR128-2	MFAP3
MIR1287	MFAP3L
MIR1290	MFAP4
MIR129-1	MFAP5
MIR129-2	MFF
MIR1299	MFGE8
MIR1305	MFHAS1
MIR1307	MFN1
MIR130A	MFN2
MIR130B	MFNG
MIR132	MFSD1
MIR1322	MFSD10
MIR133	MFSD11

MIR133A1	MFSD12
MIR133A-1	MFSD12A
MIR133B	MFSD14A
MIR134	MFSD14B
MIR135A	MFSD14CP
MIR135A1	MFSD2
MIR135A-1	MFSD2A
MIR135A-2	MFSD2B
MIR135B	MFSD3
MIR136	MFSD4A
MIR137	MFSD4B
MIR137HG	MFSD5
MIR138	MFSD6
MIR138-1	MFSD6B
MIR138-2	MFSD6L
MIR139	MFSD8
MIR140	MFSD9
MIR141	MGA
MIR142	MGAA
MIR143	MGAM
MIR144	MGAM2
MIR145	MGARP
MIR145A	MGAT1
MIR146A	MGAT2
MIR146B	MGAT3
MIR1471	MGAT4A
MIR148A	MGAT4B
MIR148B	MGAT4C
MIR149	MGAT4D
MIR150	MGAT5
MIR151	MGAT5B
MIR151A	MGL2
MIR152	MGLL
MIR153	MGME1
MIR1538	MGMT
MIR154	MGP

MIR155	MGRN1
MIR155HG	MGST1
MIR15A	MGST2
MIR15B	MGST3
MIR16	MHC1LAA
MIR16-1	MHC1ZBA
MIR16-2	MHENCR
MIR17	MIA
MIR17HG	MIA2
MIR181A1HG	MIA2-AS1
MIR181A2	MIA3
MIR181A2HG	MIAT
MIR181B1	MIB1
MIR181B2	MIB2
MIR181C	MIBP
MIR181D	MICA
MIR182	MICAL1
MIR1825	MICAL2
MIR183	MICAL3
MIR1839	MICALL1
MIR184	MICALL2
MIR185	MICB
MIR186	MICOS10
MIR187	MICOS10-NBL1
MIR188	MICOS10P1
MIR1897	MICOS13
MIR18A	MICU1
MIR18B	MICU2
MIR1902	MICU3
MIR1903	MID1
MIR1908	MID1IP1
MIR1909	MID2
MIR190B	MIDEAS
MIR191	MIDN
MIR1914	MIEF1
MIR1915	MIEF2

MIR1915HG	MIEN1
MIR192	MIER1
MIR193A	MIER1A
MIR193B	MIER2
MIR194	MIER3
MIR195	MIF
MIR1952	MIF4GD
MIR196A1	MIF-AS1
MIR196B	MIGA1
MIR196C	MIIP
MIR197	MILIP
MIR1973	MILR1
MIR198	MIMT1
MIR1983	MINAR1
MIR199B	MINCR
MIR19A	MINDY1
MIR1A-1	MINDY2
MIR1B	MINDY3
MIR200A	MINDY4
MIR200B	MINK1
MIR200C	MINPP1
MIR202	MIOS
MIR203	MIOX
MIR203A	MIP
MIR204	MIPB
MIR205	MIPEP
MIR205HG	MIPEPP3
MIR206	MIPOL1
MIR207	MIR1
MIR208A	MIR100
MIR20A	MIR100HG
MIR20B	MIR101-1
MIR21	MIR101A
MIR210	MIR101B
MIR210HG	MIR101C
MIR211	MIR103A1

MIR2110	MIR103A2
MIR212	MIR105-1
MIR2137	MIR106A
MIR214	MIR106B
MIR215	MIR107
MIR216B	MIR10A
MIR217	MIR10B
MIR218	MIR1-1
MIR218-2	MIR1179
MIR21A	MIR1180
MIR22	MIR1181
MIR221	MIR1182
MIR222	MIR1183
MIR223	MIR1194
MIR224	MIR1197
MIR2278	MIR1-1HG
MIR22HG	MIR1-2
MIR23A	MIR1202
MIR23B	MIR1204
MIR24-1	MIR1207
MIR24-2	MIR1208
MIR25	MIR122
MIR26A	MIR1224
MIR26A1	MIR1225
MIR26B	MIR1228
MIR27A	MIR1231
MIR27B	MIR1238
MIR28	MIR124-1
MIR296	MIR124-1HG
MIR297	MIR124-2
MIR297A-2	MIR124-2HG
MIR299	MIR124-3
MIR29A	MIR1244-1
MIR29B	MIR1246
MIR29B1	MIR124A-1
MIR29B2	MIR1251

MIR29C	MIR1253
MIR301	MIR1256
MIR301A	MIR1258
MIR301B	MIR125A
MIR302B	MIR125B1
MIR302C	MIR125B2
MIR302D	MIR126
MIR3065	MIR1260A
MIR3081	MIR1260B
MIR3087	MIR1268A
MIR3095	MIR1268B
MIR3099	MIR126A
MIR30A	MIR127
MIR30B	MIR1271
MIR30C1	MIR1275
MIR30C2	MIR128-1
MIR30C-2	MIR1282
MIR30D	MIR128-2
MIR30E	MIR1287
MIR31	MIR1290
MIR3102	MIR129-1
MIR3127	MIR129-2
MIR3135B	MIR1297
MIR3142HG	MIR1298
MIR3148	MIR1299
MIR3149	MIR1304
MIR3150BHG	MIR1305
MIR3178	MIR1307
MIR3188	MIR130A
MIR3195	MIR130B
MIR3196	MIR132
MIR31HG	MIR1322
MIR32	MIR133
MIR320	MIR133A1
MIR3200	MIR133A-1
MIR320A	MIR133B

MIR322	MIR134
MIR323	MIR135A
MIR323A	MIR135A1
MIR323B	MIR135A-1
MIR324	MIR135A-2
MIR326	MIR135B
MIR327	MIR136
MIR328	MIR137
MIR331	MIR137HG
MIR335	MIR138
MIR337	MIR138-1
MIR339	MIR138-2
MIR33A	MIR139
MIR33B	MIR140
MIR340	MIR141
MIR342	MIR142
MIR345	MIR143
MIR346	MIR144
MIR3470A	MIR145
MIR3470B	MIR145A
MIR3473	MIR146A
MIR3473B	MIR146B
MIR34A	MIR1471
MIR34B	MIR147A
MIR34C	MIR147B
MIR350	MIR148A
MIR351	MIR148B
MIR352	MIR149
MIR3588	MIR150
MIR361	MIR151
MIR362	MIR151A
MIR363	MIR152
MIR365A	MIR153
MIR365B	MIR1538
MIR3661	MIR154
MIR3665	MIR155

MIR3667HG	MIR155HG
MIR3682	MIR15A
MIR369	MIR15B
MIR370	MIR16
MIR372	MIR16-1
MIR373	MIR16-2
MIR374A	MIR17
MIR374B	MIR17HG
MIR375	MIR181A1
MIR376A1	MIR181A1HG
MIR376B	MIR181A2
MIR376C	MIR181A2HG
MIR378	MIR181B1
MIR378A	MIR181B2
MIR378C	MIR181C
MIR378E	MIR181D
MIR378F	MIR182
MIR379	MIR1825
MIR381	MIR183
MIR382	MIR1839
MIR383	MIR184
MIR384	MIR185
MIR3907	MIR186
MIR3911	MIR187
MIR3916	MIR188
MIR3936	MIR1897
MIR3936HG	MIR18A
MIR3940	MIR18B
MIR3945	MIR1902
MIR3960	MIR1903
MIR3965	MIR1908
MIR409	MIR1909
MIR410	MIR190B
MIR411	MIR191
MIR421	MIR1914
MIR422A	MIR1915

MIR423	MIR1915HG
MIR424	MIR192
MIR425	MIR193A
MIR4257	MIR193B
MIR4269	MIR194
MIR4273	MIR195
MIR4286	MIR1952
MIR429	MIR196A1
MIR4298	MIR196A2
MIR431	MIR196B
MIR432	MIR196C
MIR4321	MIR197
MIR4322	MIR1973
MIR4324	MIR1976
MIR433	MIR198
MIR434	MIR1983
MIR4423	MIR199A1
MIR4435-2HG	MIR199B
MIR4443	MIR19A
MIR4453HG	MIR19B1
MIR4454	MIR1A-1
MIR4458HG	MIR1B
MIR4466	MIR200A
MIR4484	MIR200B
MIR4488	MIR200C
MIR4492	MIR202
MIR4496	MIR203
MIR4498	MIR203A
MIR449A	MIR204
MIR449B	MIR205
MIR449C	MIR205HG
MIR4508	MIR206
MIR450A1	MIR207
MIR450B	MIR208A
MIR4516	MIR20A
MIR451A	MIR20B

MIR4521	MIR21
MIR4530	MIR210
MIR454	MIR210HG
MIR455	MIR211
MIR465	MIR2110
MIR4651	MIR212
MIR4664	MIR2137
MIR466D	MIR214
MIR467C	MIR215
MIR467E	MIR216A
MIR467H	MIR216B
MIR4690	MIR217
MIR4707	MIR218
MIR4722	MIR218-1
MIR4732	MIR218-2
MIR4737	MIR219A1
MIR4742	MIR21A
MIR4745	MIR22
MIR4750	MIR221
MIR4787	MIR222
MIR483	MIR223
MIR484	MIR224
MIR485	MIR2278
MIR486	MIR22HG
MIR486-1	MIR23A
MIR487B	MIR23B
MIR488	MIR24-1
MIR489	MIR24-2
MIR491	MIR25
MIR493	MIR26A
MIR494	MIR26A1
MIR495	MIR26B
MIR497	MIR27A
MIR499	MIR27B
MIR500	MIR28
MIR501	MIR296

MIR503	MIR297
MIR503HG	MIR297A-2
MIR504	MIR299
MIR505	MIR29A
MIR507	MIR29B
MIR508	MIR29B1
MIR5103	MIR29B2
MIR5107	MIR29C
MIR513C	MIR300
MIR517A	MIR301
MIR5189	MIR301A
MIR518B	MIR301B
MIR518C	MIR302A
MIR518F	MIR302B
MIR519B	MIR302C
MIR520H	MIR302D
MIR525	MIR3065
MIR532	MIR3081
MIR539	MIR3087
MIR541	MIR3095
MIR542	MIR3099
MIR547	MIR30A
MIR548AP	MIR30B
MIR548I4	MIR30C1
MIR548V	MIR30C2
MIR551B	MIR30C-2
MIR556	MIR30D
MIR557	MIR30E
MIR5585	MIR31
MIR568	MIR3102
MIR569	MIR3120
MIR570	MIR3127
MIR571	MIR3135B
MIR572	MIR3142HG
MIR574	MIR3148
MIR575	MIR3149

MIR576	MIR3150BHG
MIR577	MIR3163
MIR582	MIR3178
MIR584	MIR3188
MIR586	MIR3195
MIR590	MIR3196
MIR592	MIR31HG
MIR595	MIR32
MIR600HG	MIR320
MIR601	MIR3200
MIR605	MIR320A
MIR610	MIR322
MIR612	MIR323
MIR615	MIR323A
MIR6215	MIR323B
MIR622	MIR324
MIR625	MIR325
MIR628	MIR326
MIR629	MIR327
MIR630	MIR328
MIR631	MIR330
MIR632	MIR331
MIR6366	MIR335
MIR637	MIR337
MIR6370	MIR338
MIR638	MIR339
MIR639	MIR33A
MIR642A	MIR33B
MIR642B	MIR340
MIR645	MIR342
MIR646HG	MIR345
MIR652	MIR346
MIR654	MIR3470A
MIR657	MIR3470B
MIR659	MIR3473
MIR663A	MIR3473B

MIR663AHG	MIR34A
MIR663B	MIR34B
MIR665	MIR34C
MIR667	MIR350
MIR669C	MIR351
MIR669D	MIR352
MIR670HG	MIR3588
MIR671	MIR361
MIR672	MIR3619
MIR673	MIR362
MIR674	MIR363
MIR675	MIR365A
MIR680-2	MIR365B
MIR687	MIR3661
MIR691	MIR3665
MIR692-1	MIR3666
MIR696	MIR3667HG
MIR6968	MIR367
MIR6980	MIR3682
MIR700	MIR369
MIR7018	MIR370
MIR702	MIR372
MIR7039	MIR373
MIR706	MIR374A
MIR708	MIR374B
MIR7081	MIR375
MIR7-1	MIR376A1
MIR7109	MIR376B
MIR717	MIR376C
MIR7-3	MIR377
MIR7-3HG	MIR378
MIR743	MIR378A
MIR743B	MIR378C
MIR744	MIR378E
MIR760	MIR378F
MIR761	MIR379

MIR762	MIR381
MIR765	MIR382
MIR769	MIR383
MIR770	MIR384
MIR7977	MIR3907
MIR802	MIR3911
MIR872	MIR3916
MIR874	MIR3936
MIR885	MIR3936HG
MIR888	MIR3940
MIR9-1	MIR3945
MIR9-1HG	MIR3960
MIR921	MIR3965
MIR924HG	MIR409
MIR92A1	MIR410
MIR92A2	MIR411
MIR92B	MIR421
MIR93	MIR422A
MIR935	MIR423
MIR937	MIR424
MIR939	MIR425
MIR9-3HG	MIR4257
MIR96	MIR4262
MIR98	MIR4269
MIR99A	MIR4270
MIR99AHG	MIR4273
MIR99B	MIR4286
MIRLET7A1	MIR429
MIRLET7B	MIR4293
MIRLET7BHG	MIR4298
MIRLET7C	MIR4299
MIRLET7C1	MIR4301
MIRLET7C-1	MIR431
MIRLET7C2	MIR4317
MIRLET7C-2	MIR4319
MIRLET7D	MIR432

MIRLET7E	MIR4321
MIRLET7G	MIR4322
MIRLET7I	MIR4324
MIRT2	MIR433
MIS12	MIR434
MIS18A	MIR4423
MIS18BP1	MIR4435-2HG
MISP	MIR4443
MISP3	MIR4453HG
MITD1	MIR4454
MITF	MIR4458
MITFA	MIR4458HG
MIX23	MIR4465
MIXL1	MIR4466
MKI67	MIR448
MKI67IP	MIR4484
MKKS	MIR4488
MKLN1	MIR4492
MKNK1	MIR4496
MKNK1-AS1	MIR4498
MKNK2	MIR449A
MKNK2B	MIR449B
MKP3	MIR449C
MKRN1	MIR4500
MKRN2	MIR4508
MKS1	MIR450A1
MKX	MIR450B
MLANA	MIR4516
MLC1	MIR451A
MLEC	MIR452
MLF1	MIR4521
MLF2	MIR4530
MLH1	MIR454
MLH3	MIR455
MLIP	MIR465
MLKL	MIR4651

MLLT1	MIR4664
MLLT10	MIR466D
MLLT10P1	MIR4677
MLLT11	MIR467C
MLLT3	MIR467E
MLLT6	MIR467H
MLN	MIR4690
MLNR	MIR4707
MLPH	MIR4722
MLST8	MIR4732
MLX	MIR4735
MLXIP	MIR4737
MLXIPL	MIR4742
MLYCD	MIR4745
MMAA	MIR4750
MMAB	MIR4782
MMACHC	MIR4787
MMADHC	MIR483
MMD	MIR484
MMD2	MIR485
MME	MIR486
MMEL1	MIR486-1
MMGT1	MIR487B
MMGT2	MIR488
MMP1	MIR489
MMP10	MIR491
MMP11	MIR493
MMP12	MIR494
MMP13	MIR495
MMP14	MIR496
MMP14A	MIR497
MMP15	MIR498
MMP16	MIR499
MMP17	MIR499A
MMP19	MIR500
MMP1B	MIR500A

MMP2	MIR501
MMP20	MIR502
MMP21	MIR503
MMP23	MIR503HG
MMP23A	MIR504
MMP23B	MIR505
MMP24	MIR506
MMP24OS	MIR507
MMP25	MIR508
MMP26	MIR509-1
MMP28	MIR510
MMP3	MIR5100
MMP7	MIR5103
MMP8	MIR5107
MMP9	MIR511
MMRN1	MIR513C
MMRN2	MIR517A
MMS19	MIR5189
MMS22L	MIR518B
MMUT	MIR518C
MN1	MIR518F
MNAT1	MIR5195
MND1	MIR519B
MNDA	MIR519D
MNDAL	MIR520A
MNS1	MIR520E
MNT	MIR520F
MNX1	MIR520H
MNX1-AS1	MIR522
MOAP1	MIR525
MOB1A	MIR526B
MOB1B	MIR527
MOB2	MIR532
MOB3A	MIR539
MOB3B	MIR541
MOB3C	MIR542

MOB4	MIR543
MOBP	MIR545
MOCOS	MIR547
MOCS1	MIR548AP
MOCS2	MIR548I4
MOCS3	MIR548L
MOG	MIR548V
MOGAT1	MIR551B
MOGAT2	MIR556
MOGAT3	MIR557
MOGS	MIR5585
MOK	MIR561
MON1A	MIR568
MON1B	MIR569
MON2	MIR570
MORC1	MIR5702
MORC2	MIR571
MORC2-AS1	MIR572
MORC3	MIR574
MORC4	MIR575
MORF4L1	MIR576
MORF4L2	MIR577
MORF4L2-AS1	MIR582
MORG1	MIR584
MORN1	MIR585
MORN2	MIR586
MORN3	MIR589
MORN4	MIR590
MORN5	MIR592
MOS	MIR593
MOSMO	MIR595
MOSPD1	MIR598
MOSPD2	MIR599
MOSPD3	MIR600
MOV10	MIR600HG
MOV10B.1	MIR601

MOV10L1	MIR605
MOXD1	MIR608
MPC1	MIR610
MPC2	MIR612
MPDU1	MIR613
MPDU1A	MIR615
MPDZ	MIR616
MPEG1	MIR621
MPG	MIR6215
MPHOSPH10	MIR622
MPHOSPH6	MIR625
MPHOSPH8	MIR628
MPHOSPH9	MIR629
MPI	MIR630
MPIG6B	MIR631
MPL	MIR632
MPLKIP	MIR635
MPND	MIR6366
MPO	MIR637
MPO.S	MIR6370
MPP1	MIR638
MPP2	MIR639
MPP3	MIR641
MPP4	MIR642A
MPP7	MIR642B
MPPE1	MIR645
MPPED1	MIR646HG
MPPED2	MIR647
MPPED2-AS1	MIR650
MPRIP	MIR6507
MPST	MIR652
MPV17	MIR653
MPV17L	MIR654
MPV17L2	MIR655
MPZ	MIR657
MPZL1	MIR659

MPZL2	MIR660
MPZL3	MIR661
MR1	MIR663A
MRAP	MIR663AHG
MRAP2	MIR663B
MRAS	MIR665
MRC1	MIR667
MRC2	MIR669C
MRCL3	MIR669D
MRE11	MIR670HG
MRE11A	MIR671
MREG	MIR672
MRFAP1L1	MIR673
MRGBP	MIR674
MRGPRA2B	MIR675
MRGPRB2	MIR6754
MRGPRB8	MIR680-2
MRGPRE	MIR687
MRGPRF	MIR691
MRGPRG	MIR692-1
MRGPRG-AS1	MIR696
MRGPRX1	MIR6968
MRGPRX2	MIR6980
MRGPRX4	MIR700
MRI1	MIR7018
MRLN	MIR702
MRM1	MIR7039
MRM2	MIR706
MRM3	MIR708
MRNIP	MIR7081
MRO	MIR7-1
MROCKI	MIR7109
MROH1	MIR717
MROH2A	MIR718
MROH2B	MIR7-2
MROH5	MIR7-3

MROH6	MIR7-3HG
MROH7	MIR743
MROH8	MIR743B
MROH9	MIR744
MRP4	MIR758
MRPL1	MIR760
MRPL10	MIR761
MRPL11	MIR762
MRPL12	MIR765
MRPL13	MIR769
MRPL14	MIR770
MRPL15	MIR7977
MRPL16	MIR802
MRPL17	MIR872
MRPL18	MIR873
MRPL19	MIR874
MRPL2	MIR875
MRPL20	MIR877
MRPL21	MIR885
MRPL22	MIR888
MRPL23	MIR889
MRPL23-AS1	MIR9-1
MRPL24	MIR9-1HG
MRPL27	MIR921
MRPL28	MIR924HG
MRPL3	MIR92A1
MRPL30	MIR92A2
MRPL32	MIR92B
MRPL33	MIR93
MRPL34	MIR9-3
MRPL35	MIR935
MRPL36	MIR936
MRPL37	MIR937
MRPL38	MIR939
MRPL39	MIR9-3HG
MRPL4	MIR940

MRPL40	MIR942
MRPL41	MIR944
MRPL42	MIR95
MRPL42P5	MIR96
MRPL43	MIR98
MRPL44	MIR99A
MRPL45	MIR99AHG
MRPL46	MIR99B
MRPL47	MIRLET7A1
MRPL48	MIRLET7A2
MRPL49	MIRLET7A3
MRPL50	MIRLET7B
MRPL51	MIRLET7BHG
MRPL52	MIRLET7C
MRPL53	MIRLET7C1
MRPL54	MIRLET7C-1
MRPL55	MIRLET7C2
MRPL57	MIRLET7C-2
MRPL9	MIRLET7D
MRPS10	MIRLET7E
MRPS11	MIRLET7F1
MRPS12	MIRLET7G
MRPS14	MIRLET7I
MRPS15	MIRT2
MRPS16	MIS12
MRPS17	MIS18A
MRPS18A	MIS18BP1
MRPS18B	MISP
MRPS18C	MISP3
MRPS2	MITD1
MRPS21	MITF
MRPS22	MITFA
MRPS23	MIX23
MRPS24	MIXL1
MRPS25	MKI67
MRPS26	MKI67IP

MRPS27	MKKS
MRPS28	MKLN1
MRPS30	MKNK1
MRPS31	MKNK1-AS1
MRPS33	MKNK2
MRPS34	MKNK2B
MRPS35	MKP3
MRPS36	MKRN1
MRPS5	MKRN2
MRPS6	MKS1
MRPS7	MKX
MRPS9	MLANA
MRRF	MLC1
MRS2	MLEC
MRTFA	MLF1
MRTFA-AS1	MLF2
MRTFB	MLH1
MRTO4	MLH3
MS4A1	MLIP
MS4A10	MLKL
MS4A13	MLLT1
MS4A14	MLLT10
MS4A17A.8	MLLT10P1
MS4A2	MLLT11
MS4A3	MLLT3
MS4A4B	MLLT6
MS4A4C	MLN
MS4A4D	MLNR
MS4A5	MLPH
MS4A6A	MLRL
MS4A6B	MLST8
MS4A6BL	MLX
MS4A6C	MLXIP
MS4A6D	MLXIPL
MS4A6E	MLYCD
MS4A7	MMAA

MS4A8	MMAB
MSANTD1	MMACHC
MSANTD2	MMADHC
MSANTD3	MMD
MSANTD3-TMEFF1	MMD2
MSANTD4	MME
MSC	MMEL1
MSC-AS1	MMGT1
MSD-2	MMGT2
MSF	MMP1
MSGN1	MMP10
MSH2	MMP11
MSH3	MMP12
MSH4	MMP13
MSH5	MMP14
MSH6	MMP14A
MSI1	MMP15
MSI2	MMP16
MSL1	MMP17
MSL2	MMP19
MSL3	MMP1B
MSL3L2	MMP2
MSL3P1	MMP20
MSLN	MMP21
MSLNL	MMP23
MSMB	MMP23A
MSMO1	MMP23B
MSMP	MMP24
MSN	MMP24OS
MSP-10	MMP25
MSP-113	MMP26
MSP-142	MMP28
MSP-152	MMP3
MSP-19	MMP7
MSP-3	MMP8
MSP-33	MMP9

MSP-36	MMRN1
MSP-38	MMRN2
MSP-40	MMS19
MSP-45	MMS22L
MSP-49	MMUT
MSP-50	MN1
MSP-51	MNAT1
MSP-53	MND1
MSP-55	MNDA
MSP-56	MNDAL
MSP-57	MNS1
MSP-59	MNS16A
MSP-64	MNT
MSP-65	MNX1
MSP-76	MNX1-AS1
MSP-77	MOAP1
MSP-78	MOB1A
MSP-81	MOB1B
MSR1	MOB2
MSR-110	MOB3A
MSRA	MOB3B
MSRB1	MOB3C
MSRB2	MOB4
MSRB3	MOBP
MSS51	MOCOS
MST1	MOCS1
MST1L	MOCS2
MST1P2	MOCS3
MST1R	MOG
MSTN	MOGAT1
MSTO1	MOGAT2
MSX1	MOGAT3
MSX2	MOGS
MSX3	MOK
MT	MON1A
MT1	MON1B

MT1A	MON2
MT1B	MORC1
MT1CP	MORC2
MT1E	MORC2-AS1
MT1F	MORC3
MT1G	MORC4
MT1H	MORF4L1
MT1HL1	MORF4L2
MT1IP	MORF4L2-AS1
MT1JP	MORG1
MT1L	MORN1
MT1M	MORN2
MT1X	MORN3
MT2	MORN4
MT2A	MORN5
MT3	MOS
MTA1	MOSMO
MTA2	MOSPD1
MTA3	MOSPD2
MTAP	MOSPD3
MTARC1	MOV10
MTARC2	MOV10B.1
MTBP	MOV10L1
MTCH1	MOXD1
MTCH2	MPC1
MTCL1	MPC2
MTCP1	MPDU1
MTDH	MPDU1A
MTDHB	MPDZ
MTERF1	MPEG1
MTERF1A	MPG
MTERF2	MPHOSPH10
MTERF3	MPHOSPH6
MTERF4	MPHOSPH8
MTERFD1	MPHOSPH9
MTF	MPI

MTF1	MPIG6B
MTF2	MPL
MTFMT	MPLKIP
MTFP1	MPND
MTFR1	MPO
MTFR1L	MPO.S
MTFR2	MPP1
MTG1	MPP2
MTHFD1	MPP3
MTHFD1L	MPP4
MTHFD2	MPP7
MTHFD2L	MPPE1
MTHFR	MPPED1
MTHFS	MPPED2
MTHFSD	MPPED2-AS1
MTIF2	MPRIP
MTIF3	MPST
MTK	MPV17
MTLN	MPV17L
MTM1	MPV17L2
MTMR1	MPZ
MTMR10	MPZL1
MTMR11	MPZL2
MTMR12	MPZL3
MTMR14	MR1
MTMR2	MRAP
MTMR3	MRAP2
MTMR4	MRAS
MTMR6	MRC1
MTMR7	MRC2
MTMR8	MRCL3
MTMR9	MRE11
MTMR9LP	MRE11A
MTND4P12	MREG
MTND4P24	MRFAP1L1
MTNR1A	MRGBP

MTNR1B	MRGPRA2B
MTO1	MRGPRB2
MTOR	MRGPRB8
MTPAP	MRGPRD
MTPN	MRGPRE
MTR	MRGPRF
MTRES1	MRGPRG
MTREX	MRGPRG-AS1
MTRF1	MRGPRX1
MTRF1L	MRGPRX2
MTRFR	MRGPRX3
MTRNR2L1	MRGPRX4
MTRNR2L12	MRI1
MTRNR2L2	MRLN
MTRNR2L3	MRM1
MTRR	MRM2
MTSS1	MRM3
MTSS2	MRNIP
MTTP	MRO
MTURN	MROCKI
MTUS1	MROH1
MTUS1-DT	MROH2A
MTUS2	MROH2B
MTX1	MROH5
MTX2	MROH6
MTX3	MROH7
MUC1	MROH8
MUC12	MROH9
MUC13	MRP4
MUC15	MRPL1
MUC16	MRPL10
MUC17	MRPL11
MUC19	MRPL12
MUC2	MRPL13
MUC20	MRPL14
MUC21	MRPL15

MUC3A	MRPL16
MUC3B	MRPL17
MUC4	MRPL18
MUC5AC	MRPL19
MUC5B	MRPL2
MUC6	MRPL20
MUC7	MRPL21
MUCL1	MRPL22
MUCL2	MRPL23
MUCL3	MRPL23-AS1
MUG1	MRPL24
MUG2	MRPL27
MUG-PS1	MRPL28
MUL1	MRPL3
MUL-1	MRPL30
MUP1	MRPL32
MUP14	MRPL33
MUP21	MRPL34
MUP3	MRPL35
MUP4	MRPL36
MUP5	MRPL37
MUP6	MRPL38
MUP9	MRPL39
MUS81	MRPL4
MUSK	MRPL40
MUSTN1	MRPL41
MUTYH	MRPL42
MVB12A	MRPL42P5
MVB12B	MRPL43
MVD	MRPL44
MVK	MRPL45
MVP	MRPL46
MX1	MRPL47
MX2	MRPL48
MXC	MRPL49
MXD1	MRPL50

MXD3	MRPL51
MXD4	MRPL52
MXI1	MRPL53
MXRA5	MRPL54
MXRA7	MRPL55
MXRA8	MRPL57
MYADM	MRPL58
MYADML	MRPL9
MYADML2	MRPS10
MYB	MRPS11
MYBBP1A	MRPS12
MYBL1	MRPS14
MYBL2	MRPS15
MYBPC1	MRPS16
MYBPC2	MRPS17
MYBPC2A	MRPS18A
MYBPC2B	MRPS18B
MYBPC3	MRPS18C
MYBPH	MRPS2
MYBPHA	MRPS21
MYBPHL	MRPS22
MYC	MRPS23
MYCBP	MRPS24
MYCBP2	MRPS25
MYCBPAP	MRPS26
MYCL	MRPS27
MYCN	MRPS28
MYCN.L	MRPS30
MYCNOS	MRPS31
MYCS	MRPS33
MYCT1	MRPS34
MYD88	MRPS35
MYDGF	MRPS36
MYEF2	MRPS5
MYEOV	MRPS6
MYEOV2	MRPS7

MYF5	MRPS9
MYF6	MRRF
MYG1	MRS2
MYH1	MRTFA
MYH10	MRTFA-AS1
MYH11	MRTFB
MYH13	MRTO4
MYH14	MS4A1
MYH15	MS4A10
MYH16	MS4A13
MYH2	MS4A14
MYH3	MS4A17A.8
MYH4	MS4A2
MYH6	MS4A3
MYH7	MS4A4B
MYH7B	MS4A4C
MYH8	MS4A4D
MYH9	MS4A5
MYHZ1.1	MS4A6A
MYHZ2	MS4A6B
MYL1	MS4A6BL
MYL10	MS4A6C
MYL11	MS4A6D
MYL12A	MS4A6E
MYL12B	MS4A7
MYL2	MS4A8
MYL3	MSANTD1
MYL4	MSANTD2
MYL5	MSANTD3
MYL6	MSANTD3-TMEFF1
MYL6B	MSANTD4
MYL7	MSC
MYL9	MSC-AS1
MYLIP	MSD-2
MYLIPB	MSF
MYLK	MSGN1

MYLK2	MSH2
MYLK3	MSH3
MYLK4	MSH4
MYLPF	MSH5
MYLPFA	MSH6
MYLPFB	MSI1
MYLZ3	MSI2
MYMK	MSL1
MYNN	MSL2
MYO10	MSL3
MYO15A	MSL3L2
MYO15B	MSL3P1
MYO16	MSLN
MYO16-AS1	MSLNL
MYO18A	MSMB
MYO18B	MSMO1
MYO18B-AS1	MSMP
MYO19	MSN
MYO1B	MSP-10
MYO1C	MSP-113
MYO1D	MSP-142
MYO1E	MSP-152
MYO1F	MSP-19
MYO1G	MSP-3
MYO3A	MSP-33
MYO3B	MSP-36
MYO5A	MSP-38
MYO5B	MSP-40
MYO5C	MSP-45
MYO6	MSP-49
MYO7A	MSP-50
MYO7B	MSP-51
MYO9A	MSP-53
MYO9B	MSP-55
MYOC	MSP-56
MYOCD	MSP-57

MYOD1	MSP-59
MYOF	MSP-64
MYOG	MSP-65
MYOM1	MSP-76
MYOM2	MSP-77
MYOM3	MSP-78
MYORG	MSP-81
MYOT	MSR1
MYOZ1	MSR-110
MYOZ1B	MSRA
MYOZ2	MSRB1
MYOZ3	MSRB2
MYPN	MSRB3
MYPOP	MSS51
MYRF	MST1
MYRF-AS1	MST1L
MYRFL	MST1P2
MYRIP	MST1R
MYSM1	MSTN
MYST2	MSTO1
MYT1	MSX1
MYT1L	MSX2
MYZAP	MSX3
MZB1	MT
MZF1	MT1
MZF1-AS1	MT1A
MZT1	MT1B
MZT2A	MT1CP
MZT2B	MT1E
N4BP1	MT1F
N4BP2	MT1G
N4BP2L1	MT1H
N4BP2L2	MT1HL1
N4BP2L2-IT2	MT1IP
N4BP3	MT1JP
N6AMT1	MT1L

N6AMT2	MT1M
NAA10	MT1X
NAA15	MT2
NAA16	MT2A
NAA20	MT3
NAA25	MT4
NAA30	MTA1
NAA35	MTA2
NAA38	MTA3
NAA40	MTAP
NAA50	MTARC1
NAA60	MTARC2
NAA80	MTBP
NAAA	MTCH1
NAALAD2	MTCH2
NAALADL1	MTCL1
NAALADL2	MTCO2P12
NAB1	MTCP1
NAB2	MTDH
NABP1	MTDHB
NABP2	MTERF1
NACA	MTERF1A
NACA4P	MTERF2
NACAD	MTERF3
NACC1	MTERF4
NACC2	MTERFD1
NACHRA1	MTF
NACHRALPHA1	MTF1
NADK	MTF2
NADK2	MTFMT
NADSYN1	MTFP1
NAE1	MTFR1
NAF1	MTFR1L
NAGA	MTFR2
NAGK	MTG1
NAGLU	MTHFD1

NAGPA	MTHFD1L
NAGS	MTHFD2
NAIF1	MTHFD2L
NAIP	MTHFR
NAIP1	MTHFS
NAIP2	MTHFSD
NAIP5	MTIF2
NALCN	MTIF3
NALF1	MTK
NALF2	MTLN
NALT1	MTM1
NAMPT	MTMR1
NAMPT1	MTMR10
NANOG	MTMR11
NANOS1	MTMR12
NANOS2	MTMR14
NANOS3	MTMR2
NANP	MTMR3
NANS	MTMR4
NAP1L1	MTMR6
NAP1L2	MTMR7
NAP1L3	MTMR8
NAP1L4	MTMR9
NAP1L5	MTMR9LP
NAPA	MTND4P12
NAPAA	MTND4P24
NAPA-AS1	MTNR1A
NAPB	MTNR1B
NAPEPLD	MTO1
NAPG	MTOR
NAPRT	MTPAP
NAPSA	MTPN
NAPSB	MTR
NARF	MTRES1
NARG2	MTREX
NARS	MTRF1

NARS1	MTRF1L
NARS2	MTRFR
NASP	MTRNR2L1
NAT1	MTRNR2L12
NAT10	MTRNR2L2
NAT14	MTRNR2L3
NAT16	MTRR
NAT2	MTSS1
NAT5	MTSS2
NAT8	MTTP
NAT8B	MTURN
NAT8F1	MTUS1
NAT8F2	MTUS1-DT
NAT8F3	MTUS2
NAT8F5	MTX1
NAT8L	MTX2
NAT9	MTX3
NATD1	MUC1
NAV1	MUC12
NAV2	MUC13
NAV3	MUC15
NAXD	MUC16
NAXE	MUC17
NBAS	MUC19
NBDY	MUC2
NBEA	MUC20
NBEAL1	MUC20-OT1
NBEAL2	MUC21
NBEAP1	MUC3A
NBL1	MUC3B
NBN	MUC4
NBPF1	MUC5AC
NBPF10	MUC5B
NBPF12	MUC5B-AS1
NBPF14	MUC6
NBPF15	MUC7

NBPF20	MUCL1
NBPF3	MUCL2
NBPF4	MUCL3
NBPF8	MUG1
NBPF9	MUG2
NBR1	MUG-PS1
NBR2	MUL1
NCALD	MUL-1
NCAM1	MUP1
NCAM2	MUP14
NCAN	MUP21
NCANA	MUP3
NCAPD2	MUP4
NCAPD3	MUP5
NCAPG	MUP6
NCAPG2	MUP9
NCAPH	MUS81
NCAPH2	MUSK
NCBP1	MUSTN1
NCBP2	MUTYH
NCBP2AS2	MVB12A
NCBP3	MVB12B
NCCRP1	MVD
NCDN	MVK
NCEH1	MVP
NCF1	MX1
NCF1B	MX2
NCF1C	MXC
NCF2	MXD1
NCF4	MXD3
NCK1	MXD4
NCK2	MXI1
NCK2A	MXRA5
NCKAP1	MXRA7
NCKAP1L	MXRA8
NCKAP5	MYADM

NCKAP5L	MYADML
NCKIPSD	MYADML2
NCL	MYB
NCLN	MYBBP1A
NCMAP	MYBL1
NCOA1	MYBL2
NCOA2	MYBPC1
NCOA3	MYBPC2
NCOA4	MYBPC2A
NCOA5	MYBPC2B
NCOA6	MYBPC3
NCOA7	MYBPH
NCOR1	MYBPHA
NCOR1P1	MYBPHL
NCOR2	MYC
NCR1	MYCBP
NCR2	MYCBP2
NCR3	MYCBPAP
NCR3LG1	MYCL
NCRUPAR	MYCN
NCS1	MYCN.L
NCSTN	MYCNOS
ND1	MYCS
ND2	MYCT1
ND3	MYD88
ND4	MYDGF
ND4L	MYEF2
ND5	MYEOV
ND6	MYEOV2
NDC1	MYF5
NDC80	MYF6
NDE1	MYG1
NDEL1	MYH1
NDFIP1	MYH10
NDFIP2	MYH11
NDN	MYH13

NDNF	MYH14
NDNL2	MYH15
NDOR1	MYH16
NDP	MYH2
NDRG1	MYH3
NDRG1.L	MYH4
NDRG2	MYH6
NDRG3	MYH7
NDRG4	MYH7B
NDRG4.L	MYH8
NDST1	MYH9
NDST2	MYHZ1.1
NDST3	MYHZ2
NDST4	MYL1
NDUFA1	MYL10
NDUFA10	MYL11
NDUFA11	MYL12A
NDUFA12	MYL12B
NDUFA13	MYL2
NDUFA2	MYL3
NDUFA3	MYL4
NDUFA4	MYL5
NDUFA4L2	MYL6
NDUFA5	MYL6B
NDUFA6	MYL7
NDUFA6-DT	MYL9
NDUFA7	MYLIP
NDUFA8	MYLIPB
NDUFA9	MYLK
NDUFAB1	MYLK2
NDUFAF1	MYLK3
NDUFAF2	MYLK4
NDUFAF3	MYLPF
NDUFAF4	MYLPFA
NDUFAF6	MYLPFB
NDUFAF7	MYLZ3

NDUFAF8	MYMK
NDUFB1	MYNN
NDUFB10	MYO10
NDUFB11	MYO15A
NDUFB2	MYO15B
NDUFB2-AS1	MYO16
NDUFB3	MYO16-AS1
NDUFB4	MYO18A
NDUFB5	MYO18B
NDUFB6	MYO18B-AS1
NDUFB7	MYO19
NDUFB8	MYO1B
NDUFB9	MYO1C
NDUFC1	MYO1D
NDUFC2	MYO1E
NDUFC2-KCTD14	MYO1F
NDUFS1	MYO1G
NDUFS2	MYO3A
NDUFS3	MYO3B
NDUFS4	MYO5A
NDUFS5	MYO5B
NDUFS6	MYO5C
NDUFS7	MYO6
NDUFS8	MYO7A
NDUFV1	MYO7B
NDUFV2	MYO9A
NDUFV2-AS1	MYO9B
NDUFV3	MYOC
NEAT1	MYOCD
NEB	MYOD1
NEBL	MYOF
NEBL-AS1	MYOG
NECAB1	MYOM1
NECAB2	MYOM2
NECAB3	MYOM3
NECAP1	MYORG

NECAP2	MYOT
NECTIN1	MYOZ1
NECTIN2	MYOZ1B
NECTIN3	MYOZ2
NECTIN4	MYOZ3
NEDD1	MYPN
NEDD4	MYPOP
NEDD4A	MYRF
NEDD4L	MYRF-AS1
NEDD8	MYRFL
NEDD9	MYRIP
NEFH	MYSM1
NEFL	MYST2
NEFM	MYT1
NEGR1	MYT1L
NEIL1	MYZAP
NEIL2	MZB1
NEIL3	MZF1
NEK1	MZF1-AS1
NEK10	MZT1
NEK11	MZT2A
NEK2	MZT2B
NEK3	N4BP1
NEK4	N4BP2
NEK5	N4BP2L1
NEK6	N4BP2L2
NEK7	N4BP2L2-IT2
NEK8	N4BP3
NEK9	N6AMT1
NELFA	N6AMT2
NELFB	NAA10
NELFCD	NAA15
NELFE	NAA16
NELL1	NAA20
NELL2	NAA25
NEMF	NAA30

NEMP1	NAA35
NEMP2	NAA38
NENF	NAA40
NEO1	NAA50
NEPN	NAA60
NEPRO	NAA80
NES	NAAA
NET1	NAALAD2
NETO1	NAALADL1
NETO2	NAALADL2
NEU1	NAB1
NEU2	NAB2
NEU3	NABP1
NEU4	NABP2
NEURL1	NACA
NEURL1A	NACA4P
NEURL1B	NACAD
NEURL2	NACC1
NEURL3	NACC2
NEURL4	NACHRA1
NEUROD1	NACHRALPHA1
NEUROD2	NADK
NEUROD4	NADK2
NEUROD6	NADSYN1
NEUROG1	NAE1
NEUROG2	NAF1
NEUROG3	NAGA
NEXMIF	NAGK
NEXN	NAGLU
NF1	NAGPA
NF2	NAGS
NFAM1	NAIF1
NFASC	NAIP
NFAT5	NAIP1
NFATC1	NAIP2
NFATC2	NAIP5

NFATC2IP	NALCN
NFATC3	NALF1
NFATC4	NALF2
NFE2	NALT1
NFE2L1	NAMPT
NFE2L1A	NAMPT1
NFE2L2	NANOG
NFE2L2A	NANOS1
NFE2L2B	NANOS2
NFE2L3	NANOS3
NFE2L3P2	NANP
NFE4	NANS
NFIA	NAP1L1
NFIB	NAP1L2
NFIC	NAP1L3
NFIL3	NAP1L4
NFIL3-6	NAP1L5
NFIX	NAPA
NFKB1	NAPAA
NFKB2	NAPA-AS1
NFKBIA	NAPB
NFKBIAB	NAPEPLD
NFKBIB	NAPG
NFKBID	NAPRT
NFKBIE	NAPSA
NFKBIL1	NAPSB
NFKBIZ	NARF
NFRKB	NARG2
NFS1	NARS
NFU1	NARS1
NFX1	NARS2
NFXL1	NASP
NFYA	NAT1
NFYB	NAT10
NFYC	NAT14
NFYC-AS1	NAT16

NGB	NAT2
NGDN	NAT5
NGEF	NAT8
NGF	NAT8B
NGFG	NAT8F1
NGFR	NAT8F2
NGFR-AS1	NAT8F3
NGLY1	NAT8F5
NGP	NAT8L
NGRN	NAT9
NHEG1	NATD1
NHEJ1	NAV1
NHLH1	NAV2
NHLH2	NAV3
NHLRC1	NAXD
NHLRC2	NAXE
NHLRC3	NBAS
NHLRC4	NBAT1
NHP2	NBDY
NHS	NBEA
NHSL1	NBEAL1
NIBAN1	NBEAL2
NIBAN2	NBEAP1
NIBAN3	NBL1
NICN1	NBN
NID1	NBPF1
NID2	NBPF10
NIF.S	NBPF12
NIF3L1	NBPF14
NIFK	NBPF15
NIFK-AS1	NBPF20
NIM1K	NBPF3
NIN	NBPF4
NINAA	NBPF8
NINJ1	NBPF9
NINJ2	NBR1

NINL	NBR2
NIP7	NCALD
NIPA1	NCAM1
NIPA2	NCAM2
NIPAL1	NCAN
NIPAL2	NCANA
NIPAL3	NCAPD2
NIPAL4	NCAPD3
NIPBL	NCAPG
NIPSNAP1	NCAPG2
NIPSNAP2	NCAPH
NIPSNAP3A	NCAPH2
NIPSNAP3B	NCBP1
NISCH	NCBP2
NIT1	NCBP2AS2
NIT2	NCBP3
NITR3D	NCCRP1
NITR4A	NCDN
NK4	NCEH1
NKAIN1	NCF1
NKAIN2	NCF1B
NKAIN3	NCF1C
NKAIN4	NCF2
NKAP	NCF4
NKAPD1	NCK1
NKAPL	NCK2
NKAPP1	NCK2A
NKD1	NCKAP1
NKD2	NCKAP1L
NKG7	NCKAP5
NKIRAS1	NCKAP5L
NKIRAS2	NCKIPSD
NKPD1	NCL
NKRF	NCLN
NKTR	NCMAP
NKX1-1	NCOA1

NKX1-2	NCOA2
NKX2.1	NCOA3
NKX2.2A	NCOA4
NKX2.2B	NCOA5
NKX2.5	NCOA6
NKX2.9	NCOA7
NKX2-1	NCOR1
NKX2-2	NCOR1P1
NKX2-3	NCOR2
NKX2-4	NCR1
NKX2-5	NCR2
NKX2-8	NCR3
NKX2-9	NCR3LG1
NKX3-1	NCRUPAR
NKX3-2	NCS1
NKX6-1	NCSTN
NKX6-2	ND1
NKX6-3	ND2
NLE1	ND3
NLF2	ND4
NLGN1	ND4L
NLGN2	ND5
NLGN3	ND6
NLGN4X	NDC1
NLGN4Y	NDC80
NLK	NDE1
NLN	NDEL1
NLRC3	NDFIP1
NLRC4	NDFIP2
NLRC5	NDN
NLRP1	NDNF
NLRP10	NDNL2
NLRP12	NDOR1
NLRP13	NDP
NLRP14	NDRG1
NLRP1A	NDRG1.L

NLRP1B	NDRG2
NLRP1C-PS	NDRG3
NLRP2	NDRG4
NLRP3	NDRG4.L
NLRP4F	NDST1
NLRP5	NDST2
NLRP6	NDST3
NLRP7	NDST4
NLRP9	NDUFA1
NLRP9C	NDUFA10
NLRX1	NDUFA11
NMA	NDUFA12
NMB	NDUFA13
NMBR	NDUFA2
NMD3	NDUFA3
NMDA1	NDUFA4
NME1	NDUFA4L2
NME1-NME2	NDUFA5
NME2	NDUFA6
NME2B.2	NDUFA6-DT
NME3	NDUFA7
NME4	NDUFA8
NME5	NDUFA9
NME6	NDUFAB1
NME7	NDUFAF1
NME8	NDUFAF2
NME9	NDUFAF3
NMES1	NDUFAF4
NMI	NDUFAF6
NMNAT1	NDUFAF7
NMNAT2	NDUFAF8
NMNAT3	NDUFB1
NMRAL1	NDUFB10
NMRAL2P	NDUFB11
NMRK1	NDUFB2
NMRK2	NDUFB2-AS1

NMS	NDUFB3
NMT1	NDUFB4
NMT2	NDUFB5
NMU	NDUFB6
NMUR1	NDUFB7
NMUR2	NDUFB8
NNAT	NDUFB9
NNMT	NDUFC1
NNT	NDUFC2
NNT-AS1	NDUFC2-KCTD14
NOA1	NDUFS1
NOB1	NDUFS2
NOBOX	NDUFS3
NOC2L	NDUFS4
NOC3L	NDUFS5
NOC4L	NDUFS6
NOCT	NDUFS7
NOD1	NDUFS8
NOD2	NDUFV1
NODAL	NDUFV2
NOG	NDUFV2-AS1
NOL1	NDUFV3
NOL10	NEAT1
NOL11	NEB
NOL12	NEBL
NOL3	NEBL-AS1
NOL4	NECAB1
NOL4L	NECAB2
NOL5	NECAB3
NOL6	NECAP1
NOL7	NECAP2
NOL8	NECTIN1
NOL9	NECTIN2
NOLC1	NECTIN3
NOM1	NECTIN4
NOMO1	NEDD1

NOMO2	NEDD4
NOMO3	NEDD4A
NONO	NEDD4L
NOP10	NEDD8
NOP14	NEDD9
NOP14-AS1	NEFH
NOP16	NEFL
NOP2	NEFM
NOP53	NEGR1
NOP56	NEIL1
NOP56P2	NEIL2
NOP58	NEIL3
NOP9	NEK1
NOPCHAP1	NEK10
NORAD	NEK11
NOS	NEK2
NOS1	NEK3
NOS1AP	NEK4
NOS2	NEK5
NOS2A	NEK6
NOS2B	NEK7
NOS3	NEK8
NOSIP	NEK9
NOSTRIN	NELFA
NOT2	NELFB
NOTCH1	NELFCD
NOTCH2	NELFE
NOTCH2NLA	NELL1
NOTCH3	NELL2
NOTCH4	NEMF
NOTO	NEMP1
NOTUM	NEMP2
NOTUM1B	NENF
NOVA1	NEO1
NOVA2	NEPN
NOX1	NEPRO

NOX3	NES
NOX4	NET1
NOX5	NETO1
NOXA1	NETO2
NOXO1	NEU1
NOXRED1	NEU2
NP	NEU3
NPAP1	NEU4
NPAS1	NEURL1
NPAS2	NEURL1A
NPAS3	NEURL1B
NPAS4	NEURL2
NPAT	NEURL3
NPB	NEURL4
NPBWR1	NEUROD1
NPBWR2	NEUROD2
NPC1	NEUROD4
NPC1L1	NEUROD6
NPC2	NEUROG1
NPDC1	NEUROG2
NPEPL1	NEUROG3
NPEPPS	NEXMIF
NPFF	NEXN
NPFFR1	NEXN-AS1
NPFFR2	NF1
NPFFR2A	NF2
NPHP1	NFAM1
NPHP3	NFASC
NPHP3-AS1	NFAT5
NPHP4	NFATC1
NPHS1	NFATC2
NPHS1OS	NFATC2IP
NPHS2	NFATC3
NPIPA1	NFATC4
NPIPA5	NFE2
NPIPB11	NFE2L1

NPIPB6	NFE2L1A
NPL	NFE2L2
NPLOC4	NFE2L2A
NPM1	NFE2L2B
NPM2	NFE2L3
NPM3	NFE2L3P2
NPNT	NFE4
NPPA	NFIA
NPPB	NFIB
NPPC	NFIC
NPR1	NFIL3
NPR2	NFIL3-6
NPR3	NFIX
NPRL2	NFKB1
NPRL3	NFKB2
NPSN	NFKBIA
NPSR1	NFKBIAB
NPTN	NFKBIB
NPTX1	NFKBID
NPTX2	NFKBIE
NPTXR	NFKBIL1
NPVF	NFKBIZ
NPW	NFRKB
NPY	NFS1
NPY1R	NFU1
NPY2R	NFX1
NPY4R	NFXL1
NPY5R	NFYA
NPY6R	NFYB
NQO1	NFYC
NQO2	NFYC-AS1
NR0B1	NGB
NR0B2	NGDN
NR1D1	NGEF
NR1D2	NGF
NR1D2A	NGFG

NR1H2	NGFR
NR1H3	NGFR-AS1
NR1H4	NGLY1
NR1H5	NGP
NR1I2	NGRN
NR1I3	NHEG1
NR2C1	NHEJ1
NR2C2	NHLH1
NR2C2AP	NHLH2
NR2E1	NHLRC1
NR2E3	NHLRC2
NR2F1	NHLRC3
NR2F1-AS1	NHLRC4
NR2F2	NHP2
NR2F2-AS1	NHS
NR2F5	NHSL1
NR2F6	NIBAN1
NR3C1	NIBAN2
NR3C2	NIBAN3
NR4A1	NICN1
NR4A2	NID1
NR4A3	NID2
NR5A1	NIF.S
NR5A2	NIF3L1
NR5A2.L	NIFK
NR6A1	NIFK-AS1
NRAD1	NIM1K
NRADD	NIN
NRADDP	NINAA
NRAP	NINJ1
NRARP	NINJ2
NRARPB	NINL
NRAS	NIP7
NRAV	NIPA1
NRBF2	NIPA2
NRBP1	NIPAL1

NRBP2	NIPAL2
NRCAM	NIPAL3
NRD1	NIPAL4
NRDC	NIPBL
NRDE2	NIPSNAP1
NREP	NIPSNAP2
NRF1	NIPSNAP3A
NRG1	NIPSNAP3B
NRG2	NISCH
NRG3	NIT1
NRG4	NIT2
NRGN	NITR3D
NRIP1	NITR4A
NRIP2	NK4
NRIP3	NKAIN1
NRK	NKAIN2
NRL	NKAIN3
NRM	NKAIN4
NRN1	NKAP
NRN1L	NKAPD1
NRP	NKAPL
NRP1	NKAPP1
NRP2	NKD1
NRP2B	NKD2
NRROS	NKG7
NRSN2	NKILA
NRTN	NKIRAS1
NRXN1	NKIRAS2
NRXN2	NKPD1
NRXN3	NKRF
NSD1	NKTR
NSD2	NKX1-1
NSD3	NKX1-2
NSDHL	NKX2.1
NSF	NKX2.2A
NSFB	NKX2.2B

NSFL1C	NKX2.5
NSG1	NKX2.9
NSG2	NKX2-1
NSL1	NKX2-2
NSMAF	NKX2-3
NSMCE1	NKX2-4
NSMCE1-DT	NKX2-5
NSMCE2	NKX2-8
NSMCE3	NKX2-9
NSMCE4A	NKX3-1
NSMF	NKX3-2
NSPB-8	NKX6-1
NSPH-3.1	NKX6-2
NSPH-3.2	NKX6-3
NSRP1	NLE1
NSUN2	NLF2
NSUN3	NLGN1
NSUN4	NLGN2
NSUN5	NLGN3
NSUN5P1	NLGN4X
NSUN5P2	NLGN4Y
NSUN6	NLK
NSUN7	NLN
NT5C	NLRC3
NT5C1A	NLRC4
NT5C1B	NLRC5
NT5C2	NLRP1
NT5C2A	NLRP10
NT5C3	NLRP12
NT5C3A	NLRP13
NT5C3B	NLRP14
NT5DC1	NLRP1A
NT5DC2	NLRP1B
NT5DC3	NLRP1C-PS
NT5DC4	NLRP2
NT5E	NLRP3

NT5M	NLRP4F
NTAN1	NLRP5
NTAQ1	NLRP6
NTF3	NLRP7
NTF4	NLRP9
NTHL1	NLRP9C
NTM	NLRX1
NTMT1	NM
NTMT2	NMA
NTN1	NMB
NTN3	NMBR
NTN4	NMD3
NTN5	NMDA1
NTNG1	NME1
NTNG2	NME1-NME2
NTPCR	NME2
NTRK1	NME2B.2
NTRK2	NME3
NTRK3	NME4
NTS	NME5
NTSR1	NME6
NTSR2	NME7
NUAK1	NME8
NUAK1A	NME9
NUAK2	NMES1
NUB1	NMI
NUBP1	NMNAT1
NUBP2	NMNAT2
NUBPL	NMNAT3
NUCB1	NMRAL1
NUCB2	NMRAL2P
NUCKS1	NMRK1
NUCKS1A	NMRK2
NUDC	NMS
NUDCD1	NMT1
NUDCD2	NMT2

NUDCD3	NMU
NUDT1	NMUR1
NUDT10	NMUR2
NUDT11	NNAT
NUDT12	NNMT
NUDT13	NNT
NUDT14	NNT-AS1
NUDT15	NOA1
NUDT16	NOB1
NUDT16-DT	NOBOX
NUDT16L1	NOC2L
NUDT16L2P	NOC3L
NUDT17	NOC4L
NUDT18	NOCT
NUDT19	NOD1
NUDT2	NOD2
NUDT21	NODAL
NUDT22	NOG
NUDT3	NOL1
NUDT3B	NOL10
NUDT4	NOL11
NUDT4B	NOL12
NUDT5	NOL3
NUDT6	NOL4
NUDT7	NOL4L
NUDT8	NOL5
NUDT9	NOL6
NUF2	NOL7
NUFIP1	NOL8
NUFIP2	NOL9
NUMA1	NOLC1
NUMB	NOM1
NUMBL	NOMO1
NUP107	NOMO2
NUP133	NOMO3
NUP153	NONO

NUP155	NOP10
NUP160	NOP14
NUP188	NOP14-AS1
NUP205	NOP16
NUP210	NOP2
NUP214	NOP53
NUP35	NOP56
NUP37	NOP56P2
NUP43	NOP58
NUP50	NOP9
NUP54	NOPCHAP1
NUP58	NORAD
NUP62	NOS
NUP62CL	NOS1
NUP85	NOS1AP
NUP88	NOS2
NUP88.S	NOS2A
NUP93	NOS2B
NUP98	NOS3
NUPL1	NOSIP
NUPL2	NOSTRIN
NUPR1	NOT2
NUPR1.L	NOTCH1
NUPR1L	NOTCH2
NUPR2	NOTCH2NLA
NURF-1	NOTCH3
NUS1	NOTCH4
NUS1P3	NOTO
NUSAP1	NOTUM
NUTF2	NOTUM1B
NUTM1	NOVA1
NUTM2A	NOVA2
NUTM2A-AS1	NOX1
NUTM2B	NOX3
NUTM2B-AS1	NOX4
NUTM2D	NOX5

NUTM2E	NOXA1
NUTM2F	NOXO1
NUTM2G	NOXRED1
NVL	NP
NWD1	NPAP1
NWD2	NPAS1
NXF1	NPAS2
NXF2	NPAS3
NXF2B	NPAS4
NXF7	NPAT
NXN	NPB
NXNL2	NPBWR1
NXPE2	NPBWR2
NXPE3	NPC1
NXPE4	NPC1L1
NXPH1	NPC2
NXPH2	NPDC1
NXPH3	NPEPL1
NXPH4	NPEPPS
NXT1	NPFF
NXT2	NPFFR1
NYAP1	NPFFR2
NYAP2	NPFFR2A
NYNRIN	NPHP1
NYX	NPHP3
OAF	NPHP3-AS1
OARD1	NPHP4
OAS1	NPHS1
OAS1A	NPHS1OS
OAS1G	NPHS2
OAS1H	NPIPA1
OAS1I	NPIPA5
OAS2	NPIPB11
OAS3	NPIPB6
OASL	NPL
OASL1	NPLOC4

OASL2	NPM1
OAT	NPM2
OAZ1	NPM3
OAZ2	NPNT
OAZ2B	NPPA
OAZ3	NPPB
OBI1	NPPC
OBOX3	NPR1
OBOX5	NPR2
OBP1A	NPR3
OBP1F	NPRL2
OBP2A	NPRL3
OBP2B	NPSN
OBP3	NPSR1
OBP99A	NPTN
OBSCN	NPTN-IT1
OBSL1	NPTX1
OCA2	NPTX2
OCEL1	NPTXR
OCIAD1	NPVF
OCIAD2	NPW
OCLN	NPY
OCLNA	NPY1R
OCM	NPY2R
OCM2	NPY4R
OCM4.3.L	NPY5R
OCRL	NPY6R
OCSTAMP	NQO1
ODAD1	NQO2
ODAD2	NR0B1
ODAD3	NR0B2
ODAD4	NR1D1
ODAPH	NR1D2
ODC1	NR1D2A
ODF1	NR1H2
ODF2	NR1H3

ODF2L	NR1H4
ODF3	NR1H5
ODF3B	NR1I2
ODF3L1	NR1I3
ODF3L2	NR2C1
ODF4	NR2C2
ODR4	NR2C2AP
OFCC1	NR2E1
OFD1	NR2E3
OGA	NR2F1
OGDH	NR2F1-AS1
OGDHL	NR2F2
OGFOD1	NR2F2-AS1
OGFOD2	NR2F5
OGFOD3	NR2F6
OGFR	NR3C1
OGFRL1	NR3C2
OGG1	NR4A1
OGN	NR4A2
OGT	NR4A3
OIP5	NR5A1
OIP5-AS1	NR5A2
OIT3	NR5A2.L
OLA1	NR6A1
OLAH	NRAD1
OLD-1	NRADD
OLFM1	NRADDP
OLFM2	NRAP
OLFM3	NRARP
OLFM4	NRARPB
OLFML1	NRAS
OLFML2A	NRAV
OLFML2B	NRBF2
OLFML3	NRBP1
OLFR100	NRBP2
OLFR197	NRCAM

OLFR209	NRD1
OLFR766-PS1	NRDC
OLFR908	NRDE2
OLIG1	NREP
OLIG2	NRF1
OLIG3	NRG1
OLMALINC	NRG2
OLR1	NRG3
OLR1108	NRG4
OLR1280	NRGN
OLR63	NRIP1
OLR737	NRIP2
OLR961-PS	NRIP3
OMA1	NRK
OMD	NRL
OMG	NRM
OMP	NRN1
ONECUT1	NRN1L
ONECUT2	NRP
ONECUT3	NRP1
OOG3	NRP2
OOG4	NRP2B
OOSP2	NRROS
OPA1	NRSN2
OPA3	NRTN
OPALIN	NRXN1
OPCML	NRXN2
OPHN1	NRXN3
OPLAH	NSD1
OPN1LW	NSD2
OPN1MW	NSD3
OPN1MW2	NSDHL
OPN1SW	NSF
OPN1SW2	NSFB
OPN3	NSFL1C
OPN4	NSG1

OPN5	NSG2
OPRD1	NSL1
OPRK1	NSMAF
OPRL1	NSMCE1
OPRM1	NSMCE1-DT
OPTC	NSMCE2
OPTN	NSMCE3
OR10A4	NSMCE4A
OR10A5	NSMF
OR10A6	NSPB-8
OR10AA1	NSPH-3.1
OR10AD1C	NSPH-3.2
OR10AG57	NSRP1
OR10AH1-PS1	NSUN2
OR10AK11	NSUN3
OR10AL5	NSUN4
OR10B1P	NSUN5
OR10C1	NSUN5P1
OR10D1	NSUN5P2
OR10D4B	NSUN6
OR10G1B	NSUN7
OR10G2	NT5C
OR10G7	NT5C1A
OR10G9	NT5C1B
OR10H2	NT5C2
OR10H5	NT5C2A
OR10J3	NT5C3
OR10K1	NT5C3A
OR10P21	NT5C3B
OR10Q1	NT5DC1
OR10V5	NT5DC2
OR10X1	NT5DC3
OR10Z1	NT5DC4
OR11G2	NT5E
OR11H4	NT5M
OR11L3	NTAN1

OR12D2	NTAQ1
OR12E9	NTF3
OR12J4	NTF4
OR12K8	NTHL1
OR13A1	NTM
OR13A27	NTMT1
OR13A28	NTMT2
OR13C4	NTN1
OR13C7	NTN3
OR13C9	NTN4
OR13D1	NTN5
OR13E8	NTNG1
OR13H1	NTNG2
OR13J1	NTPCR
OR13P3	NTRK1
OR14C39	NTRK2
OR14C45	NTRK3
OR14J1	NTS
OR14J3	NTSR1
OR14J5	NTSR2
OR14R1-PS1	NUAK1
OR1A2	NUAK1A
OR1AD6	NUAK2
OR1C1	NUB1
OR1D2	NUBP1
OR1D4	NUBP2
OR1E22	NUBPL
OR1E3	NUCB1
OR1E33	NUCB2
OR1F1	NUCKS1
OR1F12P	NUCKS1A
OR1F19	NUDC
OR1F2P	NUDCD1
OR1I1	NUDCD2
OR1J19	NUDCD3
OR1K1	NUDT1

OR1L4	NUDT10
OR1L8	NUDT11
OR1M1	NUDT12
OR1O11	NUDT13
OR2A1	NUDT14
OR2A2	NUDT15
OR2A4	NUDT16
OR2A42	NUDT16-DT
OR2A5	NUDT16L1
OR2A52	NUDT16L2P
OR2A57	NUDT17
OR2A9P	NUDT18
OR2AG1	NUDT19
OR2AJ6	NUDT2
OR2AV9	NUDT21
OR2B11	NUDT22
OR2B28	NUDT3
OR2C1	NUDT3B
OR2D36	NUDT4
OR2F1	NUDT4B
OR2F2	NUDT5
OR2G6	NUDT6
OR2H1	NUDT7
OR2H15	NUDT8
OR2H2	NUDT9
OR2K2	NUF2
OR2L13	NUFIP1
OR2M1P	NUFIP2
OR2M2	NUMA1
OR2M3	NUMB
OR2M5	NUMBL
OR2N1	NUP107
OR2N1D	NUP133
OR2S2	NUP153
OR2T10	NUP155
OR2T11	NUP160

OR2T12	NUP188
OR2T2	NUP205
OR2T3	NUP210
OR2T33	NUP214
OR2T34	NUP35
OR2T35	NUP37
OR2T4	NUP43
OR2T44	NUP50
OR2T6	NUP54
OR2V1	NUP58
OR2W4	NUP62
OR2Y1	NUP62CL
OR2Y11	NUP85
OR2Y15	NUP88
OR2Y16	NUP88.S
OR2Y1C	NUP93
OR2Y1F	NUP98
OR2Y6	NUPL1
OR2Z1	NUPL2
OR2Z2	NUPR1
OR3A10	NUPR1.L
OR3A1B	NUPR1L
OR3A4P	NUPR2
OR4A15	NURF-1
OR4A5	NUS1
OR4A71	NUS1P3
OR4A72	NUSAP1
OR4A75	NUTF2
OR4A81	NUTM1
OR4B1D	NUTM2A
OR4C1	NUTM2A-AS1
OR4C10	NUTM2B
OR4C115	NUTM2B-AS1
OR4C12	NUTM2D
OR4C15	NUTM2E
OR4C3	NUTM2F

OR4C35	NUTM2G
OR4C3D	NVL
OR4D11	NWD1
OR4D6	NWD2
OR4E5	NXF1
OR4F15	NXF2
OR4F53	NXF2B
OR4F57	NXF7
OR4F6	NXN
OR4F60	NXNL2
OR4F61	NXPE2
OR4K15	NXPE3
OR4K17	NXPE4
OR4K49	NXPH1
OR4M1	NXPH2
OR4M2	NXPH3
OR4N4	NXPH4
OR4P22	NXT1
OR4Q3	NXT2
OR4S1	NYAP1
OR4X1	NYAP2
OR4X13	NYNRIN
OR4X2	NYX
OR51A7	OAF
OR51AH3	OARD1
OR51B2	OAS1
OR51B4	OAS1A
OR51D1	OAS1G
OR51E1	OAS1H
OR51E2	OAS1I
OR51F2	OAS2
OR51G1	OAS3
OR51H1	OASL
OR51H5	OASL1
OR51I1	OASL2
OR51L14	OAT

OR51Q1	OAZ1
OR51Q1C	OAZ2
OR51S1	OAZ2B
OR51V1	OAZ3
OR51V15-PS1	OBI1
OR51V8	OBOX3
OR52A1	OBOX5
OR52AE9	OBP1A
OR52B3	OBP1F
OR52E2	OBP2A
OR52E4	OBP2B
OR52E7	OBP3
OR52E8	OBP99A
OR52E8B	OBSCN
OR52H1	OBSL1
OR52I2	OCA2
OR52J3	OCEL1
OR52K2	OCIAD1
OR52K3P	OCIAD2
OR52L1	OCLN
OR52N1	OCLNA
OR52N20	OCM
OR52P1	OCM2
OR52S19	OCM4.3.L
OR52S1B	OCRL
OR52W1	OCSTAMP
OR56A1	ODAD1
OR56A3	ODAD2
OR56A5	ODAD3
OR56B2	ODAD4
OR5AC23	ODAPH
OR5AK2	ODC1
OR5AK22	ODF1
OR5AK24	ODF2
OR5AQ6	ODF2L
OR5AR1	ODF3

OR5AS1	ODF3B
OR5AU1	ODF3L1
OR5B101	ODF3L2
OR5B108	ODF4
OR5B109	ODR4
OR5B112	OFCC1
OR5B117	OFD1
OR5B12	OGA
OR5B121	OGDH
OR5B122	OGDHL
OR5B2	OGFOD1
OR5B97	OGFOD2
OR5C1	OGFOD3
OR5D14	OGFR
OR5D16	OGFRL1
OR5D18	OGFRP1
OR5D41	OGG1
OR5E1	OGN
OR5E1P	OGT
OR5H1	OIP5
OR5H14	OIP5-AS1
OR5H19	OIT3
OR5H2	OLA1
OR5H6	OLAH
OR5I1	OLD-1
OR5K1	OLFM1
OR5K2	OLFM2
OR5L1	OLFM3
OR5L13	OLFM4
OR5M1	OLFML1
OR5M10	OLFML2A
OR5M10B	OLFML2B
OR5M11	OLFML3
OR5M5	OLFR100
OR5P1	OLFR197
OR5P3	OLFR209

OR5P67	OLFR766-PS1
OR5P79	OLFR908
OR5P80	OLIG1
OR5T15	OLIG2
OR5T2	OLIG3
OR5T9	OLMALINC
OR5V1	OLR1
OR5W13	OLR1108
OR5W15	OLR1280
OR64B4	OLR63
OR6A2	OLR737
OR6B2	OLR961-PS
OR6B3	OMA1
OR6C2	OMD
OR6C206	OMG
OR6C209	OMP
OR6C212	ONECUT1
OR6C214	ONECUT2
OR6C216	ONECUT3
OR6C33	OOG3
OR6C4	OOG4
OR6C5	OOSP2
OR6C66	OPA1
OR6C68	OPA3
OR6C74	OPALIN
OR6F2	OPCML
OR6J1	OPHN1
OR6K2	OPLAH
OR6K3	OPN1LW
OR6N1	OPN1MW
OR6N2	OPN1MW2
OR6P1	OPN1SW
OR6Q1	OPN1SW2
OR6T1	OPN3
OR7D10	OPN4
OR7D2	OPN5

OR7E125P	OPRD1
OR7E12P	OPRK1
OR7E156P	OPRL1
OR7E169	OPRM1
OR7E170	OPTC
OR7E19P	OPTN
OR7E47P	OR10A4
OR7E91P	OR10A5
OR7G1	OR10A6
OR7G17	OR10AA1
OR7G18	OR10AD1C
OR7G22	OR10AG57
OR7G30	OR10AH1-PS1
OR7G35	OR10AK11
OR7H8	OR10AL5
OR8A1B	OR10B1P
OR8B12	OR10C1
OR8B1C	OR10D1
OR8B1D	OR10D4B
OR8B3	OR10G1B
OR8B4	OR10G2
OR8B41	OR10G7
OR8B54	OR10G9
OR8B8	OR10H2
OR8C20	OR10H5
OR8C8	OR10J3
OR8D6	OR10K1
OR8G18	OR10P21
OR8G2	OR10Q1
OR8G29-PS1	OR10V5
OR8G5	OR10X1
OR8G50	OR10Z1
OR8H1	OR11G2
OR8I2	OR11H4
OR8J3	OR11L3
OR8K18	OR12D2

OR8K25	OR12E9
OR9A2	OR12J4
OR9A4	OR12K8
OR9G1	OR13A1
OR9G3	OR13A27
OR9G4	OR13A28
OR9G9	OR13C4
OR9I1	OR13C7
OR9I14	OR13C9
OR9K2	OR13D1
OR9M1B	OR13E8
OR9Q1	OR13H1
OR9Q2	OR13J1
OR9S18	OR13P3
ORAI1	OR14C39
ORAI1B	OR14C45
ORAI2	OR14J1
ORAI3	OR14J3
ORC1	OR14J5
ORC2	OR14R1-PS1
ORC3	OR1A2
ORC4	OR1AD6
ORC5	OR1C1
ORC6	OR1D2
ORM1	OR1D4
ORM2	OR1E22
ORM3	OR1E3
ORMDL1	OR1E33
ORMDL2	OR1F1
ORMDL3	OR1F12P
OS9	OR1F19
OSBP	OR1F2P
OSBP2	OR1I1
OSBPL10	OR1J19
OSBPL11	OR1K1
OSBPL1A	OR1L4

OSBPL2	OR1L8
OSBPL3	OR1M1
OSBPL3A	OR1O11
OSBPL5	OR2A1
OSBPL6	OR2A2
OSBPL7	OR2A4
OSBPL8	OR2A42
OSBPL9	OR2A5
OSCAR	OR2A52
OSCP1	OR2A57
OSER1	OR2A9P
OSER1-DT	OR2AG1
OSGEP	OR2AJ6
OSGEPL1	OR2AV9
OSGIN1	OR2B11
OSGIN2	OR2B28
OSGN1	OR2C1
OSM	OR2D36
OSMR	OR2F1
OSR1	OR2F2
OSR2	OR2G6
OST4	OR2H1
OSTC	OR2H15
OSTCP1	OR2H2
OSTF1	OR2J3
OSTM1	OR2K2
OSTN	OR2L13
OTC	OR2M1P
OTOF	OR2M2
OTOG	OR2M3
OTOGL	OR2M5
OTOL1	OR2N1
OTOMP	OR2N1D
OTOP1	OR2S2
OTOP2	OR2T10
OTOP3	OR2T11

OTOS	OR2T12
OTP	OR2T2
OTPA	OR2T3
OTPB	OR2T33
OTUB1	OR2T34
OTUB2	OR2T35
OTUD1	OR2T4
OTUD3	OR2T44
OTUD4	OR2T6
OTUD5	OR2V1
OTUD6A	OR2W4
OTUD6B	OR2Y1
OTUD6B-AS1	OR2Y11
OTUD7A	OR2Y15
OTUD7B	OR2Y16
OTULIN	OR2Y1C
OTULINL	OR2Y1F
OTX1	OR2Y6
OTX2	OR2Z1
OTX2-AS1	OR2Z2
OVAL	OR3A10
OVCA2	OR3A1B
OVCH2	OR3A4P
OVGP1	OR4A15
OVOL1	OR4A5
OVOL2	OR4A71
OVOS2	OR4A72
OXA1L	OR4A75
OXCT1	OR4A81
OXCT2	OR4B1D
OXCT2A	OR4C1
OXER1	OR4C10
OXGR1	OR4C115
OXI-1	OR4C12
OXLD1	OR4C15
OXNAD1	OR4C3

OXR1	OR4C35
OXSM	OR4C3D
OXSR1	OR4D11
OXT	OR4D6
OXTR	OR4E5
P24B	OR4F15
P2RX1	OR4F53
P2RX2	OR4F57
P2RX3	OR4F6
P2RX4	OR4F60
P2RX5	OR4F61
P2RX6	OR4K15
P2RX6P	OR4K17
P2RX7	OR4K49
P2RY1	OR4M1
P2RY10	OR4M2
P2RY11	OR4N4
P2RY12	OR4P22
P2RY13	OR4Q3
P2RY14	OR4S1
P2RY2	OR4X1
P2RY4	OR4X13
P2RY5	OR4X2
P2RY6	OR51A7
P2RY8	OR51AH3
P3H1	OR51B2
P3H2	OR51B4
P3H3	OR51D1
P3H4	OR51E1
P4HA1	OR51E2
P4HA2	OR51F2
P4HA3	OR51G1
P4HB	OR51H1
P4HTM	OR51H5
PA2G4	OR51I1
PA2G4P4	OR51L14

PAAF1	OR51Q1
PABIR1	OR51Q1C
PABIR2	OR51S1
PABIR3	OR51V1
PABP2	OR51V15-PS1
PABPC1	OR51V8
PABPC1.S	OR52A1
PABPC1B	OR52AE9
PABPC1L	OR52B3
PABPC1L2A	OR52E2
PABPC1L2B	OR52E4
PABPC3	OR52E7
PABPC4	OR52E8
PABPC4L	OR52E8B
PABPC5	OR52H1
PABPN1	OR52I2
PABPN1L	OR52J3
PACC1	OR52K2
PACRG	OR52K3P
PACRG-AS1	OR52L1
PACRG-AS3	OR52N1
PACRGL	OR52N20
PACS1	OR52P1
PACS2	OR52S19
PACSIN1	OR52S1B
PACSIN2	OR52W1
PACSIN3	OR56A1
PADI1	OR56A3
PADI2	OR56A5
PADI3	OR56B2
PADI4	OR5AC23
PADI6	OR5AK2
PAEP	OR5AK22
PAF1	OR5AK24
PAFAH1B1	OR5AQ6
PAFAH1B1A	OR5AR1

PAFAH1B1B	OR5AS1
PAFAH1B2	OR5AU1
PAFAH1B3	OR5B101
PAFAH2	OR5B108
PAG1	OR5B109
PAGE1	OR5B112
PAGE2	OR5B117
PAGE2B	OR5B12
PAGE4	OR5B121
PAGE5	OR5B122
PAGR1	OR5B2
PAH	OR5B97
PAICS	OR5C1
PAIP1	OR5D14
PAIP2	OR5D16
PAIP2B	OR5D18
PAK1	OR5D41
PAK1IP1	OR5E1
PAK2	OR5E1P
PAK3	OR5H1
PAK4	OR5H14
PAK5	OR5H19
PAK6	OR5H2
PAK6-AS1	OR5H6
PALB2	OR5I1
PALD1	OR5K1
PALLD	OR5K2
PALM	OR5L1
PALM2AKAP2	OR5L13
PALM3	OR5M1
PALMD	OR5M10
PALS1	OR5M10B
PALS2	OR5M11
PAM	OR5M5
PAM16	OR5P1
PAMR1	OR5P3

PAN2	OR5P67
PAN3	OR5P79
PAN3-AS1	OR5P80
PANK1	OR5T15
PANK2	OR5T2
PANK3	OR5T9
PANK4	OR5V1
PANX1	OR5W13
PANX2	OR5W15
PANX3	OR64B4
PAOX	OR6A2
PAPLN	OR6B2
PAPOLA	OR6B3
PAPOLG	OR6C2
PAPPA	OR6C206
PAPPA2	OR6C209
PAPSS1	OR6C212
PAPSS2	OR6C214
PAQR3	OR6C216
PAQR4	OR6C33
PAQR5	OR6C4
PAQR6	OR6C5
PAQR7	OR6C66
PAQR8	OR6C68
PAQR9	OR6C74
PARD3	OR6F2
PARD3B	OR6J1
PARD6A	OR6K2
PARD6B	OR6K3
PARD6G	OR6N1
PARD6G-AS1	OR6N2
PARG	OR6P1
PARK7	OR6Q1
PARL	OR6T1
PARM1	OR7D10
PARN	OR7D2

PARP	OR7E125P
PARP1	OR7E12P
PARP10	OR7E156P
PARP11	OR7E169
PARP12	OR7E170
PARP14	OR7E19P
PARP15	OR7E47P
PARP16	OR7E91P
PARP2	OR7G1
PARP3	OR7G17
PARP4	OR7G18
PARP6	OR7G22
PARP8	OR7G30
PARP9	OR7G35
PARPBP	OR7H8
PARS2	OR8A1B
PART1	OR8B12
PARTICL	OR8B1C
PARVA	OR8B1D
PARVB	OR8B3
PARVG	OR8B4
PASD1	OR8B41
PASK	OR8B54
PATE1	OR8B8
PATJ	OR8C20
PATL1	OR8C8
PATL2	OR8D6
PATZ1	OR8G18
PAWR	OR8G2
PAX1	OR8G29-PS1
PAX2	OR8G5
PAX3	OR8G50
PAX4	OR8H1
PAX5	OR8I2
PAX6	OR8J3
PAX6B	OR8K18

PAX6OS1	OR8K25
PAX7	OR9A2
PAX7A	OR9A4
PAX8	OR9G1
PAX8-AS1	OR9G3
PAX9	OR9G4
PAXBP1	OR9G9
PAXBP1-AS1	OR9I1
PAXIP1	OR9I14
PAXIP1-DT	OR9K2
PAXX	OR9M1B
PBDC1	OR9Q1
PBK	OR9Q2
PBLD	OR9S18
PBLD1	ORAI1
PBRM1	ORAI1B
PBX1	ORAI2
PBX1A	ORAI3
PBX2	ORC1
PBX3	ORC2
PBX4	ORC3
PBXIP1	ORC4
PC	ORC5
PCAT19	ORC6
PCAT4	ORM1
PCAT6	ORM2
PCBD1	ORM3
PCBD2	ORMDL1
PCBP1	ORMDL2
PCBP1-AS1	ORMDL3
PCBP2	OS9
PCBP3	OSBP
PCBP4	OSBP2
PCCA	OSBPL10
PCCB	OSBPL11
PCDH1	OSBPL1A

PCDH10	OSBPL2
PCDH11X	OSBPL3
PCDH11Y	OSBPL3A
PCDH12	OSBPL5
PCDH15	OSBPL6
PCDH17	OSBPL7
PCDH18	OSBPL8
PCDH19	OSBPL9
PCDH1GB2	OSCAR
PCDH20	OSCP1
PCDH2AC	OSER1
PCDH7	OSER1-DT
PCDH8	OSGEP
PCDH9	OSGEPL1
PCDHA10	OSGIN1
PCDHA11	OSGIN2
PCDHA12	OSGN1
PCDHA13	OSM
PCDHA2	OSMR
PCDHA4	OSR1
PCDHA5	OSR2
PCDHA8	OST4
PCDHA9	OSTC
PCDHAC1	OSTCP1
PCDHAC2	OSTF1
PCDHB1	OSTM1
PCDHB10	OSTN
PCDHB11	OTC
PCDHB12	OTOF
PCDHB13	OTOG
PCDHB14	OTOGL
PCDHB15	OTOL1
PCDHB16	OTOMP
PCDHB17	OTOP1
PCDHB19P	OTOP2
PCDHB2	OTOP3

PCDHB20	OTOS
PCDHB21	OTP
PCDHB22	OTPA
PCDHB3	OTPB
PCDHB4	OTUB1
PCDHB5	OTUB2
PCDHB6	OTUD1
PCDHB7	OTUD3
PCDHB8	OTUD4
PCDHB9	OTUD5
PCDHGA1	OTUD6A
PCDHGA10	OTUD6B
PCDHGA11	OTUD6B-AS1
PCDHGA12	OTUD7A
PCDHGA2	OTUD7B
PCDHGA3	OTULIN
PCDHGA4	OTULINL
PCDHGA5	OTX1
PCDHGA6	OTX2
PCDHGA7	OTX2-AS1
PCDHGA8	OVAL
PCDHGA9	OVCA2
PCDHGB1	OVCH2
PCDHGB2	OVGP1
PCDHGB3	OVOL1
PCDHGB4	OVOL2
PCDHGB5	OVOS2
PCDHGB6	OXA1L
PCDHGB7	OXCT1
PCDHGC3	OXCT2
PCDHGC4	OXCT2A
PCDHGC5	OXER1
PCED1A	OXGR1
PCED1B	OXI-1
PCF11	OXLD1
PCGEM1	OXNAD1

PCGF1	OXR1
PCGF2	OXSM
PCGF3	OXSR1
PCGF5	OXT
PCGF5A	OXTR
PCGF6	P24B
PCID2	P2RX1
PCIF1	P2RX2
PCK1	P2RX3
PCK2	P2RX4
PCLAF	P2RX5
PCLO	P2RX6
PCM1	P2RX6P
PCMT1	P2RX7
PCMTD1	P2RY1
PCMTD2	P2RY10
PCMTL	P2RY11
PCNA	P2RY12
PCNA-AS1	P2RY13
PCNP	P2RY14
PCNT	P2RY2
PCNX	P2RY4
PCNX1	P2RY5
PCNX2	P2RY6
PCNX3	P2RY8
PCNX4	P3H1
PCOLCE	P3H2
PCOLCE2	P3H3
PCOTH	P3H4
PCP2	P4HA1
PCP4	P4HA2
PCP4L1	P4HA3
PCSK1	P4HB
PCSK1N	P4HTM
PCSK2	PA2G4
PCSK4	PA2G4P4

PCSK5	PAAF1
PCSK6	PABIR1
PCSK7	PABIR2
PCSK9	PABIR3
PCTP	PABP2
PCX	PABPC1
PCYOX1	PABPC1.S
PCYOX1L	PABPC1B
PCYT1A	PABPC1L
PCYT1AB	PABPC1L2A
PCYT1B	PABPC1L2B
PCYT1BA	PABPC3
PCYT2	PABPC4
PDAP1	PABPC4L
PDC	PABPC5
PDCD1	PABPN1
PDCD10	PABPN1L
PDCD11	PACC1
PDCD1LG2	PACRG
PDCD2	PACRG-AS1
PDCD2L	PACRG-AS3
PDCD4	PACRGL
PDCD4-AS1	PACS1
PDCD5	PACS2
PDCD6	PACSIN1
PDCD6IP	PACSIN2
PDCD7	PACSIN3
PDCL	PADI1
PDCL3	PADI2
PDE10A	PADI3
PDE11A	PADI4
PDE12	PADI6
PDE1A	PAEP
PDE1B	PAF1
PDE1C	PAFAH1B1
PDE2A	PAFAH1B1A

PDE3A	PAFAH1B1B
PDE3B	PAFAH1B2
PDE4A	PAFAH1B3
PDE4B	PAFAH2
PDE4BA	PAG1
PDE4C	PAGE1
PDE4D	PAGE2
PDE4DIP	PAGE2B
PDE5A	PAGE4
PDE6A	PAGE5
PDE6B	PAGR1
PDE6B-AS1	PAH
PDE6C	PAICS
PDE6D	PAIP1
PDE6G	PAIP2
PDE6H	PAIP2B
PDE7A	PAK1
PDE7B	PAK1IP1
PDE8A	PAK2
PDE8B	PAK3
PDE9A	PAK4
PDF	PAK5
PDGFA	PAK6
PDGFA-DT	PAK6-AS1
PDGFB	PALB2
PDGFC	PALD1
PDGFD	PALLD
PDGFRA	PALM
PDGFRB	PALM2AKAP2
PDGFRL	PALM3
PDHA1	PALMD
PDHB	PALS1
PDHX	PALS2
PDIA2	PAM
PDIA3	PAM16
PDIA4	PAMR1

PDIA5	PAN2
PDIA6	PAN3
PDIK1L	PAN3-AS1
PDILT	PANDAR
PDK1	PANK1
PDK2	PANK2
PDK2A	PANK3
PDK3	PANK4
PDK4	PANX1
PDL1	PANX2
PDLIM1	PANX3
PDLIM2	PAOX
PDLIM3	PAPLN
PDLIM4	PAPOLA
PDLIM5	PAPOLG
PDLIM7	PAPPA
PDP1	PAPPA2
PDP2	PAPSS1
PDPK1	PAPSS2
PDPN	PAQR3
PDPR	PAQR4
PDRG1	PAQR5
PDS5A	PAQR6
PDS5B	PAQR7
PDSS1	PAQR8
PDSS2	PAQR9
PDX1	PARD3
PDXDC1	PARD3B
PDXDC2P	PARD6A
PDXDC2P-NPIPB14P	PARD6B
PDXK	PARD6G
PDXP	PARD6G-AS1
PDYN	PARG
PDZD11	PARK7
PDZD2	PARL
PDZD3	PARM1

PDZD3A	PARN
PDZD4	PARP
PDZD7	PARP1
PDZD8	PARP10
PDZD9	PARP11
PDZK1	PARP12
PDZK1IP1	PARP14
PDZK1P1	PARP15
PDZPH1	PARP16
PDZRN3	PARP2
PDZRN4	PARP3
PEA15	PARP4
PEA15A	PARP6
PEAK1	PARP8
PEAK3	PARP9
PEAR1	PARPBP
PEBP1	PARS2
PEBP4	PART1
PECAM1	PARTICL
PECR	PARVA
PEDS1	PARVB
PEDS1-UBE2V1	PARVG
PEF1	PASD1
PEG10	PASK
PEG3	PATE1
PEG3-AS1	PATJ
PELATON	PATL1
PELI1	PATL2
PELI2	PATZ1
PELI3	PAWR
PELO	PAX1
PELP1	PAX2
PEMT	PAX3
PENK	PAX4
PEPD	PAX5
PER1	PAX6

PER1A	PAX6B
PER2	PAX6OS1
PER3	PAX7
PERM1	PAX7A
PERP	PAX8
PES1	PAX8-AS1
PET100	PAX9
PET117	PAXBP1
PEX1	PAXBP1-AS1
PEX10	PAXIP1
PEX11A	PAXIP1-DT
PEX11B	PAXX
PEX11G	PBDC1
PEX12	PBK
PEX13	PBLD
PEX14	PBLD1
PEX16	PBRM1
PEX19	PBX1
PEX2	PBX1A
PEX26	PBX2
PEX3	PBX3
PEX5L	PBX4
PEX6	PBXIP1
PEX7	PC
PF4	PCAP
PF4V1	PCAT1
PFAS	PCAT19
PFDN1	PCAT29
PFDN2	PCAT4
PFDN4	PCAT6
PFDN5	PCAT7
PFDN6	PCBD1
PFKFB1	PCBD2
PFKFB2	PCBP1
PFKFB3	PCBP1-AS1
PFKFB4	PCBP2

PFKFB4B	PCBP2-OT1
PFKL	PCBP3
PFKM	PCBP4
PFKMA	PCCA
PFKMB	PCCB
PFKP	PCDH1
PFN1	PCDH10
PFN2	PCDH11X
PFN3	PCDH11Y
PFN4	PCDH12
PGA3	PCDH15
PGA4	PCDH17
PGA5	PCDH18
PGAM1	PCDH19
PGAM1A	PCDH1GB2
PGAM1B	PCDH20
PGAM2	PCDH2AC
PGAM4	PCDH7
PGAM5	PCDH8
PGAP1	PCDH9
PGAP2	PCDHA10
PGAP3	PCDHA11
PGAP4	PCDHA12
PGAP6	PCDHA13
PGBD1	PCDHA2
PGBD3	PCDHA4
PGBD4	PCDHA5
PGBD5	PCDHA8
PGBP	PCDHA9
PGC	PCDHAC1
PGCP	PCDHAC2
PGD	PCDHB1
PGF	PCDHB10
PGGHG	PCDHB11
PGGT1B	PCDHB12
PGK1	PCDHB13

PGK2	PCDHB14
PGLS	PCDHB15
PGLS-DT	PCDHB16
PGLYRP1	PCDHB17
PGLYRP2	PCDHB19P
PGLYRP3	PCDHB2
PGLYRP4	PCDHB20
PGM1	PCDHB21
PGM2	PCDHB22
PGM2L1	PCDHB3
PGM3	PCDHB4
PGM5	PCDHB5
PGM5-AS1	PCDHB6
PGM5P2	PCDHB7
PGP	PCDHB8
PGP-5	PCDHB9
PGPEP1	PCDHGA1
PGPEP1L	PCDHGA10
PGR	PCDHGA11
PGRMC1	PCDHGA12
PGRMC2	PCDHGA2
PGRP-LC	PCDHGA3
PGRP-SC1B	PCDHGA4
PGS1	PCDHGA5
PHACTR1	PCDHGA6
PHACTR2	PCDHGA7
PHACTR3	PCDHGA8
PHACTR4	PCDHGA9
PHAF1	PCDHGB1
PHAX	PCDHGB2
PHB	PCDHGB3
PHB1	PCDHGB4
PHB2	PCDHGB5
PHC1	PCDHGB6
PHC2	PCDHGB7
PHC3	PCDHGC3

PHETA1	PCDHGC4
PHETA2	PCDHGC5
PHEX	PCED1A
PHF1	PCED1B
PHF10	PCF11
PHF11	PCGEM1
PHF11D	PCGF1
PHF12	PCGF2
PHF13	PCGF3
PHF14	PCGF5
PHF19	PCGF5A
PHF2	PCGF6
PHF20	PCID2
PHF20A	PCIF1
PHF20L1	PCK1
PHF21A	PCK2
PHF21B	PCLAF
PHF23	PCLO
PHF24	PCM1
PHF2P1	PCMT1
PHF3	PCMTD1
PHF5A	PCMTD2
PHF6	PCMTL
PHF7	PCNA
PHF8	PCNA-AS1
PHGDH	PCNP
PHGR1	PCNT
PHIP	PCNX
PHKA1	PCNX1
PHKA2	PCNX2
PHKB	PCNX3
PHKG1	PCNX4
PHKG1A	PCOLCE
PHKG1B	PCOLCE2
PHKG2	PCOTH
PHLDA1	PCP2

PHLDA2	PCP4
PHLDA3	PCP4L1
PHLDB1	PCSK1
PHLDB2	PCSK1N
PHLDB3	PCSK2
PHLPP	PCSK4
PHLPP1	PCSK5
PHLPP2	PCSK6
PHOSPHO1	PCSK7
PHOSPHO2	PCSK9
PHOSPHO2-KLHL23	PCTP
PHOX2	PCX
PHOX2A	PCYOX1
PHOX2B	PCYOX1L
PHPT1	PCYT1A
PHTF1	PCYT1AB
PHTF2	PCYT1B
PHUM_PHUM154200	PCYT1BA
PHXR2	PCYT2
PHXR4	PDAP1
PHXR5	PDB1
PHYH	PDC
PHYHD1	PDCD1
PHYHIP	PDCD10
PHYHIPL	PDCD11
PHYKPL	PDCD1LG2
PI15	PDCD2
PI16	PDCD2L
PI3	PDCD4
PI4K2A	PDCD4-AS1
PI4K2B	PDCD5
PI4KA	PDCD6
PI4KAP1	PDCD6IP
PI4KAP2	PDCD7
PI4KB	PDCL
PIANP	PDCL3

PIAS1	PDE10A
PIAS2	PDE11A
PIAS3	PDE12
PIAS4	PDE1A
PIBF1	PDE1B
PICALM	PDE1C
PICART1	PDE2A
PICK1	PDE3A
PID1	PDE3B
PIDD1	PDE4A
PIERCE1	PDE4B
PIERCE2	PDE4BA
PIEZO1	PDE4C
PIEZO2	PDE4D
PIF1	PDE4DIP
PIFO	PDE5A
PIGA	PDE6A
PIGB	PDE6B
PIGC	PDE6B-AS1
PIGF	PDE6C
PIGG	PDE6D
PIGH	PDE6G
PIGK	PDE6H
PIGL	PDE7A
PIGM	PDE7B
PIGN	PDE8A
PIGO	PDE8B
PIGP	PDE9A
PIGQ	PDF
PIGR	PDGFA
PIGS	PDGFA-DT
PIGT	PDGFB
PIGU	PDGFC
PIGV	PDGFD
PIGW	PDGFRA
PIGX	PDGFRB

PIGY	PDGFRL
PIGZ	PDHA1
PIH1D1	PDHB
PIH1D2	PDHX
PIK3AP1	PDIA2
PIK3C2A	PDIA3
PIK3C2B	PDIA3P1
PIK3C2G	PDIA4
PIK3C3	PDIA5
PIK3CA	PDIA6
PIK3CB	PDIK1L
PIK3CD	PDILT
PIK3CD-AS1	PDK1
PIK3CG	PDK2
PIK3IP1	PDK2A
PIK3R1	PDK3
PIK3R2	PDK4
PIK3R3	PDL1
PIK3R3A	PDLIM1
PIK3R4	PDLIM2
PIK3R5	PDLIM3
PIK3R6	PDLIM4
PIKFYVE	PDLIM5
PILRA	PDLIM7
PILRB	PDP1
PILRB2	PDP2
PIM1	PDPK1
PIM2	PDPN
PIM3	PDPR
PIMREG	PDRG1
PIN1	PDS5A
PIN4	PDS5B
PIN4P1	PDSS1
PINK1	PDSS2
PINLYP	PDX1
PINX1	PDXDC1

PIP	PDXDC2P
PIP4K2A	PDXDC2P-NPIPB14P
PIP4K2B	PDXK
PIP4K2C	PDXP
PIP4P1	PDYN
PIP4P2	PDZD11
PIP5K1A	PDZD2
PIP5K1B	PDZD3
PIP5K1BB	PDZD3A
PIP5K1C	PDZD4
PIP5K1P1	PDZD7
PIP5KL1	PDZD8
PIPOX	PDZD9
PIPSL	PDZK1
PIR	PDZK1IP1
PIRA1	PDZK1P1
PIRA11	PDZPH1
PIRA6	PDZRN3
PIRB	PDZRN4
PIRT	PEA15
PISD	PEA15A
PISD-PS3	PEAK1
PITHD1	PEAK3
PITPNA	PEAR1
PITPNA-AS1	PEBP1
PITPNB	PEBP4
PITPNB.2.L	PECAM1
PITPNC1	PECR
PITPNM1	PEDS1
PITPNM2	PEDS1-UBE2V1
PITPNM3	PEF1
PITRM1	PEG10
PITRM1-AS1	PEG3
PITX1	PEG3-AS1
PITX1-AS1	PELATON
PITX2	PELI1

PITX3	PELI2
PIWIL1	PELI3
PIWIL2	PELO
PIWIL3	PELP1
PIWIL4	PEMT
PIX	PENK
PJA1	PEPD
PJA2	PER1
PJVK	PER1A
PKD1	PER2
PKD1L1	PER3
PKD1L2	PERM1
PKD1L3	PERP
PKD1P1	PES1
PKD1P6	PET100
PKD2	PET117
PKD2L1	PEX1
PKD2L2	PEX10
PKDCC	PEX11A
PKHD1	PEX11B
PKHD1L1	PEX11G
PKIA	PEX12
PKIB	PEX13
PKIG	PEX14
PKLR	PEX16
PKM	PEX19
PKMYT1	PEX2
PKN1	PEX26
PKN2	PEX3
PKN3	PEX5L
PKNOX1	PEX6
PKNOX2	PEX7
PKP1	PF4
PKP2	PF4V1
PKP3	PFAS
PKP4	PFDN1

PLA1A	PFDN2
PLA2G10	PFDN4
PLA2G12A	PFDN5
PLA2G12B	PFDN6
PLA2G15	PFKFB1
PLA2G1B	PFKFB2
PLA2G2A	PFKFB3
PLA2G2C	PFKFB4
PLA2G2D	PFKFB4B
PLA2G2E	PFKL
PLA2G2F	PFKM
PLA2G3	PFKMA
PLA2G4A	PFKMB
PLA2G4AA	PFKP
PLA2G4B	PFN1
PLA2G4C	PFN2
PLA2G4D	PFN3
PLA2G4E	PFN4
PLA2G4E-AS1	PGA3
PLA2G4F	PGA4
PLA2G5	PGA5
PLA2G6	PGAM1
PLA2G7	PGAM1A
PLA2R1	PGAM1B
PLAA	PGAM2
PLAAT1	PGAM4
PLAAT2	PGAM5
PLAAT3	PGAP1
PLAAT4	PGAP2
PLAAT5	PGAP3
PLAC1	PGAP4
PLAC4	PGAP6
PLAC8	PGBD1
PLAC8L1	PGBD3
PLAC9	PGBD4
PLAG1	PGBD5

PLAGL1	PGBP
PLAGL2	PGC
PLAT	PGCP
PLAU	PGD
PLAUR	PGF
PLB1	PGGHG
PLBD1	PGGT1B
PLBD2	PGK1
PLCB1	PGK2
PLCB2	PGLS
PLCB3	PGLS-DT
PLCB4	PGLYRP1
PLCD1	PGLYRP2
PLCD3	PGLYRP3
PLCD4	PGLYRP4
PLCE1	PGM1
PLCE1-AS2	PGM2
PLCG1	PGM2L1
PLCG2	PGM3
PLCH1	PGM5
PLCH2	PGM5-AS1
PLCL1	PGM5P2
PLCL2	PGP
PLCXD1	PGP-5
PLCXD2	PGPEP1
PLCXD3	PGPEP1L
PLD1	PGR
PLD1B	PGRMC1
PLD2	PGRMC2
PLD3	PGRP-LC
PLD4	PGRP-SC1B
PLD5	PGS1
PLD6	PHACTR1
PLEC	PHACTR2
PLEK	PHACTR3
PLEK2	PHACTR4

PLEKHA1	PHAF1
PLEKHA2	PHAX
PLEKHA3	PHB
PLEKHA4	PHB1
PLEKHA5	PHB2
PLEKHA6	PHC1
PLEKHA7	PHC2
PLEKHA8	PHC3
PLEKHA8P1	PHETA1
PLEKHB1	PHETA2
PLEKHB2	PHEX
PLEKHD1	PHF1
PLEKHF1	PHF10
PLEKHF2	PHF11
PLEKHG1	PHF11D
PLEKHG2	PHF12
PLEKHG3	PHF13
PLEKHG4	PHF14
PLEKHG4B	PHF19
PLEKHG5	PHF2
PLEKHG6	PHF20
PLEKHG7	PHF20A
PLEKHH1	PHF20L1
PLEKHH2	PHF21A
PLEKHH3	PHF21B
PLEKHJ1	PHF23
PLEKHM1	PHF24
PLEKHM1P1	PHF2P1
PLEKHM2	PHF3
PLEKHM3	PHF5A
PLEKHN1	PHF6
PLEKHO1	PHF7
PLEKHO2	PHF8
PLEKHS1	PHGDH
PLG	PHGR1
PLGLB1	PHIP

PLGLB2	PHKA1
PLGRKT	PHKA2
PLIN	PHKB
PLIN1	PHKG1
PLIN2	PHKG1A
PLIN3	PHKG1B
PLIN4	PHKG2
PLIN5	PHLDA1
PLK	PHLDA2
PLK1	PHLDA3
PLK2	PHLDB1
PLK3	PHLDB2
PLK4	PHLDB3
PLK5	PHLPP
PLLP	PHLPP1
PLN	PHLPP2
PLOD1	PHOSPHO1
PLOD2	PHOSPHO2
PLOD3	PHOSPHO2-KLHL23
PLP1	PHOX2
PLP1A	PHOX2A
PLP1B	PHOX2B
PLP2	PHPT1
PLPBP	PHRF1
PLPBP.L	PHTF1
PLPP1	PHTF2
PLPP2	PHUM_PHUM154200
PLPP3	PHXR2
PLPP4	PHXR4
PLPP5	PHXR5
PLPP6	PHYH
PLPP7	PHYHD1
PLPPR1	PHYHIP
PLPPR2	PHYHIPL
PLPPR3	PHYKPL
PLPPR4	PI15

PLPPR5	PI16
PLRG1	PI3
PLS1	PI4K2A
PLS3	PI4K2B
PLSCR1	PI4KA
PLSCR2	PI4KAP1
PLSCR3	PI4KAP2
PLSCR4	PI4KB
PLTP	PIANP
PLVAP	PIAS1
PLXDC1	PIAS2
PLXDC2	PIAS3
PLXNA1	PIAS4
PLXNA2	PIBF1
PLXNA3	PICALM
PLXNA4	PICART1
PLXNB1	PICK1
PLXNB2	PID1
PLXNB3	PIDD1
PLXNC1	PIERCE1
PLXND1	PIERCE2
PM20D1	PIEZO1
PM20D2	PIEZO2
PMAIP1	PIF1
PMEL	PIFO
PMEPA1	PIGA
PMF1	PIGB
PMF1-BGLAP	PIGC
PMFBP1	PIGF
PML	PIGG
PMM1	PIGH
PMM2	PIGK
PMP2	PIGL
PMP22	PIGM
PMP22A	PIGN
PMPCA	PIGO

PMPCB	PIGP
PMS1	PIGQ
PMS2	PIGR
PMS2CL	PIGS
PMS2P2	PIGT
PMS2P3	PIGU
PMS2P4	PIGV
PMS2P5	PIGW
PMS2P6	PIGX
PMS2P9	PIGY
PMVK	PIGZ
PNCK	PIH1D1
PNISR	PIH1D2
PNKD	PIK3AP1
PNKP	PIK3C2A
PNLDC1	PIK3C2B
PNLIP	PIK3C2G
PNLIPRP1	PIK3C3
PNLIPRP2	PIK3CA
PNLIPRP3	PIK3CB
PNMA1	PIK3CD
PNMA2	PIK3CD-AS1
PNMA3	PIK3CG
PNMA5	PIK3IP1
PNMA6A	PIK3R1
PNMA8A	PIK3R2
PNMA8B	PIK3R3
PNMT	PIK3R3A
PNN	PIK3R4
PNO1	PIK3R5
PNOC	PIK3R6
PNP	PIKFYVE
PNP4B	PILRA
PNP5B	PILRB
PNPLA1	PILRB2
PNPLA2	PIM1

PNPLA3	PIM2
PNPLA4	PIM3
PNPLA5	PIMREG
PNPLA6	PIN1
PNPLA7	PIN4
PNPLA8	PIN4P1
PNPO	PINK1
PNPT1	PINLYP
PNRC1	PINX1
PNRC2	PIP
PNUTS	PIP4K2A
POC1A	PIP4K2B
POC1B	PIP4K2C
POC1B-GALNT4	PIP4P1
POC5	PIP4P2
PODN	PIP5K1A
PODNL1	PIP5K1B
PODXL	PIP5K1BB
PODXL2	PIP5K1C
POF1B	PIP5K1P1
POFUT1	PIP5KL1
POFUT2	PIPOX
POGK	PIPSL
POGLUT1	PIR
POGLUT2	PIRA1
POGLUT3	PIRA11
POGZ	PIRA6
POLA1	PIRB
POLA2	PIRT
POLB	PISD
POLD1	PISD-PS3
POLD2	PITHD1
POLD3	PITPNA
POLD4	PITPNA-AS1
POLDIP2	PITPNB
POLDIP3	PITPNB.2.L

POLE	PITPNC1
POLE2	PITPNM1
POLE3	PITPNM2
POLE4	PITPNM3
POLG	PITRM1
POLG2	PITRM1-AS1
POLH	PITX1
POLH-1	PITX1-AS1
POLI	PITX2
POLK	PITX3
POLL	PIWIL1
POLM	PIWIL2
POLN	PIWIL3
POLQ	PIWIL4
POLR1A	PIX
POLR1B	PJA1
POLR1C	PJA2
POLR1D	PJVK
POLR1E	PKD1
POLR1F	PKD1L1
POLR1G	PKD1L2
POLR1H	PKD1L3
POLR1HASP	PKD1P1
POLR2A	PKD1P6
POLR2B	PKD2
POLR2C	PKD2L1
POLR2D	PKD2L2
POLR2E	PKDCC
POLR2F	PKHD1
POLR2G	PKHD1L1
POLR2H	PKIA
POLR2H-PS1	PKIB
POLR2I	PKIG
POLR2J	PKLR
POLR2J2	PKM
POLR2J3	PKMYT1

POLR2K	PKN1
POLR2L	PKN2
POLR2M	PKN3
POLR3A	PKNOX1
POLR3B	PKNOX2
POLR3C	PKP1
POLR3D	PKP2
POLR3E	PKP3
POLR3F	PKP4
POLR3G	PLA1A
POLR3GL	PLA2G10
POLR3H	PLA2G12A
POLR3K	PLA2G12B
POLRMT	PLA2G15
POM121	PLA2G1B
POM121C	PLA2G2A
POM121L10P	PLA2G2C
POM121L12	PLA2G2D
POM121L1P	PLA2G2E
POM121L4P	PLA2G2F
POM121L8P	PLA2G3
POM121L9P	PLA2G4A
POMC	PLA2G4AA
POMGNT1	PLA2G4B
POMGNT2	PLA2G4C
POMK	PLA2G4D
POMP	PLA2G4E
POMT1	PLA2G4E-AS1
POMT2	PLA2G4F
POMZP3	PLA2G5
PON1	PLA2G6
PON2	PLA2G7
PON3	PLA2R1
POP1	PLAA
POP4	PLAAT1
POP5	PLAAT2

POP7	PLAAT3
POPDC2	PLAAT4
POPDC3	PLAAT5
POR	PLAC1
PORCN	PLAC4
POSTN	PLAC8
POT1	PLAC8L1
POTEA	PLAC9
POTEC	PLAC9P1
POTED	PLAG1
POTEE	PLAGL1
POTEF	PLAGL2
POTEG	PLAT
POTEI	PLAU
POTEJ	PLAUR
POTEM	PLB1
POU1F1	PLBD1
POU2AF1	PLBD2
POU2AF3	PLCB1
POU2F1	PLCB2
POU2F2	PLCB3
POU2F3	PLCB4
POU3F1	PLCD1
POU3F2	PLCD3
POU3F3	PLCD4
POU3F3A	PLCE1
POU3F3B	PLCE1-AS2
POU4F1	PLCG1
POU4F2	PLCG2
POU4F3	PLCH1
POU5F1	PLCH2
POU5F1B	PLCL1
POU5F1P3	PLCL2
POU5F1P4	PLCXD1
POU5F2	PLCXD2
POU5F3.1.L	PLCXD3

POU6F1	PLD1
POU6F2	PLD1B
POU6F2-AS2	PLD2
PP12613	PLD3
PP7080	PLD4
PPA1	PLD5
PPA2	PLD6
PPAN	PLEC
PPAN-P2RY11	PLEK
PPAP2A	PLEK2
PPAP2B	PLEKHA1
PPARA	PLEKHA2
PPARD	PLEKHA3
PPARG	PLEKHA4
PPARGC1A	PLEKHA5
PPARGC1B	PLEKHA6
PPAT	PLEKHA7
PPBP	PLEKHA8
PPBPP1	PLEKHA8P1
PPBPP2	PLEKHB1
PPCDC	PLEKHB2
PPCS	PLEKHD1
PPDPF	PLEKHF1
PPDPFA	PLEKHF2
PPFIA1	PLEKHG1
PPFIA3	PLEKHG2
PPFIA4	PLEKHG3
PPFIBP1	PLEKHG4
PPFIBP2	PLEKHG4B
PPHLN1	PLEKHG5
PPIA	PLEKHG6
PPIAL4A	PLEKHG7
PPIAL4G	PLEKHH1
PPIAP46	PLEKHH2
PPIB	PLEKHH3
PPIC	PLEKHJ1

PPID	PLEKHM1
PPIE	PLEKHM1P1
PPIEL	PLEKHM2
PPIF	PLEKHM3
PPIG	PLEKHN1
PPIH	PLEKHO1
PPIL1	PLEKHO2
PPIL2	PLEKHS1
PPIL3	PLF
PPIL4	PLG
PPIL6	PLGLB1
PPIP5K1	PLGLB2
PPL	PLGRKT
PPM1A	PLIN
PPM1B	PLIN1
PPM1D	PLIN2
PPM1E	PLIN3
PPM1F	PLIN4
PPM1G	PLIN5
PPM1H	PLK
PPM1J	PLK1
PPM1K	PLK2
PPM1L	PLK3
PPM1LB	PLK4
PPM1M	PLK5
PPM1N	PLLP
PPM1NA	PLN
PPME1	PLOD1
PPOX	PLOD2
PPP1CA	PLOD3
PPP1CB	PLP1
PPP1CC	PLP1A
PPP1R10	PLP1B
PPP1R11	PLP2
PPP1R12A	PLPBP
PPP1R12B	PLPBP.L

PPP1R12C	PLPP1
PPP1R13B	PLPP2
PPP1R13B-DT	PLPP3
PPP1R13L	PLPP4
PPP1R14A	PLPP5
PPP1R14B	PLPP6
PPP1R14C	PLPP7
PPP1R14D	PLPPR1
PPP1R15A	PLPPR2
PPP1R15B	PLPPR3
PPP1R16A	PLPPR4
PPP1R16B	PLPPR5
PPP1R17	PLRG1
PPP1R18	PLS1
PPP1R1A	PLS3
PPP1R1B	PLSCR1
PPP1R1C	PLSCR2
PPP1R2	PLSCR3
PPP1R21	PLSCR4
PPP1R26	PLTP
PPP1R26-AS1	PLVAP
PPP1R27	PLXDC1
PPP1R2B	PLXDC2
PPP1R2C	PLXNA1
PPP1R32	PLXNA2
PPP1R35	PLXNA3
PPP1R36	PLXNA4
PPP1R37	PLXNB1
PPP1R3A	PLXNB2
PPP1R3B	PLXNB3
PPP1R3C	PLXNC1
PPP1R3D	PLXND1
PPP1R3DB	PM20D1
PPP1R3E	PM20D2
PPP1R3F	PMAIP1
PPP1R3G	PMCH

PPP1R42	PMEL
PPP1R7	PMEPA1
PPP1R8	PMF1
PPP1R9A	PMF1-BGLAP
PPP1R9B	PMFBP1
PPP2CA	PML
PPP2CB	PMM1
PPP2R1A	PMM2
PPP2R1B	PMP2
PPP2R2A	PMP22
PPP2R2B	PMP22A
PPP2R2C	PMPCA
PPP2R2D	PMPCB
PPP2R3A	PMS1
PPP2R3B	PMS2
PPP2R3C	PMS2CL
PPP2R4	PMS2P2
PPP2R5A	PMS2P3
PPP2R5B	PMS2P4
PPP2R5C	PMS2P5
PPP2R5D	PMS2P6
PPP2R5E	PMS2P9
PPP3CA	PMVK
PPP3CB	PNCK
PPP3CC	PNISR
PPP3R1	PNKD
PPP3R2	PNKP
PPP4C	PNLDC1
PPP4R1	PNLIP
PPP4R1L	PNLIPRP1
PPP4R1L-PS	PNLIPRP2
PPP4R2	PNLIPRP3
PPP4R3A	PNMA1
PPP4R3B	PNMA2
PPP4R4	PNMA3
PPP5C	PNMA5

PPP5D1P	PNMA6A
PPP6C	PNMA8A
PPP6R1	PNMA8B
PPP6R2	PNMT
PPP6R3	PNN
PPRC1	PNO1
PPT1	PNOC
PPT2	PNP
PPT2-EGFL8	PNP4B
PPTC7	PNP5B
PPWD1	PNPLA1
PPY	PNPLA2
PPY2P	PNPLA3
PQBP1	PNPLA4
PQLC3	PNPLA5
PQN-54	PNPLA6
PQN-76	PNPLA7
PQN-78	PNPLA8
PQN-91	PNPO
PRADC1	PNPT1
PRAF2	PNRC1
PRAG1	PNRC2
PRAM1	PNUTS
PRAME	POC1A
PRAMEF11	POC1B
PRAMEF13	POC1B-GALNT4
PRAMEF15	POC5
PRAMEF18	PODN
PRAMEF19	PODNL1
PRAMEF20	PODXL
PRAMEF22	PODXL2
PRAMEF5	POF1B
PRAMEF7	POFUT1
PRAMEL5	POFUT2
PRAMEL7	POGK
PRAP1	POGLUT1

PRB1	POGLUT2
PRB2	POGLUT3
PRB3	POGZ
PRC1	POLA1
PRCC	POLA2
PRCD	POLB
PRCP	POLD1
PRDM1	POLD2
PRDM10	POLD3
PRDM10-DT	POLD4
PRDM11	POLDIP2
PRDM12	POLDIP3
PRDM13	POLE
PRDM14	POLE2
PRDM15	POLE3
PRDM16	POLE4
PRDM16-DT	POLG
PRDM2	POLG2
PRDM4	POLH
PRDM5	POLH-1
PRDM6	POLI
PRDM7	POLK
PRDM8	POLL
PRDX1	POLM
PRDX2	POLN
PRDX3	POLQ
PRDX4	POLR1A
PRDX5	POLR1B
PRDX6	POLR1C
PRDX6-AS1	POLR1D
PRDX6B	POLR1E
PREB	POLR1F
PRECSIT	POLR1G
PRELID1	POLR1H
PRELID2	POLR1HASP
PRELID3A	POLR2A

PRELID3B	POLR2B
PRELP	POLR2C
PREP	POLR2D
PREPL	POLR2E
PREX1	POLR2F
PREX2	POLR2G
PRF1	POLR2H
PRG1	POLR2H-PS1
PRG2	POLR2I
PRG3	POLR2J
PRG4	POLR2J2
PRH1-PRR4	POLR2J3
PRH2	POLR2K
PRICKLE1	POLR2L
PRICKLE2	POLR2M
PRICKLE3	POLR3A
PRICKLE4	POLR3B
PRIM1	POLR3C
PRIM2	POLR3D
PRIMA1	POLR3E
PRIMPOL	POLR3F
PRKAA1	POLR3G
PRKAA2	POLR3GL
PRKAB1	POLR3H
PRKAB2	POLR3K
PRKACA	POLRMT
PRKACB	POM121
PRKACG	POM121C
PRKAG1	POM121L10P
PRKAG2	POM121L12
PRKAG2-AS1	POM121L1P
PRKAG3	POM121L4P
PRKAR1A	POM121L8P
PRKAR1AA	POM121L9P
PRKAR1B	POMC
PRKAR2A	POMGNT1

PRKAR2A-AS1	POMGNT2
PRKAR2B	POMK
PRKCA	POMP
PRKCB	POMT1
PRKCD	POMT2
PRKCE	POMZP3
PRKCG	PON1
PRKCH	PON2
PRKCI	PON3
PRKCQ	POP1
PRKCQ-AS1	POP4
PRKCSH	POP5
PRKCZ	POP7
PRKD1	POPDC2
PRKD2	POPDC3
PRKD3	POR
PRKDC	PORCN
PRKG1	POSTN
PRKG2	POT1
PRKN	POTEA
PRKRA	POTEC
PRKRIP1	POTED
PRKX	POTEE
PRKXP1	POTEF
PRKY	POTEG
PRL	POTEI
PRL2B1	POTEJ
PRL2C2	POTEM
PRL3A1	POU1F1
PRL7B1	POU2AF1
PRL8A4	POU2AF3
PRLH	POU2F1
PRLHR	POU2F2
PRLR	POU2F3
PRM1	POU3F1
PRM2	POU3F2

PRM3	POU3F3
PRMT1	POU3F3A
PRMT2	POU3F3B
PRMT3	POU4F1
PRMT5	POU4F2
PRMT6	POU4F3
PRMT7	POU5F1
PRMT8	POU5F1B
PRND	POU5F1P3
PRNP	POU5F1P4
PROB1	POU5F2
PROC	POU5F3.1.L
PROCA1	POU6F1
PROCR	POU6F2
PRODH	POU6F2-AS2
PRODH2	PP12613
PROK1	PP7080
PROK2	PPA1
PROKR1	PPA2
PROKR2	PPAN
PROL1	PPAN-P2RY11
PROM1	PPAP2A
PROM2	PPAP2B
PROP1	PPARA
PRORP	PPARD
PRORSD1	PPARG
PRORSD1P	PPARGC1A
PROS1	PPARGC1B
PROSER1	PPAT
PROSER2	PPBP
PROSER3	PPBPP1
PROX1	PPBPP2
PROX2	PPCDC
PROZ	PPCS
PRP2L1	PPDPF
PRPF18	PPDPFA

PRPF19	PPFIA1
PRPF3	PPFIA3
PRPF31	PPFIA4
PRPF38A	PPFIBP1
PRPF38B	PPFIBP2
PRPF39	PPHLN1
PRPF4	PPIA
PRPF40A	PPIAL4A
PRPF40B	PPIAL4G
PRPF4B	PPIAP46
PRPF6	PPIB
PRPF8	PPIC
PRPH	PPID
PRPH2	PPIE
PRPS1	PPIEL
PRPS1B	PPIF
PRPS1L3	PPIG
PRPS2	PPIH
PRPSAP1	PPIL1
PRPSAP2	PPIL2
PRR11	PPIL3
PRR12	PPIL4
PRR13	PPIL6
PRR14	PPIP5K1
PRR14L	PPL
PRR15	PPM1A
PRR15L	PPM1B
PRR16	PPM1D
PRR18	PPM1E
PRR19	PPM1F
PRR20A	PPM1G
PRR20B	PPM1H
PRR20C	PPM1J
PRR20D	PPM1K
PRR20E	PPM1L
PRR21	PPM1LB

PRR22	PPM1M
PRR23A	PPM1N
PRR23B	PPM1NA
PRR23C	PPME1
PRR25	PPOX
PRR27	PPP1CA
PRR29	PPP1CB
PRR29-AS1	PPP1CC
PRR3	PPP1R10
PRR30	PPP1R11
PRR32	PPP1R12A
PRR33	PPP1R12B
PRR34-AS1	PPP1R12C
PRR35	PPP1R13B
PRR36	PPP1R13B-DT
PRR4	PPP1R13L
PRR5	PPP1R14A
PRR5L	PPP1R14B
PRR7	PPP1R14C
PRRC1	PPP1R14D
PRRC2A	PPP1R15A
PRRC2B	PPP1R15B
PRRC2C	PPP1R16A
PRRG1	PPP1R16B
PRRG2	PPP1R17
PRRG3	PPP1R18
PRRG4	PPP1R1A
PRRT1	PPP1R1B
PRRT2	PPP1R1C
PRRT3	PPP1R2
PRRT4	PPP1R21
PRRX1	PPP1R26
PRRX1A	PPP1R26-AS1
PRRX2	PPP1R27
PRSS1	PPP1R2B
PRSS12	PPP1R2C

PRSS16	PPP1R32
PRSS2	PPP1R35
PRSS21	PPP1R36
PRSS22	PPP1R37
PRSS23	PPP1R3A
PRSS27	PPP1R3B
PRSS3	PPP1R3C
PRSS30	PPP1R3D
PRSS30P	PPP1R3DB
PRSS32	PPP1R3E
PRSS33	PPP1R3F
PRSS34	PPP1R3G
PRSS35	PPP1R42
PRSS36	PPP1R7
PRSS37	PPP1R8
PRSS38	PPP1R9A
PRSS40A	PPP1R9B
PRSS41	PPP2CA
PRSS42	PPP2CB
PRSS42P	PPP2R1A
PRSS45P	PPP2R1B
PRSS54	PPP2R2A
PRSS57	PPP2R2B
PRSS58	PPP2R2C
PRSS8	PPP2R2D
PRTFDC1	PPP2R3A
PRTG	PPP2R3B
PRTGA	PPP2R3C
PRTN3	PPP2R4
PRUNE	PPP2R5A
PRUNE1	PPP2R5B
PRUNE2	PPP2R5C
PRX	PPP2R5D
PRX2540-1	PPP2R5E
PRX2540-2	PPP3CA
PRX6005	PPP3CB

PRXL2A	PPP3CC
PRXL2B	PPP3R1
PRXL2C	PPP3R2
PRY	PPP4C
PSA	PPP4R1
PSAP	PPP4R1L
PSAPL1	PPP4R1L-PS
PSAT1	PPP4R2
PSAT1P3	PPP4R3A
PSCA	PPP4R3B
PSD	PPP4R4
PSD2	PPP5C
PSD3	PPP5D1P
PSD4	PPP6C
PSEN1	PPP6R1
PSEN2	PPP6R2
PSENEN	PPP6R3
PSG1	PPRC1
PSG11	PPT1
PSG17	PPT2
PSG19	PPT2-EGFL8
PSG20	PPTC7
PSG23	PPWD1
PSG3	PPY
PSG4	PPY2P
PSG5	PQBP1
PSG6	PQLC3
PSG8	PQN-54
PSG9	PQN-76
PSIP1	PQN-78
PSKH1	PQN-91
PSMA1	PRADC1
PSMA2	PRAF2
PSMA3	PRAG1
PSMA3-AS1	PRAL
PSMA4	PRAM1

PSMA5	PRAME
PSMA6	PRAMEF11
PSMA7	PRAMEF13
PSMB1	PRAMEF15
PSMB10	PRAMEF18
PSMB11	PRAMEF19
PSMB2	PRAMEF20
PSMB3	PRAMEF22
PSMB4	PRAMEF5
PSMB5	PRAMEF7
PSMB6	PRAMEL5
PSMB7	PRAMEL7
PSMB8	PRAP1
PSMB8-AS1	PRB1
PSMB9	PRB2
PSMC1	PRB3
PSMC2	PRC1
PSMC3	PRCC
PSMC3IP	PRCD
PSMC4	PRCP
PSMC5	PRDM1
PSMC6	PRDM10
PSMD1	PRDM10-DT
PSMD10	PRDM11
PSMD11	PRDM12
PSMD12	PRDM13
PSMD13	PRDM14
PSMD14	PRDM15
PSMD2	PRDM16
PSMD3	PRDM16-DT
PSMD4	PRDM2
PSMD5	PRDM4
PSMD6	PRDM5
PSMD6-AS2	PRDM6
PSMD7	PRDM7
PSMD8	PRDM8

PSMD9	PRDX1
PSME1	PRDX2
PSME2	PRDX3
PSME3	PRDX4
PSME3IP1	PRDX5
PSME4	PRDX6
PSMF1	PRDX6-AS1
PSMG1	PRDX6B
PSMG2	PREB
PSMG3	PRECSIT
PSMG3-AS1	PRELID1
PSMG4	PRELID2
PSORS1C1	PRELID3A
PSORS1C2	PRELID3B
PSPC1	PRELP
PSPH	PREP
PSPN	PREPL
PSRC1	PREX1
PSTK	PREX2
PSTPIP1	PRF1
PSTPIP2	PRG1
PTAFR	PRG2
PTAR1	PRG3
PTAR1.S	PRG4
PTBP1	PRH1-PRR4
PTBP2	PRH2
PTBP3	PRICKLE1
PTCD1	PRICKLE2
PTCD2	PRICKLE3
PTCD3	PRICKLE4
PTCH1	PRIM1
PTCH2	PRIM2
PTCHD1	PRIMA1
PTCHD3	PRIMPOL
PTCHD4	PRKAA1
PTDSS1	PRKAA2

PTDSS2	PRKAB1
PTEN	PRKAB2
PTER	PRKACA
PTGDR	PRKACB
PTGDR2	PRKACG
PTGDS	PRKAG1
PTGER1	PRKAG2
PTGER2	PRKAG2-AS1
PTGER2A	PRKAG3
PTGER3	PRKAR1A
PTGER4	PRKAR1AA
PTGER4P2-CDK2AP2P2	PRKAR1B
PTGES	PRKAR2A
PTGES2	PRKAR2A-AS1
PTGES3	PRKAR2B
PTGES3L1	PRKCA
PTGFR	PRKCB
PTGFRN	PRKCD
PTGIR	PRKCE
PTGIS	PRKCG
PTGR1	PRKCH
PTGR2	PRKCI
PTGR3	PRKCQ
PTGS1	PRKCQ-AS1
PTGS2	PRKCSH
PTH1R	PRKCZ
PTH2	PRKCZ-AS1
PTH2R	PRKD1
PTHLH	PRKD2
PTK2	PRKD3
PTK2B	PRKDC
PTK6	PRKG1
PTK7	PRKG2
PTMA	PRKN
PTMS	PRKRA
PTN	PRKRIP1

PTOV1	PRKX
PTOV1-AS1	PRKXP1
PTP4A1	PRKY
PTP4A2	PRL
PTP4A3	PRL2B1
PTP-5.1	PRL2C2
PTPA	PRL3A1
PTPDC1	PRL7B1
PTPMT1	PRL8A4
PTPN1	PRLH
PTPN11	PRLHR
PTPN12	PRLR
PTPN13	PRM1
PTPN14	PRM2
PTPN18	PRM3
PTPN2	PRMT1
PTPN20	PRMT2
PTPN21	PRMT3
PTPN22	PRMT5
PTPN23	PRMT6
PTPN3	PRMT7
PTPN4	PRMT8
PTPN5	PRNCR1
PTPN6	PRND
PTPN7	PRNP
PTPN9	PROB1
PTPRA	PROC
PTPRB	PROCA1
PTPRC	PROCR
PTPRCAP	PRODH
PTPRD	PRODH2
PTPRD-AS1	PROK1
PTPRE	PROK2
PTPRF	PROKR1
PTPRG	PROKR2
PTPRG-AS1	PROL1

PTPRH	PROM1
PTPRJ	PROM2
PTPRK	PROP1
PTPRM	PRORP
PTPRN	PRORSD1
PTPRN2	PRORSD1P
PTPRO	PROS1
PTPRQ	PROSER1
PTPRR	PROSER2
PTPRS	PROSER3
PTPRT	PROX1
PTPRU	PROX2
PTPRZ1	PROZ
PTRH1	PRP2L1
PTRH2	PRPF18
PTRHD1	PRPF19
PTS	PRPF3
PTTG1	PRPF31
PTTG1IP	PRPF38A
PTTG3P	PRPF38B
PTX3	PRPF39
PTX4	PRPF4
PUDP	PRPF40A
PUF60	PRPF40B
PUM1	PRPF4B
PUM2	PRPF6
PUM3	PRPF8
PURA	PRPH
PURB	PRPH2
PURG	PRPS1
PURPL	PRPS1B
PUS1	PRPS1L3
PUS10	PRPS2
PUS3	PRPSAP1
PUS7	PRPSAP2
PUS7L	PRR11

PUSL1	PRR12
PVALB	PRR13
PVALB1	PRR14
PVALB2	PRR14L
PVALB3	PRR15
PVALB4	PRR15L
PVALB8	PRR16
PVALEF	PRR18
PVR	PRR19
PVRIG	PRR20A
PVRL1	PRR20B
PVT1	PRR20C
PWAR1	PRR20D
PWAR4	PRR20E
PWAR5	PRR21
PWAR6	PRR22
PWP1	PRR23A
PWP2	PRR23B
PWRN1	PRR23C
PWWP2A	PRR25
PWWP2B	PRR27
PWWP3A	PRR29
PWWP3B	PRR29-AS1
PWWP4B	PRR3
PXDC1	PRR30
PXDN	PRR32
PXDNL	PRR33
PXK	PRR34-AS1
PXMP2	PRR35
PXMP4	PRR36
PXN	PRR4
PXN-AS1	PRR5
PXT1	PRR5L
PXYLP1	PRR7
PYCARD	PRRC1
PYCARD-AS1	PRRC2A

PYCR1	PRRC2B
PYCR2	PRRC2C
PYCR3	PRRG1
PYCRL	PRRG2
PYDC1	PRRG3
PYGB	PRRG4
PYGL	PRRT1
PYGM	PRRT2
PYGMB	PRRT3
PYGO1	PRRT4
PYGO2	PRRX1
PYHIN1	PRRX1A
PYK	PRRX2
PYM1	PRSS1
PYROXD1	PRSS12
PYROXD2	PRSS16
PYY	PRSS2
PYY2	PRSS21
PYYA	PRSS22
PZP	PRSS23
QARS	PRSS27
QARS1	PRSS3
QDPR	PRSS30
QDPRA	PRSS30P
QK	PRSS32
QKI	PRSS33
QKIA	PRSS34
QPCT	PRSS35
QPCTL	PRSS36
QPRT	PRSS37
QRFP	PRSS38
QRFPR	PRSS40A
QRICH1	PRSS41
QRICH2	PRSS42
QRSL1	PRSS42P
QSER1	PRSS45P

QSOX1	PRSS50
QSOX2	PRSS54
QTRT1	PRSS55
QTRT2	PRSS57
R09E10.2	PRSS58
R3HCC1	PRSS8
R3HCC1L	PRTFDC1
R3HDM1	PRTG
R3HDM2	PRTGA
R3HDM4	PRTN3
R3HDML	PRUNE
RAB10	PRUNE1
RAB11A	PRUNE2
RAB11B	PRX
RAB11B-AS1	PRX2540-1
RAB11FIP1	PRX2540-2
RAB11FIP2	PRX6005
RAB11FIP3	PRXL2A
RAB11FIP4	PRXL2B
RAB11FIP5	PRXL2C
RAB12	PRY
RAB13	PSA
RAB14	PSAP
RAB15	PSAPL1
RAB17	PSAT1
RAB18	PSAT1P3
RAB19	PSCA
RAB1A	PSD
RAB1B	PSD2
RAB2	PSD3
RAB20	PSD4
RAB21	PSEN1
RAB22A	PSEN2
RAB23	PSENEN
RAB24	PSG1
RAB25	PSG11

RAB26	PSG17
RAB27A	PSG19
RAB27B	PSG2
RAB28	PSG20
RAB29	PSG23
RAB2A	PSG3
RAB2B	PSG4
RAB30	PSG5
RAB30-DT	PSG6
RAB31	PSG8
RAB32	PSG9
RAB33A	PSIP1
RAB33B	PSKH1
RAB34	PSMA1
RAB35	PSMA2
RAB36	PSMA3
RAB37	PSMA3-AS1
RAB38	PSMA4
RAB39B	PSMA5
RAB3A	PSMA6
RAB3B	PSMA7
RAB3C	PSMB1
RAB3D	PSMB10
RAB3GAP1	PSMB11
RAB3GAP2	PSMB2
RAB3IL1	PSMB3
RAB3IP	PSMB4
RAB4	PSMB5
RAB40A	PSMB6
RAB40AL	PSMB7
RAB40B	PSMB8
RAB40C	PSMB8-AS1
RAB41	PSMB9
RAB42	PSMC1
RAB43	PSMC2
RAB44	PSMC3

RAB4A	PSMC3IP
RAB4B	PSMC4
RAB4B-EGLN2	PSMC5
RAB5A	PSMC6
RAB5B	PSMD1
RAB5C	PSMD10
RAB5IF	PSMD11
RAB6A	PSMD12
RAB6B	PSMD13
RAB6C	PSMD14
RAB7	PSMD2
RAB7A	PSMD3
RAB7B	PSMD4
RAB7L1	PSMD5
RAB8A	PSMD6
RAB8B	PSMD6-AS2
RAB9	PSMD7
RAB9A	PSMD8
RAB9B	PSMD9
RABAC1	PSME1
RABEP1	PSME2
RABEP2	PSME3
RABEPK	PSME3IP1
RABGAP1	PSME4
RABGAP1L	PSMF1
RABGEF1	PSMG1
RABGEF1P1	PSMG2
RABGGTA	PSMG3
RABGGTB	PSMG3-AS1
RABIF	PSMG4
RABL2A	PSORS1C1
RABL2B	PSORS1C2
RABL3	PSPC1
RABL6	PSPH
RAC1	PSPN
RAC2	PSRC1

RAC3	PSTK
RACGAP1	PSTPIP1
RACK1	PSTPIP2
RAD1	PTAFR
RAD17	PTAR1
RAD18	PTAR1.S
RAD21	PTBP1
RAD21L1	PTBP2
RAD23A	PTBP3
RAD23B	PTCD1
RAD50	PTCD2
RAD51	PTCD3
RAD51AP1	PTCH1
RAD51AP2	PTCH2
RAD51-AS1	PTCHD1
RAD51B	PTCHD3
RAD51C	PTCHD4
RAD51D	PTDSS1
RAD51L3-RFFL	PTDSS2
RAD52	PTEN
RAD54B	PTER
RAD54L	PTGDR
RAD54L2	PTGDR2
RAD9A	PTGDS
RAD9B	PTGER1
RADIL	PTGER2
RADX	PTGER2A
RAE1	PTGER3
RAET1A	PTGER4
RAET1D	PTGER4P2-CDK2AP2P2
RAET1E	PTGES
RAET1E-AS1	PTGES2
RAET1G	PTGES3
RAET1K	PTGES3L1
RAET1L	PTGFR
RAF	PTGFRN

RAF1	PTGIR
RAG1	PTGIS
RAG1AP1	PTGR1
RAI1	PTGR2
RAI14	PTGR3
RAI1-AS1	PTGS1
RAI2	PTGS2
RALA	PTH1R
RALAA	PTH2
RALB	PTH2R
RALBP1	PTHLH
RALGAPA1	PTK2
RALGAPA2	PTK2B
RALGAPB	PTK6
RALGDS	PTK7
RALGPS1	PTMA
RALGPS2	PTMAP4
RALY	PTMS
RALYL	PTN
RAMAC	PTOV1
RAMP1	PTOV1-AS1
RAMP2	PTP4A1
RAMP3	PTP4A2
RAN	PTP4A3
RANBP1	PTP-5.1
RANBP10	PTPA
RANBP17	PTPDC1
RANBP2	PTPMT1
RANBP3	PTPN1
RANBP3-DT	PTPN11
RANBP3L	PTPN12
RANBP6	PTPN13
RANBP9	PTPN14
RANGAP1	PTPN18
RANGRF	PTPN2
RAP1A	PTPN20

RAP1B	PTPN21
RAP1BL	PTPN22
RAP1GAP	PTPN23
RAP1GAP2	PTPN3
RAP1GAP2A	PTPN4
RAP1GDS1	PTPN5
RAP2A	PTPN6
RAP2B	PTPN7
RAP2C	PTPN9
RAPGEF1	PTPRA
RAPGEF2	PTPRB
RAPGEF3	PTPRC
RAPGEF4	PTPRCAP
RAPGEF5	PTPRD
RAPGEF6	PTPRD-AS1
RAPGEFL1	PTPRE
RAPH1	PTPRF
RAPSN	PTPRG
RARA	PTPRG-AS1
RARB	PTPRH
RARG	PTPRJ
RARGA	PTPRK
RARRES1	PTPRM
RARRES2	PTPRN
RARS	PTPRN2
RARS1	PTPRO
RARS2	PTPRQ
RASA1	PTPRR
RASA2	PTPRS
RASA3	PTPRT
RASA4	PTPRU
RASA4CP	PTPRZ1
RASAL1	PTRH1
RASAL2	PTRH2
RASAL2-AS1	PTRHD1
RASAL3	PTS

RASD1	PTTG1
RASD2	PTTG1IP
RASEF	PTTG2
RASGEF1A	PTTG3P
RASGEF1B	PTX3
RASGEF1BA	PTX4
RASGEF1C	PUDP
RASGRF1	PUF60
RASGRF2	PUM1
RASGRP1	PUM2
RASGRP2	PUM3
RASGRP3	PURA
RASGRP4	PURB
RASIP1	PURG
RASL10A	PURPL
RASL10B	PUS1
RASL11A	PUS10
RASL11B	PUS3
RASL12	PUS7
RASSF1	PUS7L
RASSF10	PUSL1
RASSF2	PVALB
RASSF3	PVALB1
RASSF4	PVALB2
RASSF5	PVALB3
RASSF6	PVALB4
RASSF7	PVALB8
RASSF8	PVALEF
RASSF8-AS1	PVR
RASSF9	PVRIG
RAVER1	PVRL1
RAVER2	PVT1
RAX	PWAR1
RAX2	PWAR4
RB	PWAR5
RB1	PWAR6

RB1CC1	PWP1
RB1-DT	PWP2
RBAK	PWRN1
RBAK-RBAKDN	PWWP2A
RBBP4	PWWP2B
RBBP5	PWWP3A
RBBP6	PWWP3B
RBBP7	PWWP4B
RBBP8	PXDC1
RBBP8NL	PXDN
RBBP9	PXDNL
RBCK1	PXK
RBFA	PXMP2
RBFOX1	PXMP4
RBFOX1L	PXN
RBFOX2	PXN-AS1
RBFOX3	PXT1
RBIS	PXYLP1
RBKS	PYCARD
RBL1	PYCARD-AS1
RBL2	PYCR1
RBM10	PYCR2
RBM11	PYCR3
RBM12	PYCRL
RBM12B	PYDC1
RBM12B-AS1	PYGB
RBM13	PYGL
RBM14	PYGM
RBM14-RBM4	PYGMB
RBM15	PYGO1
RBM15B	PYGO2
RBM17	PYHIN1
RBM18	PYK
RBM19	PYM1
RBM20	PYROXD1
RBM22	PYROXD2

RBM23	PYY
RBM24	PYY2
RBM25	PYYA
RBM26	PZP
RBM26-AS1	QARS
RBM27	QARS1
RBM28	QDPR
RBM3	QDPRA
RBM33	QK
RBM34	QKI
RBM38	QKIA
RBM38-AS1	QPCT
RBM39	QPCTL
RBM4	QPRT
RBM41	QRFP
RBM42	QRFPR
RBM43	QRICH1
RBM44	QRICH2
RBM45	QRSL1
RBM46	QSER1
RBM47	QSOX1
RBM48	QSOX2
RBM4B	QTRT1
RBM5	QTRT2
RBM6	R09E10.2
RBM7	R3HCC1
RBM8A	R3HCC1L
RBMS1	R3HDM1
RBMS2	R3HDM2
RBMS3	R3HDM4
RBMX	R3HDML
RBMX2	RAB10
RBMXL1	RAB11A
RBMXL1B	RAB11B
RBMXL2	RAB11B-AS1
RBMXL3	RAB11FIP1

RBP1	RAB11FIP2
RBP2	RAB11FIP3
RBP2A	RAB11FIP4
RBP2B	RAB11FIP5
RBP3	RAB12
RBP4	RAB13
RBP4L	RAB14
RBP5	RAB15
RBP7	RAB17
RBPJ	RAB18
RBPJA	RAB19
RBPJL	RAB1A
RBPMS	RAB1B
RBPMS2	RAB2
RBPMS-AS1	RAB20
RBSN	RAB21
RBX1	RAB22A
RC3H1	RAB23
RC3H2	RAB24
RCAN1	RAB25
RCAN1A	RAB26
RCAN2	RAB27A
RCAN3	RAB27B
RCBTB1	RAB28
RCBTB2	RAB29
RCC1	RAB2A
RCC1L	RAB2B
RCC2	RAB30
RCCD1	RAB30-DT
RCE1	RAB31
RCHY1	RAB32
RCL1	RAB33A
RCN1	RAB33B
RCN2	RAB34
RCN3	RAB35
RCOR1	RAB36

RCOR2	RAB37
RCOR3	RAB38
RCSD1	RAB39B
RD3	RAB3A
RDH1	RAB3B
RDH10	RAB3C
RDH10-AS1	RAB3D
RDH10B	RAB3GAP1
RDH11	RAB3GAP2
RDH12	RAB3IL1
RDH12L	RAB3IP
RDH13	RAB4
RDH14	RAB40A
RDH16	RAB40AL
RDH19	RAB40B
RDH2	RAB40C
RDH5	RAB41
RDH7	RAB42
RDM1	RAB43
RDX	RAB44
REC114	RAB4A
REC8	RAB4B
RECK	RAB4B-EGLN2
RECQL	RAB5A
RECQL4	RAB5B
RECQL5	RAB5C
REEP1	RAB5IF
REEP2	RAB6A
REEP3	RAB6B
REEP4	RAB6C
REEP5	RAB7
REEP6	RAB7A
REG1A	RAB7B
REG1B	RAB7L1
REG2	RAB8A
REG3A	RAB8B

REG3B	RAB9
REG3D	RAB9A
REG3G	RAB9B
REG4	RABAC1
REL	RABEP1
RELA	RABEP2
RELB	RABEPK
RELCH	RABGAP1
REL-DT	RABGAP1L
RELL1	RABGEF1
RELL2	RABGEF1P1
RELN	RABGGTA
RELT	RABGGTB
REM1	RABIF
REM2	RABL2A
REN	RABL2B
RENBP	RABL3
REP15	RABL6
REPIN1	RAC1
REPS1	RAC2
REPS2	RAC3
RER1	RACGAP1
RERE	RACK1
REREB	RAD1
RERG	RAD17
RESF1	RAD18
REST	RAD21
RET	RAD21L1
RETN	RAD23A
RETNLA	RAD23B
RETNLB	RAD50
RETNLG	RAD51
RETREG1	RAD51AP1
RETREG2	RAD51AP2
RETREG3	RAD51-AS1
RETSAT	RAD51B

REV1	RAD51C
REV3L	RAD51D
REX1BD	RAD51L3-RFFL
REXO1	RAD52
REXO1L1P	RAD54B
REXO1L2P	RAD54L
REXO2	RAD54L2
REXO4	RAD9A
REXO5	RAD9B
RFC1	RADIL
RFC2	RADX
RFC3	RAE1
RFC4	RAET1A
RFC5	RAET1D
RFESD	RAET1E
RFFL	RAET1E-AS1
RFK	RAET1G
RFLNA	RAET1K
RFLNB	RAET1L
RFNG	RAF
RFPL1S	RAF1
RFPL2	RAG1
RFPL3	RAG1AP1
RFPL3S	RAG2
RFPL4B	RAI1
RFT1	RAI14
RFTN1	RAI1-AS1
RFTN2	RAI2
RFWD3	RALA
RFX1	RALAA
RFX2	RALB
RFX3	RALBP1
RFX3-DT	RALGAPA1
RFX4	RALGAPA2
RFX5	RALGAPB
RFX6	RALGDS

RFX7	RALGPS1
RFX8	RALGPS2
RFXANK	RALY
RFXAP	RALYL
RGCC	RAMAC
RGD1304567	RAMP1
RGD1304622	RAMP2
RGD1304728	RAMP3
RGD1305184	RAN
RGD1305298	RANBP1
RGD1305807	RANBP10
RGD1306271	RANBP17
RGD1307100	RANBP2
RGD1307182	RANBP3
RGD1307603	RANBP3-DT
RGD1308065	RANBP3L
RGD1309104	RANBP6
RGD1309350	RANBP9
RGD1309362	RANGAP1
RGD1309534	RANGRF
RGD1309779	RAP1A
RGD1309808	RAP1B
RGD1310166	RAP1BL
RGD1310352	RAP1GAP
RGD1310587	RAP1GAP2
RGD1311300	RAP1GAP2A
RGD1311595	RAP1GDS1
RGD1559588	RAP2A
RGD1559600	RAP2B
RGD1559896	RAP2C
RGD1560289	RAPGEF1
RGD1561157	RAPGEF2
RGD1561636	RAPGEF3
RGD1562339	RAPGEF4
RGD1562625	RAPGEF5
RGD1562844	RAPGEF6

RGD1563888	RAPGEFL1
RGD1564480	RAPH1
RGD1564664	RAPSN
RGD1564899	RARA
RGD1565033	RARB
RGD1565685	RARG
RGD1566265	RARGA
RGD735065	RARRES1
RGL1	RARRES2
RGL2	RARS
RGL3	RARS1
RGL4	RARS2
RGMA	RASA1
RGMB	RASA2
RGN	RASA3
RGP1	RASA4
RGPD1	RASA4CP
RGPD2	RASAL1
RGPD3	RASAL2
RGPD4	RASAL2-AS1
RGPD4-AS1	RASAL3
RGPD5	RASD1
RGPD8	RASD2
RGR	RASEF
RGS1	RASGEF1A
RGS10	RASGEF1B
RGS11	RASGEF1BA
RGS12	RASGEF1C
RGS13	RASGRF1
RGS14	RASGRF2
RGS16	RASGRP1
RGS17	RASGRP2
RGS18	RASGRP3
RGS19	RASGRP4
RGS2	RASIP1
RGS20	RASL10A

RGS21	RASL10B
RGS22	RASL11A
RGS3	RASL11B
RGS4	RASL12
RGS5	RASSF1
RGS5-AS1	RASSF10
RGS7	RASSF2
RGS7BP	RASSF3
RGS7BPB	RASSF4
RGS8	RASSF5
RGS9	RASSF6
RGS9BP	RASSF7
RHAG	RASSF8
RHBDD1	RASSF8-AS1
RHBDD2	RASSF9
RHBDD3	RAVER1
RHBDF1	RAVER2
RHBDF2	RAX
RHBDL1	RAX2
RHBDL2	RB
RHBDL3	RB1
RHBG	RB1CC1
RHCG	RB1-DT
RHCGL1	RBAK
RHD	RBAK-RBAKDN
RHEB	RBBP4
RHEBL1	RBBP5
RHEX	RBBP6
RHNO1	RBBP7
RHO	RBBP8
RHOA	RBBP8NL
RHOB	RBBP9
RHOBTB1	RBCK1
RHOBTB2	RBFA
RHOBTB2B	RBFOX1
RHOBTB3	RBFOX1L

RHOC	RBFOX2
RHOD	RBFOX3
RHOF	RBIS
RHOG	RBKS
RHOH	RBL1
RHOJ	RBL2
RHOL	RBM10
RHOQ	RBM11
RHOQP2	RBM12
RHOT1	RBM12B
RHOT2	RBM12B-AS1
RHOU	RBM13
RHOV	RBM14
RHOX3	RBM14-RBM4
RHOX5	RBM15
RHOX6	RBM15B
RHOX9	RBM17
RHOXF1	RBM18
RHOXF1-AS1	RBM19
RHOXF2	RBM20
RHOXF2B	RBM22
RHPN1	RBM23
RHPN1-AS1	RBM24
RHPN2	RBM25
RIBC1	RBM26
RIBC2	RBM26-AS1
RIC1	RBM27
RIC3	RBM28
RIC-3	RBM3
RIC8A	RBM33
RIC8B	RBM34
RICTOR	RBM38
RIDA	RBM38-AS1
RIF1	RBM39
RIIAD1	RBM4
RILP	RBM41

RILPL1	RBM42
RILPL2	RBM43
RIMBP2	RBM44
RIMBP3	RBM45
RIMBP3B	RBM46
RIMBP3C	RBM47
RIMKLA	RBM48
RIMKLB	RBM4B
RIMOC1	RBM5
RIMS1	RBM6
RIMS2	RBM7
RIMS3	RBM8A
RIMS4	RBMS1
RIN1	RBMS2
RIN2	RBMS3
RIN3	RBMX
RING1	RBMX2
RINL	RBMXL1
RINT1	RBMXL1B
RIOK1	RBMXL2
RIOK2	RBMXL3
RIOK3	RBP1
RIOX1	RBP2
RIOX2	RBP2A
RIPK1	RBP2B
RIPK2	RBP3
RIPK3	RBP4
RIPK4	RBP4L
RIPOR1	RBP5
RIPOR2	RBP7
RIPOR3	RBPJ
RIPPLY1	RBPJA
RIPPLY2	RBPJL
RIPPLY3	RBPMS
RIT1	RBPMS2
RIT2	RBPMS-AS1

RITA1	RBSN
RLBP1	RBX1
RLF	RC3H1
RLIM	RC3H2
RLN1	RCAN1
RLN2	RCAN1A
RLN3	RCAN2
RMC1	RCAN3
RMDN1	RCBTB1
RMDN2	RCBTB2
RMDN3	RCC1
RMI1	RCC1L
RMI2	RCC2
RMND1	RCCD1
RMND5A	RCE1
RMND5B	RCHY1
RMRP	RCL1
RMST	RCN1
RN4.5S	RCN2
RN7SK	RCN3
RN7SL1	RCOR1
RNA18SN4	RCOR2
RNA45SN4	RCOR3
RNASE1	RCSD1
RNASE10	RCVRN
RNASE13	RD3
RNASE2	RDH1
RNASE2A	RDH10
RNASE2B	RDH10-AS1
RNASE3	RDH10B
RNASE4	RDH11
RNASE6	RDH12
RNASE7	RDH12L
RNASEH1	RDH13
RNASEH1-DT	RDH14
RNASEH2A	RDH16

RNASEH2B	RDH19
RNASEH2C	RDH2
RNASEK	RDH5
RNASEL	RDH7
RNASET2	RDM1
RND1	RDX
RND2	REC114
RND3	REC8
RNF10	RECK
RNF103	RECQL
RNF11	RECQL4
RNF111	RECQL5
RNF112	REEP1
RNF113A	REEP2
RNF113B	REEP3
RNF114	REEP4
RNF115	REEP5
RNF121	REEP6
RNF122	REG1A
RNF123	REG1B
RNF125	REG2
RNF126	REG3A
RNF126P1	REG3B
RNF128	REG3D
RNF13	REG3G
RNF130	REG4
RNF133	REL
RNF135	RELA
RNF138	RELB
RNF139	RELCH
RNF14	REL-DT
RNF141	RELL1
RNF144A	RELL2
RNF144B	RELN
RNF145	RELT
RNF146	REM1

RNF148	REM2
RNF149	REN
RNF150	RENBP
RNF150B	REP15
RNF152	REPIN1
RNF157	REPS1
RNF157-AS1	REPS2
RNF165	RER1
RNF166	RERE
RNF167	REREB
RNF168	RERG
RNF169	RESF1
RNF17	REST
RNF170	RET
RNF175	RETN
RNF180	RETNLA
RNF181	RETNLB
RNF182	RETNLG
RNF183	RETREG1
RNF185	RETREG2
RNF186	RETREG3
RNF187	RETSAT
RNF19A	REV1
RNF19B	REV3L
RNF2	REX1BD
RNF20	REXO1
RNF207	REXO1L1P
RNF208	REXO1L2P
RNF212	REXO2
RNF213	REXO4
RNF213-AS1	REXO5
RNF214	RFC1
RNF215	RFC2
RNF216	RFC3
RNF216P1	RFC4
RNF217	RFC5

RNF220	RFESD
RNF222	RFFL
RNF223	RFK
RNF224	RFLNA
RNF225	RFLNB
RNF227	RFNG
RNF24	RFPL1S
RNF25	RFPL2
RNF26	RFPL3
RNF31	RFPL3S
RNF32	RFPL4B
RNF32-DT	RFT1
RNF34	RFTN1
RNF38	RFTN2
RNF39	RFWD3
RNF4	RFX1
RNF40	RFX2
RNF41	RFX3
RNF43	RFX3-DT
RNF44	RFX4
RNF5	RFX5
RNF5P1	RFX6
RNF6	RFX7
RNF7	RFX8
RNF8	RFXANK
RNFT1	RFXAP
RNFT2	RGCC
RNGTT	RGD1304567
RNH1	RGD1304622
RNLS	RGD1304728
RNMT	RGD1305184
RNPC3	RGD1305298
RNPEP	RGD1305807
RNPEPL1	RGD1306271
RNPS1	RGD1307100
RNR1	RGD1307182

RNR2	RGD1307603
RNU11	RGD1308065
RNU12	RGD1309104
RNU4-2	RGD1309350
RNU5D-1	RGD1309362
RNU5E-1	RGD1309534
RNU6ATAC	RGD1309779
RNVU1-18	RGD1309808
RNVU1-7	RGD1310166
RNY5	RGD1310352
RO60	RGD1310587
ROBO1	RGD1311300
ROBO2	RGD1311595
ROBO3	RGD1559588
ROBO4	RGD1559600
ROCK	RGD1559896
ROCK1	RGD1560289
ROCK1P1	RGD1561157
ROCK2	RGD1561636
ROGDI	RGD1562339
ROM1	RGD1562625
ROM1B	RGD1562844
ROMO1	RGD1563888
ROPN1	RGD1564480
ROPN1B	RGD1564664
ROPN1L	RGD1564899
ROR1	RGD1565033
ROR2	RGD1565685
RORA	RGD1566265
RORB	RGD735065
RORC	RGL1
ROS	RGL2
ROS1	RGL3
RP1	RGL4
RP1L1	RGMA
RP2	RGMB

RP9	RGMB-AS1
RP9P	RGN
RPA1	RGP1
RPA2	RGPD1
RPA3	RGPD2
RPA4	RGPD3
RPAIN	RGPD4
RPAIN.L	RGPD4-AS1
RPAP1	RGPD5
RPAP2	RGPD8
RPAP3	RGR
RPE	RGS1
RPE65	RGS10
RPF1	RGS11
RPF2	RGS12
RPGR	RGS13
RPGRIP1	RGS14
RPGRIP1L	RGS16
RPH3A	RGS17
RPH3AL	RGS18
RPIA	RGS19
RPL10	RGS2
RPL10A	RGS20
RPL10L	RGS21
RPL10P12	RGS22
RPL12	RGS3
RPL13	RGS4
RPL13A	RGS5
RPL13AP20	RGS5-AS1
RPL13AP3	RGS6
RPL13AP5	RGS7
RPL13AP6	RGS7BP
RPL13P5	RGS7BPB
RPL14	RGS8
RPL14P1	RGS9
RPL15	RGS9BP

RPL17	RHAG
RPL18	RHBDD1
RPL18A	RHBDD2
RPL18AP3	RHBDD3
RPL19	RHBDF1
RPL21	RHBDF2
RPL21P4	RHBDL1
RPL21P44	RHBDL2
RPL22	RHBDL3
RPL22L1	RHBG
RPL23	RHCG
RPL23A	RHCGL1
RPL23AP1	RHD
RPL23AP44	RHEB
RPL23AP53	RHEBL1
RPL23AP87	RHEX
RPL23P8	RHNO1
RPL24	RHO
RPL26	RHOA
RPL27	RHOB
RPL27A	RHOBTB1
RPL27P2	RHOBTB2
RPL28	RHOBTB2B
RPL29	RHOBTB3
RPL29P15	RHOC
RPL29P2	RHOD
RPL3	RHOF
RPL30	RHOG
RPL31	RHOH
RPL32	RHOJ
RPL32P3	RHOL
RPL34	RHOQ
RPL34-DT	RHOQP2
RPL35A	RHOT1
RPL36	RHOT2
RPL36A	RHOU

RPL36A-HNRNPH2	RHOV
RPL36AL	RHOX3
RPL36AP33	RHOX5
RPL37	RHOX6
RPL37A	RHOX9
RPL37P6	RHOXF1
RPL38	RHOXF1-AS1
RPL39	RHOXF2
RPL39L	RHOXF2B
RPL3L	RHPN1
RPL4	RHPN1-AS1
RPL41	RHPN2
RPL5	RIBC1
RPL6	RIBC2
RPL7	RIC1
RPL7A	RIC3
RPL7L1	RIC-3
RPL8	RIC8A
RPL9	RIC8B
RPLP0	RICTOR
RPLP0P2	RIDA
RPLP1	RIF1
RPLP2	RIIAD1
RPLP2P3	RILP
RPN1	RILPL1
RPN2	RILPL2
RPP14	RIMBP2
RPP21	RIMBP3
RPP25	RIMBP3B
RPP25L	RIMBP3C
RPP30	RIMKLA
RPP38	RIMKLB
RPP38-DT	RIMOC1
RPP40	RIMS1
RPPH1	RIMS2
RPRD1A	RIMS3

RPRD1B	RIMS4
RPRD2	RIN1
RPRL2	RIN2
RPRM	RIN3
RPRML	RING1
RPS10	RINL
RPS10L1	RINT1
RPS10-NUDT3	RIOK1
RPS10P7	RIOK2
RPS11	RIOK3
RPS12	RIOX1
RPS13	RIOX2
RPS14	RIPK1
RPS15	RIPK2
RPS15A	RIPK3
RPS15AP11	RIPK4
RPS15AP12	RIPOR1
RPS15AP17	RIPOR2
RPS15AP19	RIPOR3
RPS15AP24	RIPPLY1
RPS16	RIPPLY2
RPS16P5	RIPPLY3
RPS17	RIT1
RPS17P5	RIT2
RPS18	RITA1
RPS18P12	RLBP1
RPS19	RLF
RPS19BP1	RLIM
RPS2	RLN1
RPS20	RLN2
RPS21	RLN3
RPS23	RMC1
RPS24	RMDN1
RPS24P9	RMDN2
RPS25	RMDN3
RPS26	RMI1

RPS26L	RMI2
RPS26P11	RMND1
RPS26P35	RMND5A
RPS27	RMND5B
RPS27A	RMRP
RPS27L	RMST
RPS27P19	RN4.5S
RPS27P29	RN7SK
RPS28	RN7SL1
RPS29	RNA18SN4
RPS2P32	RNA45SN4
RPS2P35	RNASE1
RPS3	RNASE10
RPS3A	RNASE13
RPS4X	RNASE2
RPS4Y1	RNASE2A
RPS4Y2	RNASE2B
RPS5	RNASE3
RPS6	RNASE4
RPS6KA1	RNASE6
RPS6KA2	RNASE7
RPS6KA3	RNASEH1
RPS6KA4	RNASEH1-DT
RPS6KA5	RNASEH2A
RPS6KA6	RNASEH2B
RPS6KB1	RNASEH2C
RPS6KB2	RNASEK
RPS6KC1	RNASEL
RPS6KL1	RNASET2
RPS7	RND1
RPS7P5	RND2
RPS8P10	RND3
RPS9	RNF10
RPSA	RNF103
RPSAP52	RNF11
RPTN	RNF111

RPTOR	RNF112
RPTOROS	RNF113A
RPUSD1	RNF113B
RPUSD2	RNF114
RPUSD3	RNF115
RPUSD4	RNF121
RRAD	RNF122
RRAGA	RNF123
RRAGB	RNF125
RRAGC	RNF126
RRAGD	RNF126P1
RRAS	RNF128
RRAS2	RNF13
RRBP1	RNF130
RREB1	RNF133
RRH	RNF135
RRM1	RNF138
RRM2	RNF139
RRM2.1.L	RNF14
RRM2B	RNF141
RRN3	RNF144A
RRN3P1	RNF144B
RRN3P2	RNF145
RRP1	RNF146
RRP12	RNF148
RRP15	RNF149
RRP1B	RNF150
RRP36	RNF150B
RRP7A	RNF152
RRP7BP	RNF157
RRP8	RNF157-AS1
RRP9	RNF165
RRS1	RNF166
RRS1-DT	RNF167
RS1	RNF168
RS1A	RNF169

RSAD1	RNF17
RSAD2	RNF170
RSBN1	RNF175
RSBN1L	RNF180
RSC1A1	RNF181
RSF1	RNF182
RSKR	RNF183
RSL1D1	RNF185
RSL24D1	RNF186
RSPH1	RNF187
RSPH10B	RNF19A
RSPH10B2	RNF19B
RSPH14	RNF2
RSPH3	RNF20
RSPH3A	RNF207
RSPH4A	RNF208
RSPH6A	RNF212
RSPH9	RNF213
RSPO1	RNF213-AS1
RSPO2	RNF214
RSPO3	RNF215
RSPO4	RNF216
RSPRY1	RNF216P1
RSRC1	RNF217
RSRC2	RNF220
RSRP1	RNF222
RSU1	RNF223
RT1-BA	RNF224
RT1-BB	RNF225
RT1-CE12	RNF227
RT1-CE16	RNF24
RT1-CE5	RNF25
RT1-CL	RNF26
RT1-DA	RNF31
RT1-DB1	RNF32
RT1-DMA	RNF32-DT

RT1-DMB	RNF34
RT1-EC2	RNF38
RT1-M6-1	RNF39
RT1-N3	RNF4
RT1-S3	RNF40
RTBDN	RNF41
RTCA	RNF43
RTCB	RNF44
RTEL1	RNF5
RTEL1-TNFRSF6B	RNF5P1
RTF2	RNF6
RTFDC1	RNF7
RTKN	RNF8
RTKN2	RNFT1
RTL1	RNFT2
RTL10	RNGTT
RTL3	RNH1
RTL4	RNLS
RTL5	RNMT
RTL6	RNPC3
RTL8A	RNPEP
RTL8B	RNPEPL1
RTL8C	RNPS1
RTL9	RNR1
RTN1	RNR2
RTN1.L	RNU11
RTN2	RNU1-1
RTN2B	RNU12
RTN3	RNU4-2
RTN4	RNU5D-1
RTN4B	RNU5E-1
RTN4IP1	RNU6ATAC
RTN4R	RNVU1-18
RTN4RL1	RNVU1-7
RTN4RL2	RNY1
RTN4RL2A	RNY3

RTN4RL2B	RNY5
RTP1	RO60
RTP3	ROBO1
RTP4	ROBO2
RTP5	ROBO3
RTRAF	ROBO4
RTTN	ROCK
RUBCN	ROCK1
RUBCNL	ROCK1P1
RUFY1	ROCK2
RUFY1-AS1	ROGDI
RUFY2	ROM1
RUFY3	ROM1B
RUFY4	ROMO1
RUNDC1	ROPN1
RUNDC3A	ROPN1B
RUNDC3AB	ROPN1L
RUNDC3B	ROR1
RUNX1	ROR2
RUNX1T1	RORA
RUNX2	RORB
RUNX2-AS1	RORC
RUNX3	ROS
RUP2	ROS1
RUSC1	RP1
RUSC1-AS1	RP1L1
RUSC2	RP2
RUSF1	RP9
RUVBL1	RP9P
RUVBL2	RPA1
RWDD1	RPA2
RWDD2A	RPA3
RWDD2B	RPA4
RWDD3	RPAIN
RWDD4	RPAIN.L
RWDD4A	RPAP1

RXFP1	RPAP2
RXFP2	RPAP3
RXFP4	RPE
RXRA	RPE65
RXRB	RPF1
RXRG	RPF2
RXYLT1	RPGR
RYBP	RPGRIP1
RYK	RPGRIP1L
RYR1	RPH3A
RYR2	RPH3AL
RYR3	RPIA
S100A1	RPL10
S100A10	RPL10A
S100A11	RPL10L
S100A11P1	RPL10P12
S100A12	RPL12
S100A13	RPL13
S100A14	RPL13A
S100A16	RPL13AP20
S100A2	RPL13AP3
S100A3	RPL13AP5
S100A4	RPL13AP6
S100A5	RPL13P5
S100A6	RPL14
S100A7	RPL14P1
S100A7A	RPL15
S100A8	RPL17
S100A9	RPL17-C18orf32
S100B	RPL18
S100G	RPL18A
S100P	RPL18AP3
S100PBP	RPL19
S100Z	RPL21
S1PR1	RPL21P4
S1PR2	RPL21P44

S1PR3	RPL22
S1PR4	RPL22L1
S1PR5	RPL23
S6K	RPL23A
SAA	RPL23AP1
SAA1	RPL23AP44
SAA2	RPL23AP53
SAA3	RPL23AP87
SAA4	RPL23P8
SAAL1	RPL24
SAC3D1	RPL26
SACM1L	RPL27
SACS	RPL27A
SAE1	RPL27P2
SAFB	RPL28
SAFB2	RPL29
SAG	RPL29P15
SAGA	RPL29P2
SAGB	RPL3
SAH	RPL30
SALL1	RPL31
SALL2	RPL32
SALL3	RPL32P3
SALL4	RPL34
SALRNA3	RPL34-DT
SALS	RPL35A
SAMD1	RPL36
SAMD10	RPL36A
SAMD11	RPL36A-HNRNPH2
SAMD12	RPL36AL
SAMD13	RPL36AP33
SAMD14	RPL37
SAMD15	RPL37A
SAMD3	RPL37P6
SAMD4A	RPL38
SAMD4B	RPL39

SAMD5	RPL39L
SAMD8	RPL3L
SAMD9	RPL4
SAMD9L	RPL41
SAMHD1	RPL5
SAMM50	RPL6
SAMSN1	RPL7
SANBR	RPL7A
SAO	RPL7L1
SAP130	RPL8
SAP18	RPL9
SAP30	RPLP0
SAP30BP	RPLP0P2
SAP30L	RPLP1
SAP30L-AS1	RPLP2
SAPCD1	RPLP2P3
SAPCD2	RPN1
SAR1A	RPN2
SAR1B	RPP14
SARA	RPP21
SARAF	RPP25
SARDH	RPP25L
SARM1	RPP30
SARNP	RPP38
SARS	RPP38-DT
SARS1	RPP40
SARS2	RPPH1
SART1	RPRD1A
SART3	RPRD1B
SASH1	RPRD2
SASH3	RPRL2
SASS6	RPRM
SAT1	RPRML
SAT2	RPS10
SATB1	RPS10L1
SATB2	RPS10-NUDT3

SATB2-AS1	RPS10P7
SATL1	RPS11
SAV1	RPS12
SAXO1	RPS13
SAXO2	RPS14
SAYSD1	RPS15
SBDS	RPS15A
SBDSP1	RPS15AP11
SBF1	RPS15AP12
SBF1P1	RPS15AP17
SBF2	RPS15AP19
SBF2-AS1	RPS15AP24
SBK1	RPS16
SBK2	RPS16P5
SBK3	RPS17
SBNO1	RPS17P5
SBNO2	RPS18
SBPL	RPS18P12
SBSN	RPS19
SBSPON	RPS19BP1
SC5D	RPS2
SCAF1	RPS20
SCAF11	RPS21
SCAF4	RPS23
SCAF8	RPS24
SCAI	RPS24P9
SCAMP1	RPS25
SCAMP2	RPS26
SCAMP3	RPS26L
SCAMP4	RPS26P11
SCAMP5	RPS26P35
SCAND1	RPS27
SCAND2P	RPS27A
SCAP	RPS27L
SCAPER	RPS27P19
SCARA3	RPS27P29

SCARA5	RPS28
SCARB1	RPS29
SCARB2	RPS2P32
SCARF1	RPS2P35
SCARF2	RPS3
SCARNA10	RPS3A
SCARNA12	RPS4X
SCARNA13	RPS4Y1
SCARNA15	RPS4Y2
SCARNA16	RPS5
SCARNA17	RPS6
SCARNA2	RPS6KA1
SCARNA20	RPS6KA2
SCARNA21	RPS6KA3
SCARNA23	RPS6KA4
SCARNA4	RPS6KA5
SCARNA5	RPS6KA6
SCARNA6	RPS6KB1
SCARNA7	RPS6KB2
SCARNA8	RPS6KC1
SCARNA9	RPS6KL1
SCARNA9L	RPS7
SCART1	RPS7P5
SCAT1	RPS8P10
SCAT8	RPS9
SCCPDH	RPSA
SCD	RPSAP52
SCD1	RPTN
SCD2	RPTOR
SCD3	RPTOROS
SCD4	RPUSD1
SCD5	RPUSD2
SCDP1	RPUSD3
SCEL	RPUSD4
SCFD1	RRAD
SCFD2	RRAGA

SCG2	RRAGB
SCG2A	RRAGC
SCG2B	RRAGD
SCG3	RRAS
SCG5	RRAS2
SCGB1A1	RRBP1
SCGB1B2P	RREB1
SCGB1B3	RRH
SCGB1C1	RRM1
SCGB1D2	RRM2
SCGB1D4	RRM2.1.L
SCGB2A1	RRM2B
SCGB2A2	RRN3
SCGB2B12	RRN3P1
SCGB2B19	RRN3P2
SCGB2B20	RRP1
SCGB2B27	RRP12
SCGB2B7	RRP15
SCGB3A1	RRP1B
SCGB3A2	RRP36
SCGN	RRP7A
SCHIP1	RRP7BP
SCIMP	RRP8
SCIN	RRP9
SCIRT	RRS1
SCLT1	RRS1-DT
SCLY	RS1
SCMH1	RS1A
SCML1	RSAD1
SCML2	RSAD2
SCML4	RSBN1
SCN10A	RSBN1L
SCN11A	RSC1A1
SCN1A	RSF1
SCN1A-AS1	RSKR
SCN1B	RSL1D1

SCN2A	RSL24D1
SCN2B	RSPH1
SCN3A	RSPH10B
SCN3B	RSPH10B2
SCN4A	RSPH14
SCN4B	RSPH3
SCN5A	RSPH3A
SCN7A	RSPH4A
SCN8A	RSPH6A
SCN8AA	RSPH9
SCN9A	RSPO1
SCNM1	RSPO2
SCNN1A	RSPO3
SCNN1B	RSPO4
SCNN1D	RSPRY1
SCNN1G	RSRC1
SCO1	RSRC2
SCO2	RSRP1
SCOC	RSS
SCOC-AS1	RSU1
SCP1	RT1-BA
SCP2	RT1-BB
SCPEP1	RT1-CE12
SCRG1	RT1-CE16
SCRIB	RT1-CE5
SCRN1	RT1-CL
SCRN2	RT1-DA
SCRN3	RT1-DB1
SCRT1	RT1-DMA
SCRT2	RT1-DMB
SCT	RT1-EC2
SCTR	RT1-M6-1
SCUBE1	RT1-N3
SCUBE2	RT1-S3
SCUBE3	RTBDN
SCX	RTCA

SCYL1	RTCB
SCYL2	RTEL1
SCYL3	RTEL1-TNFRSF6B
SDAD1	RTF2
SDAD1P1	RTFDC1
SDC1	RTKN
SDC2	RTKN2
SDC2.S	RTL1
SDC3	RTL10
SDC4	RTL3
SDC4P	RTL4
SDCBP	RTL5
SDCBP2	RTL6
SDCBP2-AS1	RTL8A
SDCCAG8	RTL8B
SDE2	RTL8C
SDF2	RTL9
SDF2L1	RTN1
SDF4	RTN1.L
SDHA	RTN2
SDHAF1	RTN2B
SDHAF2	RTN3
SDHAF3	RTN4
SDHAF4	RTN4B
SDHAP1	RTN4IP1
SDHAP2	RTN4R
SDHB	RTN4RL1
SDHB-1	RTN4RL2
SDHC	RTN4RL2A
SDHD	RTN4RL2B
SDHDA	RTP1
SDK1	RTP3
SDK2	RTP4
SDR16C5	RTP5
SDR16C6P	RTRAF
SDR39U1	RTTN

SDR42E1	RUBCN
SDR42E2	RUBCNL
SDR9C7	RUFY1
SDS	RUFY1-AS1
SDSL	RUFY2
SDZ-8	RUFY3
SEBOX	RUFY4
SEC1	RUNDC1
SEC11A	RUNDC3A
SEC11C	RUNDC3AB
SEC13	RUNDC3B
SEC14L1	RUNX1
SEC14L1P1	RUNX1T1
SEC14L2	RUNX2
SEC14L3	RUNX2-AS1
SEC14L4	RUNX3
SEC14L5	RUP2
SEC16A	RUSC1
SEC16B	RUSC1-AS1
SEC1P	RUSC2
SEC22A	RUSF1
SEC22B	RUVBL1
SEC22C	RUVBL2
SEC23A	RWDD1
SEC23B	RWDD2A
SEC23IP	RWDD2B
SEC24A	RWDD3
SEC24B	RWDD4
SEC24B-AS1	RWDD4A
SEC24C	RXFP1
SEC24D	RXFP2
SEC31A	RXFP4
SEC31B	RXRA
SEC61A1	RXRB
SEC61A2	RXRG
SEC61B	RXYLT1

SEC61G	RYBP
SEC62	RYK
SEC63	RYR1
SECISBP2	RYR2
SECISBP2L	RYR3
SECTM1	S100A1
SECTM1A	S100A10
SECTM1B	S100A11
SEH1L	S100A11P1
SEL1L	S100A12
SEL1L3	S100A13
SELE	S100A14
SELENBP1	S100A16
SELENBP2	S100A2
SELENOF	S100A3
SELENOH	S100A4
SELENOI	S100A5
SELENOK	S100A6
SELENOM	S100A7
SELENOO	S100A7A
SELENOP	S100A8
SELENOS	S100A9
SELENOT	S100B
SELENOT2	S100G
SELENOV	S100P
SELENOW	S100PBP
SELENOW1	S100Z
SELENOW2B	S1PR1
SELK	S1PR2
SELL	S1PR3
SELM	S1PR4
SELP	S1PR5
SELPLG	S6K
SEM1	SAA
SEMA3A	SAA1
SEMA3B	SAA2

SEMA3C	SAA3
SEMA3D	SAA4
SEMA3E	SAAL1
SEMA3F	SAC3D1
SEMA3F-AS1	SACM1L
SEMA3G	SACS
SEMA4A	SAE1
SEMA4B	SAFB
SEMA4C	SAFB2
SEMA4D	SAG
SEMA4E	SAGA
SEMA4F	SAGB
SEMA4G	SAGE1
SEMA5A	SAH
SEMA5B	SAI1
SEMA6A	SALL1
SEMA6B	SALL2
SEMA6C	SALL3
SEMA6D	SALL4
SEMA7A	SALRNA3
SEMG1	SALS
SENP1	SAMD1
SENP2	SAMD10
SENP3	SAMD11
SENP3-EIF4A1	SAMD12
SENP5	SAMD13
SENP6	SAMD14
SENP7	SAMD15
SENP8	SAMD3
SEPHS1	SAMD4A
SEPHS2	SAMD4B
SEPN1	SAMD5
SEPSECS	SAMD8
SEPT5-GP1BB	SAMD9
SEPTIN1	SAMD9L
SEPTIN10	SAMHD1

SEPTIN11	SAMM50
SEPTIN12	SAMSN1
SEPTIN14	SANBR
SEPTIN14P20	SAO
SEPTIN2	SAP130
SEPTIN3	SAP18
SEPTIN4	SAP30
SEPTIN5	SAP30BP
SEPTIN6	SAP30L
SEPTIN7	SAP30L-AS1
SEPTIN7P2	SAPCD1
SEPTIN8	SAPCD2
SEPTIN9	SAR1A
SEPTIN9A	SAR1B
SEPTIN9-DT	SARA
SEPW1	SARAF
SERAC1	SARDH
SERBP1	SARM1
SERF1A	SARNP
SERF1B	SARS
SERF2	SARS1
SERF2-C15ORF63	SARS2
SERGEF	SART1
SERHL	SART3
SERHL2	SASH1
SERINC1	SASH3
SERINC2	SASS6
SERINC3	SAT1
SERINC4	SAT2
SERINC5	SATB1
SERP1	SATB2
SERP2	SATB2-AS1
SERPINA1	SATL1
SERPINA10	SAV1
SERPINA11	SAXO1
SERPINA12	SAXO2

SERPINA13P	SAYSD1
SERPINA1B	SBDS
SERPINA1D	SBDSP1
SERPINA1E	SBF1
SERPINA3	SBF1P1
SERPINA3B	SBF2
SERPINA3C	SBF2-AS1
SERPINA3K	SBK1
SERPINA3M	SBK2
SERPINA3N	SBK3
SERPINA4	SBNO1
SERPINA5	SBNO2
SERPINA6	SBPL
SERPINA7	SBSN
SERPINA9	SBSPON
SERPINB1	SC5D
SERPINB10	SCAF1
SERPINB11	SCAF11
SERPINB12	SCAF4
SERPINB13	SCAF8
SERPINB1A	SCAI
SERPINB1B	SCAMP1
SERPINB1C	SCAMP2
SERPINB1L3	SCAMP3
SERPINB2	SCAMP4
SERPINB3	SCAMP5
SERPINB3B	SCAND1
SERPINB3C	SCAND2P
SERPINB4	SCAP
SERPINB5	SCAPER
SERPINB6	SCARA3
SERPINB6A	SCARA5
SERPINB6B	SCARB1
SERPINB6C	SCARB2
SERPINB7	SCARF1
SERPINB8	SCARF2

SERPINB9	SCARNA10
SERPINB9B	SCARNA12
SERPINB9F	SCARNA13
SERPINC1	SCARNA15
SERPIND1	SCARNA16
SERPINE1	SCARNA17
SERPINE2	SCARNA2
SERPINE3	SCARNA20
SERPINF1	SCARNA21
SERPINF2	SCARNA23
SERPING1	SCARNA4
SERPINH1	SCARNA5
SERPINI1	SCARNA6
SERPINI2	SCARNA7
SERT1	SCARNA8
SERTAD1	SCARNA9
SERTAD2	SCARNA9L
SERTAD3	SCART1
SERTAD4	SCAT1
SERTAD4-AS1	SCAT8
SERTM1	SCCPDH
SESN1	SCD
SESN2	SCD1
SESN3	SCD2
SESTD1	SCD3
SET	SCD4
SETBP1	SCD5
SETD1A	SCDP1
SETD1B	SCEL
SETD2	SCFD1
SETD3	SCFD2
SETD4	SCG2
SETD5	SCG2A
SETD6	SCG2B
SETD7	SCG3
SETD9	SCG5

SETDB1	SCGB1A1
SETDB2	SCGB1B2P
SETMAR	SCGB1B3
SETX	SCGB1C1
SEZ6	SCGB1D2
SEZ6L	SCGB1D4
SEZ6L2	SCGB2A1
SF1	SCGB2A2
SF3A1	SCGB2B12
SF3A2	SCGB2B19
SF3A3	SCGB2B20
SF3B1	SCGB2B27
SF3B2	SCGB2B7
SF3B3	SCGB3A1
SF3B4	SCGB3A2
SF3B5	SCGN
SF3B6	SCHIP1
SFI1	SCIMP
SFMBT1	SCIN
SFMBT2	SCIRT
SFN	SCLC1
SFPQ	SCLT1
SFR1	SCLY
SFRP1	SCMH1
SFRP2	SCML1
SFRP4	SCML2
SFRP5	SCML4
SFRS1	SCN10A
SFRS16	SCN11A
SFRS3	SCN1A
SFRS6	SCN1A-AS1
SFSWAP	SCN1B
SFT2D1	SCN2A
SFT2D3	SCN2B
SFTA2	SCN3A
SFTA3	SCN3B

SFTPA1	SCN4A
SFTPA2	SCN4B
SFTPB	SCN5A
SFTPC	SCN7A
SFTPD	SCN8A
SFXN1	SCN8AA
SFXN2	SCN9A
SFXN3	SCNM1
SFXN4	SCNN1A
SFXN5	SCNN1B
SFXN5B	SCNN1D
SGCA	SCNN1G
SGCB	SCO1
SGCD	SCO2
SGCE	SCOC
SGCG	SCOC-AS1
SGF29	SCP1
SGIP1	SCP2
SGK1	SCPEP1
SGK2	SCRG1
SGK3	SCRIB
SGMS1	SCRN1
SGMS2	SCRN2
SGO1	SCRN3
SGO2	SCRT1
SGPL1	SCRT2
SGPP1	SCT
SGPP2	SCTR
SGSH	SCUBE1
SGSM1	SCUBE2
SGSM2	SCUBE3
SGSM3	SCX
SGTA	SCYL1
SGTB	SCYL2
SH2B1	SCYL3
SH2B2	SDAD1

SH2B3	SDAD1P1
SH2D1A	SDC1
SH2D1B	SDC2
SH2D1B1	SDC2.S
SH2D2A	SDC3
SH2D3A	SDC4
SH2D3C	SDC4P
SH2D4A	SDCBP
SH2D4B	SDCBP2
SH2D5	SDCBP2-AS1
SH2D6	SDCCAG8
SH2D7	SDE2
SH3BGR	SDF2
SH3BGRL	SDF2L1
SH3BGRL2	SDF4
SH3BGRL3	SDHA
SH3BP1	SDHAF1
SH3BP2	SDHAF2
SH3BP4	SDHAF3
SH3BP5	SDHAF4
SH3BP5-AS1	SDHAP1
SH3BP5L	SDHAP2
SH3D19	SDHB
SH3D21	SDHB-1
SH3GL1	SDHC
SH3GL2	SDHD
SH3GL3	SDHDA
SH3GLB1	SDK1
SH3GLB2	SDK2
SH3KBP1	SDR16C5
SH3PXD2A	SDR16C6P
SH3PXD2B	SDR39U1
SH3RF1	SDR42E1
SH3RF2	SDR42E2
SH3RF3	SDR9C7
SH3RF3-AS1	SDS

SH3TC1	SDSL
SH3TC2	SDZ-8
SH3YL1	SEA
SHANK1	SEBOX
SHANK2	SEC1
SHANK2-AS1	SEC11A
SHANK3	SEC11C
SHARPIN	SEC13
SHB	SEC14L1
SHC1	SEC14L1P1
SHC1P1	SEC14L2
SHC2	SEC14L3
SHC3	SEC14L4
SHC4	SEC14L5
SHCBP1	SEC16A
SHCBP1L	SEC16B
SHD	SEC1P
SHE	SEC22A
SHF	SEC22B
SHFL	SEC22C
SHFM1	SEC23A
SHH	SEC23B
SHHA	SEC23IP
SHISA2	SEC24A
SHISA3	SEC24B
SHISA4	SEC24B-AS1
SHISA5	SEC24C
SHISA6	SEC24D
SHISA7	SEC31A
SHISA8	SEC31B
SHISA9	SEC61A1
SHISA9A	SEC61A2
SHISAL1	SEC61B
SHISAL2A	SEC61G
SHKBP1	SEC62
SHLD1	SEC63

SHLD2	SECISBP2
SHLD2P1	SECISBP2L
SHLD2P3	SECTM1
SHLD3	SECTM1A
SHMT1	SECTM1B
SHMT2	SEH1L
SHOC1	SEL1L
SHOC2	SEL1L3
SHOX	SELE
SHOX2	SELENBP1
SHPK	SELENBP2
SHPRH	SELENOF
SHQ1	SELENOH
SHROOM1	SELENOI
SHROOM2	SELENOK
SHROOM3	SELENOM
SHROOM4	SELENOO
SHTN1	SELENOP
SI	SELENOS
SIAE	SELENOT
SIAH1	SELENOT2
SIAH1A	SELENOV
SIAH1B	SELENOW
SIAH2	SELENOW1
SIAH3	SELENOW2B
SID4	SELK
SIDT1	SELL
SIDT2	SELM
SIGIRR	SELP
SIGLEC1	SELPLG
SIGLEC10	SEM1
SIGLEC11	SEMA3A
SIGLEC12	SEMA3B
SIGLEC14	SEMA3C
SIGLEC16	SEMA3D
SIGLEC17P	SEMA3E

SIGLEC5	SEMA3F
SIGLEC6	SEMA3F-AS1
SIGLEC7	SEMA3G
SIGLEC8	SEMA4A
SIGLEC9	SEMA4B
SIGLECE	SEMA4C
SIGLECG	SEMA4D
SIGLECH	SEMA4E
SIGLECL1	SEMA4F
SIGMAR1	SEMA4G
SIK1	SEMA5A
SIK2	SEMA5B
SIK3	SEMA6A
SIKE1	SEMA6B
SIL1	SEMA6C
SILC1	SEMA6D
SIM1	SEMA7A
SIM2	SEMG1
SIMA	SENP1
SIMC1	SENP2
SIN3A	SENP3
SIN3B	SENP3-EIF4A1
SINHCAF	SENP5
SIPA1	SENP6
SIPA1L1	SENP7
SIPA1L2	SENP8
SIPA1L3	SEPHS1
SIR-2.1	SEPHS2
SIRPA	SEPN1
SIRPB1	SEPSECS
SIRPB2	SEPT5-GP1BB
SIRPD	SEPTIN1
SIRPG	SEPTIN10
SIRPG-AS1	SEPTIN11
SIRT1	SEPTIN12
SIRT2	SEPTIN14

SIRT3	SEPTIN14P20
SIRT4	SEPTIN2
SIRT5	SEPTIN3
SIRT6	SEPTIN4
SIRT7	SEPTIN5
SIT1	SEPTIN6
SIVA1	SEPTIN7
SIX1	SEPTIN7P2
SIX2	SEPTIN8
SIX3	SEPTIN9
SIX3-AS1	SEPTIN9A
SIX4	SEPTIN9-DT
SIX4A	SEPW1
SIX5	SERAC1
SIX6	SERBP1
SKA1	SERF1A
SKA2	SERF1B
SKA3	SERF2
SKAP1	SERF2-C15ORF63
SKAP2	SERGEF
SKI	SERHL
SKIA	SERHL2
SKIB	SERINC1
SKIC2	SERINC2
SKIC3	SERINC3
SKIC8	SERINC4
SKIDA1	SERINC5
SKIL	SERP1
SKINT5	SERP2
SKINT9	SERPINA1
SKIV2L2	SERPINA10
SKN-1	SERPINA11
SKOR1	SERPINA12
SKOR2	SERPINA13P
SKP1	SERPINA1B
SKP2	SERPINA1D

SLA	SERPINA1E
SLA2	SERPINA3
SLAIN1	SERPINA3B
SLAIN2	SERPINA3C
SLAMF1	SERPINA3K
SLAMF6	SERPINA3M
SLAMF7	SERPINA3N
SLAMF8	SERPINA4
SLAMF9	SERPINA5
SLBP	SERPINA6
SLC10A1	SERPINA7
SLC10A2	SERPINA9
SLC10A3	SERPINB1
SLC10A4	SERPINB10
SLC10A5	SERPINB11
SLC10A6	SERPINB12
SLC10A7	SERPINB13
SLC11A1	SERPINB1A
SLC11A2	SERPINB1B
SLC12A1	SERPINB1C
SLC12A2	SERPINB1L3
SLC12A2-DT	SERPINB2
SLC12A3	SERPINB3
SLC12A4	SERPINB3B
SLC12A5	SERPINB3C
SLC12A6	SERPINB4
SLC12A7	SERPINB5
SLC12A8	SERPINB6
SLC12A9	SERPINB6A
SLC13A1	SERPINB6B
SLC13A2	SERPINB6C
SLC13A3	SERPINB7
SLC13A4	SERPINB8
SLC13A5	SERPINB9
SLC14A1	SERPINB9B
SLC14A2	SERPINB9F

SLC15A1	SERPINC1
SLC15A2	SERPIND1
SLC15A3	SERPINE1
SLC15A4	SERPINE2
SLC16A1	SERPINE3
SLC16A10	SERPINF1
SLC16A11	SERPINF2
SLC16A12	SERPING1
SLC16A13	SERPINH1
SLC16A14	SERPINI1
SLC16A1-AS1	SERPINI2
SLC16A2	SERT1
SLC16A3	SERTAD1
SLC16A4	SERTAD2
SLC16A5	SERTAD3
SLC16A6	SERTAD4
SLC16A6B	SERTAD4-AS1
SLC16A7	SERTM1
SLC16A8	SESN1
SLC16A9	SESN2
SLC16A9A	SESN3
SLC16A9B	SESTD1
SLC17A1	SET
SLC17A2	SETBP1
SLC17A3	SETD1A
SLC17A4	SETD1B
SLC17A5	SETD2
SLC17A6	SETD3
SLC17A7	SETD4
SLC17A8	SETD5
SLC17A9	SETD6
SLC18A1	SETD7
SLC18A2	SETD9
SLC18A3	SETDB1
SLC18B1	SETDB2
SLC19A1	SETMAR

SLC19A2	SETX
SLC19A3	SEZ6
SLC1A1	SEZ6L
SLC1A2	SEZ6L2
SLC1A3	SF1
SLC1A4	SF3A1
SLC1A5	SF3A2
SLC1A6	SF3A3
SLC1A7	SF3B1
SLC1A8B	SF3B2
SLC1A9	SF3B3
SLC20A1	SF3B4
SLC20A2	SF3B5
SLC22A1	SF3B6
SLC22A10	SFI1
SLC22A11	SFMBT1
SLC22A12	SFMBT2
SLC22A13	SFN
SLC22A14	SFPQ
SLC22A15	SFR1
SLC22A16	SFRP1
SLC22A17	SFRP2
SLC22A18	SFRP4
SLC22A18AS	SFRP5
SLC22A19	SFRS1
SLC22A2	SFRS16
SLC22A20	SFRS3
SLC22A21	SFRS6
SLC22A22	SFSWAP
SLC22A23	SFT2D1
SLC22A24	SFT2D3
SLC22A26	SFTA1P
SLC22A27	SFTA2
SLC22A3	SFTA3
SLC22A30	SFTPA1
SLC22A4	SFTPA2

SLC22A5	SFTPB
SLC22A6	SFTPC
SLC22A7	SFTPD
SLC22A7A	SFXN1
SLC22A7B.1	SFXN2
SLC22A8	SFXN3
SLC22A9	SFXN4
SLC23A1	SFXN5
SLC23A2	SFXN5B
SLC23A3	SGCA
SLC24A1	SGCB
SLC24A2	SGCD
SLC24A3	SGCE
SLC24A4	SGCG
SLC24A5	SGF29
SLC25A1	SGIP1
SLC25A10	SGK1
SLC25A11	SGK2
SLC25A12	SGK3
SLC25A13	SGMS1
SLC25A14	SGMS2
SLC25A15	SGO1
SLC25A15B	SGO1-AS1
SLC25A16	SGO2
SLC25A17	SGPL1
SLC25A18	SGPP1
SLC25A19	SGPP2
SLC25A20	SGSH
SLC25A21	SGSM1
SLC25A21-AS1	SGSM2
SLC25A22	SGSM3
SLC25A23	SGTA
SLC25A24	SGTB
SLC25A25	SH2B1
SLC25A25-AS1	SH2B2
SLC25A26	SH2B3

SLC25A27	SH2D1A
SLC25A28	SH2D1B
SLC25A29	SH2D1B1
SLC25A3	SH2D2A
SLC25A30	SH2D3A
SLC25A31	SH2D3C
SLC25A32	SH2D4A
SLC25A32A	SH2D4B
SLC25A33	SH2D5
SLC25A34	SH2D6
SLC25A35	SH2D7
SLC25A36	SH3BGR
SLC25A36L1	SH3BGRL
SLC25A37	SH3BGRL2
SLC25A38	SH3BGRL3
SLC25A38A	SH3BP1
SLC25A39	SH3BP2
SLC25A3P1	SH3BP4
SLC25A4	SH3BP5
SLC25A40	SH3BP5-AS1
SLC25A41	SH3BP5L
SLC25A42	SH3D19
SLC25A43	SH3D21
SLC25A44	SH3GL1
SLC25A45	SH3GL2
SLC25A46	SH3GL3
SLC25A46.S	SH3GLB1
SLC25A47	SH3GLB2
SLC25A48	SH3KBP1
SLC25A5	SH3PXD2A
SLC25A51	SH3PXD2B
SLC25A51P1	SH3RF1
SLC25A52	SH3RF2
SLC25A53	SH3RF3
SLC25A5-AS1	SH3RF3-AS1
SLC25A6	SH3TC1

SLC26A1	SH3TC2
SLC26A10P	SH3YL1
SLC26A11	SHANK1
SLC26A2	SHANK2
SLC26A3	SHANK2-AS1
SLC26A3.2	SHANK3
SLC26A4	SHARPIN
SLC26A5	SHB
SLC26A6	SHC1
SLC26A7	SHC1P1
SLC26A9	SHC2
SLC27A1	SHC3
SLC27A2	SHC4
SLC27A3	SHCBP1
SLC27A4	SHCBP1L
SLC27A5	SHD
SLC27A6	SHE
SLC28A1	SHF
SLC28A2	SHFL
SLC28A3	SHFM1
SLC29A1	SHH
SLC29A2	SHHA
SLC29A3	SHISA2
SLC29A4	SHISA3
SLC29A4P1	SHISA4
SLC2A1	SHISA5
SLC2A10	SHISA6
SLC2A11	SHISA7
SLC2A11B	SHISA8
SLC2A11L	SHISA9
SLC2A12	SHISA9A
SLC2A13	SHISAL1
SLC2A14	SHISAL2A
SLC2A15B	SHKBP1
SLC2A1-DT	SHLD1
SLC2A2	SHLD2

SLC2A3	SHLD2P1
SLC2A4	SHLD2P3
SLC2A4RG	SHLD3
SLC2A4RG-PS	SHMT1
SLC2A5	SHMT2
SLC2A6	SHOC1
SLC2A7	SHOC2
SLC2A8	SHOX
SLC2A9	SHOX2
SLC2A9L2	SHPK
SLC30A1	SHPRH
SLC30A10	SHQ1
SLC30A2	SHROOM1
SLC30A3	SHROOM2
SLC30A4	SHROOM3
SLC30A5	SHROOM4
SLC30A6	SHTN1
SLC30A7	SI
SLC30A8	SIAE
SLC30A9	SIAH1
SLC31A1	SIAH1A
SLC31A2	SIAH1B
SLC32A1	SIAH2
SLC33A1	SIAH3
SLC34A1	SID4
SLC34A2	SIDT1
SLC34A3	SIDT2
SLC35A1	SIGIRR
SLC35A2	SIGLEC1
SLC35A3	SIGLEC10
SLC35A4	SIGLEC11
SLC35A5	SIGLEC12
SLC35B1	SIGLEC14
SLC35B2	SIGLEC16
SLC35B3	SIGLEC17P
SLC35B4	SIGLEC5

SLC35C1	SIGLEC6
SLC35C2	SIGLEC7
SLC35D1	SIGLEC8
SLC35D2	SIGLEC9
SLC35D3	SIGLECE
SLC35E1	SIGLECG
SLC35E2	SIGLECH
SLC35E2A	SIGLECL1
SLC35E2B	SIGMAR1
SLC35E3	SIK1
SLC35E4	SIK1B
SLC35F1	SIK2
SLC35F2	SIK3
SLC35F3	SIKE1
SLC35F4	SIL1
SLC35F5	SILC1
SLC35F6	SIM1
SLC35G1	SIM2
SLC35G2	SIMA
SLC35G3	SIMC1
SLC35G5	SIN3A
SLC35G6	SIN3B
SLC36A1	SINHCAF
SLC36A2	SIPA1
SLC36A3	SIPA1L1
SLC36A4	SIPA1L2
SLC37A1	SIPA1L3
SLC37A2	SIR-2.1
SLC37A3	SIRPA
SLC37A4	SIRPB1
SLC38A1	SIRPB2
SLC38A10	SIRPD
SLC38A11	SIRPG
SLC38A2	SIRPG-AS1
SLC38A3	SIRT1
SLC38A4	SIRT2

SLC38A5	SIRT3
SLC38A6	SIRT4
SLC38A7	SIRT5
SLC38A8	SIRT6
SLC38A9	SIRT7
SLC39A1	SIT1
SLC39A10	SIVA1
SLC39A11	SIX1
SLC39A12	SIX2
SLC39A13	SIX3
SLC39A14	SIX3-AS1
SLC39A2	SIX4
SLC39A3	SIX4A
SLC39A4	SIX5
SLC39A5	SIX6
SLC39A6	SKA1
SLC39A7	SKA2
SLC39A8	SKA3
SLC39A9	SKAP1
SLC3A1	SKAP2
SLC3A2	SKI
SLC40A1	SKIA
SLC41A1	SKIB
SLC41A2	SKIC2
SLC41A3	SKIC3
SLC43A1	SKIC8
SLC43A1A	SKIDA1
SLC43A2	SKIL
SLC43A2B	SKINT5
SLC43A3	SKINT9
SLC44A1	SKIV2L2
SLC44A2	SKN-1
SLC44A3	SKOR1
SLC44A4	SKOR2
SLC44A5	SKP1
SLC45A1	SKP2

SLC45A2	SLA
SLC45A3	SLA2
SLC45A4	SLAIN1
SLC46A1	SLAIN2
SLC46A2	SLAMF1
SLC46A3	SLAMF6
SLC47A1	SLAMF7
SLC47A2	SLAMF8
SLC48A1	SLAMF9
SLC49A3	SLBP
SLC49A4	SLC10A1
SLC4A1	SLC10A2
SLC4A10	SLC10A3
SLC4A11	SLC10A4
SLC4A1AP	SLC10A5
SLC4A2	SLC10A6
SLC4A3	SLC10A7
SLC4A4	SLC11A1
SLC4A5	SLC11A2
SLC4A7	SLC12A1
SLC4A8	SLC12A2
SLC50A1	SLC12A2-DT
SLC51A	SLC12A3
SLC51B	SLC12A4
SLC52A1	SLC12A5
SLC52A2	SLC12A6
SLC52A3	SLC12A7
SLC5A1	SLC12A8
SLC5A10	SLC12A9
SLC5A11	SLC13A1
SLC5A12	SLC13A2
SLC5A2	SLC13A3
SLC5A3	SLC13A4
SLC5A4	SLC13A5
SLC5A4B	SLC14A1
SLC5A5	SLC14A2

SLC5A6	SLC15A1
SLC5A7	SLC15A2
SLC5A8	SLC15A3
SLC5A9	SLC15A4
SLC66A1	SLC16A1
SLC66A1L	SLC16A10
SLC66A2	SLC16A11
SLC66A3	SLC16A12
SLC6A1	SLC16A13
SLC6A10P	SLC16A14
SLC6A10PB	SLC16A1-AS1
SLC6A11	SLC16A2
SLC6A12	SLC16A3
SLC6A13	SLC16A4
SLC6A14	SLC16A5
SLC6A15	SLC16A6
SLC6A16	SLC16A6B
SLC6A17	SLC16A7
SLC6A18	SLC16A8
SLC6A19	SLC16A9
SLC6A19A.1	SLC16A9A
SLC6A2	SLC16A9B
SLC6A20	SLC17A1
SLC6A20A	SLC17A2
SLC6A3	SLC17A3
SLC6A4	SLC17A4
SLC6A5	SLC17A5
SLC6A6	SLC17A6
SLC6A7	SLC17A7
SLC6A8	SLC17A8
SLC6A9	SLC17A9
SLC7A1	SLC18A1
SLC7A10	SLC18A2
SLC7A11	SLC18A3
SLC7A11-AS1	SLC18B1
SLC7A12	SLC19A1

SLC7A13	SLC19A2
SLC7A14	SLC19A3
SLC7A2	SLC1A1
SLC7A3	SLC1A2
SLC7A4	SLC1A3
SLC7A5	SLC1A4
SLC7A5P1	SLC1A5
SLC7A5P2	SLC1A6
SLC7A6	SLC1A7
SLC7A6OS	SLC1A8B
SLC7A7	SLC1A9
SLC7A8	SLC20A1
SLC7A9	SLC20A2
SLC8A1	SLC22A1
SLC8A2	SLC22A10
SLC8A3	SLC22A11
SLC8B1	SLC22A12
SLC9A1	SLC22A13
SLC9A2	SLC22A14
SLC9A3	SLC22A15
SLC9A3R1	SLC22A16
SLC9A3R2	SLC22A17
SLC9A4	SLC22A18
SLC9A5	SLC22A18AS
SLC9A6	SLC22A19
SLC9A7	SLC22A2
SLC9A8	SLC22A20
SLC9A9	SLC22A21
SLC9B1	SLC22A22
SLC9B2	SLC22A23
SLC9C1	SLC22A24
SLC9C2	SLC22A26
SLCO1A1	SLC22A27
SLCO1A2	SLC22A3
SLCO1A4	SLC22A30
SLCO1B1	SLC22A4

SLCO1B2	SLC22A5
SLCO1B3	SLC22A6
SLCO1B7	SLC22A7
SLCO1C1	SLC22A7A
SLCO1D1	SLC22A7B.1
SLCO2A1	SLC22A8
SLCO2B1	SLC22A9
SLCO3A1	SLC23A1
SLCO4A1	SLC23A2
SLCO4A1-AS1	SLC23A3
SLCO4C1	SLC24A1
SLCO5A1	SLC24A2
SLCO6A1	SLC24A3
SLF1	SLC24A4
SLF2	SLC24A5
SLFN1	SLC25A1
SLFN11	SLC25A10
SLFN12	SLC25A11
SLFN12L	SLC25A12
SLFN13	SLC25A13
SLFN2	SLC25A14
SLFN3	SLC25A15
SLFN4	SLC25A15B
SLFN5	SLC25A16
SLFN8	SLC25A17
SLFNL1	SLC25A18
SLIRP	SLC25A19
SLIT1	SLC25A20
SLIT2	SLC25A21
SLIT3	SLC25A21-AS1
SLITRK1	SLC25A22
SLITRK2	SLC25A23
SLITRK3	SLC25A24
SLITRK4	SLC25A25
SLITRK5	SLC25A25-AS1
SLITRK6	SLC25A26

SLK	SLC25A27
SLMAP	SLC25A28
SLN	SLC25A29
SLPI	SLC25A3
SLPR	SLC25A30
SLTM	SLC25A31
SLU7	SLC25A32
SLUG	SLC25A32A
SLURP1	SLC25A33
SLURP1L.S	SLC25A34
SLX1A	SLC25A35
SLX1B	SLC25A36
SLX4	SLC25A36L1
SLX4IP	SLC25A37
SLX9	SLC25A38
SMAD1	SLC25A38A
SMAD2	SLC25A39
SMAD3	SLC25A3P1
SMAD4	SLC25A4
SMAD5	SLC25A40
SMAD5-AS1	SLC25A41
SMAD6	SLC25A42
SMAD7	SLC25A43
SMAD9	SLC25A44
SMAGP	SLC25A45
SMAP1	SLC25A46
SMAP2	SLC25A46.S
SMARCA1	SLC25A47
SMARCA2	SLC25A48
SMARCA4	SLC25A5
SMARCA5	SLC25A51
SMARCAD1	SLC25A51P1
SMARCAL1	SLC25A52
SMARCB1	SLC25A53
SMARCC1	SLC25A5-AS1
SMARCC2	SLC25A6

SMARCD1	SLC26A1
SMARCD2	SLC26A10P
SMARCD3	SLC26A11
SMARCE1	SLC26A2
SMC1A	SLC26A3
SMC1B	SLC26A3.2
SMC2	SLC26A4
SMC2-DT	SLC26A5
SMC3	SLC26A6
SMC4	SLC26A7
SMC5	SLC26A9
SMC6	SLC27A1
SMCHD1	SLC27A2
SMCO1	SLC27A3
SMCO3	SLC27A4
SMCO4	SLC27A5
SMCR2	SLC27A6
SMCR5	SLC28A1
SMCR8	SLC28A2
SMDT1	SLC28A3
SMEK1	SLC29A1
SMEK2	SLC29A2
SMG1	SLC29A3
SMG1P5	SLC29A4
SMG1P7	SLC29A4P1
SMG5	SLC2A1
SMG6	SLC2A10
SMG7	SLC2A11
SMG7-AS1	SLC2A11B
SMG8	SLC2A11L
SMG9	SLC2A12
SMGC	SLC2A13
SMIM1	SLC2A14
SMIM10	SLC2A15B
SMIM10L2A	SLC2A1-DT
SMIM10L2B	SLC2A2

SMIM11	SLC2A3
SMIM12	SLC2A4
SMIM13	SLC2A4RG
SMIM14	SLC2A4RG-PS
SMIM15	SLC2A5
SMIM17	SLC2A6
SMIM19	SLC2A7
SMIM2	SLC2A8
SMIM20	SLC2A9
SMIM22	SLC2A9L2
SMIM24	SLC30A1
SMIM26	SLC30A10
SMIM27	SLC30A2
SMIM29	SLC30A3
SMIM2-IT1	SLC30A4
SMIM3	SLC30A5
SMIM30	SLC30A6
SMIM31	SLC30A7
SMIM32	SLC30A8
SMIM34	SLC30A9
SMIM35	SLC31A1
SMIM4	SLC31A2
SMIM43	SLC32A1
SMIM45	SLC33A1
SMIM5	SLC34A1
SMIM6	SLC34A2
SMIM7	SLC34A3
SMIM8	SLC35A1
SMKR1	SLC35A2
SMN1	SLC35A3
SMN2	SLC35A4
SMNDC1	SLC35A5
SMO	SLC35B1
SMOC1	SLC35B2
SMOC2	SLC35B3
SMOX	SLC35B4

SMP-30	SLC35C1
SMPD1	SLC35C2
SMPD2	SLC35D1
SMPD3	SLC35D2
SMPD4	SLC35D3
SMPDL3A	SLC35E1
SMPDL3B	SLC35E2
SMPX	SLC35E2A
SMR3A	SLC35E2B
SMS	SLC35E3
SMTN	SLC35E4
SMTNL1	SLC35F1
SMTNL2	SLC35F2
SMU1	SLC35F3
SMUG1	SLC35F4
SMURF1	SLC35F5
SMURF2	SLC35F6
SMYD1	SLC35G1
SMYD2	SLC35G2
SMYD2A	SLC35G3
SMYD3	SLC35G5
SMYD4	SLC35G6
SMYD5	SLC36A1
SN	SLC36A2
SNAI1	SLC36A3
SNAI2	SLC36A4
SNAI3	SLC37A1
SNAI3-AS1	SLC37A2
SNAP23	SLC37A3
SNAP25	SLC37A4
SNAP25A	SLC38A1
SNAP25-AS1	SLC38A10
SNAP29	SLC38A11
SNAP47	SLC38A2
SNAP91	SLC38A3
SNAPC1	SLC38A4

SNAPC2	SLC38A5
SNAPC3	SLC38A6
SNAPC4	SLC38A7
SNAPC5	SLC38A8
SNAPIN	SLC38A9
SNAR-A1	SLC39A1
SNAR-A3	SLC39A10
SNAR-B2	SLC39A11
SNAR-D	SLC39A12
SNAR-F	SLC39A13
SNAR-G1	SLC39A14
SNAR-G2	SLC39A2
SNAR-H	SLC39A3
SNAR-I	SLC39A4
SNCA	SLC39A5
SNCAIP	SLC39A6
SNCB	SLC39A7
SNCG	SLC39A8
SNCGB	SLC39A9
SND1	SLC3A1
SND1-IT1	SLC3A2
SNED1	SLC40A1
SNF8	SLC41A1
SNHG1	SLC41A2
SNHG10	SLC41A3
SNHG11	SLC43A1
SNHG12	SLC43A1A
SNHG14	SLC43A2
SNHG15	SLC43A2B
SNHG16	SLC43A3
SNHG17	SLC44A1
SNHG19	SLC44A2
SNHG21	SLC44A3
SNHG26	SLC44A4
SNHG28	SLC44A5
SNHG29	SLC45A1

SNHG3	SLC45A2
SNHG32	SLC45A3
SNHG4	SLC45A4
SNHG5	SLC46A1
SNHG6	SLC46A2
SNHG7	SLC46A3
SNHG8	SLC47A1
SNHG9	SLC47A2
SNIP1	SLC48A1
SNL	SLC49A3
SNN	SLC49A4
SNN.S	SLC4A1
SNORA1	SLC4A10
SNORA10	SLC4A11
SNORA11D	SLC4A1AP
SNORA12	SLC4A2
SNORA13	SLC4A3
SNORA14A	SLC4A4
SNORA16A	SLC4A5
SNORA17	SLC4A7
SNORA19	SLC4A8
SNORA20	SLC50A1
SNORA21	SLC51A
SNORA22	SLC51B
SNORA23	SLC52A1
SNORA24	SLC52A2
SNORA25	SLC52A3
SNORA27	SLC5A1
SNORA28	SLC5A10
SNORA29	SLC5A11
SNORA2A	SLC5A12
SNORA3	SLC5A2
SNORA31	SLC5A3
SNORA33	SLC5A4
SNORA34	SLC5A4B
SNORA35	SLC5A5

SNORA36A	SLC5A6
SNORA37	SLC5A7
SNORA38	SLC5A8
SNORA38B	SLC5A9
SNORA3B	SLC66A1
SNORA4	SLC66A1L
SNORA40	SLC66A2
SNORA41	SLC66A3
SNORA43	SLC6A1
SNORA44	SLC6A10P
SNORA46	SLC6A10PB
SNORA48	SLC6A11
SNORA49	SLC6A12
SNORA50A	SLC6A13
SNORA50C	SLC6A14
SNORA52	SLC6A15
SNORA53	SLC6A16
SNORA54	SLC6A17
SNORA55	SLC6A18
SNORA56	SLC6A19
SNORA59A	SLC6A19A.1
SNORA59B	SLC6A2
SNORA5A	SLC6A20
SNORA5C	SLC6A20A
SNORA6	SLC6A3
SNORA60	SLC6A4
SNORA61	SLC6A5
SNORA62	SLC6A6
SNORA63	SLC6A7
SNORA64	SLC6A8
SNORA65	SLC6A9
SNORA66	SLC7A1
SNORA68	SLC7A10
SNORA70	SLC7A11
SNORA71A	SLC7A11-AS1
SNORA71B	SLC7A12

SNORA71C	SLC7A13
SNORA71D	SLC7A14
SNORA72	SLC7A2
SNORA73A	SLC7A3
SNORA73B	SLC7A4
SNORA74A	SLC7A5
SNORA75	SLC7A5P1
SNORA77	SLC7A5P2
SNORA79	SLC7A6
SNORA80A	SLC7A6OS
SNORA80B	SLC7A7
SNORA81	SLC7A8
SNORA84	SLC7A9
SNORA9	SLC8A1
SNORC	SLC8A2
SNORD10	SLC8A3
SNORD100	SLC8B1
SNORD101	SLC9A1
SNORD103A	SLC9A2
SNORD103C	SLC9A3
SNORD104	SLC9A3R1
SNORD105B	SLC9A3R2
SNORD11	SLC9A4
SNORD110	SLC9A5
SNORD111	SLC9A6
SNORD113-4	SLC9A7
SNORD113-5	SLC9A8
SNORD113-6	SLC9A9
SNORD113-7	SLC9B1
SNORD114-3	SLC9B2
SNORD114-6	SLC9C1
SNORD115-1	SLC9C2
SNORD115-10	SLCO1A1
SNORD115-11	SLCO1A2
SNORD115-12	SLCO1A4
SNORD115-14	SLCO1B1

SNORD115-16	SLCO1B2
SNORD115-2	SLCO1B3
SNORD115-22	SLCO1B7
SNORD115-23	SLCO1C1
SNORD115-24	SLCO1D1
SNORD115-25	SLCO2A1
SNORD115-26	SLCO2B1
SNORD115-27	SLCO3A1
SNORD115-28	SLCO4A1
SNORD115-29	SLCO4A1-AS1
SNORD115-3	SLCO4C1
SNORD115-30	SLCO5A1
SNORD115-35	SLCO6A1
SNORD115-36	SLF1
SNORD115-37	SLF2
SNORD115-38	SLFN1
SNORD115-39	SLFN11
SNORD115-4	SLFN12
SNORD115-40	SLFN12L
SNORD115-41	SLFN13
SNORD115-42	SLFN2
SNORD115-43	SLFN3
SNORD115-44	SLFN4
SNORD115-5	SLFN5
SNORD115-6	SLFN8
SNORD115-7	SLFNL1
SNORD115-8	SLIRP
SNORD115-9	SLIT1
SNORD116@	SLIT2
SNORD116-1	SLIT3
SNORD116-10	SLITRK1
SNORD116-11	SLITRK2
SNORD116-19	SLITRK3
SNORD116-20	SLITRK4
SNORD116-21	SLITRK5
SNORD116-22	SLITRK6

SNORD116-23	SLK
SNORD116-24	SLMAP
SNORD116-28	SLN
SNORD116-29	SLPI
SNORD116-4	SLPR
SNORD116-6	SLTM
SNORD116-8	SLU7
SNORD117	SLUG
SNORD11B	SLURP1
SNORD12	SLURP1L.S
SNORD123	SLX1A
SNORD124	SLX1B
SNORD126	SLX4
SNORD12B	SLX4IP
SNORD12C	SLX9
SNORD139	SMAD1
SNORD14C	SMAD2
SNORD14E	SMAD3
SNORD15A	SMAD4
SNORD15B	SMAD5
SNORD16	SMAD5-AS1
SNORD17	SMAD6
SNORD19	SMAD7
SNORD1B	SMAD9
SNORD1C	SMAGP
SNORD21	SMAP1
SNORD22	SMAP2
SNORD23	SMARCA1
SNORD25	SMARCA2
SNORD26	SMARCA4
SNORD28	SMARCA5
SNORD30	SMARCAD1
SNORD32A	SMARCAL1
SNORD32B	SMARCB1
SNORD33	SMARCC1
SNORD34	SMARCC2

SNORD35A	SMARCD1
SNORD35B	SMARCD2
SNORD37	SMARCD3
SNORD38A	SMARCE1
SNORD38B	SMC1A
SNORD3A	SMC1B
SNORD3B-1	SMC2
SNORD3C	SMC2-DT
SNORD41	SMC3
SNORD42B	SMC4
SNORD43	SMC5
SNORD44	SMC6
SNORD45A	SMCHD1
SNORD45B	SMCO1
SNORD45C	SMCO3
SNORD46	SMCO4
SNORD47	SMCR2
SNORD49A	SMCR5
SNORD4A	SMCR8
SNORD4B	SMDT1
SNORD5	SMEK1
SNORD50A	SMEK2
SNORD50B	SMG1
SNORD51	SMG1P5
SNORD54	SMG1P7
SNORD55	SMG5
SNORD56	SMG6
SNORD56B	SMG7
SNORD57	SMG7-AS1
SNORD58C	SMG8
SNORD59A	SMG9
SNORD60	SMGC
SNORD61	SMIM1
SNORD62A	SMIM10
SNORD62B	SMIM10L2A
SNORD63	SMIM10L2B

SNORD64	SMIM11
SNORD65	SMIM12
SNORD66	SMIM13
SNORD67	SMIM14
SNORD68	SMIM15
SNORD69	SMIM17
SNORD70	SMIM19
SNORD71	SMIM2
SNORD73A	SMIM20
SNORD74	SMIM22
SNORD75	SMIM24
SNORD76	SMIM26
SNORD77	SMIM27
SNORD78	SMIM29
SNORD79	SMIM2-IT1
SNORD8	SMIM3
SNORD80	SMIM30
SNORD81	SMIM31
SNORD82	SMIM32
SNORD83A	SMIM34
SNORD83B	SMIM35
SNORD84	SMIM4
SNORD87	SMIM43
SNORD89	SMIM45
SNORD91A	SMIM5
SNORD91B	SMIM6
SNORD93	SMIM7
SNORD94	SMIM8
SNORD95	SMKR1
SNORD96A	SMN1
SNORD97	SMN2
SNORD99	SMNDC1
SNPH	SMO
SNRK	SMOC1
SNRNP200	SMOC2
SNRNP25	SMOX

SNRNP27	SMP-30
SNRNP35	SMPD1
SNRNP40	SMPD2
SNRNP48	SMPD3
SNRNP70	SMPD4
SNRPA	SMPDL3A
SNRPA1	SMPDL3B
SNRPB	SMPX
SNRPB2	SMR3A
SNRPC	SMR3B
SNRPD1	SMS
SNRPD2	SMTN
SNRPD3	SMTNL1
SNRPE	SMTNL2
SNRPF	SMU1
SNRPF-DT	SMUG1
SNRPG	SMURF1
SNRPN	SMURF2
SNTA1	SMYD1
SNTB1	SMYD2
SNTB2	SMYD2A
SNTG1	SMYD3
SNTG2	SMYD4
SNTN	SMYD5
SNU13	SN
SNUPN	SNAI1
SNURF	SNAI2
SNW1	SNAI3
SNX1	SNAI3-AS1
SNX10	SNAP23
SNX11	SNAP25
SNX12	SNAP25A
SNX13	SNAP25-AS1
SNX14	SNAP29
SNX15	SNAP47
SNX16	SNAP91

SNX17	SNAPC1
SNX18	SNAPC2
SNX19	SNAPC3
SNX2	SNAPC4
SNX20	SNAPC5
SNX21	SNAPIN
SNX22	SNAR-A1
SNX24	SNAR-A3
SNX25	SNAR-B2
SNX27	SNAR-D
SNX27A	SNAR-F
SNX29	SNAR-G1
SNX29P2	SNAR-G2
SNX3	SNAR-H
SNX30	SNAR-I
SNX31	SNCA
SNX33	SNCAIP
SNX4	SNCB
SNX5	SNCG
SNX6	SNCGB
SNX7	SND1
SNX8	SND1-IT1
SNX9	SNED1
SOAT1	SNF8
SOAT2	SNHG1
SOBP	SNHG10
SOCS1	SNHG11
SOCS1A	SNHG12
SOCS2	SNHG14
SOCS3	SNHG15
SOCS4	SNHG16
SOCS5	SNHG17
SOCS6	SNHG19
SOCS7	SNHG20
SOD	SNHG21
SOD1	SNHG26

SOD-1	SNHG28
SOD2	SNHG29
SOD3	SNHG3
SOD-3	SNHG32
SODH-1	SNHG4
SOGA1	SNHG5
SOGA2	SNHG6
SOGA3	SNHG7
SOHLH1	SNHG8
SOHLH2	SNHG9
SON	SNIP1
SORBS1	SNL
SORBS2	SNN
SORBS3	SNN.S
SORCS1	SNORA1
SORCS2	SNORA10
SORCS3	SNORA11D
SORD	SNORA12
SORD2P	SNORA13
SORL1	SNORA14A
SORT1	SNORA16A
SOS1	SNORA17
SOS2	SNORA19
SOST	SNORA20
SOSTDC1	SNORA21
SOUL5L	SNORA22
SOWAHA	SNORA23
SOWAHB	SNORA24
SOWAHC	SNORA25
SOWAHD	SNORA27
SOX1	SNORA28
SOX10	SNORA29
SOX11	SNORA2A
SOX12	SNORA3
SOX13	SNORA31
SOX14	SNORA33

SOX15	SNORA34
SOX17	SNORA35
SOX17B.1.S	SNORA36A
SOX18	SNORA37
SOX19A	SNORA38
SOX19B	SNORA38B
SOX1A	SNORA3A
SOX2	SNORA3B
SOX21	SNORA4
SOX21A	SNORA40
SOX21-AS1	SNORA41
SOX21B	SNORA43
SOX2OT	SNORA44
SOX2-OT	SNORA46
SOX3	SNORA48
SOX30	SNORA49
SOX4	SNORA50A
SOX5	SNORA50C
SOX6	SNORA52
SOX6OS	SNORA53
SOX7	SNORA54
SOX8	SNORA55
SOX9	SNORA56
SOX9A	SNORA59A
SOX9B	SNORA59B
SP1	SNORA5A
SP100	SNORA5C
SP110	SNORA6
SP140	SNORA60
SP140L	SNORA61
SP2	SNORA62
SP2-AS1	SNORA63
SP2-DT	SNORA64
SP3	SNORA65
SP4	SNORA66
SP5	SNORA68

SP5L	SNORA70
SP6	SNORA71A
SP7	SNORA71B
SP8	SNORA71C
SP9	SNORA71D
SPA17	SNORA72
SPAAR	SNORA73A
SPACA4	SNORA73B
SPACA6	SNORA74A
SPACA9	SNORA75
SPACDR	SNORA77
SPAG1	SNORA78
SPAG11A	SNORA79
SPAG11B	SNORA80A
SPAG16	SNORA80B
SPAG17	SNORA80E
SPAG4	SNORA81
SPAG5	SNORA84
SPAG5-AS1	SNORA9
SPAG6	SNORC
SPAG7	SNORD10
SPAG8	SNORD100
SPAG9	SNORD101
SPANXA1	SNORD103A
SPANXA2-OT1	SNORD103C
SPANXC	SNORD104
SPANXD	SNORD105B
SPANXN1	SNORD11
SPANXN2	SNORD110
SPANXN4	SNORD111
SPANXN5	SNORD113-4
SPARC	SNORD113-5
SPARCL1	SNORD113-6
SPART	SNORD113-7
SPAST	SNORD114-3
SPATA12	SNORD114-6

SPATA13	SNORD115-1
SPATA16	SNORD115-10
SPATA17	SNORD115-11
SPATA18	SNORD115-12
SPATA19	SNORD115-14
SPATA2	SNORD115-16
SPATA20	SNORD115-2
SPATA22	SNORD115-22
SPATA24	SNORD115-23
SPATA25	SNORD115-24
SPATA2L	SNORD115-25
SPATA3	SNORD115-26
SPATA31A1	SNORD115-27
SPATA31C1	SNORD115-28
SPATA31C2	SNORD115-29
SPATA31D1	SNORD115-3
SPATA31D5P	SNORD115-30
SPATA31E1	SNORD115-35
SPATA32	SNORD115-36
SPATA33	SNORD115-37
SPATA4	SNORD115-38
SPATA41	SNORD115-39
SPATA45	SNORD115-4
SPATA48	SNORD115-40
SPATA5	SNORD115-41
SPATA5L1	SNORD115-42
SPATA6	SNORD115-43
SPATA6L	SNORD115-44
SPATA7	SNORD115-5
SPATA8	SNORD115-6
SPATC1	SNORD115-7
SPATC1L	SNORD115-8
SPATS1	SNORD115-9
SPATS2	SNORD116@
SPATS2L	SNORD116-1
SPC24	SNORD116-10

SPC25	SNORD116-11
SPCH-1	SNORD116-19
SPCS2	SNORD116-20
SPCS2P1	SNORD116-21
SPCS3	SNORD116-22
SPDEF	SNORD116-23
SPDL1	SNORD116-24
SPDYA	SNORD116-28
SPDYC	SNORD116-29
SPDYE1	SNORD116-4
SPDYE17	SNORD116-6
SPDYE2	SNORD116-8
SPDYE21	SNORD117
SPDYE2B	SNORD118
SPDYE4	SNORD11B
SPDYE5	SNORD12
SPDYE7P	SNORD123
SPDYE8	SNORD124
SPECC1	SNORD126
SPECC1L	SNORD12B
SPECC1LB	SNORD12C
SPEER2	SNORD138
SPEER4D	SNORD139
SPEER4F1	SNORD14B
SPEER8-PS1	SNORD14C
SPEF1	SNORD14D
SPEF2	SNORD14E
SPEG	SNORD15A
SPEM1	SNORD15B
SPEM2	SNORD16
SPEN	SNORD17
SPESP1	SNORD19
SPG11	SNORD1B
SPG20	SNORD1C
SPG21	SNORD21
SPG7	SNORD22

SPHK1	SNORD23
SPHK2	SNORD25
SPHKAP	SNORD26
SPI1	SNORD28
SPIA1	SNORD30
SPIB	SNORD32A
SPIC	SNORD32B
SPICE1	SNORD33
SPIDR	SNORD34
SPIN	SNORD35A
SPIN1	SNORD35B
SPIN2A	SNORD37
SPIN2B	SNORD38A
SPIN2D	SNORD38B
SPIN3	SNORD3A
SPIN4	SNORD3B-1
SPINDOC	SNORD3C
SPINK1	SNORD41
SPINK12	SNORD42B
SPINK13	SNORD43
SPINK2	SNORD44
SPINK4	SNORD45A
SPINK5	SNORD45B
SPINK8	SNORD45C
SPINT1	SNORD46
SPINT2	SNORD47
SPINT4	SNORD49A
SPIRE1	SNORD4A
SPIRE2	SNORD4B
SPN	SNORD5
SPN42DD	SNORD50A
SPNS1	SNORD50B
SPNS2	SNORD51
SPNS3	SNORD54
SPO11	SNORD55
SPOCD1	SNORD56

SPOCK1	SNORD56B
SPOCK2	SNORD57
SPOCK3	SNORD58C
SPON1	SNORD59A
SPON1A	SNORD60
SPON1B	SNORD61
SPON2	SNORD62A
SPOP	SNORD62B
SPOPL	SNORD63
SPOUT1	SNORD64
SPP1	SNORD65
SPP2	SNORD66
SPPL2A	SNORD67
SPPL2B	SNORD68
SPPL2C	SNORD69
SPPL3	SNORD70
SPR	SNORD71
SPRED1	SNORD73A
SPRED2	SNORD74
SPRED3	SNORD75
SPRI	SNORD76
SPRING1	SNORD77
SPRN	SNORD78
SPRNP1	SNORD79
SPRR1A	SNORD8
SPRR1B	SNORD80
SPRR2A	SNORD81
SPRR2B	SNORD82
SPRR2C	SNORD83A
SPRR2D	SNORD83B
SPRR2E	SNORD84
SPRR2F	SNORD87
SPRR2G	SNORD89
SPRR2H	SNORD91A
SPRR2I	SNORD91B
SPRR2J-PS	SNORD93

SPRR2K	SNORD94
SPRR3	SNORD95
SPRR4	SNORD96A
SPRTN	SNORD97
SPRY1	SNORD99
SPRY2	SNPH
SPRY3	SNRK
SPRY4	SNRNP200
SPRYD3	SNRNP25
SPRYD4	SNRNP27
SPRYD7	SNRNP35
SPSB1	SNRNP40
SPSB2	SNRNP48
SPSB3	SNRNP70
SPSB4	SNRPA
SPT1	SNRPA1
SPTA1	SNRPB
SPTAN1	SNRPB2
SPTB	SNRPC
SPTBN1	SNRPD1
SPTBN2	SNRPD2
SPTBN4	SNRPD3
SPTBN5	SNRPE
SPTLC1	SNRPF
SPTLC2	SNRPF-DT
SPTLC3	SNRPG
SPTSSA	SNRPN
SPTSSB	SNTA1
SPTY2D1	SNTB1
SPTY2D1OS	SNTB2
SPX	SNTG1
SPZ1	SNTG2
SQLE	SNTN
SQOR	SNU13
SQSTM1	SNUPN
SRA1	SNURF

SRARP	SNW1
SRBD1	SNX1
SRC	SNX10
SRCAP	SNX11
SRCIN1	SNX12
SRD	SNX13
SRD5A1	SNX14
SRD5A2	SNX15
SRD5A3	SNX16
SRD5A3-AS1	SNX17
SREBF1	SNX18
SREBF2	SNX19
SREK1	SNX2
SREK1IP1	SNX20
SRF	SNX21
SRFBP1	SNX22
SRGAP1	SNX24
SRGAP2	SNX25
SRGAP2B	SNX27
SRGAP2C	SNX27A
SRGAP3	SNX29
SRGAP3-AS2	SNX29P2
SRGAP3-AS3	SNX3
SRGAP3-AS4	SNX30
SRGN	SNX31
SRI	SNX33
SRL	SNX4
SRM	SNX5
SRMS	SNX6
SRP14	SNX7
SRP14-DT	SNX8
SRP19	SNX9
SRP54	SOAT1
SRP54A	SOAT2
SRP54C	SOBP
SRP68	SOCS1

SRP72	SOCS1A
SRP9	SOCS2
SRPK1	SOCS3
SRPK2	SOCS4
SRPK3	SOCS5
SRPR	SOCS6
SRPRA	SOCS7
SRPRB	SOD
SRPX	SOD1
SRPX2	SOD-1
SRR	SOD2
SRRD	SOD3
SRRM1	SOD-3
SRRM2	SODH-1
SRRM2-AS1	SOGA1
SRRM3	SOGA2
SRRM4	SOGA3
SRRM5	SOHLH1
SRRT	SOHLH2
SRSF1	SON
SRSF10	SORBS1
SRSF11	SORBS2
SRSF12	SORBS3
SRSF2	SORCS1
SRSF3	SORCS2
SRSF4	SORCS3
SRSF5	SORD
SRSF6	SORD2P
SRSF7	SORL1
SRSF8	SORT1
SRSF9	SOS1
SRST	SOS2
SRXN1	SOST
SS18	SOSTDC1
SS18L1	SOUL5L
SS18L2	SOWAHA

SSB	SOWAHB
SSBP1	SOWAHC
SSBP2	SOWAHD
SSBP3	SOX1
SSBP3-AS1	SOX10
SSBP4	SOX11
SSC4D	SOX12
SSC5D	SOX13
SSH1	SOX14
SSH2	SOX15
SSH3	SOX17
SSL	SOX17B.1.S
SSNA1	SOX18
SSP-10	SOX19A
SSPN	SOX19B
SSPO	SOX1A
SSPOP	SOX2
SSR1	SOX21
SSR2	SOX21A
SSR3	SOX21-AS1
SSR4	SOX21B
SSR4P1	SOX2OT
SSRP1	SOX2-OT
SSS-1	SOX3
SST	SOX30
SST-20	SOX4
SSTR1	SOX5
SSTR2	SOX6
SSTR3	SOX6OS
SSTR4	SOX7
SSTR5	SOX8
SSTR5-AS1	SOX9
SSTY1	SOX9A
SSTY2	SOX9B
SSU72	SP1
SSUH2	SP100

SSX1	SP110
SSX2	SP140
SSX2IP	SP140L
SSX3	SP2
SSX4	SP2-AS1
SSX5	SP2-DT
SSX6P	SP3
SSX8P	SP4
SSX9	SP5
ST13	SP5L
ST14	SP6
ST18	SP7
ST1A8	SP8
ST20	SP9
ST20-AS1	SPA17
ST3GAL1	SPAAR
ST3GAL2	SPACA4
ST3GAL3	SPACA6
ST3GAL4	SPACA9
ST3GAL5	SPACDR
ST3GAL6	SPAG1
ST6GAL1	SPAG11A
ST6GAL2	SPAG11B
ST6GALNAC1	SPAG16
ST6GALNAC2	SPAG17
ST6GALNAC3	SPAG4
ST6GALNAC4	SPAG5
ST6GALNAC5	SPAG5-AS1
ST6GALNAC6	SPAG6
ST7	SPAG7
ST7-AS1	SPAG8
ST7L	SPAG9
ST7-OT4	SPANXA1
ST8SIA1	SPANXA2-OT1
ST8SIA2	SPANXC
ST8SIA3	SPANXD

ST8SIA4
ST8SIA5
ST8SIA6
ST8SIA6-AS1
STAB1
STAB2
STAC
STAC2
STAC3
STAG1
STAG2
STAG3
STAG3L1
STAG3L3
STAG3L4
STAI
STAM
STAM2
STAMBP
STAMBPL1
STAP1
STAP2
STAP2A
STAR
STARD10
STARD13
STARD3
STARD3NL
STARD4
STARD4-AS1
STARD5
STARD7
STARD7-AS1
STARD8
STARD9
STAT1
SPANXN1
SPANXN2
SPANXN4
SPANXN5
SPARC
SPARCL1
SPART
SPAST
SPATA12
SPATA13
SPATA16
SPATA17
SPATA18
SPATA19
SPATA2
SPATA20
SPATA22
SPATA24
SPATA25
SPATA2L
SPATA3
SPATA31A1
SPATA31C1
SPATA31C2
SPATA31D1
SPATA31D5P
SPATA31E1
SPATA32
SPATA33
SPATA4
SPATA41
SPATA45
SPATA48
SPATA5
SPATA5L1
SPATA6

STAT2	SPATA6L
STAT3	SPATA7
STAT4	SPATA8
STAT5	SPATC1
STAT5A	SPATC1L
STAT5B	SPATS1
STAT6	SPATS2
STATH	SPATS2L
STAU1	SPC24
STAU2	SPC25
STAU2.S	SPCH-1
STAU2-AS1	SPCS2
STBD1	SPCS2P1
STC1	SPCS3
STC1L	SPDEF
STC2	SPDL1
STCH	SPDYA
STEAP1	SPDYC
STEAP1B	SPDYE1
STEAP2	SPDYE17
STEAP3	SPDYE2
STEAP3-AS1	SPDYE21
STEAP4	SPDYE2B
STEEP1	SPDYE4
STFA2	SPDYE5
STFA2L1	SPDYE7P
STFA2L2	SPDYE8
STFA3	SPECC1
STG	SPECC1L
STIL	SPECC1LB
STIM1	SPEER2
STIM2	SPEER4D
STIMATE	SPEER4F1
STIMATE-MUSTN1	SPEER8-PS1
STING1	SPEF1
STIP1	SPEF2

STK10	SPEG
STK11	SPEM1
STK11IP	SPEM2
STK16	SPEN
STK17A	SPESP1
STK17B	SPG11
STK19	SPG20
STK24	SPG21
STK24A	SPG7
STK25	SPHK1
STK26	SPHK2
STK3	SPHKAP
STK31	SPI1
STK32A	SPIA1
STK32B	SPIB
STK32C	SPIC
STK33	SPICE1
STK35	SPIDR
STK36	SPIN
STK38	SPIN1
STK38L	SPIN2A
STK39	SPIN2B
STK4	SPIN2D
STK40	SPIN3
STKLD1	SPIN4
STMN1	SPINDOC
STMN2	SPINK1
STMN2A	SPINK12
STMN3	SPINK13
STMN4	SPINK2
STMND1	SPINK4
STMP1	SPINK5
STN1	SPINK8
STOM	SPINT1
STOML1	SPINT2
STOML2	SPINT4

STOML3	SPIRE1
STOML3A	SPIRE2
STON1	SPN
STON1-GTF2A1L	SPN42DD
STON2	SPNS1
STOX1	SPNS2
STOX2	SPNS3
STPG1	SPO11
STPG2	SPOCD1
STPG3	SPOCK1
STRA6	SPOCK2
STRA6L	SPOCK3
STRADA	SPON1
STRADB	SPON1A
STRAP	SPON1B
STRBP	SPON2
STRC	SPOP
STRIP1	SPOPL
STRIP2	SPOUT1
STRN	SPP1
STRN3	SPP2
STRN4	SPPL2A
STS	SPPL2B
STT3A	SPPL2C
STT3B	SPPL3
STUB1	SPR
STUM	SPRED1
STX10	SPRED2
STX11	SPRED3
STX12	SPRI
STX16	SPRING1
STX17	SPRN
STX18	SPRNP1
STX18-AS1	SPRR1A
STX19	SPRR1B
STX1A	SPRR2A

STX1B	SPRR2B
STX2	SPRR2C
STX3	SPRR2D
STX4	SPRR2E
STX4A	SPRR2F
STX5	SPRR2G
STX5A	SPRR2H
STX6	SPRR2I
STX7	SPRR2J-PS
STX8	SPRR2K
STXBP1	SPRR3
STXBP2	SPRR4
STXBP3	SPRTN
STXBP4	SPRY1
STXBP5	SPRY2
STXBP5L	SPRY3
STXBP6	SPRY4
STYK1	SPRY4-IT1
STYX	SPRYD3
STYXL1	SPRYD4
STYXL2	SPRYD7
SU(R)	SPSB1
SUB1	SPSB2
SUCLA2	SPSB3
SUCLG1	SPSB4
SUCLG2	SPT1
SUCNR1	SPTA1
SUCO	SPTAN1
SUDS3	SPTB
SUFU	SPTBN1
SUGCT	SPTBN2
SUGP1	SPTBN4
SUGP2	SPTBN5
SUGT1	SPTLC1
SUGT1P1	SPTLC2
SUGT1P3	SPTLC3

SULF1	SPTSSA
SULF2	SPTSSB
SULF2A	SPTY2D1
SULT	SPTY2D1OS
SULT1A1	SPX
SULT1A2	SPZ1
SULT1A3	SQLE
SULT1A4	SQOR
SULT1B1	SQSTM1
SULT1C1	SRA1
SULT1C2	SRARP
SULT1C2A	SRBD1
SULT1C2P1	SRC
SULT1C3	SRCAP
SULT1C4	SRCIN1
SULT1D1	SRD
SULT1E1	SRD5A1
SULT1ST1	SRD5A2
SULT2A1	SRD5A3
SULT2A2	SRD5A3-AS1
SULT2A4	SREBF1
SULT2A6	SREBF2
SULT2A8	SREK1
SULT2B1	SREK1IP1
SULT4A1	SRF
SULT6B1	SRFBP1
SUMF1	SRGAP1
SUMF2	SRGAP2
SUMO1	SRGAP2B
SUMO2	SRGAP2C
SUMO2P1	SRGAP3
SUMO3	SRGAP3-AS2
SUMO4	SRGAP3-AS3
SUN1	SRGAP3-AS4
SUN2	SRGN
SUN3	SRI

SUN5	SRL
SUOX	SRM
SUPT16H	SRMS
SUPT20	SRP14
SUPT20HL1	SRP14-DT
SUPT20HL2	SRP19
SUPT3H	SRP54
SUPT4H1	SRP54A
SUPT5H	SRP54C
SUPT6H	SRP68
SUPT7L	SRP72
SUPV3L1	SRP9
SURF1	SRPK1
SURF2	SRPK2
SURF4	SRPK3
SURF6	SRPR
SUSD1	SRPRA
SUSD2	SRPRB
SUSD3	SRPX
SUSD4	SRPX2
SUSD5	SRR
SUSD6	SRRD
SUV39H1	SRRM1
SUV39H2	SRRM2
SUZ12	SRRM2-AS1
SUZ12P1	SRRM3
SV2A	SRRM4
SV2B	SRRM5
SV2C	SRRT
SVBP	SRSF1
SVEP1	SRSF10
SVIL	SRSF11
SVIL2P	SRSF12
SVIL-AS1	SRSF2
SVIP	SRSF3
SVOP	SRSF4

SVOPL	SRSF5
SVS5	SRSF6
SWAP70	SRSF7
SWI5	SRSF8
SWINGN	SRSF9
SWSAP1	SRST
SWT1	SRXN1
SXE2	SRY
SYAP1	SS18
SYBU	SS18L1
SYCE1	SS18L2
SYCE1L	SSB
SYCE2	SSBP1
SYCE3	SSBP2
SYCN	SSBP3
SYCP1	SSBP3-AS1
SYCP2	SSBP4
SYCP2L	SSC4D
SYCP3	SSC5D
SYDE1	SSH1
SYDE2	SSH2
SYF2	SSH3
SYK	SSL
SYMPK	SSNA1
SYN1	SSP-10
SYN2	SSPN
SYN3	SSPO
SYNB	SSPOP
SYNC	SSR1
SYNCRIP	SSR2
SYNDIG1	SSR3
SYNE1	SSR4
SYNE2	SSR4P1
SYNE3	SSRP1
SYNE4	SSS-1
SYNGAP1	SST

SYNGR1	SST-20
SYNGR2	SSTR1
SYNGR3	SSTR2
SYNGR4	SSTR3
SYNJ1	SSTR4
SYNJ2	SSTR5
SYNJ2BP	SSTR5-AS1
SYNJ2BP-COX16	SSTY1
SYNM	SSTY2
SYNPO	SSU72
SYNPO2	SSUH2
SYNPO2L	SSX1
SYNPR	SSX2
SYNPR-AS1	SSX2IP
SYNRG	SSX3
SYP	SSX4
SYPB	SSX5
SYPL1	SSX6P
SYPL2	SSX8P
SYS1	SSX9
SYT1	ST13
SYT10	ST14
SYT11	ST18
SYT12	ST1A8
SYT13	ST20
SYT14	ST20-AS1
SYT15	ST3GAL1
SYT16	ST3GAL2
SYT17	ST3GAL3
SYT2	ST3GAL4
SYT3	ST3GAL5
SYT4	ST3GAL6
SYT5	ST6GAL1
SYT6	ST6GAL2
SYT7	ST6GALNAC1
SYT8	ST6GALNAC2

SYT9	ST6GALNAC3
SYTL1	ST6GALNAC4
SYTL2	ST6GALNAC5
SYTL3	ST6GALNAC6
SYTL4	ST7
SYTL5	ST7-AS1
SYVN1	ST7L
SZRD1	ST7-OT4
SZT2	ST8SIA1
T	ST8SIA2
T01D3.6	ST8SIA3
T13F2.9	ST8SIA4
T16A9.5	ST8SIA5
T24C4.4	ST8SIA6
T27E7.1	ST8SIA6-AS1
TAAR3	STAB1
TAAR4	STAB2
TAAR5	STAC
TAAR7A	STAC2
TAAR8	STAC3
TAAR9	STAG1
TAB1	STAG2
TAB2	STAG3
TAB3	STAG3L1
TAC1	STAG3L2
TAC3	STAG3L3
TAC4	STAG3L4
TACC1	STAI
TACC2	STAM
TACC3	STAM2
TACO1	STAMBP
TACR1	STAMBPL1
TACR2	STAP1
TACSTD2	STAP2
TADA1	STAP2A
TADA2A	STAR

TADA2B	STARD10
TADA3	STARD13
TADA3L	STARD3
TAF1	STARD3NL
TAF10	STARD4
TAF11	STARD4-AS1
TAF12	STARD5
TAF13	STARD7
TAF15	STARD7-AS1
TAF1A	STARD8
TAF1A-AS1	STARD9
TAF1B	STAT1
TAF1C	STAT2
TAF1D	STAT3
TAF2	STAT4
TAF3	STAT5
TAF4	STAT5A
TAF4B	STAT5B
TAF5	STAT6
TAF5L	STATH
TAF6	STAU1
TAF6L	STAU2
TAF7	STAU2.S
TAF7L	STAU2-AS1
TAF8	STBD1
TAF9	STC1
TAF9B	STC1L
TAFA1	STC2
TAFA2	STCH
TAFA3	STEAP1
TAFA4	STEAP1B
TAFA5	STEAP2
TAFAZZIN	STEAP3
TAGAP	STEAP3-AS1
TAGLN	STEAP4
TAGLN2	STEEP1

TAGLN3	STFA2
TAK1	STFA2L1
TAL1	STFA2L2
TAL2	STFA3
TALDO1	STG
TAMALIN	STIL
TAMM41	STIM1
TANC1	STIM2
TANC2	STIMATE
TANGO2	STIMATE-MUSTN1
TANGO6	STING1
TANK	STIP1
TAOK1	STK10
TAOK2	STK11
TAOK3	STK11IP
TAP1	STK16
TAP2	STK17A
TAPBP	STK17B
TAPBPL	STK19
TAPT1	STK24
TAPT1-AS1	STK24A
TARBP1	STK25
TARBP2	STK26
TARDBP	STK3
TARM1	STK31
TARP	STK32A
TARS	STK32B
TARS1	STK32C
TARS2	STK33
TARS3	STK35
TARSL2	STK36
TAS1R1	STK38
TAS1R2	STK38L
TAS1R3	STK39
TAS2R1	STK4
TAS2R10	STK40

TAS2R105	STKLD1
TAS2R108	STMN1
TAS2R119	STMN2
TAS2R14	STMN2A
TAS2R16	STMN3
TAS2R30	STMN4
TAS2R31	STMND1
TAS2R38	STMP1
TAS2R4	STN1
TAS2R42	STOM
TAS2R43	STOML1
TAS2R5	STOML2
TAS2R50	STOML3
TAS2R60	STOML3A
TASOR	STON1
TASOR2	STON1-GTF2A1L
TASP1	STON2
TAT	STOX1
TATDN1	STOX2
TATDN2	STPG1
TATDN3	STPG2
TAX1BP1	STPG3
TAX1BP3	STPG4
TAX-6	STRA6
TAZ	STRA6L
TBATA	STRADA
TBC1D1	STRADB
TBC1D10A	STRAP
TBC1D10B	STRBP
TBC1D10C	STRC
TBC1D12	STRIP1
TBC1D13	STRIP2
TBC1D14	STRN
TBC1D15	STRN3
TBC1D16	STRN4
TBC1D17	STS

TBC1D19	STT3A
TBC1D2	STT3B
TBC1D20	STUB1
TBC1D21	STUM
TBC1D22A	STX10
TBC1D22B	STX11
TBC1D23	STX12
TBC1D24	STX16
TBC1D24.1.L	STX17
TBC1D25	STX18
TBC1D26	STX18-AS1
TBC1D28	STX19
TBC1D29P	STX1A
TBC1D2B	STX1B
TBC1D3	STX2
TBC1D30	STX3
TBC1D31	STX4
TBC1D32	STX4A
TBC1D3B	STX5
TBC1D3C	STX5A
TBC1D3F	STX6
TBC1D3H	STX7
TBC1D3P5	STX8
TBC1D4	STXBP1
TBC1D5	STXBP2
TBC1D7	STXBP3
TBC1D8	STXBP4
TBC1D8B	STXBP5
TBC1D9	STXBP5-AS1
TBC1D9B	STXBP5L
TBCA	STXBP6
TBCB	STYK1
TBCC	STYX
TBCCD1	STYXL1
TBCD	STYXL2
TBCE	SU(R)

TBCEL	SUB1
TBCK	SUCLA2
TBILA	SUCLG1
TBK1	SUCLG2
TBKBP1	SUCNR1
TBL1X	SUCO
TBL1XR1	SUDS3
TBL1Y	SUFU
TBL2	SUGCT
TBL3	SUGP1
TBP	SUGP2
TBPL1	SUGT1
TBPL2	SUGT1P1
TBR1	SUGT1P3
TBRG1	SULF1
TBRG4	SULF2
TBX1	SULF2A
TBX10	SULT
TBX15	SULT1A1
TBX18	SULT1A2
TBX19	SULT1A3
TBX2	SULT1A4
TBX20	SULT1B1
TBX21	SULT1C1
TBX22	SULT1C2
TBX2B	SULT1C2A
TBX3	SULT1C2P1
TBX4	SULT1C3
TBX5	SULT1C4
TBX6	SULT1D1
TBXA2R	SULT1E1
TBXAS1	SULT1ST1
TBXT	SULT2A1
TBXT.S	SULT2A2
TBXTA	SULT2A4
TC2N	SULT2A6

TCAF1	SULT2A8
TCAF2	SULT2B1
TCAF2P1	SULT4A1
TCAF3	SULT6B1
TCAIM	SUMF1
TCAM1	SUMF2
TCAM1P	SUMO1
TCAP	SUMO1P3
TCEA1	SUMO2
TCEA2	SUMO2P1
TCEA3	SUMO3
TCEAL1	SUMO4
TCEAL2	SUN1
TCEAL3	SUN2
TCEAL4	SUN3
TCEAL5	SUN5
TCEAL6	SUOX
TCEAL7	SUPT16H
TCEAL8	SUPT20
TCEAL9	SUPT20HL1
TCEANC	SUPT20HL2
TCEANC2	SUPT3H
TCEB2	SUPT4H1
TCERG1	SUPT5H
TCERG1L	SUPT6H
TCF12	SUPT7L
TCF15	SUPV3L1
TCF19	SURF1
TCF20	SURF2
TCF21	SURF4
TCF23	SURF6
TCF24	SUSD1
TCF25	SUSD2
TCF3	SUSD3
TCF4	SUSD4
TCF7	SUSD5

TCF7L1	SUSD6
TCF7L2	SUV39H1
TCFAP2B	SUV39H2
TCFL5	SUZ12
TCHH	SUZ12P1
TCHP	SV2A
TCIM	SV2B
TCIRG1	SV2C
TCL1B	SVBP
TCL1B1	SVEP1
TCL1B3	SVIL
TCL1B4	SVIL2P
TCN1	SVIL-AS1
TCN2	SVIP
TCOF1	SVOP
TCP1	SVOPL
TCP10A	SVS5
TCP10L	SWAP70
TCP11	SWI5
TCP11L1	SWINGN
TCP11L2	SWSAP1
TCRA	SWT1
TCRA-V54	SXE2
TCRB	SYAP1
TCRD	SYBU
TCTA	SYCE1
TCTE1	SYCE1L
TCTE2	SYCE2
TCTN1	SYCE3
TCTN2	SYCN
TCTN3	SYCP1
TDG	SYCP2
TDGF1	SYCP2L
TDGF1P3	SYCP3
TDGF1P5	SYDE1
TDH	SYDE2

TDO2	SYF2
TDP1	SYK
TDP2	SYMPK
TDPOZ1	SYN1
TDRD1	SYN2
TDRD10	SYN3
TDRD12	SYNB
TDRD3	SYNC
TDRD5	SYNCRIP
TDRD6	SYNDIG1
TDRD7	SYNE1
TDRD9	SYNE2
TDRG1	SYNE3
TDRKH	SYNE4
TDRP	SYNGAP1
TEAD1	SYNGR1
TEAD2	SYNGR2
TEAD3	SYNGR3
TEAD4	SYNGR4
TEC	SYNJ1
TECPR1	SYNJ2
TECPR2	SYNJ2BP
TECR	SYNJ2BP-COX16
TECRL	SYNM
TECTA	SYNPO
TECTB	SYNPO2
TEDC1	SYNPO2L
TEDC2	SYNPR
TEF	SYNPR-AS1
TEFM	SYNRG
TEGT	SYP
TEK	SYPB
TEKT1	SYPL1
TEKT2	SYPL2
TEKT3	SYS1
TEKT4	SYT1

TEKT4P2	SYT10
TEKTIP1	SYT11
TELO2	SYT12
TEN1	SYT13
TEN1-CDK3	SYT14
TENM1	SYT15
TENM2	SYT16
TENM3	SYT17
TENM4	SYT2
TENT2	SYT3
TENT4A	SYT4
TENT4B	SYT5
TENT5A	SYT6
TENT5B	SYT7
TENT5C	SYT8
TEP1	SYT9
TEP2	SYTL1
TEPP	SYTL2
TEPSIN	SYTL3
TERA	SYTL4
TERB1	SYTL5
TERB2	SYVN1
TERC	SZRD1
TERF1	SZT2
TERF2	T
TERF2IP	T01D3.6
TERT	T13F2.9
TES	T16A9.5
TES3-PS	T24C4.4
TESC	T27E7.1
TESK1	TAAR3
TESK2	TAAR4
TESMIN	TAAR5
TESPA1	TAAR7A
TET1	TAAR8
TET2	TAAR9

TET3	TAB1
TEX10	TAB2
TEX101	TAB3
TEX11	TAC1
TEX12	TAC3
TEX13A	TAC4
TEX13B	TACC1
TEX14	TACC2
TEX15	TACC3
TEX16	TACO1
TEX19	TACR1
TEX2	TACR2
TEX22	TACSTD2
TEX26	TADA1
TEX261	TADA2A
TEX264	TADA2B
TEX28	TADA3
TEX29	TADA3L
TEX30	TAF1
TEX37	TAF10
TEX41	TAF11
TEX44	TAF12
TEX47	TAF13
TEX49	TAF15
TEX55	TAF1A
TEX9	TAF1A-AS1
TF	TAF1B
TFAM	TAF1C
TFAP2A	TAF1D
TFAP2A-AS1	TAF2
TFAP2B	TAF3
TFAP2C	TAF4
TFAP2D	TAF4B
TFAP2E	TAF5
TFAP4	TAF5L
TFB1M	TAF6

TFB2M	TAF6L
TFCP2	TAF7
TFCP2L1	TAF7L
TFDP1	TAF8
TFDP2	TAF9
TFE3	TAF9B
TFEB	TAFA1
TFEC	TAFA2
TFF1	TAFA3
TFF2	TAFA4
TFF3	TAFA5
TFG	TAFAZZIN
TFIP11	TAGAP
TFPI	TAGLN
TFPI2	TAGLN2
TFPT	TAGLN3
TFR2	TAK1
TFRC	TAL1
TG	TAL2
TGDS	TALDO1
TGFA	TAM
TGFB1	TAMALIN
TGFB1A	TAMM41
TGFB1I1	TANC1
TGFB2	TANC2
TGFB2-AS1	TANGO2
TGFB3	TANGO6
TGFBI	TANK
TGFBR1	TAOK1
TGFBR2	TAOK2
TGFBR3	TAOK3
TGFBR3L	TAP1
TGFBRAP1	TAP2
TGIF1	TAPBP
TGIF2	TAPBPL
TGIF2-RAB5IF	TAPT1

TGM1	TAPT1-AS1
TGM2	TARBP1
TGM3	TARBP2
TGM4	TARDBP
TGM5	TARM1
TGM6	TARP
TGM7	TARS
TGOLN1	TARS1
TGOLN2	TARS2
TGS1	TARS3
TGTP1	TARSL2
TH	TAS1R1
THADA	TAS1R2
THAP1	TAS1R3
THAP10	TAS2R1
THAP11	TAS2R10
THAP12	TAS2R105
THAP2	TAS2R108
THAP3	TAS2R119
THAP4	TAS2R14
THAP5	TAS2R16
THAP6	TAS2R30
THAP7	TAS2R31
THAP7-AS1	TAS2R38
THAP8	TAS2R4
THAP9	TAS2R42
THAP9-AS1	TAS2R43
THBD	TAS2R5
THBS1	TAS2R50
THBS2	TAS2R60
THBS3	TASOR
THBS4	TASOR2
THBS4-AS1	TASP1
THD1	TAT
THEG	TATDN1
THEGL	TATDN2

THEM4	TATDN3
THEM5	TAX1BP1
THEM6	TAX1BP3
THEM7	TAX-6
THEMIS	TAZ
THEMIS2	TBATA
THG1L	TBC1D1
THNSL1	TBC1D10A
THNSL2	TBC1D10B
THOC1	TBC1D10C
THOC2	TBC1D12
THOC3	TBC1D13
THOC4	TBC1D14
THOC5	TBC1D15
THOC6	TBC1D16
THOC7	TBC1D17
THOC7-AS1	TBC1D19
THOP1	TBC1D2
THOR	TBC1D20
THPO	TBC1D21
THRA	TBC1D22A
THRAP3	TBC1D22B
THRB	TBC1D23
THRB-IT1	TBC1D24
THRSP	TBC1D24.1.L
THSD1	TBC1D25
THSD1P1	TBC1D26
THSD4	TBC1D28
THSD7A	TBC1D29P
THSD7B	TBC1D2B
THTPA	TBC1D3
THUMPD1	TBC1D30
THUMPD3	TBC1D31
THUMPD3-AS1	TBC1D32
THY1	TBC1D3B
THYN1	TBC1D3C

TIA1	TBC1D3F
TIAL1	TBC1D3H
TIAM1	TBC1D3P5
TIAM2	TBC1D4
TICAM1	TBC1D5
TICAM2	TBC1D7
TICRR	TBC1D8
TIE1	TBC1D8B
TIFA	TBC1D9
TIFAB	TBC1D9B
TIGAR	TBCA
TIGD1	TBCB
TIGD2	TBCC
TIGD3	TBCCD1
TIGD4	TBCD
TIGD5	TBCE
TIGD7	TBCEL
TIMD2	TBCK
TIMD4	TBILA
TIMELESS	TBK1
TIMM10	TBKBP1
TIMM10B	TBL1X
TIMM13	TBL1XR1
TIMM17A	TBL1Y
TIMM17B	TBL2
TIMM21	TBL3
TIMM22	TBP
TIMM23	TBPL1
TIMM23B	TBPL2
TIMM29	TBR1
TIMM44	TBRG1
TIMM50	TBRG4
TIMM8A	TBX1
TIMM8A1	TBX10
TIMM8A2	TBX15
TIMM8B	TBX18

TIMM9	TBX19
TIMMDC1	TBX2
TIMP	TBX20
TIMP1	TBX21
TIMP2	TBX22
TIMP3	TBX2B
TIMP4	TBX3
TINAG	TBX4
TINAGL1	TBX5
TINCR	TBX6
TINF2	TBXA2R
TINP1	TBXAS1
TIPARP	TBXT
TIPARP-AS1	TBXT.S
TIPIN	TBXTA
TIPIN.L	TC2N
TIPRL	TCAF1
TIRAP	TCAF2
TJAP1	TCAF2P1
TJP1	TCAF3
TJP2	TCAIM
TJP3	TCAM1
TK1	TCAM1P
TK2	TCAP
TKFC	TCEA1
TKT	TCEA2
TKTL1	TCEA3
TKTL2	TCEAL1
TLCD1	TCEAL2
TLCD2	TCEAL3
TLCD3A	TCEAL4
TLCD3B	TCEAL5
TLCD4	TCEAL6
TLCD4B	TCEAL7
TLCD5	TCEAL8
TLDC2	TCEAL9

TLE1	TCEANC
TLE2	TCEANC2
TLE3	TCEB2
TLE4	TCERG1
TLE5	TCERG1L
TLE6	TCF12
TLK1	TCF15
TLK2	TCF19
TLL	TCF20
TLL1	TCF21
TLL2	TCF23
TLN1	TCF24
TLN2	TCF25
TLNRD1	TCF3
TLR1	TCF4
TLR10	TCF7
TLR11	TCF7L1
TLR12	TCF7L2
TLR13	TCFAP2B
TLR2	TCFL5
TLR3	TCHH
TLR4	TCHP
TLR5	TCIM
TLR6	TCIRG1
TLR7	TCL1B
TLR8	TCL1B1
TLR9	TCL1B3
TLX1	TCL1B4
TLX1NB	TCN1
TLX2	TCN2
TLX3	TCOF1
TM2D1	TCP1
TM2D2	TCP10A
TM2D3	TCP10L
TM4SF1	TCP11
TM4SF18	TCP11L1

TM4SF19	TCP11L2
TM4SF19-AS1	TCRA
TM4SF19-DYNLT2B	TCRA-V54
TM4SF20	TCRB
TM4SF4	TCRD
TM4SF5	TCTA
TM6SF1	TCTE1
TM6SF2	TCTE2
TM7SF2	TCTN1
TM7SF3	TCTN2
TM9SF1	TCTN3
TM9SF2	TDG
TM9SF3	TDGF1
TM9SF4	TDGF1P3
TMA16	TDGF1P5
TMA7	TDH
TMBIM1	TDO2
TMBIM4	TDP1
TMBIM6	TDP2
TMC1	TDPOZ1
TMC3	TDRD1
TMC4	TDRD10
TMC5	TDRD12
TMC6	TDRD3
TMC7	TDRD5
TMC8	TDRD6
TMCC1	TDRD7
TMCC1-DT	TDRD9
TMCC2	TDRG1
TMCC3	TDRKH
TMCO1	TDRP
TMCO3	TEAD1
TMCO4	TEAD2
TMCO5A	TEAD3
TMCO6	TEAD4
TMED1	TEC

TMED10	TECPR1
TMED10P1	TECPR2
TMED11	TECR
TMED11P	TECRL
TMED1A	TECTA
TMED2	TECTB
TMED3	TEDC1
TMED4	TEDC2
TMED5	TEF
TMED6	TEFM
TMED7	TEGT
TMED7-TICAM2	TEK
TMED8	TEKT1
TMED9	TEKT2
TMEFF1	TEKT3
TMEFF1A	TEKT4
TMEFF2	TEKT4P2
TMEM100	TEKTIP1
TMEM101	TELO2
TMEM102	TEN1
TMEM104	TEN1-CDK3
TMEM105	TENM1
TMEM106A	TENM2
TMEM106B	TENM3
TMEM106C	TENM4
TMEM107	TENT2
TMEM108	TENT4A
TMEM109	TENT4B
TMEM11	TENT5A
TMEM115	TENT5B
TMEM116	TENT5C
TMEM117	TEP1
TMEM119	TEP2
TMEM120A	TEPP
TMEM120B	TEPSIN
TMEM121	TERA

TMEM121B	TERB1
TMEM123	TERB2
TMEM123-DT	TERC
TMEM125	TERF1
TMEM126A	TERF2
TMEM126B	TERF2IP
TMEM127	TERT
TMEM128	TES
TMEM129	TES3-PS
TMEM130	TESC
TMEM131	TESK1
TMEM131L	TESK2
TMEM132A	TESMIN
TMEM132B	TESPA1
TMEM132C	TET1
TMEM132D	TET2
TMEM132D-AS1	TET3
TMEM132E	TEX10
TMEM132E-DT	TEX101
TMEM134	TEX11
TMEM135	TEX12
TMEM138	TEX13A
TMEM139	TEX13B
TMEM140	TEX14
TMEM141	TEX15
TMEM143	TEX16
TMEM144	TEX19
TMEM145	TEX2
TMEM147	TEX22
TMEM147-AS1	TEX26
TMEM14A	TEX261
TMEM14B	TEX264
TMEM14C	TEX28
TMEM150A	TEX29
TMEM150B	TEX30
TMEM150C	TEX37

TMEM151A	TEX41
TMEM151B	TEX44
TMEM154	TEX47
TMEM156	TEX49
TMEM158	TEX55
TMEM159	TEX9
TMEM160	TF
TMEM161A	TFAM
TMEM161B	TFAP2A
TMEM161B-DT	TFAP2A-AS1
TMEM163	TFAP2B
TMEM164	TFAP2C
TMEM165	TFAP2D
TMEM167	TFAP2E
TMEM167A	TFAP4
TMEM167B	TFB1M
TMEM168	TFB2M
TMEM169	TFCP2
TMEM17	TFCP2L1
TMEM170A	TFDP1
TMEM170B	TFDP2
TMEM171	TFE3
TMEM175	TFEB
TMEM176A	TFEC
TMEM176B	TFF1
TMEM177	TFF2
TMEM178	TFF3
TMEM178A	TFG
TMEM178B	TFIP11
TMEM179B	TFPI
TMEM18	TFPI2
TMEM181	TFPT
TMEM181A	TFR2
TMEM182	TFRC
TMEM183BP	TG
TMEM184A	TGDS

TMEM184B	TGFA
TMEM184C	TGFB1
TMEM185A	TGFB1A
TMEM185B	TGFB1I1
TMEM186	TGFB2
TMEM187	TGFB2-AS1
TMEM19	TGFB3
TMEM190	TGFBI
TMEM191A	TGFBR1
TMEM192	TGFBR2
TMEM196	TGFBR3
TMEM198	TGFBR3L
TMEM198B	TGFBRAP1
TMEM199	TGIF1
TMEM200A	TGIF2
TMEM200B	TGIF2-RAB5IF
TMEM200C	TGM1
TMEM201	TGM2
TMEM202-AS1	TGM3
TMEM203	TGM4
TMEM204	TGM5
TMEM205	TGM6
TMEM208	TGM7
TMEM209	TGOLN1
TMEM210	TGOLN2
TMEM212	TGS1
TMEM213	TGTP1
TMEM214	TH
TMEM215	THADA
TMEM216	THAP1
TMEM217	THAP10
TMEM218	THAP11
TMEM219	THAP12
TMEM220	THAP2
TMEM220-AS1	THAP3
TMEM221	THAP4

TMEM222	THAP5
TMEM223	THAP6
TMEM225	THAP7
TMEM225B	THAP7-AS1
TMEM229B	THAP8
TMEM230	THAP9
TMEM231	THAP9-AS1
TMEM232	THAS
TMEM234	THBD
TMEM235	THBS1
TMEM236	THBS2
TMEM237	THBS3
TMEM237B	THBS4
TMEM238	THBS4-AS1
TMEM239	THD1
TMEM240	THEG
TMEM241	THEGL
TMEM242	THEM4
TMEM243	THEM5
TMEM244	THEM6
TMEM245	THEM7
TMEM246-AS1	THEMIS
TMEM247	THEMIS2
TMEM248	THG1L
TMEM249	THNSL1
TMEM25	THNSL2
TMEM250	THOC1
TMEM251	THOC2
TMEM252	THOC3
TMEM253	THOC4
TMEM254	THOC5
TMEM254.S	THOC6
TMEM254-AS1	THOC7
TMEM255A	THOC7-AS1
TMEM256	THOP1
TMEM256-PLSCR3	THOR

TMEM258	THPO
TMEM259	THRA
TMEM26	THRAP3
TMEM260	THRB
TMEM262	THRB-IT1
TMEM263	THRSP
TMEM266	THSD1
TMEM267	THSD1P1
TMEM268	THSD4
TMEM269	THSD7A
TMEM270	THSD7B
TMEM29	THTPA
TMEM30A	THUMPD1
TMEM30B	THUMPD3
TMEM30C	THUMPD3-AS1
TMEM31	THY1
TMEM33	THYN1
TMEM35A	TIA1
TMEM35B	TIAL1
TMEM37	TIAM1
TMEM38A	TIAM2
TMEM38B	TICAM1
TMEM39A	TICAM2
TMEM39B	TICRR
TMEM40	TIE1
TMEM41A	TIFA
TMEM41B	TIFAB
TMEM42	TIGAR
TMEM43	TIGD1
TMEM44	TIGD2
TMEM44-AS1	TIGD3
TMEM45A	TIGD4
TMEM45B	TIGD5
TMEM47	TIGD7
TMEM50A	TIMD2
TMEM50B	TIMD4

TMEM51	TIMELESS
TMEM52	TIMM10
TMEM52B	TIMM10B
TMEM53	TIMM13
TMEM54	TIMM17A
TMEM59	TIMM17B
TMEM59L	TIMM21
TMEM60	TIMM22
TMEM61	TIMM23
TMEM62	TIMM23B
TMEM63A	TIMM29
TMEM63B	TIMM44
TMEM63C	TIMM50
TMEM64	TIMM8A
TMEM65	TIMM8A1
TMEM67	TIMM8A2
TMEM68	TIMM8B
TMEM69	TIMM9
TMEM70	TIMMDC1
TMEM71	TIMP
TMEM72	TIMP1
TMEM74	TIMP2
TMEM74B	TIMP3
TMEM79	TIMP4
TMEM80	TINAG
TMEM81	TINAGL1
TMEM82	TINCR
TMEM86A	TINF2
TMEM87A	TINP1
TMEM87B	TIPARP
TMEM88	TIPARP-AS1
TMEM89	TIPIN
TMEM8B	TIPIN.L
TMEM9	TIPRL
TMEM91	TIRAP
TMEM92	TJAP1

TMEM92-AS1	TJP1
TMEM94	TJP2
TMEM97	TJP3
TMEM98	TK1
TMEM9B	TK2
TMEM9B-AS1	TKFC
TMF1	TKT
TMIE	TKTL1
TMIGD2	TKTL2
TMLHE	TLCD1
TMOD1	TLCD2
TMOD2	TLCD3A
TMOD3	TLCD3B
TMOD4	TLCD4
TMPO	TLCD4B
TMPO-AS1	TLCD5
TMPRSS11B	TLDC2
TMPRSS11D	TLE1
TMPRSS11E	TLE2
TMPRSS12	TLE3
TMPRSS13	TLE4
TMPRSS13A	TLE5
TMPRSS15	TLE6
TMPRSS2	TLK1
TMPRSS3	TLK2
TMPRSS4	TLL
TMPRSS5	TLL1
TMPRSS6	TLL2
TMPRSS7	TLN1
TMPRSS9	TLN2
TMSB	TLNRD1
TMSB10	TLR1
TMSB15A	TLR10
TMSB15B	TLR11
TMSB15B2	TLR12
TMSB15L	TLR13

TMSB4X	TLR2
TMSB4XP6	TLR3
TMSB4XP8	TLR4
TMSB4Y	TLR5
TMTC1	TLR6
TMTC2	TLR7
TMTC3	TLR8
TMTC4	TLR9
TMUB1	TLX1
TMUB2	TLX1NB
TMX1	TLX2
TMX2	TLX3
TMX2A	TM2D1
TMX3	TM2D2
TMX4	TM2D3
TNC	TM4SF1
TNF	TM4SF18
TNFA	TM4SF19
TNFAIP1	TM4SF19-AS1
TNFAIP2	TM4SF19-DYNLT2B
TNFAIP3	TM4SF20
TNFAIP6	TM4SF4
TNFAIP8	TM4SF5
TNFAIP8L1	TM6SF1
TNFAIP8L2	TM6SF2
TNFAIP8L3	TM7SF2
TNFRSF10A	TM7SF3
TNFRSF10B	TM9SF1
TNFRSF10C	TM9SF2
TNFRSF10D	TM9SF3
TNFRSF11A	TM9SF4
TNFRSF11B	TMA16
TNFRSF12A	TMA7
TNFRSF13B	TMBIM1
TNFRSF13C	TMBIM4
TNFRSF14	TMBIM6

TNFRSF14-AS1	TMC1
TNFRSF17	TMC3
TNFRSF18	TMC4
TNFRSF19	TMC5
TNFRSF1A	TMC6
TNFRSF1B	TMC7
TNFRSF21	TMC8
TNFRSF25	TMCC1
TNFRSF26	TMCC1-DT
TNFRSF4	TMCC2
TNFRSF6B	TMCC3
TNFRSF8	TMCO1
TNFRSF9	TMCO3
TNFSF10	TMCO4
TNFSF10L	TMCO5A
TNFSF11	TMCO6
TNFSF12	TMED1
TNFSF13	TMED10
TNFSF13B	TMED10P1
TNFSF14	TMED11
TNFSF15	TMED11P
TNFSF18	TMED1A
TNFSF4	TMED2
TNFSF8	TMED3
TNFSF9	TMED4
TNIK	TMED5
TNIP1	TMED6
TNIP2	TMED7
TNIP3	TMED7-TICAM2
TNK1	TMED8
TNK2	TMED9
TNKS	TMEFF1
TNKS1BP1	TMEFF1A
TNKS2	TMEFF2
TNMD	TMEM100
TNN	TMEM101

TNNC1	TMEM102
TNNC2	TMEM104
TNNI1	TMEM105
TNNI1.2.L	TMEM106A
TNNI2	TMEM106B
TNNI2A.2	TMEM106C
TNNI2A.4	TMEM107
TNNI2B.1	TMEM108
TNNI3	TMEM109
TNNI3K	TMEM11
TNNT1	TMEM115
TNNT2	TMEM116
TNNT3	TMEM117
TNNT3A	TMEM119
TNNT3B	TMEM120A
TNP1	TMEM120B
TNP2	TMEM121
TNPO1	TMEM121B
TNPO2	TMEM123
TNPO3	TMEM123-DT
TNR	TMEM125
TNRC18	TMEM126A
TNRC6A	TMEM126B
TNRC6B	TMEM127
TNRC6C	TMEM128
TNS1	TMEM129
TNS2	TMEM130
TNS3	TMEM131
TNS4	TMEM131L
TNXB	TMEM132A
TO	TMEM132B
TOB1	TMEM132C
TOB1-AS1	TMEM132D
TOB2	TMEM132D-AS1
TOB2P1	TMEM132E
TOE1	TMEM132E-DT

TOGARAM1	TMEM134
TOGARAM2	TMEM135
TOLL-9	TMEM138
TOLLIP	TMEM139
TOLLIP-DT	TMEM140
TOM1	TMEM141
TOM1L1	TMEM143
TOM1L2	TMEM144
TOMM20	TMEM145
TOMM22	TMEM147
TOMM34	TMEM147-AS1
TOMM40	TMEM14A
TOMM40L	TMEM14B
TOMM5	TMEM14C
TOMM6	TMEM150A
TOMM7	TMEM150B
TOMM70	TMEM150C
TOMM70A	TMEM151A
TONSL	TMEM151B
TONSL-AS1	TMEM154
TOP1	TMEM156
TOP1MT	TMEM158
TOP1P1	TMEM159
TOP1P2	TMEM160
TOP2	TMEM161A
TOP2A	TMEM161B
TOP2B	TMEM161B-DT
TOP3A	TMEM163
TOP3B	TMEM164
TOPBP1	TMEM165
TOPORS	TMEM167
TOR1A	TMEM167A
TOR1AIP1	TMEM167B
TOR1AIP2	TMEM168
TOR1B	TMEM169
TOR2A	TMEM17

TOR3A	TMEM170A
TOR4A	TMEM170B
TOX	TMEM171
TOX2	TMEM175
TOX3	TMEM176A
TOX4	TMEM176B
TP53	TMEM177
TP53AIP1	TMEM178
TP53BP1	TMEM178A
TP53BP2	TMEM178B
TP53I11	TMEM179B
TP53I13	TMEM18
TP53I3	TMEM181
TP53INP1	TMEM181A
TP53INP2	TMEM182
TP53TG1	TMEM183BP
TP53TG3	TMEM184A
TP53TG3B	TMEM184B
TP53TG5	TMEM184C
TP63	TMEM185A
TP73	TMEM185B
TP73-AS1	TMEM186
TPBG	TMEM187
TPBPA	TMEM19
TPCN1	TMEM190
TPCN2	TMEM191A
TPD52	TMEM192
TPD52L1	TMEM196
TPD52L2	TMEM198
TPGS1	TMEM198B
TPGS2	TMEM199
TPH1	TMEM200A
TPH2	TMEM200B
TPI	TMEM200C
TPI1	TMEM201
TPI1A	TMEM202-AS1

TPI1P2	TMEM203
TPK1	TMEM204
TPM1	TMEM205
TPM2	TMEM208
TPM3	TMEM209
TPM3P9	TMEM210
TPM4	TMEM212
TPM4A	TMEM213
TPMA	TMEM214
TPMT	TMEM215
TPMT.2	TMEM216
TPO	TMEM217
TPP1	TMEM218
TPP2	TMEM219
TPPP	TMEM220
TPPP2	TMEM220-AS1
TPPP3	TMEM221
TPR	TMEM222
TPRA1	TMEM223
TPRG1	TMEM225
TPRG1L	TMEM225B
TPRKB	TMEM229B
TPRN	TMEM230
TPRXL	TMEM231
TPSAB1	TMEM232
TPSB2	TMEM234
TPSD1	TMEM235
TPSG1	TMEM236
TPST1	TMEM237
TPST2	TMEM237B
TPT1	TMEM238
TPT1-AS1	TMEM239
TPT1P8	TMEM240
TPT1P9	TMEM241
TPTE	TMEM242
TPTE2	TMEM243

TPTE2P1	TMEM244
TPTE2P2	TMEM245
TPTE2P5	TMEM246-AS1
TPTE2P6	TMEM247
TPTEP1	TMEM248
TPX2	TMEM249
TRA	TMEM25
TRA2A	TMEM250
TRA2B	TMEM251
TRABD	TMEM252
TRABD2A	TMEM253
TRABD2B	TMEM254
TRAC	TMEM254.S
TRADD	TMEM254-AS1
TRAF1	TMEM255A
TRAF2	TMEM256
TRAF3	TMEM256-PLSCR3
TRAF3IP1	TMEM258
TRAF3IP2	TMEM259
TRAF3IP2-AS1	TMEM26
TRAF3IP3	TMEM260
TRAF4	TMEM262
TRAF5	TMEM263
TRAF6	TMEM266
TRAF7	TMEM267
TRAFD1	TMEM268
TRAIP	TMEM269
TRAK1	TMEM270
TRAK2	TMEM29
TRAM1	TMEM30A
TRAM1L1	TMEM30B
TRAM2	TMEM30C
TRAM2-AS1	TMEM31
TRANK1	TMEM33
TRAP1	TMEM35A
TRAPPC1	TMEM35B

TRAPPC10	TMEM37
TRAPPC11	TMEM38A
TRAPPC12	TMEM38B
TRAPPC13	TMEM39A
TRAPPC14	TMEM39B
TRAPPC2	TMEM40
TRAPPC2B	TMEM41A
TRAPPC2L	TMEM41B
TRAPPC3	TMEM42
TRAPPC4	TMEM43
TRAPPC5	TMEM44
TRAPPC6A	TMEM44-AS1
TRAPPC6B	TMEM45A
TRAPPC8	TMEM45B
TRAPPC9	TMEM47
TRARG1	TMEM50A
TRAT1	TMEM50B
TRAV13-2	TMEM51
TRAV4D-4	TMEM52
TRAV7-4	TMEM52B
TRAV8-3	TMEM53
TRAV9D-3	TMEM54
TRBC1	TMEM59
TRBC2	TMEM59L
TRBL	TMEM60
TRBV5-4	TMEM61
TRD	TMEM62
TRD-GTC10-1	TMEM63A
TRDMT1	TMEM63B
TRDN	TMEM63C
TRDV3	TMEM64
TRE-CTC1-6	TMEM65
TREM1	TMEM67
TREM2	TMEM68
TREM3	TMEM69
TREML1	TMEM70

TREML2	TMEM71
TREML4	TMEM72
TRERF1	TMEM74
TREX1	TMEM74B
TREX2	TMEM79
TRF	TMEM80
TRF-GAA1-2	TMEM81
TRG-AS1	TMEM82
TRGC2	TMEM86A
TRGV5	TMEM87A
TRGV9	TMEM87B
TRH	TMEM88
TRHDE	TMEM89
TRHDE-AS1	TMEM8B
TRHR	TMEM9
TRIAP1	TMEM91
TRIB1	TMEM92
TRIB2	TMEM92-AS1
TRIB3	TMEM94
TRIL	TMEM97
TRIM10	TMEM98
TRIM11	TMEM9B
TRIM13	TMEM9B-AS1
TRIM14	TMF1
TRIM15	TMIE
TRIM16	TMIGD2
TRIM16L	TMLHE
TRIM17	TMOD1
TRIM2	TMOD2
TRIM21	TMOD3
TRIM22	TMOD4
TRIM23	TMPO
TRIM24	TMPO-AS1
TRIM25	TMPRSS11B
TRIM25L	TMPRSS11D
TRIM26	TMPRSS11E

TRIM27	TMPRSS12
TRIM28	TMPRSS13
TRIM29	TMPRSS13A
TRIM3	TMPRSS15
TRIM30B	TMPRSS2
TRIM30D	TMPRSS3
TRIM31	TMPRSS4
TRIM32	TMPRSS5
TRIM33	TMPRSS6
TRIM34	TMPRSS7
TRIM35	TMPRSS9
TRIM36	TMSB
TRIM37	TMSB10
TRIM38	TMSB15A
TRIM39	TMSB15B
TRIM4	TMSB15B2
TRIM41	TMSB15L
TRIM43	TMSB4X
TRIM44	TMSB4XP6
TRIM45	TMSB4XP8
TRIM46	TMSB4Y
TRIM47	TMTC1
TRIM5	TMTC2
TRIM50	TMTC3
TRIM52-AS1	TMTC4
TRIM53AP	TMUB1
TRIM54	TMUB2
TRIM55	TMX1
TRIM55A	TMX2
TRIM56	TMX2A
TRIM59	TMX2-CTNND1
TRIM6	TMX3
TRIM60	TMX4
TRIM61	TNC
TRIM62	TNF
TRIM63	TNFA

TRIM64	TNFAIP1
TRIM65	TNFAIP2
TRIM66	TNFAIP3
TRIM67	TNFAIP6
TRIM68	TNFAIP8
TRIM69	TNFAIP8L1
TRIM6-TRIM34	TNFAIP8L2
TRIM7	TNFAIP8L3
TRIM71	TNFRSF10A
TRIM72	TNFRSF10B
TRIM73	TNFRSF10C
TRIM74	TNFRSF10D
TRIM8	TNFRSF11A
TRIM80	TNFRSF11B
TRIM9	TNFRSF12A
TRIML1	TNFRSF13B
TRIML2	TNFRSF13C
TRIO	TNFRSF14
TRIOBP	TNFRSF14-AS1
TRIP10	TNFRSF17
TRIP11	TNFRSF18
TRIP12	TNFRSF19
TRIP13	TNFRSF1A
TRIP4	TNFRSF1B
TRIP6	TNFRSF21
TRIQK	TNFRSF25
TRIR	TNFRSF26
TRIT1	TNFRSF4
TRL-CAG2-1	TNFRSF6B
TRMO	TNFRSF8
TRMT1	TNFRSF9
TRMT10A	TNFSF10
TRMT10B	TNFSF10L
TRMT10C	TNFSF11
TRMT11	TNFSF12
TRMT112	TNFSF13

TRMT12	TNFSF13B
TRMT13	TNFSF14
TRMT1L	TNFSF15
TRMT2A	TNFSF18
TRMT2B	TNFSF4
TRMT44	TNFSF8
TRMT5	TNFSF9
TRMT6	TNIK
TRMT61A	TNIP1
TRMT61B	TNIP2
TRMT9B	TNIP3
TRMU	TNK1
TRNA	TNK2
TRNAU1AP	TNK2-AS1
TRNC	TNKS
TRND	TNKS1BP1
TRNG	TNKS2
TRNI	TNMD
TRNL1	TNN
TRNM	TNNC1
TRNN	TNNC2
TRNP	TNNI1
TRNP1	TNNI1.2.L
TRNQ	TNNI2
TRNS1	TNNI2A.2
TRNT	TNNI2A.4
TRNT1	TNNI2B.1
TRNV	TNNI3
TRNY	TNNI3K
TRO	TNNT1
TROAP	TNNT2
TROAP-AS1	TNNT3
TRP53	TNNT3A
TRP53BP1	TNNT3B
TRP53BP2	TNP1
TRP53I11	TNP2

TRP53INP1	TNPO1
TRP53INP2	TNPO2
TRP53TG5	TNPO3
TRP63	TNR
TRP73	TNRC18
TRPA1	TNRC6A
TRPC1	TNRC6B
TRPC2	TNRC6C
TRPC3	TNS1
TRPC4	TNS2
TRPC5	TNS3
TRPC6	TNS4
TRPM1	TNXB
TRPM2	TO
TRPM3	TOB1
TRPM4	TOB1-AS1
TRPM4A	TOB2
TRPM5	TOB2P1
TRPM6	TOE1
TRPM7	TOGARAM1
TRPM8	TOGARAM2
TRPS1	TOLL-9
TRPT1	TOLLIP
TRPV1	TOLLIP-DT
TRPV2	TOM1
TRPV3	TOM1L1
TRPV4	TOM1L2
TRPV5	TOMM20
TRPV6	TOMM22
TRRAP	TOMM34
TRUB1	TOMM40
TRUB2	TOMM40L
TRV-CAC4-1	TOMM5
TRXR-1	TOMM6
TSACC	TOMM7
TSC1	TOMM70

TSC1A	TOMM70A
TSC1B	TONSL
TSC2	TONSL-AS1
TSC22D1	TOP1
TSC22D1-AS1	TOP1MT
TSC22D2	TOP1P1
TSC22D3	TOP1P2
TSC22D4	TOP2
TSEN15	TOP2A
TSEN2	TOP2B
TSEN34	TOP3A
TSEN54	TOP3B
TSFM	TOPBP1
TSG1	TOPORS
TSG101	TOR1A
TSGA10	TOR1AIP1
TSGA10IP	TOR1AIP2
TSGA13	TOR1B
TSHB	TOR2A
TSHR	TOR3A
TSHZ1	TOR4A
TSHZ2	TOX
TSHZ3	TOX2
TSKS	TOX3
TSKU	TOX4
TSLP	TP53
TSN	TP53AIP1
TSNARE1	TP53BP1
TSNAX	TP53BP2
TSNAXIP1	TP53COR1
TSP1	TP53I11
TSPAN1	TP53I13
TSPAN10	TP53I3
TSPAN11	TP53INP1
TSPAN12	TP53INP2
TSPAN13	TP53TG1

TSPAN13B	TP53TG3
TSPAN14	TP53TG3B
TSPAN15	TP53TG5
TSPAN17	TP63
TSPAN18	TP73
TSPAN19	TP73-AS1
TSPAN2	TPBG
TSPAN3	TPBPA
TSPAN31	TPCN1
TSPAN32	TPCN2
TSPAN33	TPD52
TSPAN34	TPD52L1
TSPAN4	TPD52L2
TSPAN5	TPGS1
TSPAN6	TPGS2
TSPAN7	TPH1
TSPAN8	TPH2
TSPAN9	TPI
TSPEAR	TPI1
TSPEAR-AS2	TPI1A
TSPO	TPI1P2
TSPO2	TPK1
TSPOAP1	TPM1
TSPOAP1-AS1	TPM2
TSPY2	TPM3
TSPY26P	TPM3P9
TSPYL1	TPM4
TSPYL2	TPM4A
TSPYL3	TPMA
TSPYL4	TPMT
TSPYL5	TPMT.2
TSPYL6	TPO
TSR1	TPP1
TSR2	TPP2
TSR3	TPPP
TSSC4	TPPP2

TSSK1B	TPPP3
TSSK2	TPR
TSSK3	TPRA1
TSSK6	TPRG1
TST	TPRG1L
TSTD1	TPRKB
TSTD2	TPRN
TSTD3	TPRXL
TSX	TPSAB1
TTBK1	TPSB2
TTBK2	TPSD1
TTBK-2	TPSG1
TTC1	TPST1
TTC12	TPST2
TTC13	TPT1
TTC14	TPT1-AS1
TTC16	TPT1P8
TTC17	TPT1P9
TTC19	TPTE
TTC21A	TPTE2
TTC21B	TPTE2P1
TTC22	TPTE2P2
TTC23	TPTE2P5
TTC23L	TPTE2P6
TTC24	TPTEP1
TTC26	TPX2
TTC27	TRA
TTC28	TRA2A
TTC28-AS1	TRA2B
TTC29	TRABD
TTC3	TRABD2A
TTC30A	TRABD2B
TTC30A1	TRAC
TTC30B	TRADD
TTC31	TRAF1
TTC32	TRAF2

TTC33	TRAF3
TTC34	TRAF3IP1
TTC35	TRAF3IP2
TTC36	TRAF3IP2-AS1
TTC38	TRAF3IP3
TTC39A	TRAF4
TTC39B	TRAF5
TTC39C	TRAF6
TTC3P1	TRAF7
TTC4	TRAFD1
TTC41	TRAIP
TTC5	TRAK1
TTC6	TRAK2
TTC7	TRAM1
TTC7A	TRAM1L1
TTC7B	TRAM2
TTC8	TRAM2-AS1
TTC9	TRANK1
TTC9B	TRAP1
TTC9C	TRAPPC1
TTC9-DT	TRAPPC10
TTF1	TRAPPC11
TTF2	TRAPPC12
TTI1	TRAPPC13
TTI2	TRAPPC14
TTK	TRAPPC2
TTL	TRAPPC2B
TTLL1	TRAPPC2L
TTLL10	TRAPPC3
TTLL10-AS1	TRAPPC4
TTLL11	TRAPPC5
TTLL12	TRAPPC6A
TTLL2	TRAPPC6B
TTLL3	TRAPPC8
TTLL4	TRAPPC9
TTLL5	TRARG1

TTLL6	TRAT1
TTLL7	TRAV13-2
TTLL8	TRAV4D-4
TTLL9	TRAV7-4
TTN	TRAV8-3
TTN.1	TRAV9D-3
TTN.2	TRBC1
TTN-AS1	TRBC2
TTPA	TRBL
TTPAL	TRBV20OR9-2
TTR	TRBV5-4
TTTY13	TRD
TTTY14	TRD-GTC10-1
TTTY23	TRDMT1
TTTY23B	TRDN
TTTY4B	TRDV3
TTTY4C	TRE-CTC1-6
TTX-1	TREH
TTYH1	TREM1
TTYH2	TREM2
TTYH3	TREM3
TUB	TREML1
TUBA1A	TREML2
TUBA1B	TREML4
TUBA1C	TRERF1
TUBA2	TRE-TTC3-1
TUBA3C	TREX1
TUBA3D	TREX2
TUBA3E	TRF
TUBA3FP	TRF-GAA1-2
TUBA4A	TRG-AS1
TUBA4B	TRGC2
TUBA8	TRGV5
TUBAL3	TRGV9
TUBAP2	TRH
TUBB	TRHDE

TUBB1	TRHDE-AS1
TUBB2A	TRHR
TUBB2B	TRIAP1
TUBB3	TRIB1
TUBB4A	TRIB2
TUBB4B	TRIB3
TUBB5	TRIL
TUBB6	TRIM10
TUBB7P	TRIM11
TUBB8	TRIM13
TUBBP1	TRIM14
TUBBP2	TRIM15
TUBBP5	TRIM16
TUBD1	TRIM16L
TUBE1	TRIM17
TUBG1	TRIM2
TUBG2	TRIM21
TUBGCP2	TRIM22
TUBGCP3	TRIM23
TUBGCP4	TRIM24
TUBGCP6	TRIM25
TUFM	TRIM25L
TUFT1	TRIM26
TUG1	TRIM27
TULP2	TRIM28
TULP3	TRIM29
TULP4	TRIM3
TUSC1	TRIM30B
TUSC2	TRIM30D
TUSC3	TRIM31
TUSC7	TRIM32
TUT1	TRIM33
TUT4	TRIM34
TUT7	TRIM35
TVP23A	TRIM36
TVP23B	TRIM37

TVP23C	TRIM38
TWF1	TRIM39
TWF2	TRIM4
TWIST1	TRIM41
TWIST2	TRIM43
TWNK	TRIM44
TWSG1	TRIM45
TXK	TRIM46
TXLNA	TRIM47
TXLNB	TRIM5
TXLNBB	TRIM50
TXLNG	TRIM52-AS1
TXLNGY	TRIM53AP
TXN	TRIM54
TXN1	TRIM55
TXN2	TRIM55A
TXNDC11	TRIM56
TXNDC12	TRIM59
TXNDC15	TRIM6
TXNDC16	TRIM60
TXNDC17	TRIM61
TXNDC2	TRIM62
TXNDC5	TRIM63
TXNDC8	TRIM64
TXNDC9	TRIM65
TXNIP	TRIM66
TXNL1	TRIM67
TXNL4A	TRIM68
TXNL4B	TRIM69
TXNRD1	TRIM6-TRIM34
TXNRD2	TRIM7
TXNRD3	TRIM71
TYK2	TRIM72
TYMP	TRIM73
TYMS	TRIM74
TYMSOS	TRIM8

TYR	TRIM80
TYRO3	TRIM9
TYRO3P	TRIML1
TYROBP	TRIML2
TYRP1	TRIO
TYSND1	TRIOBP
TYW1	TRIP10
TYW3	TRIP11
TYW5	TRIP12
U2AF1	TRIP13
U2AF1L4	TRIP4
U2AF2	TRIP6
U2SURP	TRIQK
U90926	TRIR
UACA	TRIT1
UAP1	TRL-CAG2-1
UAP1L1	TRMO
UBA1	TRMT1
UBA1.L	TRMT10A
UBA1Y	TRMT10B
UBA2	TRMT10C
UBA3	TRMT11
UBA5	TRMT112
UBA52	TRMT12
UBA6	TRMT13
UBA6-DT	TRMT1L
UBA7	TRMT2A
UBAC1	TRMT2B
UBAC2	TRMT44
UBALD1	TRMT5
UBALD2	TRMT6
UBAP1	TRMT61A
UBAP1L	TRMT61B
UBAP2	TRMT9B
UBAP2L	TRMU
UBASH3A	TRNA

UBASH3B	TRNAU1AP
UBB	TRNC
UBBP4	TRND
UBC	TRNG
UBD	TRNI
UBE2A	TRNL1
UBE2B	TRNM
UBE2C	TRNN
UBE2D1	TRNP
UBE2D2	TRNP1
UBE2D3	TRNQ
UBE2D3-AS1	TRNS1
UBE2D4	TRNT
UBE2E1	TRNT1
UBE2E2	TRNV
UBE2E3	TRNY
UBE2F	TRO
UBE2G1	TROAP
UBE2G2	TROAP-AS1
UBE2H	TRP53
UBE2I	TRP53BP1
UBE2J1	TRP53BP2
UBE2J2	TRP53I11
UBE2K	TRP53INP1
UBE2L3	TRP53INP2
UBE2L6	TRP53TG5
UBE2M	TRP63
UBE2MP1	TRP73
UBE2N	TRPA1
UBE2NL	TRPC1
UBE2O	TRPC2
UBE2Q1	TRPC3
UBE2Q2	TRPC4
UBE2Q2P1	TRPC5
UBE2QL1	TRPC6
UBE2R2	TRPM1

UBE2S	TRPM2
UBE2T	TRPM2-AS
UBE2U	TRPM3
UBE2V1	TRPM4
UBE2V2	TRPM4A
UBE2W	TRPM5
UBE2Z	TRPM6
UBE3A	TRPM7
UBE3B	TRPM8
UBE3C	TRPS1
UBE3D	TRPT1
UBE4A	TRPV1
UBE4B	TRPV2
UBFD1	TRPV3
UBIAD1	TRPV4
UBI-P63E	TRPV5
UBL3	TRPV6
UBL4A	TRRAP
UBL4B	TRUB1
UBL7	TRUB2
UBL7-DT	TRV-CAC4-1
UBLCP1	TRXR-1
UBN1	TSACC
UBN2	TSC1
UBOX5	TSC1A
UBP1	TSC1B
UBQLN1	TSC2
UBQLN2	TSC22D1
UBQLN3	TSC22D1-AS1
UBQLN4	TSC22D2
UBQLNL	TSC22D3
UBR1	TSC22D4
UBR2	TSEN15
UBR3	TSEN2
UBR4	TSEN34
UBR5	TSEN54

UBR7	TSFM
UBTD1	TSG1
UBTD2	TSG101
UBTF	TSGA10
UBTF.L	TSGA10IP
UBTFL	TSGA13
UBXD2	TSHB
UBXN1	TSHR
UBXN10	TSHZ1
UBXN11	TSHZ2
UBXN2A	TSHZ3
UBXN2B	TSIX
UBXN4	TSKS
UBXN6	TSKU
UBXN7	TSLP
UBXN8	TSN
UCA1	TSNARE1
UCHL1	TSNAX
UCHL3	TSNAXIP1
UCHL3-PS1	TSP1
UCHL4	TSPAN1
UCHL5	TSPAN10
UCK1	TSPAN11
UCK2	TSPAN12
UCK2B	TSPAN13
UCKL1	TSPAN13B
UCKL1-AS1	TSPAN14
UCMA	TSPAN15
UCN	TSPAN17
UCN2	TSPAN18
UCN3	TSPAN19
UCP1	TSPAN2
UCP2	TSPAN3
UCP3	TSPAN31
UEVLD	TSPAN32
UFC1	TSPAN33

UFD1	TSPAN34
UFD1L	TSPAN4
UFL1	TSPAN5
UFM1	TSPAN6
UFSP1	TSPAN7
UFSP2	TSPAN8
UGCG	TSPAN9
UGDH	TSPEAR
UGDH-AS1	TSPEAR-AS2
UGGT1	TSPO
UGGT2	TSPO2
UGP2	TSPOAP1
UGT1A	TSPOAP1-AS1
UGT1A1	TSPY2
UGT1A10	TSPY26P
UGT1A3	TSPYL1
UGT1A4	TSPYL2
UGT1A5	TSPYL3
UGT1A6	TSPYL4
UGT1A6A	TSPYL5
UGT1A7	TSPYL6
UGT1A8	TSR1
UGT1A9	TSR2
UGT1B1	TSR3
UGT2A1	TSSC4
UGT2A2	TSSK1B
UGT2A3	TSSK2
UGT2A4	TSSK3
UGT2B	TSSK6
UGT2B1	TST
UGT2B11	TSTD1
UGT2B15	TSTD2
UGT2B17	TSTD3
UGT2B28	TSX
UGT2B3	TTBK1
UGT2B34	TTBK2

UGT2B36	TTBK-2
UGT2B37	TTC1
UGT2B38	TTC12
UGT2B5	TTC13
UGT2B7	TTC14
UGT303A1	TTC16
UGT-31	TTC17
UGT35B1	TTC19
UGT3A1	TTC21A
UGT3A2	TTC21B
UGT-48	TTC22
UGT-53	TTC23
UGT5A1	TTC23L
UGT5A2	TTC24
UGT8	TTC26
UHMK1	TTC27
UHRF1	TTC28
UHRF2	TTC28-AS1
UICLM	TTC29
UIMC1	TTC3
UK114	TTC30A
ULBP1	TTC30A1
ULBP2	TTC30B
ULBP3	TTC31
ULE-3	TTC32
ULK1	TTC33
ULK2	TTC34
ULK3	TTC35
ULK4	TTC36
ULK4P1	TTC38
ULK4P2	TTC39A
ULK4P3	TTC39B
UMAD1	TTC39C
UMOD	TTC3P1
UMODL1	TTC4
UMODL1-AS1	TTC41

UMPS	TTC5
UNC-103	TTC6
UNC119	TTC7
UNC119B	TTC7A
UNC13A	TTC7B
UNC13B	TTC8
UNC13C	TTC9
UNC13D	TTC9B
UNC-29	TTC9C
UNC-38	TTC9-DT
UNC45A	TTF1
UNC45B	TTF2
UNC50	TTI1
UNC-50	TTI2
UNC5A	TTK
UNC5B	TTL
UNC5C	TTLL1
UNC5CL	TTLL10
UNC5D	TTLL10-AS1
UNC-63	TTLL11
UNC-68	TTLL12
UNC79	TTLL2
UNC80	TTLL3
UNC93A	TTLL4
UNC93B1	TTLL5
UNCX	TTLL6
UNG	TTLL7
UNG-1	TTLL8
UNK	TTLL9
UNKL	TTN
UOX	TTN.1
UPB1	TTN.2
UPF1	TTN-AS1
UPF2	TTPA
UPF3A	TTPAL
UPF3B	TTR

UPK1A	TTTY13
UPK1B	TTTY14
UPK2	TTTY15
UPK3A	TTTY23
UPK3B	TTTY23B
UPK3BL	TTTY4B
UPP1	TTTY4C
UPP2	TTX-1
UPRT	TTYH1
UQCC	TTYH2
UQCC1	TTYH3
UQCC2	TUB
UQCR	TUBA1A
UQCR10	TUBA1B
UQCR11	TUBA1C
UQCR2	TUBA2
UQCRB	TUBA3C
UQCRBP1	TUBA3D
UQCRC1	TUBA3E
UQCRC2	TUBA3FP
UQCRFS1	TUBA4A
UQCRH	TUBA4B
UQCRHL	TUBA8
UQCRQ	TUBAL3
URB1	TUBAP2
URB1-AS1	TUBB
URB2	TUBB1
URGCP	TUBB2A
URGCP-MRPS24	TUBB2B
URI1	TUBB3
URM1	TUBB4A
UROC1	TUBB4B
UROD	TUBB5
UROS	TUBB6
USB1	TUBB7P
USE1	TUBB8

USF1	TUBBP1
USF2	TUBBP2
USF3	TUBBP5
USH1C	TUBD1
USH1G	TUBE1
USH2A	TUBG1
USHBP1	TUBG2
USO1	TUBGCP2
USP1	TUBGCP3
USP10	TUBGCP4
USP11	TUBGCP6
USP12	TUFM
USP13	TUFT1
USP14	TUG1
USP15	TULP2
USP16	TULP3
USP17L2	TULP4
USP17L30	TUSC1
USP17LD	TUSC2
USP17LE	TUSC3
USP18	TUSC7
USP2	TUT1
USP21	TUT4
USP22	TUT7
USP24	TVP23A
USP25	TVP23B
USP27X	TVP23C
USP27X-DT	TWF1
USP28	TWF2
USP29	TWIST1
USP3	TWIST2
USP30	TWNK
USP31	TWSG1
USP32	TXK
USP32P1	TXLNA
USP32P2	TXLNB

USP33	TXLNBB
USP34	TXLNG
USP35	TXLNGY
USP36	TXN
USP37	TXN1
USP38	TXN2
USP39	TXNDC11
USP3-AS1	TXNDC12
USP4	TXNDC15
USP40	TXNDC16
USP42	TXNDC17
USP43	TXNDC2
USP44	TXNDC5
USP45	TXNDC8
USP46	TXNDC9
USP47	TXNIP
USP48	TXNL1
USP49	TXNL4A
USP5	TXNL4B
USP5.S	TXNRD1
USP51	TXNRD2
USP53	TXNRD3
USP54	TYK2
USP6	TYMP
USP6NL	TYMS
USP7	TYMSOS
USP8	TYR
USP9X	TYRO3
USPL1	TYRO3P
UST	TYROBP
UST4R	TYRP1
UTF1	TYSND1
UTP11	TYW1
UTP14A	TYW3
UTP14B	TYW5
UTP14C	U2AF1

UTP15	U2AF1L4
UTP18	U2AF2
UTP20	U2SURP
UTP23	U90926
UTP25	UACA
UTP3	UAP1
UTP4	UAP1L1
UTP6	UBA1
UTRN	UBA1.L
UTS2	UBA1Y
UTS2B	UBA2
UTS2R	UBA3
UTY	UBA5
UVRAG	UBA52
UVRAG-DT	UBA6
UVSSA	UBA6-DT
UXS1	UBA7
UXT	UBAC1
UXT-AS1	UBAC2
V	UBALD1
VAC14	UBALD2
VAMP1	UBAP1
VAMP2	UBAP1L
VAMP2.S	UBAP2
VAMP3	UBAP2L
VAMP4	UBASH3A
VAMP5	UBASH3B
VAMP7	UBB
VAMP8	UBBP4
VANGL1	UBC
VANGL2	UBD
VANGL2.L	UBE2A
VAPA	UBE2B
VAPB	UBE2C
VARS1	UBE2D1
VARS2	UBE2D2

VASH1	UBE2D3
VASH1-AS1	UBE2D3-AS1
VASH2	UBE2D4
VASN	UBE2E1
VASP	UBE2E2
VAT1	UBE2E3
VAT1L	UBE2F
VAV1	UBE2G1
VAV2	UBE2G2
VAV3	UBE2H
VAX1	UBE2I
VAX2	UBE2J1
VBP1	UBE2J2
VCAM1	UBE2K
VCAN	UBE2L3
VCL	UBE2L6
VCP	UBE2M
VCPIP1	UBE2MP1
VCPKMT	UBE2N
VCX	UBE2NL
VCX2	UBE2O
VCX3A	UBE2Q1
VCY	UBE2Q2
VDAC1	UBE2Q2P1
VDAC1P1	UBE2QL1
VDAC2	UBE2R2
VDAC3	UBE2S
VDR	UBE2T
VED	UBE2U
VEGFA	UBE2V1
VEGFAA	UBE2V2
VEGFAB	UBE2W
VEGFB	UBE2Z
VEGFC	UBE3A
VEGFD	UBE3B
VEGT.L	UBE3C

VENTX	UBE3D
VENTX1.1	UBE4A
VENTX2.1.L	UBE4B
VENTX2.1.S	UBFD1
VENTX2.2.L	UBIAD1
VENTX3.2.S	UBI-P63E
VENTXP1	UBL3
VENTXP7	UBL4A
VEPH1	UBL4B
VEZF1	UBL7
VEZT	UBL7-DT
VGF	UBLCP1
VGLL1	UBN1
VGLL2	UBN2
VGLL3	UBOX5
VGLL4	UBP1
VHL	UBQLN1
VHLL	UBQLN2
VIAAT	UBQLN3
VIL1	UBQLN4
VILL	UBQLNL
VIM	UBR1
VIM-AS1	UBR2
VIP	UBR3
VIPAS39	UBR4
VIPR1	UBR5
VIPR2	UBR7
VIRMA	UBTD1
VIT	UBTD2
VKORC1	UBTF
VKORC1L1	UBTF.L
VLDLR	UBTFL
VLDLR-AS1	UBXD2
VMA21	UBXN1
VMAC	UBXN10
VMN1R185	UBXN11

VMN1R201	UBXN2A
VMN1R205	UBXN2B
VMN1R212	UBXN4
VMN1R227	UBXN6
VMN1R232	UBXN7
VMN1R26	UBXN8
VMN1R27	UCA1
VMN1R28	UCHL1
VMN1R43	UCHL3
VMN1R49	UCHL3-PS1
VMN1R52	UCHL4
VMN1R58	UCHL5
VMN1R69	UCK1
VMN1R71	UCK2
VMN1R72	UCK2B
VMN1R80	UCKL1
VMN1R83	UCKL1-AS1
VMN1R-PS8	UCMA
VMN2R10	UCN
VMN2R107	UCN2
VMN2R20	UCN3
VMN2R29	UCP1
VMN2R34	UCP2
VMN2R37	UCP3
VMN2R42	UEVLD
VMN2R43	UFC1
VMN2R50	UFD1
VMN2R51	UFD1L
VMN2R-PS54	UFL1
VMO1	UFM1
VMP1	UFSP1
VN1R1	UFSP2
VN1R10P	UGCG
VN1R3	UGDH
VNN1	UGDH-AS1
VNN2	UGGT1

VNN3	UGGT2
VNN3P	UGP2
VOF16	UGT1A
VOM1R90	UGT1A1
VOM2R44	UGT1A10
VOM2R-PS45	UGT1A3
VOPP1	UGT1A4
VOX	UGT1A5
VPREB1	UGT1A6
VPREB2	UGT1A6A
VPREB3	UGT1A7
VPS11	UGT1A8
VPS13A	UGT1A9
VPS13B	UGT1B1
VPS13C	UGT2A1
VPS13D	UGT2A2
VPS16	UGT2A3
VPS18	UGT2A4
VPS24	UGT2B
VPS25	UGT2B1
VPS26A	UGT2B11
VPS26B	UGT2B15
VPS26C	UGT2B17
VPS28	UGT2B28
VPS29	UGT2B3
VPS33A	UGT2B34
VPS33B	UGT2B36
VPS35	UGT2B37
VPS35L	UGT2B38
VPS36	UGT2B5
VPS37A	UGT2B7
VPS37B	UGT303A1
VPS37C	UGT-31
VPS37D	UGT35B1
VPS41	UGT3A1
VPS45	UGT3A2

VPS4A	UGT-48
VPS4B	UGT-53
VPS50	UGT5A1
VPS51	UGT5A2
VPS52	UGT8
VPS53	UHMK1
VPS54	UHRF1
VPS72	UHRF2
VPS8	UICLM
VPS9D1	UIMC1
VRK1	UK114
VRK2	ULBP1
VRK3	ULBP2
VRTN	ULBP3
VSIG1	ULE-3
VSIG10	ULK1
VSIG10L	ULK2
VSIG2	ULK3
VSIG4	ULK4
VSIG8	ULK4P1
VSIR	ULK4P2
VSNL1	ULK4P3
VSTM1	UMAD1
VSTM2A	UMOD
VSTM2L	UMODL1
VSTM4	UMODL1-AS1
VSX1	UMPS
VSX2	UNC-103
VTA1	UNC119
VTCN1	UNC119B
VTG1	UNC13A
VTG2	UNC13B
VTI1A	UNC13C
VTI1B	UNC13D
VTN	UNC-29
VTNB	UNC-38

VTRNA1-1	UNC45A
VTRNA1-2	UNC45B
VTRNA1-3	UNC50
VTRNA2-1	UNC-50
VWA1	UNC5A
VWA2	UNC5B
VWA3A	UNC5C
VWA3B	UNC5CL
VWA5A	UNC5D
VWA5B2	UNC-63
VWA7	UNC-68
VWA8	UNC79
VWC2	UNC80
VWC2L	UNC93A
VWCE	UNC93B1
VWDE	UNCX
VWF	UNG
VXN	UNG-1
W03D8.9	UNK
W03F9.4	UNKL
W06H8.12	UOX
W06H8.2	UPB1
WAC	UPF1
WAKMAR2	UPF2
WAPL	UPF3A
WARS1	UPF3B
WARS2	UPK1A
WAS	UPK1B
WASF1	UPK2
WASF2	UPK3A
WASF3	UPK3B
WASH2P	UPK3BL
WASH3P	UPP1
WASHC1	UPP2
WASHC2A	UPRT
WASHC2C	UQCC

WASHC3	UQCC1
WASHC4	UQCC2
WASHC5	UQCR
WASIR2	UQCR10
WASL	UQCR11
WBP1	UQCR2
WBP11	UQCRB
WBP11P1	UQCRBP1
WBP1L	UQCRC1
WBP2	UQCRC2
WBP2NL	UQCRFS1
WBP4	UQCRH
WDCP	UQCRHL
WDFY1	UQCRQ
WDFY2	URB1
WDFY3	URB1-AS1
WDFY3-AS2	URB2
WDFY4	URGCP
WDHD1	URGCP-MRPS24
WDPCP	URI1
WDR1	URM1
WDR11	UROC1
WDR12	UROD
WDR13	UROS
WDR17	USB1
WDR18	USE1
WDR19	USF1
WDR20	USF2
WDR24	USF3
WDR25	USH1C
WDR26	USH1G
WDR27	USH2A
WDR3	USHBP1
WDR31	USO1
WDR33	USP1
WDR35	USP10

WDR36	USP11
WDR37	USP12
WDR38	USP13
WDR4	USP14
WDR41	USP15
WDR43	USP16
WDR44	USP17L2
WDR45	USP17L24
WDR45B	USP17L25
WDR46	USP17L26
WDR47	USP17L27
WDR48	USP17L28
WDR49	USP17L29
WDR5	USP17L30
WDR52	USP17L9P
WDR53	USP17LD
WDR54	USP17LE
WDR55	USP18
WDR59	USP2
WDR5B	USP21
WDR6	USP22
WDR62	USP24
WDR63	USP25
WDR64	USP27X
WDR67	USP27X-DT
WDR7	USP28
WDR70	USP29
WDR72	USP3
WDR73	USP30
WDR74	USP31
WDR75	USP32
WDR76	USP32P1
WDR77	USP32P2
WDR81	USP33
WDR82	USP34
WDR83	USP35

WDR83OS	USP36
WDR86	USP37
WDR86-AS1	USP38
WDR87	USP39
WDR88	USP3-AS1
WDR89	USP4
WDR90	USP40
WDR91	USP42
WDR93	USP43
WDR97	USP44
WDSUB1	USP45
WDTC1	USP46
WEE1	USP47
WEE1.S	USP48
WEE2	USP49
WEE2-AS1	USP5
WFDC1	USP5.S
WFDC11	USP51
WFDC12	USP53
WFDC13	USP54
WFDC15A	USP6
WFDC15B	USP6NL
WFDC17	USP7
WFDC18	USP8
WFDC2	USP9X
WFDC21	USPL1
WFDC21P	UST
WFDC3	UST4R
WFDC5	UTF1
WFDC6	UTP11
WFDC6A	UTP14A
WFDC8	UTP14B
WFIKKN1	UTP14C
WFIKKN2	UTP15
WFS1	UTP18
WHAMM	UTP20

WHAMMP1	UTP23
WHAMMP2	UTP25
WHAMMP3	UTP3
WHRN	UTP4
WIBG	UTP6
WIF1	UTRN
WIPF1	UTS2
WIPF2	UTS2B
WIPF3	UTS2R
WIPI1	UTY
WIPI2	UVRAG
WISP1	UVRAG-DT
WIZ	UVSSA
WLS	UXS1
WNK1	UXT
WNK2	UXT-AS1
WNK3	V
WNK4	VAC14
WNT1	VAMP1
WNT10A	VAMP2
WNT10B	VAMP2.S
WNT11	VAMP3
WNT16	VAMP4
WNT2	VAMP5
WNT2B	VAMP7
WNT3	VAMP8
WNT3A	VANGL1
WNT4	VANGL2
WNT5A	VANGL2.L
WNT5B	VAPA
WNT6	VAPB
WNT7A	VARS1
WNT7B	VARS2
WNT8A	VASH1
WNT8B	VASH1-AS1
WNT9A	VASH2

WNT9B	VASN
WRAP53	VASP
WRAP73	VAT1
WRN	VAT1L
WRNIP1	VAV1
WSB1	VAV2
WSB2	VAV3
WSCD1	VAX1
WSCD2	VAX2
WT1	VBP1
WT1-AS	VCAM1
WTAP	VCAN
WTIP	VCL
WWC1	VCP
WWC2	VCPIP1
WWC2-AS2	VCPKMT
WWC3	VCX
WWOX	VCX2
WWP1	VCX3A
WWP1-AS1	VCY
WWP2	VDAC1
WWTR1	VDAC1P1
WWTR1-AS1	VDAC2
XAB2	VDAC3
XAF1	VDR
XAGE1A	VED
XAGE1B	VEGFA
XAGE2	VEGFAA
XAGE5	VEGFAB
XB5922676.S	VEGFB
XBP1	VEGFC
XCL1	VEGFD
XCL2	VEGT.L
XCR1	VENTX
XDH	VENTX1.1
XENF.L	VENTX2.1.L

XEPSIN.L	VENTX2.1.S
XG	VENTX2.2.L
XGB	VENTX3.2.S
XIAP	VENTXP1
XIRP1	VENTXP7
XIRP2	VEPH1
XIRP2A	VEZF1
XIST	VEZT
XK	VGF
XKR4	VGLL1
XKR5	VGLL2
XKR6	VGLL3
XKR7	VGLL4
XKR8	VHL
XKRX	VHLL
XLKD1	VIAAT
XLR3A	VIL1
XLR3B	VILL
XPA	VIM
XPC	VIM-AS1
XPNPEP1	VIP
XPNPEP2	VIPAS39
XPNPEP3	VIPR1
XPO1	VIPR2
XPO4	VIRMA
XPO5	VIT
XPO6	VKORC1
XPO7	VKORC1L1
XPOT	VLDLR
XPR1	VLDLR-AS1
XRCC1	VMA21
XRCC2	VMAC
XRCC3	VMN1R185
XRCC4	VMN1R201
XRCC5	VMN1R205
XRCC6	VMN1R212

XRCC6BP1	VMN1R227
XRN1	VMN1R232
XRN2	VMN1R26
XRRA1	VMN1R27
XXYLT1	VMN1R28
XYLB	VMN1R43
XYLT1	VMN1R49
XYLT2	VMN1R52
Y1A5A.1	VMN1R58
Y24D9A.9	VMN1R69
Y37H2A.18	VMN1R71
Y37H2A.7	VMN1R72
Y43F8A.2	VMN1R80
Y47G7B.2	VMN1R83
Y51B9A.8	VMN1R-PS8
Y54G2A.48	VMN2R10
Y57G11A.2	VMN2R107
Y57G11A.5	VMN2R20
Y59E9AR.1	VMN2R29
Y60A9.3	VMN2R34
Y69E1A.1	VMN2R37
Y7A5A.10	VMN2R42
YAE1	VMN2R43
YAF2	VMN2R50
YAP1	VMN2R51
YARS	VMN2R-PS54
YARS1	VMO1
YARS2	VMP1
YBEY	VN1R1
YBX1	VN1R10P
YBX2	VN1R17P
YBX3	VN1R3
YBX3P1	VNN1
YDJC	VNN2
YEATS2	VNN3
YEATS4	VNN3P

YES1	VOF16
YIF1A	VOM1R90
YIF1B	VOM2R44
YIPF1	VOM2R-PS45
YIPF2	VOPP1
YIPF3	VOX
YIPF4	VPREB1
YIPF5	VPREB2
YIPF6	VPREB3
YIPF7	VPS11
YJEFN3	VPS13A
YJU2	VPS13B
YKT6	VPS13C
YLPM1	VPS13D
YME1L1	VPS16
YOD1	VPS18
YPEL1	VPS24
YPEL2	VPS25
YPEL3	VPS26A
YPEL4	VPS26B
YPEL5	VPS26C
YRDC	VPS28
YTHDC1	VPS29
YTHDC2	VPS33A
YTHDF1	VPS33B
YTHDF2	VPS35
YTHDF3	VPS35L
YTHDF3-DT	VPS36
YWHAB	VPS37A
YWHAE	VPS37B
YWHAE1	VPS37C
YWHAG	VPS37D
YWHAG2	VPS41
YWHAH	VPS45
YWHAQ	VPS4A
YWHAQA	VPS4B

YWHAZ	VPS50
YY1	VPS51
YY1AP1	VPS52
YY1P2	VPS53
YY2	VPS54
ZADH2	VPS72
ZAN	VPS8
ZAP70	VPS9D1
ZAR1	VPS9D1-AS1
ZAR1L	VRK1
ZBBX	VRK2
ZBED1	VRK3
ZBED10P	VRTN
ZBED2	VSIG1
ZBED3	VSIG10
ZBED4	VSIG10L
ZBED5	VSIG2
ZBED5-AS1	VSIG4
ZBED6	VSIG8
ZBED8	VSIR
ZBED9	VSNL1
ZBP1	VSTM1
ZBTB1	VSTM2A
ZBTB10	VSTM2L
ZBTB11	VSTM4
ZBTB11-AS1	VSX1
ZBTB12	VSX2
ZBTB14	VTA1
ZBTB16	VTCN1
ZBTB17	VTG1
ZBTB18	VTG2
ZBTB2	VTI1A
ZBTB20	VTI1B
ZBTB20-AS1	VTN
ZBTB21	VTNB
ZBTB22	VTRNA1-1

ZBTB24	VTRNA1-2
ZBTB25	VTRNA1-3
ZBTB26	VTRNA2-1
ZBTB2A	VWA1
ZBTB3	VWA2
ZBTB32	VWA3A
ZBTB33	VWA3B
ZBTB34	VWA5A
ZBTB37	VWA5B2
ZBTB38	VWA7
ZBTB39	VWA8
ZBTB4	VWC2
ZBTB40	VWC2L
ZBTB41	VWCE
ZBTB42	VWDE
ZBTB43	VWF
ZBTB44	VXN
ZBTB45	W03D8.9
ZBTB46	W03F9.4
ZBTB47	W06H8.12
ZBTB48	W06H8.2
ZBTB49	WAC
ZBTB5	WAKMAR2
ZBTB6	WAPL
ZBTB7A	WARS1
ZBTB7B	WARS2
ZBTB7C	WAS
ZBTB8A	WASF1
ZBTB8B	WASF2
ZBTB8OS	WASF3
ZBTB9	WASH2P
ZC2HC1A	WASH3P
ZC2HC1C	WASHC1
ZC3H10	WASHC2A
ZC3H11A	WASHC2C
ZC3H12A	WASHC3

ZC3H12A-DT	WASHC4
ZC3H12B	WASHC5
ZC3H12C	WASIR2
ZC3H12D	WASL
ZC3H13	WBP1
ZC3H14	WBP11
ZC3H15	WBP11P1
ZC3H18	WBP1L
ZC3H3	WBP2
ZC3H4	WBP2NL
ZC3H6	WBP4
ZC3H7A	WDCP
ZC3H7B	WDFY1
ZC3H8	WDFY2
ZC3HAV1	WDFY3
ZC3HAV1L	WDFY3-AS2
ZC4H2	WDFY4
ZCCHC10	WDHD1
ZCCHC12	WDPCP
ZCCHC14	WDR1
ZCCHC17	WDR11
ZCCHC18	WDR12
ZCCHC2	WDR13
ZCCHC24	WDR17
ZCCHC3	WDR18
ZCCHC4	WDR19
ZCCHC6	WDR20
ZCCHC7	WDR24
ZCCHC8	WDR25
ZCCHC9	WDR26
ZCRB1	WDR27
ZCWPW1	WDR3
ZCWPW1L1	WDR31
ZCWPW2	WDR33
ZDBF2	WDR35
ZDHHC1	WDR36

ZDHHC11	WDR37
ZDHHC11B	WDR38
ZDHHC12	WDR4
ZDHHC13	WDR41
ZDHHC14	WDR43
ZDHHC15	WDR44
ZDHHC16	WDR45
ZDHHC17	WDR45B
ZDHHC18	WDR46
ZDHHC19	WDR47
ZDHHC2	WDR48
ZDHHC20	WDR49
ZDHHC21	WDR5
ZDHHC22	WDR52
ZDHHC23	WDR53
ZDHHC24	WDR54
ZDHHC3	WDR55
ZDHHC4	WDR59
ZDHHC5	WDR5B
ZDHHC6	WDR6
ZDHHC7	WDR62
ZDHHC8	WDR63
ZDHHC8P1	WDR64
ZDHHC9	WDR67
ZEB1	WDR7
ZEB1-AS1	WDR70
ZEB2	WDR72
ZEB2A	WDR73
ZEN	WDR74
ZER1	WDR75
ZFAND1	WDR76
ZFAND2A	WDR77
ZFAND2B	WDR81
ZFAND3	WDR82
ZFAND4	WDR83
ZFAND5	WDR83OS

ZFAND5A	WDR86
ZFAND5B	WDR86-AS1
ZFAND6	WDR87
ZFAS1	WDR88
ZFAT	WDR89
ZFAT-AS1	WDR90
ZFC3H1	WDR91
ZFHX2	WDR93
ZFHX3	WDR97
ZFHX4	WDSUB1
ZFHX4-AS1	WDTC1
ZFP1	WEE1
ZFP101	WEE1.S
ZFP105	WEE2
ZFP106	WEE2-AS1
ZFP11	WFDC1
ZFP110	WFDC11
ZFP112	WFDC12
ZFP119B	WFDC13
ZFP12	WFDC15A
ZFP125	WFDC15B
ZFP131	WFDC17
ZFP14	WFDC18
ZFP146	WFDC2
ZFP148	WFDC21
ZFP161	WFDC21P
ZFP174	WFDC3
ZFP180	WFDC5
ZFP182	WFDC6
ZFP189	WFDC6A
ZFP2	WFDC8
ZFP202	WFIKKN1
ZFP207	WFIKKN2
ZFP219	WFS1
ZFP235	WHAMM
ZFP236	WHAMMP1

ZFP239	WHAMMP2
ZFP24	WHAMMP3
ZFP26	WHRN
ZFP263	WIBG
ZFP266	WIF1
ZFP27	WIPF1
ZFP273	WIPF2
ZFP276	WIPF3
ZFP277	WIPI1
ZFP28	WIPI2
ZFP280D	WISP1
ZFP282	WIZ
ZFP286	WLS
ZFP287	WNK1
ZFP292	WNK2
ZFP296	WNK3
ZFP3	WNK4
ZFP30	WNT1
ZFP316	WNT10A
ZFP317	WNT10B
ZFP319	WNT11
ZFP326	WNT16
ZFP329	WNT2
ZFP330	WNT2B
ZFP33B	WNT3
ZFP345	WNT3A
ZFP346	WNT4
ZFP35	WNT5A
ZFP354A	WNT5B
ZFP354C	WNT6
ZFP36	WNT7A
ZFP365	WNT7B
ZFP366	WNT8A
ZFP367	WNT8B
ZFP369	WNT9A
ZFP36L1	WNT9B

ZFP36L2	WRAP53
ZFP37	WRAP73
ZFP385A	WRN
ZFP385B	WRNIP1
ZFP385C	WSB1
ZFP386	WSB2
ZFP389	WSCD1
ZFP40	WSCD2
ZFP407	WSPAR
ZFP41	WT1
ZFP418	WT1-AS
ZFP42	WTAP
ZFP420	WTIP
ZFP422-PS	WWC1
ZFP423	WWC2
ZFP426	WWC2-AS2
ZFP429	WWC3
ZFP433	WWOX
ZFP444	WWP1
ZFP445	WWP1-AS1
ZFP451	WWP2
ZFP454	WWTR1
ZFP455	WWTR1-AS1
ZFP459	XAB2
ZFP462	XAF1
ZFP467	XAGE1A
ZFP473	XAGE1B
ZFP493	XAGE2
ZFP51	XAGE5
ZFP518A	XB5922676.S
ZFP52	XBP1
ZFP521	XCL1
ZFP532	XCL2
ZFP536	XCR1
ZFP54	XDH
ZFP558	XENF.L

ZFP563	XEPSIN.L
ZFP566	XG
ZFP568	XGB
ZFP57	XIAP
ZFP59	XIRP1
ZFP592	XIRP2
ZFP593	XIRP2A
ZFP598	XIST
ZFP606	XK
ZFP607A	XKR4
ZFP608	XKR5
ZFP609	XKR6
ZFP612	XKR7
ZFP617	XKR8
ZFP62	XKRX
ZFP623	XLKD1
ZFP628	XLR3A
ZFP638	XLR3B
ZFP64	XPA
ZFP641	XPC
ZFP646	XPNPEP1
ZFP663	XPNPEP2
ZFP664	XPNPEP3
ZFP668	XPO1
ZFP687	XPO4
ZFP688	XPO5
ZFP689	XPO6
ZFP69	XPO7
ZFP697	XPOT
ZFP69B	XPR1
ZFP7	XRCC1
ZFP704	XRCC2
ZFP709	XRCC3
ZFP709L1	XRCC4
ZFP710	XRCC5
ZFP715	XRCC6

ZFP72	XRCC6BP1
ZFP740	XRCC6P5
ZFP747	XRN1
ZFP750	XRN2
ZFP758	XRRA1
ZFP759	XXYLT1
ZFP760	XYLB
ZFP763	XYLT1
ZFP772	XYLT2
ZFP775	Y1A5A.1
ZFP777	Y24D9A.9
ZFP780B	Y37H2A.18
ZFP781	Y37H2A.7
ZFP786	Y43F8A.2
ZFP787	Y47G7B.2
ZFP804B	Y51B9A.8
ZFP809	Y54G2A.48
ZFP81	Y57G11A.2
ZFP810	Y57G11A.5
ZFP819	Y59E9AR.1
ZFP82	Y60A9.3
ZFP820	Y69E1A.1
ZFP830	Y7A5A.10
ZFP831	YAE1
ZFP839	YAF2
ZFP84	YAP1
ZFP866	YARS
ZFP868	YARS1
ZFP871	YARS2
ZFP873	YBEY
ZFP874B	YBX1
ZFP882	YBX2
ZFP9	YBX3
ZFP90	YBX3P1
ZFP91	YDJC
ZFP91-CNTF	YEATS2

ZFP92	YEATS4
ZFP933	YES1
ZFP934	YIF1A
ZFP937	YIF1B
ZFP940	YIPF1
ZFP945	YIPF2
ZFP948	YIPF3
ZFP950	YIPF4
ZFP951	YIPF5
ZFP953	YIPF6
ZFP954	YIPF7
ZFP961	YJEFN3
ZFP982	YJU2
ZFPL1	YKT6
ZFPM1	YLPM1
ZFPM2	YME1L1
ZFR	YOD1
ZFR2	YPEL1
ZFTA	YPEL2
ZFTRAF1	YPEL3
ZFX	YPEL4
ZFY	YPEL5
ZFYVE1	YRDC
ZFYVE16	YTHDC1
ZFYVE19	YTHDC2
ZFYVE21	YTHDF1
ZFYVE26	YTHDF2
ZFYVE28	YTHDF3
ZFYVE9	YTHDF3-DT
ZG16	YWHAB
ZG16B	YWHAE
ZGLP1	YWHAE1
ZGPAT	YWHAG
ZGRF1	YWHAG2
ZHX1	YWHAH
ZHX1-C8ORF76	YWHAQ

ZHX2	YWHAQA
ZHX3	YWHAZ
ZIC1	YY1
ZIC2	YY1AP1
ZIC3	YY1P2
ZIC4	YY2
ZIC5	ZADH2
ZIC6	ZAN
ZIK1	ZAP70
ZIM1	ZAR1
ZIM2	ZAR1L
ZK637.18	ZBBX
ZKSCAN1	ZBED1
ZKSCAN14	ZBED10P
ZKSCAN2	ZBED2
ZKSCAN3	ZBED3
ZKSCAN4	ZBED4
ZKSCAN5	ZBED5
ZKSCAN6	ZBED5-AS1
ZKSCAN7	ZBED6
ZKSCAN8	ZBED8
ZKSCAN8P1	ZBED9
ZMAT1	ZBP1
ZMAT2	ZBTB1
ZMAT3	ZBTB10
ZMAT4	ZBTB11
ZMAT5	ZBTB11-AS1
ZMIZ1	ZBTB12
ZMIZ1-AS1	ZBTB14
ZMIZ2	ZBTB16
ZMPSTE24	ZBTB17
ZMYM1	ZBTB18
ZMYM2	ZBTB2
ZMYM3	ZBTB20
ZMYM4	ZBTB20-AS1
ZMYM5	ZBTB21

ZMYM6	ZBTB22
ZMYND10	ZBTB24
ZMYND11	ZBTB25
ZMYND12	ZBTB26
ZMYND15	ZBTB2A
ZMYND19	ZBTB3
ZMYND8	ZBTB32
ZNF10	ZBTB33
ZNF100	ZBTB34
ZNF101	ZBTB37
ZNF106	ZBTB38
ZNF107	ZBTB39
ZNF112	ZBTB4
ZNF114	ZBTB40
ZNF117	ZBTB41
ZNF12	ZBTB42
ZNF121	ZBTB43
ZNF124	ZBTB44
ZNF131	ZBTB45
ZNF132	ZBTB46
ZNF133	ZBTB47
ZNF134	ZBTB48
ZNF135	ZBTB49
ZNF136	ZBTB5
ZNF137P	ZBTB6
ZNF138	ZBTB7A
ZNF14	ZBTB7B
ZNF140	ZBTB7C
ZNF141	ZBTB8A
ZNF142	ZBTB8B
ZNF143	ZBTB8OS
ZNF146	ZBTB9
ZNF148	ZC2HC1A
ZNF155	ZC2HC1C
ZNF157	ZC3H10
ZNF16	ZC3H11A

ZNF160	ZC3H12A
ZNF165	ZC3H12A-DT
ZNF169	ZC3H12B
ZNF174	ZC3H12C
ZNF175	ZC3H12D
ZNF177	ZC3H13
ZNF18	ZC3H14
ZNF180	ZC3H15
ZNF181	ZC3H18
ZNF182	ZC3H3
ZNF184	ZC3H4
ZNF185	ZC3H6
ZNF189	ZC3H7A
ZNF19	ZC3H7B
ZNF195	ZC3H8
ZNF197	ZC3HAV1
ZNF20	ZC3HAV1L
ZNF200	ZC4H2
ZNF202	ZCCHC10
ZNF204P	ZCCHC12
ZNF205	ZCCHC14
ZNF205-AS1	ZCCHC17
ZNF207	ZCCHC18
ZNF211	ZCCHC2
ZNF212	ZCCHC24
ZNF213	ZCCHC3
ZNF214	ZCCHC4
ZNF215	ZCCHC6
ZNF217	ZCCHC7
ZNF219	ZCCHC8
ZNF22	ZCCHC9
ZNF221	ZCRB1
ZNF222	ZCWPW1
ZNF223	ZCWPW1L1
ZNF224	ZCWPW2
ZNF225	ZDBF2

ZNF226	ZDHHC1
ZNF227	ZDHHC11
ZNF229	ZDHHC11B
ZNF22-AS1	ZDHHC12
ZNF23	ZDHHC13
ZNF230	ZDHHC14
ZNF232	ZDHHC15
ZNF233	ZDHHC16
ZNF234	ZDHHC17
ZNF235	ZDHHC18
ZNF236	ZDHHC19
ZNF238.2.L	ZDHHC2
ZNF239	ZDHHC20
ZNF24	ZDHHC21
ZNF248	ZDHHC22
ZNF25	ZDHHC23
ZNF250	ZDHHC24
ZNF251	ZDHHC3
ZNF252P	ZDHHC4
ZNF253	ZDHHC5
ZNF254	ZDHHC6
ZNF256	ZDHHC7
ZNF26	ZDHHC8
ZNF260	ZDHHC8P1
ZNF263	ZDHHC9
ZNF264	ZEB1
ZNF266	ZEB1-AS1
ZNF267	ZEB2
ZNF268	ZEB2A
ZNF271P	ZEB2-AS1
ZNF273	ZEN
ZNF274	ZER1
ZNF275	ZFAND1
ZNF276	ZFAND2A
ZNF277	ZFAND2B
ZNF28	ZFAND3

ZNF280A	ZFAND4
ZNF280B	ZFAND5
ZNF280C	ZFAND5A
ZNF280D	ZFAND5B
ZNF281	ZFAND6
ZNF282	ZFAS1
ZNF283	ZFAT
ZNF284	ZFAT-AS1
ZNF286A	ZFC3H1
ZNF287	ZFHX2
ZNF292	ZFHX3
ZNF295-AS1	ZFHX4
ZNF296	ZFHX4-AS1
ZNF29P	ZFP1
ZNF3	ZFP101
ZNF30	ZFP105
ZNF300	ZFP106
ZNF300P1	ZFP11
ZNF302	ZFP110
ZNF304	ZFP112
ZNF311	ZFP119B
ZNF317	ZFP12
ZNF318	ZFP125
ZNF319	ZFP131
ZNF32	ZFP14
ZNF320	ZFP146
ZNF321P	ZFP148
ZNF322	ZFP161
ZNF322P1	ZFP174
ZNF324	ZFP180
ZNF324B	ZFP182
ZNF326	ZFP189
ZNF329	ZFP2
ZNF330	ZFP202
ZNF331	ZFP207
ZNF333	ZFP219

ZNF334	ZFP235
ZNF335	ZFP236
ZNF337	ZFP239
ZNF337-AS1	ZFP24
ZNF33A	ZFP26
ZNF33B	ZFP263
ZNF34	ZFP266
ZNF341	ZFP27
ZNF341-AS1	ZFP273
ZNF345	ZFP276
ZNF346	ZFP277
ZNF347	ZFP28
ZNF35	ZFP280D
ZNF350	ZFP282
ZNF354A	ZFP286
ZNF354B	ZFP287
ZNF354C	ZFP292
ZNF358	ZFP296
ZNF362	ZFP3
ZNF365	ZFP30
ZNF366	ZFP316
ZNF367	ZFP317
ZNF37A	ZFP319
ZNF37BP	ZFP326
ZNF382	ZFP329
ZNF383	ZFP330
ZNF384	ZFP33B
ZNF385A	ZFP345
ZNF385B	ZFP346
ZNF385C	ZFP35
ZNF385D	ZFP354A
ZNF391	ZFP354C
ZNF394	ZFP36
ZNF395	ZFP365
ZNF396	ZFP366
ZNF397	ZFP367

ZNF398	ZFP369
ZNF404	ZFP36L1
ZNF407	ZFP36L2
ZNF407-AS1	ZFP37
ZNF408	ZFP385A
ZNF41	ZFP385B
ZNF410	ZFP385C
ZNF414	ZFP386
ZNF415	ZFP389
ZNF416	ZFP40
ZNF418	ZFP407
ZNF419	ZFP41
ZNF420	ZFP418
ZNF423	ZFP42
ZNF425	ZFP420
ZNF426	ZFP422-PS
ZNF428	ZFP423
ZNF429	ZFP426
ZNF43	ZFP429
ZNF430	ZFP433
ZNF431	ZFP444
ZNF432	ZFP445
ZNF433	ZFP451
ZNF436	ZFP454
ZNF436-AS1	ZFP455
ZNF438	ZFP459
ZNF439	ZFP462
ZNF44	ZFP467
ZNF440	ZFP473
ZNF441	ZFP493
ZNF442	ZFP51
ZNF443	ZFP518A
ZNF444	ZFP52
ZNF445	ZFP521
ZNF446	ZFP532
ZNF449	ZFP536

ZNF45	ZFP54
ZNF451	ZFP558
ZNF454	ZFP563
ZNF460	ZFP566
ZNF461	ZFP568
ZNF462	ZFP57
ZNF467	ZFP59
ZNF468	ZFP592
ZNF469	ZFP593
ZNF470	ZFP598
ZNF473	ZFP606
ZNF474	ZFP607A
ZNF479	ZFP608
ZNF48	ZFP609
ZNF480	ZFP612
ZNF483	ZFP617
ZNF484	ZFP62
ZNF485	ZFP623
ZNF486	ZFP628
ZNF487	ZFP638
ZNF488	ZFP64
ZNF491	ZFP641
ZNF492	ZFP646
ZNF493	ZFP663
ZNF496	ZFP664
ZNF497	ZFP668
ZNF500	ZFP687
ZNF501	ZFP688
ZNF503	ZFP689
ZNF503-AS1	ZFP69
ZNF503-AS2	ZFP697
ZNF506	ZFP69B
ZNF507	ZFP7
ZNF510	ZFP704
ZNF511	ZFP709
ZNF512	ZFP709L1

ZNF512B	ZFP710
ZNF513	ZFP715
ZNF514	ZFP72
ZNF516	ZFP740
ZNF516-AS1	ZFP747
ZNF517	ZFP750
ZNF518A	ZFP758
ZNF518B	ZFP759
ZNF519	ZFP760
ZNF521	ZFP763
ZNF524	ZFP772
ZNF525	ZFP775
ZNF526	ZFP777
ZNF527	ZFP780B
ZNF528-AS1	ZFP781
ZNF529	ZFP786
ZNF530	ZFP787
ZNF532	ZFP804B
ZNF536	ZFP809
ZNF540	ZFP81
ZNF541	ZFP810
ZNF542P	ZFP819
ZNF543	ZFP82
ZNF544	ZFP820
ZNF546	ZFP830
ZNF547	ZFP831
ZNF548	ZFP839
ZNF549	ZFP84
ZNF550	ZFP866
ZNF551	ZFP868
ZNF552	ZFP871
ZNF554	ZFP873
ZNF555	ZFP874B
ZNF556	ZFP882
ZNF557	ZFP9
ZNF558	ZFP90

ZNF559	ZFP91
ZNF559-ZNF177	ZFP91-CNTF
ZNF560	ZFP92
ZNF561	ZFP933
ZNF561-AS1	ZFP934
ZNF562	ZFP937
ZNF563	ZFP940
ZNF564	ZFP945
ZNF565	ZFP948
ZNF566	ZFP950
ZNF567	ZFP951
ZNF567-DT	ZFP953
ZNF568	ZFP954
ZNF569	ZFP961
ZNF57	ZFP982
ZNF570	ZFPL1
ZNF571	ZFPM1
ZNF572	ZFPM2
ZNF573	ZFR
ZNF574	ZFR2
ZNF575	ZFTA
ZNF576	ZFTRAF1
ZNF577	ZFX
ZNF578	ZFY
ZNF579	ZFYVE1
ZNF580	ZFYVE16
ZNF581	ZFYVE19
ZNF582	ZFYVE21
ZNF583	ZFYVE26
ZNF584	ZFYVE28
ZNF585A	ZFYVE9
ZNF585B	ZG16
ZNF586	ZG16B
ZNF587	ZGLP1
ZNF587B	ZGPAT
ZNF589	ZGRF1

ZNF592	ZHX1
ZNF593	ZHX1-C8ORF76
ZNF594	ZHX2
ZNF594-DT	ZHX3
ZNF595	ZIC1
ZNF596	ZIC2
ZNF597	ZIC3
ZNF598	ZIC4
ZNF599	ZIC5
ZNF6	ZIC6
ZNF600	ZIK1
ZNF605	ZIM1
ZNF606	ZIM2
ZNF607	ZK637.18
ZNF608	ZKSCAN1
ZNF609	ZKSCAN14
ZNF610	ZKSCAN2
ZNF611	ZKSCAN3
ZNF613	ZKSCAN4
ZNF614	ZKSCAN5
ZNF615	ZKSCAN6
ZNF616	ZKSCAN7
ZNF618	ZKSCAN8
ZNF620	ZKSCAN8P1
ZNF621	ZMAT1
ZNF622	ZMAT2
ZNF623	ZMAT3
ZNF624	ZMAT4
ZNF625	ZMAT5
ZNF626	ZMIZ1
ZNF627	ZMIZ1-AS1
ZNF628	ZMIZ2
ZNF629	ZMPSTE24
ZNF630	ZMYM1
ZNF638	ZMYM2
ZNF639	ZMYM3

ZNF641	ZMYM4
ZNF643	ZMYM5
ZNF644	ZMYM6
ZNF646	ZMYND10
ZNF648	ZMYND11
ZNF649	ZMYND12
ZNF652	ZMYND15
ZNF653	ZMYND19
ZNF654	ZMYND8
ZNF655	ZNF10
ZNF658	ZNF100
ZNF66	ZNF101
ZNF660	ZNF106
ZNF662	ZNF107
ZNF664	ZNF112
ZNF665	ZNF114
ZNF667	ZNF117
ZNF667-AS1	ZNF12
ZNF668	ZNF121
ZNF669	ZNF124
ZNF670	ZNF131
ZNF671	ZNF132
ZNF672	ZNF133
ZNF674	ZNF134
ZNF674-AS1	ZNF135
ZNF675	ZNF136
ZNF677	ZNF137P
ZNF678	ZNF138
ZNF681	ZNF14
ZNF682	ZNF140
ZNF683	ZNF141
ZNF684	ZNF142
ZNF688	ZNF143
ZNF689	ZNF146
ZNF69	ZNF148
ZNF691	ZNF155

ZNF692	ZNF157
ZNF695	ZNF16
ZNF696	ZNF160
ZNF697	ZNF165
ZNF699	ZNF169
ZNF7	ZNF174
ZNF70	ZNF175
ZNF700	ZNF177
ZNF701	ZNF18
ZNF702P	ZNF180
ZNF703	ZNF181
ZNF704	ZNF182
ZNF706	ZNF184
ZNF707	ZNF185
ZNF708	ZNF189
ZNF709	ZNF19
ZNF71	ZNF195
ZNF710	ZNF197
ZNF710-AS1	ZNF20
ZNF711	ZNF200
ZNF713	ZNF202
ZNF714	ZNF204P
ZNF717	ZNF205
ZNF718	ZNF205-AS1
ZNF721	ZNF207
ZNF724	ZNF211
ZNF726	ZNF212
ZNF729	ZNF213
ZNF733P	ZNF214
ZNF737	ZNF215
ZNF738	ZNF217
ZNF74	ZNF219
ZNF740	ZNF22
ZNF746	ZNF221
ZNF747	ZNF222
ZNF750	ZNF223

ZNF75A	ZNF224
ZNF75D	ZNF225
ZNF76	ZNF226
ZNF761	ZNF227
ZNF763	ZNF229
ZNF764	ZNF22-AS1
ZNF765	ZNF23
ZNF766	ZNF230
ZNF767P	ZNF232
ZNF768	ZNF233
ZNF77	ZNF234
ZNF770	ZNF235
ZNF771	ZNF236
ZNF773	ZNF238.2.L
ZNF774	ZNF239
ZNF775	ZNF24
ZNF776	ZNF248
ZNF777	ZNF25
ZNF778	ZNF250
ZNF780A	ZNF251
ZNF780B	ZNF252P
ZNF781	ZNF253
ZNF782	ZNF254
ZNF784	ZNF256
ZNF785	ZNF26
ZNF786	ZNF260
ZNF787	ZNF263
ZNF788P	ZNF264
ZNF789	ZNF266
ZNF79	ZNF267
ZNF790	ZNF268
ZNF791	ZNF271P
ZNF792	ZNF273
ZNF793	ZNF274
ZNF799	ZNF275
ZNF800	ZNF276

ZNF804A	ZNF277
ZNF804B	ZNF28
ZNF805	ZNF280A
ZNF81	ZNF280B
ZNF813	ZNF280C
ZNF814	ZNF280D
ZNF815P	ZNF281
ZNF816	ZNF282
ZNF818P	ZNF283
ZNF821	ZNF284
ZNF823	ZNF286A
ZNF826P	ZNF287
ZNF827	ZNF292
ZNF829	ZNF295-AS1
ZNF83	ZNF296
ZNF830	ZNF29P
ZNF831	ZNF3
ZNF833P	ZNF30
ZNF835	ZNF300
ZNF836	ZNF300P1
ZNF837	ZNF302
ZNF839	ZNF304
ZNF84	ZNF311
ZNF840P	ZNF317
ZNF841	ZNF318
ZNF843	ZNF319
ZNF844	ZNF32
ZNF845	ZNF320
ZNF846	ZNF321P
ZNF84-DT	ZNF322
ZNF85	ZNF322P1
ZNF850	ZNF324
ZNF853	ZNF324B
ZNF862	ZNF326
ZNF865	ZNF329
ZNF875	ZNF330

ZNF878	ZNF331
ZNF879	ZNF333
ZNF880	ZNF334
ZNF883	ZNF335
ZNF890P	ZNF337
ZNF891	ZNF337-AS1
ZNF8-DT	ZNF33A
ZNF90	ZNF33B
ZNF91	ZNF34
ZNF92	ZNF341
ZNF93	ZNF341-AS1
ZNFX1	ZNF345
ZNG1A	ZNF346
ZNG1C	ZNF347
ZNG1E	ZNF35
ZNHIT1	ZNF350
ZNHIT2	ZNF354A
ZNHIT3	ZNF354B
ZNHIT6	ZNF354C
ZNRD2	ZNF358
ZNRF1	ZNF362
ZNRF2	ZNF365
ZNRF2P1	ZNF366
ZNRF3	ZNF367
ZNRF4	ZNF37A
ZP1	ZNF37BP
ZP2	ZNF382
ZP2.3	ZNF383
ZP3	ZNF384
ZP3.1	ZNF385A
ZP3R	ZNF385B
ZP4	ZNF385C
ZPBP	ZNF385D
ZPBP2	ZNF391
ZPLD1	ZNF394
ZPLD2P	ZNF395

ZPR1	ZNF396
ZRANB1	ZNF397
ZRANB2	ZNF398
ZRANB2-DT	ZNF404
ZRANB3	ZNF407
ZRSR1	ZNF407-AS1
ZRSR2	ZNF408
ZSCAN1	ZNF41
ZSCAN10	ZNF410
ZSCAN12	ZNF414
ZSCAN16	ZNF415
ZSCAN16-AS1	ZNF416
ZSCAN18	ZNF418
ZSCAN2	ZNF419
ZSCAN20	ZNF420
ZSCAN21	ZNF423
ZSCAN22	ZNF425
ZSCAN23	ZNF426
ZSCAN25	ZNF428
ZSCAN26	ZNF429
ZSCAN29	ZNF43
ZSCAN30	ZNF430
ZSCAN31	ZNF431
ZSCAN32	ZNF432
ZSCAN4	ZNF433
ZSCAN4C	ZNF436
ZSCAN5A	ZNF436-AS1
ZSCAN5B	ZNF438
ZSCAN9	ZNF439
ZSWIM2	ZNF44
ZSWIM3	ZNF440
ZSWIM4	ZNF441
ZSWIM5	ZNF442
ZSWIM6	ZNF443
ZSWIM7	ZNF444
ZSWIM8	ZNF445

ZSWIM9	ZNF446
ZUP1	ZNF449
ZW10	ZNF45
ZWILCH	ZNF451
ZWINT	ZNF454
ZXDA	ZNF460
ZXDB	ZNF461
ZXDC	ZNF462
ZYG11A	ZNF467
ZYG11B	ZNF468
ZYX	ZNF469
ZZEF1	ZNF470
ZZZ3	ZNF473
	ZNF474
	ZNF479
	ZNF48
	ZNF480
	ZNF483
	ZNF484
	ZNF485
	ZNF486
	ZNF487
	ZNF488
	ZNF491
	ZNF492
	ZNF493
	ZNF496
	ZNF497
	ZNF500
	ZNF501
	ZNF503
	ZNF503-AS1
	ZNF503-AS2
	ZNF506
	ZNF507
	ZNF510

ZNF511
ZNF512
ZNF512B
ZNF513
ZNF514
ZNF516
ZNF516-AS1
ZNF517
ZNF518A
ZNF518B
ZNF519
ZNF521
ZNF524
ZNF525
ZNF526
ZNF527
ZNF528-AS1
ZNF529
ZNF530
ZNF532
ZNF536
ZNF540
ZNF541
ZNF542P
ZNF543
ZNF544
ZNF546
ZNF547
ZNF548
ZNF549
ZNF550
ZNF551
ZNF552
ZNF554
ZNF555
ZNF556

ZNF557

ZNF558

ZNF559

ZNF559-ZNF177

ZNF560

ZNF561

ZNF561-AS1

ZNF562

ZNF563

ZNF564

ZNF565

ZNF566

ZNF567

ZNF567-DT

ZNF568

ZNF569

ZNF57

ZNF570

ZNF571

ZNF572

ZNF573

ZNF574

ZNF575

ZNF576

ZNF577

ZNF578

ZNF579

ZNF580

ZNF581

ZNF582

ZNF583

ZNF584

ZNF585A

ZNF585B

ZNF586

ZNF587

ZNF587B
ZNF589
ZNF592
ZNF593
ZNF594
ZNF594-DT
ZNF595
ZNF596
ZNF597
ZNF598
ZNF599
ZNF6
ZNF600
ZNF605
ZNF606
ZNF607
ZNF608
ZNF609
ZNF610
ZNF611
ZNF613
ZNF614
ZNF615
ZNF616
ZNF618
ZNF620
ZNF621
ZNF622
ZNF623
ZNF624
ZNF625
ZNF626
ZNF627
ZNF628
ZNF629
ZNF630

ZNF638

ZNF639

ZNF641

ZNF643

ZNF644

ZNF646

ZNF648

ZNF649

ZNF652

ZNF653

ZNF654

ZNF655

ZNF658

ZNF66

ZNF660

ZNF662

ZNF664

ZNF665

ZNF667

ZNF667-AS1

ZNF668

ZNF669

ZNF670

ZNF671

ZNF672

ZNF674

ZNF674-AS1

ZNF675

ZNF677

ZNF678

ZNF681

ZNF682

ZNF683

ZNF684

ZNF688

ZNF689

ZNF69
ZNF691
ZNF692
ZNF695
ZNF696
ZNF697
ZNF699
ZNF7
ZNF70
ZNF700
ZNF701
ZNF702P
ZNF703
ZNF704
ZNF706
ZNF707
ZNF708
ZNF709
ZNF71
ZNF710
ZNF710-AS1
ZNF711
ZNF713
ZNF714
ZNF717
ZNF718
ZNF721
ZNF724
ZNF726
ZNF729
ZNF733P
ZNF737
ZNF738
ZNF74
ZNF740
ZNF746

ZNF747

ZNF750

ZNF75A

ZNF75D

ZNF76

ZNF761

ZNF763

ZNF764

ZNF765

ZNF766

ZNF767P

ZNF768

ZNF77

ZNF770

ZNF771

ZNF773

ZNF774

ZNF775

ZNF776

ZNF777

ZNF778

ZNF780A

ZNF780B

ZNF781

ZNF782

ZNF784

ZNF785

ZNF786

ZNF787

ZNF788P

ZNF789

ZNF79

ZNF790

ZNF791

ZNF792

ZNF793

ZNF799

ZNF800

ZNF804A

ZNF804B

ZNF805

ZNF81

ZNF813

ZNF814

ZNF815P

ZNF816

ZNF818P

ZNF821

ZNF823

ZNF826P

ZNF827

ZNF829

ZNF83

ZNF830

ZNF831

ZNF833P

ZNF835

ZNF836

ZNF837

ZNF839

ZNF84

ZNF840P

ZNF841

ZNF843

ZNF844

ZNF845

ZNF846

ZNF84-DT

ZNF85

ZNF850

ZNF853

ZNF862

ZNF865

ZNF875

ZNF878

ZNF879

ZNF880

ZNF883

ZNF890P

ZNF891

ZNF8-DT

ZNF90

ZNF91

ZNF92

ZNF93

ZNFX1

ZNG1A

ZNG1C

ZNG1E

ZNHIT1

ZNHIT2

ZNHIT3

ZNHIT6

ZNRD1ASP

ZNRD2

ZNRF1

ZNRF2

ZNRF2P1

ZNRF3

ZNRF4

ZP1

ZP2

ZP2.3

ZP3

ZP3.1

ZP3R

ZP4

ZPBP

ZPBP2
ZPLD1
ZPLD2P
ZPR1
ZRANB1
ZRANB2
ZRANB2-DT
ZRANB3
ZRSR1
ZRSR2
ZSCAN1
ZSCAN10
ZSCAN12
ZSCAN16
ZSCAN16-AS1
ZSCAN18
ZSCAN2
ZSCAN20
ZSCAN21
ZSCAN22
ZSCAN23
ZSCAN25
ZSCAN26
ZSCAN29
ZSCAN30
ZSCAN31
ZSCAN32
ZSCAN4
ZSCAN4C
ZSCAN5A
ZSCAN5B
ZSCAN9
ZSWIM2
ZSWIM3
ZSWIM4
ZSWIM5

ZSWIM6
ZSWIM7
ZSWIM8
ZSWIM9
ZUP1
ZW10
ZWILCH
ZWINT
ZXDA
ZXDB
ZXDC
ZYG11A
ZYG11B
ZYX
ZZEF1
ZZZ3

Table S4. GO Terms and KEGG pathways associated with the 79 identified key targets

ID	Term	Ontology Source	Term PValue	Term PValue Corrected with Benjamini-Hochberg	% Associated Genes	Number of Genes	Associated Genes Found
KEGG:05200	Pathways in cancer	KEGG	5.99E-65	5.04E-62	9.981168	53	[AKT1, BCL2L1, CASP3, CASP8, CCNA2, CCND1, CDH1, CDK2, CDK4, CDK6, CHUK, EGFR, ERBB2, ESR1, ESR2, FN1, FOS, FOXO1, GSK3B, GSTP1, HMOX1, HSP90AA1, IGF1, IGF1R, IL6, JUN, KEAP1, KRAS, MAP2K1, MAPK1, MAPK3, MAPK8, MMP2, MMP9, MTOR, MYC, NFE2L2, NFKB1, NFKBIA, NOS2, NOTCH1, PPARG, PRKCA, PTGS2, PTK2, RB1, RELA, RXRA, SMAD2, STAT1, STAT3, TP53, VEGFA]
GO:1901701	cellular response to oxygen-containing compound	GO_BP	7.52E-49	3.17E-46	4.344453	56	[AHR, AKT1, APP, ATM, BCL2L1, CCL2, CCNA2, CCNB1, CDH1, CDK1, CDK2, CDK4, CDK5, CHUK, EGFR, ESR1, ESR2, EZH2, FOS, FOXO1, GSK3B, GSTP1, HMGB1, IGF1, IGF1R, IL1B, IL6, INS, IRS1, JUN, MAPK1, MAPK14, MAPK3, MAPK8, MMP2, MMP9, MTOR, MYC, NFE2L2, NFKB1, NFKBIA, NOS2, NOS3, PARP1, PDPK1, PPARG, PRKCA, PRKCD, PTGS2, PTK2, RELA, SRC, STAT1, STAT3, TLR4, TP53]
KEGG:05417	Lipid and atherosclerosis	KEGG	1.28E-46	3.60E-44	15.813953	34	[AKT1, BCL2L1, CASP3, CASP8, CCL2, CHUK, FOS, GSK3B, HSP90AA1, HSPA8, IL1B, IL6, JUN, KRAS, MAPK1, MAPK14, MAPK3, MAPK8, MMP9, NFATC1, NFE2L2, NFKB1, NFKBIA, NOS3, PDPK1, PPARG, PRKCA, PTK2, RELA, RXRA, SRC, STAT3, TLR4, TP53]
GO:0010243	response to organonitrogen compound	GO_BP	4.05E-44	8.52E-42	4.4894366	51	[AHR, AKT1, APP, BCL2L1, CASP3, CAT, CCNA2, CCND1, CDH1, CDK1, CDK4, CDK5, CHUK, EGFR, EZH2, FOS, FOXO1, GSK3B, GSTP1, IGF1, IGF1R, IL1B, IL6, INS, IRS1, JUN, MAPK1, MAPK14, MAPK3, MMP2, MMP9, MTOR, MYC, NFE2L2, NFKB1, NFKBIA, NOTCH1, PARP1, PDPK1, PPARA, PPARG, PRKCA, PRKCD, PTGS2,

							PTK2, RELA, SRC, STAT1, STAT3, TLR4, TP53]
GO:1901698	response to nitrogen compound	GO_BP	5.84E-44	9.84E-42	4.248366	52	[AHR, AKT1, APP, BCL2L1, CASP3, CAT, CCNA2, CCND1, CDH1, CDK1, CDK2, CDK4, CDK5, CHUK, EGFR, EZH2, FOS, FOXO1, GSK3B, GSTP1, IGF1, IGF1R, IL1B, IL6, INS, IRS1, JUN, MAPK1, MAPK14, MAPK3, MMP2, MMP9, MTOR, MYC, NFE2L2, NFKB1, NFKBIA, NOTCH1, PARP1, PDPK1, PPARA, PPARG, PRKCA, PRKCD, PTGS2, PTK2, RELA, SRC, STAT1, STAT3, TLR4, TP53]
KEGG:04151	PI3K-Akt signaling pathway	KEGG	3.01E-37	1.95E-35	9.322034	33	[AKT1, BCL2L1, CCND1, CDK2, CDK4, CDK6, CHUK, EGFR, ERBB2, FN1, GSK3B, HSP90AA1, IGF1, IGF1R, IL6, INS, IRS1, KRAS, MAP2K1, MAPK1, MAPK3, MTOR, MYC, NFKB1, NOS3, PDPK1, PRKCA, PTK2, RELA, RXRA, TLR4, TP53, VEGFA]
KEGG:05163	Human cytomegalovirus infection	KEGG	2.49E-40	2.62E-38	13.777778	31	[AKT1, CASP3, CASP8, CCL2, CCND1, CDK4, CDK6, CHUK, EGFR, GSK3B, IL1B, IL6, KRAS, MAP2K1, MAPK1, MAPK14, MAPK3, MTOR, MYC, NFATC1, NFKB1, NFKBIA, PRKCA, PTGS2, PTK2, RB1, RELA, SRC, STAT3, TP53, VEGFA]
KEGG:05167	Kaposi sarcoma-associated herpesvirus infection	KEGG	1.21E-40	1.46E-38	15.544042	30	[AKT1, CASP3, CASP8, CCND1, CDK4, CDK6, CHUK, FOS, GSK3B, IL6, JUN, KRAS, MAP2K1, MAPK1, MAPK14, MAPK3, MAPK8, MTOR, MYC, NFATC1, NFKB1, NFKBIA, PTGS2, RB1, RELA, SRC, STAT1, STAT3, TP53, VEGFA]
KEGG:05161	Hepatitis B	KEGG	3.15E-39	2.65E-37	17.28395	28	[AKT1, CASP3, CASP8, CCNA2, CDK2, CHUK, FOS, IL6, JUN, KRAS, MAP2K1, MAPK1, MAPK14, MAPK3, MAPK8, MMP9, MYC, NFATC1, NFKB1, NFKBIA, PRKCA, RB1, RELA, SRC, STAT1, STAT3, TLR4, TP53]
KEGG:05165	Human papillomavirus infection	KEGG	3.71E-30	1.01E-28	8.459214	28	[AKT1, ATM, CASP3, CASP8, CCNA2, CCND1, CDK2, CDK4, CDK6, CHUK, EGFR, FN1, FOXO1, GSK3B, KRAS, MAP2K1, MAPK1, MAPK3,

							MTOR, NFKB1, NOTCH1, PTGS2, PTK2, RB1, RELA, STAT1, TP53, VEGFA]
GO:0014070	response to organic cyclic compound	GO_BP	3.29E-38	2.52E-36	4.3233085	46	[AHR, AKT1, APP, BCL2L1, CASP3, CASP8, CAT, CCL2, CCNA2, CCNB1, CCND1, CDH1, CDK1, CDK4, CDK5, EGFR, ESR1, ESR2, EZH2, FOS, FOXO1, GSK3B, GSTP1, HMGB1, IL1B, IL6, JUN, KRAS, MAPK1, MAPK3, MTOR, MYC, NFKB1, NFKBIA, NOTCH1, PARP1, PPARA, PPARG, PRKCA, PTGS2, RELA, RXRA, SMAD2, SRC, STAT1, STAT3]
GO:1901652	response to peptide	GO_BP	1.54E-37	1.08E-35	6.5857887	38	[AKT1, APP, CAT, CCNA2, CDK4, CDK5, CHUK, FOXO1, GSK3B, GSTP1, IGF1, IGF1R, IL1B, INS, IRS1, MAPK14, MMP2, MMP9, MTOR, MYC, NFE2L2, NFKB1, NFKBIA, NOTCH1, PARP1, PDPK1, PPARA, PPARG, PRKCA, PRKCD, PTGS2, PTK2, RELA, SRC, STAT1, STAT3, TLR4, TP53]
KEGG:05205	Proteoglycans in cancer	KEGG	2.13E-34	9.96E-33	13.170732	27	[AKT1, CASP3, CCND1, EGFR, ERBB2, ESR1, FN1, IGF1, IGF1R, KRAS, MAP2K1, MAPK1, MAPK14, MAPK3, MMP2, MMP9, MTOR, MYC, PDPK1, PRKCA, PTK2, SMAD2, SRC, STAT3, TLR4, TP53, VEGFA]
GO:0062197	cellular response to chemical stress	GO_BP	1.02E-36	6.12E-35	8.991826	33	[AKT1, ATM, CASP3, CAT, CDK1, CHUK, EGFR, EZH2, FOS, FOXO1, GSTP1, HMOX1, IL6, INS, JUN, KEAP1, MAPK1, MAPK3, MAPK8, MMP2, MMP9, MYC, NFE2L2, NOS3, PARP1, PDPK1, PPARG, PRKCD, PTGS2, RELA, SRC, TLR4, TP53]
GO:0033993	response to lipid	GO_BP	1.62E-36	9.10E-35	4.38247	44	[AHR, AKT1, ATM, CASP3, CASP8, CAT, CCL2, CCNA2, CCNB1, CCND1, CDK4, CHUK, EGFR, ESR1, ESR2, EZH2, FOS, FOXO1, GSTP1, HMGB1, IL1B, IL6, IRS1, JUN, KRAS, MAPK1, MAPK14, MAPK3, MYC, NFKBIA, NOS2, NOS3, NOTCH1, PARP1, PPARA, PPARG, PRKCA, PTGS2, RELA, RXRA, SMAD2, SRC, STAT3, TLR4]
KEGG:04933	AGE-RAGE signaling pathway in diabetic complications	KEGG	1.82E-41	2.55E-39	26	26	[AKT1, CASP3, CCL2, CCND1, CDK4, FN1, FOXO1, IL1B, IL6, JUN, KRAS, MAPK1, MAPK14, MAPK3, MAPK8, MMP2, NFATC1, NFKB1, NOS3, PRKCA, PRKCD, RELA, SMAD2, STAT1, STAT3,

							VEGFA]
GO:0009725	response to hormone	GO_BP	7.31E-35	3.62E-33	4.2198234	43	[AKT1, CASP3, CAT, CCNA2, CCND1, CDK4, CHUK, EGFR, ESR1, ESR2, FOS, FOXO1, GSK3B, GSTP1, HMGB1, HMOX1, IGF1R, IL1B, IL6, INS, IRS1, KRAS, MAPK1, MTOR, MYC, NFE2L2, NFKB1, NOS2, NOS3, NOTCH1, PARP1, PDPK1, PPARA, PPARG, PRKCA, PRKCD, PTGS2, PTK2, RELA, RXRA, SRC, STAT1, STAT3]
KEGG:05215	Prostate cancer	KEGG	9.76E-40	9.13E-38	25.773195	25	[AKT1, CCND1, CDK2, CHUK, EGFR, ERBB2, FOXO1, GSK3B, GSTP1, HSP90AA1, IGF1, IGF1R, INS, KRAS, MAP2K1, MAPK1, MAPK3, MMP9, MTOR, NFKB1, NFKBIA, PDPK1, RB1, RELA, TP53]
GO:1901699	cellular response to nitrogen compound	GO_BP	2.56E-34	1.13E-32	5.098039	39	[AHR, AKT1, APP, BCL2L1, CASP3, CCNA2, CDH1, CDK2, CDK4, CDK5, EZH2, FOXO1, GSK3B, GSTP1, IGF1, IGF1R, IL1B, INS, IRS1, MAPK1, MAPK3, MMP2, MTOR, MYC, NFE2L2, NFKB1, PARP1, PDPK1, PPARG, PRKCA, PRKCD, PTGS2, PTK2, RELA, SRC, STAT1, STAT3, TLR4, TP53]
GO:0071417	cellular response to organonitrogen compound	GO_BP	2.71E-34	1.14E-32	5.4054055	38	[AHR, AKT1, APP, BCL2L1, CASP3, CCNA2, CDH1, CDK4, CDK5, EZH2, FOXO1, GSK3B, GSTP1, IGF1, IGF1R, IL1B, INS, IRS1, MAPK1, MAPK3, MMP2, MTOR, MYC, NFE2L2, NFKB1, PARP1, PDPK1, PPARG, PRKCA, PRKCD, PTGS2, PTK2, RELA, SRC, STAT1, STAT3, TLR4, TP53]
GO:0060548	negative regulation of cell death	GO_BP	4.48E-34	1.80E-32	4.041353	43	[AKT1, AURKA, BCL2L1, CASP8, CAT, CCL2, CCNA2, CDK1, CDK5, EGFR, FOXO1, GSK3B, GSTP1, HMOX1, IGF1, IGF1R, IL1B, IL6, INS, KRAS, MAPK1, MAPK3, MAPK8, MMP9, MTOR, MYC, NFE2L2, NFKB1, NFKBIA, NOS3, NOTCH1, PDPK1, PPARA, PRKCA, PRKCD, PTGS2, PTK2, RB1, RELA, SRC, STAT3, TP53, VEGFA]

GO:0043066	negative regulation of apoptotic process	GO_BP	1.60E-33	6.14E-32	4.347826	41	[AKT1, AURKA, BCL2L1, CASP8, CAT, CCL2, CCNA2, CDK1, EGFR, FOXO1, GSK3B, GSTP1, HMOX1, IGF1, IGF1R, IL1B, IL6, INS, KRAS, MAPK1, MAPK3, MAPK8, MMP9, MTOR, MYC, NFE2L2, NFKB1, NFKBIA, NOS3, NOTCH1, PDPK1, PPARA, PRKCA, PRKCD, PTGS2, PTK2, RB1, RELA, SRC, TP53, VEGFA]
GO:0043069	negative regulation of programmed cell death	GO_BP	3.87E-33	1.42E-31	4.253112	41	[AKT1, AURKA, BCL2L1, CASP8, CAT, CCL2, CCNA2, CDK1, EGFR, FOXO1, GSK3B, GSTP1, HMOX1, IGF1, IGF1R, IL1B, IL6, INS, KRAS, MAPK1, MAPK3, MAPK8, MMP9, MTOR, MYC, NFE2L2, NFKB1, NFKBIA, NOS3, NOTCH1, PDPK1, PPARA, PRKCA, PRKCD, PTGS2, PTK2, RB1, RELA, SRC, TP53, VEGFA]
GO:0008284	positive regulation of cell population proliferation	GO_BP	7.48E-33	2.62E-31	4.1836734	41	[AKT1, BCL2L1, CCNA2, CCNB1, CCND1, CDK1, CDK2, CDK4, CDK6, EGFR, ERBB2, ESR1, EZH2, FN1, HMGB1, HMOX1, IGF1, IGF1R, IL1B, IL6, INS, IRS1, JUN, KRAS, MAPK1, MAPK14, MAPK3, MMP2, MMP9, MTOR, MYC, NOTCH1, PDPK1, PRKCA, PTGS2, PTK2, RELA, STAT1, STAT3, TLR4, VEGFA]
KEGG:04010	MAPK signaling pathway	KEGG	6.63E-27	1.12E-25	8.503402	25	[AKT1, CASP3, CHUK, EGFR, ERBB2, FOS, HSPA8, IGF1, IGF1R, IL1B, INS, JUN, KRAS, MAP2K1, MAPK1, MAPK14, MAPK3, MAPK8, MYC, NFATC1, NFKB1, PRKCA, RELA, TP53, VEGFA]
KEGG:04218	Cellular senescence	KEGG	3.20E-32	1.08E-30	15.384615	24	[AKT1, ATM, CCNA2, CCNB1, CCND1, CDK1, CDK2, CDK4, CDK6, FOXO1, IL6, KRAS, MAP2K1, MAPK1, MAPK14, MAPK3, MTOR, MYC, NFATC1, NFKB1, RB1, RELA, SMAD2, TP53]
KEGG:05160	Hepatitis C	KEGG	3.77E-32	1.22E-30	15.286624	24	[AKT1, CASP3, CASP8, CCND1, CDK2, CDK4, CDK6, CHUK, EGFR, GSK3B, KRAS, MAP2K1, MAPK1, MAPK3, MYC, NFKB1, NFKBIA, PPARA, RB1, RELA, RXRA, STAT1, STAT3, TP53]
GO:0006979	response to oxidative stress	GO_BP	1.98E-31	5.95E-30	6.7085953	32	[AKT1, APP, CASP3, CAT, CDK1, CHUK, EGFR, EZH2, FOS, FOXO1, GSTP1, HMOX1, IL6, INS, JUN, KEAP1, MAPK1, MAPK3, MAPK8, MMP2,

							MMP9, NFE2L2, NOS3, PARP1, PDPK1, PRKCD, PTGS2, RELA, SRC, STAT1, TLR4, TP53]
GO:0034599	cellular response to oxidative stress	GO_BP	7.57E-31	2.20E-29	8.945687	28	[AKT1, CAT, CDK1, CHUK, EGFR, EZH2, FOS, FOXO1, GSTP1, HMOX1, IL6, INS, JUN, KEAP1, MAPK1, MAPK3, MAPK8, MMP2, MMP9, NFE2L2, NOS3, PARP1, PDPK1, PRKCD, RELA, SRC, TLR4, TP53]
KEGG:05170	Human immunodeficiency virus 1 infection	KEGG	7.25E-29	1.70E-27	11.320755	24	[AKT1, ATM, BCL2L1, CASP3, CASP8, CCNB1, CDK1, CHUK, FOS, JUN, KRAS, MAP2K1, MAPK1, MAPK14, MAPK3, MAPK8, MTOR, NFATC1, NFKB1, NFKBIA, PRKCA, PTK2, RELA, TLR4]
KEGG:05166	Human T-cell leukemia virus 1 infection	KEGG	1.62E-28	3.42E-27	10.958904	24	[AKT1, ATM, BCL2L1, CCNA2, CCND1, CDK2, CDK4, CHUK, FOS, IL6, JUN, KRAS, MAP2K1, MAPK1, MAPK3, MAPK8, MYC, NFATC1, NFKB1, NFKBIA, RB1, RELA, SMAD2, TP53]
KEGG:05162	Measles	KEGG	1.21E-31	3.77E-30	16.546762	23	[AKT1, BCL2L1, CASP3, CASP8, CCND1, CDK2, CDK4, CDK6, CHUK, FOS, GSK3B, HSPA8, IL1B, IL6, JUN, MAPK8, NFKB1, NFKBIA, RELA, STAT1, STAT3, TLR4, TP53]
KEGG:05206	MicroRNAs in cancer	KEGG	2.32E-23	2.72E-22	7.419355	23	[ATM, CASP3, CCND1, CDK6, EGFR, ERBB2, EZH2, HMOX1, IRS1, KRAS, MAP2K1, MAPK1, MAPK3, MMP9, MTOR, MYC, NFKB1, NOTCH1, PRKCA, PTGS2, STAT3, TP53, VEGFA]
GO:0010035	response to inorganic substance	GO_BP	5.24E-29	1.30E-27	5.254777	33	[AKT1, APP, CASP3, CASP8, CAT, CCNA2, CCNB1, CCND1, CDH1, CDK1, CDK2, CDK4, CHUK, EGFR, EZH2, FOS, FOXO1, HMOX1, IL6, JUN, MAPK1, MAPK3, MAPK8, MMP9, MYC, NFE2L2, NOS3, PARP1, PRKCD, PTGS2, RELA, SRC, STAT1]
KEGG:05212	Pancreatic cancer	KEGG	3.31E-36	1.74E-34	28.947369	22	[AKT1, BCL2L1, CCND1, CDK4, CDK6, CHUK, EGFR, ERBB2, KRAS, MAP2K1, MAPK1, MAPK3, MAPK8, MTOR, NFKB1, RB1, RELA, SMAD2, STAT1, STAT3, TP53, VEGFA]
KEGG:04068	FoxO signaling pathway	KEGG	2.05E-	5.74E-29	16.793894	22	[AKT1, ATM, CAT, CCNB1, CCND1, CDK2, CHUK, EGFR, FOXO1, IGF1, IGF1R, IL6, INS,

			30				IRS1, KRAS, MAP2K1, MAPK1, MAPK14, MAPK3, MAPK8, PDPK1, STAT3]
KEGG:05418	Fluid shear stress and atherosclerosis	KEGG	8.26E-30	2.17E-28	15.827338	22	[AKT1, CCL2, CHUK, FOS, GSTP1, HMOX1, HSP90AA1, IL1B, JUN, KEAP1, MAPK14, MAPK8, MMP2, MMP9, NFE2L2, NFKB1, NOS3, PTK2, RELA, SRC, TP53, VEGFA]
KEGG:05224	Breast cancer	KEGG	3.06E-29	7.81E-28	14.965986	22	[AKT1, CCND1, CDK4, CDK6, EGFR, ERBB2, ESR1, ESR2, FOS, GSK3B, IGF1, IGF1R, JUN, KRAS, MAP2K1, MAPK1, MAPK3, MTOR, MYC, NOTCH1, RB1, TP53]
KEGG:05225	Hepatocellular carcinoma	KEGG	6.77E-28	1.27E-26	13.095238	22	[AKT1, BCL2L1, CCND1, CDK4, CDK6, EGFR, GSK3B, GSTP1, HMOX1, IGF1R, KEAP1, KRAS, MAP2K1, MAPK1, MAPK3, MTOR, MYC, NFE2L2, PRKCA, RB1, SMAD2, TP53]
KEGG:05131	Shigellosis	KEGG	3.85E-24	4.99E-23	8.9430895	22	[AKT1, ATM, BCL2L1, CHUK, EGFR, FOXO1, GSK3B, IL1B, JUN, MAPK1, MAPK14, MAPK3, MAPK8, MTOR, NFKB1, NFKBIA, PRKCD, PTK2, RELA, SRC, TLR4, TP53]
KEGG:04926	Relaxin signaling pathway	KEGG	9.91E-29	2.26E-27	16.27907	21	[AKT1, EGFR, FOS, JUN, KRAS, MAP2K1, MAPK1, MAPK14, MAPK3, MAPK8, MMP2, MMP9, NFKB1, NFKBIA, NOS2, NOS3, PRKCA, RELA, SMAD2, SRC, VEGFA]
KEGG:05135	Yersinia infection	KEGG	3.81E-28	7.64E-27	15.328467	21	[AKT1, CCL2, CHUK, FN1, FOS, GSK3B, IL1B, IL6, JUN, MAP2K1, MAPK1, MAPK14, MAPK3, MAPK8, NFATC1, NFKB1, NFKBIA, PTK2, RELA, SRC, TLR4]
KEGG:05169	Epstein-Barr virus infection	KEGG	1.90E-24	2.53E-23	10.39604	21	[AKT1, CASP3, CASP8, CCNA2, CCND1, CDK2, CDK4, CDK6, CHUK, IL6, JUN, MAPK14, MAPK8, MYC, NFKB1, NFKBIA, RB1, RELA, STAT1, STAT3, TP53]
KEGG:05010	Alzheimer disease	KEGG	6.10E-19	4.32E-18	5.6910567	21	[AKT1, APP, CASP3, CASP8, CDK5, CHUK, GSK3B, IL1B, IL6, INS, IRS1, KRAS, MAP2K1, MAPK1, MAPK3, MAPK8, MTOR, NFKB1, NOS2, PTGS2, RELA]

KEGG:05022	Pathways of neurodegeneration	KEGG	1.04E-16	5.74E-16	4.4210525	21	[APP, BCL2L1, CASP3, CASP8, CAT, CDK5, GSK3B, IL1B, IL6, KRAS, MAP2K1, MAPK1, MAPK14, MAPK3, MAPK8, MTOR, NFKB1, NOS2, PRKCA, PTGS2, RELA]
GO:0000302	response to reactive oxygen species	GO_BP	1.14E-27	2.09E-26	10.126582	24	[AKT1, CASP3, CAT, CDK1, CHUK, EGFR, EZH2, FOS, FOXO1, GSTP1, HMOX1, IL6, JUN, MAPK1, MAPK3, MAPK8, MMP2, MMP9, NFE2L2, NOS3, PRKCD, RELA, SRC, STAT1]
GO:0019221	cytokine-mediated signaling pathway	GO_BP	2.93E-27	5.24E-26	4.107981	35	[AKT1, BCL2L1, CASP3, CASP8, CCL2, CCND1, CHUK, FN1, FOS, FOXO1, GSTP1, HMOX1, HSP90AA1, HSPA8, IL1B, IL6, IRS1, KRAS, MAPK3, MMP2, MMP9, MYC, NFKB1, NFKBIA, NOS2, PPARG, PRKCA, PRKCD, PTGS2, RELA, SRC, STAT1, STAT3, TP53, VEGFA]
GO:1901653	cellular response to peptide	GO_BP	4.19E-27	7.36E-26	6.5882354	28	[AKT1, APP, CCNA2, CDK4, CDK5, FOXO1, GSK3B, GSTP1, IGF1, IGF1R, IL1B, INS, IRS1, MYC, NFE2L2, NFKB1, PARP1, PDPK1, PPARG, PRKCA, PRKCD, PTK2, RELA, SRC, STAT1, STAT3, TLR4, TP53]
KEGG:04659	Th17 cell differentiation	KEGG	1.15E-28	2.56E-27	18.69159	20	[AHR, CHUK, FOS, HSP90AA1, IL1B, IL6, JUN, MAPK1, MAPK14, MAPK3, MAPK8, MTOR, NFATC1, NFKB1, NFKBIA, RELA, RXRA, SMAD2, STAT1, STAT3]
KEGG:04066	HIF-1 signaling pathway	KEGG	1.73E-28	3.54E-27	18.348623	20	[AKT1, EGFR, ERBB2, HMOX1, IGF1, IGF1R, IL6, INS, MAP2K1, MAPK1, MAPK3, MTOR, NFKB1, NOS2, NOS3, PRKCA, RELA, STAT3, TLR4, VEGFA]
KEGG:05222	Small cell lung cancer	KEGG	3.81E-28	7.47E-27	20.652174	19	[AKT1, BCL2L1, CASP3, CCND1, CDK2, CDK4, CDK6, CHUK, FN1, MYC, NFKB1, NFKBIA, NOS2, PTGS2, PTK2, RB1, RELA, RXRA, TP53]
KEGG:04657	IL-17 signaling pathway	KEGG	5.97E-28	1.14E-26	20.212767	19	[CASP3, CASP8, CCL2, CHUK, FOS, GSK3B, HSP90AA1, IL1B, IL6, JUN, MAPK1, MAPK14, MAPK3, MAPK8, MMP9, NFKB1, NFKBIA, PTGS2, RELA]
GO:0043434	response to peptide hormone	GO_BP	1.93E-	3.07E-24	5.7377048	28	[AKT1, CAT, CCNA2, CDK4, CHUK, FOXO1, GSK3B, GSTP1, IGF1R, IL1B, INS, IRS1, MTOR,

			25				MYC, NFE2L2, NFKB1, PARP1, PDPK1, PPARA, PPARG, PRKCA, PRKCD, PTGS2, PTK2, RELA, SRC, STAT1, STAT3]
GO:0030335	positive regulation of cell migration	GO_BP	2.19E-25	3.42E-24	5.2919707	29	[AKT1, APP, ATM, CCNA2, EGFR, FN1, HMGB1, HMOX1, IGF1, IGF1R, IL1B, IL6, INS, MAPK1, MAPK14, MAPK3, MMP9, MTOR, MYC, NFE2L2, NOS3, NOTCH1, PDPK1, PRKCA, PTGS2, PTK2, SRC, STAT3, VEGFA]
KEGG:04625	C-type lectin receptor signaling pathway	KEGG	4.84E-27	8.32E-26	18.26923	19	[AKT1, CASP8, CHUK, IL1B, IL6, JUN, KRAS, MAPK1, MAPK14, MAPK3, MAPK8, NFATC1, NFKB1, NFKBIA, PRKCD, PTGS2, RELA, SRC, STAT1]
GO:0051090	regulation of DNA-binding transcription factor activity	GO_BP	4.28E-25	6.43E-24	6.0402684	27	[AKT1, APP, CAT, CHUK, ESR1, ESR2, EZH2, FOS, HMOX1, IL1B, IL6, INS, JUN, KEAP1, KRAS, MAPK1, MAPK14, MAPK3, MAPK8, NFKB1, NFKBIA, PPARG, RB1, RELA, STAT3, TLR4, VEGFA]
GO:2000147	positive regulation of cell motility	GO_BP	7.40E-25	1.09E-23	5.06993	29	[AKT1, APP, ATM, CCNA2, EGFR, FN1, HMGB1, HMOX1, IGF1, IGF1R, IL1B, IL6, INS, MAPK1, MAPK14, MAPK3, MMP9, MTOR, MYC, NFE2L2, NOS3, NOTCH1, PDPK1, PRKCA, PTGS2, PTK2, SRC, STAT3, VEGFA]
KEGG:04668	TNF signaling pathway	KEGG	2.21E-26	3.58E-25	16.964285	19	[AKT1, CASP3, CASP8, CCL2, CHUK, FOS, IL1B, IL6, JUN, MAP2K1, MAPK1, MAPK14, MAPK3, MAPK8, MMP9, NFKB1, NFKBIA, PTGS2, RELA]
GO:0018209	peptidyl-serine modification	GO_BP	1.23E-24	1.76E-23	6.9060774	25	[AKT1, APP, ATM, AURKA, CCNB1, CDK1, CDK2, CDK5, CHUK, EGFR, GSK3B, HSP90AA1, IL6, MAPK1, MAPK14, MAPK3, MAPK8, MTOR, PARP1, PDPK1, PRKCA, PRKCD, PTGS2, SRC, VEGFA]
KEGG:04210	Apoptosis	KEGG	1.11E-24	1.62E-23	13.970589	19	[AKT1, ATM, BCL2L1, CASP3, CASP8, CHUK, FOS, JUN, KRAS, MAP2K1, MAPK1, MAPK3, MAPK8, NFKB1, NFKBIA, PARP1, PDPK1, RELA, TP53]
GO:0051272	positive regulation of cellular component	GO_BP	1.54E-	2.12E-23	4.940375	29	[AKT1, APP, ATM, CCNA2, EGFR, FN1, HMGB1, HMOX1, IGF1, IGF1R, IL1B, IL6, INS, MAPK1,

	movement		24				MAPK14, MAPK3, MMP9, MTOR, MYC, NFE2L2, NOS3, NOTCH1, PDPK1, PRKCA, PTGS2, PTK2, SRC, STAT3, VEGFA]
GO:0040017	positive regulation of locomotion	GO_BP	1.61E-24	2.19E-23	4.931973	29	[AKT1, APP, ATM, CCNA2, EGFR, FN1, HMGB1, HMOX1, IGF1, IGF1R, IL1B, IL6, INS, MAPK1, MAPK14, MAPK3, MMP9, MTOR, MYC, NFE2L2, NOS3, NOTCH1, PDPK1, PRKCA, PTGS2, PTK2, SRC, STAT3, VEGFA]
KEGG:05203	Viral carcinogenesis	KEGG	3.20E-21	2.83E-20	9.313725	19	[CASP3, CASP8, CCNA2, CCND1, CDK1, CDK2, CDK4, CDK6, JUN, KRAS, MAPK1, MAPK3, NFKB1, NFKBIA, RB1, RELA, SRC, STAT3, TP53]
KEGG:05132	Salmonella infection	KEGG	1.46E-19	1.08E-18	7.6305223	19	[AKT1, CASP3, CASP8, CHUK, FOS, HSP90AA1, IL1B, IL6, JUN, MAP2K1, MAPK1, MAPK14, MAPK3, MAPK8, MYC, NFKB1, NFKBIA, RELA, TLR4]
KEGG:04917	Prolactin signaling pathway	KEGG	1.34E-28	2.90E-27	25.714285	18	[AKT1, CCND1, ESR1, ESR2, FOS, GSK3B, INS, KRAS, MAP2K1, MAPK1, MAPK14, MAPK3, MAPK8, NFKB1, RELA, SRC, STAT1, STAT3]
GO:0018105	peptidyl-serine phosphorylation	GO_BP	5.10E-24	6.51E-23	7.1641793	24	[AKT1, APP, ATM, AURKA, CCNB1, CDK1, CDK2, CDK5, CHUK, EGFR, GSK3B, HSP90AA1, IL6, MAPK1, MAPK14, MAPK3, MAPK8, MTOR, PDPK1, PRKCA, PRKCD, PTGS2, SRC, VEGFA]
KEGG:05235	PD-L1 expression and PD-1 checkpoint pathway in cancer	KEGG	1.66E-26	2.74E-25	20.22472	18	[AKT1, CHUK, EGFR, FOS, JUN, KRAS, MAP2K1, MAPK1, MAPK14, MAPK3, MTOR, NFATC1, NFKB1, NFKBIA, RELA, STAT1, STAT3, TLR4]
KEGG:05142	Chagas disease	KEGG	2.39E-25	3.66E-24	17.647058	18	[AKT1, CASP8, CCL2, CHUK, FOS, IL1B, IL6, JUN, MAPK1, MAPK14, MAPK3, MAPK8, NFKB1, NFKBIA, NOS2, RELA, SMAD2, TLR4]
GO:0071396	cellular response to lipid	GO_BP	1.08E-23	1.32E-22	4.6104927	29	[AHR, AKT1, ATM, CCL2, CCNA2, CCNB1, CDK4, EGFR, ESR1, ESR2, FOXO1, GSTP1, HMGB1, IL1B, IRS1, MAPK1, MAPK14, MAPK3, MYC, NFKBIA, NOS2, PARP1, PPARA, PPARG, PRKCA, RELA, RXRA, SRC, TLR4]
GO:0045860	positive regulation of protein kinase activity	GO_BP	2.00E-23	2.40E-22	4.8442907	28	[AKT1, CCNA2, CCNB1, CCND1, CDK5, EGFR, ERBB2, EZH2, HMGB1, HSP90AA1, IGF1, IGF1R,

							IL1B, IL6, INS, KRAS, MAP2K1, MAPK1, MAPK14, MAPK3, MTOR, PDPK1, PRKCA, PRKCD, PTK2, SRC, TLR4, VEGFA]
KEGG:05145	Toxoplasmosis	KEGG	1.45E-24	2.04E-23	16.071428	18	[AKT1, BCL2L1, CASP3, CASP8, CHUK, HSPA8, MAPK1, MAPK14, MAPK3, MAPK8, NFKB1, NFKBIA, NOS2, PDPK1, RELA, STAT1, STAT3, TLR4]
KEGG:04919	Thyroid hormone signaling pathway	KEGG	6.38E-24	7.89E-23	14.876033	18	[AKT1, CCND1, ESR1, FOXO1, GSK3B, KRAS, MAP2K1, MAPK1, MAPK3, MTOR, MYC, NOTCH1, PDPK1, PRKCA, RXRA, SRC, STAT1, TP53]
KEGG:05164	Influenza A	KEGG	4.65E-21	3.95E-20	10.4651165	18	[AKT1, CASP3, CASP8, CCL2, CDK4, CDK6, CHUK, IL1B, IL6, MAP2K1, MAPK1, MAPK3, NFKB1, NFKBIA, PRKCA, RELA, STAT1, TLR4]
GO:0032870	cellular response to hormone stimulus	GO_BP	2.59E-23	2.99E-22	4.178273	30	[AKT1, CCNA2, CDK4, EGFR, ESR1, ESR2, FOS, FOXO1, GSK3B, GSTP1, IGF1R, IL1B, INS, IRS1, MYC, NFE2L2, NFKB1, NOTCH1, PARP1, PDPK1, PPARA, PPARG, PRKCA, PRKCD, PTK2, RELA, RXRA, SRC, STAT1, STAT3]
GO:0071407	cellular response to organic cyclic compound	GO_BP	2.71E-23	3.08E-22	4.4615383	29	[AHR, AKT1, APP, CASP3, CASP8, CCL2, CCNA2, CCNB1, CDH1, CDK4, EGFR, ESR1, ESR2, EZH2, FOXO1, GSK3B, GSTP1, IL1B, MAPK1, MAPK3, MYC, NFKB1, PARP1, PPARA, PPARG, PRKCA, PTGS2, RXRA, SRC]
GO:0033674	positive regulation of kinase activity	GO_BP	3.22E-23	3.61E-22	4.434251	29	[AKT1, CCNA2, CCNB1, CCND1, CDK5, EGFR, ERBB2, EZH2, HMGB1, HSP90AA1, IGF1, IGF1R, IL1B, IL6, INS, IRS1, KRAS, MAP2K1, MAPK1, MAPK14, MAPK3, MTOR, PDPK1, PRKCA, PRKCD, PTK2, SRC, TLR4, VEGFA]
GO:0080135	regulation of cellular response to stress	GO_BP	3.43E-23	3.80E-22	4.137931	30	[AKT1, APP, ATM, BCL2L1, CDK6, EGFR, FOXO1, GSK3B, GSTP1, H2AX, HMGB1, HSP90AA1, HSPA8, IGF1R, IL1B, IL6, INS, KRAS, MAP2K1, MAPK1, MAPK3, MTOR, MYC, NFE2L2, PARP1, PRKCD, PTGS2, TLR4, TP53, VEGFA]
GO:0042063	gliogenesis	GO_BP	3.88E-	4.25E-22	7.2555203	23	[AKT1, APP, CCL2, CDK1, CDK5, CDK6, EGFR, ERBB2, EZH2, GSTP1, IL1B, IL6, KRAS,

			23				MAP2K1, MAPK1, MAPK3, MTOR, MYC, NOTCH1, PPARG, RELA, STAT3, TLR4]
GO:0034614	cellular response to reactive oxygen species	GO_BP	4.04E-23	4.36E-22	11.656442	19	[AKT1, CDK1, CHUK, EGFR, EZH2, FOS, FOXO1, IL6, JUN, MAPK1, MAPK3, MAPK8, MMP2, MMP9, NFE2L2, NOS3, PRKCD, RELA, SRC]
KEGG:04621	NOD-like receptor signaling pathway	KEGG	1.19E-20	1.00E-19	9.944752	18	[BCL2L1, CASP8, CCL2, CHUK, HSP90AA1, IL1B, IL6, JUN, MAPK1, MAPK14, MAPK3, MAPK8, NFKB1, NFKBIA, PRKCD, RELA, STAT1, TLR4]
GO:0051347	positive regulation of transferase activity	GO_BP	5.50E-23	5.79E-22	4.070556	30	[AKT1, CCNA2, CCNB1, CCND1, CDK5, EGFR, ERBB2, EZH2, HMGB1, HSP90AA1, IGF1, IGF1R, IL1B, IL6, INS, IRS1, KRAS, MAP2K1, MAPK1, MAPK14, MAPK3, MTOR, MYC, PDPK1, PRKCA, PRKCD, PTK2, SRC, TLR4, VEGFA]
GO:0033002	muscle cell proliferation	GO_BP	8.97E-23	9.32E-22	9.803922	20	[AKT1, CCNB1, CDK1, EGFR, GSTP1, HMOX1, IGF1, IL6, JUN, MAPK1, MAPK14, MMP2, MMP9, MTOR, MYC, NOTCH1, PPARG, PTGS2, STAT1, STAT3]
GO:0002237	response to molecule of bacterial origin	GO_BP	9.69E-23	9.94E-22	6.3324537	24	[AKT1, CASP3, CASP8, CCL2, CDK4, CHUK, FOS, GSTP1, HMGB1, IL1B, IL6, JUN, MAPK1, MAPK14, MAPK3, NFKBIA, NOS2, NOS3, NOTCH1, PRKCA, PTGS2, RELA, SRC, TLR4]
GO:0045596	negative regulation of cell differentiation	GO_BP	1.33E-22	1.35E-21	4.2151165	29	[APP, CCND1, CDK5, CDK6, EGFR, ERBB2, EZH2, FOXO1, GSK3B, HMGB1, IGF1, IL1B, IL6, KRAS, MAPK1, MMP9, MYC, NFATC1, NFE2L2, NFKBIA, NOTCH1, PPARA, PPARG, RB1, STAT1, STAT3, TLR4, TP53, VEGFA]
GO:0008134	transcription factor binding	GO_MF	1.46E-22	1.47E-21	4.501608	28	[AHR, CCND1, ESR1, EZH2, FOS, FOXO1, GSK3B, HMGB1, JUN, KEAP1, MAPK14, MTOR, MYC, NFATC1, NFE2L2, NFKBIA, NR1I2, PARP1, PPARA, PPARG, RB1, RELA, RXRA, SMAD2, SRC, STAT1, STAT3, TP53]
GO:0002521	leukocyte differentiation	GO_BP	3.46E-22	3.39E-21	4.6875	27	[APP, ATM, CASP8, CDK2, CDK4, CDK6, ERBB2, EZH2, FOS, HMGB1, IL1B, IL6, JUN, MAPK14, MMP9, MTOR, MYC, PARP1, PPARG, PRKCA, RB1, RXRA, SRC, STAT3, TLR4, TP53, VEGFA]

GO:0009611	response to wounding	GO_BP	3.45E-22	3.42E-21	4.073034	29	[CASP3, CCNA2, CDK1, CDK5, EGFR, ERBB2, FN1, HMGB1, HMOX1, IGF1, IL6, INS, MAP2K1, MAPK1, MAPK14, MAPK3, MTOR, MYC, NFE2L2, NOS3, PDPK1, PPARA, PRKCA, PRKCD, PTK2, SMAD2, SRC, TLR4, VEGFA]
KEGG:04510	Focal adhesion	KEGG	8.09E-20	6.19E-19	8.955224	18	[AKT1, CCND1, EGFR, ERBB2, FN1, GSK3B, IGF1, IGF1R, JUN, MAP2K1, MAPK1, MAPK3, MAPK8, PDPK1, PRKCA, PTK2, SRC, VEGFA]
GO:0071375	cellular response to peptide hormone stimulus	GO_BP	5.88E-22	5.63E-21	6.442577	23	[AKT1, CCNA2, CDK4, FOXO1, GSK3B, GSTP1, IGF1R, IL1B, INS, IRS1, MYC, NFE2L2, NFKB1, PARP1, PDPK1, PPARG, PRKCA, PRKCD, PTK2, RELA, SRC, STAT1, STAT3]
GO:0010942	positive regulation of cell death	GO_BP	6.95E-22	6.58E-21	4.248862	28	[AKT1, ATM, BCL2L1, CASP3, CASP8, CCL2, CCNA2, CDK4, CDK5, FOS, FOXO1, GSK3B, HMGB1, HMOX1, IL6, JUN, MAPK8, MMP9, MTOR, NOS2, NOTCH1, PARP1, PPARG, PRKCD, PTGS2, SRC, TLR4, TP53]
GO:0032496	response to lipopolysaccharide	GO_BP	7.12E-22	6.66E-21	6.388889	23	[AKT1, CASP3, CASP8, CCL2, CDK4, CHUK, FOS, GSTP1, HMGB1, IL1B, JUN, MAPK1, MAPK14, MAPK3, NFKBIA, NOS2, NOS3, NOTCH1, PRKCA, PTGS2, RELA, SRC, TLR4]
KEGG:05171	Coronavirus disease	KEGG	1.09E-18	7.57E-18	7.7586207	18	[CCL2, CHUK, EGFR, FOS, IL1B, IL6, JUN, MAPK1, MAPK14, MAPK3, MAPK8, NFKB1, NFKBIA, PRKCA, RELA, STAT1, STAT3, TLR4]
GO:0050673	epithelial cell proliferation	GO_BP	2.13E-21	1.95E-20	5.5555553	24	[AKT1, CCL2, CCND1, CDK6, EGFR, ERBB2, ESR1, HMGB1, HMOX1, IGF1, IL6, MAP2K1, MAPK1, MTOR, MYC, NOTCH1, PDPK1, PPARG, PRKCA, RB1, STAT1, STAT3, TLR4, VEGFA]
KEGG:04660	T cell receptor signaling pathway	KEGG	2.31E-23	2.74E-22	16.346153	17	[AKT1, CDK4, CHUK, FOS, GSK3B, JUN, KRAS, MAP2K1, MAPK1, MAPK14, MAPK3, MAPK8, NFATC1, NFKB1, NFKBIA, PDPK1, RELA]
KEGG:04620	Toll-like receptor signaling pathway	KEGG	2.31E-23	2.74E-22	16.346153	17	[AKT1, CASP8, CHUK, FOS, IL1B, IL6, JUN, MAP2K1, MAPK1, MAPK14, MAPK3, MAPK8, NFKB1, NFKBIA, RELA, STAT1, TLR4]

KEGG:04915	Estrogen signaling pathway	KEGG	3.65E-21	3.21E-20	12.318841	17	[AKT1, EGFR, ESR1, ESR2, FOS, HSP90AA1, HSPA8, JUN, KRAS, MAP2K1, MAPK1, MAPK3, MMP2, MMP9, NOS3, PRKCD, SRC]
KEGG:05226	Gastric cancer	KEGG	1.41E-20	1.16E-19	11.409396	17	[AKT1, CCND1, CDH1, CDK2, EGFR, ERBB2, GSK3B, KRAS, MAP2K1, MAPK1, MAPK3, MTOR, MYC, RB1, RXRA, SMAD2, TP53]
GO:0030099	myeloid cell differentiation	GO_BP	4.28E-21	3.71E-20	5.3932586	24	[APP, CASP3, CASP8, CDK2, CDK4, CDK6, FOS, HMGB1, JUN, MAPK14, MMP9, MTOR, MYC, NFKBIA, PARP1, PPARG, PRKCA, RB1, RXRA, SRC, STAT1, STAT3, TLR4, VEGFA]
GO:0097190	apoptotic signaling pathway	GO_BP	4.39E-21	3.77E-20	4.2519684	27	[AKT1, ATM, BCL2L1, CASP3, CASP8, GSK3B, GSTP1, HMOX1, IGF1, IL1B, IL6, INS, JUN, MAPK8, MMP9, NFE2L2, NOS3, PARP1, PDPK1, PRKCA, PRKCD, PTGS2, RB1, RELA, SRC, TLR4, TP53]
KEGG:05415	Diabetic cardiomyopathy	KEGG	2.99E-18	2.03E-17	8.374384	17	[AKT1, GSK3B, INS, IRS1, MAPK14, MAPK8, MMP2, MMP9, MTOR, NFKB1, NOS3, PARP1, PPARA, PRKCA, PRKCD, RELA, SMAD2]
KEGG:05219	Bladder cancer	KEGG	6.59E-29	1.59E-27	39.02439	16	[CCND1, CDH1, CDK4, EGFR, ERBB2, KRAS, MAP2K1, MAPK1, MAPK3, MMP2, MMP9, MYC, RB1, SRC, TP53, VEGFA]
KEGG:05223	Non-small cell lung cancer	KEGG	2.40E-24	3.16E-23	22.222221	16	[AKT1, CCND1, CDK4, CDK6, EGFR, ERBB2, KRAS, MAP2K1, MAPK1, MAPK3, PDPK1, PRKCA, RB1, RXRA, STAT3, TP53]
KEGG:05220	Chronic myeloid leukemia	KEGG	6.24E-24	7.84E-23	21.052631	16	[AKT1, BCL2L1, CCND1, CDK4, CDK6, CHUK, KRAS, MAP2K1, MAPK1, MAPK3, MYC, NFKB1, NFKBIA, RB1, RELA, TP53]
KEGG:05210	Colorectal cancer	KEGG	5.41E-23	5.76E-22	18.60465	16	[AKT1, CASP3, CCND1, EGFR, FOS, GSK3B, JUN, KRAS, MAP2K1, MAPK1, MAPK3, MAPK8, MTOR, MYC, SMAD2, TP53]
KEGG:04914	Progesterone-mediated oocyte maturation	KEGG	7.22E-22	6.68E-21	16	16	[AKT1, AURKA, CCNA2, CCNB1, CDK1, CDK2, HSP90AA1, IGF1, IGF1R, INS, KRAS, MAP2K1, MAPK1, MAPK14, MAPK3, MAPK8]

GO:0002573	myeloid leukocyte differentiation	GO_BP	2.29E-20	1.85E-19	8.40708	19	[APP, CASP8, CDK2, CDK4, CDK6, FOS, JUN, MAPK14, MMP9, MTOR, MYC, PARP1, PPARG, PRKCA, RB1, RXRA, SRC, TLR4, VEGFA]
GO:0009612	response to mechanical stimulus	GO_BP	4.10E-20	3.29E-19	8.154507	19	[AKT1, CASP8, CCNB1, EGFR, FOS, IL1B, JUN, MAPK14, MAPK3, MAPK8, NFKB1, NFKBIA, PPARG, PTGS2, PTK2, RELA, SRC, STAT1, TLR4]
KEGG:04931	Insulin resistance	KEGG	2.67E-21	2.41E-20	14.814815	16	[AKT1, FOXO1, GSK3B, IL6, INS, IRS1, MAPK8, MTOR, NFKB1, NFKBIA, NOS3, PDPK1, PPARA, PRKCD, RELA, STAT3]
KEGG:04935	Growth hormone synthesis, secretion and action	KEGG	1.36E-20	1.14E-19	13.445378	16	[AKT1, FOS, GSK3B, IGF1, IRS1, KRAS, MAP2K1, MAPK1, MAPK14, MAPK3, MAPK8, MTOR, PRKCA, PTK2, STAT1, STAT3]
GO:0010001	glial cell differentiation	GO_BP	5.68E-20	4.43E-19	8.016877	19	[AKT1, APP, CDK1, CDK5, CDK6, EGFR, ERBB2, GSTP1, IL6, KRAS, MAP2K1, MAPK1, MAPK3, MTOR, NOTCH1, PPARG, RELA, STAT3, TLR4]
GO:0042493	response to drug	GO_BP	7.30E-20	5.64E-19	5.20362	23	[CASP3, CAT, CCNB1, CCND1, CDH1, CDK1, CDK4, CHUK, EGFR, FOS, HMOX1, JUN, MYC, NFE2L2, NOS2, NR1I2, PPARG, PTGS2, RELA, SRC, STAT1, STAT3, TP53]
KEGG:04722	Neurotrophin signaling pathway	KEGG	1.36E-20	1.14E-19	13.445378	16	[AKT1, GSK3B, IRS1, JUN, KRAS, MAP2K1, MAPK1, MAPK14, MAPK3, MAPK8, NFKB1, NFKBIA, PDPK1, PRKCD, RELA, TP53]
GO:0071241	cellular response to inorganic substance	GO_BP	8.46E-20	6.42E-19	7.8512397	19	[AKT1, APP, CCNA2, CCNB1, CDH1, CDK2, CHUK, EGFR, FOS, FOXO1, HMOX1, JUN, MAPK1, MAPK3, MAPK8, MMP9, NFE2L2, PARP1, PTGS2]
GO:0042060	wound healing	GO_BP	1.02E-19	7.65E-19	4.347826	25	[CASP3, CCNA2, EGFR, ERBB2, FN1, HMGB1, HMOX1, IGF1, IL6, INS, MAPK1, MAPK14, MAPK3, MTOR, NFE2L2, NOS3, PDPK1, PPARA, PRKCA, PRKCD, PTK2, SMAD2, SRC, TLR4, VEGFA]
KEGG:04380	Osteoclast differentiation	KEGG	4.61E-20	3.66E-19	12.5	16	[AKT1, CHUK, FOS, IL1B, JUN, MAP2K1, MAPK1, MAPK14, MAPK3, MAPK8, NFATC1, NFKB1, NFKBIA, PPARG, RELA, STAT1]

GO:0140297	DNA-binding transcription factor binding	GO_MF	1.80E-19	1.33E-18	5.4726367	22	[ESR1, FOS, GSK3B, HMGB1, JUN, MAPK14, MYC, NFATC1, NFE2L2, NFKBIA, NR1I2, PARP1, PPARA, PPARG, RB1, RELA, RXRA, SMAD2, SRC, STAT1, STAT3, TP53]
GO:0010038	response to metal ion	GO_BP	3.22E-19	2.36E-18	5.3268766	22	[AKT1, APP, CASP3, CASP8, CAT, CCNB1, CCND1, CDH1, CDK1, CDK4, CHUK, EGFR, FOS, HMOX1, JUN, MAPK1, MAPK3, MAPK8, MMP9, NFE2L2, PARP1, PTGS2]
KEGG:04062	Chemokine signaling pathway	KEGG	3.50E-17	2.04E-16	8.333333	16	[AKT1, CCL2, CHUK, GSK3B, KRAS, MAP2K1, MAPK1, MAPK3, NFKB1, NFKBIA, PRKCD, PTK2, RELA, SRC, STAT1, STAT3]
GO:0001525	angiogenesis	GO_BP	5.18E-19	3.73E-18	4.3875685	24	[AKT1, CASP8, CCL2, CCNA2, ERBB2, FN1, HMGB1, HMOX1, IL1B, IL6, JUN, MAPK14, MMP2, NFE2L2, NOS3, NOTCH1, PDPK1, PPARG, PRKCA, PTGS2, PTK2, STAT1, STAT3, VEGFA]
GO:0032868	response to insulin	GO_BP	5.49E-19	3.92E-18	6.3492064	20	[AKT1, CAT, CDK4, FOXO1, GSK3B, GSTP1, IGF1R, IL1B, INS, IRS1, MTOR, MYC, PARP1, PDPK1, PPARA, PPARG, PRKCD, RELA, SRC, STAT1]
KEGG:05130	Pathogenic Escherichia coli infection	KEGG	5.28E-17	3.01E-16	8.121827	16	[CASP3, CASP8, CHUK, FOS, IL1B, IL6, JUN, MAPK1, MAPK14, MAPK3, MAPK8, NFKB1, NFKBIA, RELA, SRC, TLR4]
GO:0071496	cellular response to external stimulus	GO_BP	6.10E-19	4.32E-18	5.6910567	21	[AKT1, CASP8, EGFR, FOS, FOXO1, GSTP1, HMOX1, HSPA8, IL1B, JUN, MAPK1, MAPK3, MAPK8, MTOR, NFE2L2, NFKB1, PPARA, PPARG, PTGS2, TLR4, TP53]
GO:0051098	regulation of binding	GO_BP	8.49E-19	5.96E-18	5.6	21	[AKT1, APP, AURKA, CDK5, GSK3B, HMGB1, HMOX1, IGF1, JUN, MAPK3, MAPK8, MMP9, MYC, NFKBIA, PARP1, PPARA, PPARG, PRKCA, PRKCD, SMAD2, SRC]
KEGG:04014	Ras signaling pathway	KEGG	7.17E-16	3.60E-15	6.8965516	16	[AKT1, BCL2L1, CHUK, EGFR, IGF1, IGF1R, INS, KRAS, MAP2K1, MAPK1, MAPK3, MAPK8, NFKB1, PRKCA, RELA, VEGFA]
GO:0009314	response to radiation	GO_BP	1.69E-18	1.16E-17	4.518664	23	[AKT1, APP, ATM, BCL2L1, CASP3, CAT, CCND1, CDK5, EGFR, FOS, H2AX, JUN, KRAS, MAPK14,

							MMP2, MMP9, MTOR, MYC, PARP1, PRKCD, PTGS2, RELA, TP53]
KEGG:05214	Glioma	KEGG	4.10E-22	3.96E-21	20	15	[AKT1, CCND1, CDK4, CDK6, EGFR, IGF1, IGF1R, KRAS, MAP2K1, MAPK1, MAPK3, MTOR, PRKCA, RB1, TP53]
KEGG:04012	ErbB signaling pathway	KEGG	3.13E-21	2.81E-20	17.647058	15	[AKT1, EGFR, ERBB2, GSK3B, JUN, KRAS, MAP2K1, MAPK1, MAPK3, MAPK8, MTOR, MYC, PRKCA, PTK2, SRC]
KEGG:04932	Non-alcoholic fatty liver disease	KEGG	2.33E-17	1.39E-16	10	15	[AKT1, CASP3, CASP8, FOS, GSK3B, IL1B, IL6, INS, IRS1, JUN, MAPK8, NFKB1, PPARA, RELA, RXRA]
GO:0051403	stress-activated MAPK cascade	GO_BP	3.14E-18	2.10E-17	6.4846416	19	[AKT1, APP, CHUK, EGFR, FOXO1, GSTP1, HMGB1, IGF1R, IL1B, IL6, MAP2K1, MAPK1, MAPK14, MAPK3, MAPK8, MYC, NFKB1, TLR4, VEGFA]
GO:1902532	negative regulation of intracellular signal transduction	GO_BP	3.95E-18	2.62E-17	4.347826	23	[AKT1, ATM, BCL2L1, CASP8, ESR1, FOXO1, GSK3B, GSTP1, IGF1R, IL1B, INS, MAPK14, MMP9, MTOR, MYC, NFE2L2, PPARA, PRKCD, PTGS2, RELA, SRC, STAT1, TLR4]
GO:0006352	DNA-templated transcription, initiation	GO_BP	4.05E-18	2.67E-17	6.3973064	19	[CCNB1, CCND1, CDK1, CDK4, ESR1, ESR2, HMGB1, IL6, JUN, MAPK3, MYC, NFKB1, NOTCH1, NR1I2, PPARA, PPARG, RELA, RXRA, TP53]
GO:0009266	response to temperature stimulus	GO_BP	4.16E-18	2.72E-17	7.2	18	[AKT1, ATM, CASP8, FOS, FOXO1, GSK3B, HMOX1, HSP90AA1, HSPA8, IGF1, MAPK1, MAPK3, MTOR, NFKBIA, NOS3, PPARG, PRKCA, PTGS2]
GO:0031098	stress-activated protein kinase signaling cascade	GO_BP	5.21E-18	3.37E-17	6.3122926	19	[AKT1, APP, CHUK, EGFR, FOXO1, GSTP1, HMGB1, IGF1R, IL1B, IL6, MAP2K1, MAPK1, MAPK14, MAPK3, MAPK8, MYC, NFKB1, TLR4, VEGFA]
KEGG:05152	Tuberculosis	KEGG	3.71E-16	1.94E-15	8.333333	15	[AKT1, CASP3, CASP8, IL1B, IL6, MAPK1, MAPK14, MAPK3, MAPK8, NFKB1, NOS2, RELA, SRC, STAT1, TLR4]

GO:0070482	response to oxygen levels	GO_BP	6.42E-18	4.09E-17	5.072464	21	[AKT1, CASP3, CAT, CCNA2, CCNB1, CDK4, FOXO1, HMOX1, MMP2, MTOR, MYC, NFE2L2, NOS2, NOTCH1, PDPK1, PPARA, PPARG, PTGS2, SRC, TP53, VEGFA]
GO:0016572	histone phosphorylation	GO_BP	6.84E-18	4.33E-17	26.190475	11	[ATM, AURKA, CCNA2, CCNB1, CDK1, CDK2, CDK5, IL1B, MAPK3, PRKCA, PRKCD]
KEGG:05202	Transcriptional misregulation in cancer	KEGG	9.78E-16	4.79E-15	7.8125	15	[ATM, BCL2L1, CCNA2, FOXO1, IGF1, IGF1R, IL6, MMP9, MYC, NFKB1, PPARG, PTK2, RELA, RXRA, TP53]
GO:0071900	regulation of protein serine/threonine kinase activity	GO_BP	8.59E-18	5.36E-17	4.19708	23	[AKT1, CASP3, CCNA2, CCNB1, CCND1, EGFR, ERBB2, EZH2, GSTP1, IGF1, IGF1R, IL1B, IL6, KRAS, MAP2K1, MAPK1, MAPK14, MAPK3, PRKCD, RB1, SRC, TLR4, VEGFA]
GO:0050678	regulation of epithelial cell proliferation	GO_BP	1.16E-17	7.18E-17	5.4347825	20	[AKT1, CCL2, CCND1, CDK6, EGFR, ERBB2, HMGB1, HMOX1, IGF1, MTOR, MYC, NOTCH1, PDPK1, PPARG, PRKCA, RB1, STAT1, STAT3, TLR4, VEGFA]
GO:0061629	RNA polymerase II-specific DNA-binding transcription factor binding	GO_MF	1.73E-17	1.06E-16	5.919003	19	[ESR1, FOS, GSK3B, JUN, MAPK14, NFATC1, NFE2L2, NFKBIA, NR1I2, PARP1, PPARA, PPARG, RB1, RELA, RXRA, SRC, STAT1, STAT3, TP53]
GO:0070997	neuron death	GO_BP	1.85E-17	1.13E-16	5.30504	20	[AKT1, APP, BCL2L1, CASP3, CASP8, CCL2, CDK5, FOS, GSK3B, HMOX1, JUN, KRAS, MTOR, PARP1, PDPK1, PPARA, RB1, STAT3, TLR4, TP53]
GO:0104004	cellular response to environmental stimulus	GO_BP	2.16E-17	1.31E-16	5.263158	20	[AKT1, ATM, BCL2L1, CASP3, CASP8, EGFR, H2AX, IL1B, MAPK14, MAPK3, MAPK8, MMP2, MMP9, MYC, NFKB1, PARP1, PRKCD, PTGS2, TLR4, TP53]
GO:0071214	cellular response to abiotic stimulus	GO_BP	2.16E-17	1.31E-16	5.263158	20	[AKT1, ATM, BCL2L1, CASP3, CASP8, EGFR, H2AX, IL1B, MAPK14, MAPK3, MAPK8, MMP2, MMP9, MYC, NFKB1, PARP1, PRKCD, PTGS2, TLR4, TP53]
KEGG:05218	Melanoma	KEGG	1.66E-20	1.36E-19	19.444445	14	[AKT1, CCND1, CDH1, CDK4, CDK6, EGFR, IGF1, IGF1R, KRAS, MAP2K1, MAPK1, MAPK3, RB1, TP53]

KEGG:04140	Autophagy	KEGG	2.15E-16	1.16E-15	10.218978	14	[AKT1, BCL2L1, HMGB1, IGF1R, INS, IRS1, KRAS, MAP2K1, MAPK1, MAPK3, MAPK8, MTOR, PDPK1, PRKCD]
GO:2001233	regulation of apoptotic signaling pathway	GO_BP	2.52E-17	1.49E-16	5.221932	20	[AKT1, BCL2L1, CASP8, GSK3B, GSTP1, HMOX1, IGF1, IL1B, IL6, INS, MMP9, NFE2L2, NOS3, PARP1, PRKCD, PTGS2, RB1, RELA, SRC, TP53]
GO:0062012	regulation of small molecule metabolic process	GO_BP	3.04E-17	1.79E-16	4.6979866	21	[AKT1, APP, GSK3B, HMGB1, IGF1, IL1B, INS, IRS1, MTOR, MYC, NFKB1, NOS2, NOS3, PARP1, PDPK1, PPARA, PPARG, PTGS2, SRC, STAT3, TP53]
KEGG:04150	mTOR signaling pathway	KEGG	1.25E-15	5.90E-15	9.032258	14	[AKT1, CHUK, GSK3B, IGF1, IGF1R, INS, IRS1, KRAS, MAP2K1, MAPK1, MAPK3, MTOR, PDPK1, PRKCA]
KEGG:04015	Rap1 signaling pathway	KEGG	8.70E-14	3.16E-13	6.6666665	14	[AKT1, CDH1, EGFR, IGF1, IGF1R, INS, KRAS, MAP2K1, MAPK1, MAPK14, MAPK3, PRKCA, SRC, VEGFA]
GO:0032147	activation of protein kinase activity	GO_BP	4.37E-17	2.52E-16	5.0761423	20	[AKT1, CCNB1, EGFR, HMGB1, IGF1, IGF1R, IL1B, IL6, INS, MAP2K1, MAPK1, MAPK14, MAPK3, MTOR, PDPK1, PRKCA, PRKCD, SRC, TLR4, VEGFA]
GO:0062013	positive regulation of small molecule metabolic process	GO_BP	4.67E-17	2.67E-16	9.55414	15	[AKT1, APP, HMGB1, IGF1, IL1B, INS, IRS1, MYC, NOS2, NOS3, PPARA, PPARG, PTGS2, SRC, STAT3]
KEGG:05213	Endometrial cancer	KEGG	5.51E-20	4.34E-19	22.413794	13	[AKT1, CCND1, CDH1, EGFR, ERBB2, GSK3B, KRAS, MAP2K1, MAPK1, MAPK3, MYC, PDPK1, TP53]
GO:0070371	ERK1 and ERK2 cascade	GO_BP	5.32E-17	3.01E-16	5.5718474	19	[APP, CCL2, CCNA2, CDK1, EGFR, ERBB2, FN1, GSTP1, HMGB1, IGF1, IL1B, MAP2K1, MAPK1, MAPK3, MYC, NOTCH1, PRKCA, SRC, TLR4]
GO:0032869	cellular response to insulin stimulus	GO_BP	8.36E-17	4.70E-16	6.8825912	17	[AKT1, CDK4, FOXO1, GSK3B, GSTP1, IGF1R, IL1B, INS, IRS1, MYC, PARP1, PDPK1, PPARG, PRKCD, RELA, SRC, STAT1]
KEGG:05221	Acute myeloid leukemia	KEGG	4.31E-	3.13E-18	19.402985	13	[AKT1, CCNA2, CCND1, CHUK, KRAS, MAP2K1, MAPK1, MAPK3, MTOR, MYC, NFKB1, RELA,

			19				STAT3]
KEGG:05133	Pertussis	KEGG	2.52E-18	1.73E-17	17.105263	13	[CASP3, FOS, IL1B, IL6, JUN, MAPK1, MAPK14, MAPK3, MAPK8, NFKB1, NOS2, RELA, TLR4]
GO:0071248	cellular response to metal ion	GO_BP	1.59E-16	8.73E-16	7.582938	16	[AKT1, APP, CCNB1, CDH1, CHUK, EGFR, FOS, HMOX1, JUN, MAPK1, MAPK3, MAPK8, MMP9, NFE2L2, PARP1, PTGS2]
KEGG:05140	Leishmaniasis	KEGG	3.03E-18	2.04E-17	16.883118	13	[FOS, IL1B, JUN, MAPK1, MAPK14, MAPK3, NFKB1, NFKBIA, NOS2, PTGS2, RELA, STAT1, TLR4]
GO:2001234	negative regulation of apoptotic signaling pathway	GO_BP	1.63E-16	8.91E-16	6.614786	17	[AKT1, BCL2L1, CASP8, GSK3B, GSTP1, HMOX1, IGF1, IL1B, IL6, INS, MMP9, NFE2L2, NOS3, PTGS2, RB1, RELA, SRC]
KEGG:04662	B cell receptor signaling pathway	KEGG	7.24E-18	4.55E-17	15.853659	13	[AKT1, CHUK, FOS, GSK3B, JUN, KRAS, MAP2K1, MAPK1, MAPK3, NFATC1, NFKB1, NFKBIA, RELA]
GO:1903829	positive regulation of cellular protein localization	GO_BP	2.52E-16	1.35E-15	5.714286	18	[AKT1, CASP8, CDH1, CDK1, CDK5, EGFR, ERBB2, GSK3B, INS, MAPK1, MAPK14, MAPK8, PARP1, PDPK1, PRKCD, PTGS2, SRC, TP53]
GO:0044843	cell cycle G1/S phase transition	GO_BP	2.56E-16	1.36E-15	6.439394	17	[AKT1, ATM, AURKA, CCL2, CCNA2, CCNB1, CCND1, CDK1, CDK2, CDK4, CDK5, CDK6, EGFR, EZH2, MYC, RB1, TP53]
GO:0043467	regulation of generation of precursor metabolites and energy	GO_BP	2.88E-16	1.52E-15	8.474576	15	[AKT1, APP, CCNB1, CDK1, GSK3B, HMGB1, IGF1, INS, IRS1, MTOR, MYC, NOS2, PPARA, STAT3, TP53]
GO:0043491	protein kinase B signaling	GO_BP	3.09E-16	1.63E-15	6.367041	17	[AKT1, CCL2, EGFR, ERBB2, ESR1, HSP90AA1, IGF1, IGF1R, IL1B, IL6, INS, IRS1, MTOR, PDPK1, PPARA, PTK2, SRC]
KEGG:04211	Longevity regulating pathway	KEGG	2.23E-17	1.34E-16	14.606742	13	[AKT1, CAT, FOXO1, IGF1, IGF1R, INS, IRS1, KRAS, MTOR, NFKB1, PPARG, RELA, TP53]
GO:0046686	response to cadmium ion	GO_BP	4.65E-16	2.42E-15	14.457831	12	[AKT1, CAT, CDK1, CHUK, EGFR, FOS, HMOX1, JUN, MAPK1, MAPK3, MAPK8, MMP9]
GO:0048660	regulation of smooth muscle cell proliferation	GO_BP	4.84E-16	2.50E-15	9.655172	14	[AKT1, EGFR, GSTP1, HMOX1, IGF1, IL6, JUN, MMP2, MMP9, MTOR, MYC, PPARG, PTGS2,

							STAT1]
GO:0006367	transcription initiation from RNA polymerase II promoter	GO_BP	6.25E-16	3.21E-15	6.9565215	16	[CCNB1, CCND1, CDK1, CDK4, ESR1, ESR2, HMGB1, IL6, NFKB1, NOTCH1, NR1I2, PPARA, PPARG, RELA, RXRA, TP53]
GO:1901214	regulation of neuron death	GO_BP	6.33E-16	3.23E-15	5.4216866	18	[AKT1, BCL2L1, CASP3, CASP8, CCL2, CDK5, FOS, GSK3B, HMOX1, JUN, KRAS, MTOR, PARP1, PDPK1, PPARA, STAT3, TLR4, TP53]
GO:0048659	smooth muscle cell proliferation	GO_BP	6.47E-16	3.28E-15	9.459459	14	[AKT1, EGFR, GSTP1, HMOX1, IGF1, IL6, JUN, MMP2, MMP9, MTOR, MYC, PPARG, PTGS2, STAT1]
GO:0097305	response to alcohol	GO_BP	6.84E-16	3.45E-15	6.071429	17	[AHR, AKT1, BCL2L1, CASP8, CAT, CCND1, CDH1, CDK1, CDK4, FOS, GSTP1, MYC, PARP1, PPARA, PPARG, SMAD2, STAT3]
KEGG:04658	Th1 and Th2 cell differentiation	KEGG	3.51E-17	2.04E-16	14.130435	13	[CHUK, FOS, JUN, MAPK1, MAPK14, MAPK3, MAPK8, NFATC1, NFKB1, NFKBIA, NOTCH1, RELA, STAT1]
GO:0051091	positive regulation of DNA-binding transcription factor activity	GO_BP	7.71E-16	3.84E-15	6.028369	17	[AKT1, APP, CAT, CHUK, ESR1, ESR2, IL1B, IL6, INS, KRAS, NFKB1, NFKBIA, PPARG, RELA, STAT3, TLR4, VEGFA]
GO:0031331	positive regulation of cellular catabolic process	GO_BP	8.25E-16	4.08E-15	4.3572984	20	[AKT1, APP, AURKA, FOXO1, GSK3B, HMGB1, HMOX1, HSP90AA1, IGF1, IL1B, IL6, INS, IRS1, KEAP1, MAPK3, MYC, NFE2L2, PPARA, PRKCD, PTK2]
GO:0032872	regulation of stress-activated MAPK cascade	GO_BP	8.36E-16	4.12E-15	7.894737	15	[AKT1, APP, EGFR, FOXO1, GSTP1, HMGB1, IGF1R, IL1B, IL6, MAP2K1, MAPK1, MAPK3, MYC, TLR4, VEGFA]
KEGG:04071	Sphingolipid signaling pathway	KEGG	1.13E-15	5.44E-15	10.92437	13	[AKT1, KRAS, MAP2K1, MAPK1, MAPK14, MAPK3, MAPK8, NFKB1, NOS3, PDPK1, PRKCA, RELA, TP53]
GO:0070302	regulation of stress-activated protein kinase signaling cascade	GO_BP	1.06E-15	5.15E-15	7.772021	15	[AKT1, APP, EGFR, FOXO1, GSTP1, HMGB1, IGF1R, IL1B, IL6, MAP2K1, MAPK1, MAPK3, MYC, TLR4, VEGFA]

GO:0010632	regulation of epithelial cell migration	GO_BP	1.07E-15	5.20E-15	6.722689	16	[AKT1, HMGB1, HMOX1, MAPK14, MMP9, MTOR, NFE2L2, NOS3, NOTCH1, PDPK1, PPARG, PRKCA, PTGS2, PTK2, SRC, VEGFA]
KEGG:04110	Cell cycle	KEGG	1.96E-15	9.05E-15	10.4838705	13	[ATM, CCNA2, CCNB1, CCND1, CDK1, CDK2, CDK4, CDK6, GSK3B, MYC, RB1, SMAD2, TP53]
GO:0071276	cellular response to cadmium ion	GO_BP	1.14E-15	5.44E-15	22.727272	10	[AKT1, CHUK, EGFR, FOS, HMOX1, JUN, MAPK1, MAPK3, MAPK8, MMP9]
GO:0000082	G1/S transition of mitotic cell cycle	GO_BP	1.23E-15	5.84E-15	6.6666665	16	[AKT1, ATM, AURKA, CCL2, CCNB1, CCND1, CDK1, CDK2, CDK4, CDK5, CDK6, EGFR, EZH2, MYC, RB1, TP53]
KEGG:04921	Oxytocin signaling pathway	KEGG	3.40E-14	1.34E-13	8.441559	13	[CCND1, EGFR, FOS, JUN, KRAS, MAP2K1, MAPK1, MAPK3, NFATC1, NOS3, PRKCA, PTGS2, SRC]
GO:0048661	positive regulation of smooth muscle cell proliferation	GO_BP	1.48E-15	6.95E-15	13.186813	12	[AKT1, EGFR, HMOX1, IGF1, IL6, JUN, MMP2, MMP9, MTOR, MYC, PTGS2, STAT1]
GO:0031100	animal organ regeneration	GO_BP	1.48E-15	6.95E-15	13.186813	12	[CCNA2, CCND1, CDK1, CDK4, EGFR, EZH2, GSTP1, HMOX1, IL6, MYC, NOTCH1, PPARG]
GO:2000045	regulation of G1/S transition of mitotic cell cycle	GO_BP	1.63E-15	7.65E-15	8.86076	14	[AKT1, ATM, AURKA, CCL2, CCNB1, CCND1, CDK1, CDK2, CDK4, CDK6, EGFR, EZH2, RB1, TP53]
KEGG:04613	Neutrophil extracellular trap formation	KEGG	5.16E-13	1.75E-12	6.8421054	13	[AKT1, H2AX, HMGB1, MAP2K1, MAPK1, MAPK14, MAPK3, MTOR, NFKB1, PRKCA, RELA, SRC, TLR4]
KEGG:04370	VEGF signaling pathway	KEGG	5.79E-18	3.72E-17	20.338984	12	[AKT1, KRAS, MAP2K1, MAPK1, MAPK14, MAPK3, NOS3, PRKCA, PTGS2, PTK2, SRC, VEGFA]
GO:0071222	cellular response to lipopolysaccharide	GO_BP	2.42E-15	1.11E-14	7.352941	15	[AKT1, CCL2, CDK4, GSTP1, HMGB1, IL1B, MAPK1, MAPK14, MAPK3, NFKBIA, NOS2, PRKCA, RELA, SRC, TLR4]
GO:0046777	protein autophosphorylation	GO_BP	2.49E-15	1.14E-14	6.374502	16	[AKT1, ATM, AURKA, CDK5, EGFR, ERBB2, GSK3B, IGF1R, INS, MAPK3, MTOR, PDPK1, PRKCA, PTK2, SRC, VEGFA]

KEGG:04115	p53 signaling pathway	KEGG	9.10E-17	5.08E-16	16.438356	12	[ATM, BCL2L1, CASP3, CASP8, CCNB1, CCND1, CDK1, CDK2, CDK4, CDK6, IGF1, TP53]
GO:0009416	response to light stimulus	GO_BP	2.73E-15	1.23E-14	4.98615	18	[AKT1, APP, CASP3, CAT, CCND1, CDK5, EGFR, FOS, KRAS, MMP2, MMP9, MTOR, MYC, PARP1, PRKCD, PTGS2, RELA, TP53]
GO:0043535	regulation of blood vessel endothelial cell migration	GO_BP	2.88E-15	1.30E-14	12.5	12	[AKT1, HMGB1, HMOX1, MAPK14, NFE2L2, NOS3, NOTCH1, PDPK1, PPARG, PRKCA, PTGS2, VEGFA]
GO:0014065	phosphatidylinositol 3-kinase signaling	GO_BP	3.28E-15	1.47E-14	8.433735	14	[AKT1, CAT, EGFR, ERBB2, FN1, IGF1, IGF1R, IL6, INS, IRS1, MAPK1, MAPK3, PTK2, SRC]
GO:0071902	positive regulation of protein serine/threonine kinase activity	GO_BP	3.63E-15	1.62E-14	4.904632	18	[AKT1, CCNB1, CCND1, EGFR, ERBB2, EZH2, IGF1, IGF1R, IL1B, IL6, KRAS, MAP2K1, MAPK1, MAPK14, MAPK3, SRC, TLR4, VEGFA]
KEGG:04912	GnRH signaling pathway	KEGG	1.94E-15	9.02E-15	12.903226	12	[EGFR, JUN, KRAS, MAP2K1, MAPK1, MAPK14, MAPK3, MAPK8, MMP2, PRKCA, PRKCD, SRC]
GO:0010631	epithelial cell migration	GO_BP	3.91E-15	1.72E-14	5.466238	17	[AKT1, HMGB1, HMOX1, MAPK14, MMP9, MTOR, NFE2L2, NOS3, NOTCH1, PDPK1, PPARG, PRKCA, PTGS2, PTK2, SRC, STAT1, VEGFA]
GO:0001666	response to hypoxia	GO_BP	4.18E-15	1.83E-14	4.864865	18	[AKT1, CASP3, CAT, CCNA2, CCNB1, HMOX1, MMP2, MTOR, MYC, NFE2L2, NOS2, NOTCH1, PDPK1, PPARA, PTGS2, SRC, TP53, VEGFA]
GO:0050679	positive regulation of epithelial cell proliferation	GO_BP	4.30E-15	1.87E-14	7.075472	15	[AKT1, CCND1, EGFR, ERBB2, HMGB1, HMOX1, IGF1, MTOR, MYC, NOTCH1, PDPK1, PRKCA, STAT3, TLR4, VEGFA]
GO:0004707	MAP kinase activity	GO_BP	4.38E-15	1.90E-14	4.851752	18	[EGFR, ERBB2, EZH2, GSTP1, IGF1, IGF1R, IL1B, IL6, KRAS, MAP2K1, MAPK1, MAPK14, MAPK3, MAPK8, PRKCD, SRC, TLR4, VEGFA]
GO:0090132	epithelium migration	GO_BP	4.58E-15	1.98E-14	5.414013	17	[AKT1, HMGB1, HMOX1, MAPK14, MMP9, MTOR, NFE2L2, NOS3, NOTCH1, PDPK1, PPARG, PRKCA, PTGS2, PTK2, SRC, STAT1, VEGFA]
GO:0071219	cellular response to molecule of bacterial origin	GO_BP	4.94E-15	2.12E-14	7.009346	15	[AKT1, CCL2, CDK4, GSTP1, HMGB1, IL1B, MAPK1, MAPK14, MAPK3, NFKBIA, NOS2, PRKCA, RELA, SRC, TLR4]

GO:1902911	protein kinase complex	GO_CC	6.90E-15	2.95E-14	11.650485	12	[CCNA2, CCNB1, CCND1, CDK1, CDK2, CDK4, CDK5, CDK6, CHUK, IGF1R, IRS1, RB1]
GO:0036293	response to decreased oxygen levels	GO_BP	8.32E-15	3.54E-14	4.6753244	18	[AKT1, CASP3, CAT, CCNA2, CCNB1, HMOX1, MMP2, MTOR, MYC, NFE2L2, NOS2, NOTCH1, PDPK1, PPARA, PTGS2, SRC, TP53, VEGFA]
GO:0031099	regeneration	GO_BP	8.52E-15	3.60E-14	6.756757	15	[CCNA2, CCNB1, CCND1, CDK1, CDK4, EGFR, EZH2, GSTP1, HMOX1, IGF1, IL6, MAP2K1, MYC, NOTCH1, PPARG]
GO:0031349	positive regulation of defense response	GO_BP	8.70E-15	3.66E-14	4.6632123	18	[APP, CHUK, EGFR, HMGB1, HSP90AA1, IL1B, IL6, KRAS, MAPK3, NFKB1, NFKBIA, PDPK1, PRKCA, PRKCD, PTGS2, RELA, SRC, TLR4]
GO:1902806	regulation of cell cycle G1/S phase transition	GO_BP	1.02E-14	4.28E-14	7.7777777	14	[AKT1, ATM, AURKA, CCL2, CCNB1, CCND1, CDK1, CDK2, CDK4, CDK6, EGFR, EZH2, RB1, TP53]
GO:0006109	regulation of carbohydrate metabolic process	GO_BP	1.04E-14	4.33E-14	6.6666665	15	[AKT1, APP, GSK3B, HMGB1, IGF1, INS, IRS1, MTOR, MYC, NFKB1, PDPK1, PPARA, SRC, STAT3, TP53]
GO:0044389	ubiquitin-like protein ligase binding	GO_MF	1.09E-14	4.53E-14	5.1359515	17	[AURKA, CASP8, CCNB1, EGFR, FOXO1, GSK3B, HSP90AA1, HSPA8, JUN, NFKBIA, PRKCA, RB1, RELA, SMAD2, SRC, STAT1, TP53]
GO:0022407	regulation of cell-cell adhesion	GO_BP	1.27E-14	5.25E-14	4.130435	19	[AKT1, CASP3, CCL2, CDH1, ERBB2, HMGB1, IGF1, IL1B, IL6, MAPK14, NOTCH1, PDPK1, PPARA, PRKCA, PRKCD, PTK2, RELA, SRC, VEGFA]
GO:0071216	cellular response to biotic stimulus	GO_BP	1.86E-14	7.62E-14	6.4102564	15	[AKT1, CCL2, CDK4, GSTP1, HMGB1, IL1B, MAPK1, MAPK14, MAPK3, NFKBIA, NOS2, PRKCA, RELA, SRC, TLR4]
GO:2000134	negative regulation of G1/S transition of mitotic cell cycle	GO_BP	1.94E-14	7.92E-14	10.714286	12	[ATM, AURKA, CCL2, CCNB1, CCND1, CDK1, CDK2, CDK4, CDK6, EZH2, RB1, TP53]
GO:0071453	cellular response to oxygen levels	GO_BP	1.98E-14	8.04E-14	6.382979	15	[AKT1, CCNA2, CCNB1, FOXO1, HMOX1, MTOR, MYC, NFE2L2, NOTCH1, PDPK1, PPARG, PTGS2, SRC, TP53, VEGFA]

GO:2001237	negative regulation of extrinsic apoptotic signaling pathway	GO_BP	2.41E-14	9.75E-14	10.526316	12	[AKT1, BCL2L1, CASP8, GSK3B, GSTP1, HMOX1, IGF1, IL1B, IL6, NOS3, RELA, SRC]
GO:0051222	positive regulation of protein transport	GO_BP	2.49E-14	1.00E-13	4.8850574	17	[CASP8, CDH1, CDK1, CDK5, EGFR, ERBB2, IGF1, IL1B, MAPK1, MAPK14, MAPK8, PRKCA, PRKCD, PTGS2, SRC, TLR4, TP53]
GO:1902807	negative regulation of cell cycle G1/S phase transition	GO_BP	2.98E-14	1.19E-13	10.344828	12	[ATM, AURKA, CCL2, CCNB1, CCND1, CDK1, CDK2, CDK4, CDK6, EZH2, RB1, TP53]
GO:0048145	regulation of fibroblast proliferation	GO_BP	2.98E-14	1.19E-13	12.941176	11	[CCNA2, CCNB1, CDK4, CDK6, ESR1, FN1, GSTP1, IGF1, MYC, PPARG, TP53]
GO:0048545	response to steroid hormone	GO_BP	3.03E-14	1.20E-13	4.3373494	18	[CASP3, CCND1, EGFR, ESR1, ESR2, FOS, FOXO1, GSTP1, HMGB1, IL6, KRAS, NOTCH1, PARP1, PPARA, PTGS2, RELA, RXRA, SRC]
GO:0043536	positive regulation of blood vessel endothelial cell migration	GO_BP	3.29E-14	1.30E-13	16.666666	10	[AKT1, HMGB1, HMOX1, MAPK14, NFE2L2, NOS3, PDPK1, PRKCA, PTGS2, VEGFA]
GO:0048144	fibroblast proliferation	GO_BP	3.41E-14	1.33E-13	12.790698	11	[CCNA2, CCNB1, CDK4, CDK6, ESR1, FN1, GSTP1, IGF1, MYC, PPARG, TP53]
KEGG:05231	Choline metabolism in cancer	KEGG	3.72E-15	1.65E-14	12.244898	12	[AKT1, EGFR, FOS, JUN, KRAS, MAP2K1, MAPK1, MAPK3, MAPK8, MTOR, PDPK1, PRKCA]
GO:0048708	astrocyte differentiation	GO_BP	3.89E-14	1.51E-13	12.643678	11	[APP, CDK6, EGFR, IL6, KRAS, MAP2K1, MAPK1, MAPK3, NOTCH1, STAT3, TLR4]
GO:0042542	response to hydrogen peroxide	GO_BP	4.37E-14	1.70E-13	8.280254	13	[CASP3, CAT, CDK1, EZH2, FOXO1, HMOX1, IL6, JUN, NFE2L2, PRKCD, RELA, SRC, STAT1]
GO:2000142	regulation of DNA-templated transcription, initiation	GO_BP	4.43E-14	1.71E-13	12.5	11	[CCNB1, CCND1, CDK1, CDK4, ESR1, HMGB1, IL6, JUN, NFKB1, RELA, TP53]
GO:0043405	regulation of MAP kinase activity	GO_BP	4.53E-14	1.74E-13	4.7091413	17	[EGFR, ERBB2, EZH2, GSTP1, IGF1, IGF1R, IL1B, IL6, KRAS, MAP2K1, MAPK1, MAPK14, MAPK3, PRKCD, SRC, TLR4, VEGFA]
KEGG:04910	Insulin signaling pathway	KEGG	2.26E-13	7.92E-13	8.759124	12	[AKT1, FOXO1, GSK3B, INS, IRS1, KRAS, MAP2K1, MAPK1, MAPK3, MAPK8, MTOR,

							PDPK1]
GO:1904951	positive regulation of establishment of protein localization	GO_BP	4.95E-14	1.89E-13	4.6831956	17	[CASP8, CDH1, CDK1, CDK5, EGFR, ERBB2, IGF1, IL1B, MAPK1, MAPK14, MAPK8, PRKCA, PRKCD, PTGS2, SRC, TLR4, TP53]
GO:0032355	response to estradiol	GO_BP	5.15E-14	1.95E-13	8.176101	13	[CASP3, CASP8, CAT, CCNA2, CCND1, EGFR, ESR1, ESR2, EZH2, GSTP1, MYC, PTGS2, STAT3]
GO:0043534	blood vessel endothelial cell migration	GO_BP	5.52E-14	2.08E-13	9.836065	12	[AKT1, HMGB1, HMOX1, MAPK14, NFE2L2, NOS3, NOTCH1, PDPK1, PPARG, PRKCA, PTGS2, VEGFA]
GO:0019902	phosphatase binding	GO_MF	5.83E-14	2.19E-13	6.8627453	14	[AKT1, EGFR, ERBB2, FOXO1, MAPK1, MAPK14, MAPK3, PPARA, PPARG, PTK2, SMAD2, STAT1, STAT3, TP53]
GO:0010634	positive regulation of epithelial cell migration	GO_BP	6.06E-14	2.27E-13	8.074534	13	[AKT1, HMGB1, HMOX1, MAPK14, MMP9, MTOR, NFE2L2, NOS3, PDPK1, PRKCA, PTGS2, SRC, VEGFA]
GO:0009411	response to UV	GO_BP	6.57E-14	2.45E-13	8.024692	13	[AKT1, CASP3, CAT, CCND1, EGFR, MMP2, MMP9, MYC, PARP1, PRKCD, PTGS2, RELA, TP53]
GO:0018107	peptidyl-threonine phosphorylation	GO_BP	6.73E-14	2.50E-13	9.67742	12	[AKT1, APP, CDK1, CDK5, GSK3B, MAP2K1, MAPK1, MAPK8, MTOR, PDPK1, PRKCA, PRKCD]
GO:0072593	reactive oxygen species metabolic process	GO_BP	6.96E-14	2.57E-13	5.859375	15	[AKT1, CAT, CCNA2, EGFR, GSTP1, INS, MAPK14, NFE2L2, NOS2, NOS3, PPARA, PRKCD, STAT3, TLR4, TP53]
GO:0048015	phosphatidylinositol-mediated signaling	GO_BP	7.13E-14	2.62E-13	6.763285	14	[AKT1, CAT, EGFR, ERBB2, FN1, IGF1, IGF1R, IL6, INS, IRS1, MAPK1, MAPK3, PTK2, SRC]
GO:0045787	positive regulation of cell cycle	GO_BP	7.42E-14	2.71E-13	5.1282053	16	[AKT1, APP, AURKA, CCNB1, CCND1, CDK1, CDK4, EGFR, EZH2, IGF1, IL1B, INS, MYC, PRKCA, RB1, SRC]
KEGG:04550	Signaling pathways regulating pluripotency of stem cells	KEGG	3.78E-13	1.30E-12	8.391608	12	[AKT1, GSK3B, IGF1, IGF1R, KRAS, MAP2K1, MAPK1, MAPK14, MAPK3, MYC, SMAD2, STAT3]
KEGG:04024	cAMP signaling pathway	KEGG	5.01E-	1.30E-10	5.5555553	12	[AKT1, FOS, JUN, MAP2K1, MAPK1, MAPK3,

			11				MAPK8, NFATC1, NFKB1, NFKBIA, PPARA, RELA]
GO:0048017	inositol lipid-mediated signaling	GO_BP	9.29E-14	3.36E-13	6.6350713	14	[AKT1, CAT, EGFR, ERBB2, FN1, IGF1, IGF1R, IL6, INS, IRS1, MAPK1, MAPK3, PTK2, SRC]
GO:0004693	cyclin-dependent protein serine/threonine kinase activity	GO_BP	1.09E-13	3.92E-13	9.302325	12	[AKT1, CASP3, CCNA2, CCNB1, CCND1, CDK1, CDK2, CDK4, CDK5, CDK6, EGFR, SRC]
GO:0097472	cyclin-dependent protein kinase activity	GO_BP	1.58E-13	5.65E-13	9.022556	12	[AKT1, CASP3, CCNA2, CCNB1, CCND1, CDK1, CDK2, CDK4, CDK5, CDK6, EGFR, SRC]
KEGG:04920	Adipocytokine signaling pathway	KEGG	2.68E-15	1.22E-14	15.942029	11	[AKT1, CHUK, IRS1, MAPK8, MTOR, NFKB1, NFKBIA, PPARA, RELA, RXRA, STAT3]
KEGG:04152	AMPK signaling pathway	KEGG	1.45E-12	4.65E-12	9.166667	11	[AKT1, CCNA2, CCND1, FOXO1, IGF1, IGF1R, INS, IRS1, MTOR, PDPK1, PPARG]
GO:0010594	regulation of endothelial cell migration	GO_BP	2.07E-13	7.33E-13	7.3446326	13	[AKT1, HMGB1, HMOX1, MAPK14, NFE2L2, NOS3, NOTCH1, PDPK1, PPARG, PRKCA, PTGS2, PTK2, VEGFA]
GO:0009408	response to heat	GO_BP	2.07E-13	7.33E-13	7.3446326	13	[AKT1, ATM, GSK3B, HMOX1, HSP90AA1, HSPA8, IGF1, MAPK1, MAPK3, MTOR, NOS3, PRKCA, PTGS2]
GO:0018210	peptidyl-threonine modification	GO_BP	2.07E-13	7.34E-13	8.823529	12	[AKT1, APP, CDK1, CDK5, GSK3B, MAP2K1, MAPK1, MAPK8, MTOR, PDPK1, PRKCA, PRKCD]
GO:0034612	response to tumor necrosis factor	GO_BP	2.12E-13	7.47E-13	4.790419	16	[AKT1, CASP3, CASP8, CCL2, CHUK, GSTP1, MAPK1, MAPK14, MAPK3, NFE2L2, NFKB1, NFKBIA, PTGS2, RELA, STAT1, TP53]
KEGG:04114	Oocyte meiosis	KEGG	3.24E-12	9.84E-12	8.527132	11	[AURKA, CCNB1, CDK1, CDK2, IGF1, IGF1R, INS, MAP2K1, MAPK1, MAPK14, MAPK3]
GO:0043406	positive regulation of MAP kinase activity	GO_BP	2.57E-13	8.99E-13	5.357143	15	[EGFR, ERBB2, EZH2, IGF1, IGF1R, IL1B, IL6, KRAS, MAP2K1, MAPK1, MAPK14, MAPK3, SRC, TLR4, VEGFA]
GO:0048638	regulation of developmental growth	GO_BP	2.66E-13	9.27E-13	4.719764	16	[AKT1, APP, CCNB1, CDK1, CDK4, CDK5, FN1, GSK3B, IGF1, MAPK1, MAPK14, MTOR, NOTCH1, PPARA, STAT3, VEGFA]

GO:0043542	endothelial cell migration	GO_BP	3.42E-13	1.19E-12	6.034483	14	[AKT1, HMGB1, HMOX1, MAPK14, NFE2L2, NOS3, NOTCH1, PDPK1, PPARG, PRKCA, PTGS2, PTK2, STAT1, VEGFA]
GO:0034349	glial cell apoptotic process	GO_BP	3.50E-13	1.21E-12	38.88889	7	[CASP3, CCL2, CDK5, PRKCA, PRKCD, RB1, TP53]
KEGG:04371	Apelin signaling pathway	KEGG	6.28E-12	1.84E-11	8.029197	11	[AKT1, CCND1, CDH1, KRAS, MAP2K1, MAPK1, MAPK3, MTOR, NOS2, NOS3, SMAD2]
GO:0010506	regulation of autophagy	GO_BP	3.81E-13	1.30E-12	4.610951	16	[AKT1, ATM, CASP3, CDK5, FOXO1, GSK3B, HMGB1, HMOX1, IL6, KEAP1, MAPK3, MAPK8, MTOR, PRKCA, STAT3, TP53]
GO:0097191	extrinsic apoptotic signaling pathway	GO_BP	4.59E-13	1.56E-12	5.907173	14	[AKT1, BCL2L1, CASP3, CASP8, GSK3B, GSTP1, HMOX1, IGF1, IL1B, IL6, NOS3, PDPK1, RELA, SRC]
KEGG:04934	Cushing syndrome	KEGG	2.42E-11	6.61E-11	7.096774	11	[AHR, CCND1, CDK2, CDK4, CDK6, EGFR, GSK3B, MAP2K1, MAPK1, MAPK3, RB1]
GO:0051896	regulation of protein kinase B signaling	GO_BP	6.45E-13	2.18E-12	5.761317	14	[AKT1, EGFR, ERBB2, ESR1, HSP90AA1, IGF1R, IL6, INS, IRS1, MTOR, PDPK1, PPARA, PTK2, SRC]
GO:1904645	response to amyloid-beta	GO_BP	6.72E-13	2.26E-12	16.666666	9	[APP, CDK5, GSK3B, IGF1, IGF1R, MMP2, MMP9, PARP1, TLR4]
GO:0031960	response to corticosteroid	GO_BP	7.20E-13	2.42E-12	6.6666665	13	[CASP3, CCND1, EGFR, FOS, FOXO1, GSTP1, HMGB1, IL6, KRAS, NOTCH1, PARP1, PTGS2, SRC]
GO:0043500	muscle adaptation	GO_BP	7.44E-13	2.48E-12	9.734513	11	[EZH2, FOXO1, HMOX1, IGF1, IL1B, MTOR, NOS3, NOTCH1, PARP1, PPARA, PRKCA]
GO:0090316	positive regulation of intracellular protein transport	GO_BP	7.69E-13	2.55E-12	6.632653	13	[CASP8, CDH1, CDK1, CDK5, ERBB2, IL1B, MAPK1, MAPK14, MAPK8, PRKCA, PRKCD, PTGS2, TP53]
GO:0060260	regulation of transcription initiation from RNA polymerase II promoter	GO_BP	7.66E-13	2.55E-12	12.345679	10	[CCNB1, CCND1, CDK1, CDK4, ESR1, HMGB1, IL6, NFKB1, RELA, TP53]
GO:0034504	protein localization to nucleus	GO_BP	8.07E-13	2.67E-12	4.9504952	15	[AKT1, CDH1, CDK1, CDK5, GSK3B, INS, MAPK1, MAPK14, NFKBIA, PARP1, PRKCD,

							PTGS2, SRC, STAT3, TP53]
GO:0031983	vesicle lumen	GO_CC	8.96E-13	2.95E-12	4.359673	16	[APP, CAT, EGFR, FN1, GSTP1, HMGB1, HSP90AA1, HSPA8, IGF1, IL6, INS, MAPK1, MAPK14, NFKB1, PRKCD, VEGFA]
GO:0045913	positive regulation of carbohydrate metabolic process	GO_BP	9.86E-13	3.23E-12	12.048193	10	[AKT1, APP, HMGB1, IGF1, INS, IRS1, MYC, NFKB1, PPARA, SRC]
KEGG:05020	Prion disease	KEGG	9.93E-09	1.92E-08	4.029304	11	[CASP3, GSK3B, HSPA8, IL1B, IL6, MAPK1, MAPK14, MAPK3, MAPK8, NOTCH1, PRKCD]
GO:0031625	ubiquitin protein ligase binding	GO_MF	1.23E-12	4.00E-12	4.8076925	15	[AURKA, CASP8, EGFR, FOXO1, GSK3B, HSP90AA1, HSPA8, JUN, NFKBIA, PRKCA, RB1, RELA, SMAD2, SRC, TP53]
GO:0010675	regulation of cellular carbohydrate metabolic process	GO_BP	1.35E-12	4.36E-12	7.5471697	12	[AKT1, GSK3B, HMGB1, IGF1, INS, IRS1, MTOR, PDPK1, PPARA, SRC, STAT3, TP53]
GO:0070372	regulation of ERK1 and ERK2 cascade	GO_BP	1.35E-12	4.37E-12	4.77707	15	[APP, CCL2, CCNA2, EGFR, ERBB2, FN1, GSTP1, HMGB1, IL1B, MAP2K1, MAPK3, NOTCH1, PRKCA, SRC, TLR4]
GO:0071456	cellular response to hypoxia	GO_BP	1.45E-12	4.63E-12	6.3106794	13	[AKT1, CCNA2, CCNB1, HMOX1, MTOR, MYC, NFE2L2, NOTCH1, PDPK1, PTGS2, SRC, TP53, VEGFA]
KEGG:05216	Thyroid cancer	KEGG	1.63E-16	8.86E-16	27.027027	10	[CCND1, CDH1, KRAS, MAP2K1, MAPK1, MAPK3, MYC, PPARG, RXRA, TP53]
KEGG:04213	Longevity regulating pathway	KEGG	4.66E-14	1.78E-13	16.129032	10	[AKT1, CAT, FOXO1, HSPA8, IGF1, IGF1R, INS, IRS1, KRAS, MTOR]
GO:0045637	regulation of myeloid cell differentiation	GO_BP	1.62E-12	5.14E-12	5.3846154	14	[CASP8, CDK6, FOS, HMGB1, JUN, MAPK14, MTOR, MYC, NFKBIA, PRKCA, RB1, STAT1, STAT3, TLR4]
GO:0038093	Fc receptor signaling pathway	GO_BP	1.71E-12	5.40E-12	5.3639846	14	[CHUK, FOS, HSP90AA1, JUN, MAPK1, MAPK3, MAPK8, NFATC1, NFKB1, PDPK1, PRKCD, PTK2, RELA, SRC]
GO:1902554	serine/threonine protein kinase complex	GO_CC	1.80E-12	5.68E-12	11.363636	10	[CCNA2, CCNB1, CCND1, CDK1, CDK2, CDK4, CDK5, CDK6, CHUK, RB1]

GO:0051054	positive regulation of DNA metabolic process	GO_BP	1.86E-12	5.83E-12	6.1904764	13	[AKT1, ATM, EGFR, H2AX, HMGB1, HSP90AA1, IL6, MAPK1, MAPK3, MYC, PARP1, PRKCD, SRC]
GO:0045834	positive regulation of lipid metabolic process	GO_BP	1.95E-12	6.11E-12	7.3170733	12	[AKT1, CCNA2, IL1B, INS, IRS1, MTOR, PPARA, PPARG, PRKCD, PTGS2, PTK2, SRC]
GO:0032386	regulation of intracellular transport	GO_BP	2.09E-12	6.51E-12	4.123711	16	[CASP8, CDH1, CDK1, CDK5, ERBB2, IL1B, MAP2K1, MAPK1, MAPK14, MAPK3, MAPK8, PRKCA, PRKCD, PTGS2, SRC, TP53]
GO:0031663	lipopolysaccharide-mediated signaling pathway	GO_BP	2.14E-12	6.66E-12	14.754098	9	[AKT1, CCL2, IL1B, MAPK1, MAPK14, MAPK3, NFKBIA, PRKCA, TLR4]
GO:1900182	positive regulation of protein localization to nucleus	GO_BP	2.27E-12	7.02E-12	11.111111	10	[AKT1, CDH1, CDK1, INS, MAPK1, MAPK14, PARP1, PRKCD, PTGS2, SRC]
GO:0004517	nitric-oxide synthase activity	GO_MF	2.50E-12	7.71E-12	14.5161295	9	[AKT1, EGFR, ESR1, HSP90AA1, IL1B, INS, KRAS, NOS2, NOS3]
GO:0045862	positive regulation of proteolysis	GO_BP	2.53E-12	7.78E-12	4.0712466	16	[AKT1, APP, AURKA, CASP8, CCNA2, FN1, GSK3B, HMGB1, IL1B, KEAP1, MYC, NFE2L2, PPARG, PTK2, SRC, STAT3]
GO:0036294	cellular response to decreased oxygen levels	GO_BP	2.66E-12	8.13E-12	6.0185184	13	[AKT1, CCNA2, CCNB1, HMOX1, MTOR, MYC, NFE2L2, NOTCH1, PDPK1, PTGS2, SRC, TP53, VEGFA]
GO:2001236	regulation of extrinsic apoptotic signaling pathway	GO_BP	3.00E-12	9.14E-12	7.0588236	12	[AKT1, BCL2L1, CASP8, GSK3B, GSTP1, HMOX1, IGF1, IL1B, IL6, NOS3, RELA, SRC]
KEGG:05321	Inflammatory bowel disease	KEGG	7.68E-14	2.80E-13	15.384615	10	[IL1B, IL6, JUN, NFATC1, NFKB1, RELA, SMAD2, STAT1, STAT3, TLR4]
GO:0010906	regulation of glucose metabolic process	GO_BP	3.24E-12	9.84E-12	8.527132	11	[AKT1, GSK3B, HMGB1, IGF1, INS, IRS1, MTOR, PDPK1, PPARA, SRC, TP53]
GO:0045088	regulation of innate immune response	GO_BP	3.27E-12	9.92E-12	4.491018	15	[CASP8, CHUK, HMGB1, HSP90AA1, INS, KRAS, NFKB1, PDPK1, PPARG, PRKCA, PRKCD, RELA, SRC, STAT1, TLR4]
GO:1901654	response to ketone	GO_BP	3.35E-12	1.01E-11	5.909091	13	[AHR, AKT1, BCL2L1, CCND1, CDK4, EGFR, FOS, FOXO1, MYC, PARP1, PPARG, RELA, SRC]

GO:0070661	leukocyte proliferation	GO_BP	3.72E-12	1.12E-11	4.4510384	15	[AHR, ATM, CASP3, ERBB2, GSTP1, HMGB1, IGF1, IL1B, IL6, MAPK1, MAPK3, PRKCD, PTK2, TLR4, TP53]
GO:0051092	positive regulation of NF-kappaB transcription factor activity	GO_BP	3.95E-12	1.18E-11	6.8965516	12	[APP, CAT, CHUK, IL1B, IL6, INS, KRAS, NFKB1, NFKBIA, RELA, STAT3, TLR4]
GO:0034250	positive regulation of cellular amide metabolic process	GO_BP	3.95E-12	1.18E-11	6.8965516	12	[CASP3, CCNA2, CDK4, ERBB2, IL6, MAPK1, MAPK3, MTOR, MYC, NFE2L2, PRKCD, RELA]
GO:0009636	response to toxic substance	GO_BP	4.00E-12	1.20E-11	5.035971	14	[AHR, CAT, CCNB1, CDH1, CDK1, CDK4, CHUK, FOS, GSTP1, MAPK1, MAPK3, NFE2L2, NOS3, PTGS2]
GO:0000307	cyclin-dependent protein kinase holoenzyme complex	GO_CC	5.33E-12	1.58E-11	18.60465	8	[CCNA2, CCNB1, CCND1, CDK1, CDK2, CDK4, CDK6, RB1]
GO:0090068	positive regulation of cell cycle process	GO_BP	5.89E-12	1.75E-11	5.652174	13	[AKT1, APP, AURKA, CCNB1, CCND1, CDK1, CDK4, EGFR, EZH2, IGF1, IL1B, INS, RB1]
KEGG:05230	Central carbon metabolism in cancer	KEGG	1.68E-13	5.98E-13	14.285714	10	[AKT1, EGFR, ERBB2, KRAS, MAP2K1, MAPK1, MAPK3, MTOR, MYC, TP53]
GO:0046824	positive regulation of nucleocytoplasmic transport	GO_BP	5.96E-12	1.76E-11	13.235294	9	[CDH1, CDK1, IL1B, MAPK1, MAPK14, PRKCA, PRKCD, PTGS2, TP53]
GO:1901216	positive regulation of neuron death	GO_BP	6.00E-12	1.77E-11	10.10101	10	[CASP3, CASP8, CDK5, FOS, GSK3B, JUN, MTOR, PARP1, TLR4, TP53]
KEGG:05120	Epithelial cell signaling in Helicobacter pylori infection	KEGG	1.68E-13	5.98E-13	14.285714	10	[CASP3, CHUK, EGFR, JUN, MAPK14, MAPK8, NFKB1, NFKBIA, RELA, SRC]
GO:0031668	cellular response to extracellular stimulus	GO_BP	6.44E-12	1.88E-11	4.861111	14	[FOS, FOXO1, GSTP1, HMOX1, HSPA8, JUN, MAPK1, MAPK3, MAPK8, MTOR, NFE2L2, PPARA, PPARG, TP53]
GO:0014066	regulation of phosphatidylinositol 3-kinase signaling	GO_BP	6.80E-12	1.98E-11	7.9710145	11	[CAT, EGFR, FN1, IGF1, IGF1R, IL6, INS, MAPK1, MAPK3, PTK2, SRC]
KEGG:05146	Amoebiasis	KEGG	8.13E-12	2.36E-11	9.803922	10	[CASP3, FN1, IL1B, IL6, NFKB1, NOS2, PRKCA, PTK2, RELA, TLR4]

GO:0034774	secretory granule lumen	GO_CC	9.92E-12	2.86E-11	4.1551247	15	[APP, CAT, FN1, GSTP1, HMGB1, HSP90AA1, HSPA8, IGF1, IL6, INS, MAPK1, MAPK14, NFKB1, PRKCD, VEGFA]
KEGG:04064	NF-kappa B signaling pathway	KEGG	9.90E-12	2.87E-11	9.615385	10	[ATM, BCL2L1, CHUK, IL1B, NFKB1, NFKBIA, PARP1, PTGS2, RELA, TLR4]
GO:0035270	endocrine system development	GO_BP	1.01E-11	2.89E-11	7.6923075	11	[AKT1, CDH1, CDK6, FOXO1, GSK3B, IL6, MAP2K1, MAPK1, MAPK3, PDPK1, SMAD2]
GO:0097193	intrinsic apoptotic signaling pathway	GO_BP	1.02E-11	2.92E-11	4.6979866	14	[AKT1, ATM, BCL2L1, CASP3, HMOX1, INS, MMP9, NFE2L2, PARP1, PDPK1, PRKCD, PTGS2, SRC, TP53]
GO:0048872	homeostasis of number of cells	GO_BP	1.07E-11	3.04E-11	4.6822743	14	[CASP3, CDK6, EZH2, HMGB1, HMOX1, IL6, KRAS, MAPK14, NOS3, NOTCH1, RB1, STAT1, STAT3, VEGFA]
GO:0051897	positive regulation of protein kinase B signaling	GO_BP	1.12E-11	3.17E-11	6.3157897	12	[EGFR, ERBB2, ESR1, HSP90AA1, IGF1R, IL6, INS, IRS1, MTOR, PDPK1, PTK2, SRC]
GO:0060205	cytoplasmic vesicle lumen	GO_CC	1.16E-11	3.29E-11	4.109589	15	[APP, CAT, FN1, GSTP1, HMGB1, HSP90AA1, HSPA8, IGF1, IL6, INS, MAPK1, MAPK14, NFKB1, PRKCD, VEGFA]
GO:1900180	regulation of protein localization to nucleus	GO_BP	1.17E-11	3.31E-11	7.586207	11	[AKT1, CDH1, CDK1, GSK3B, INS, MAPK1, MAPK14, PARP1, PRKCD, PTGS2, SRC]
GO:0032388	positive regulation of intracellular transport	GO_BP	1.18E-11	3.32E-11	5.3497944	13	[CASP8, CDH1, CDK1, CDK5, ERBB2, IL1B, MAPK1, MAPK14, MAPK8, PRKCA, PRKCD, PTGS2, TP53]
GO:0006006	glucose metabolic process	GO_BP	1.24E-11	3.48E-11	5.327869	13	[AKT1, GSK3B, HMGB1, IGF1, INS, IRS1, MAPK14, MTOR, MYC, PDPK1, PPARA, SRC, TP53]
GO:0042692	muscle cell differentiation	GO_BP	1.30E-11	3.64E-11	4.076087	15	[AKT1, CASP3, CCNB1, CDK1, CHUK, EZH2, IGF1, KRAS, MAPK14, MTOR, NFATC1, NOTCH1, PPARA, RB1, VEGFA]
GO:0008286	insulin receptor signaling pathway	GO_BP	1.36E-11	3.79E-11	7.482993	11	[AKT1, CDK4, FOXO1, GSK3B, IGF1R, IL1B, INS, IRS1, PRKCD, RELA, SRC]
GO:0001936	regulation of endothelial cell proliferation	GO_BP	1.36E-11	3.79E-11	7.482993	11	[AKT1, CCL2, HMGB1, HMOX1, MTOR, PDPK1, PPARG, PRKCA, STAT1, STAT3, VEGFA]

GO:0032881	regulation of polysaccharide metabolic process	GO_BP	1.36E-11	3.79E-11	16.666666	8	[AKT1, GSK3B, HMGB1, IGF1, INS, IRS1, MTOR, NFKB1]
GO:0071356	cellular response to tumor necrosis factor	GO_BP	1.52E-11	4.20E-11	4.560261	14	[AKT1, CASP8, CCL2, CHUK, GSTP1, MAPK1, MAPK14, MAPK3, NFE2L2, NFKB1, NFKBIA, RELA, STAT1, TP53]
GO:0038127	ERBB signaling pathway	GO_BP	1.70E-11	4.68E-11	7.3333335	11	[AKT1, APP, EGFR, ERBB2, HSP90AA1, MAPK1, MMP9, PDPK1, PRKCA, PTK2, SRC]
GO:0043393	regulation of protein binding	GO_BP	1.92E-11	5.29E-11	6.030151	12	[AKT1, APP, AURKA, CDK5, GSK3B, MAPK3, MAPK8, MMP9, PPARA, PRKCA, PRKCD, SRC]
GO:1990776	response to angiotensin	GO_BP	2.16E-11	5.92E-11	23.333334	7	[MYC, NFE2L2, NFKB1, PRKCD, PTGS2, RELA, SRC]
GO:0046622	positive regulation of organ growth	GO_BP	2.28E-11	6.23E-11	15.686275	8	[AKT1, CCNB1, CDK1, IGF1, MAPK1, MAPK14, MTOR, NOTCH1]
KEGG:04217	Necroptosis	KEGG	6.80E-10	1.55E-09	6.289308	10	[CASP8, H2AX, HMGB1, HSP90AA1, IL1B, MAPK8, PARP1, STAT1, STAT3, TLR4]
GO:0046822	regulation of nucleocytoplasmic transport	GO_BP	2.50E-11	6.80E-11	8.77193	10	[CDH1, CDK1, CDK5, IL1B, MAPK1, MAPK14, PRKCA, PRKCD, PTGS2, TP53]
GO:0007569	cell aging	GO_BP	2.50E-11	6.80E-11	8.77193	10	[ATM, CDK1, CDK6, H2AX, KRAS, MAP2K1, MAPK14, MTOR, PRKCD, TP53]
GO:1900544	positive regulation of purine nucleotide metabolic process	GO_BP	2.69E-11	7.27E-11	15.384615	8	[APP, IGF1, INS, MYC, NOS2, NOS3, PPARA, STAT3]
GO:0045981	positive regulation of nucleotide metabolic process	GO_BP	2.69E-11	7.27E-11	15.384615	8	[APP, IGF1, INS, MYC, NOS2, NOS3, PPARA, STAT3]
GO:0070663	regulation of leukocyte proliferation	GO_BP	2.75E-11	7.42E-11	5	13	[AHR, ATM, CASP3, ERBB2, GSTP1, HMGB1, IGF1, IL1B, IL6, MAPK1, MAPK3, PTK2, TLR4]
GO:0002218	activation of innate immune response	GO_BP	2.99E-11	8.01E-11	6.962025	11	[CHUK, HMGB1, HSP90AA1, KRAS, NFKB1, PDPK1, PRKCA, PRKCD, RELA, SRC, TLR4]

GO:0016709	oxidoreductase activity, acting on paired donors, with incorporation or reduction of molecular oxygen, NAD(P)H as one donor, and incorporation of one atom of oxygen	GO_MF	2.98E-11	8.02E-11	8.620689	10	[AKT1, EGFR, ESR1, HSP90AA1, IL1B, INS, KRAS, NFKB1, NOS2, NOS3]
GO:0048146	positive regulation of fibroblast proliferation	GO_BP	3.15E-11	8.43E-11	15.094339	8	[CCNA2, CCNB1, CDK4, CDK6, ESR1, FN1, IGF1, MYC]
GO:0061695	transferase complex, transferring phosphorus-containing groups	GO_CC	3.18E-11	8.46E-11	4.942966	13	[CCNA2, CCNB1, CCND1, CDK1, CDK2, CDK4, CDK5, CDK6, CHUK, IGF1R, IRS1, RB1, TP53]
GO:0010595	positive regulation of endothelial cell migration	GO_BP	3.54E-11	9.40E-11	8.474576	10	[AKT1, HMGB1, HMOX1, MAPK14, NFE2L2, NOS3, PDPK1, PRKCA, PTGS2, VEGFA]
GO:2000377	regulation of reactive oxygen species metabolic process	GO_BP	3.66E-11	9.70E-11	6.8322983	11	[AKT1, EGFR, GSTP1, INS, MAPK14, NFE2L2, PPARA, PRKCD, STAT3, TLR4, TP53]
GO:0007565	female pregnancy	GO_BP	3.81E-11	1.01E-10	5.687204	12	[AKT1, ESR1, FOS, IL1B, KRAS, MAPK1, MAPK3, MMP2, MMP9, MTOR, PTGS2, VEGFA]
GO:0045927	positive regulation of growth	GO_BP	4.02E-11	1.06E-10	4.850746	13	[AKT1, CCNB1, CDK1, EGFR, ERBB2, FN1, IGF1, INS, MAPK1, MAPK14, MTOR, NOTCH1, VEGFA]
GO:0033157	regulation of intracellular protein transport	GO_BP	4.02E-11	1.06E-10	4.850746	13	[CASP8, CDH1, CDK1, CDK5, ERBB2, IL1B, MAPK1, MAPK14, MAPK8, PRKCA, PRKCD, PTGS2, TP53]
GO:0001935	endothelial cell proliferation	GO_BP	4.19E-11	1.10E-10	6.7484665	11	[AKT1, CCL2, HMGB1, HMOX1, MTOR, PDPK1, PPARG, PRKCA, STAT1, STAT3, VEGFA]
GO:0070849	response to epidermal growth factor	GO_BP	4.31E-11	1.13E-10	14.545455	8	[AKT1, EGFR, ERBB2, GSTP1, MAPK1, MAPK3, MYC, PDPK1]
GO:0051972	regulation of telomerase activity	GO_BP	4.31E-11	1.13E-10	14.545455	8	[ATM, HSP90AA1, MAPK1, MAPK3, MYC, PPARG, SRC, TP53]
GO:1901988	negative regulation of cell cycle phase transition	GO_BP	4.48E-11	1.17E-10	4.204204	14	[ATM, AURKA, CCL2, CCNB1, CCND1, CDK1, CDK2, CDK4, CDK6, EZH2, H2AX, MAPK14, RB1, TP53]

KEGG:05134	Legionellosis	KEGG	1.13E-12	3.68E-12	15.789474	9	[CASP3, CASP8, HSPA8, IL1B, IL6, NFKB1, NFKBIA, RELA, TLR4]
GO:0051146	striated muscle cell differentiation	GO_BP	5.54E-11	1.43E-10	4.7272725	13	[AKT1, CASP3, CCNB1, CDK1, CHUK, EZH2, IGF1, KRAS, MAPK14, MTOR, NOTCH1, PPARA, RB1]
GO:0070555	response to interleukin-1	GO_BP	5.58E-11	1.44E-10	5.504587	12	[APP, CCL2, CHUK, IL1B, IL6, MAPK3, MYC, NFKB1, NFKBIA, PRKCA, RELA, SRC]
GO:0055023	positive regulation of cardiac muscle tissue growth	GO_BP	5.63E-11	1.45E-10	20.588236	7	[CCNB1, CDK1, IGF1, MAPK1, MAPK14, MTOR, NOTCH1]
GO:0051101	regulation of DNA binding	GO_BP	6.30E-11	1.62E-10	8	10	[HMGB1, HMOX1, IGF1, JUN, MAPK8, MMP9, MYC, NFKBIA, PARP1, PPARG]
GO:0071260	cellular response to mechanical stimulus	GO_BP	6.49E-11	1.66E-10	10.227273	9	[AKT1, CASP8, EGFR, IL1B, MAPK3, MAPK8, NFKB1, PTGS2, TLR4]
GO:0003720	telomerase activity	GO_BP	7.74E-11	1.98E-10	13.559322	8	[ATM, HSP90AA1, MAPK1, MAPK3, MYC, PPARG, SRC, TP53]
GO:0017038	protein import	GO_BP	8.05E-11	2.05E-10	5.3333335	12	[AKT1, CDH1, CDK1, HSP90AA1, HSPA8, MAPK1, MAPK14, NFKBIA, PRKCD, PTGS2, STAT3, TP53]
GO:0019318	hexose metabolic process	GO_BP	8.63E-11	2.19E-10	4.5614033	13	[AKT1, GSK3B, HMGB1, IGF1, INS, IRS1, MAPK14, MTOR, MYC, PDPK1, PPARA, SRC, TP53]
GO:0098531	ligand-activated transcription factor activity	GO_BP	1.02E-10	2.58E-10	13.114754	8	[AHR, ESR1, ESR2, NR1I2, PPARA, PPARG, RXRA, STAT3]
GO:0004879	nuclear receptor activity	GO_BP	1.02E-10	2.58E-10	13.114754	8	[AHR, ESR1, ESR2, NR1I2, PPARA, PPARG, RXRA, STAT3]
GO:0030168	platelet activation	GO_BP	1.02E-10	2.58E-10	6.2146893	11	[FN1, IL6, MAPK1, MAPK14, MAPK3, NOS3, PDPK1, PRKCA, PRKCD, SRC, TLR4]
GO:0034605	cellular response to heat	GO_BP	1.08E-10	2.72E-10	7.5757575	10	[ATM, GSK3B, HMOX1, HSP90AA1, HSPA8, MAPK1, MAPK3, MTOR, PRKCA, PTGS2]
GO:0008637	apoptotic mitochondrial changes	GO_BP	1.08E-10	2.72E-10	7.5757575	10	[AKT1, BCL2L1, CASP8, GSK3B, IGF1, IL6, JUN, MAPK8, MMP9, TP53]

GO:1900542	regulation of purine nucleotide metabolic process	GO_BP	1.17E-10	2.93E-10	7.518797	10	[APP, IGF1, INS, MYC, NOS2, NOS3, PARP1, PDPK1, PPARA, STAT3]
GO:0060421	positive regulation of heart growth	GO_BP	1.30E-10	3.26E-10	18.421053	7	[CCNB1, CDK1, IGF1, MAPK1, MAPK14, MTOR, NOTCH1]
GO:0055021	regulation of cardiac muscle tissue growth	GO_BP	1.34E-10	3.33E-10	12.698413	8	[CCNB1, CDK1, IGF1, MAPK1, MAPK14, MTOR, NOTCH1, PPARA]
GO:0006140	regulation of nucleotide metabolic process	GO_BP	1.36E-10	3.37E-10	7.4074073	10	[APP, IGF1, INS, MYC, NOS2, NOS3, PARP1, PDPK1, PPARA, STAT3]
GO:0045765	regulation of angiogenesis	GO_BP	1.38E-10	3.41E-10	4.391892	13	[ERBB2, HMGB1, HMOX1, IL1B, IL6, NFE2L2, NOS3, PDPK1, PPARG, PRKCA, STAT1, STAT3, VEGFA]
GO:0035265	organ growth	GO_BP	1.38E-10	3.41E-10	6.0439563	11	[AKT1, CCNB1, CDK1, ESR1, IGF1, MAPK1, MAPK14, MTOR, NOTCH1, PPARA, SMAD2]
GO:1901342	regulation of vasculature development	GO_BP	1.63E-10	4.01E-10	4.3333335	13	[ERBB2, HMGB1, HMOX1, IL1B, IL6, NFE2L2, NOS3, PDPK1, PPARG, PRKCA, STAT1, STAT3, VEGFA]
GO:0070873	regulation of glycogen metabolic process	GO_BP	1.91E-10	4.70E-10	17.5	7	[AKT1, GSK3B, HMGB1, IGF1, INS, IRS1, MTOR]
GO:0050994	regulation of lipid catabolic process	GO_BP	1.96E-10	4.79E-10	12.121212	8	[AKT1, CDK4, IL1B, INS, IRS1, MTOR, PPARA, PRKCD]
GO:0097153	cysteine-type endopeptidase activity involved in apoptotic process	GO_BP	1.96E-10	4.80E-10	4.9382715	12	[AKT1, CASP3, CASP8, CCNA2, HMGB1, IL6, MMP9, MYC, PPARG, PTGS2, SRC, VEGFA]
GO:0016705	oxidoreductase activity, acting on paired donors, with incorporation or reduction of molecular oxygen	GO_MF	2.15E-10	5.24E-10	4.897959	12	[AKT1, EGFR, ESR1, HMOX1, HSP90AA1, IL1B, INS, KRAS, NFKB1, NOS2, NOS3, PTGS2]
GO:0014015	positive regulation of gliogenesis	GO_BP	2.22E-10	5.39E-10	11.940298	8	[EGFR, IL1B, IL6, MTOR, MYC, NOTCH1, PPARG, RELA]
GO:0046620	regulation of organ growth	GO_BP	2.28E-10	5.51E-10	8.910892	9	[AKT1, CCNB1, CDK1, IGF1, MAPK1, MAPK14, MTOR, NOTCH1, PPARA]

GO:0032885	regulation of polysaccharide biosynthetic process	GO_BP	2.30E-10	5.55E-10	17.073172	7	[AKT1, GSK3B, IGF1, INS, IRS1, MTOR, NFKB1]
GO:0010907	positive regulation of glucose metabolic process	GO_BP	2.30E-10	5.55E-10	17.073172	7	[AKT1, HMGB1, IGF1, INS, IRS1, PPARA, SRC]
GO:0003964	RNA-directed DNA polymerase activity	GO_BP	2.51E-10	6.04E-10	11.764706	8	[ATM, HSP90AA1, MAPK1, MAPK3, MYC, PPARG, SRC, TP53]
GO:0005996	monosaccharide metabolic process	GO_BP	2.64E-10	6.33E-10	4.1666665	13	[AKT1, GSK3B, HMGB1, IGF1, INS, IRS1, MAPK14, MTOR, MYC, PDPK1, PPARA, SRC, TP53]
GO:0014013	regulation of gliogenesis	GO_BP	2.72E-10	6.50E-10	8.7378645	9	[EGFR, EZH2, IL1B, IL6, MTOR, MYC, NOTCH1, PPARG, RELA]
GO:0035051	cardiocyte differentiation	GO_BP	2.75E-10	6.56E-10	6.8965516	10	[CCNB1, CDK1, EGFR, IGF1, MAPK1, MAPK3, MTOR, NOTCH1, PPARA, VEGFA]
GO:0032768	regulation of monooxygenase activity	GO_BP	2.83E-10	6.73E-10	11.594203	8	[AKT1, EGFR, HSP90AA1, IL1B, INS, KRAS, NFKB1, NOS3]
GO:0045639	positive regulation of myeloid cell differentiation	GO_BP	2.97E-10	7.03E-10	8.653846	9	[CASP8, FOS, HMGB1, JUN, MAPK14, PRKCA, RB1, STAT1, STAT3]
GO:0019217	regulation of fatty acid metabolic process	GO_BP	2.97E-10	7.03E-10	8.653846	9	[AKT1, IL1B, INS, IRS1, MTOR, PDPK1, PPARA, PPARG, PTGS2]
GO:0005158	insulin receptor binding	GO_MF	2.99E-10	7.07E-10	26.086956	6	[IGF1, IGF1R, INS, IRS1, PDPK1, SRC]
GO:0045930	negative regulation of mitotic cell cycle	GO_BP	3.09E-10	7.28E-10	4.113924	13	[ATM, AURKA, CCL2, CCNB1, CCND1, CDK1, CDK2, CDK4, CDK6, EGFR, EZH2, RB1, TP53]
GO:0060420	regulation of heart growth	GO_BP	3.18E-10	7.49E-10	11.428572	8	[CCNB1, CDK1, IGF1, MAPK1, MAPK14, MTOR, NOTCH1, PPARA]
GO:1901989	positive regulation of cell cycle phase transition	GO_BP	3.23E-10	7.58E-10	8.571428	9	[AKT1, APP, CCNB1, CCND1, CDK1, CDK4, EGFR, EZH2, RB1]
GO:1904646	cellular response to amyloid-beta	GO_BP	3.28E-10	7.67E-10	16.27907	7	[APP, CDK5, GSK3B, IGF1, IGF1R, PARP1, TLR4]
GO:0001101	response to acid chemical	GO_BP	3.36E-10	7.85E-10	6.756757	10	[BCL2L1, CASP3, CHUK, GSTP1, INS, MMP2, MTOR, MYC, RELA, VEGFA]

GO:0051402	neuron apoptotic process	GO_BP	3.73E-10	8.68E-10	4.6692605	12	[APP, BCL2L1, CASP3, CCL2, CDK5, HMOX1, JUN, KRAS, PARP1, PDPK1, RB1, TP53]
GO:0031669	cellular response to nutrient levels	GO_BP	3.90E-10	9.05E-10	4.6511626	12	[FOXO1, HMOX1, HSPA8, JUN, MAPK1, MAPK3, MAPK8, MTOR, NFE2L2, PPARA, PPARG, TP53]
GO:1901991	negative regulation of mitotic cell cycle phase transition	GO_BP	4.26E-10	9.86E-10	4.6153846	12	[ATM, AURKA, CCL2, CCNB1, CCND1, CDK1, CDK2, CDK4, CDK6, EZH2, RB1, TP53]
GO:1903798	regulation of production of miRNAs involved in gene silencing by miRNA	GO_BP	5.21E-10	1.20E-09	24	6	[EGFR, ESR1, IL6, MAP2K1, STAT3, TP53]
GO:0070920	regulation of production of small RNA involved in gene silencing by RNA	GO_BP	5.21E-10	1.20E-09	24	6	[EGFR, ESR1, IL6, MAP2K1, STAT3, TP53]
GO:0035994	response to muscle stretch	GO_BP	5.21E-10	1.20E-09	24	6	[FOS, MAPK14, NFKB1, NFKBIA, PTK2, RELA]
GO:0045931	positive regulation of mitotic cell cycle	GO_BP	5.33E-10	1.23E-09	8.1081085	9	[AKT1, APP, CCNB1, CCND1, CDK1, CDK4, EGFR, PRKCA, RB1]
KEGG:04664	Fc epsilon RI signaling pathway	KEGG	5.96E-12	1.76E-11	13.235294	9	[AKT1, KRAS, MAP2K1, MAPK1, MAPK14, MAPK3, MAPK8, PDPK1, PRKCA]
GO:0001890	placenta development	GO_BP	6.01E-10	1.37E-09	6.3694267	10	[AKT1, CASP8, CCNA2, MAP2K1, MAPK1, MAPK14, MAPK3, PPARG, PTGS2, PTK2]
GO:0006278	RNA-dependent DNA biosynthetic process	GO_BP	6.26E-10	1.43E-09	7.964602	9	[ATM, CDK2, HSP90AA1, MAPK1, MAPK3, MYC, PPARG, SRC, TP53]
KEGG:04072	Phospholipase D signaling pathway	KEGG	6.88E-09	1.37E-08	6.081081	9	[AKT1, EGFR, INS, KRAS, MAP2K1, MAPK1, MAPK3, MTOR, PRKCA]
GO:0019903	protein phosphatase binding	GO_MF	6.80E-10	1.55E-09	6.289308	10	[AKT1, EGFR, ERBB2, FOXO1, MAPK14, PPARG, PTK2, STAT1, STAT3, TP53]
GO:0006606	protein import into nucleus	GO_BP	7.69E-10	1.74E-09	6.21118	10	[AKT1, CDH1, CDK1, MAPK1, MAPK14, NFKBIA, PRKCD, PTGS2, STAT3, TP53]
GO:1904019	epithelial cell apoptotic process	GO_BP	7.92E-10	1.79E-09	7.7586207	9	[BCL2L1, CCL2, HMOX1, IL6, MTOR, NFE2L2, PDPK1, PPARA, RB1]

GO:1900371	regulation of purine nucleotide biosynthetic process	GO_BP	8.58E-10	1.94E-09	14.285714	7	[MYC, NOS2, NOS3, PARP1, PDPK1, PPARA, STAT3]
GO:1904385	cellular response to angiotensin	GO_BP	8.66E-10	1.95E-09	22.222221	6	[MYC, NFE2L2, NFKB1, PRKCD, RELA, SRC]
GO:0006809	nitric oxide biosynthetic process	GO_BP	9.47E-10	2.13E-09	10	8	[AKT1, HSP90AA1, IL1B, MTOR, NOS2, NOS3, PTGS2, TLR4]
GO:0010821	regulation of mitochondrion organization	GO_BP	9.77E-10	2.19E-09	6.060606	10	[AKT1, AURKA, BCL2L1, CASP8, GSK3B, IGF1, IL6, MAPK8, MMP9, TP53]
GO:0030808	regulation of nucleotide biosynthetic process	GO_BP	9.94E-10	2.22E-09	14	7	[MYC, NOS2, NOS3, PARP1, PDPK1, PPARA, STAT3]
GO:0030225	macrophage differentiation	GO_BP	9.94E-10	2.22E-09	14	7	[APP, CASP8, MMP9, PARP1, PRKCA, RB1, VEGFA]
GO:0051100	negative regulation of binding	GO_BP	1.10E-09	2.45E-09	5.9880238	10	[AKT1, AURKA, GSK3B, HMOX1, JUN, MAPK3, MAPK8, NFKBIA, PPARA, PRKCD]
GO:0071478	cellular response to radiation	GO_BP	1.14E-09	2.54E-09	4.954955	11	[ATM, BCL2L1, H2AX, MAPK14, MMP2, MMP9, MYC, PARP1, PRKCD, PTGS2, TP53]
GO:1901992	positive regulation of mitotic cell cycle phase transition	GO_BP	1.16E-09	2.56E-09	9.756098	8	[AKT1, APP, CCNB1, CCND1, CDK1, CDK4, EGFR, RB1]
GO:0010827	regulation of glucose transmembrane transport	GO_BP	1.16E-09	2.56E-09	9.756098	8	[AKT1, IGF1, IL1B, INS, IRS1, MAPK14, MYC, NFE2L2]
GO:0021782	glial cell development	GO_BP	1.16E-09	2.56E-09	7.4380164	9	[AKT1, APP, CDK5, CDK6, EGFR, GSTP1, IL6, KRAS, TLR4]
GO:0002761	regulation of myeloid leukocyte differentiation	GO_BP	1.16E-09	2.56E-09	7.4380164	9	[CASP8, CDK6, FOS, JUN, MTOR, MYC, PRKCA, RB1, TLR4]
GO:0016458	gene silencing	GO_BP	1.21E-09	2.68E-09	4.2105265	12	[CDK2, EGFR, ESR1, EZH2, H2AX, HMGB1, IL6, MAP2K1, PPARG, SMAD2, STAT3, TP53]
GO:1990858	cellular response to lectin	GO_BP	1.24E-09	2.73E-09	7.377049	9	[CHUK, KRAS, MYC, NFKB1, PDPK1, PRKCA, PRKCD, RELA, SRC]
GO:1990840	response to lectin	GO_BP	1.24E-	2.73E-09	7.377049	9	[CHUK, KRAS, MYC, NFKB1, PDPK1, PRKCA,

			09				PRKCD, RELA, SRC]
GO:0002262	myeloid cell homeostasis	GO_BP	1.31E-09	2.87E-09	5.882353	10	[CASP3, CDK6, HMGB1, HMOX1, IL6, MAPK14, RB1, STAT1, STAT3, VEGFA]
GO:0035196	production of miRNAs involved in gene silencing by miRNA	GO_BP	1.52E-09	3.32E-09	13.207547	7	[EGFR, ESR1, IL6, MAP2K1, SMAD2, STAT3, TP53]
GO:0007249	I-kappaB kinase/NF-kappaB signaling	GO_BP	1.54E-09	3.35E-09	4.123711	12	[AKT1, CASP8, CHUK, ESR1, GSTP1, HMOX1, IL1B, NFKBIA, PDPK1, RELA, STAT1, TLR4]
GO:2000278	regulation of DNA biosynthetic process	GO_BP	1.54E-09	3.36E-09	7.2	9	[ATM, HSP90AA1, IL6, MAPK1, MAPK3, MYC, PPARG, SRC, TP53]
GO:1904018	positive regulation of vasculature development	GO_BP	1.64E-09	3.56E-09	5.7471266	10	[HMGB1, HMOX1, IL1B, IL6, NFE2L2, NOS3, PDPK1, PRKCA, STAT3, VEGFA]
GO:0045766	positive regulation of angiogenesis	GO_BP	1.64E-09	3.56E-09	5.7471266	10	[HMGB1, HMOX1, IL1B, IL6, NFE2L2, NOS3, PDPK1, PRKCA, STAT3, VEGFA]
GO:0060485	mesenchyme development	GO_BP	1.66E-09	3.60E-09	4.095563	12	[EZH2, GSK3B, IL1B, IL6, MAPK1, MAPK3, MTOR, MYC, NOS3, NOTCH1, SMAD2, STAT1]
GO:0044839	cell cycle G2/M phase transition	GO_BP	1.66E-09	3.60E-09	4.095563	12	[APP, ATM, AURKA, CCNA2, CCNB1, CCND1, CDK1, CDK2, CDK4, HSP90AA1, PRKCA, TP53]
GO:0044853	plasma membrane raft	GO_CC	1.78E-09	3.84E-09	7.086614	9	[CDH1, HMOX1, IRS1, MAPK1, MAPK3, NOS3, PRKCA, PTGS2, SRC]
GO:0005976	polysaccharide metabolic process	GO_BP	1.78E-09	3.84E-09	7.086614	9	[AKT1, GSK3B, HMGB1, IGF1, INS, IRS1, MTOR, MYC, NFKB1]
GO:1902749	regulation of cell cycle G2/M phase transition	GO_BP	1.82E-09	3.92E-09	4.7413793	11	[APP, ATM, AURKA, CCNB1, CCND1, CDK1, CDK2, CDK4, HSP90AA1, PRKCA, TP53]
GO:0051384	response to glucocorticoid	GO_BP	1.83E-09	3.93E-09	5.681818	10	[CASP3, CCND1, EGFR, FOS, FOXO1, GSTP1, HMGB1, IL6, KRAS, PTGS2]
GO:0055017	cardiac muscle tissue growth	GO_BP	1.87E-09	4.00E-09	9.195402	8	[CCNB1, CDK1, IGF1, MAPK1, MAPK14, MTOR, NOTCH1, PPARA]
GO:0046209	nitric oxide metabolic process	GO_BP	1.87E-09	4.00E-09	9.195402	8	[AKT1, HSP90AA1, IL1B, MTOR, NOS2, NOS3, PTGS2, TLR4]
GO:0003300	cardiac muscle hypertrophy	GO_BP	1.87E-	4.00E-09	9.195402	8	[EZH2, FOXO1, IGF1, MTOR, NOTCH1, PARP1,

			09				PPARA, PRKCA]
GO:0001649	osteoblast differentiation	GO_BP	1.90E-09	4.06E-09	4.72103	11	[AKT1, CAT, CCNA2, CDK6, IGF1, IL6, MAPK14, NOTCH1, PPARG, PRKCA, PTK2]
GO:0050999	regulation of nitric-oxide synthase activity	GO_BP	1.99E-09	4.24E-09	12.727273	7	[AKT1, EGFR, HSP90AA1, IL1B, INS, KRAS, NOS3]
KEGG:04630	JAK-STAT signaling pathway	KEGG	1.52E-08	2.86E-08	5.5555553	9	[AKT1, BCL2L1, CCND1, EGFR, IL6, MTOR, MYC, STAT1, STAT3]
GO:2001057	reactive nitrogen species metabolic process	GO_BP	2.05E-09	4.34E-09	9.090909	8	[AKT1, HSP90AA1, IL1B, MTOR, NOS2, NOS3, PTGS2, TLR4]
GO:0048639	positive regulation of developmental growth	GO_BP	2.04E-09	4.35E-09	5.6179776	10	[AKT1, CCNB1, CDK1, FN1, IGF1, MAPK1, MAPK14, MTOR, NOTCH1, VEGFA]
GO:0070918	production of small RNA involved in gene silencing by RNA	GO_BP	2.26E-09	4.79E-09	12.5	7	[EGFR, ESR1, IL6, MAP2K1, SMAD2, STAT3, TP53]
GO:0031050	dsRNA processing	GO_BP	2.26E-09	4.79E-09	12.5	7	[EGFR, ESR1, IL6, MAP2K1, SMAD2, STAT3, TP53]
GO:0051170	import into nucleus	GO_BP	2.28E-09	4.80E-09	5.5555553	10	[AKT1, CDH1, CDK1, MAPK1, MAPK14, NFKBIA, PRKCD, PTGS2, STAT3, TP53]
GO:0004497	monooxygenase activity	GO_MF	2.28E-09	4.80E-09	5.5555553	10	[AKT1, EGFR, ESR1, HSP90AA1, IL1B, INS, KRAS, NFKB1, NOS2, NOS3]
GO:0045089	positive regulation of innate immune response	GO_BP	2.27E-09	4.80E-09	4.6413503	11	[CHUK, HMGB1, HSP90AA1, KRAS, NFKB1, PDPK1, PRKCA, PRKCD, RELA, SRC, TLR4]
GO:0043281	regulation of cysteine-type endopeptidase activity involved in apoptotic process	GO_BP	2.27E-09	4.80E-09	4.6413503	11	[AKT1, CASP8, CCNA2, HMGB1, IL6, MMP9, MYC, PPARG, PTGS2, SRC, VEGFA]
GO:0043502	regulation of muscle adaptation	GO_BP	2.45E-09	5.15E-09	8.888889	8	[FOXO1, IGF1, MTOR, NOS3, NOTCH1, PARP1, PPARA, PRKCA]
GO:0014897	striated muscle hypertrophy	GO_BP	2.45E-09	5.15E-09	8.888889	8	[EZH2, FOXO1, IGF1, MTOR, NOTCH1, PARP1, PPARA, PRKCA]
GO:0050769	positive regulation of	GO_BP	2.48E-	5.20E-09	4.6025105	11	[EGFR, FN1, IL1B, IL6, MAP2K1, MTOR, MYC,

	neurogenesis		09				NOTCH1, PPARG, RELA, VEGFA]
GO:1990874	vascular associated smooth muscle cell proliferation	GO_BP	2.57E-09	5.38E-09	12.280702	7	[GSTP1, HMOX1, IGF1, JUN, MMP2, MMP9, PPARG]
GO:1904705	regulation of vascular associated smooth muscle cell proliferation	GO_BP	2.57E-09	5.38E-09	12.280702	7	[GSTP1, HMOX1, IGF1, JUN, MMP2, MMP9, PPARG]
GO:1902893	regulation of pri-miRNA transcription by RNA polymerase II	GO_BP	2.57E-09	5.38E-09	12.280702	7	[FOS, JUN, PPARA, PPARG, RELA, STAT3, TP53]
GO:0061614	pri-miRNA transcription by RNA polymerase II	GO_BP	2.57E-09	5.38E-09	12.280702	7	[FOS, JUN, PPARA, PPARG, RELA, STAT3, TP53]
GO:0010676	positive regulation of cellular carbohydrate metabolic process	GO_BP	2.57E-09	5.38E-09	12.280702	7	[AKT1, HMGB1, IGF1, INS, IRS1, PPARA, SRC]
GO:0046889	positive regulation of lipid biosynthetic process	GO_BP	2.93E-09	6.10E-09	8.695652	8	[AKT1, CCNA2, IL1B, INS, MTOR, PPARA, PRKCD, PTGS2]
GO:0014896	muscle hypertrophy	GO_BP	2.93E-09	6.10E-09	8.695652	8	[EZH2, FOXO1, IGF1, MTOR, NOTCH1, PARP1, PPARA, PRKCA]
GO:0005901	caveola	GO_CC	2.93E-09	6.10E-09	8.695652	8	[CDH1, HMOX1, IRS1, MAPK1, MAPK3, NOS3, PTGS2, SRC]
GO:0038095	Fc-epsilon receptor signaling pathway	GO_BP	2.97E-09	6.17E-09	5.4054055	10	[CHUK, FOS, JUN, MAPK1, MAPK3, MAPK8, NFATC1, NFKB1, PDPK1, RELA]
GO:0060251	regulation of glial cell proliferation	GO_BP	3.18E-09	6.59E-09	18.181818	6	[EGFR, IL1B, IL6, MTOR, MYC, NOTCH1]
GO:0010962	regulation of glucan biosynthetic process	GO_BP	3.18E-09	6.59E-09	18.181818	6	[AKT1, GSK3B, IGF1, INS, IRS1, MTOR]
GO:0005979	regulation of glycogen biosynthetic process	GO_BP	3.18E-09	6.59E-09	18.181818	6	[AKT1, GSK3B, IGF1, INS, IRS1, MTOR]
GO:0046890	regulation of lipid biosynthetic process	GO_BP	3.30E-09	6.82E-09	5.347594	10	[AKT1, CCNA2, CDK4, IL1B, INS, MTOR, NFKB1, PPARA, PRKCD, PTGS2]

GO:0061419	positive regulation of transcription from RNA polymerase II promoter in response to hypoxia	GO_BP	3.36E-09	6.93E-09	66.666664	4	[NFE2L2, NOTCH1, TP53, VEGFA]
GO:0070141	response to UV-A	GO_BP	3.56E-09	7.34E-09	31.25	5	[AKT1, CCND1, EGFR, MMP2, MMP9]
KEGG:04360	Axon guidance	KEGG	4.17E-08	7.26E-08	4.945055	9	[CDK5, GSK3B, KRAS, MAPK1, MAPK3, PDPK1, PRKCA, PTK2, SRC]
GO:0010611	regulation of cardiac muscle hypertrophy	GO_BP	3.72E-09	7.65E-09	11.666667	7	[FOXO1, IGF1, MTOR, NOTCH1, PARP1, PPARA, PRKCA]
GO:0060419	heart growth	GO_BP	3.79E-09	7.76E-09	8.421053	8	[CCNB1, CDK1, IGF1, MAPK1, MAPK14, MTOR, NOTCH1, PPARA]
GO:0006112	energy reserve metabolic process	GO_BP	3.79E-09	7.76E-09	8.421053	8	[AKT1, GSK3B, HMGB1, IGF1, INS, IRS1, MTOR, MYC]
GO:0042594	response to starvation	GO_BP	3.81E-09	7.80E-09	4.4176707	11	[FOXO1, HSPA8, JUN, MAPK1, MAPK3, MAPK8, MTOR, NFE2L2, PPARA, PPARG, TP53]
GO:0046324	regulation of glucose import	GO_BP	4.19E-09	8.55E-09	11.4754095	7	[AKT1, IGF1, INS, IRS1, MAPK14, MYC, NFE2L2]
GO:0043470	regulation of carbohydrate catabolic process	GO_BP	4.48E-09	9.08E-09	8.247422	8	[APP, HMGB1, IGF1, INS, MYC, PPARA, STAT3, TP53]
GO:0022408	negative regulation of cell-cell adhesion	GO_BP	4.47E-09	9.09E-09	5.1813474	10	[AKT1, CASP3, CDH1, ERBB2, HMGB1, NOTCH1, PPARA, PRKCD, PTK2, VEGFA]
GO:0007219	Notch signaling pathway	GO_BP	4.47E-09	9.09E-09	5.1813474	10	[AKT1, APP, EGFR, MYC, NFKBIA, NOS3, NOTCH1, SRC, STAT1, STAT3]
GO:0036473	cell death in response to oxidative stress	GO_BP	4.86E-09	9.83E-09	8.163265	8	[AKT1, IL6, INS, NFE2L2, PARP1, PDPK1, PRKCD, TLR4]
GO:0009267	cellular response to starvation	GO_BP	4.94E-09	9.97E-09	5.1282053	10	[FOXO1, HSPA8, JUN, MAPK1, MAPK3, MAPK8, MTOR, NFE2L2, PPARA, TP53]
GO:0014743	regulation of muscle hypertrophy	GO_BP	5.28E-09	1.06E-08	11.111111	7	[FOXO1, IGF1, MTOR, NOTCH1, PARP1, PPARA, PRKCA]
GO:0097421	liver regeneration	GO_BP	5.54E-	1.11E-08	16.666666	6	[CCND1, EGFR, EZH2, HMOX1, IL6, MYC]

			09				
KEGG:04810	Regulation of actin cytoskeleton	KEGG	1.95E-07	3.07E-07	4.1284404	9	[EGFR, FN1, INS, KRAS, MAP2K1, MAPK1, MAPK3, PTK2, SRC]
GO:0032651	regulation of interleukin-1 beta production	GO_BP	6.18E-09	1.24E-08	7.920792	8	[APP, CASP8, GSTP1, HMGB1, IGF1, IL6, STAT3, TLR4]
GO:0032611	interleukin-1 beta production	GO_BP	6.18E-09	1.24E-08	7.920792	8	[APP, CASP8, GSTP1, HMGB1, IGF1, IL6, STAT3, TLR4]
GO:0070301	cellular response to hydrogen peroxide	GO_BP	6.68E-09	1.33E-08	7.8431373	8	[CDK1, EZH2, FOXO1, IL6, NFE2L2, PRKCD, RELA, SRC]
GO:0014068	positive regulation of phosphatidylinositol 3-kinase signaling	GO_BP	6.68E-09	1.33E-08	7.8431373	8	[CAT, FN1, IGF1, IGF1R, IL6, INS, PTK2, SRC]
KEGG:04960	Aldosterone-regulated sodium reabsorption	KEGG	1.45E-12	4.65E-12	21.621622	8	[IGF1, INS, IRS1, KRAS, MAPK1, MAPK3, PDPK1, PRKCA]
GO:0060252	positive regulation of glial cell proliferation	GO_BP	6.95E-09	1.38E-08	27.777779	5	[EGFR, IL1B, IL6, MTOR, MYC]
GO:0001938	positive regulation of endothelial cell proliferation	GO_BP	7.22E-09	1.43E-08	7.76699	8	[AKT1, HMGB1, HMOX1, MTOR, PDPK1, PRKCA, STAT3, VEGFA]
GO:2000116	regulation of cysteine-type endopeptidase activity	GO_BP	7.30E-09	1.44E-08	4.1509433	11	[AKT1, CASP8, CCNA2, HMGB1, IL6, MMP9, MYC, PPARG, PTGS2, SRC, VEGFA]
GO:1901655	cellular response to ketone	GO_BP	7.80E-09	1.53E-08	7.6923075	8	[AHR, AKT1, CDK4, EGFR, FOXO1, MYC, PPARG, SRC]
GO:0034061	DNA polymerase activity	GO_BP	7.80E-09	1.53E-08	7.6923075	8	[ATM, HSP90AA1, MAPK1, MAPK3, MYC, PPARG, SRC, TP53]
GO:0097718	disordered domain specific binding	GO_MF	7.80E-09	1.54E-08	15.789474	6	[FN1, HSP90AA1, KEAP1, RB1, SMAD2, TP53]
GO:0007584	response to nutrient	GO_BP	7.98E-09	1.57E-08	4.878049	10	[CAT, CCND1, EGFR, GSTP1, HMOX1, MTOR, PPARG, PTGS2, RELA, STAT1]
GO:0001836	release of cytochrome c from mitochondria	GO_BP	8.20E-09	1.61E-08	10.447762	7	[AKT1, BCL2L1, IGF1, IL6, JUN, MMP9, TP53]
GO:0000187	activation of MAPK activity	GO_BP	8.36E-	1.63E-08	4.854369	10	[EGFR, IGF1, IGF1R, IL1B, IL6, MAP2K1,

			09				MAPK1, MAPK14, MAPK3, TLR4]
GO:0043255	regulation of carbohydrate biosynthetic process	GO_BP	9.08E-09	1.77E-08	7.5471697	8	[AKT1, GSK3B, IGF1, INS, IRS1, MTOR, NFKB1, PPARA]
KEGG:04540	Gap junction	KEGG	2.05E-09	4.34E-09	9.090909	8	[CDK1, EGFR, KRAS, MAP2K1, MAPK1, MAPK3, PRKCA, SRC]
GO:1904589	regulation of protein import	GO_BP	9.12E-09	1.77E-08	10.294118	7	[CDH1, CDK1, HSPA8, MAPK1, MAPK14, PRKCD, PTGS2]
GO:0046326	positive regulation of glucose import	GO_BP	9.19E-09	1.78E-08	15.384615	6	[AKT1, IGF1, INS, IRS1, MAPK14, NFE2L2]
KEGG:04726	Serotonergic synapse	KEGG	1.73E-08	3.22E-08	6.9565215	8	[APP, CASP3, KRAS, MAP2K1, MAPK1, MAPK3, PRKCA, PTGS2]
GO:1905475	regulation of protein localization to membrane	GO_BP	1.00E-08	1.94E-08	4.7619047	10	[AKT1, BCL2L1, CASP8, CDK5, EGFR, ERBB2, INS, MAPK8, PDPK1, TP53]
KEGG:04310	Wnt signaling pathway	KEGG	3.00E-07	4.54E-07	4.8192773	8	[CCND1, GSK3B, JUN, MAPK8, MYC, NFATC1, PRKCA, TP53]
GO:0046626	regulation of insulin receptor signaling pathway	GO_BP	1.01E-08	1.95E-08	10.144928	7	[CDK4, IL1B, INS, IRS1, PRKCD, RELA, SRC]
GO:0031571	mitotic G1 DNA damage checkpoint signaling	GO_BP	1.01E-08	1.95E-08	10.144928	7	[ATM, AURKA, CCNB1, CCND1, CDK1, CDK2, TP53]
GO:0005178	integrin binding	GO_MF	1.03E-08	1.98E-08	5.806452	9	[CCNA2, EGFR, FN1, HMGB1, IGF1, IL1B, PRKCA, PTK2, SRC]
GO:0071354	cellular response to interleukin-6	GO_BP	1.08E-08	2.07E-08	15	6	[IL6, NFKB1, RELA, SRC, STAT1, STAT3]
KEGG:04022	cGMP-PKG signaling pathway	KEGG	3.14E-07	4.73E-07	4.790419	8	[AKT1, INS, IRS1, MAP2K1, MAPK1, MAPK3, NFATC1, NOS3]
GO:0070542	response to fatty acid	GO_BP	1.12E-08	2.14E-08	10	7	[CAT, CCNB1, CDK4, FOXO1, IRS1, PTGS2, SRC]
GO:0044819	mitotic G1/S transition checkpoint signaling	GO_BP	1.12E-08	2.14E-08	10	7	[ATM, AURKA, CCNB1, CCND1, CDK1, CDK2, TP53]
GO:0032757	positive regulation of interleukin-8 production	GO_BP	1.12E-08	2.14E-08	10	7	[HMGB1, IL1B, IL6, NOS2, RELA, STAT3, TLR4]

KEGG:04930	Type II diabetes mellitus	KEGG	5.39E-10	1.24E-09	15.217391	7	[INS, IRS1, MAPK1, MAPK3, MAPK8, MTOR, PRKCD]
GO:0090398	cellular senescence	GO_BP	1.24E-08	2.37E-08	9.859155	7	[CDK6, H2AX, KRAS, MAP2K1, MAPK14, PRKCD, TP53]
GO:0070875	positive regulation of glycogen metabolic process	GO_BP	1.25E-08	2.38E-08	25	5	[AKT1, HMGB1, IGF1, INS, IRS1]
GO:0001221	transcription coregulator binding	GO_MF	1.37E-08	2.60E-08	9.722222	7	[AHR, ESR1, EZH2, FOXO1, NFE2L2, PPARA, RELA]
GO:1902895	positive regulation of pri-miRNA transcription by RNA polymerase II	GO_BP	1.46E-08	2.78E-08	14.285714	6	[FOS, JUN, PPARG, RELA, STAT3, TP53]
GO:0043523	regulation of neuron apoptotic process	GO_BP	1.50E-08	2.84E-08	4.5662103	10	[BCL2L1, CASP3, CCL2, CDK5, HMOX1, JUN, KRAS, PARP1, PDPK1, TP53]
KEGG:04730	Long-term depression	KEGG	3.72E-09	7.65E-09	11.666667	7	[IGF1, IGF1R, KRAS, MAP2K1, MAPK1, MAPK3, PRKCA]
GO:1901215	negative regulation of neuron death	GO_BP	1.57E-08	2.95E-08	4.5454545	10	[AKT1, BCL2L1, CCL2, CDK5, GSK3B, HMOX1, KRAS, PDPK1, PPARA, STAT3]
GO:0070374	positive regulation of ERK1 and ERK2 cascade	GO_BP	1.57E-08	2.95E-08	4.5454545	10	[APP, CCL2, EGFR, HMGB1, MAP2K1, MAPK3, NOTCH1, PRKCA, SRC, TLR4]
GO:0016241	regulation of macroautophagy	GO_BP	1.60E-08	3.01E-08	5.5214725	9	[AKT1, CASP3, CDK5, HMOX1, MAPK3, MAPK8, MTOR, PRKCA, TP53]
GO:0060965	negative regulation of gene silencing by miRNA	GO_BP	1.63E-08	3.07E-08	23.809525	5	[ESR1, IL6, PPARG, STAT3, TP53]
GO:0045429	positive regulation of nitric oxide biosynthetic process	GO_BP	1.70E-08	3.17E-08	13.953488	6	[AKT1, HSP90AA1, IL1B, MTOR, PTGS2, TLR4]
GO:0010389	regulation of G2/M transition of mitotic cell cycle	GO_BP	1.71E-08	3.19E-08	4.5045047	10	[APP, ATM, AURKA, CCNB1, CCND1, CDK1, CDK2, CDK4, HSP90AA1, PRKCA]
KEGG:04929	GnRH secretion	KEGG	5.92E-09	1.19E-08	10.9375	7	[AKT1, ESR2, KRAS, MAP2K1, MAPK1, MAPK3, PRKCA]
GO:1904659	glucose transmembrane	GO_BP	1.73E-	3.22E-08	6.9565215	8	[AKT1, IGF1, IL1B, INS, IRS1, MAPK14, MYC,

	transport		08				NFE2L2]
GO:0010212	response to ionizing radiation	GO_BP	1.78E-08	3.31E-08	5.4545455	9	[ATM, BCL2L1, CASP3, CCND1, H2AX, MAPK14, MYC, PARP1, TP53]
GO:0000077	DNA damage checkpoint signaling	GO_BP	1.78E-08	3.31E-08	5.4545455	9	[ATM, AURKA, CCNB1, CCND1, CDK1, CDK2, H2AX, MAPK14, TP53]
GO:2000573	positive regulation of DNA biosynthetic process	GO_BP	1.83E-08	3.39E-08	9.333333	7	[ATM, HSP90AA1, IL6, MAPK1, MAPK3, MYC, SRC]
GO:0044264	cellular polysaccharide metabolic process	GO_BP	1.85E-08	3.43E-08	6.8965516	8	[AKT1, GSK3B, HMGB1, IGF1, INS, IRS1, MTOR, MYC]
GO:0002685	regulation of leukocyte migration	GO_BP	1.94E-08	3.58E-08	4.4444447	10	[AKT1, APP, CCL2, HMGB1, HMOX1, IL6, MAPK1, MAPK3, PTK2, VEGFA]
GO:1904407	positive regulation of nitric oxide metabolic process	GO_BP	1.96E-08	3.61E-08	13.636364	6	[AKT1, HSP90AA1, IL1B, MTOR, PTGS2, TLR4]
GO:1903580	positive regulation of ATP metabolic process	GO_BP	1.96E-08	3.61E-08	13.636364	6	[APP, IGF1, INS, MYC, PPARA, STAT3]
GO:0070741	response to interleukin-6	GO_BP	1.96E-08	3.61E-08	13.636364	6	[IL6, NFKB1, RELA, SRC, STAT1, STAT3]
GO:0042307	positive regulation of protein import into nucleus	GO_BP	1.96E-08	3.61E-08	13.636364	6	[CDH1, CDK1, MAPK1, MAPK14, PRKCD, PTGS2]
GO:2000379	positive regulation of reactive oxygen species metabolic process	GO_BP	2.00E-08	3.68E-08	9.210526	7	[EGFR, GSTP1, MAPK14, NFE2L2, PRKCD, TLR4, TP53]
GO:1901796	regulation of signal transduction by p53 class mediator	GO_BP	2.02E-08	3.71E-08	4.424779	10	[AKT1, ATM, AURKA, CDK1, CDK2, CDK5, MAPK14, MTOR, PDPK1, TP53]
GO:0060967	negative regulation of gene silencing by RNA	GO_BP	2.11E-08	3.86E-08	22.727272	5	[ESR1, IL6, PPARG, STAT3, TP53]
GO:0060149	negative regulation of posttranscriptional gene silencing	GO_BP	2.11E-08	3.86E-08	22.727272	5	[ESR1, IL6, PPARG, STAT3, TP53]
GO:0051341	regulation of oxidoreductase	GO_BP	2.12E-	3.87E-08	6.779661	8	[AKT1, EGFR, HSP90AA1, IL1B, INS, KRAS,

	activity		08				NFKB1, NOS3]
GO:0046323	glucose import	GO_BP	2.20E-08	4.01E-08	9.090909	7	[AKT1, IGF1, INS, IRS1, MAPK14, MYC, NFE2L2]
GO:0014002	astrocyte development	GO_BP	2.25E-08	4.09E-08	13.333333	6	[APP, CDK6, EGFR, IL6, KRAS, TLR4]
GO:0008645	hexose transmembrane transport	GO_BP	2.27E-08	4.11E-08	6.722689	8	[AKT1, IGF1, IL1B, INS, IRS1, MAPK14, MYC, NFE2L2]
GO:2001242	regulation of intrinsic apoptotic signaling pathway	GO_BP	2.31E-08	4.18E-08	5.2941175	9	[AKT1, BCL2L1, INS, MMP9, NFE2L2, PARP1, PTGS2, SRC, TP53]
GO:0032652	regulation of interleukin-1 production	GO_BP	2.42E-08	4.37E-08	6.6666665	8	[APP, CASP8, GSTP1, HMGB1, IGF1, IL6, STAT3, TLR4]
GO:0032612	interleukin-1 production	GO_BP	2.42E-08	4.37E-08	6.6666665	8	[APP, CASP8, GSTP1, HMGB1, IGF1, IL6, STAT3, TLR4]
GO:0015749	monosaccharide transmembrane transport	GO_BP	2.58E-08	4.65E-08	6.6115704	8	[AKT1, IGF1, IL1B, INS, IRS1, MAPK14, MYC, NFE2L2]
GO:0002223	stimulatory C-type lectin receptor signaling pathway	GO_BP	2.58E-08	4.65E-08	6.6115704	8	[CHUK, KRAS, NFKB1, PDPK1, PRKCA, PRKCD, RELA, SRC]
GO:1904591	positive regulation of protein import	GO_BP	2.58E-08	4.65E-08	13.043478	6	[CDH1, CDK1, MAPK1, MAPK14, PRKCD, PTGS2]
GO:0030857	negative regulation of epithelial cell differentiation	GO_BP	2.58E-08	4.65E-08	13.043478	6	[CCND1, EZH2, MMP9, NOTCH1, STAT1, VEGFA]
GO:0002066	columnar/cuboidal epithelial cell development	GO_BP	2.58E-08	4.65E-08	13.043478	6	[AKT1, CDK6, FOXO1, GSK3B, PDPK1, SRC]
GO:0071897	DNA biosynthetic process	GO_BP	2.59E-08	4.65E-08	4.3103447	10	[ATM, CDK2, HSP90AA1, IL6, MAPK1, MAPK3, MYC, PPARG, SRC, TP53]
GO:1905952	regulation of lipid localization	GO_BP	2.69E-08	4.81E-08	5.202312	9	[AKT1, IL1B, IL6, NFKB1, NFKBIA, PPARA, PPARG, PRKCD, RXRA]
GO:0001889	liver development	GO_BP	2.69E-08	4.81E-08	5.202312	9	[CCND1, EGFR, EZH2, HMOX1, IL6, KRAS, MYC, NOTCH1, RELA]
GO:0030235	nitric-oxide synthase regulator activity	GO_BP	2.79E-08	5.00E-08	44.444443	4	[AKT1, EGFR, ESR1, HSP90AA1]

GO:0048762	mesenchymal cell differentiation	GO_BP	2.81E-08	5.02E-08	4.2735043	10	[EZH2, GSK3B, IL1B, IL6, MAPK1, MAPK3, MTOR, NOTCH1, SMAD2, STAT1]
GO:0031570	DNA integrity checkpoint signaling	GO_BP	2.82E-08	5.03E-08	5.172414	9	[ATM, AURKA, CCNB1, CCND1, CDK1, CDK2, H2AX, MAPK14, TP53]
GO:0010828	positive regulation of glucose transmembrane transport	GO_BP	2.95E-08	5.24E-08	12.765958	6	[AKT1, IGF1, INS, IRS1, MAPK14, NFE2L2]
GO:0061008	hepaticobiliary system development	GO_BP	3.12E-08	5.53E-08	5.1136365	9	[CCND1, EGFR, EZH2, HMOX1, IL6, KRAS, MYC, NOTCH1, RELA]
GO:0006801	superoxide metabolic process	GO_BP	3.14E-08	5.55E-08	8.641975	7	[AKT1, EGFR, GSTP1, NFE2L2, NOS2, NOS3, PRKCD]
GO:0002532	production of molecular mediator involved in inflammatory response	GO_BP	3.14E-08	5.55E-08	8.641975	7	[IL6, INS, MAPK14, NOS2, PPARA, STAT3, TLR4]
GO:0002220	innate immune response activating cell surface receptor signaling pathway	GO_BP	3.33E-08	5.89E-08	6.4	8	[CHUK, KRAS, NFKB1, PDPK1, PRKCA, PRKCD, RELA, SRC]
GO:0002064	epithelial cell development	GO_BP	3.43E-08	6.05E-08	4.1841006	10	[AKT1, CDK6, ESR1, FOXO1, GSK3B, IL1B, NOTCH1, PDPK1, SRC, VEGFA]
GO:1903578	regulation of ATP metabolic process	GO_BP	3.55E-08	6.24E-08	6.3492064	8	[APP, IGF1, INS, MYC, PARP1, PPARA, STAT3, TP53]
GO:0045667	regulation of osteoblast differentiation	GO_BP	3.55E-08	6.24E-08	6.3492064	8	[CCNA2, CDK6, IGF1, IL6, NOTCH1, PPARG, PRKCA, PTK2]
GO:0043200	response to amino acid	GO_BP	3.55E-08	6.24E-08	6.3492064	8	[BCL2L1, CASP3, CHUK, GSTP1, INS, MMP2, MTOR, RELA]
GO:0042752	regulation of circadian rhythm	GO_BP	3.55E-08	6.24E-08	6.3492064	8	[CDK1, EZH2, GSK3B, MAPK8, MTOR, PPARA, PPARG, TP53]
GO:0002758	innate immune response-activating signal transduction	GO_BP	3.55E-08	6.24E-08	6.3492064	8	[CHUK, KRAS, NFKB1, PDPK1, PRKCA, PRKCD, RELA, SRC]
GO:0055006	cardiac cell development	GO_BP	3.72E-08	6.53E-08	8.433735	7	[CCNB1, CDK1, IGF1, MTOR, NOTCH1, PPARA, VEGFA]

GO:0005977	glycogen metabolic process	GO_BP	3.72E-08	6.53E-08	8.433735	7	[AKT1, GSK3B, HMGB1, IGF1, INS, IRS1, MTOR]
GO:0001046	core promoter sequence-specific DNA binding	GO_MF	3.82E-08	6.68E-08	12.244898	6	[EZH2, FOS, MYC, RELA, STAT1, TP53]
GO:0044042	glucan metabolic process	GO_BP	4.05E-08	7.07E-08	8.333333	7	[AKT1, GSK3B, HMGB1, IGF1, INS, IRS1, MTOR]
GO:0006073	cellular glucan metabolic process	GO_BP	4.05E-08	7.07E-08	8.333333	7	[AKT1, GSK3B, HMGB1, IGF1, INS, IRS1, MTOR]
GO:0048588	developmental cell growth	GO_BP	4.17E-08	7.26E-08	4.0983605	10	[APP, AURKA, CDK5, FN1, GSK3B, HSP90AA1, IGF1, MTOR, PPARA, VEGFA]
KEGG:04137	Mitophagy	KEGG	9.12E-09	1.77E-08	10.294118	7	[BCL2L1, JUN, KRAS, MAPK8, RELA, SRC, TP53]
GO:0051099	positive regulation of binding	GO_BP	4.17E-08	7.26E-08	4.945055	9	[APP, CDK5, GSK3B, HMGB1, IGF1, MMP9, MYC, PARP1, PPARG]
GO:1905562	regulation of vascular endothelial cell proliferation	GO_BP	4.22E-08	7.32E-08	20	5	[CCL2, HMGB1, PDPK1, PPARG, STAT3]
GO:0101023	vascular endothelial cell proliferation	GO_BP	4.22E-08	7.32E-08	20	5	[CCL2, HMGB1, PDPK1, PPARG, STAT3]
GO:0060045	positive regulation of cardiac muscle cell proliferation	GO_BP	4.22E-08	7.32E-08	20	5	[CCNB1, CDK1, MAPK1, MAPK14, NOTCH1]
GO:0031281	positive regulation of cyclase activity	GO_BP	4.22E-08	7.32E-08	20	5	[MAPK14, MAPK3, MAPK8, NOS2, NOS3]
GO:1905953	negative regulation of lipid localization	GO_BP	4.32E-08	7.49E-08	12	6	[AKT1, IL6, NFKB1, NFKBIA, PPARA, PPARG]
GO:0010718	positive regulation of epithelial to mesenchymal transition	GO_BP	4.32E-08	7.49E-08	12	6	[EZH2, IL1B, IL6, MTOR, NOTCH1, SMAD2]
GO:0008631	intrinsic apoptotic signaling pathway in response to oxidative stress	GO_BP	4.32E-08	7.49E-08	12	6	[AKT1, INS, NFE2L2, PARP1, PDPK1, PRKCD]

GO:0038061	NIK/NF-kappaB signaling	GO_BP	4.37E-08	7.55E-08	4.9180326	9	[AKT1, APP, CHUK, EGFR, HMGB1, IL1B, NFKBIA, RELA, TLR4]
GO:0031016	pancreas development	GO_BP	4.40E-08	7.59E-08	8.235294	7	[AKT1, CDK6, FOXO1, GSK3B, IL6, PDPK1, SMAD2]
GO:0002526	acute inflammatory response	GO_BP	4.81E-08	8.28E-08	6.10687	8	[FN1, GSTP1, IL1B, IL6, INS, PPARG, PTGS2, STAT3]
GO:0071695	anatomical structure maturation	GO_BP	4.86E-08	8.36E-08	4.032258	10	[APP, AURKA, CCNB1, IGF1, MMP2, MTOR, PPARG, PRKCA, RB1, VEGFA]
GO:0071364	cellular response to epidermal growth factor stimulus	GO_BP	4.88E-08	8.38E-08	11.764706	6	[AKT1, EGFR, ERBB2, GSTP1, MYC, PDPK1]
GO:0002673	regulation of acute inflammatory response	GO_BP	4.88E-08	8.38E-08	11.764706	6	[GSTP1, IL1B, IL6, INS, PPARG, PTGS2]
GO:0045598	regulation of fat cell differentiation	GO_BP	5.11E-08	8.74E-08	6.060606	8	[AKT1, FOXO1, IL6, INS, MAPK14, MTOR, PPARG, PTGS2]
GO:1900034	regulation of cellular response to heat	GO_BP	5.17E-08	8.84E-08	8.045977	7	[ATM, GSK3B, HSP90AA1, HSPA8, MAPK1, MAPK3, MTOR]
GO:0032481	positive regulation of type I interferon production	GO_BP	5.17E-08	8.84E-08	8.045977	7	[CHUK, HMGB1, HSP90AA1, NFKB1, RELA, STAT1, TLR4]
GO:1901522	positive regulation of transcription from RNA polymerase II promoter involved in cellular response to chemical stimulus	GO_BP	5.20E-08	8.87E-08	19.23077	5	[NFE2L2, NOTCH1, RELA, TP53, VEGFA]
GO:0003323	type B pancreatic cell development	GO_BP	5.20E-08	8.87E-08	19.23077	5	[AKT1, CDK6, FOXO1, GSK3B, PDPK1]
GO:0046328	regulation of JNK cascade	GO_BP	5.41E-08	9.21E-08	6.0150375	8	[AKT1, APP, EGFR, GSTP1, HMGB1, IGF1R, IL1B, TLR4]
GO:0034219	carbohydrate transmembrane transport	GO_BP	5.41E-08	9.21E-08	6.0150375	8	[AKT1, IGF1, IL1B, INS, IRS1, MAPK14, MYC, NFE2L2]
GO:0014009	glial cell proliferation	GO_BP	5.50E-08	9.34E-08	11.538462	6	[EGFR, IL1B, IL6, MTOR, MYC, NOTCH1]

GO:0009409	response to cold	GO_BP	5.50E-08	9.34E-08	11.538462	6	[CASP8, FOS, FOXO1, HSP90AA1, NFKBIA, PPARG]
GO:0009250	glucan biosynthetic process	GO_BP	5.50E-08	9.34E-08	11.538462	6	[AKT1, GSK3B, IGF1, INS, IRS1, MTOR]
GO:0005978	glycogen biosynthetic process	GO_BP	5.50E-08	9.34E-08	11.538462	6	[AKT1, GSK3B, IGF1, INS, IRS1, MTOR]
GO:0000271	polysaccharide biosynthetic process	GO_BP	5.60E-08	9.49E-08	7.9545455	7	[AKT1, GSK3B, IGF1, INS, IRS1, MTOR, NFKB1]
GO:0001659	temperature homeostasis	GO_BP	5.77E-08	9.75E-08	4.7619047	9	[FOXO1, IGF1R, IL1B, NOTCH1, PTGS2, RB1, STAT3, TLR4, VEGFA]
GO:0071347	cellular response to interleukin-1	GO_BP	6.03E-08	1.02E-07	4.736842	9	[CCL2, CHUK, IL1B, IL6, MAPK3, MYC, NFKB1, NFKBIA, RELA]
GO:1905477	positive regulation of protein localization to membrane	GO_BP	6.08E-08	1.02E-07	5.9259257	8	[AKT1, CASP8, CDK5, EGFR, ERBB2, MAPK8, PDPK1, TP53]
GO:0035094	response to nicotine	GO_BP	6.19E-08	1.04E-07	11.320755	6	[CASP3, HMOX1, MAPK1, NFKB1, PPARA, RELA]
GO:1900373	positive regulation of purine nucleotide biosynthetic process	GO_BP	6.37E-08	1.07E-07	18.518518	5	[MYC, NOS2, NOS3, PPARA, STAT3]
GO:0030810	positive regulation of nucleotide biosynthetic process	GO_BP	6.37E-08	1.07E-07	18.518518	5	[MYC, NOS2, NOS3, PPARA, STAT3]
GO:0010971	positive regulation of G2/M transition of mitotic cell cycle	GO_BP	6.37E-08	1.07E-07	18.518518	5	[APP, CCNB1, CCND1, CDK1, CDK4]
GO:0030324	lung development	GO_BP	6.60E-08	1.10E-07	4.6875	9	[EGFR, HMGB1, KRAS, MAP2K1, MAPK1, MAPK3, NOTCH1, SMAD2, VEGFA]
GO:0034101	erythrocyte homeostasis	GO_BP	6.82E-08	1.14E-07	5.839416	8	[CASP3, CDK6, HMOX1, MAPK14, RB1, STAT1, STAT3, VEGFA]
GO:0090199	regulation of release of cytochrome c from	GO_BP	6.94E-08	1.16E-07	11.111111	6	[AKT1, BCL2L1, IGF1, IL6, MMP9, TP53]

	mitochondria						
GO:0031018	endocrine pancreas development	GO_BP	6.94E-08	1.16E-07	11.111111	6	[AKT1, CDK6, FOXO1, GSK3B, IL6, PDPK1]
GO:0032677	regulation of interleukin-8 production	GO_BP	7.64E-08	1.27E-07	7.6086955	7	[HMGB1, IL1B, IL6, NOS2, RELA, STAT3, TLR4]
GO:0032637	interleukin-8 production	GO_BP	7.64E-08	1.27E-07	7.6086955	7	[HMGB1, IL1B, IL6, NOS2, RELA, STAT3, TLR4]
GO:0032368	regulation of lipid transport	GO_BP	7.63E-08	1.27E-07	5.755396	8	[AKT1, IL1B, NFKB1, NFKBIA, PPARA, PPARG, PRKCD, RXRA]
GO:1900015	regulation of cytokine production involved in inflammatory response	GO_BP	7.77E-08	1.29E-07	10.909091	6	[IL6, MAPK14, NOS2, PPARA, STAT3, TLR4]
GO:0002534	cytokine production involved in inflammatory response	GO_BP	7.77E-08	1.29E-07	10.909091	6	[IL6, MAPK14, NOS2, PPARA, STAT3, TLR4]
GO:0030323	respiratory tube development	GO_BP	7.88E-08	1.30E-07	4.591837	9	[EGFR, HMGB1, KRAS, MAP2K1, MAPK1, MAPK3, NOTCH1, SMAD2, VEGFA]
GO:1904813	ficolin-1-rich granule lumen	GO_CC	8.07E-08	1.33E-07	5.714286	8	[CAT, GSTP1, HMGB1, HSP90AA1, HSPA8, MAPK1, MAPK14, MMP9]
KEGG:05211	Renal cell carcinoma	KEGG	1.01E-08	1.95E-08	10.144928	7	[AKT1, JUN, KRAS, MAP2K1, MAPK1, MAPK3, VEGFA]
GO:1904035	regulation of epithelial cell apoptotic process	GO_BP	8.23E-08	1.36E-07	7.5268817	7	[CCL2, HMOX1, IL6, MTOR, NFE2L2, PDPK1, PPARA]
GO:0010565	regulation of cellular ketone metabolic process	GO_BP	8.59E-08	1.41E-07	4.5454545	9	[AKT1, IL1B, INS, IRS1, MTOR, PDPK1, PPARA, PPARG, PTGS2]
GO:0010833	telomere maintenance via telomere lengthening	GO_BP	8.87E-08	1.46E-07	7.4468083	7	[ATM, CDK2, HSP90AA1, MAPK1, MAPK3, PARP1, SRC]
GO:0002065	columnar/cuboidal epithelial cell differentiation	GO_BP	8.87E-08	1.46E-07	7.4468083	7	[AKT1, CDK6, FOXO1, GSK3B, NOTCH1, PDPK1, SRC]
GO:0090559	regulation of membrane permeability	GO_BP	1.03E-07	1.68E-07	7.2916665	7	[BCL2L1, CASP8, GSK3B, MAPK8, MTOR, STAT3, TP53]

GO:0060968	regulation of gene silencing	GO_BP	1.06E-07	1.73E-07	5.5172415	8	[CDK2, EGFR, ESR1, IL6, MAP2K1, PPARG, STAT3, TP53]
GO:1903800	positive regulation of production of miRNAs involved in gene silencing by miRNA	GO_BP	1.09E-07	1.78E-07	33.333332	4	[EGFR, IL6, MAP2K1, TP53]
GO:0034350	regulation of glial cell apoptotic process	GO_BP	1.09E-07	1.78E-07	33.333332	4	[CCL2, CDK5, PRKCA, PRKCD]
GO:0032755	positive regulation of interleukin-6 production	GO_BP	1.10E-07	1.80E-07	7.216495	7	[APP, HMGB1, IL1B, IL6, NOS2, STAT3, TLR4]
GO:0032091	negative regulation of protein binding	GO_BP	1.10E-07	1.80E-07	7.216495	7	[AKT1, AURKA, GSK3B, MAPK3, MAPK8, PPARA, PRKCD]
GO:1902751	positive regulation of cell cycle G2/M phase transition	GO_BP	1.11E-07	1.81E-07	16.666666	5	[APP, CCNB1, CCND1, CDK1, CDK4]
KEGG:04622	RIG-I-like receptor signaling pathway	KEGG	1.12E-08	2.14E-08	10	7	[CASP8, CHUK, MAPK14, MAPK8, NFKB1, NFKBIA, RELA]
GO:0051205	protein insertion into membrane	GO_BP	1.18E-07	1.92E-07	7.142857	7	[APP, CASP8, EGFR, HMOX1, HSP90AA1, MAPK8, TP53]
GO:0010332	response to gamma radiation	GO_BP	1.19E-07	1.93E-07	10.169492	6	[ATM, BCL2L1, H2AX, MYC, PARP1, TP53]
GO:0032642	regulation of chemokine production	GO_BP	1.27E-07	2.05E-07	7.070707	7	[APP, GSTP1, HMGB1, HMOX1, IL1B, IL6, TLR4]
GO:0032602	chemokine production	GO_BP	1.27E-07	2.05E-07	7.070707	7	[APP, GSTP1, HMGB1, HMOX1, IL1B, IL6, TLR4]
GO:0016922	nuclear receptor binding	GO_MF	1.31E-07	2.11E-07	5.3691278	8	[ESR1, NR1I2, PARP1, PPARG, RXRA, SRC, STAT1, STAT3]
GO:0003179	heart valve morphogenesis	GO_BP	1.32E-07	2.13E-07	10	6	[CCNA2, MTOR, NFATC1, NOS3, NOTCH1, RB1]
GO:2000637	positive regulation of gene silencing by miRNA	GO_BP	1.32E-07	2.13E-07	16.129032	5	[EGFR, IL6, MAP2K1, STAT3, TP53]
GO:0001223	transcription coactivator	GO_MF	1.32E-	2.13E-07	16.129032	5	[AHR, ESR1, FOXO1, PPARA, RELA]

	binding		07				
GO:0043388	positive regulation of DNA binding	GO_BP	1.46E-07	2.34E-07	9.836065	6	[HMGB1, IGF1, MMP9, MYC, PARP1, PPARG]
GO:0002067	glandular epithelial cell differentiation	GO_BP	1.46E-07	2.34E-07	9.836065	6	[AKT1, CDK6, FOXO1, GSK3B, NOTCH1, PDPK1]
GO:0034644	cellular response to UV	GO_BP	1.46E-07	2.34E-07	6.930693	7	[MMP2, MMP9, MYC, PARP1, PRKCD, PTGS2, TP53]
GO:0060148	positive regulation of posttranscriptional gene silencing	GO_BP	1.56E-07	2.50E-07	15.625	5	[EGFR, IL6, MAP2K1, STAT3, TP53]
GO:0002068	glandular epithelial cell development	GO_BP	1.56E-07	2.50E-07	15.625	5	[AKT1, CDK6, FOXO1, GSK3B, PDPK1]
GO:0030856	regulation of epithelial cell differentiation	GO_BP	1.60E-07	2.55E-07	5.2287583	8	[CCND1, EZH2, IL1B, KEAP1, MMP9, NOTCH1, STAT1, VEGFA]
GO:0007179	transforming growth factor beta receptor signaling pathway	GO_BP	1.60E-07	2.56E-07	4.2253523	9	[FOS, JUN, PARP1, PDPK1, PPARA, PTK2, SMAD2, SRC, TP53]
GO:0045428	regulation of nitric oxide biosynthetic process	GO_BP	1.61E-07	2.56E-07	9.67742	6	[AKT1, HSP90AA1, IL1B, MTOR, PTGS2, TLR4]
GO:0070498	interleukin-1-mediated signaling pathway	GO_BP	1.67E-07	2.65E-07	6.7961164	7	[CHUK, IL1B, IL6, MAPK3, NFKB1, NFKBIA, RELA]
GO:0062014	negative regulation of small molecule metabolic process	GO_BP	1.67E-07	2.65E-07	6.7961164	7	[AKT1, INS, NFKB1, PARP1, PPARA, STAT3, TP53]
GO:0000079	regulation of cyclin-dependent protein serine/threonine kinase activity	GO_BP	1.67E-07	2.65E-07	6.7961164	7	[AKT1, CASP3, CCNA2, CCNB1, CCND1, EGFR, SRC]
KEGG:04520	Adherens junction	KEGG	1.24E-08	2.37E-08	9.859155	7	[CDH1, EGFR, ERBB2, IGF1R, MAPK1, MAPK3, SRC]
GO:0045737	positive regulation of cyclin-dependent protein serine/threonine kinase	GO_BP	1.84E-07	2.90E-07	15.151515	5	[AKT1, CCNB1, CCND1, EGFR, SRC]

	activity						
GO:0038128	ERBB2 signaling pathway	GO_BP	1.84E-07	2.90E-07	15.151515	5	[EGFR, ERBB2, HSP90AA1, PRKCA, SRC]
GO:0003309	type B pancreatic cell differentiation	GO_BP	1.84E-07	2.90E-07	15.151515	5	[AKT1, CDK6, FOXO1, GSK3B, PDPK1]
GO:0007254	JNK cascade	GO_BP	1.88E-07	2.96E-07	4.147465	9	[AKT1, APP, EGFR, GSTP1, HMGB1, IGF1R, IL1B, MAPK8, TLR4]
GO:0080164	regulation of nitric oxide metabolic process	GO_BP	1.95E-07	3.07E-07	9.375	6	[AKT1, HSP90AA1, IL1B, MTOR, PTGS2, TLR4]
GO:0006953	acute-phase response	GO_BP	1.95E-07	3.07E-07	9.375	6	[FN1, IL1B, IL6, INS, PTGS2, STAT3]
GO:0070665	positive regulation of leukocyte proliferation	GO_BP	1.96E-07	3.07E-07	5.0955415	8	[HMGB1, IGF1, IL1B, IL6, MAPK1, MAPK3, PTK2, TLR4]
GO:0007006	mitochondrial membrane organization	GO_BP	1.96E-07	3.07E-07	5.0955415	8	[BCL2L1, CASP8, GSK3B, HSP90AA1, MAPK8, MYC, STAT3, TP53]
KEGG:05323	Rheumatoid arthritis	KEGG	8.23E-08	1.36E-07	7.5268817	7	[CCL2, FOS, IL1B, IL6, JUN, TLR4, VEGFA]
KEGG:04750	Inflammatory mediator regulation of TRP channels	KEGG	1.18E-07	1.92E-07	7.142857	7	[IGF1, IL1B, MAPK14, MAPK8, PRKCA, PRKCD, SRC]
GO:1901222	regulation of NIK/NF-kappaB signaling	GO_BP	2.03E-07	3.18E-07	6.6037736	7	[APP, EGFR, HMGB1, IL1B, NFKBIA, RELA, TLR4]
GO:0097194	execution phase of apoptosis	GO_BP	2.03E-07	3.18E-07	6.6037736	7	[AKT1, BCL2L1, CASP3, CASP8, HMGB1, IL6, TP53]
GO:0035195	gene silencing by miRNA	GO_BP	2.05E-07	3.21E-07	5.063291	8	[EGFR, ESR1, IL6, MAP2K1, PPARG, SMAD2, STAT3, TP53]
GO:0150077	regulation of neuroinflammatory response	GO_BP	2.15E-07	3.34E-07	14.705882	5	[IGF1, IL1B, IL6, MMP9, PTGS2]
GO:0090322	regulation of superoxide metabolic process	GO_BP	2.15E-07	3.34E-07	14.705882	5	[AKT1, EGFR, GSTP1, NFE2L2, PRKCD]

GO:0042306	regulation of protein import into nucleus	GO_BP	2.14E-07	3.34E-07	9.230769	6	[CDH1, CDK1, MAPK1, MAPK14, PRKCD, PTGS2]
GO:0008643	carbohydrate transport	GO_BP	2.16E-07	3.35E-07	5.0314465	8	[AKT1, IGF1, IL1B, INS, IRS1, MAPK14, MYC, NFE2L2]
GO:1904029	regulation of cyclin-dependent protein kinase activity	GO_BP	2.17E-07	3.36E-07	6.542056	7	[AKT1, CASP3, CCNA2, CCNB1, CCND1, EGFR, SRC]
GO:0008593	regulation of Notch signaling pathway	GO_BP	2.17E-07	3.36E-07	6.542056	7	[AKT1, EGFR, NFKBIA, NOS3, NOTCH1, SRC, STAT3]
GO:0004712	protein serine/threonine/tyrosine kinase activity	GO_BP	2.17E-07	3.36E-07	6.542056	7	[AURKA, EGFR, IGF1R, MAP2K1, MAPK1, MAPK14, MAPK3]
GO:0060541	respiratory system development	GO_BP	2.19E-07	3.39E-07	4.072398	9	[EGFR, HMGB1, KRAS, MAP2K1, MAPK1, MAPK3, NOTCH1, SMAD2, VEGFA]
GO:0000075	cell cycle checkpoint signaling	GO_BP	2.19E-07	3.39E-07	4.072398	9	[ATM, AURKA, CCNB1, CCND1, CDK1, CDK2, H2AX, MAPK14, TP53]
GO:2001243	negative regulation of intrinsic apoptotic signaling pathway	GO_BP	2.31E-07	3.57E-07	6.4814816	7	[AKT1, BCL2L1, INS, MMP9, NFE2L2, PTGS2, SRC]
GO:0032731	positive regulation of interleukin-1 beta production	GO_BP	2.35E-07	3.63E-07	9.090909	6	[APP, CASP8, HMGB1, IL6, STAT3, TLR4]
GO:0042770	signal transduction in response to DNA damage	GO_BP	2.36E-07	3.63E-07	4.0358744	9	[ATM, AURKA, CCNB1, CCND1, CDK1, CDK2, H2AX, MAPK14, TP53]
GO:0090170	regulation of Golgi inheritance	GO_BP	2.36E-07	3.64E-07	75	3	[MAP2K1, MAPK1, MAPK3]
GO:0033273	response to vitamin	GO_BP	2.46E-07	3.78E-07	6.4220185	7	[CAT, CCND1, EGFR, GSTP1, PPARG, PTGS2, RELA]
GO:0014074	response to purine-containing compound	GO_BP	2.49E-07	3.81E-07	4.9382715	8	[AHR, APP, FOS, JUN, PPARG, PTGS2, RELA, STAT1]
GO:0050321	tau-protein kinase activity	GO_BP	2.50E-07	3.82E-07	14.285714	5	[CDK5, GSK3B, HSP90AA1, IL6, RB1]

GO:0043276	anoikis	GO_BP	2.50E-07	3.82E-07	14.285714	5	[AKT1, MTOR, NOTCH1, PTK2, SRC]
GO:0034405	response to fluid shear stress	GO_BP	2.50E-07	3.82E-07	14.285714	5	[AKT1, NFE2L2, NOS3, PTGS2, SRC]
GO:0014855	striated muscle cell proliferation	GO_BP	2.57E-07	3.93E-07	8.955224	6	[CCNB1, CDK1, MAPK1, MAPK14, NOTCH1, STAT3]
GO:0007088	regulation of mitotic nuclear division	GO_BP	2.62E-07	3.99E-07	6.3636365	7	[ATM, AURKA, CCNB1, IGF1, IL1B, INS, RB1]
GO:0045471	response to ethanol	GO_BP	2.73E-07	4.16E-07	4.878049	8	[CASP8, CAT, CCND1, CDK1, GSTP1, MYC, PPARA, STAT3]
GO:0060969	negative regulation of gene silencing	GO_BP	2.89E-07	4.39E-07	13.888889	5	[ESR1, IL6, PPARG, STAT3, TP53]
GO:1903799	negative regulation of production of miRNAs involved in gene silencing by miRNA	GO_BP	2.97E-07	4.50E-07	26.666666	4	[ESR1, IL6, STAT3, TP53]
KEGG:04928	Parathyroid hormone synthesis, secretion and action	KEGG	2.03E-07	3.18E-07	6.6037736	7	[EGFR, FOS, MAP2K1, MAPK1, MAPK3, PRKCA, RXRA]
GO:0010660	regulation of muscle cell apoptotic process	GO_BP	3.07E-07	4.64E-07	8.695652	6	[HMOX1, IGF1, NFE2L2, PDPK1, PPARG, TP53]
KEGG:04725	Cholinergic synapse	KEGG	3.15E-07	4.74E-07	6.19469	7	[AKT1, FOS, KRAS, MAP2K1, MAPK1, MAPK3, PRKCA]
KEGG:04650	Natural killer cell mediated cytotoxicity	KEGG	8.60E-07	1.21E-06	5.3435116	7	[CASP3, KRAS, MAP2K1, MAPK1, MAPK3, NFATC1, PRKCA]
GO:0035194	post-transcriptional gene silencing by RNA	GO_BP	3.29E-07	4.93E-07	4.7619047	8	[EGFR, ESR1, IL6, MAP2K1, PPARG, SMAD2, STAT3, TP53]
GO:1904031	positive regulation of cyclin-dependent protein kinase activity	GO_BP	3.33E-07	4.99E-07	13.513514	5	[AKT1, CCNB1, CCND1, EGFR, SRC]
GO:0051385	response to mineralocorticoid	GO_BP	3.33E-07	4.99E-07	13.513514	5	[CCND1, FOS, KRAS, PARP1, SRC]

GO:0046320	regulation of fatty acid oxidation	GO_BP	3.33E-07	4.99E-07	13.513514	5	[AKT1, IRS1, MTOR, PPARA, PPARG]
GO:0035883	enteroendocrine cell differentiation	GO_BP	3.33E-07	4.99E-07	13.513514	5	[AKT1, CDK6, FOXO1, GSK3B, PDPK1]
GO:0030224	monocyte differentiation	GO_BP	3.33E-07	4.99E-07	13.513514	5	[CDK6, JUN, MYC, PPARG, VEGFA]
GO:1901224	positive regulation of NIK/NF-kappaB signaling	GO_BP	3.35E-07	5.01E-07	8.571428	6	[APP, EGFR, HMGB1, IL1B, RELA, TLR4]
GO:1900076	regulation of cellular response to insulin stimulus	GO_BP	3.35E-07	5.01E-07	8.571428	6	[IL1B, INS, IRS1, PRKCD, RELA, SRC]
GO:0003170	heart valve development	GO_BP	3.35E-07	5.01E-07	8.571428	6	[CCNA2, MTOR, NFATC1, NOS3, NOTCH1, RB1]
GO:2001022	positive regulation of response to DNA damage stimulus	GO_BP	3.55E-07	5.30E-07	6.0869565	7	[ATM, EGFR, H2AX, HMGB1, MYC, PARP1, PRKCD]
GO:0050806	positive regulation of synaptic transmission	GO_BP	3.60E-07	5.36E-07	4.7058825	8	[APP, CCL2, CDK5, EGFR, GSK3B, INS, MAPK1, PTGS2]
GO:0060135	maternal process involved in female pregnancy	GO_BP	3.65E-07	5.43E-07	8.450705	6	[AKT1, ESR1, MAPK1, MAPK3, MTOR, PTGS2]
GO:0043433	negative regulation of DNA-binding transcription factor activity	GO_BP	3.76E-07	5.59E-07	4.6783624	8	[CAT, CHUK, ESR1, EZH2, HMOX1, KEAP1, NFKBIA, RB1]
GO:0150076	neuroinflammatory response	GO_BP	3.83E-07	5.67E-07	13.157895	5	[IGF1, IL1B, IL6, MMP9, PTGS2]
GO:0016441	posttranscriptional gene silencing	GO_BP	3.93E-07	5.82E-07	4.6511626	8	[EGFR, ESR1, IL6, MAP2K1, PPARG, SMAD2, STAT3, TP53]
GO:0048313	Golgi inheritance	GO_BP	3.95E-07	5.84E-07	25	4	[CDK1, MAP2K1, MAPK1, MAPK3]
GO:0048308	organelle inheritance	GO_BP	3.95E-07	5.84E-07	25	4	[CDK1, MAP2K1, MAPK1, MAPK3]
GO:0035173	histone kinase activity	GO_BP	3.95E-	5.84E-07	25	4	[AURKA, CDK1, CDK2, PRKCA]

			07				
GO:0010657	muscle cell apoptotic process	GO_BP	3.97E-07	5.85E-07	8.333333	6	[HMOX1, IGF1, NFE2L2, PDPK1, PPARG, TP53]
GO:0030307	positive regulation of cell growth	GO_BP	4.11E-07	6.05E-07	4.6242776	8	[AKT1, EGFR, ERBB2, FN1, IGF1, INS, MTOR, VEGFA]
GO:0045923	positive regulation of fatty acid metabolic process	GO_BP	4.38E-07	6.43E-07	12.820513	5	[IL1B, IRS1, PPARA, PPARG, PTGS2]
GO:0007259	receptor signaling pathway via JAK-STAT	GO_BP	4.49E-07	6.57E-07	4.571429	8	[CCL2, CDK5, IGF1, IL6, NOTCH1, STAT1, STAT3, VEGFA]
GO:0071675	regulation of mononuclear cell migration	GO_BP	4.48E-07	6.58E-07	5.882353	7	[AKT1, APP, CCL2, HMGB1, MAPK1, MAPK3, PTK2]
GO:0043620	regulation of DNA-templated transcription in response to stress	GO_BP	5.02E-07	7.34E-07	5.785124	7	[HMOX1, JUN, NFE2L2, NOTCH1, RELA, TP53, VEGFA]
GO:1903201	regulation of oxidative stress-induced cell death	GO_BP	5.07E-07	7.39E-07	8	6	[AKT1, IL6, INS, NFE2L2, PARP1, TLR4]
GO:0032722	positive regulation of chemokine production	GO_BP	5.07E-07	7.39E-07	8	6	[APP, HMGB1, HMOX1, IL1B, IL6, TLR4]
GO:0043409	negative regulation of MAPK cascade	GO_BP	5.11E-07	7.44E-07	4.494382	8	[AKT1, FOXO1, GSTP1, IGF1R, IL1B, MYC, PRKCD, TLR4]
GO:0097696	receptor signaling pathway via STAT	GO_BP	5.56E-07	8.09E-07	4.4444447	8	[CCL2, CDK5, IGF1, IL6, NOTCH1, STAT1, STAT3, VEGFA]
GO:0060964	regulation of gene silencing by miRNA	GO_BP	5.62E-07	8.15E-07	5.6910567	7	[EGFR, ESR1, IL6, MAP2K1, PPARG, STAT3, TP53]
GO:0090077	foam cell differentiation	GO_BP	5.66E-07	8.20E-07	12.195122	5	[NFKB1, NFKBIA, PPARA, PPARG, STAT1]
GO:0060043	regulation of cardiac muscle cell proliferation	GO_BP	5.66E-07	8.20E-07	12.195122	5	[CCNB1, CDK1, MAPK1, MAPK14, NOTCH1]
GO:0010742	macrophage derived foam cell differentiation	GO_BP	5.66E-07	8.20E-07	12.195122	5	[NFKB1, NFKBIA, PPARA, PPARG, STAT1]
GO:0120041	positive regulation of	GO_BP	5.89E-	8.52E-07	60	3	[MAPK1, MAPK3, PTK2]

	macrophage proliferation		07				
GO:0014805	smooth muscle adaptation	GO_BP	5.89E-07	8.52E-07	60	3	[HMOX1, IL1B, NOS3]
GO:0000723	telomere maintenance	GO_BP	6.05E-07	8.73E-07	4.3956046	8	[ATM, CDK2, HSP90AA1, MAPK1, MAPK3, MYC, PARP1, SRC]
GO:0060147	regulation of posttranscriptional gene silencing	GO_BP	6.26E-07	9.03E-07	5.6	7	[EGFR, ESR1, IL6, MAP2K1, PPARG, STAT3, TP53]
GO:0044773	mitotic DNA damage checkpoint signaling	GO_BP	6.26E-07	9.03E-07	5.6	7	[ATM, AURKA, CCNB1, CCND1, CDK1, CDK2, TP53]
GO:0045840	positive regulation of mitotic nuclear division	GO_BP	6.41E-07	9.22E-07	11.904762	5	[AURKA, IGF1, IL1B, INS, RB1]
GO:0060966	regulation of gene silencing by RNA	GO_BP	6.61E-07	9.48E-07	5.5555553	7	[EGFR, ESR1, IL6, MAP2K1, PPARG, STAT3, TP53]
GO:0043279	response to alkaloid	GO_BP	6.61E-07	9.48E-07	5.5555553	7	[BCL2L1, CASP3, CCNA2, CDK5, MTOR, PPARG, RELA]
GO:0045725	positive regulation of glycogen biosynthetic process	GO_BP	6.61E-07	9.49E-07	22.222221	4	[AKT1, IGF1, INS, IRS1]
GO:0045685	regulation of glial cell differentiation	GO_BP	6.91E-07	9.89E-07	7.594937	6	[CDK1, IL6, MTOR, NOTCH1, PPARG, RELA]
GO:0030218	erythrocyte differentiation	GO_BP	6.98E-07	9.97E-07	5.5118113	7	[CASP3, CDK6, MAPK14, RB1, STAT1, STAT3, VEGFA]
GO:1902042	negative regulation of extrinsic apoptotic signaling pathway via death domain receptors	GO_BP	7.23E-07	1.03E-06	11.627907	5	[CASP8, GSK3B, HMOX1, IL6, NOS3]
GO:0007173	epidermal growth factor receptor signaling pathway	GO_BP	7.36E-07	1.05E-06	5.46875	7	[AKT1, APP, EGFR, MMP9, PDPK1, PTK2, SRC]
GO:0002040	sprouting angiogenesis	GO_BP	7.36E-07	1.05E-06	5.46875	7	[AKT1, HMGB1, HMOX1, NOTCH1, PDPK1, PTGS2, VEGFA]

GO:0032732	positive regulation of interleukin-1 production	GO_BP	7.44E-07	1.06E-06	7.5	6	[APP, CASP8, HMGB1, IL6, STAT3, TLR4]
GO:0071346	cellular response to interferon-gamma	GO_BP	7.73E-07	1.10E-06	4.255319	8	[CCL2, MYC, NOS2, PPARG, PRKCD, STAT1, TLR4, TP53]
GO:0010721	negative regulation of cell development	GO_BP	7.73E-07	1.10E-06	4.255319	8	[CDK5, GSK3B, IGF1, IL1B, IL6, NOTCH1, TP53, VEGFA]
GO:0033692	cellular polysaccharide biosynthetic process	GO_BP	8.01E-07	1.14E-06	7.4074073	6	[AKT1, GSK3B, IGF1, INS, IRS1, MTOR]
GO:0090575	RNA polymerase II transcription regulator complex	GO_CC	8.05E-07	1.14E-06	4.2328043	8	[FOS, JUN, PPARG, RB1, RXRA, SMAD2, STAT3, TP53]
GO:0044774	mitotic DNA integrity checkpoint signaling	GO_BP	8.17E-07	1.15E-06	5.3846154	7	[ATM, AURKA, CCNB1, CCND1, CDK1, CDK2, TP53]
KEGG:04623	Cytosolic DNA-sensing pathway	KEGG	1.77E-07	2.81E-07	9.523809	6	[CHUK, IL1B, IL6, NFKB1, NFKBIA, RELA]
GO:0051817	modulation of process of other organism involved in symbiotic interaction	GO_BP	8.60E-07	1.21E-06	5.3435116	7	[CASP8, HSPA8, JUN, MAPK1, MAPK3, MYC, RXRA]
GO:0048469	cell maturation	GO_BP	8.71E-07	1.23E-06	4.188482	8	[APP, AURKA, CCNB1, MTOR, PPARG, PRKCA, RB1, VEGFA]
GO:0046902	regulation of mitochondrial membrane permeability	GO_BP	9.26E-07	1.30E-06	7.2289157	6	[BCL2L1, CASP8, GSK3B, MAPK8, STAT3, TP53]
GO:0019199	transmembrane receptor protein kinase activity	GO_BP	9.80E-07	1.37E-06	4.123711	8	[APP, EGFR, ERBB2, IGF1R, MAP2K1, PRKCD, PTK2, SRC]
GO:0007004	telomere maintenance via telomerase	GO_BP	9.94E-07	1.39E-06	7.142857	6	[ATM, CDK2, HSP90AA1, MAPK1, MAPK3, SRC]
GO:2000641	regulation of early endosome to late endosome transport	GO_BP	1.04E-06	1.45E-06	20	4	[MAP2K1, MAPK1, MAPK3, SRC]
GO:0051769	regulation of nitric-oxide synthase biosynthetic process	GO_BP	1.04E-06	1.45E-06	20	4	[CCL2, GSTP1, STAT1, TLR4]

GO:0051767	nitric-oxide synthase biosynthetic process	GO_BP	1.04E-06	1.45E-06	20	4	[CCL2, GSTP1, STAT1, TLR4]
GO:0032495	response to muramyl dipeptide	GO_BP	1.04E-06	1.45E-06	20	4	[MAPK14, NFKBIA, NOTCH1, RELA]
GO:0000979	RNA polymerase II core promoter sequence-specific DNA binding	GO_MF	1.04E-06	1.45E-06	20	4	[EZH2, FOS, RELA, STAT1]
GO:0032200	telomere organization	GO_BP	1.06E-06	1.48E-06	4.0816326	8	[ATM, CDK2, HSP90AA1, MAPK1, MAPK3, MYC, PARP1, SRC]
GO:0031047	gene silencing by RNA	GO_BP	1.06E-06	1.48E-06	4.0816326	8	[EGFR, ESR1, IL6, MAP2K1, PPARG, SMAD2, STAT3, TP53]
GO:1900407	regulation of cellular response to oxidative stress	GO_BP	1.14E-06	1.59E-06	6.976744	6	[AKT1, IL6, INS, NFE2L2, PARP1, TLR4]
GO:0004708	MAP kinase kinase activity	GO_BP	1.14E-06	1.59E-06	6.976744	6	[EGFR, IGF1R, MAP2K1, MAPK1, MAPK14, MAPK3]
GO:0032606	type I interferon production	GO_BP	1.16E-06	1.62E-06	5.109489	7	[CHUK, HMGB1, HSP90AA1, NFKB1, RELA, STAT1, TLR4]
GO:0032479	regulation of type I interferon production	GO_BP	1.16E-06	1.62E-06	5.109489	7	[CHUK, HMGB1, HSP90AA1, NFKB1, RELA, STAT1, TLR4]
GO:0002688	regulation of leukocyte chemotaxis	GO_BP	1.16E-06	1.62E-06	5.109489	7	[CCL2, HMGB1, IL6, MAPK1, MAPK3, PTK2, VEGFA]
GO:0120040	regulation of macrophage proliferation	GO_BP	1.18E-06	1.63E-06	50	3	[MAPK1, MAPK3, PTK2]
GO:0060440	trachea formation	GO_BP	1.18E-06	1.63E-06	50	3	[MAP2K1, MAPK1, MAPK3]
GO:0032204	regulation of telomere maintenance	GO_BP	1.22E-06	1.70E-06	6.8965516	6	[ATM, MAPK1, MAPK3, MYC, PARP1, SRC]
GO:0006110	regulation of glycolytic process	GO_BP	1.22E-06	1.70E-06	6.8965516	6	[APP, IGF1, INS, MYC, PPARA, STAT3]
GO:0045727	positive regulation of translation	GO_BP	1.28E-06	1.78E-06	5.035971	7	[CDK4, ERBB2, IL6, MAPK1, MAPK3, MTOR, MYC]

GO:0019915	lipid storage	GO_BP	1.40E-06	1.93E-06	6.741573	6	[IL1B, IL6, NFKB1, NFKBIA, PPARA, PPARG]
GO:0010822	positive regulation of mitochondrion organization	GO_BP	1.40E-06	1.93E-06	6.741573	6	[AURKA, CASP8, GSK3B, MAPK8, MMP9, TP53]
GO:0060711	labyrinthine layer development	GO_BP	1.41E-06	1.94E-06	10.204082	5	[AKT1, CASP8, CCNA2, MAP2K1, MAPK1]
GO:0046683	response to organophosphorus	GO_BP	1.41E-06	1.95E-06	4.964539	7	[AHR, APP, FOS, JUN, PTGS2, RELA, STAT1]
GO:0051783	regulation of nuclear division	GO_BP	1.48E-06	2.04E-06	4.9295774	7	[ATM, AURKA, CCNB1, IGF1, IL1B, INS, RB1]
GO:0046677	response to antibiotic	GO_BP	1.56E-06	2.14E-06	10	5	[CASP3, CASP8, EZH2, HSP90AA1, TP53]
GO:2001169	regulation of ATP biosynthetic process	GO_BP	1.56E-06	2.14E-06	18.181818	4	[MYC, PARP1, PPARA, STAT3]
GO:0070412	R-SMAD binding	GO_MF	1.56E-06	2.14E-06	18.181818	4	[FOS, JUN, PARP1, SMAD2]
GO:0070102	interleukin-6-mediated signaling pathway	GO_BP	1.56E-06	2.14E-06	18.181818	4	[IL6, SRC, STAT1, STAT3]
GO:0043154	negative regulation of cysteine-type endopeptidase activity involved in apoptotic process	GO_BP	1.60E-06	2.18E-06	6.5934067	6	[AKT1, IL6, MMP9, PTGS2, SRC, VEGFA]
GO:0010717	regulation of epithelial to mesenchymal transition	GO_BP	1.60E-06	2.18E-06	6.5934067	6	[EZH2, IL1B, IL6, MTOR, NOTCH1, SMAD2]
GO:0002687	positive regulation of leukocyte migration	GO_BP	1.63E-06	2.22E-06	4.861111	7	[APP, HMGB1, IL6, MAPK1, MAPK3, PTK2, VEGFA]
GO:0050729	positive regulation of inflammatory response	GO_BP	1.70E-06	2.32E-06	4.827586	7	[APP, EGFR, IL1B, IL6, NFKBIA, PTGS2, TLR4]
GO:0043627	response to estrogen	GO_BP	1.70E-06	2.32E-06	6.521739	6	[CCND1, ESR1, HMOX1, MAPK1, MYC, PPARG]
GO:0060038	cardiac muscle cell	GO_BP	1.72E-	2.34E-06	9.803922	5	[CCNB1, CDK1, MAPK1, MAPK14, NOTCH1]

	proliferation		06				
GO:0032727	positive regulation of interferon-alpha production	GO_BP	1.88E-06	2.56E-06	17.391304	4	[CHUK, HMGB1, STAT1, TLR4]
GO:0031998	regulation of fatty acid beta-oxidation	GO_BP	1.88E-06	2.56E-06	17.391304	4	[AKT1, IRS1, MTOR, PPARA]
GO:0051219	phosphoprotein binding	GO_MF	1.93E-06	2.62E-06	6.382979	6	[IRS1, MAPK1, MAPK3, MTOR, RB1, SRC]
GO:0051153	regulation of striated muscle cell differentiation	GO_BP	1.93E-06	2.62E-06	6.382979	6	[EZH2, IGF1, MAPK14, MTOR, NOTCH1, PPARA]
GO:0010507	negative regulation of autophagy	GO_BP	1.93E-06	2.62E-06	6.382979	6	[AKT1, HMOX1, IL6, MTOR, STAT3, TP53]
GO:0032675	regulation of interleukin-6 production	GO_BP	1.95E-06	2.64E-06	4.7297297	7	[APP, HMGB1, IL1B, IL6, NOS2, STAT3, TLR4]
GO:0032635	interleukin-6 production	GO_BP	1.95E-06	2.64E-06	4.7297297	7	[APP, HMGB1, IL1B, IL6, NOS2, STAT3, TLR4]
GO:1902882	regulation of response to oxidative stress	GO_BP	2.18E-06	2.95E-06	6.25	6	[AKT1, IL6, INS, NFE2L2, PARP1, TLR4]
GO:0090335	regulation of brown fat cell differentiation	GO_BP	2.25E-06	3.04E-06	16.666666	4	[INS, MAPK14, MTOR, PTGS2]
GO:0038083	peptidyl-tyrosine autophosphorylation	GO_BP	2.25E-06	3.04E-06	16.666666	4	[EGFR, MAPK3, SRC, VEGFA]
GO:0036003	positive regulation of transcription from RNA polymerase II promoter in response to stress	GO_BP	2.25E-06	3.04E-06	16.666666	4	[NFE2L2, NOTCH1, TP53, VEGFA]
GO:0010888	negative regulation of lipid storage	GO_BP	2.25E-06	3.04E-06	16.666666	4	[IL6, NFKBIA, PPARA, PPARG]
GO:0003707	steroid hormone receptor activity	GO_BP	2.25E-06	3.04E-06	16.666666	4	[ESR1, ESR2, PPARA, RXRA]
GO:1904036	negative regulation of epithelial cell apoptotic	GO_BP	2.30E-06	3.09E-06	9.259259	5	[HMOX1, MTOR, NFE2L2, PDPK1, PPARA]

	process						
GO:0010823	negative regulation of mitochondrion organization	GO_BP	2.30E-06	3.09E-06	9.259259	5	[AKT1, BCL2L1, IGF1, IL6, TP53]
KEGG:04666	Fc gamma R-mediated phagocytosis	KEGG	2.32E-06	3.12E-06	6.185567	6	[AKT1, MAP2K1, MAPK1, MAPK3, PRKCA, PRKCD]
GO:0071482	cellular response to light stimulus	GO_BP	2.33E-06	3.13E-06	4.605263	7	[MMP2, MMP9, MYC, PARP1, PRKCD, PTGS2, TP53]
GO:0001837	epithelial to mesenchymal transition	GO_BP	2.33E-06	3.13E-06	4.605263	7	[EZH2, GSK3B, IL1B, IL6, MTOR, NOTCH1, SMAD2]
GO:2000378	negative regulation of reactive oxygen species metabolic process	GO_BP	2.52E-06	3.37E-06	9.090909	5	[AKT1, INS, PPARA, STAT3, TP53]
GO:0031279	regulation of cyclase activity	GO_BP	2.52E-06	3.37E-06	9.090909	5	[MAPK14, MAPK3, MAPK8, NOS2, NOS3]
GO:0007566	embryo implantation	GO_BP	2.52E-06	3.37E-06	9.090909	5	[IL1B, MMP2, MMP9, PTGS2, VEGFA]
GO:0002042	cell migration involved in sprouting angiogenesis	GO_BP	2.52E-06	3.37E-06	9.090909	5	[AKT1, HMOX1, NOTCH1, PTGS2, VEGFA]
GO:0048010	vascular endothelial growth factor receptor signaling pathway	GO_BP	2.62E-06	3.50E-06	6.060606	6	[HSP90AA1, IL1B, MAPK14, PTK2, SRC, VEGFA]
GO:0090201	negative regulation of release of cytochrome c from mitochondria	GO_BP	2.67E-06	3.57E-06	16	4	[AKT1, BCL2L1, IGF1, IL6]
GO:0051709	regulation of killing of cells of other organism	GO_BP	2.67E-06	3.57E-06	16	4	[CASP8, MAPK1, MAPK3, NOS2]
GO:0051785	positive regulation of nuclear division	GO_BP	2.76E-06	3.68E-06	8.928572	5	[AURKA, IGF1, IL1B, INS, RB1]
GO:0097306	cellular response to alcohol	GO_BP	2.77E-06	3.69E-06	6	6	[AHR, AKT1, CDH1, CDK4, MYC, PPARG]
GO:0048709	oligodendrocyte	GO_BP	2.77E-	3.69E-06	6	6	[CDK5, ERBB2, GSTP1, MTOR, NOTCH1, PPARG]

	differentiation		06				
GO:0051147	regulation of muscle cell differentiation	GO_BP	2.77E-06	3.69E-06	4.4871793	7	[EZH2, IGF1, MAPK14, MTOR, NFATC1, NOTCH1, PPARA]
GO:0033135	regulation of peptidyl-serine phosphorylation	GO_BP	2.77E-06	3.69E-06	4.4871793	7	[AKT1, APP, EGFR, HSP90AA1, IL6, PTGS2, VEGFA]
KEGG:04916	Melanogenesis	KEGG	2.94E-06	3.90E-06	5.940594	6	[GSK3B, KRAS, MAP2K1, MAPK1, MAPK3, PRKCA]
GO:0034637	cellular carbohydrate biosynthetic process	GO_BP	2.94E-06	3.90E-06	5.940594	6	[AKT1, GSK3B, IGF1, INS, IRS1, MTOR]
GO:0008585	female gonad development	GO_BP	2.94E-06	3.90E-06	5.940594	6	[BCL2L1, CASP3, ESR1, MYC, SRC, VEGFA]
GO:0002690	positive regulation of leukocyte chemotaxis	GO_BP	2.94E-06	3.90E-06	5.940594	6	[HMGB1, IL6, MAPK1, MAPK3, PTK2, VEGFA]
GO:0045912	negative regulation of carbohydrate metabolic process	GO_BP	3.01E-06	3.99E-06	8.77193	5	[GSK3B, INS, PPARA, STAT3, TP53]
GO:0010883	regulation of lipid storage	GO_BP	3.01E-06	3.99E-06	8.77193	5	[IL6, NFKB1, NFKBIA, PPARA, PPARG]
GO:0006977	DNA damage response, signal transduction by p53 class mediator resulting in cell cycle arrest	GO_BP	3.01E-06	3.99E-06	8.77193	5	[ATM, AURKA, CCNB1, CDK1, TP53]
GO:0001541	ovarian follicle development	GO_BP	3.01E-06	3.99E-06	8.77193	5	[BCL2L1, ESR1, MYC, SRC, VEGFA]
GO:2000117	negative regulation of cysteine-type endopeptidase activity	GO_BP	3.11E-06	4.12E-06	5.882353	6	[AKT1, IL6, MMP9, PTGS2, SRC, VEGFA]
GO:0045821	positive regulation of glycolytic process	GO_BP	3.15E-06	4.16E-06	15.384615	4	[APP, IGF1, INS, MYC]
GO:0071223	cellular response to lipoteichoic acid	GO_BP	3.27E-06	4.32E-06	37.5	3	[MAPK14, RELA, TLR4]

GO:0070391	response to lipoteichoic acid	GO_BP	3.27E-06	4.32E-06	37.5	3	[MAPK14, RELA, TLR4]
GO:0032071	regulation of endodeoxyribonuclease activity	GO_BP	3.27E-06	4.32E-06	37.5	3	[AKT1, HMGB1, PRKCD]
GO:0002763	positive regulation of myeloid leukocyte differentiation	GO_BP	3.28E-06	4.33E-06	8.620689	5	[CASP8, FOS, JUN, PRKCA, RB1]
GO:1903364	positive regulation of cellular protein catabolic process	GO_BP	3.42E-06	4.50E-06	4.347826	7	[AKT1, AURKA, GSK3B, HSP90AA1, KEAP1, NFE2L2, PTK2]
GO:0051591	response to cAMP	GO_BP	3.49E-06	4.58E-06	5.769231	6	[AHR, APP, FOS, JUN, RELA, STAT1]
GO:0007612	learning	GO_BP	3.56E-06	4.67E-06	4.3209877	7	[APP, CDK5, FOS, JUN, KRAS, MTOR, PTGS2]
GO:0044003	modulation by symbiont of host process	GO_BP	3.58E-06	4.68E-06	8.474576	5	[CASP8, MAPK1, MAPK3, MYC, RXRA]
GO:0032928	regulation of superoxide anion generation	GO_BP	3.69E-06	4.82E-06	14.814815	4	[AKT1, EGFR, GSTP1, PRKCD]
GO:0020037	heme binding	GO_MF	3.86E-06	5.04E-06	4.268293	7	[CAT, HMOX1, NFE2L2, NOS2, NOS3, PTGS2, SRC]
GO:0002224	toll-like receptor signaling pathway	GO_BP	3.86E-06	5.04E-06	4.268293	7	[CASP8, CHUK, ESR1, HMGB1, NFKBIA, PDPK1, TLR4]
GO:0046545	development of primary female sexual characteristics	GO_BP	3.90E-06	5.08E-06	5.6603775	6	[BCL2L1, CASP3, ESR1, MYC, SRC, VEGFA]
GO:0097110	scaffold protein binding	GO_MF	4.23E-06	5.50E-06	8.196721	5	[CASP8, HSP90AA1, MAP2K1, MAPK3, SRC]
GO:1903579	negative regulation of ATP metabolic process	GO_BP	4.29E-06	5.57E-06	14.285714	4	[PARP1, PPARA, STAT3, TP53]
GO:0010288	response to lead ion	GO_BP	4.29E-06	5.57E-06	14.285714	4	[APP, CAT, CDK4, PTGS2]

GO:1903076	regulation of protein localization to plasma membrane	GO_BP	4.34E-06	5.63E-06	5.5555553	6	[AKT1, BCL2L1, CDK5, EGFR, INS, PDPK1]
GO:0032655	regulation of interleukin-12 production	GO_BP	4.58E-06	5.94E-06	8.064516	5	[HMGB1, MAPK14, NFKB1, RELA, TLR4]
GO:0032615	interleukin-12 production	GO_BP	4.58E-06	5.94E-06	8.064516	5	[HMGB1, MAPK14, NFKB1, RELA, TLR4]
GO:0032680	regulation of tumor necrosis factor production	GO_BP	4.71E-06	6.09E-06	4.1420116	7	[APP, GSTP1, HMGB1, IGF1, IL6, STAT3, TLR4]
GO:0032640	tumor necrosis factor production	GO_BP	4.71E-06	6.09E-06	4.1420116	7	[APP, GSTP1, HMGB1, IGF1, IL6, STAT3, TLR4]
GO:0060020	Bergmann glial cell differentiation	GO_BP	4.89E-06	6.32E-06	33.333332	3	[MAP2K1, MAPK1, MAPK3]
GO:0033129	positive regulation of histone phosphorylation	GO_BP	4.89E-06	6.32E-06	33.333332	3	[CCNB1, IL1B, MAPK3]
GO:0055093	response to hyperoxia	GO_BP	4.96E-06	6.40E-06	13.793103	4	[CAT, CDK4, FOXO1, PPARG]
GO:0055001	muscle cell development	GO_BP	5.09E-06	6.54E-06	4.0935674	7	[CCNB1, CDK1, IGF1, MTOR, NOTCH1, PPARA, VEGFA]
GO:0019233	sensory perception of pain	GO_BP	5.09E-06	6.55E-06	5.4054055	6	[CCL2, CDK5, MAPK1, MAPK3, MTOR, PTGS2]
GO:2000060	positive regulation of ubiquitin-dependent protein catabolic process	GO_BP	5.36E-06	6.88E-06	5.357143	6	[AKT1, AURKA, GSK3B, KEAP1, NFE2L2, PTK2]
GO:0046906	tetrapyrrole binding	GO_MF	5.50E-06	7.04E-06	4.0462427	7	[CAT, HMOX1, NFE2L2, NOS2, NOS3, PTGS2, SRC]
GO:1905954	positive regulation of lipid localization	GO_BP	5.64E-06	7.22E-06	5.3097343	6	[IL1B, NFKB1, NFKBIA, PPARG, PRKCD, RXRA]
GO:0050996	positive regulation of lipid catabolic process	GO_BP	5.71E-06	7.29E-06	13.333333	4	[IL1B, IRS1, PPARA, PRKCD]
GO:0048147	negative regulation of	GO_BP	5.71E-	7.29E-06	13.333333	4	[GSTP1, MYC, PPARG, TP53]

	fibroblast proliferation		06				
GO:0032647	regulation of interferon-alpha production	GO_BP	5.71E-06	7.29E-06	13.333333	4	[CHUK, HMGB1, STAT1, TLR4]
GO:0032607	interferon-alpha production	GO_BP	5.71E-06	7.29E-06	13.333333	4	[CHUK, HMGB1, STAT1, TLR4]
GO:1902041	regulation of extrinsic apoptotic signaling pathway via death domain receptors	GO_BP	5.79E-06	7.39E-06	7.6923075	5	[CASP8, GSK3B, HMOX1, IL6, NOS3]
GO:1903555	regulation of tumor necrosis factor superfamily cytokine production	GO_BP	5.93E-06	7.55E-06	4	7	[APP, GSTP1, HMGB1, IGF1, IL6, STAT3, TLR4]
GO:0071706	tumor necrosis factor superfamily cytokine production	GO_BP	5.93E-06	7.55E-06	4	7	[APP, GSTP1, HMGB1, IGF1, IL6, STAT3, TLR4]
GO:0007093	mitotic cell cycle checkpoint signaling	GO_BP	5.93E-06	7.55E-06	4	7	[ATM, AURKA, CCNB1, CCND1, CDK1, CDK2, TP53]
GO:0014910	regulation of smooth muscle cell migration	GO_BP	6.25E-06	7.94E-06	7.5757575	5	[GSTP1, IGF1, MYC, NFE2L2, SRC]
GO:0043618	regulation of transcription from RNA polymerase II promoter in response to stress	GO_BP	6.25E-06	7.95E-06	5.2173915	6	[HMOX1, JUN, NFE2L2, NOTCH1, TP53, VEGFA]
GO:1903649	regulation of cytoplasmic transport	GO_BP	6.53E-06	8.28E-06	12.903226	4	[MAP2K1, MAPK1, MAPK3, SRC]
GO:0030332	cyclin binding	GO_MF	6.53E-06	8.28E-06	12.903226	4	[CDK1, CDK2, CDK4, CDK6]
GO:0010613	positive regulation of cardiac muscle hypertrophy	GO_BP	6.53E-06	8.28E-06	12.903226	4	[IGF1, MTOR, PARP1, PRKCA]
GO:0002360	T cell lineage commitment	GO_BP	6.53E-06	8.28E-06	12.903226	4	[IL6, MTOR, STAT3, TP53]
KEGG:04611	Platelet activation	KEGG	9.64E-06	1.19E-05	4.83871	6	[AKT1, MAPK1, MAPK14, MAPK3, NOS3, SRC]

GO:1904356	regulation of telomere maintenance via telomere lengthening	GO_BP	6.73E-06	8.53E-06	7.4626865	5	[ATM, MAPK1, MAPK3, PARP1, SRC]
GO:0045600	positive regulation of fat cell differentiation	GO_BP	6.73E-06	8.53E-06	7.4626865	5	[AKT1, INS, MAPK14, PPARG, PTGS2]
GO:0019395	fatty acid oxidation	GO_BP	6.90E-06	8.72E-06	5.1282053	6	[AKT1, IRS1, MAPK14, MTOR, PPARA, PPARG]
GO:0034351	negative regulation of glial cell apoptotic process	GO_BP	6.97E-06	8.80E-06	30	3	[CCL2, PRKCA, PRKCD]
GO:0033668	negative regulation by symbiont of host apoptotic process	GO_BP	6.97E-06	8.80E-06	30	3	[CASP8, MAPK1, MAPK3]
GO:0032070	regulation of deoxyribonuclease activity	GO_BP	6.97E-06	8.80E-06	30	3	[AKT1, HMGB1, PRKCD]
GO:0033138	positive regulation of peptidyl-serine phosphorylation	GO_BP	7.25E-06	9.12E-06	5.084746	6	[AKT1, APP, EGFR, HSP90AA1, PTGS2, VEGFA]
GO:0035924	cellular response to vascular endothelial growth factor stimulus	GO_BP	7.25E-06	9.13E-06	7.352941	5	[AKT1, MAPK14, NOTCH1, RELA, VEGFA]
KEGG:04728	Dopaminergic synapse	KEGG	1.38E-05	1.68E-05	4.5454545	6	[AKT1, FOS, GSK3B, MAPK14, MAPK8, PRKCA]
GO:0014742	positive regulation of muscle hypertrophy	GO_BP	7.44E-06	9.35E-06	12.5	4	[IGF1, MTOR, PARP1, PRKCA]
GO:0010165	response to X-ray	GO_BP	7.44E-06	9.35E-06	12.5	4	[ATM, CASP3, CCND1, TP53]
GO:0007263	nitric oxide mediated signal transduction	GO_BP	7.44E-06	9.35E-06	12.5	4	[EGFR, INS, NOS2, NOS3]
GO:0042826	histone deacetylase binding	GO_MF	7.61E-06	9.55E-06	5.042017	6	[CCND1, HSP90AA1, MAPK8, PARP1, RELA, TP53]
GO:0021761	limbic system development	GO_BP	7.98E-06	1.00E-05	5	6	[CASP3, CDK5, CDK6, EZH2, GSK3B, MYC]

GO:0097192	extrinsic apoptotic signaling pathway in absence of ligand	GO_BP	8.36E-06	1.05E-05	7.142857	5	[AKT1, BCL2L1, CASP3, GSK3B, IL1B]
GO:0038034	signal transduction in absence of ligand	GO_BP	8.36E-06	1.05E-05	7.142857	5	[AKT1, BCL2L1, CASP3, GSK3B, IL1B]
GO:0046660	female sex differentiation	GO_BP	8.37E-06	1.05E-05	4.958678	6	[BCL2L1, CASP3, ESR1, MYC, SRC, VEGFA]
GO:1902175	regulation of oxidative stress-induced intrinsic apoptotic signaling pathway	GO_BP	8.44E-06	1.05E-05	12.121212	4	[AKT1, INS, NFE2L2, PARP1]
GO:0071480	cellular response to gamma radiation	GO_BP	8.44E-06	1.05E-05	12.121212	4	[ATM, BCL2L1, H2AX, TP53]
GO:0046825	regulation of protein export from nucleus	GO_BP	8.44E-06	1.05E-05	12.121212	4	[CDK5, IL1B, PRKCA, TP53]
GO:0034440	lipid oxidation	GO_BP	8.78E-06	1.09E-05	4.9180326	6	[AKT1, IRS1, MAPK14, MTOR, PPARA, PPARG]
GO:0051721	protein phosphatase 2A binding	GO_MF	9.54E-06	1.19E-05	11.764706	4	[AKT1, FOXO1, STAT1, TP53]
GO:2000074	regulation of type B pancreatic cell development	GO_BP	9.56E-06	1.19E-05	27.272728	3	[AKT1, FOXO1, GSK3B]
GO:0060439	trachea morphogenesis	GO_BP	9.56E-06	1.19E-05	27.272728	3	[MAP2K1, MAPK1, MAPK3]
GO:0052150	modulation by symbiont of host apoptotic process	GO_BP	9.56E-06	1.19E-05	27.272728	3	[CASP8, MAPK1, MAPK3]
GO:0052041	negative regulation by symbiont of host programmed cell death	GO_BP	9.56E-06	1.19E-05	27.272728	3	[CASP8, MAPK1, MAPK3]
GO:0031093	platelet alpha granule lumen	GO_CC	9.61E-06	1.19E-05	6.9444447	5	[APP, FN1, IGF1, IL6, VEGFA]
KEGG:04723	Retrograde endocannabinoid signaling	KEGG	2.64E-05	3.11E-05	4.0540543	6	[MAPK1, MAPK14, MAPK3, MAPK8, PRKCA, PTGS2]

GO:0051881	regulation of mitochondrial membrane potential	GO_BP	1.03E-05	1.27E-05	6.849315	5	[AKT1, BCL2L1, MYC, PARP1, SRC]
GO:0035690	cellular response to drug	GO_BP	1.03E-05	1.27E-05	6.849315	5	[EGFR, MYC, NFE2L2, NOS2, TP53]
GO:0060674	placenta blood vessel development	GO_BP	1.07E-05	1.33E-05	11.428572	4	[AKT1, CCNA2, MAP2K1, MAPK1]
GO:0051973	positive regulation of telomerase activity	GO_BP	1.07E-05	1.33E-05	11.428572	4	[HSP90AA1, MAPK1, MAPK3, MYC]
GO:0046627	negative regulation of insulin receptor signaling pathway	GO_BP	1.07E-05	1.33E-05	11.428572	4	[IL1B, IRS1, PRKCD, RELA]
GO:0016242	negative regulation of macroautophagy	GO_BP	1.07E-05	1.33E-05	11.428572	4	[AKT1, HMOX1, MTOR, TP53]
GO:0010743	regulation of macrophage derived foam cell differentiation	GO_BP	1.07E-05	1.33E-05	11.428572	4	[NFKB1, NFKBIA, PPARA, PPARG]
GO:0003180	aortic valve morphogenesis	GO_BP	1.07E-05	1.33E-05	11.428572	4	[NFATC1, NOS3, NOTCH1, RB1]
GO:0014909	smooth muscle cell migration	GO_BP	1.10E-05	1.36E-05	6.756757	5	[GSTP1, IGF1, MYC, NFE2L2, SRC]
GO:1901030	positive regulation of mitochondrial outer membrane permeabilization involved in apoptotic signaling pathway	GO_BP	1.20E-05	1.48E-05	11.111111	4	[CASP8, GSK3B, MAPK8, TP53]
GO:0036296	response to increased oxygen levels	GO_BP	1.20E-05	1.48E-05	11.111111	4	[CAT, CDK4, FOXO1, PPARG]
GO:0001893	maternal placenta development	GO_BP	1.20E-05	1.48E-05	11.111111	4	[AKT1, MAPK1, MAPK3, PTGS2]
GO:1904375	regulation of protein localization to cell periphery	GO_BP	1.21E-05	1.49E-05	4.6511626	6	[AKT1, BCL2L1, CDK5, EGFR, INS, PDPK1]
GO:0072584	caveolin-mediated	GO_BP	1.27E-	1.56E-05	25	3	[MAPK1, MAPK3, SRC]

	endocytosis		05				
GO:0061517	macrophage proliferation	GO_BP	1.27E-05	1.56E-05	25	3	[MAPK1, MAPK3, PTK2]
GO:0014745	negative regulation of muscle adaptation	GO_BP	1.27E-05	1.56E-05	25	3	[FOXO1, MTOR, NOS3]
GO:0007077	mitotic nuclear membrane disassembly	GO_BP	1.27E-05	1.56E-05	25	3	[CCNB1, CDK1, PRKCA]
GO:0006096	glycolytic process	GO_BP	1.32E-05	1.62E-05	4.5801525	6	[APP, IGF1, INS, MYC, PPARA, STAT3]
GO:0055013	cardiac muscle cell development	GO_BP	1.34E-05	1.63E-05	6.4935064	5	[CCNB1, CDK1, IGF1, MTOR, PPARA]
GO:1904707	positive regulation of vascular associated smooth muscle cell proliferation	GO_BP	1.35E-05	1.65E-05	10.810811	4	[IGF1, JUN, MMP2, MMP9]
KEGG:04720	Long-term potentiation	KEGG	6.73E-06	8.53E-06	7.4626865	5	[KRAS, MAP2K1, MAPK1, MAPK3, PRKCA]
GO:0006757	ATP generation from ADP	GO_BP	1.38E-05	1.68E-05	4.5454545	6	[APP, IGF1, INS, MYC, PPARA, STAT3]
GO:0048565	digestive tract development	GO_BP	1.44E-05	1.75E-05	4.511278	6	[CCNB1, EGFR, NOTCH1, RB1, SMAD2, SRC]
GO:0090049	regulation of cell migration involved in sprouting angiogenesis	GO_BP	1.50E-05	1.83E-05	10.526316	4	[HMOX1, NOTCH1, PTGS2, VEGFA]
GO:0048009	insulin-like growth factor receptor signaling pathway	GO_BP	1.50E-05	1.83E-05	10.526316	4	[AKT1, IGF1, IGF1R, IRS1]
GO:0032770	positive regulation of monooxygenase activity	GO_BP	1.50E-05	1.83E-05	10.526316	4	[AKT1, IL1B, INS, KRAS]
GO:0010656	negative regulation of muscle cell apoptotic process	GO_BP	1.50E-05	1.83E-05	10.526316	4	[HMOX1, IGF1, NFE2L2, PDPK1]
GO:0061180	mammary gland epithelium	GO_BP	1.51E-	1.84E-05	6.329114	5	[AKT1, CCND1, ESR1, MAPK1, SRC]

	development		05				
GO:0071229	cellular response to acid chemical	GO_BP	1.61E-05	1.95E-05	6.25	5	[BCL2L1, MMP2, MTOR, MYC, VEGFA]
GO:1902947	regulation of tau-protein kinase activity	GO_BP	1.65E-05	2.00E-05	23.076923	3	[HSP90AA1, IL6, RB1]
GO:0045898	regulation of RNA polymerase II transcription preinitiation complex assembly	GO_BP	1.65E-05	2.00E-05	23.076923	3	[ESR1, HMGB1, TP53]
GO:0043471	regulation of cellular carbohydrate catabolic process	GO_BP	1.65E-05	2.00E-05	23.076923	3	[HMGB1, INS, TP53]
GO:0002674	negative regulation of acute inflammatory response	GO_BP	1.65E-05	2.00E-05	23.076923	3	[GSTP1, INS, PPARG]
GO:1900077	negative regulation of cellular response to insulin stimulus	GO_BP	1.67E-05	2.02E-05	10.256411	4	[IL1B, IRS1, PRKCD, RELA]
GO:0071479	cellular response to ionizing radiation	GO_BP	1.82E-05	2.19E-05	6.097561	5	[ATM, BCL2L1, H2AX, MAPK14, TP53]
GO:0007492	endoderm development	GO_BP	1.82E-05	2.19E-05	6.097561	5	[FN1, MMP2, MMP9, NOTCH1, SMAD2]
GO:0003176	aortic valve development	GO_BP	1.85E-05	2.23E-05	10	4	[NFATC1, NOS3, NOTCH1, RB1]
GO:1903052	positive regulation of proteolysis involved in cellular protein catabolic process	GO_BP	1.85E-05	2.23E-05	4.316547	6	[AKT1, AURKA, GSK3B, KEAP1, NFE2L2, PTK2]
GO:0046031	ADP metabolic process	GO_BP	1.93E-05	2.32E-05	4.285714	6	[APP, IGF1, INS, MYC, PPARA, STAT3]
GO:0061041	regulation of wound healing	GO_BP	2.01E-05	2.41E-05	4.255319	6	[HMGB1, MTOR, NFE2L2, NOS3, PRKCD, PTK2]
GO:0034103	regulation of tissue	GO_BP	2.04E-	2.45E-05	5.952381	5	[EGFR, IL6, PRKCA, SRC, TP53]

	remodeling		05				
GO:0071398	cellular response to fatty acid	GO_BP	2.04E-05	2.45E-05	9.756098	4	[CCNB1, CDK4, IRS1, SRC]
GO:0033173	calcineurin-NFAT signaling cascade	GO_BP	2.04E-05	2.45E-05	9.756098	4	[GSK3B, IGF1, MTOR, NFATC1]
GO:0032735	positive regulation of interleukin-12 production	GO_BP	2.04E-05	2.45E-05	9.756098	4	[HMGB1, MAPK14, RELA, TLR4]
GO:0032733	positive regulation of interleukin-10 production	GO_BP	2.04E-05	2.45E-05	9.756098	4	[HMGB1, IL6, STAT3, TLR4]
GO:0097284	hepatocyte apoptotic process	GO_BP	2.09E-05	2.50E-05	21.428572	3	[BCL2L1, PPARA, RB1]
GO:0033127	regulation of histone phosphorylation	GO_BP	2.09E-05	2.50E-05	21.428572	3	[CCNB1, IL1B, MAPK3]
GO:0071901	negative regulation of protein serine/threonine kinase activity	GO_BP	2.09E-05	2.50E-05	4.2253523	6	[AKT1, CASP3, GSTP1, IL1B, PRKCD, RB1]
GO:0007422	peripheral nervous system development	GO_BP	2.16E-05	2.58E-05	5.882353	5	[AKT1, CDK1, CDK5, ERBB2, RELA]
GO:0060416	response to growth hormone	GO_BP	2.25E-05	2.68E-05	9.523809	4	[AKT1, MYC, PTK2, STAT3]
GO:0014037	Schwann cell differentiation	GO_BP	2.25E-05	2.68E-05	9.523809	4	[AKT1, CDK1, CDK5, RELA]
GO:0090101	negative regulation of transmembrane receptor protein serine/threonine kinase signaling pathway	GO_BP	2.35E-05	2.79E-05	4.137931	6	[NOTCH1, PDPK1, PPARA, PPARG, SMAD2, TP53]
GO:0055123	digestive system development	GO_BP	2.35E-05	2.79E-05	4.137931	6	[CCNB1, EGFR, NOTCH1, RB1, SMAD2, SRC]
GO:0031145	anaphase-promoting complex-dependent catabolic process	GO_BP	2.42E-05	2.88E-05	5.7471266	5	[AURKA, CCNB1, CDK1, CDK2, RB1]

GO:0035264	multicellular organism growth	GO_BP	2.44E-05	2.90E-05	4.109589	6	[APP, CDK4, IGF1, MTOR, STAT3, TP53]
GO:0071392	cellular response to estradiol stimulus	GO_BP	2.47E-05	2.93E-05	9.302325	4	[CCNA2, EGFR, ESR1, ESR2]
GO:0045022	early endosome to late endosome transport	GO_BP	2.47E-05	2.93E-05	9.302325	4	[MAP2K1, MAPK1, MAPK3, SRC]
GO:0042554	superoxide anion generation	GO_BP	2.47E-05	2.93E-05	9.302325	4	[AKT1, EGFR, GSTP1, PRKCD]
GO:0030331	estrogen receptor binding	GO_MF	2.47E-05	2.93E-05	9.302325	4	[ESR1, PARP1, PPARG, SRC]
GO:0010543	regulation of platelet activation	GO_BP	2.47E-05	2.93E-05	9.302325	4	[NOS3, PRKCA, PRKCD, TLR4]
GO:0032370	positive regulation of lipid transport	GO_BP	2.56E-05	3.03E-05	5.681818	5	[IL1B, NFKBIA, PPARG, PRKCD, RXRA]
GO:0021766	hippocampus development	GO_BP	2.56E-05	3.03E-05	5.681818	5	[CASP3, CDK5, CDK6, EZH2, GSK3B]
GO:0008306	associative learning	GO_BP	2.56E-05	3.03E-05	5.681818	5	[APP, CDK5, FOS, KRAS, MTOR]
GO:2001171	positive regulation of ATP biosynthetic process	GO_BP	2.61E-05	3.08E-05	20	3	[MYC, PPARA, STAT3]
GO:0052040	modulation by symbiont of host programmed cell death	GO_BP	2.61E-05	3.08E-05	20	3	[CASP8, MAPK1, MAPK3]
GO:0051974	negative regulation of telomerase activity	GO_BP	2.61E-05	3.08E-05	20	3	[PPARG, SRC, TP53]
GO:0051081	nuclear membrane disassembly	GO_BP	2.61E-05	3.08E-05	20	3	[CCNB1, CDK1, PRKCA]
GO:0046146	tetrahydrobiopterin metabolic process	GO_BP	2.61E-05	3.08E-05	20	3	[AKT1, HSP90AA1, NOS3]
GO:0033197	response to vitamin E	GO_BP	2.61E-05	3.08E-05	20	3	[CAT, CCND1, PPARG]
GO:0031053	primary miRNA processing	GO_BP	2.61E-	3.08E-05	20	3	[IL6, SMAD2, STAT3]

			05				
GO:0030397	membrane disassembly	GO_BP	2.61E-05	3.08E-05	20	3	[CCNB1, CDK1, PRKCA]
GO:0010745	negative regulation of macrophage derived foam cell differentiation	GO_BP	2.61E-05	3.08E-05	20	3	[NFKBIA, PPARA, PPARG]
KEGG:04670	Leukocyte transendothelial migration	KEGG	8.86E-05	1.00E-04	4.385965	5	[MAPK14, MMP2, MMP9, PRKCA, PTK2]
GO:1905314	semi-lunar valve development	GO_BP	2.71E-05	3.19E-05	9.090909	4	[NFATC1, NOS3, NOTCH1, RB1]
GO:0008625	extrinsic apoptotic signaling pathway via death domain receptors	GO_BP	2.85E-05	3.36E-05	5.5555553	5	[CASP8, GSK3B, HMOX1, IL6, NOS3]
GO:0045687	positive regulation of glial cell differentiation	GO_BP	2.97E-05	3.48E-05	8.888889	4	[MTOR, NOTCH1, PPARG, RELA]
GO:0030212	hyaluronan metabolic process	GO_BP	2.97E-05	3.48E-05	8.888889	4	[AKT1, IL1B, IL6, NFKB1]
GO:0014812	muscle cell migration	GO_BP	3.17E-05	3.72E-05	5.4347825	5	[GSTP1, IGF1, MYC, NFE2L2, SRC]
GO:0006970	response to osmotic stress	GO_BP	3.17E-05	3.72E-05	5.4347825	5	[CASP3, EGFR, PDPK1, PTGS2, TP53]
GO:0051770	positive regulation of nitric-oxide synthase biosynthetic process	GO_BP	3.20E-05	3.74E-05	18.75	3	[CCL2, STAT1, TLR4]
GO:0046321	positive regulation of fatty acid oxidation	GO_BP	3.20E-05	3.74E-05	18.75	3	[IRS1, PPARA, PPARG]
GO:0045651	positive regulation of macrophage differentiation	GO_BP	3.20E-05	3.74E-05	18.75	3	[CASP8, PRKCA, RB1]
GO:1901028	regulation of mitochondrial outer membrane permeabilization involved in apoptotic signaling pathway	GO_BP	3.24E-05	3.79E-05	8.695652	4	[CASP8, GSK3B, MAPK8, TP53]

GO:0098927	vesicle-mediated transport between endosomal compartments	GO_BP	3.24E-05	3.79E-05	8.695652	4	[MAP2K1, MAPK1, MAPK3, SRC]
GO:0097720	calcineurin-mediated signaling	GO_BP	3.24E-05	3.79E-05	8.695652	4	[GSK3B, IGF1, MTOR, NFATC1]
GO:0032436	positive regulation of proteasomal ubiquitin-dependent protein catabolic process	GO_BP	3.52E-05	4.11E-05	5.319149	5	[AKT1, AURKA, GSK3B, KEAP1, NFE2L2]
GO:0001892	embryonic placenta development	GO_BP	3.52E-05	4.11E-05	5.319149	5	[AKT1, CASP8, CCNA2, MAP2K1, MAPK1]
GO:1903426	regulation of reactive oxygen species biosynthetic process	GO_BP	3.53E-05	4.12E-05	8.510638	4	[INS, PPARA, STAT3, TLR4]
GO:1902808	positive regulation of cell cycle G1/S phase transition	GO_BP	3.53E-05	4.12E-05	8.510638	4	[AKT1, CCND1, EGFR, EZH2]
GO:0060324	face development	GO_BP	3.53E-05	4.12E-05	8.510638	4	[MAP2K1, MAPK1, MAPK3, MMP2]
GO:0043124	negative regulation of I-kappaB kinase/NF-kappaB signaling	GO_BP	3.53E-05	4.12E-05	8.510638	4	[CASP8, ESR1, GSTP1, STAT1]
GO:0042088	T-helper 1 type immune response	GO_BP	3.53E-05	4.12E-05	8.510638	4	[HMGB1, IL1B, MTOR, TLR4]
GO:2000811	negative regulation of anoikis	GO_BP	3.87E-05	4.51E-05	17.647058	3	[NOTCH1, PTK2, SRC]
GO:0090336	positive regulation of brown fat cell differentiation	GO_BP	3.87E-05	4.51E-05	17.647058	3	[INS, MAPK14, PTGS2]
GO:0045120	pronucleus	GO_CC	3.87E-05	4.51E-05	17.647058	3	[AURKA, CCNA2, EZH2]
GO:0016004	phospholipase activator activity	GO_MF	3.87E-05	4.51E-05	17.647058	3	[CASP3, PDPK1, SRC]
GO:0005159	insulin-like growth factor	GO_MF	3.87E-	4.51E-05	17.647058	3	[IGF1, INS, IRS1]

	receptor binding		05				
GO:0031091	platelet alpha granule	GO_CC	4.10E-05	4.76E-05	5.1546392	5	[APP, FN1, IGF1, IL6, VEGFA]
KEGG:04215	Apoptosis	KEGG	7.44E-06	9.35E-06	12.5	4	[BCL2L1, CASP3, CASP8, MAPK8]
GO:0070303	negative regulation of stress-activated protein kinase signaling cascade	GO_BP	4.17E-05	4.84E-05	8.163265	4	[AKT1, FOXO1, GSTP1, MYC]
GO:0070266	necroptotic process	GO_BP	4.17E-05	4.84E-05	8.163265	4	[CASP8, HSP90AA1, TLR4, TP53]
GO:0060425	lung morphogenesis	GO_BP	4.17E-05	4.84E-05	8.163265	4	[KRAS, MAP2K1, MAPK1, MAPK3]
GO:0048246	macrophage chemotaxis	GO_BP	4.17E-05	4.84E-05	8.163265	4	[CCL2, MAPK1, MAPK3, PTK2]
GO:0045646	regulation of erythrocyte differentiation	GO_BP	4.17E-05	4.84E-05	8.163265	4	[CDK6, MAPK14, STAT1, STAT3]
GO:0038066	p38MAPK cascade	GO_BP	4.17E-05	4.84E-05	8.163265	4	[IL1B, IL6, MAPK14, VEGFA]
GO:0032873	negative regulation of stress-activated MAPK cascade	GO_BP	4.17E-05	4.84E-05	8.163265	4	[AKT1, FOXO1, GSTP1, MYC]
GO:0106027	neuron projection organization	GO_BP	4.52E-05	5.23E-05	5.050505	5	[APP, CDK5, GSK3B, IGF1R, INS]
GO:0031058	positive regulation of histone modification	GO_BP	4.52E-05	5.23E-05	5.050505	5	[CCNB1, IL1B, MAPK3, TP53, VEGFA]
KEGG:05030	Cocaine addiction	KEGG	4.17E-05	4.84E-05	8.163265	4	[CDK5, JUN, NFKB1, RELA]
GO:0051204	protein insertion into mitochondrial membrane	GO_BP	4.52E-05	5.24E-05	8	4	[CASP8, HSP90AA1, MAPK8, TP53]
GO:0048156	tau protein binding	GO_MF	4.52E-05	5.24E-05	8	4	[CDK5, GSK3B, HSP90AA1, SMAD2]

GO:0045124	regulation of bone resorption	GO_BP	4.52E-05	5.24E-05	8	4	[EGFR, IL6, PRKCA, SRC]
GO:1902894	negative regulation of pri-miRNA transcription by RNA polymerase II	GO_BP	4.63E-05	5.36E-05	16.666666	3	[PPARA, PPARG, RELA]
GO:0071732	cellular response to nitric oxide	GO_BP	4.63E-05	5.36E-05	16.666666	3	[CCNA2, CDK2, FOXO1]
GO:0003184	pulmonary valve morphogenesis	GO_BP	4.63E-05	5.36E-05	16.666666	3	[NFATC1, NOS3, NOTCH1]
KEGG:05144	Malaria	KEGG	4.52E-05	5.24E-05	8	4	[CCL2, IL1B, IL6, TLR4]
GO:0140296	general transcription initiation factor binding	GO_MF	4.89E-05	5.64E-05	7.8431373	4	[AHR, ESR1, MTOR, TP53]
GO:0016538	cyclin-dependent protein serine/threonine kinase regulator activity	GO_BP	4.89E-05	5.64E-05	7.8431373	4	[CASP3, CCNA2, CCNB1, CCND1]
GO:0010677	negative regulation of cellular carbohydrate metabolic process	GO_BP	4.89E-05	5.64E-05	7.8431373	4	[GSK3B, INS, PPARA, STAT3]
GO:0008542	visual learning	GO_BP	4.89E-05	5.64E-05	7.8431373	4	[APP, CDK5, KRAS, MTOR]
GO:0042100	B cell proliferation	GO_BP	4.97E-05	5.74E-05	4.9504952	5	[AHR, ATM, CASP3, PRKCD, TLR4]
GO:0030316	osteoclast differentiation	GO_BP	4.97E-05	5.74E-05	4.9504952	5	[FOS, MAPK14, MTOR, SRC, TLR4]
GO:1903358	regulation of Golgi organization	GO_BP	5.49E-05	6.32E-05	15.789474	3	[MAP2K1, MAPK1, MAPK3]
GO:0071637	regulation of monocyte chemotactic protein-1 production	GO_BP	5.49E-05	6.32E-05	15.789474	3	[GSTP1, HMGB1, IL1B]
GO:0071605	monocyte chemotactic protein-1 production	GO_BP	5.49E-05	6.32E-05	15.789474	3	[GSTP1, HMGB1, IL1B]

GO:0071498	cellular response to fluid shear stress	GO_BP	5.49E-05	6.32E-05	15.789474	3	[NFE2L2, PTGS2, SRC]
GO:0060438	trachea development	GO_BP	5.49E-05	6.32E-05	15.789474	3	[MAP2K1, MAPK1, MAPK3]
GO:0060229	lipase activator activity	GO_MF	5.49E-05	6.32E-05	15.789474	3	[CASP3, PDPK1, SRC]
GO:0002295	T-helper cell lineage commitment	GO_BP	5.49E-05	6.32E-05	15.789474	3	[IL6, MTOR, STAT3]
GO:1903202	negative regulation of oxidative stress-induced cell death	GO_BP	5.69E-05	6.55E-05	7.5471697	4	[AKT1, IL6, INS, NFE2L2]
GO:1902106	negative regulation of leukocyte differentiation	GO_BP	5.72E-05	6.57E-05	4.8076925	5	[CDK6, ERBB2, HMGB1, MYC, TLR4]
GO:0050873	brown fat cell differentiation	GO_BP	6.13E-05	7.03E-05	7.4074073	4	[INS, MAPK14, MTOR, PTGS2]
GO:0043331	response to dsRNA	GO_BP	6.13E-05	7.03E-05	7.4074073	4	[MAPK1, MAPK3, NFKB1, NFKBIA]
GO:0001706	endoderm formation	GO_BP	6.13E-05	7.03E-05	7.4074073	4	[FN1, MMP2, MMP9, SMAD2]
GO:1903707	negative regulation of hemopoiesis	GO_BP	6.27E-05	7.18E-05	4.716981	5	[CDK6, ERBB2, HMGB1, MYC, TLR4]
GO:0032760	positive regulation of tumor necrosis factor production	GO_BP	6.27E-05	7.18E-05	4.716981	5	[APP, HMGB1, IL6, STAT3, TLR4]
GO:1905564	positive regulation of vascular endothelial cell proliferation	GO_BP	6.44E-05	7.36E-05	15	3	[HMGB1, PDPK1, STAT3]
GO:1903427	negative regulation of reactive oxygen species biosynthetic process	GO_BP	6.44E-05	7.36E-05	15	3	[INS, PPARA, STAT3]
GO:1902170	cellular response to reactive nitrogen species	GO_BP	6.44E-05	7.36E-05	15	3	[CCNA2, CDK2, FOXO1]

GO:0061377	mammary gland lobule development	GO_BP	6.44E-05	7.36E-05	15	3	[CCND1, ESR1, VEGFA]
GO:0060749	mammary gland alveolus development	GO_BP	6.44E-05	7.36E-05	15	3	[CCND1, ESR1, VEGFA]
GO:0046827	positive regulation of protein export from nucleus	GO_BP	6.44E-05	7.36E-05	15	3	[IL1B, PRKCA, TP53]
GO:0045655	regulation of monocyte differentiation	GO_BP	6.44E-05	7.36E-05	15	3	[CDK6, JUN, MYC]
GO:0030730	sequestering of triglyceride	GO_BP	6.44E-05	7.36E-05	15	3	[IL1B, PPARA, PPARG]
GO:0010759	positive regulation of macrophage chemotaxis	GO_BP	6.44E-05	7.36E-05	15	3	[MAPK1, MAPK3, PTK2]
GO:0007252	I-kappaB phosphorylation	GO_BP	6.44E-05	7.36E-05	15	3	[AKT1, CHUK, TLR4]
GO:0090151	establishment of protein localization to mitochondrial membrane	GO_BP	6.59E-05	7.53E-05	7.2727275	4	[CASP8, HSP90AA1, MAPK8, TP53]
GO:1903557	positive regulation of tumor necrosis factor superfamily cytokine production	GO_BP	7.48E-05	8.53E-05	4.5454545	5	[APP, HMGB1, IL6, STAT3, TLR4]
GO:1904706	negative regulation of vascular associated smooth muscle cell proliferation	GO_BP	7.49E-05	8.53E-05	14.285714	3	[GSTP1, HMOX1, PPARG]
KEGG:04913	Ovarian steroidogenesis	KEGG	4.89E-05	5.64E-05	7.8431373	4	[IGF1, IGF1R, INS, PTGS2]
GO:0097345	mitochondrial outer membrane permeabilization	GO_BP	7.59E-05	8.63E-05	7.017544	4	[CASP8, GSK3B, MAPK8, TP53]
GO:0097300	programmed necrotic cell death	GO_BP	7.59E-05	8.63E-05	7.017544	4	[CASP8, HSP90AA1, TLR4, TP53]
GO:0007632	visual behavior	GO_BP	7.59E-05	8.63E-05	7.017544	4	[APP, CDK5, KRAS, MTOR]

GO:0007044	cell-substrate junction assembly	GO_BP	7.81E-05	8.87E-05	4.5045047	5	[FN1, PDPK1, PTK2, SRC, VEGFA]
GO:0072132	mesenchyme morphogenesis	GO_BP	8.12E-05	9.22E-05	6.8965516	4	[MYC, NOS3, NOTCH1, SMAD2]
GO:0032653	regulation of interleukin-10 production	GO_BP	8.12E-05	9.22E-05	6.8965516	4	[HMGB1, IL6, STAT3, TLR4]
GO:0032613	interleukin-10 production	GO_BP	8.12E-05	9.22E-05	6.8965516	4	[HMGB1, IL6, STAT3, TLR4]
GO:0006754	ATP biosynthetic process	GO_BP	8.12E-05	9.22E-05	6.8965516	4	[MYC, PARP1, PPARA, STAT3]
GO:0055007	cardiac muscle cell differentiation	GO_BP	8.14E-05	9.23E-05	4.464286	5	[CCNB1, CDK1, IGF1, MTOR, PPARA]
GO:1900543	negative regulation of purine nucleotide metabolic process	GO_BP	8.64E-05	9.78E-05	13.636364	3	[PARP1, PPARA, STAT3]
GO:0061050	regulation of cell growth involved in cardiac muscle cell development	GO_BP	8.64E-05	9.78E-05	13.636364	3	[IGF1, MTOR, PPARA]
GO:0043373	CD4-positive, alpha-beta T cell lineage commitment	GO_BP	8.64E-05	9.78E-05	13.636364	3	[IL6, MTOR, STAT3]
GO:0032069	regulation of nuclease activity	GO_BP	8.64E-05	9.78E-05	13.636364	3	[AKT1, HMGB1, PRKCD]
GO:0003177	pulmonary valve development	GO_BP	8.64E-05	9.78E-05	13.636364	3	[NFATC1, NOS3, NOTCH1]
GO:1903409	reactive oxygen species biosynthetic process	GO_BP	8.69E-05	9.82E-05	6.779661	4	[INS, PPARA, STAT3, TLR4]
GO:0046850	regulation of bone remodeling	GO_BP	8.69E-05	9.82E-05	6.779661	4	[EGFR, IL6, PRKCA, SRC]
GO:0045747	positive regulation of Notch signaling pathway	GO_BP	8.69E-05	9.82E-05	6.779661	4	[NOS3, NOTCH1, SRC, STAT3]
GO:0043525	positive regulation of	GO_BP	8.69E-	9.82E-05	6.779661	4	[CASP3, CDK5, JUN, TP53]

	neuron apoptotic process		05				
KEGG:04923	Regulation of lipolysis in adipocytes	KEGG	7.59E-05	8.63E-05	7.017544	4	[AKT1, INS, IRS1, PTGS2]
GO:0071870	cellular response to catecholamine stimulus	GO_BP	8.86E-05	1.00E-04	4.385965	5	[APP, GSK3B, MAPK1, MAPK3, PRKCA]
GO:0071868	cellular response to monoamine stimulus	GO_BP	8.86E-05	1.00E-04	4.385965	5	[APP, GSK3B, MAPK1, MAPK3, PRKCA]
GO:0002286	T cell activation involved in immune response	GO_BP	9.23E-05	1.04E-04	4.347826	5	[HMGB1, IL6, MTOR, STAT3, TP53]
GO:1902110	positive regulation of mitochondrial membrane permeability involved in apoptotic process	GO_BP	9.28E-05	1.04E-04	6.6666665	4	[CASP8, GSK3B, MAPK8, TP53]
GO:0090303	positive regulation of wound healing	GO_BP	9.28E-05	1.04E-04	6.6666665	4	[HMGB1, MTOR, NFE2L2, PTK2]
GO:0032210	regulation of telomere maintenance via telomerase	GO_BP	9.28E-05	1.04E-04	6.6666665	4	[ATM, MAPK1, MAPK3, SRC]
GO:0098869	cellular oxidant detoxification	GO_BP	9.62E-05	1.08E-04	4.3103447	5	[CAT, GSTP1, NFE2L2, NOS3, PTGS2]
GO:0030330	DNA damage response, signal transduction by p53 class mediator	GO_BP	9.62E-05	1.08E-04	4.3103447	5	[ATM, AURKA, CCNB1, CDK1, TP53]
GO:1903672	positive regulation of sprouting angiogenesis	GO_BP	9.91E-05	1.11E-04	13.043478	3	[HMGB1, PDPK1, VEGFA]
GO:1902176	negative regulation of oxidative stress-induced intrinsic apoptotic signaling pathway	GO_BP	9.91E-05	1.11E-04	13.043478	3	[AKT1, INS, NFE2L2]
GO:1900017	positive regulation of cytokine production involved in inflammatory response	GO_BP	9.91E-05	1.11E-04	13.043478	3	[IL6, STAT3, TLR4]

GO:0090050	positive regulation of cell migration involved in sprouting angiogenesis	GO_BP	9.91E-05	1.11E-04	13.043478	3	[HMOX1, PTGS2, VEGFA]
GO:0071731	response to nitric oxide	GO_BP	9.91E-05	1.11E-04	13.043478	3	[CCNA2, CDK2, FOXO1]
GO:0060716	labyrinthine layer blood vessel development	GO_BP	9.91E-05	1.11E-04	13.043478	3	[AKT1, CCNA2, MAPK1]
GO:0045980	negative regulation of nucleotide metabolic process	GO_BP	9.91E-05	1.11E-04	13.043478	3	[PARP1, PPARA, STAT3]
GO:0045649	regulation of macrophage differentiation	GO_BP	9.91E-05	1.11E-04	13.043478	3	[CASP8, PRKCA, RB1]
GO:0032930	positive regulation of superoxide anion generation	GO_BP	9.91E-05	1.11E-04	13.043478	3	[EGFR, GSTP1, PRKCD]
GO:0001091	RNA polymerase II general transcription initiation factor binding	GO_MF	9.91E-05	1.11E-04	13.043478	3	[AHR, ESR1, TP53]
GO:0006611	protein export from nucleus	GO_BP	9.90E-05	1.11E-04	6.557377	4	[CDK5, IL1B, PRKCA, TP53]
GO:0150115	cell-substrate junction organization	GO_BP	1.00E-04	1.12E-04	4.2735043	5	[FN1, PDPK1, PTK2, SRC, VEGFA]
GO:0071869	response to catecholamine	GO_BP	1.04E-04	1.17E-04	4.237288	5	[APP, GSK3B, MAPK1, MAPK3, PRKCA]
GO:0071867	response to monoamine	GO_BP	1.04E-04	1.17E-04	4.237288	5	[APP, GSK3B, MAPK1, MAPK3, PRKCA]
GO:0031341	regulation of cell killing	GO_BP	1.04E-04	1.17E-04	4.237288	5	[CASP8, MAPK1, MAPK3, MAPK8, NOS2]
GO:0006275	regulation of DNA replication	GO_BP	1.04E-04	1.17E-04	4.237288	5	[CCNA2, CDK1, EGFR, JUN, TP53]
GO:1902686	mitochondrial outer membrane permeabilization involved in programmed	GO_BP	1.06E-04	1.18E-04	6.451613	4	[CASP8, GSK3B, MAPK8, TP53]

	cell death						
GO:0048016	inositol phosphate-mediated signaling	GO_BP	1.06E-04	1.18E-04	6.451613	4	[GSK3B, IGF1, MTOR, NFATC1]
GO:1901800	positive regulation of proteasomal protein catabolic process	GO_BP	1.09E-04	1.21E-04	4.2016807	5	[AKT1, AURKA, GSK3B, KEAP1, NFE2L2]
GO:0032874	positive regulation of stress-activated MAPK cascade	GO_BP	1.09E-04	1.21E-04	4.2016807	5	[APP, HMGB1, IL1B, TLR4, VEGFA]
GO:0048143	astrocyte activation	GO_BP	1.13E-04	1.26E-04	12.5	3	[APP, EGFR, IL6]
GO:0036120	cellular response to platelet-derived growth factor stimulus	GO_BP	1.13E-04	1.26E-04	12.5	3	[CCNA2, MYC, SRC]
GO:0010226	response to lithium ion	GO_BP	1.13E-04	1.26E-04	12.5	3	[CDH1, NFE2L2, PTGS2]
GO:0002363	alpha-beta T cell lineage commitment	GO_BP	1.13E-04	1.26E-04	12.5	3	[IL6, MTOR, STAT3]
GO:0070304	positive regulation of stress-activated protein kinase signaling cascade	GO_BP	1.17E-04	1.31E-04	4.132231	5	[APP, HMGB1, IL1B, TLR4, VEGFA]
GO:0030278	regulation of ossification	GO_BP	1.17E-04	1.31E-04	4.132231	5	[CCNA2, MAPK1, MAPK14, MAPK3, NOTCH1]
GO:0035794	positive regulation of mitochondrial membrane permeability	GO_BP	1.20E-04	1.33E-04	6.25	4	[CASP8, GSK3B, MAPK8, TP53]
GO:0010508	positive regulation of autophagy	GO_BP	1.22E-04	1.35E-04	4.0983605	5	[FOXO1, GSK3B, HMGB1, HMOX1, MAPK3]
GO:1905517	macrophage migration	GO_BP	1.27E-04	1.40E-04	6.1538463	4	[CCL2, MAPK1, MAPK3, PTK2]

GO:1902108	regulation of mitochondrial membrane permeability involved in apoptotic process	GO_BP	1.27E-04	1.40E-04	6.1538463	4	[CASP8, GSK3B, MAPK8, TP53]
GO:0030888	regulation of B cell proliferation	GO_BP	1.27E-04	1.40E-04	6.1538463	4	[AHR, ATM, CASP3, TLR4]
GO:0001704	formation of primary germ layer	GO_BP	1.27E-04	1.41E-04	4.0650406	5	[FN1, MMP2, MMP9, PRKCA, SMAD2]
GO:0046628	positive regulation of insulin receptor signaling pathway	GO_BP	1.28E-04	1.41E-04	12	3	[INS, IRS1, SRC]
GO:0043369	CD4-positive or CD8-positive, alpha-beta T cell lineage commitment	GO_BP	1.28E-04	1.41E-04	12	3	[IL6, MTOR, STAT3]
GO:0036119	response to platelet-derived growth factor	GO_BP	1.28E-04	1.41E-04	12	3	[CCNA2, MYC, SRC]
GO:0006309	apoptotic DNA fragmentation	GO_BP	1.28E-04	1.41E-04	12	3	[CASP3, HMGB1, IL6]
GO:0002053	positive regulation of mesenchymal cell proliferation	GO_BP	1.28E-04	1.41E-04	12	3	[MYC, STAT1, VEGFA]
GO:0051353	positive regulation of oxidoreductase activity	GO_BP	1.35E-04	1.49E-04	6.060606	4	[AKT1, IL1B, INS, KRAS]
GO:0051148	negative regulation of muscle cell differentiation	GO_BP	1.35E-04	1.49E-04	6.060606	4	[EZH2, NFATC1, NOTCH1, PPARA]
GO:0030520	intracellular estrogen receptor signaling pathway	GO_BP	1.35E-04	1.49E-04	6.060606	4	[ESR1, ESR2, PARP1, SRC]
GO:0032374	regulation of cholesterol transport	GO_BP	1.43E-04	1.57E-04	5.970149	4	[NFKB1, NFKBIA, PPARG, RXRA]
GO:0032371	regulation of sterol transport	GO_BP	1.43E-04	1.57E-04	5.970149	4	[NFKB1, NFKBIA, PPARG, RXRA]
GO:2000737	negative regulation of stem cell differentiation	GO_BP	1.44E-04	1.59E-04	11.538462	3	[NFE2L2, NOTCH1, STAT3]

GO:2000209	regulation of anoikis	GO_BP	1.44E-04	1.59E-04	11.538462	3	[NOTCH1, PTK2, SRC]
GO:0051000	positive regulation of nitric-oxide synthase activity	GO_BP	1.44E-04	1.59E-04	11.538462	3	[AKT1, INS, KRAS]
GO:0010875	positive regulation of cholesterol efflux	GO_BP	1.44E-04	1.59E-04	11.538462	3	[NFKBIA, PPARG, RXRA]
GO:1905710	positive regulation of membrane permeability	GO_BP	1.51E-04	1.66E-04	5.882353	4	[CASP8, GSK3B, MAPK8, TP53]
GO:0040014	regulation of multicellular organism growth	GO_BP	1.51E-04	1.66E-04	5.882353	4	[APP, CDK4, IGF1, STAT3]
GO:0035004	phosphatidylinositol 3-kinase activity	GO_BP	1.51E-04	1.66E-04	5.882353	4	[ATM, IRS1, PTK2, SRC]
GO:0002294	CD4-positive, alpha-beta T cell differentiation involved in immune response	GO_BP	1.51E-04	1.66E-04	5.882353	4	[HMGB1, IL6, MTOR, STAT3]
GO:0043550	regulation of lipid kinase activity	GO_BP	1.60E-04	1.76E-04	5.7971015	4	[IRS1, PTK2, RB1, SRC]
GO:0002293	alpha-beta T cell differentiation involved in immune response	GO_BP	1.60E-04	1.76E-04	5.7971015	4	[HMGB1, IL6, MTOR, STAT3]
GO:0002287	alpha-beta T cell activation involved in immune response	GO_BP	1.60E-04	1.76E-04	5.7971015	4	[HMGB1, IL6, MTOR, STAT3]
GO:1905523	positive regulation of macrophage migration	GO_BP	1.62E-04	1.77E-04	11.111111	3	[MAPK1, MAPK3, PTK2]
GO:1904353	regulation of telomere capping	GO_BP	1.62E-04	1.77E-04	11.111111	3	[ATM, MAPK1, MAPK3]
GO:1900740	positive regulation of protein insertion into mitochondrial membrane involved in apoptotic signaling pathway	GO_BP	1.62E-04	1.77E-04	11.111111	3	[CASP8, MAPK8, TP53]

GO:1900739	regulation of protein insertion into mitochondrial membrane involved in apoptotic signaling pathway	GO_BP	1.62E-04	1.77E-04	11.111111	3	[CASP8, MAPK8, TP53]
GO:0097199	cysteine-type endopeptidase activity involved in apoptotic signaling pathway	GO_BP	1.62E-04	1.77E-04	11.111111	3	[CASP3, CASP8, MMP9]
GO:0046697	decidualization	GO_BP	1.62E-04	1.77E-04	11.111111	3	[MAPK1, MAPK3, PTGS2]
GO:0044321	response to leptin	GO_BP	1.62E-04	1.77E-04	11.111111	3	[CCNA2, CCND1, STAT3]
GO:0031954	positive regulation of protein autophosphorylation	GO_BP	1.62E-04	1.77E-04	11.111111	3	[INS, SRC, VEGFA]
GO:0048645	animal organ formation	GO_BP	1.69E-04	1.85E-04	5.714286	4	[MAP2K1, MAPK1, MAPK3, NOTCH1]
GO:0070265	necrotic cell death	GO_BP	1.79E-04	1.96E-04	5.633803	4	[CASP8, HSP90AA1, TLR4, TP53]
GO:0010518	positive regulation of phospholipase activity	GO_BP	1.79E-04	1.96E-04	5.633803	4	[CCNA2, EGFR, ESR1, PDPK1]
GO:0000186	activation of MAPKK activity	GO_BP	1.79E-04	1.96E-04	5.633803	4	[EGFR, MAP2K1, MAPK1, MAPK3]
GO:0071378	cellular response to growth hormone stimulus	GO_BP	1.81E-04	1.97E-04	10.714286	3	[MYC, PTK2, STAT3]
GO:0030878	thyroid gland development	GO_BP	1.81E-04	1.97E-04	10.714286	3	[MAP2K1, MAPK1, MAPK3]
GO:0010857	calcium-dependent protein kinase activity	GO_BP	1.81E-04	1.97E-04	10.714286	3	[HMGB1, PRKCA, PRKCD]
GO:0010575	positive regulation of vascular endothelial growth factor production	GO_BP	1.81E-04	1.97E-04	10.714286	3	[IL1B, IL6, PTGS2]
GO:0051966	regulation of synaptic transmission, glutamatergic	GO_BP	1.89E-04	2.06E-04	5.5555553	4	[CCL2, CDK5, EGFR, PTGS2]

GO:0042531	positive regulation of tyrosine phosphorylation of STAT protein	GO_BP	1.89E-04	2.06E-04	5.5555553	4	[IGF1, IL6, STAT3, VEGFA]
GO:0009206	purine ribonucleoside triphosphate biosynthetic process	GO_BP	1.89E-04	2.06E-04	5.5555553	4	[MYC, PARP1, PPARA, STAT3]
GO:1903036	positive regulation of response to wounding	GO_BP	1.99E-04	2.17E-04	5.479452	4	[HMGB1, MTOR, NFE2L2, PTK2]
GO:0009145	purine nucleoside triphosphate biosynthetic process	GO_BP	1.99E-04	2.17E-04	5.479452	4	[MYC, PARP1, PPARA, STAT3]
GO:1900078	positive regulation of cellular response to insulin stimulus	GO_BP	2.01E-04	2.19E-04	10.344828	3	[INS, IRS1, SRC]
GO:0050995	negative regulation of lipid catabolic process	GO_BP	2.01E-04	2.19E-04	10.344828	3	[AKT1, IL1B, INS]
GO:0010614	negative regulation of cardiac muscle hypertrophy	GO_BP	2.01E-04	2.19E-04	10.344828	3	[FOXO1, NOTCH1, PPARA]
GO:0001727	lipid kinase activity	GO_BP	2.10E-04	2.28E-04	5.4054055	4	[IRS1, PTK2, RB1, SRC]
GO:0002292	T cell differentiation involved in immune response	GO_BP	2.21E-04	2.40E-04	5.3333335	4	[HMGB1, IL6, MTOR, STAT3]
GO:0035886	vascular associated smooth muscle cell differentiation	GO_BP	2.23E-04	2.41E-04	10	3	[NFATC1, NOTCH1, VEGFA]
GO:0034698	response to gonadotropin	GO_BP	2.23E-04	2.41E-04	10	3	[CCNA2, MYC, NOTCH1]
GO:0002675	positive regulation of acute inflammatory response	GO_BP	2.23E-04	2.41E-04	10	3	[IL1B, IL6, PTGS2]
GO:0000737	DNA catabolic process, endonucleolytic	GO_BP	2.23E-04	2.41E-04	10	3	[CASP3, HMGB1, IL6]
KEGG:03320	PPAR signaling pathway	KEGG	2.33E-	2.52E-04	5.263158	4	[PDPK1, PPARA, PPARG, RXRA]

			04				
GO:0045453	bone resorption	GO_BP	2.33E-04	2.52E-04	5.263158	4	[EGFR, IL6, PRKCA, SRC]
KEGG:05100	Bacterial invasion of epithelial cells	KEGG	2.45E-04	2.65E-04	5.194805	4	[CDH1, FN1, PTK2, SRC]
GO:0045739	positive regulation of DNA repair	GO_BP	2.45E-04	2.65E-04	5.194805	4	[EGFR, H2AX, HMGB1, PARP1]
GO:0042698	ovulation cycle	GO_BP	2.45E-04	2.65E-04	5.194805	4	[CASP3, EGFR, ESR1, SRC]
GO:0042246	tissue regeneration	GO_BP	2.45E-04	2.65E-04	5.194805	4	[CCNB1, EZH2, IGF1, NOTCH1]
GO:0062098	regulation of programmed necrotic cell death	GO_BP	2.46E-04	2.65E-04	9.67742	3	[CASP8, HSP90AA1, TP53]
GO:0051968	positive regulation of synaptic transmission, glutamatergic	GO_BP	2.46E-04	2.65E-04	9.67742	3	[CCL2, EGFR, PTGS2]
GO:0033598	mammary gland epithelial cell proliferation	GO_BP	2.46E-04	2.65E-04	9.67742	3	[CCND1, ESR1, MAPK1]
GO:0014741	negative regulation of muscle hypertrophy	GO_BP	2.46E-04	2.65E-04	9.67742	3	[FOXO1, NOTCH1, PPARA]
GO:0001844	protein insertion into mitochondrial membrane involved in apoptotic signaling pathway	GO_BP	2.46E-04	2.65E-04	9.67742	3	[CASP8, MAPK8, TP53]
GO:0098586	cellular response to virus	GO_BP	2.57E-04	2.77E-04	5.1282053	4	[CHUK, HSP90AA1, IL6, MAPK14]
GO:0006306	DNA methylation	GO_BP	2.57E-04	2.77E-04	5.1282053	4	[EZH2, FOS, MYC, PARP1]
GO:0006305	DNA alkylation	GO_BP	2.57E-04	2.77E-04	5.1282053	4	[EZH2, FOS, MYC, PARP1]
GO:0046332	SMAD binding	GO_MF	2.70E-	2.91E-04	5.063291	4	[FOS, JUN, PARP1, SMAD2]

			04				
GO:2000191	regulation of fatty acid transport	GO_BP	2.71E-04	2.91E-04	9.375	3	[AKT1, IL1B, PPARA]
GO:0071168	protein localization to chromatin	GO_BP	2.71E-04	2.91E-04	9.375	3	[ESR1, EZH2, RB1]
GO:0070884	regulation of calcineurin-NFAT signaling cascade	GO_BP	2.71E-04	2.91E-04	9.375	3	[GSK3B, IGF1, MTOR]
GO:0060259	regulation of feeding behavior	GO_BP	2.71E-04	2.91E-04	9.375	3	[INS, MTOR, STAT3]
GO:0055094	response to lipoprotein particle	GO_BP	2.71E-04	2.91E-04	9.375	3	[AKT1, PPARG, TLR4]
GO:0051123	RNA polymerase II preinitiation complex assembly	GO_BP	2.71E-04	2.91E-04	9.375	3	[ESR1, HMGB1, TP53]
GO:0051059	NF-kappaB binding	GO_MF	2.71E-04	2.91E-04	9.375	3	[GSK3B, NFKBIA, RELA]
GO:0048384	retinoic acid receptor signaling pathway	GO_BP	2.71E-04	2.91E-04	9.375	3	[EZH2, PPARG, RXRA]
GO:0035666	TRIF-dependent toll-like receptor signaling pathway	GO_BP	2.71E-04	2.91E-04	9.375	3	[CASP8, CHUK, TLR4]
GO:0031063	regulation of histone deacetylation	GO_BP	2.71E-04	2.91E-04	9.375	3	[MAPK8, TP53, VEGFA]
GO:0010574	regulation of vascular endothelial growth factor production	GO_BP	2.71E-04	2.91E-04	9.375	3	[IL1B, IL6, PTGS2]
GO:0034121	regulation of toll-like receptor signaling pathway	GO_BP	2.84E-04	3.05E-04	5	4	[ESR1, HMGB1, PDPK1, TLR4]
GO:0031640	killing of cells of other organism	GO_BP	2.84E-04	3.05E-04	5	4	[CASP8, MAPK1, MAPK3, NOS2]
GO:0009201	ribonucleoside triphosphate biosynthetic process	GO_BP	2.84E-04	3.05E-04	5	4	[MYC, PARP1, PPARA, STAT3]

GO:2001240	negative regulation of extrinsic apoptotic signaling pathway in absence of ligand	GO_BP	2.97E-04	3.18E-04	9.090909	3	[AKT1, BCL2L1, IL1B]
GO:1901099	negative regulation of signal transduction in absence of ligand	GO_BP	2.97E-04	3.18E-04	9.090909	3	[AKT1, BCL2L1, IL1B]
GO:0106056	regulation of calcineurin-mediated signaling	GO_BP	2.97E-04	3.18E-04	9.090909	3	[GSK3B, IGF1, MTOR]
GO:0061049	cell growth involved in cardiac muscle cell development	GO_BP	2.97E-04	3.18E-04	9.090909	3	[IGF1, MTOR, PPARA]
GO:0035767	endothelial cell chemotaxis	GO_BP	2.97E-04	3.18E-04	9.090909	3	[HMGB1, NOTCH1, VEGFA]
GO:0019048	modulation by virus of host process	GO_BP	2.97E-04	3.18E-04	9.090909	3	[CASP8, MYC, RXRA]
GO:0010464	regulation of mesenchymal cell proliferation	GO_BP	2.97E-04	3.18E-04	9.090909	3	[MYC, STAT1, VEGFA]
GO:0003301	physiological cardiac muscle hypertrophy	GO_BP	2.97E-04	3.18E-04	9.090909	3	[IGF1, MTOR, PPARA]
GO:0003298	physiological muscle hypertrophy	GO_BP	2.97E-04	3.18E-04	9.090909	3	[IGF1, MTOR, PPARA]
GO:0006635	fatty acid beta-oxidation	GO_BP	3.12E-04	3.34E-04	4.878049	4	[AKT1, IRS1, MTOR, PPARA]
GO:1902692	regulation of neuroblast proliferation	GO_BP	3.25E-04	3.47E-04	8.823529	3	[NOTCH1, TP53, VEGFA]
GO:1900087	positive regulation of G1/S transition of mitotic cell cycle	GO_BP	3.25E-04	3.47E-04	8.823529	3	[AKT1, CCND1, EGFR]
GO:0071402	cellular response to lipoprotein particle stimulus	GO_BP	3.25E-04	3.47E-04	8.823529	3	[AKT1, PPARG, TLR4]
GO:0045648	positive regulation of	GO_BP	3.25E-	3.47E-04	8.823529	3	[MAPK14, STAT1, STAT3]

	erythrocyte differentiation		04				
GO:0043552	positive regulation of phosphatidylinositol 3-kinase activity	GO_BP	3.25E-04	3.47E-04	8.823529	3	[IRS1, PTK2, SRC]
GO:0030262	apoptotic nuclear changes	GO_BP	3.25E-04	3.47E-04	8.823529	3	[CASP3, HMGB1, IL6]
GO:0010039	response to iron ion	GO_BP	3.25E-04	3.47E-04	8.823529	3	[CCNB1, CCND1, HMOX1]
GO:0060193	positive regulation of lipase activity	GO_BP	3.27E-04	3.49E-04	4.8192773	4	[CCNA2, EGFR, ESR1, PDPK1]
GO:0046834	lipid phosphorylation	GO_BP	3.27E-04	3.49E-04	4.8192773	4	[IRS1, PTK2, RB1, SRC]
GO:0061418	regulation of transcription from RNA polymerase II promoter in response to hypoxia	GO_BP	3.42E-04	3.65E-04	4.7619047	4	[NFE2L2, NOTCH1, TP53, VEGFA]
GO:0045445	myoblast differentiation	GO_BP	3.42E-04	3.65E-04	4.7619047	4	[IGF1, MAPK14, NOTCH1, RB1]
GO:0010517	regulation of phospholipase activity	GO_BP	3.42E-04	3.65E-04	4.7619047	4	[CCNA2, EGFR, ESR1, PDPK1]
GO:0090183	regulation of kidney development	GO_BP	3.54E-04	3.77E-04	8.571428	3	[MYC, STAT1, VEGFA]
GO:0048873	homeostasis of number of cells within a tissue	GO_BP	3.54E-04	3.77E-04	8.571428	3	[KRAS, NOS3, NOTCH1]
GO:0044068	modulation by symbiont of host cellular process	GO_BP	3.54E-04	3.77E-04	8.571428	3	[CASP8, MAPK1, MAPK3]
GO:0043537	negative regulation of blood vessel endothelial cell migration	GO_BP	3.54E-04	3.77E-04	8.571428	3	[HMGB1, NOTCH1, PPARG]
GO:0006308	DNA catabolic process	GO_BP	3.54E-04	3.77E-04	8.571428	3	[CASP3, HMGB1, IL6]

GO:0004407	histone deacetylase activity	GO_BP	3.54E-04	3.77E-04	8.571428	3	[MAPK8, TP53, VEGFA]
GO:0060395	SMAD protein signal transduction	GO_BP	3.58E-04	3.81E-04	4.7058825	4	[FOS, JUN, PARP1, SMAD2]
GO:0043367	CD4-positive, alpha-beta T cell differentiation	GO_BP	3.58E-04	3.81E-04	4.7058825	4	[HMGB1, IL6, MTOR, STAT3]
GO:0042509	regulation of tyrosine phosphorylation of STAT protein	GO_BP	3.58E-04	3.81E-04	4.7058825	4	[IGF1, IL6, STAT3, VEGFA]
GO:0034198	cellular response to amino acid starvation	GO_BP	3.58E-04	3.81E-04	4.7058825	4	[MAPK1, MAPK3, MAPK8, MTOR]
GO:0032092	positive regulation of protein binding	GO_BP	3.58E-04	3.81E-04	4.7058825	4	[APP, CDK5, GSK3B, MMP9]
GO:0030512	negative regulation of transforming growth factor beta receptor signaling pathway	GO_BP	3.58E-04	3.81E-04	4.7058825	4	[PDPK1, PPARA, SMAD2, TP53]
GO:2000144	positive regulation of DNA-templated transcription, initiation	GO_BP	3.85E-04	4.09E-04	8.333333	3	[ESR1, JUN, TP53]
GO:0045746	negative regulation of Notch signaling pathway	GO_BP	3.85E-04	4.09E-04	8.333333	3	[AKT1, EGFR, NFKBIA]
GO:0034311	diol metabolic process	GO_BP	3.85E-04	4.09E-04	8.333333	3	[AKT1, HSP90AA1, NOS3]
GO:0033558	protein deacetylase activity	GO_BP	3.85E-04	4.09E-04	8.333333	3	[MAPK8, TP53, VEGFA]
GO:0010758	regulation of macrophage chemotaxis	GO_BP	3.85E-04	4.09E-04	8.333333	3	[MAPK1, MAPK3, PTK2]
GO:0010573	vascular endothelial growth factor production	GO_BP	3.85E-04	4.09E-04	8.333333	3	[IL1B, IL6, PTGS2]
GO:0006921	cellular component disassembly involved in	GO_BP	3.85E-04	4.09E-04	8.333333	3	[CASP3, HMGB1, IL6]

	execution phase of apoptosis						
GO:0002756	MyD88-independent toll-like receptor signaling pathway	GO_BP	3.85E-04	4.09E-04	8.333333	3	[CASP8, CHUK, TLR4]
GO:1990928	response to amino acid starvation	GO_BP	4.08E-04	4.33E-04	4.5454545	4	[MAPK1, MAPK3, MAPK8, MTOR]
GO:0046330	positive regulation of JNK cascade	GO_BP	4.08E-04	4.33E-04	4.5454545	4	[APP, HMGB1, IL1B, TLR4]
GO:0009205	purine ribonucleoside triphosphate metabolic process	GO_BP	4.08E-04	4.33E-04	4.5454545	4	[MYC, PARP1, PPARA, STAT3]
GO:0002709	regulation of T cell mediated immunity	GO_BP	4.08E-04	4.33E-04	4.5454545	4	[AHR, HMGB1, IL1B, IL6]
KEGG:04350	TGF-beta signaling pathway	KEGG	5.24E-04	5.52E-04	4.255319	4	[MAPK1, MAPK3, MYC, SMAD2]
GO:0010874	regulation of cholesterol efflux	GO_BP	4.18E-04	4.43E-04	8.1081085	3	[NFKBIA, PPARG, RXRA]
GO:0007260	tyrosine phosphorylation of STAT protein	GO_BP	4.26E-04	4.51E-04	4.494382	4	[IGF1, IL6, STAT3, VEGFA]
GO:0051057	positive regulation of small GTPase mediated signal transduction	GO_BP	4.45E-04	4.70E-04	4.4444447	4	[IGF1, KRAS, NOTCH1, SRC]
GO:0016811	hydrolase activity, acting on carbon-nitrogen (but not peptide) bonds, in linear amides	GO_MF	4.45E-04	4.70E-04	4.4444447	4	[CAT, MAPK8, TP53, VEGFA]
GO:0090218	positive regulation of lipid kinase activity	GO_BP	4.52E-04	4.78E-04	7.894737	3	[IRS1, PTK2, SRC]
GO:0045907	positive regulation of vasoconstriction	GO_BP	4.52E-04	4.78E-04	7.894737	3	[AKT1, EGFR, PTGS2]
GO:0045601	regulation of endothelial cell	GO_BP	4.52E-	4.78E-04	7.894737	3	[IL1B, NOTCH1, VEGFA]

	differentiation		04				
GO:0035633	maintenance of blood-brain barrier	GO_BP	4.52E-04	4.78E-04	7.894737	3	[IL6, PTGS2, VEGFA]
GO:0032212	positive regulation of telomere maintenance via telomerase	GO_BP	4.52E-04	4.78E-04	7.894737	3	[ATM, MAPK1, MAPK3]
GO:0032205	negative regulation of telomere maintenance	GO_BP	4.52E-04	4.78E-04	7.894737	3	[ATM, PARP1, SRC]
GO:0010665	regulation of cardiac muscle cell apoptotic process	GO_BP	4.52E-04	4.78E-04	7.894737	3	[NFE2L2, PDPK1, TP53]
GO:0009142	nucleoside triphosphate biosynthetic process	GO_BP	4.64E-04	4.89E-04	4.3956046	4	[MYC, PARP1, PPARA, STAT3]
GO:0032728	positive regulation of interferon-beta production	GO_BP	4.89E-04	5.15E-04	7.6923075	3	[HMGB1, HSP90AA1, TLR4]
KEGG:04713	Circadian entrainment	KEGG	5.91E-04	6.18E-04	4.123711	4	[FOS, MAPK1, MAPK3, PRKCA]
GO:0048013	ephrin receptor signaling pathway	GO_BP	5.24E-04	5.52E-04	4.255319	4	[MMP2, MMP9, PTK2, SRC]
GO:0045638	negative regulation of myeloid cell differentiation	GO_BP	5.24E-04	5.52E-04	4.255319	4	[CDK6, MYC, NFKBIA, TLR4]
GO:0035249	synaptic transmission, glutamatergic	GO_BP	5.24E-04	5.52E-04	4.255319	4	[CCL2, CDK5, EGFR, PTGS2]
GO:0009144	purine nucleoside triphosphate metabolic process	GO_BP	5.24E-04	5.52E-04	4.255319	4	[MYC, PARP1, PPARA, STAT3]
GO:0070423	nucleotide-binding oligomerization domain containing signaling pathway	GO_BP	5.27E-04	5.54E-04	7.5	3	[CASP8, NFKBIA, TLR4]
GO:0043368	positive T cell selection	GO_BP	5.27E-04	5.54E-04	7.5	3	[IL6, MTOR, STAT3]

GO:0042805	actinin binding	GO_MF	5.27E-04	5.54E-04	7.5	3	[NFKB1, PPARG, RELA]
GO:0032376	positive regulation of cholesterol transport	GO_BP	5.27E-04	5.54E-04	7.5	3	[NFKBIA, PPARG, RXRA]
GO:0032373	positive regulation of sterol transport	GO_BP	5.27E-04	5.54E-04	7.5	3	[NFKBIA, PPARG, RXRA]
GO:0010659	cardiac muscle cell apoptotic process	GO_BP	5.27E-04	5.54E-04	7.5	3	[NFE2L2, PDPK1, TP53]
GO:0060333	interferon-gamma-mediated signaling pathway	GO_BP	5.46E-04	5.73E-04	4.2105265	4	[PPARG, PRKCD, STAT1, TP53]
GO:1904358	positive regulation of telomere maintenance via telomere lengthening	GO_BP	5.67E-04	5.94E-04	7.3170733	3	[ATM, MAPK1, MAPK3]
GO:1903670	regulation of sprouting angiogenesis	GO_BP	5.67E-04	5.94E-04	7.3170733	3	[HMGB1, PDPK1, VEGFA]
GO:1902003	regulation of amyloid-beta formation	GO_BP	5.67E-04	5.94E-04	7.3170733	3	[CASP3, IGF1, RELA]
GO:0098761	cellular response to interleukin-7	GO_BP	5.67E-04	5.94E-04	7.3170733	3	[HMGB1, IRS1, STAT3]
GO:0098760	response to interleukin-7	GO_BP	5.67E-04	5.94E-04	7.3170733	3	[HMGB1, IRS1, STAT3]
GO:0051154	negative regulation of striated muscle cell differentiation	GO_BP	5.67E-04	5.94E-04	7.3170733	3	[EZH2, NOTCH1, PPARA]
GO:0048713	regulation of oligodendrocyte differentiation	GO_BP	5.67E-04	5.94E-04	7.3170733	3	[MTOR, NOTCH1, PPARG]
GO:0035872	nucleotide-binding domain, leucine rich repeat containing receptor signaling pathway	GO_BP	5.67E-04	5.94E-04	7.3170733	3	[CASP8, NFKBIA, TLR4]
GO:0010662	regulation of striated muscle	GO_BP	5.67E-	5.94E-04	7.3170733	3	[NFE2L2, PDPK1, TP53]

	cell apoptotic process		04				
GO:0060291	long-term synaptic potentiation	GO_BP	5.68E-04	5.95E-04	4.1666665	4	[APP, GSK3B, INS, MAPK1]
GO:0048041	focal adhesion assembly	GO_BP	5.68E-04	5.95E-04	4.1666665	4	[PDPK1, PTK2, SRC, VEGFA]
GO:0008013	beta-catenin binding	GO_MF	5.68E-04	5.95E-04	4.1666665	4	[CDH1, ESR1, FOXO1, GSK3B]
KEGG:05143	African trypanosomiasis	KEGG	4.18E-04	4.43E-04	8.1081085	3	[IL1B, IL6, PRKCA]
GO:2001251	negative regulation of chromosome organization	GO_BP	5.91E-04	6.18E-04	4.123711	4	[ATM, CCNB1, PARP1, SRC]
GO:1903321	negative regulation of protein modification by small protein conjugation or removal	GO_BP	5.91E-04	6.18E-04	4.123711	4	[AKT1, CDK5, MTOR, RELA]
GO:0070664	negative regulation of leukocyte proliferation	GO_BP	5.91E-04	6.18E-04	4.123711	4	[ATM, CASP3, ERBB2, GSTP1]
GO:0009199	ribonucleoside triphosphate metabolic process	GO_BP	5.91E-04	6.18E-04	4.123711	4	[MYC, PARP1, PPARA, STAT3]
GO:2001239	regulation of extrinsic apoptotic signaling pathway in absence of ligand	GO_BP	6.09E-04	6.36E-04	7.142857	3	[AKT1, BCL2L1, IL1B]
GO:0071312	cellular response to alkaloid	GO_BP	6.09E-04	6.36E-04	7.142857	3	[BCL2L1, CASP3, CCNA2]
GO:0042558	pteridine-containing compound metabolic process	GO_BP	6.09E-04	6.36E-04	7.142857	3	[AKT1, HSP90AA1, NOS3]
GO:0032094	response to food	GO_BP	6.09E-04	6.36E-04	7.142857	3	[AKT1, MTOR, PPARA]
GO:0030513	positive regulation of BMP signaling pathway	GO_BP	6.09E-04	6.36E-04	7.142857	3	[CCNA2, NOTCH1, SMAD2]

GO:0010939	regulation of necrotic cell death	GO_BP	6.09E-04	6.36E-04	7.142857	3	[CASP8, HSP90AA1, TP53]
GO:0007076	mitotic chromosome condensation	GO_BP	6.09E-04	6.36E-04	7.142857	3	[CCNB1, CDK1, RB1]
GO:1901184	regulation of ERBB signaling pathway	GO_BP	6.14E-04	6.40E-04	4.0816326	4	[AKT1, EGFR, ERBB2, MMP9]
GO:0048477	oogenesis	GO_BP	6.14E-04	6.40E-04	4.0816326	4	[AURKA, CCNB1, IGF1, SRC]
GO:0034502	protein localization to chromosome	GO_BP	6.38E-04	6.65E-04	4.040404	4	[CDK1, ESR1, EZH2, RB1]
GO:0045740	positive regulation of DNA replication	GO_BP	6.53E-04	6.79E-04	6.976744	3	[CDK1, EGFR, JUN]
GO:0042056	chemoattractant activity	GO_BP	6.53E-04	6.79E-04	6.976744	3	[HMGB1, IL6, VEGFA]
GO:0030851	granulocyte differentiation	GO_BP	6.53E-04	6.79E-04	6.976744	3	[CDK2, CDK4, RXRA]
GO:0014911	positive regulation of smooth muscle cell migration	GO_BP	6.53E-04	6.79E-04	6.976744	3	[IGF1, MYC, SRC]
GO:0010658	striated muscle cell apoptotic process	GO_BP	6.53E-04	6.79E-04	6.976744	3	[NFE2L2, PDPK1, TP53]
GO:0001937	negative regulation of endothelial cell proliferation	GO_BP	6.53E-04	6.79E-04	6.976744	3	[CCL2, PPARG, STAT1]
GO:0050764	regulation of phagocytosis	GO_BP	6.62E-04	6.89E-04	4	4	[CCL2, HMGB1, IL1B, PPARG]
GO:0045185	maintenance of protein location	GO_BP	6.62E-04	6.89E-04	4	4	[AKT1, CDK5, KEAP1, NFKBIA]
GO:0030516	regulation of axon extension	GO_BP	6.62E-04	6.89E-04	4	4	[CDK5, FN1, GSK3B, VEGFA]
GO:0015908	fatty acid transport	GO_BP	6.62E-04	6.89E-04	4	4	[AKT1, IL1B, PPARA, PPARG]

GO:2000772	regulation of cellular senescence	GO_BP	6.98E-04	7.25E-04	6.818182	3	[CDK6, KRAS, TP53]
GO:1900744	regulation of p38MAPK cascade	GO_BP	6.98E-04	7.25E-04	6.818182	3	[IL1B, IL6, VEGFA]
GO:0035987	endodermal cell differentiation	GO_BP	6.98E-04	7.25E-04	6.818182	3	[FN1, MMP2, MMP9]
GO:0032570	response to progesterone	GO_BP	6.98E-04	7.25E-04	6.818182	3	[FOS, RELA, SRC]
GO:2000279	negative regulation of DNA biosynthetic process	GO_BP	7.46E-04	7.74E-04	6.6666665	3	[PPARG, SRC, TP53]
GO:0048020	CCR chemokine receptor binding	GO_MF	7.46E-04	7.74E-04	6.6666665	3	[CCL2, STAT1, STAT3]
GO:0046688	response to copper ion	GO_BP	7.46E-04	7.74E-04	6.6666665	3	[APP, CDK1, NFE2L2]
GO:0042169	SH2 domain binding	GO_MF	7.46E-04	7.74E-04	6.6666665	3	[IRS1, PTK2, SRC]
GO:0014888	striated muscle adaptation	GO_BP	7.46E-04	7.74E-04	6.6666665	3	[EZH2, FOXO1, MTOR]
GO:0046006	regulation of activated T cell proliferation	GO_BP	7.96E-04	8.24E-04	6.521739	3	[CASP3, HMGB1, IGF1]
GO:0045668	negative regulation of osteoblast differentiation	GO_BP	7.96E-04	8.24E-04	6.521739	3	[CDK6, NOTCH1, PPARG]
GO:0010463	mesenchymal cell proliferation	GO_BP	7.96E-04	8.24E-04	6.521739	3	[MYC, STAT1, VEGFA]
GO:0001784	phosphotyrosine residue binding	GO_MF	7.96E-04	8.24E-04	6.521739	3	[IRS1, MAPK1, MAPK3]
GO:1902991	regulation of amyloid precursor protein catabolic process	GO_BP	8.48E-04	8.77E-04	6.382979	3	[CASP3, IGF1, RELA]
GO:0070897	transcription preinitiation complex assembly	GO_BP	8.48E-04	8.77E-04	6.382979	3	[ESR1, HMGB1, TP53]

GO:0051155	positive regulation of striated muscle cell differentiation	GO_BP	8.48E-04	8.77E-04	6.382979	3	[IGF1, MAPK14, MTOR]
GO:0042551	neuron maturation	GO_BP	8.48E-04	8.77E-04	6.382979	3	[APP, MTOR, RB1]
GO:0033146	regulation of intracellular estrogen receptor signaling pathway	GO_BP	8.48E-04	8.77E-04	6.382979	3	[ESR1, PARP1, SRC]
GO:0010830	regulation of myotube differentiation	GO_BP	8.48E-04	8.77E-04	6.382979	3	[MAPK14, MTOR, NOTCH1]
GO:0004520	endodeoxyribonuclease activity	GO_BP	8.48E-04	8.77E-04	6.382979	3	[AKT1, HMGB1, PRKCD]
GO:1905521	regulation of macrophage migration	GO_BP	9.02E-04	9.32E-04	6.25	3	[MAPK1, MAPK3, PTK2]
GO:1900271	regulation of long-term synaptic potentiation	GO_BP	9.02E-04	9.32E-04	6.25	3	[APP, GSK3B, INS]
GO:0045454	cell redox homeostasis	GO_BP	9.02E-04	9.32E-04	6.25	3	[NFE2L2, NOS2, NOS3]
GO:0043330	response to exogenous dsRNA	GO_BP	9.02E-04	9.32E-04	6.25	3	[MAPK1, MAPK3, NFKBIA]
GO:0120163	negative regulation of cold-induced thermogenesis	GO_BP	9.58E-04	9.88E-04	6.122449	3	[NOTCH1, RB1, TLR4]
GO:0048599	oocyte development	GO_BP	9.58E-04	9.88E-04	6.122449	3	[AURKA, CCNB1, IGF1]
GO:0045776	negative regulation of blood pressure	GO_BP	9.58E-04	9.88E-04	6.122449	3	[NOS2, NOS3, PPARA]
GO:0031952	regulation of protein autophosphorylation	GO_BP	9.58E-04	9.88E-04	6.122449	3	[INS, SRC, VEGFA]
GO:0022602	ovulation cycle process	GO_BP	9.58E-04	9.88E-04	6.122449	3	[CASP3, ESR1, SRC]

Table S5. GO Terms and KEGG pathways associated with the 64 identified key targets

ID	Term	Ontology Source	Term PValue	Term PValue Corrected with Benjamini-Hochberg	% Associated Genes	Number of Genes	Associated Genes Found
KEGG:05200	Pathways in cancer	KEGG	2.81E-54	1.64E-51	8.29	44.00	[AGT, AKT1, BCL2L1, CASP3, CASP9, CCNA2, CCND1, CDH1, CDK2, CDK4, CDK6, EGFR, ERBB2, ESR1, ESR2, FN1, FOS, FOXO1, GSK3B, GSTP1, HMOX1, HSP90AA1, IGF1, IL6, JUN, KEAP1, MAP2K1, MAPK1, MAPK3, MAPK8, MAPK9, MMP2, MMP9, MTOR, NFE2L2, NFKB1, NQO1, PPARG, PTGS2, PTK2, RELA, SMAD2, STAT3, TP53]
KEGG:05417	Lipid and atherosclerosis	KEGG	2.42E-40	7.07E-38	13.49	29.00	[AKT1, BCL2L1, CASP3, CASP9, CYP1A1, FOS, GSK3B, HSP90AA1, HSPA8, IL1B, IL6, JUN, MAPK1, MAPK14, MAPK3, MAPK8, MAPK9, MMP3, MMP9, NFE2L2, NFKB1, NOS3, PPARG, PTK2, RELA, SRC, STAT3, TLR4, TP53]
GO:0062197	cellular response to chemical stress	GO_BP	3.74E-35	7.28E-33	8.24	30.00	[AKT1, CASP3, CDK1, EGFR, FOS, FOXO1, HMOX1, IL6, INS, JUN, KEAP1, MAPK1, MAPK3, MAPK8, MAPK9, MCL1, MMP2, MMP3, MMP9, MTOR, NFE2L2, NOS3, NQO1, PARP1, PPARG, PTGS2, RELA, SRC, TLR4, TP53]
GO:1901699	cellular response to nitrogen compound	GO_BP	1.16E-34	1.69E-32	4.88	36.00	[AGT, AKT1, APP, BCL2L1, CASP3, CCNA2, CDH1, CDK2, CDK4, CDK5, CHEK1, EGFR, FOS, FOXO1, GSK3B, IGF1, IL1B, INS, IRS1, JUN, MAPK1, MAPK3, MMP2, MMP3, MTOR, NFE2L2, NFKB1, PARP1, PPARG, PTGS2, PTK2, RELA, SRC, STAT3, TLR4, TP53]
KEGG:05215	Prostate cancer	KEGG	1.47E-33	1.72E-31	21.65	21.00	[AKT1, CASP9, CCND1, CDK2, EGFR, ERBB2, FOXO1, GSK3B, GSTP1, HSP90AA1, IGF1, INS, MAP2K1, MAPK1, MAPK3, MMP3, MMP9, MTOR, NFKB1, RELA, TP53]

KEGG:04933	AGE-RAGE signaling pathway in diabetic complications	KEGG	2.99E-33	2.91E-31	21.00	21.00	[AGT, AKT1, CASP3, CCND1, CDK4, FN1, FOXO1, IL1B, IL6, JUN, MAPK1, MAPK14, MAPK3, MAPK8, MAPK9, MMP2, NFKB1, NOS3, RELA, SMAD2, STAT3]
GO:0071417	cellular response to organonitrogen compound	GO_BP	6.83E-33	5.70E-31	5.01	34.00	[AGT, AKT1, APP, BCL2L1, CASP3, CCNA2, CDH1, CDK4, CDK5, CHEK1, EGFR, FOS, FOXO1, GSK3B, IGF1, IL1B, INS, IRS1, JUN, MAPK1, MAPK3, MMP2, MTOR, NFE2L2, NFKB1, PARP1, PPARG, PTGS2, PTK2, RELA, SRC, STAT3, TLR4, TP53]
KEGG:04151	PI3K-Akt signaling pathway	KEGG	3.80E-32	2.77E-30	7.91	28.00	[AKT1, BCL2L1, CASP9, CCND1, CDK2, CDK4, CDK6, EGFR, ERBB2, FN1, GSK3B, HSP90AA1, IGF1, IL6, INS, IRS1, KDR, MAP2K1, MAPK1, MAPK3, MCL1, MTOR, NFKB1, NOS3, PTK2, RELA, TLR4, TP53]
GO:0006979	response to oxidative stress	GO_BP	5.27E-32	3.42E-30	6.49	30.00	[AKT1, APP, CASP3, CDK1, EGFR, FOS, FOXO1, GSTP1, HMOX1, IL6, INS, JUN, KEAP1, MAPK1, MAPK3, MAPK8, MAPK9, MCL1, MMP2, MMP3, MMP9, NFE2L2, NOS3, NQO1, PARP1, PTGS2, RELA, SRC, TLR4, TP53]
KEGG:05418	Fluid shear stress and atherosclerosis	KEGG	6.46E-32	3.77E-30	15.83	22.00	[AKT1, FOS, GSTP1, HMOX1, HSP90AA1, IL1B, JUN, KDR, KEAP1, MAPK14, MAPK8, MAPK9, MMP2, MMP9, NFE2L2, NFKB1, NOS3, NQO1, PTK2, RELA, SRC, TP53]
KEGG:05212	Pancreatic cancer	KEGG	1.12E-31	5.94E-30	25.00	19.00	[AKT1, BCL2L1, CASP9, CCND1, CDK4, CDK6, EGFR, ERBB2, MAP2K1, MAPK1, MAPK3, MAPK8, MAPK9, MTOR, NFKB1, RELA, SMAD2, STAT3, TP53]
GO:0034599	cellular response to oxidative stress	GO_BP	1.36E-30	6.63E-29	8.52	26.00	[AKT1, CDK1, EGFR, FOS, FOXO1, HMOX1, IL6, INS, JUN, KEAP1, MAPK1, MAPK3, MAPK8, MAPK9, MCL1, MMP2, MMP3, MMP9, NFE2L2, NOS3, NQO1, PARP1, RELA, SRC, TLR4, TP53]
KEGG:05167	Kaposi sarcoma-associated herpesvirus infection	KEGG	2.41E-30	1.08E-28	11.86	23.00	[AKT1, CASP3, CASP9, CCND1, CDK4, CDK6, FOS, GSK3B, IL6, JUN, MAP2K1, MAPK1, MAPK14, MAPK3, MAPK8, MAPK9, MTOR, NFKB1, PTGS2, RELA, SRC, STAT3, TP53]

KEGG:05162	Measles	KEGG	5.42E-30	2.26E-28	15.11	21.00	[AKT1, BCL2L1, CASP3, CASP9, CCND1, CDK2, CDK4, CDK6, FOS, GSK3B, HSPA8, IL1B, IL6, JUN, MAPK8, MAPK9, NFKB1, RELA, STAT3, TLR4, TP53]
GO:0010035	response to inorganic substance	GO_BP	7.26E-30	2.83E-28	5.08	31.00	[AKT1, APP, CASP3, CASP9, CCNA2, CCND1, CDH1, CDK1, CDK2, CYP1A1, EGFR, FOS, FOXO1, HMOX1, IL6, JUN, KDR, MAPK1, MAPK3, MAPK8, MAPK9, MMP2, MMP3, MMP9, NFE2L2, NOS3, NQO1, PARP1, PTGS2, RELA, SRC]
GO:1901652	response to peptide	GO_BP	7.22E-29	2.64E-27	5.54	29.00	[AGT, AKT1, APP, CCNA2, CDK4, CDK5, FOS, FOXO1, GSK3B, IGF1, IL1B, INS, IRS1, MAPK14, MMP2, MMP3, MMP9, MTOR, NFE2L2, NFKB1, PARP1, PPARG, PTGS2, PTK2, RELA, SRC, STAT3, TLR4, TP53]
KEGG:05161	Hepatitis B	KEGG	1.63E-28	5.59E-27	12.96	21.00	[AKT1, CASP3, CASP9, CCNA2, CDK2, FOS, IL6, JUN, MAP2K1, MAPK1, MAPK14, MAPK3, MAPK8, MAPK9, MMP9, NFKB1, RELA, SRC, STAT3, TLR4, TP53]
KEGG:04917	Prolactin signaling pathway	KEGG	4.06E-28	1.32E-26	24.29	17.00	[AKT1, CCND1, ESR1, ESR2, FOS, GSK3B, INS, MAP2K1, MAPK1, MAPK14, MAPK3, MAPK8, MAPK9, NFKB1, RELA, SRC, STAT3]
KEGG:05205	Proteoglycans in cancer	KEGG	5.22E-28	1.60E-26	10.73	22.00	[AKT1, CASP3, CCND1, EGFR, ERBB2, ESR1, FN1, IGF1, KDR, MAP2K1, MAPK1, MAPK14, MAPK3, MMP2, MMP9, MTOR, PTK2, SMAD2, SRC, STAT3, TLR4, TP53]
KEGG:05163	Human cytomegalovirus infection	KEGG	4.32E-27	1.26E-25	9.78	22.00	[AKT1, CASP3, CASP9, CCND1, CDK4, CDK6, EGFR, GSK3B, IL1B, IL6, MAP2K1, MAPK1, MAPK14, MAPK3, MTOR, NFKB1, PTGS2, PTK2, RELA, SRC, STAT3, TP53]
KEGG:04218	Cellular senescence	KEGG	4.79E-27	1.33E-25	12.82	20.00	[AKT1, CCNA2, CCNB1, CCND1, CDK1, CDK2, CDK4, CDK6, CHEK1, FOXO1, IL6, MAP2K1, MAPK1, MAPK14, MAPK3, MTOR, NFKB1, RELA, SMAD2, TP53]

KEGG:04657	IL-17 signaling pathway	KEGG	1.03E-25	2.73E-24	18.09	17.00	[CASP3, FOS, GSK3B, HSP90AA1, IL1B, IL6, JUN, MAPK1, MAPK14, MAPK3, MAPK8, MAPK9, MMP3, MMP9, NFKB1, PTGS2, RELA]
GO:0071241	cellular response to inorganic substance	GO_BP	1.30E-24	3.29E-23	8.57	21.00	[AKT1, APP, CCNA2, CDH1, CDK2, CYP1A1, EGFR, FOS, FOXO1, HMOX1, JUN, MAPK1, MAPK3, MAPK8, MAPK9, MMP3, MMP9, NFE2L2, NQO1, PARP1, PTGS2]
KEGG:05135	Yersinia infection	KEGG	1.51E-24	3.67E-23	13.14	18.00	[AKT1, FN1, FOS, GSK3B, IL1B, IL6, JUN, MAP2K1, MAPK1, MAPK14, MAPK3, MAPK8, MAPK9, NFKB1, PTK2, RELA, SRC, TLR4]
KEGG:05210	Colorectal cancer	KEGG	1.94E-24	4.53E-23	18.60	16.00	[AKT1, CASP3, CASP9, CCND1, EGFR, FOS, GSK3B, JUN, MAP2K1, MAPK1, MAPK3, MAPK8, MAPK9, MTOR, SMAD2, TP53]
KEGG:04668	TNF signaling pathway	KEGG	2.52E-24	5.65E-23	15.18	17.00	[AKT1, CASP3, FOS, IL1B, IL6, JUN, MAP2K1, MAPK1, MAPK14, MAPK3, MAPK8, MAPK9, MMP3, MMP9, NFKB1, PTGS2, RELA]
KEGG:05170	Human immunodeficiency virus 1 infection	KEGG	2.77E-24	5.99E-23	9.43	20.00	[AKT1, BCL2L1, CASP3, CASP9, CCNB1, CDK1, CHEK1, FOS, JUN, MAP2K1, MAPK1, MAPK14, MAPK3, MAPK8, MAPK9, MTOR, NFKB1, PTK2, RELA, TLR4]
GO:0000302	response to reactive oxygen species	GO_BP	5.91E-24	1.23E-22	9.09	20.00	[AKT1, CASP3, CDK1, EGFR, FOS, GSTP1, HMOX1, IL6, JUN, MAPK1, MAPK3, MAPK8, MAPK9, MMP2, MMP9, NFE2L2, NOS3, NQO1, RELA, SRC]
GO:0030335	positive regulation of cell migration	GO_BP	1.21E-23	2.43E-22	4.61	26.00	[AGT, AKT1, APP, CCNA2, EGFR, FN1, HMOX1, IGF1, IL1B, IL6, INS, JUN, KDR, MAPK1, MAPK14, MAPK3, MMP2, MMP9, MTOR, NFE2L2, NOS3, PTGS2, PTK2, SRC, STAT3, TLR4]
GO:1901653	cellular response to peptide	GO_BP	2.57E-23	4.99E-22	5.94	23.00	[AGT, AKT1, APP, CCNA2, CDK4, CDK5, FOS, FOXO1, GSK3B, IGF1, IL1B, INS, IRS1, NFE2L2, NFKB1, PARP1, PPARG, PTK2, RELA, SRC, STAT3, TLR4, TP53]
KEGG:04926	Relaxin signaling pathway	KEGG	3.19E-23	6.01E-22	13.18	17.00	[AKT1, EGFR, FOS, JUN, MAP2K1, MAPK1, MAPK14, MAPK3, MAPK8, MAPK9, MMP2,

							MMP9, NFKB1, NOS3, RELA, SMAD2, SRC]
GO:2000147	positive regulation of cell motility	GO_BP	3.49E-23	6.37E-22	4.42	26.00	[AGT, AKT1, APP, CCNA2, EGFR, FN1, HMOX1, IGF1, IL1B, IL6, INS, JUN, KDR, MAPK1, MAPK14, MAPK3, MMP2, MMP9, MTOR, NFE2L2, NOS3, PTGS2, PTK2, SRC, STAT3, TLR4]
KEGG:04068	FoxO signaling pathway	KEGG	4.20E-23	7.43E-22	12.98	17.00	[AKT1, CCNB1, CCND1, CDK2, EGFR, FOXO1, IGF1, IL6, INS, IRS1, MAP2K1, MAPK1, MAPK14, MAPK3, MAPK8, MAPK9, STAT3]
KEGG:05225	Hepatocellular carcinoma	KEGG	6.92E-23	1.19E-21	10.71	18.00	[AKT1, BCL2L1, CCND1, CDK4, CDK6, EGFR, GSK3B, GSTP1, HMOX1, KEAP1, MAP2K1, MAPK1, MAPK3, MTOR, NFE2L2, NQO1, SMAD2, TP53]
GO:0040017	positive regulation of locomotion	GO_BP	7.20E-23	1.20E-21	4.30	26.00	[AGT, AKT1, APP, CCNA2, EGFR, FN1, HMOX1, IGF1, IL1B, IL6, INS, JUN, KDR, MAPK1, MAPK14, MAPK3, MMP2, MMP9, MTOR, NFE2L2, NOS3, PTGS2, PTK2, SRC, STAT3, TLR4]
KEGG:04066	HIF-1 signaling pathway	KEGG	1.13E-22	1.84E-21	14.68	16.00	[AKT1, EGFR, ERBB2, HMOX1, IGF1, IL6, INS, MAP2K1, MAPK1, MAPK3, MTOR, NFKB1, NOS3, RELA, STAT3, TLR4]
KEGG:05166	Human T-cell leukemia virus 1 infection	KEGG	3.00E-22	4.74E-21	8.56	19.00	[AKT1, BCL2L1, CCNA2, CCND1, CDK2, CDK4, CHEK1, FOS, IL6, JUN, MAP2K1, MAPK1, MAPK3, MAPK8, MAPK9, NFKB1, RELA, SMAD2, TP53]
KEGG:05208	Chemical carcinogenesis	KEGG	3.27E-22	4.90E-21	8.52	19.00	[AKT1, CYP1A1, EGFR, FOS, HMOX1, JUN, KEAP1, MAP2K1, MAPK1, MAPK14, MAPK3, MAPK8, MAPK9, NFE2L2, NFKB1, NQO1, PTK2, RELA, SRC]
KEGG:05224	Breast cancer	KEGG	3.24E-22	4.99E-21	11.56	17.00	[AKT1, CCND1, CDK4, CDK6, EGFR, ERBB2, ESR1, ESR2, FOS, GSK3B, IGF1, JUN, MAP2K1, MAPK1, MAPK3, MTOR, TP53]
GO:0034614	cellular response to reactive oxygen species	GO_BP	1.61E-21	2.35E-20	10.56	17.00	[AKT1, CDK1, EGFR, FOS, IL6, JUN, MAPK1, MAPK3, MAPK8, MAPK9, MMP2, MMP9, NFE2L2, NOS3, NQO1, RELA, SRC]

KEGG:04010	MAPK signaling pathway	KEGG	2.09E-21	2.98E-20	6.80	20.00	[AKT1, CASP3, EGFR, ERBB2, FOS, HSPA8, IGF1, IL1B, INS, JUN, KDR, MAP2K1, MAPK1, MAPK14, MAPK3, MAPK8, MAPK9, NFKB1, RELA, TP53]
KEGG:05131	Shigellosis	KEGG	2.34E-21	3.26E-20	7.69	19.00	[AKT1, BCL2L1, EGFR, FOXO1, GSK3B, IL1B, JUN, MAPK1, MAPK14, MAPK3, MAPK8, MAPK9, MTOR, NFKB1, PTK2, RELA, SRC, TLR4, TP53]
KEGG:04914	Progesterone-mediated oocyte maturation	KEGG	2.67E-21	3.62E-20	14.71	15.00	[AKT1, AURKA, CCNA2, CCNB1, CDK1, CDK2, HSP90AA1, IGF1, INS, MAP2K1, MAPK1, MAPK14, MAPK3, MAPK8, MAPK9]
GO:0009611	response to wounding	GO_BP	2.73E-21	3.62E-20	4.04	25.00	[AURKA, CASP3, CCNA2, CDK1, CDK5, CYP1A1, ERBB2, FN1, HMOX1, IGF1, IL6, INS, JUN, KDR, MAP2K1, MAPK14, MMP2, MTOR, NFE2L2, NOS3, PPARG, PTK2, SMAD2, SRC, TLR4]
GO:0071396	cellular response to lipid	GO_BP	3.07E-21	3.98E-20	4.02	25.00	[AKT1, CASP9, CCNA2, CDK4, EGFR, ESR1, ESR2, GSK3B, GSTP1, HSPA8, IL1B, IL6, IRS1, MAPK1, MAPK14, MAPK3, MAPK8, MMP2, NFKB1, NOS3, PARP1, PPARG, RELA, SRC, TLR4]
KEGG:05219	Bladder cancer	KEGG	3.85E-21	4.89E-20	29.27	12.00	[CCND1, CDH1, CDK4, EGFR, ERBB2, MAP2K1, MAPK1, MAPK3, MMP2, MMP9, SRC, TP53]
KEGG:04210	Apoptosis	KEGG	4.67E-21	5.80E-20	11.76	16.00	[AKT1, BCL2L1, CASP3, CASP9, FOS, JUN, MAP2K1, MAPK1, MAPK3, MAPK8, MAPK9, MCL1, NFKB1, PARP1, RELA, TP53]
KEGG:04659	Th17 cell differentiation	KEGG	6.60E-21	8.03E-20	13.89	15.00	[FOS, HSP90AA1, IL1B, IL6, JUN, MAPK1, MAPK14, MAPK3, MAPK8, MAPK9, MTOR, NFKB1, RELA, SMAD2, STAT3]
KEGG:05165	Human papillomavirus infection	KEGG	2.22E-20	2.65E-19	6.04	20.00	[AKT1, CASP3, CCNA2, CCND1, CDK2, CDK4, CDK6, EGFR, FN1, FOXO1, GSK3B, MAP2K1, MAPK1, MAPK3, MTOR, NFKB1, PTGS2, PTK2, RELA, TP53]
KEGG:05222	Small cell lung cancer	KEGG	3.82E-20	4.46E-19	15.22	14.00	[AKT1, BCL2L1, CASP3, CASP9, CCND1, CDK2, CDK4, CDK6, FN1, NFKB1, PTGS2, PTK2, RELA, TP53]
GO:0010038	response to metal ion	GO_BP	3.90E-20	4.46E-19	5.25	21.00	[AKT1, APP, CASP3, CASP9, CCND1, CDH1, CDK1, CYP1A1, EGFR, FOS, HMOX1, JUN, MAPK1, MAPK3, MAPK8, MAPK9, MMP9,

							NFE2L2, NQO1, PARP1, PTGS2]
KEGG:05160	Hepatitis C	KEGG	5.02E-20	5.64E-19	10.19	16.00	[AKT1, CASP3, CASP9, CCND1, CDK2, CDK4, CDK6, EGFR, GSK3B, MAP2K1, MAPK1, MAPK3, NFKB1, RELA, STAT3, TP53]
KEGG:05169	Epstein-Barr virus infection	KEGG	8.32E-20	9.16E-19	8.42	17.00	[AKT1, CASP3, CASP9, CCNA2, CCND1, CDK2, CDK4, CDK6, IL6, JUN, MAPK14, MAPK8, MAPK9, NFKB1, RELA, STAT3, TP53]
KEGG:05142	Chagas disease	KEGG	1.76E-19	1.90E-18	13.73	14.00	[AKT1, FOS, IL1B, IL6, JUN, MAPK1, MAPK14, MAPK3, MAPK8, MAPK9, NFKB1, RELA, SMAD2, TLR4]
KEGG:05133	Pertussis	KEGG	1.83E-19	1.94E-18	17.11	13.00	[CASP3, FOS, IL1B, IL6, JUN, MAPK1, MAPK14, MAPK3, MAPK8, MAPK9, NFKB1, RELA, TLR4]
KEGG:05207	Chemical carcinogenesis	KEGG	1.91E-19	1.99E-18	8.02	17.00	[AKT1, CCND1, CYP1A1, EGFR, ESR1, ESR2, FOS, HSP90AA1, JUN, MAP2K1, MAPK1, MAPK3, MTOR, NFKB1, RELA, SRC, STAT3]
GO:0043434	response to peptide hormone	GO_BP	2.10E-19	2.15E-18	4.84	21.00	[AGT, AKT1, CCNA2, CDK4, FOS, FOXO1, GSK3B, IL1B, INS, IRS1, MAPK14, MTOR, NFE2L2, NFKB1, PARP1, PPARG, PTGS2, PTK2, RELA, SRC, STAT3]
KEGG:04620	Toll-like receptor signaling pathway	KEGG	2.34E-19	2.36E-18	13.46	14.00	[AKT1, FOS, IL1B, IL6, JUN, MAP2K1, MAPK1, MAPK14, MAPK3, MAPK8, MAPK9, NFKB1, RELA, TLR4]
GO:0071248	cellular response to metal ion	GO_BP	2.43E-19	2.41E-18	7.91	17.00	[AKT1, APP, CDH1, CYP1A1, EGFR, FOS, HMOX1, JUN, MAPK1, MAPK3, MAPK8, MAPK9, MMP9, NFE2L2, NQO1, PARP1, PTGS2]
KEGG:04915	Estrogen signaling pathway	KEGG	3.06E-19	2.97E-18	10.87	15.00	[AKT1, EGFR, ESR1, ESR2, FOS, HSP90AA1, HSPA8, JUN, MAP2K1, MAPK1, MAPK3, MMP2, MMP9, NOS3, SRC]
KEGG:04931	Insulin resistance	KEGG	4.08E-19	3.90E-18	12.96	14.00	[AGT, AKT1, FOXO1, GSK3B, IL6, INS, IRS1, MAPK8, MAPK9, MTOR, NFKB1, NOS3, RELA, STAT3]
KEGG:05145	Toxoplasmosis	KEGG	6.94E-	6.54E-18	12.50	14.00	[AKT1, BCL2L1, CASP3, CASP9, HSPA8, MAPK1, MAPK14, MAPK3, MAPK8, MAPK9, NFKB1,

			19				RELA, STAT3, TLR4]
KEGG:04012	ErbB signaling pathway	KEGG	8.66E-19	8.03E-18	15.29	13.00	[AKT1, EGFR, ERBB2, GSK3B, JUN, MAP2K1, MAPK1, MAPK3, MAPK8, MAPK9, MTOR, PTK2, SRC]
GO:0048144	fibroblast proliferation	GO_BP	8.99E-19	8.20E-18	12.28	14.00	[AGT, CCNA2, CCNB1, CDK1, CDK4, CDK6, EGFR, ESR1, FN1, GSTP1, IGF1, JUN, PPARG, TP53]
GO:2001233	regulation of apoptotic signaling pathway	GO_BP	9.82E-19	8.83E-18	4.99	20.00	[AGT, AKT1, BCL2L1, GSK3B, GSTP1, HMOX1, IGF1, IL1B, IL6, INS, MAPK9, MCL1, MMP9, NFE2L2, NOS3, PARP1, PTGS2, RELA, SRC, TP53]
KEGG:05235	PD-L1 expression and PD-1 checkpoint pathway in cancer	KEGG	1.63E-18	1.44E-17	14.61	13.00	[AKT1, EGFR, FOS, JUN, MAP2K1, MAPK1, MAPK14, MAPK3, MTOR, NFKB1, RELA, STAT3, TLR4]
KEGG:04935	Growth hormone synthesis, secretion and action	KEGG	1.68E-18	1.46E-17	11.76	14.00	[AKT1, FOS, GSK3B, IGF1, IRS1, MAP2K1, MAPK1, MAPK14, MAPK3, MAPK8, MAPK9, MTOR, PTK2, STAT3]
KEGG:04932	Non-alcoholic fatty liver disease	KEGG	1.83E-18	1.57E-17	9.68	15.00	[AKT1, CASP3, FOS, GSK3B, IL1B, IL6, INS, IRS1, JUN, MAPK14, MAPK8, MAPK9, NFKB1, PPARG, RELA]
GO:0032496	response to lipopolysaccharide	GO_BP	2.64E-18	2.24E-17	5.31	19.00	[AKT1, CASP3, CASP9, CDK4, CYP1A1, FOS, GSTP1, IL1B, IL6, MAPK1, MAPK14, MAPK3, MAPK8, NFKB1, NOS3, PTGS2, RELA, SRC, TLR4]
KEGG:04510	Focal adhesion	KEGG	2.82E-18	2.35E-17	7.96	16.00	[AKT1, CCND1, EGFR, ERBB2, FN1, GSK3B, IGF1, JUN, KDR, MAP2K1, MAPK1, MAPK3, MAPK8, MAPK9, PTK2, SRC]
KEGG:05415	Diabetic cardiomyopathy	KEGG	3.31E-18	2.72E-17	7.88	16.00	[AGT, AKT1, GSK3B, INS, IRS1, MAPK14, MAPK8, MAPK9, MMP2, MMP9, MTOR, NFKB1, NOS3, PARP1, RELA, SMAD2]
KEGG:05203	Viral carcinogenesis	KEGG	3.58E-18	2.90E-17	7.84	16.00	[CASP3, CCNA2, CCND1, CDK1, CDK2, CDK4, CDK6, CHEK1, JUN, MAPK1, MAPK3, NFKB1, RELA, SRC, STAT3, TP53]

GO:0048145	regulation of fibroblast proliferation	GO_BP	4.61E-18	3.69E-17	13.54	13.00	[AGT, CCNA2, CCNB1, CDK4, CDK6, EGFR, ESR1, FN1, GSTP1, IGF1, JUN, PPARG, TP53]
KEGG:05223	Non-small cell lung cancer	KEGG	6.93E-18	5.47E-17	16.67	12.00	[AKT1, CASP9, CCND1, CDK4, CDK6, EGFR, ERBB2, MAP2K1, MAPK1, MAPK3, STAT3, TP53]
GO:0002237	response to molecule of bacterial origin	GO_BP	7.29E-18	5.68E-17	5.03	19.00	[AKT1, CASP3, CASP9, CDK4, CYP1A1, FOS, GSTP1, IL1B, IL6, MAPK1, MAPK14, MAPK3, MAPK8, NFKB1, NOS3, PTGS2, RELA, SRC, TLR4]
GO:0071375	cellular response to peptide hormone stimulus	GO_BP	7.98E-18	6.14E-17	5.64	18.00	[AGT, AKT1, CCNA2, CDK4, FOS, FOXO1, GSK3B, IL1B, INS, IRS1, NFE2L2, NFKB1, PARP1, PPARG, PTK2, RELA, SRC, STAT3]
KEGG:04115	p53 signaling pathway	KEGG	8.28E-18	6.28E-17	16.44	12.00	[BCL2L1, CASP3, CASP9, CCNB1, CCND1, CDK1, CDK2, CDK4, CDK6, CHEK1, IGF1, TP53]
KEGG:05010	Alzheimer disease	KEGG	9.77E-18	7.32E-17	4.95	19.00	[AKT1, APP, CASP3, CASP9, CDK5, GSK3B, IL1B, IL6, INS, IRS1, MAP2K1, MAPK1, MAPK3, MAPK8, MAPK9, MTOR, NFKB1, PTGS2, RELA]
KEGG:04660	T cell receptor signaling pathway	KEGG	1.37E-17	1.01E-16	12.50	13.00	[AKT1, CDK4, FOS, GSK3B, JUN, MAP2K1, MAPK1, MAPK14, MAPK3, MAPK8, MAPK9, NFKB1, RELA]
KEGG:04625	C-type lectin receptor signaling pathway	KEGG	1.37E-17	1.01E-16	12.50	13.00	[AKT1, IL1B, IL6, JUN, MAPK1, MAPK14, MAPK3, MAPK8, MAPK9, NFKB1, PTGS2, RELA, SRC]
GO:0009612	response to mechanical stimulus	GO_BP	2.00E-17	1.46E-16	7.05	16.00	[AGT, CHEK1, FOS, IL1B, JUN, MAPK14, MAPK3, MAPK8, MMP2, NFKB1, PPARG, PTGS2, PTK2, RELA, SRC, TLR4]
KEGG:05213	Endometrial cancer	KEGG	3.90E-17	2.81E-16	18.97	11.00	[AKT1, CASP9, CCND1, CDH1, EGFR, ERBB2, GSK3B, MAP2K1, MAPK1, MAPK3, TP53]
GO:0071276	cellular response to cadmium ion	GO_BP	4.10E-17	2.92E-16	25.64	10.00	[AKT1, EGFR, FOS, HMOX1, JUN, MAPK1, MAPK3, MAPK8, MAPK9, MMP9]
KEGG:04370	VEGF signaling pathway	KEGG	4.78E-17	3.36E-16	18.64	11.00	[AKT1, CASP9, KDR, MAP2K1, MAPK1, MAPK14, MAPK3, NOS3, PTGS2, PTK2, SRC]
KEGG:05130	Pathogenic Escherichia coli infection	KEGG	7.05E-17	4.90E-16	7.61	15.00	[CASP3, CASP9, FOS, IL1B, IL6, JUN, MAPK1, MAPK14, MAPK3, MAPK8, MAPK9, NFKB1,

							RELA, SRC, TLR4]
KEGG:04722	Neurotrophin signaling pathway	KEGG	8.43E-17	5.79E-16	10.92	13.00	[AKT1, GSK3B, IRS1, JUN, MAP2K1, MAPK1, MAPK14, MAPK3, MAPK8, MAPK9, NFKB1, RELA, TP53]
KEGG:05132	Salmonella infection	KEGG	8.75E-17	5.94E-16	6.43	16.00	[AKT1, CASP3, FOS, HSP90AA1, IL1B, IL6, JUN, MAP2K1, MAPK1, MAPK14, MAPK3, MAPK8, MAPK9, NFKB1, RELA, TLR4]
KEGG:05206	MicroRNAs in cancer	KEGG	1.20E-16	8.08E-16	5.48	17.00	[CASP3, CCND1, CDK6, EGFR, ERBB2, HMOX1, IRS1, MAP2K1, MAPK1, MAPK3, MCL1, MMP9, MTOR, NFKB1, PTGS2, STAT3, TP53]
GO:0018209	peptidyl-serine modification	GO_BP	1.27E-16	8.41E-16	4.83	18.00	[AKT1, APP, AURKA, CDK1, CDK2, CDK5, EGFR, GSK3B, HSP90AA1, IL6, MAPK1, MAPK14, MAPK8, MAPK9, MTOR, PARP1, PTGS2, SRC]
GO:0104004	cellular response to environmental stimulus	GO_BP	1.33E-16	8.71E-16	4.81	18.00	[AGT, BCL2L1, CASP3, CASP9, CHEK1, IL1B, MAPK14, MAPK3, MAPK8, MMP2, MMP3, MMP9, MTOR, NFKB1, PARP1, PTGS2, TLR4, TP53]
GO:0071214	cellular response to abiotic stimulus	GO_BP	1.33E-16	8.71E-16	4.81	18.00	[AGT, BCL2L1, CASP3, CASP9, CHEK1, IL1B, MAPK14, MAPK3, MAPK8, MMP2, MMP3, MMP9, MTOR, NFKB1, PARP1, PTGS2, TLR4, TP53]
GO:0009411	response to UV	GO_BP	1.41E-16	9.17E-16	8.64	14.00	[AKT1, CASP3, CASP9, CCND1, CHEK1, EGFR, MAPK8, MMP2, MMP3, MMP9, PARP1, PTGS2, RELA, TP53]
GO:2001234	negative regulation of apoptotic signaling pathway	GO_BP	1.64E-16	1.05E-15	6.18	16.00	[AKT1, BCL2L1, GSK3B, GSTP1, HMOX1, IGF1, IL1B, IL6, INS, MCL1, MMP9, NFE2L2, NOS3, PTGS2, RELA, SRC]
GO:0033002	muscle cell proliferation	GO_BP	1.98E-16	1.26E-15	7.11	15.00	[AGT, AKT1, CDK1, HMOX1, IGF1, IL6, JUN, MAPK14, MMP2, MMP9, NOS3, PPARG, PTGS2, SRC, TLR4]
GO:0071216	cellular response to biotic stimulus	GO_BP	2.09E-16	1.31E-15	6.08	16.00	[AKT1, CDK4, GSK3B, GSTP1, IL1B, IL6, MAPK1, MAPK14, MAPK3, MAPK8, NFKB1, NOS3, RELA, SRC, TLR4, TP53]
KEGG:04380	Osteoclast differentiation	KEGG	2.23E-	1.39E-15	10.16	13.00	[AKT1, FOS, IL1B, JUN, MAP2K1, MAPK1, MAPK14, MAPK3, MAPK8, MAPK9, NFKB1,

			16				PPARG, RELA]
GO:0009410	response to xenobiotic stimulus	GO_BP	3.63E-16	2.23E-15	4.07	19.00	[CASP3, CCND1, CDH1, CDK1, CDK4, CYP1A1, FOS, GSTP1, HMOX1, HSP90AA1, IL1B, JUN, MMP2, NFE2L2, NQO1, PPARG, PTGS2, SRC, TP53]
GO:0050727	regulation of inflammatory response	GO_BP	4.13E-16	2.49E-15	4.51	18.00	[AGT, APP, CYP19A1, ESR1, GSTP1, IGF1, IL1B, IL6, INS, MAPK14, MMP3, MMP9, NFKB1, PPARG, PTGS2, RELA, SRC, TLR4]
GO:0032355	response to estradiol	GO_BP	4.11E-16	2.50E-15	9.70	13.00	[AGT, CASP3, CASP9, CCNA2, CCND1, CYP19A1, EGFR, ESR1, ESR2, MMP2, NQO1, PTGS2, STAT3]
GO:0046686	response to cadmium ion	GO_BP	4.24E-16	2.53E-15	15.49	11.00	[AKT1, CDK1, EGFR, FOS, HMOX1, JUN, MAPK1, MAPK3, MAPK8, MAPK9, MMP9]
KEGG:05218	Melanoma	KEGG	4.99E-16	2.95E-15	15.28	11.00	[AKT1, CCND1, CDH1, CDK4, CDK6, EGFR, IGF1, MAP2K1, MAPK1, MAPK3, TP53]
KEGG:05152	Tuberculosis	KEGG	6.28E-16	3.67E-15	7.78	14.00	[AKT1, CASP3, CASP9, IL1B, IL6, MAPK1, MAPK14, MAPK3, MAPK8, MAPK9, NFKB1, RELA, SRC, TLR4]
GO:0071496	cellular response to external stimulus	GO_BP	6.83E-16	3.95E-15	4.94	17.00	[AGT, CHEK1, FOS, FOXO1, HMOX1, HSPA8, IL1B, MAPK1, MAPK3, MAPK8, MTOR, NFE2L2, NFKB1, PPARG, PTGS2, TLR4, TP53]
KEGG:05214	Glioma	KEGG	8.03E-16	4.60E-15	14.67	11.00	[AKT1, CCND1, CDK4, CDK6, EGFR, IGF1, MAP2K1, MAPK1, MAPK3, MTOR, TP53]
GO:0018105	peptidyl-serine phosphorylation	GO_BP	8.28E-16	4.65E-15	4.89	17.00	[AKT1, APP, AURKA, CDK1, CDK2, CDK5, EGFR, GSK3B, HSP90AA1, IL6, MAPK1, MAPK14, MAPK8, MAPK9, MTOR, PTGS2, SRC]
KEGG:05171	Coronavirus disease	KEGG	8.20E-16	4.65E-15	6.47	15.00	[EGFR, FOS, IL1B, IL6, JUN, MAPK1, MAPK14, MAPK3, MAPK8, MAPK9, MMP3, NFKB1, RELA, STAT3, TLR4]
KEGG:04936	Alcoholic liver disease	KEGG	8.87E-16	4.94E-15	9.15	13.00	[AKT1, CASP3, CCND1, FOXO1, GSK3B, IL1B, IL6, MAPK14, MAPK8, MAPK9, NFKB1, RELA, TLR4]

KEGG:05220	Chronic myeloid leukemia	KEGG	9.37E-16	5.16E-15	14.47	11.00	[AKT1, BCL2L1, CCND1, CDK4, CDK6, MAP2K1, MAPK1, MAPK3, NFKB1, RELA, TP53]
GO:0070371	ERK1 and ERK2 cascade	GO_BP	9.54E-16	5.21E-15	4.84	17.00	[AGT, APP, CCNA2, CDK1, EGFR, ERBB2, FN1, GSTP1, IGF1, IL1B, JUN, KDR, MAP2K1, MAPK1, MAPK3, SRC, TLR4]
GO:0009416	response to light stimulus	GO_BP	1.05E-15	5.67E-15	4.82	17.00	[AKT1, APP, CASP3, CASP9, CCND1, CDK5, CHEK1, EGFR, FOS, MAPK8, MMP2, MMP3, MMP9, PARP1, PTGS2, RELA, TP53]
GO:2001237	negative regulation of extrinsic apoptotic signaling pathway	GO_BP	1.15E-15	6.17E-15	11.11	12.00	[AKT1, BCL2L1, GSK3B, GSTP1, HMOX1, IGF1, IL1B, IL6, MCL1, NOS3, RELA, SRC]
KEGG:05226	Gastric cancer	KEGG	1.68E-15	8.90E-15	8.72	13.00	[AKT1, CCND1, CDH1, CDK2, EGFR, ERBB2, GSK3B, MAP2K1, MAPK1, MAPK3, MTOR, SMAD2, TP53]
GO:0048660	regulation of smooth muscle cell proliferation	GO_BP	1.68E-15	8.90E-15	8.72	13.00	[AGT, AKT1, HMOX1, IGF1, IL6, JUN, MMP2, MMP9, NOS3, PPARG, PTGS2, SRC, TLR4]
GO:0031331	positive regulation of cellular catabolic process	GO_BP	1.72E-15	9.04E-15	4.16	18.00	[AKT1, APP, AURKA, FOXO1, GSK3B, HMOX1, IGF1, IL1B, IL6, INS, IRS1, KDR, KEAP1, MAPK3, MAPK9, MTOR, NFE2L2, PTK2]
GO:0010001	glial cell differentiation	GO_BP	1.74E-15	9.07E-15	6.15	15.00	[AKT1, APP, CDK1, CDK5, CDK6, ERBB2, IL1B, IL6, MAP2K1, MAPK1, MAPK3, MTOR, PPARG, STAT3, TLR4]
GO:0048659	smooth muscle cell proliferation	GO_BP	2.38E-15	1.23E-14	8.50	13.00	[AGT, AKT1, HMOX1, IGF1, IL6, JUN, MMP2, MMP9, NOS3, PPARG, PTGS2, SRC, TLR4]
GO:0048146	positive regulation of fibroblast proliferation	GO_BP	3.86E-15	1.98E-14	16.95	10.00	[AGT, CCNA2, CCNB1, CDK4, CDK6, EGFR, ESR1, FN1, IGF1, JUN]
GO:0048661	positive regulation of smooth muscle cell proliferation	GO_BP	3.90E-15	1.98E-14	12.79	11.00	[AGT, AKT1, HMOX1, IGF1, IL6, JUN, MMP2, MMP9, PTGS2, SRC, TLR4]
KEGG:04919	Thyroid hormone signaling pathway	KEGG	4.68E-15	2.36E-14	9.92	12.00	[AKT1, CASP9, CCND1, ESR1, FOXO1, GSK3B, MAP2K1, MAPK1, MAPK3, MTOR, SRC, TP53]
GO:0070997	neuron death	GO_BP	5.44E-15	2.72E-14	4.36	17.00	[AKT1, APP, BCL2L1, CASP3, CASP9, CCND1, CDK5, FOS, GSK3B, HMOX1, JUN, KDR, MCL1,

							NQO1, PARP1, TLR4, TP53]
GO:0042063	gliogenesis	GO_BP	7.41E-15	3.67E-14	4.85	16.00	[AKT1, APP, CDK1, CDK5, CDK6, ERBB2, IL1B, IL6, MAP2K1, MAPK1, MAPK3, MTOR, PPARG, STAT3, TLR4, TP53]
KEGG:05164	Influenza A	KEGG	1.02E-14	5.01E-14	7.60	13.00	[AKT1, CASP3, CASP9, CDK4, CDK6, IL1B, IL6, MAP2K1, MAPK1, MAPK3, NFKB1, RELA, TLR4]
GO:2001236	regulation of extrinsic apoptotic signaling pathway	GO_BP	1.02E-14	5.01E-14	7.60	13.00	[AGT, AKT1, BCL2L1, GSK3B, GSTP1, HMOX1, IGF1, IL1B, IL6, MCL1, NOS3, RELA, SRC]
GO:0051403	stress-activated MAPK cascade	GO_BP	1.20E-14	5.83E-14	5.40	15.00	[AGT, APP, EGFR, FOXO1, GSTP1, IL1B, IL6, MAP2K1, MAPK1, MAPK14, MAPK3, MAPK8, MAPK9, NFKB1, TLR4]
GO:1901214	regulation of neuron death	GO_BP	1.41E-14	6.83E-14	4.65	16.00	[AKT1, BCL2L1, CASP3, CASP9, CCND1, CDK5, FOS, GSK3B, HMOX1, JUN, KDR, MCL1, NQO1, PARP1, TLR4, TP53]
KEGG:05221	Acute myeloid leukemia	KEGG	1.49E-14	7.14E-14	14.93	10.00	[AKT1, CCNA2, CCND1, MAP2K1, MAPK1, MAPK3, MTOR, NFKB1, RELA, STAT3]
GO:0071222	cellular response to lipopolysaccharide	GO_BP	1.51E-14	7.19E-14	6.19	14.00	[AKT1, CDK4, GSTP1, IL1B, IL6, MAPK1, MAPK14, MAPK3, MAPK8, NFKB1, NOS3, RELA, SRC, TLR4]
GO:0031098	stress-activated protein kinase signaling cascade	GO_BP	1.73E-14	8.14E-14	5.26	15.00	[AGT, APP, EGFR, FOXO1, GSTP1, IL1B, IL6, MAP2K1, MAPK1, MAPK14, MAPK3, MAPK8, MAPK9, NFKB1, TLR4]
GO:0062012	regulation of small molecule metabolic process	GO_BP	2.21E-14	1.03E-13	4.52	16.00	[AKT1, APP, FOXO1, IGF1, IL1B, INS, IRS1, MTOR, NFKB1, NOS3, PARP1, PPARG, PTGS2, SRC, STAT3, TP53]
KEGG:04621	NOD-like receptor signaling pathway	KEGG	2.65E-14	1.23E-13	7.07	13.00	[BCL2L1, HSP90AA1, IL1B, IL6, JUN, MAPK1, MAPK14, MAPK3, MAPK8, MAPK9, NFKB1, RELA, TLR4]
GO:0071219	cellular response to molecule of bacterial origin	GO_BP	2.76E-14	1.27E-13	5.93	14.00	[AKT1, CDK4, GSTP1, IL1B, IL6, MAPK1, MAPK14, MAPK3, MAPK8, NFKB1, NOS3, RELA, SRC, TLR4]

GO:0010632	regulation of epithelial cell migration	GO_BP	3.90E-14	1.78E-13	5.79	14.00	[AGT, AKT1, HMOX1, JUN, KDR, MAPK14, MMP9, MTOR, NFE2L2, NOS3, PPARG, PTGS2, PTK2, SRC]
KEGG:05140	Leishmaniasis	KEGG	6.43E-14	2.89E-13	12.99	10.00	[FOS, IL1B, JUN, MAPK1, MAPK14, MAPK3, NFKB1, PTGS2, RELA, TLR4]
GO:0004693	cyclin-dependent protein serine/threonine kinase activity	GO_BP	6.42E-14	2.90E-13	8.00	12.00	[AKT1, CASP3, CCNA2, CCNB1, CCND1, CDK1, CDK2, CDK4, CDK5, CDK6, EGFR, SRC]
GO:0106310	protein serine kinase activity	GO_MF	7.46E-14	3.33E-13	4.18	16.00	[AKT1, AURKA, CDK1, CDK2, CDK4, CDK5, CDK6, CHEK1, GSK3B, MAP2K1, MAPK1, MAPK14, MAPK3, MAPK8, MAPK9, MTOR]
GO:0097472	cyclin-dependent protein kinase activity	GO_BP	8.15E-14	3.61E-13	7.84	12.00	[AKT1, CASP3, CCNA2, CCNB1, CCND1, CDK1, CDK2, CDK4, CDK5, CDK6, EGFR, SRC]
GO:0051222	positive regulation of protein transport	GO_BP	9.42E-14	4.14E-13	4.69	15.00	[CDH1, CDK1, CDK5, ERBB2, GSK3B, HSP90AA1, IGF1, IL1B, INS, MAPK14, MAPK8, PPARG, PTGS2, SRC, TLR4]
GO:0014065	phosphatidylinositol 3-kinase signaling	GO_BP	1.03E-13	4.49E-13	7.69	12.00	[AGT, AKT1, EGFR, ERBB2, FN1, IGF1, IL6, INS, IRS1, KDR, PTK2, SRC]
GO:0019902	phosphatase binding	GO_MF	1.14E-13	4.95E-13	6.31	13.00	[EGFR, ERBB2, FOXO1, HSP90AA1, MAPK1, MAPK14, MAPK3, MAPK8, PPARG, PTK2, SMAD2, STAT3, TP53]
GO:1904645	response to amyloid-beta	GO_BP	1.17E-13	5.01E-13	16.67	9.00	[APP, CDK5, GSK3B, IGF1, MMP2, MMP3, MMP9, PARP1, TLR4]
KEGG:04071	Sphingolipid signaling pathway	KEGG	1.55E-13	6.60E-13	9.24	11.00	[AKT1, MAP2K1, MAPK1, MAPK14, MAPK3, MAPK8, MAPK9, NFKB1, NOS3, RELA, TP53]
GO:0062013	positive regulation of small molecule metabolic process	GO_BP	1.62E-13	6.87E-13	7.41	12.00	[AKT1, APP, FOXO1, IGF1, IL1B, INS, IRS1, MTOR, NOS3, PPARG, PTGS2, SRC]
GO:0010634	positive regulation of epithelial cell migration	GO_BP	1.62E-13	6.87E-13	7.41	12.00	[AGT, AKT1, HMOX1, JUN, KDR, MAPK14, MMP9, MTOR, NFE2L2, NOS3, PTGS2, SRC]
GO:1904951	positive regulation of establishment of protein localization	GO_BP	1.91E-13	8.05E-13	4.46	15.00	[CDH1, CDK1, CDK5, ERBB2, GSK3B, HSP90AA1, IGF1, IL1B, INS, MAPK14, MAPK8, PPARG, PTGS2, SRC, TLR4]

GO:0004707	MAP kinase activity	GO_BP	2.67E-13	1.11E-12	5.91	13.00	[AGT, EGFR, ERBB2, GSTP1, IL1B, MAPK1, MAPK14, MAPK3, MAPK8, MAPK9, PPARG, SRC, TLR4]
KEGG:04211	Longevity regulating pathway	KEGG	2.89E-13	1.20E-12	11.24	10.00	[AKT1, FOXO1, IGF1, INS, IRS1, MTOR, NFKB1, PPARG, RELA, TP53]
KEGG:04110	Cell cycle	KEGG	2.93E-13	1.21E-12	8.73	11.00	[CCNA2, CCNB1, CCND1, CDK1, CDK2, CDK4, CDK6, CHEK1, GSK3B, SMAD2, TP53]
KEGG:04912	GnRH signaling pathway	KEGG	4.55E-13	1.86E-12	10.75	10.00	[EGFR, JUN, MAP2K1, MAPK1, MAPK14, MAPK3, MAPK8, MAPK9, MMP2, SRC]
GO:1901216	positive regulation of neuron death	GO_BP	5.08E-13	2.06E-12	10.64	10.00	[CASP3, CASP9, CDK5, FOS, GSK3B, MCL1, NQO1, PARP1, TLR4, TP53]
GO:0016572	histone phosphorylation	GO_BP	6.07E-13	2.44E-12	20.00	8.00	[AURKA, CCNA2, CDK1, CDK2, CDK5, CHEK1, IL1B, MAPK3]
KEGG:04014	Ras signaling pathway	KEGG	6.20E-13	2.48E-12	5.53	13.00	[AKT1, BCL2L1, EGFR, IGF1, INS, KDR, MAP2K1, MAPK1, MAPK3, MAPK8, MAPK9, NFKB1, RELA]
GO:0010506	regulation of autophagy	GO_BP	7.14E-13	2.84E-12	4.08	15.00	[AKT1, CASP3, CDK5, FOXO1, GSK3B, HMOX1, IL6, KDR, KEAP1, MAPK3, MAPK8, MCL1, MTOR, STAT3, TP53]
KEGG:04910	Insulin signaling pathway	KEGG	7.43E-13	2.93E-12	8.03	11.00	[AKT1, FOXO1, GSK3B, INS, IRS1, MAP2K1, MAPK1, MAPK3, MAPK8, MAPK9, MTOR]
KEGG:05231	Choline metabolism in cancer	KEGG	7.79E-13	3.05E-12	10.20	10.00	[AKT1, EGFR, FOS, JUN, MAP2K1, MAPK1, MAPK3, MAPK8, MAPK9, MTOR]
GO:0097191	extrinsic apoptotic signaling pathway	GO_BP	8.55E-13	3.33E-12	5.39	13.00	[AGT, AKT1, BCL2L1, GSK3B, GSTP1, HMOX1, IGF1, IL1B, IL6, MCL1, NOS3, RELA, SRC]
GO:0043467	regulation of generation of precursor metabolites and energy	GO_BP	8.73E-13	3.38E-12	7.91	11.00	[AKT1, APP, CCNB1, CDK1, GSK3B, IGF1, INS, IRS1, MTOR, STAT3, TP53]
GO:0048015	phosphatidylinositol-mediated signaling	GO_BP	1.24E-12	4.72E-12	6.25	12.00	[AGT, AKT1, EGFR, ERBB2, FN1, IGF1, IL6, INS, IRS1, KDR, PTK2, SRC]
GO:0046777	protein autophosphorylation	GO_BP	1.23E-12	4.73E-12	5.24	13.00	[AKT1, AURKA, CDK5, EGFR, ERBB2, GSK3B, INS, JUN, KDR, MAPK3, MTOR, PTK2, SRC]

KEGG:05202	Transcriptional misregulation in cancer	KEGG	1.32E-12	4.99E-12	6.22	12.00	[BCL2L1, CCNA2, FOXO1, IGF1, IL6, MMP3, MMP9, NFKB1, PPARG, PTK2, RELA, TP53]
KEGG:05120	Epithelial cell signaling in Helicobacter pylori infection	KEGG	1.37E-12	5.16E-12	12.86	9.00	[CASP3, EGFR, JUN, MAPK14, MAPK8, MAPK9, NFKB1, RELA, SRC]
GO:0048017	inositol lipid-mediated signaling	GO_BP	1.68E-12	6.28E-12	6.09	12.00	[AGT, AKT1, EGFR, ERBB2, FN1, IGF1, IL6, INS, IRS1, KDR, PTK2, SRC]
GO:0006109	regulation of carbohydrate metabolic process	GO_BP	1.68E-12	6.28E-12	6.09	12.00	[AKT1, APP, FOXO1, GSK3B, IGF1, INS, IRS1, MTOR, NFKB1, SRC, STAT3, TP53]
GO:0070372	regulation of ERK1 and ERK2 cascade	GO_BP	1.85E-12	6.88E-12	4.36	14.00	[APP, CCNA2, EGFR, ERBB2, FN1, GSTP1, IGF1, IL1B, JUN, KDR, MAP2K1, MAPK3, SRC, TLR4]
GO:0010631	epithelial cell migration	GO_BP	2.10E-12	7.76E-12	4.32	14.00	[AGT, AKT1, HMOX1, JUN, KDR, MAPK14, MMP9, MTOR, NFE2L2, NOS3, PPARG, PTGS2, PTK2, SRC]
GO:0072593	reactive oxygen species metabolic process	GO_BP	2.24E-12	8.24E-12	5.00	13.00	[AGT, CCNA2, CYP1A1, FOXO1, GSTP1, INS, MAPK14, MMP3, NFE2L2, NOS3, NQO1, TLR4, TP53]
GO:0090132	epithelium migration	GO_BP	2.38E-12	8.67E-12	4.28	14.00	[AGT, AKT1, HMOX1, JUN, KDR, MAPK14, MMP9, MTOR, NFE2L2, NOS3, PPARG, PTGS2, PTK2, SRC]
GO:0034504	protein localization to nucleus	GO_BP	2.69E-12	9.75E-12	4.24	14.00	[AGT, AKT1, CDH1, CDK1, CDK5, GSK3B, HSP90AA1, INS, MAPK14, PARP1, PTGS2, SRC, STAT3, TP53]
GO:0051402	neuron apoptotic process	GO_BP	3.14E-12	1.13E-11	4.87	13.00	[APP, BCL2L1, CASP3, CASP9, CCND1, CDK5, HMOX1, JUN, KDR, MCL1, NQO1, PARP1, TP53]
GO:0032868	response to insulin	GO_BP	4.16E-12	1.49E-11	4.76	13.00	[AGT, AKT1, CDK4, FOXO1, GSK3B, IL1B, INS, IRS1, MAPK14, MTOR, PARP1, PPARG, SRC]
GO:0010821	regulation of mitochondrion organization	GO_BP	4.40E-12	1.57E-11	6.83	11.00	[AKT1, AURKA, BCL2L1, GSK3B, IGF1, IL6, KDR, MAPK8, MMP9, PPARG, TP53]
KEGG:04662	B cell receptor signaling pathway	KEGG	5.99E-12	2.12E-11	10.98	9.00	[AKT1, FOS, GSK3B, JUN, MAP2K1, MAPK1, MAPK3, NFKB1, RELA]
GO:0045913	positive regulation of carbohydrate metabolic	GO_BP	5.99E-12	2.12E-11	10.98	9.00	[AKT1, APP, FOXO1, IGF1, INS, IRS1, MTOR, NFKB1, SRC]

	process						
GO:0070141	response to UV-A	GO_BP	7.68E-12	2.70E-11	37.50	6.00	[AKT1, CCND1, EGFR, MMP2, MMP3, MMP9]
GO:0043523	regulation of neuron apoptotic process	GO_BP	8.94E-12	3.13E-11	5.29	12.00	[BCL2L1, CASP3, CASP9, CCND1, CDK5, HMOX1, JUN, KDR, MCL1, NQO1, PARP1, TP53]
GO:0071453	cellular response to oxygen levels	GO_BP	9.65E-12	3.36E-11	6.36	11.00	[AKT1, CCNA2, FOS, FOXO1, HMOX1, MTOR, NFE2L2, PPARG, PTGS2, SRC, TP53]
GO:0004497	monooxygenase activity	GO_MF	9.65E-12	3.36E-11	6.36	11.00	[AKT1, CYP19A1, CYP1A1, EGFR, ESR1, HMOX1, HSP90AA1, IL1B, INS, NFKB1, NOS3]
KEGG:05134	Legionellosis	KEGG	1.25E-11	4.31E-11	14.04	8.00	[CASP3, CASP9, HSPA8, IL1B, IL6, NFKB1, RELA, TLR4]
GO:0048708	astrocyte differentiation	GO_BP	1.41E-11	4.86E-11	10.00	9.00	[APP, CDK6, IL1B, IL6, MAP2K1, MAPK1, MAPK3, STAT3, TLR4]
KEGG:04114	Oocyte meiosis	KEGG	1.48E-11	5.04E-11	7.63	10.00	[AURKA, CCNB1, CDK1, CDK2, IGF1, INS, MAP2K1, MAPK1, MAPK14, MAPK3]
GO:1900182	positive regulation of protein localization to nucleus	GO_BP	1.57E-11	5.31E-11	9.89	9.00	[AKT1, CDH1, CDK1, HSP90AA1, INS, MAPK14, PARP1, PTGS2, SRC]
KEGG:04658	Th1 and Th2 cell differentiation	KEGG	1.73E-11	5.84E-11	9.78	9.00	[FOS, JUN, MAPK1, MAPK14, MAPK3, MAPK8, MAPK9, NFKB1, RELA]
GO:0097193	intrinsic apoptotic signaling pathway	GO_BP	2.41E-11	8.09E-11	4.14	13.00	[AKT1, BCL2L1, CASP3, CASP9, HMOX1, INS, MCL1, MMP9, NFE2L2, PARP1, PTGS2, SRC, TP53]
GO:0016709	oxidoreductase activity, acting on paired donors, with incorporation or reduction of molecular oxygen, NAD(P)H as one donor, and incorporation of one atom of oxygen	GO_MF	2.56E-11	8.53E-11	9.38	9.00	[AKT1, CYP1A1, EGFR, ESR1, HSP90AA1, IL1B, INS, NFKB1, NOS3]
KEGG:05216	Thyroid cancer	KEGG	2.79E-11	9.26E-11	18.92	7.00	[CCND1, CDH1, MAP2K1, MAPK1, MAPK3, PPARG, TP53]

GO:1904705	regulation of vascular associated smooth muscle cell proliferation	GO_BP	2.88E-11	9.51E-11	12.70	8.00	[AGT, HMOX1, IGF1, JUN, MMP2, MMP9, PPARG, SRC]
KEGG:04140	Autophagy	KEGG	3.08E-11	1.01E-10	7.09	10.00	[AKT1, BCL2L1, INS, IRS1, MAP2K1, MAPK1, MAPK3, MAPK8, MAPK9, MTOR]
GO:0036293	response to decreased oxygen levels	GO_BP	3.30E-11	1.08E-10	4.04	13.00	[AKT1, CASP3, CCNA2, CYP1A1, FOS, HMOX1, MMP2, MTOR, NFE2L2, PPARG, PTGS2, SRC, TP53]
GO:0016705	oxidoreductase activity, acting on paired donors, with incorporation or reduction of molecular oxygen	GO_MF	3.48E-11	1.13E-10	4.71	12.00	[AKT1, CYP19A1, CYP1A1, EGFR, ESR1, HMOX1, HSP90AA1, IL1B, INS, NFKB1, NOS3, PTGS2]
KEGG:05321	Inflammatory bowel disease	KEGG	3.74E-11	1.21E-10	12.31	8.00	[IL1B, IL6, JUN, NFKB1, RELA, SMAD2, STAT3, TLR4]
GO:1990874	vascular associated smooth muscle cell proliferation	GO_BP	3.74E-11	1.21E-10	12.31	8.00	[AGT, HMOX1, IGF1, JUN, MMP2, MMP9, PPARG, SRC]
GO:0090068	positive regulation of cell cycle process	GO_BP	4.17E-11	1.34E-10	4.63	12.00	[AKT1, APP, AURKA, CCNB1, CCND1, CDK1, CDK4, CYP1A1, EGFR, IGF1, IL1B, INS]
GO:1900180	regulation of protein localization to nucleus	GO_BP	4.99E-11	1.59E-10	6.76	10.00	[AKT1, CDH1, CDK1, GSK3B, HSP90AA1, INS, MAPK14, PARP1, PTGS2, SRC]
GO:0009266	response to temperature stimulus	GO_BP	5.76E-11	1.83E-10	5.39	11.00	[AKT1, FOS, FOXO1, GSK3B, HMOX1, HSP90AA1, IGF1, MTOR, NOS3, PPARG, PTGS2]
KEGG:04920	Adipocytokine signaling pathway	KEGG	6.13E-11	1.93E-10	11.59	8.00	[AKT1, IRS1, MAPK8, MAPK9, MTOR, NFKB1, RELA, STAT3]
KEGG:05230	Central carbon metabolism in cancer	KEGG	6.90E-11	2.17E-10	11.43	8.00	[AKT1, EGFR, ERBB2, MAP2K1, MAPK1, MAPK3, MTOR, TP53]
KEGG:04921	Oxytocin signaling pathway	KEGG	7.40E-11	2.31E-10	6.49	10.00	[CCND1, EGFR, FOS, JUN, MAP2K1, MAPK1, MAPK3, NOS3, PTGS2, SRC]
GO:0031099	regeneration	GO_BP	7.47E-11	2.32E-10	5.26	11.00	[AURKA, CCNA2, CCND1, CDK1, HMOX1, IGF1, IL6, JUN, MAP2K1, MMP2, PPARG]
KEGG:04934	Cushing syndrome	KEGG	7.89E-11	2.43E-10	6.45	10.00	[AGT, CCND1, CDK2, CDK4, CDK6, EGFR, GSK3B, MAP2K1, MAPK1, MAPK3]

GO:0036294	cellular response to decreased oxygen levels	GO_BP	7.89E-11	2.43E-10	6.45	10.00	[AKT1, CCNA2, FOS, HMOX1, MTOR, NFE2L2, PPARG, PTGS2, SRC, TP53]
KEGG:04015	Rap1 signaling pathway	KEGG	7.86E-11	2.43E-10	5.24	11.00	[AKT1, CDH1, EGFR, IGF1, INS, KDR, MAP2K1, MAPK1, MAPK14, MAPK3, SRC]
GO:0150076	neuroinflammatory response	GO_BP	8.70E-11	2.66E-10	11.11	8.00	[APP, IGF1, IL1B, IL6, JUN, MMP3, MMP9, PTGS2]
GO:0019903	protein phosphatase binding	GO_MF	1.02E-10	3.09E-10	6.29	10.00	[EGFR, ERBB2, FOXO1, HSP90AA1, MAPK14, MAPK8, PPARG, PTK2, STAT3, TP53]
GO:0032869	cellular response to insulin stimulus	GO_BP	1.06E-10	3.22E-10	5.09	11.00	[AGT, AKT1, CDK4, FOXO1, GSK3B, IL1B, INS, IRS1, PARP1, PPARG, SRC]
GO:0051091	positive regulation of DNA-binding transcription factor activity	GO_BP	1.07E-10	3.23E-10	4.27	12.00	[AGT, AKT1, APP, ESR1, ESR2, IL1B, IL6, INS, PPARG, RELA, STAT3, TLR4]
KEGG:04930	Type II diabetes mellitus	KEGG	1.42E-10	4.25E-10	15.22	7.00	[INS, IRS1, MAPK1, MAPK3, MAPK8, MAPK9, MTOR]
GO:1902749	regulation of cell cycle G2/M phase transition	GO_BP	1.43E-10	4.26E-10	7.76	9.00	[APP, AURKA, CCNB1, CCND1, CDK1, CDK2, CDK4, CHEK1, TP53]
GO:0045834	positive regulation of lipid metabolic process	GO_BP	1.46E-10	4.34E-10	6.06	10.00	[AKT1, CCNA2, IL1B, INS, IRS1, MTOR, PPARG, PTGS2, PTK2, SRC]
GO:0044839	cell cycle G2/M phase transition	GO_BP	1.46E-10	4.34E-10	6.06	10.00	[APP, AURKA, CCNA2, CCNB1, CCND1, CDK1, CDK2, CDK4, CHEK1, TP53]
GO:0010675	regulation of cellular carbohydrate metabolic process	GO_BP	1.46E-10	4.34E-10	6.06	10.00	[AKT1, FOXO1, GSK3B, IGF1, INS, IRS1, MTOR, SRC, STAT3, TP53]
GO:0014066	regulation of phosphatidylinositol 3-kinase signaling	GO_BP	1.80E-10	5.30E-10	7.56	9.00	[AGT, EGFR, FN1, IGF1, IL6, INS, KDR, PTK2, SRC]
GO:0060964	regulation of miRNA-mediated gene silencing	GO_BP	1.94E-10	5.67E-10	14.58	7.00	[EGFR, ESR1, IL6, MAP2K1, PPARG, STAT3, TP53]
KEGG:04152	AMPK signaling pathway	KEGG	1.94E-10	5.69E-10	7.50	9.00	[AKT1, CCNA2, CCND1, FOXO1, IGF1, INS, IRS1, MTOR, PPARG]

GO:0008286	insulin receptor signaling pathway	GO_BP	1.94E-10	5.69E-10	7.50	9.00	[AGT, AKT1, CDK4, FOXO1, GSK3B, IL1B, INS, IRS1, SRC]
GO:0071260	cellular response to mechanical stimulus	GO_BP	2.06E-10	6.00E-10	10.00	8.00	[AGT, CHEK1, IL1B, MAPK3, MAPK8, NFKB1, PTGS2, TLR4]
GO:0090316	positive regulation of intracellular protein transport	GO_BP	2.08E-10	6.02E-10	5.85	10.00	[CDH1, CDK1, CDK5, ERBB2, GSK3B, HSP90AA1, IL1B, MAPK14, MAPK8, PTGS2]
GO:0002573	myeloid leukocyte differentiation	GO_BP	2.50E-10	7.20E-10	4.70	11.00	[APP, CDK6, FOS, JUN, MAPK14, MMP9, MTOR, PARP1, PPARG, SRC, TLR4]
GO:1903201	regulation of oxidative stress-induced cell death	GO_BP	2.53E-10	7.23E-10	9.76	8.00	[AKT1, IL6, INS, MCL1, MMP3, NFE2L2, PARP1, TLR4]
GO:1900368	regulation of post-transcriptional gene silencing by RNA	GO_BP	2.62E-10	7.46E-10	14.00	7.00	[EGFR, ESR1, IL6, MAP2K1, PPARG, STAT3, TP53]
GO:0004712	protein serine/threonine/tyrosine kinase activity	GO_MF	2.62E-10	7.46E-10	14.00	7.00	[AKT1, AURKA, MAP2K1, MAPK1, MAPK14, MAPK3, MAPK9]
GO:1903798	regulation of miRNA maturation	GO_BP	2.76E-10	7.82E-10	22.22	6.00	[EGFR, ESR1, IL6, MAP2K1, STAT3, TP53]
GO:0006809	nitric oxide biosynthetic process	GO_BP	2.79E-10	7.86E-10	9.64	8.00	[AGT, AKT1, HSP90AA1, IL1B, NOS3, NQO1, PTGS2, TLR4]
GO:0060147	regulation of post-transcriptional gene silencing	GO_BP	3.03E-10	8.50E-10	13.73	7.00	[EGFR, ESR1, IL6, MAP2K1, PPARG, STAT3, TP53]
GO:0018107	peptidyl-threonine phosphorylation	GO_BP	3.23E-10	9.02E-10	7.09	9.00	[AKT1, APP, CDK1, CDK5, CHEK1, GSK3B, MAPK1, MAPK8, MTOR]
GO:0070920	regulation of production of small RNA involved in gene silencing by RNA	GO_BP	3.50E-10	9.70E-10	21.43	6.00	[EGFR, ESR1, IL6, MAP2K1, STAT3, TP53]
GO:0060966	regulation of gene silencing by RNA	GO_BP	3.49E-10	9.71E-10	13.46	7.00	[EGFR, ESR1, IL6, MAP2K1, PPARG, STAT3, TP53]

GO:0004517	nitric-oxide synthase activity	GO_MF	3.49E-10	9.71E-10	13.46	7.00	[AKT1, EGFR, ESR1, HSP90AA1, IL1B, INS, NOS3]
GO:2001242	regulation of intrinsic apoptotic signaling pathway	GO_BP	3.64E-10	1.00E-09	5.52	10.00	[AKT1, BCL2L1, INS, MCL1, MMP9, NFE2L2, PARP1, PTGS2, SRC, TP53]
GO:0010594	regulation of endothelial cell migration	GO_BP	3.64E-10	1.00E-09	5.52	10.00	[AGT, AKT1, HMOX1, KDR, MAPK14, NFE2L2, NOS3, PPARG, PTGS2, PTK2]
GO:0038127	ERBB signaling pathway	GO_BP	3.71E-10	1.02E-09	6.98	9.00	[AGT, AKT1, APP, EGFR, ERBB2, MAPK1, MMP9, PTK2, SRC]
GO:0035994	response to muscle stretch	GO_BP	4.41E-10	1.20E-09	20.69	6.00	[FOS, JUN, MAPK14, NFKB1, PTK2, RELA]
GO:0014068	positive regulation of phosphatidylinositol 3-kinase signaling	GO_BP	4.92E-10	1.34E-09	8.99	8.00	[AGT, FN1, IGF1, IL6, INS, KDR, PTK2, SRC]
GO:2000630	positive regulation of miRNA metabolic process	GO_BP	5.25E-10	1.42E-09	12.73	7.00	[FOS, JUN, NFKB1, PPARG, RELA, STAT3, TP53]
GO:0034612	response to tumor necrosis factor	GO_BP	5.73E-10	1.54E-09	4.35	11.00	[AKT1, CASP3, GSTP1, MAPK1, MAPK14, MAPK3, NFE2L2, NFKB1, PTGS2, RELA, TP53]
KEGG:04613	Neutrophil extracellular trap formation	KEGG	5.85E-10	1.57E-09	5.26	10.00	[AKT1, MAP2K1, MAPK1, MAPK14, MAPK3, MTOR, NFKB1, RELA, SRC, TLR4]
GO:0046209	nitric oxide metabolic process	GO_BP	5.88E-10	1.57E-09	8.79	8.00	[AGT, AKT1, HSP90AA1, IL1B, NOS3, NQO1, PTGS2, TLR4]
GO:2001057	reactive nitrogen species metabolic process	GO_BP	6.43E-10	1.71E-09	8.70	8.00	[AGT, AKT1, HSP90AA1, IL1B, NOS3, NQO1, PTGS2, TLR4]
GO:1901992	positive regulation of mitotic cell cycle phase transition	GO_BP	6.43E-10	1.71E-09	8.70	8.00	[AKT1, APP, CCNB1, CCND1, CDK1, CDK4, CYP1A1, EGFR]
KEGG:04062	Chemokine signaling pathway	KEGG	6.48E-10	1.71E-09	5.21	10.00	[AKT1, GSK3B, MAP2K1, MAPK1, MAPK3, NFKB1, PTK2, RELA, SRC, STAT3]
GO:1900407	regulation of cellular response to oxidative stress	GO_BP	7.02E-10	1.85E-09	8.60	8.00	[AKT1, IL6, INS, MCL1, MMP3, NFE2L2, PARP1, TLR4]
KEGG:04371	Apelin signaling pathway	KEGG	7.24E-10	1.90E-09	6.47	9.00	[AKT1, CCND1, CDH1, MAP2K1, MAPK1, MAPK3, MTOR, NOS3, SMAD2]

GO:0042542	response to hydrogen peroxide	GO_BP	7.24E-10	1.90E-09	6.47	9.00	[CASP3, CDK1, HMOX1, IL6, MMP2, NFE2L2, NQO1, RELA, SRC]
GO:0018210	peptidyl-threonine modification	GO_BP	7.24E-10	1.90E-09	6.47	9.00	[AKT1, APP, CDK1, CDK5, CHEK1, GSK3B, MAPK1, MAPK8, MTOR]
GO:0035196	miRNA processing	GO_BP	7.72E-10	2.01E-09	12.07	7.00	[EGFR, ESR1, IL6, MAP2K1, SMAD2, STAT3, TP53]
GO:0043536	positive regulation of blood vessel endothelial cell migration	GO_BP	8.74E-10	2.27E-09	11.86	7.00	[AKT1, HMOX1, KDR, MAPK14, NFE2L2, NOS3, PTGS2]
KEGG:04550	Signaling pathways regulating pluripotency of stem cells	KEGG	9.32E-10	2.41E-09	6.29	9.00	[AKT1, GSK3B, IGF1, MAP2K1, MAPK1, MAPK14, MAPK3, SMAD2, STAT3]
GO:0043535	regulation of blood vessel endothelial cell migration	GO_BP	9.85E-10	2.54E-09	8.25	8.00	[AKT1, HMOX1, KDR, MAPK14, NFE2L2, NOS3, PPARG, PTGS2]
GO:1990776	response to angiotensin	GO_BP	1.02E-09	2.61E-09	18.18	6.00	[AGT, NFE2L2, NFKB1, PTGS2, RELA, SRC]
GO:0032872	regulation of stress-activated MAPK cascade	GO_BP	1.11E-09	2.83E-09	4.93	10.00	[APP, EGFR, FOXO1, GSTP1, IL1B, IL6, MAP2K1, MAPK1, MAPK3, TLR4]
GO:0070918	small regulatory ncRNA processing	GO_BP	1.11E-09	2.84E-09	11.48	7.00	[EGFR, ESR1, IL6, MAP2K1, SMAD2, STAT3, TP53]
GO:0034644	cellular response to UV	GO_BP	1.16E-09	2.94E-09	8.08	8.00	[CASP9, CHEK1, MMP2, MMP3, MMP9, PARP1, PTGS2, TP53]
GO:0031668	cellular response to extracellular stimulus	GO_BP	1.18E-09	2.98E-09	4.06	11.00	[FOS, FOXO1, HMOX1, HSPA8, MAPK1, MAPK3, MAPK8, MTOR, NFE2L2, PPARG, TP53]
GO:0071456	cellular response to hypoxia	GO_BP	1.19E-09	2.99E-09	6.12	9.00	[CCNA2, FOS, HMOX1, MTOR, NFE2L2, PPARG, PTGS2, SRC, TP53]
KEGG:04213	Longevity regulating pathway	KEGG	1.25E-09	3.12E-09	11.29	7.00	[AKT1, FOXO1, HSPA8, IGF1, INS, IRS1, MTOR]
GO:0070302	regulation of stress-activated protein kinase signaling cascade	GO_BP	1.29E-09	3.18E-09	4.85	10.00	[APP, EGFR, FOXO1, GSTP1, IL1B, IL6, MAP2K1, MAPK1, MAPK3, TLR4]

KEGG:05020	Prion disease	KEGG	1.28E-09	3.18E-09	4.03	11.00	[CASP3, CASP9, GSK3B, HSPA8, IL1B, IL6, MAPK1, MAPK14, MAPK3, MAPK8, MAPK9]
GO:0035270	endocrine system development	GO_BP	1.34E-09	3.31E-09	6.04	9.00	[AKT1, CDH1, CDK6, GSK3B, IL6, MAP2K1, MAPK1, MAPK3, SMAD2]
GO:0097305	response to alcohol	GO_BP	1.38E-09	3.39E-09	4.00	11.00	[AKT1, BCL2L1, CCND1, CDH1, CDK1, CDK4, FOS, NQO1, PARP1, PPARG, SMAD2]
GO:0043491	protein kinase B signaling	GO_BP	1.41E-09	3.45E-09	4.81	10.00	[AKT1, EGFR, HSP90AA1, IGF1, IL1B, INS, KDR, MTOR, PTK2, SRC]
GO:0000086	G2/M transition of mitotic cell cycle	GO_BP	1.43E-09	3.47E-09	6.00	9.00	[APP, AURKA, CCNA2, CCNB1, CCND1, CDK1, CDK2, CDK4, CHEK1]
KEGG:05146	Amoebiasis	KEGG	1.48E-09	3.58E-09	7.84	8.00	[CASP3, FN1, IL1B, IL6, NFKB1, PTK2, RELA, TLR4]
GO:0034405	response to fluid shear stress	GO_BP	1.48E-09	3.58E-09	17.14	6.00	[AKT1, MMP2, NFE2L2, NOS3, PTGS2, SRC]
GO:1902882	regulation of response to oxidative stress	GO_BP	1.60E-09	3.84E-09	7.77	8.00	[AKT1, IL6, INS, MCL1, MMP3, NFE2L2, PARP1, TLR4]
GO:0150077	regulation of neuroinflammatory response	GO_BP	1.77E-09	4.25E-09	16.67	6.00	[IGF1, IL1B, IL6, MMP3, MMP9, PTGS2]
GO:0043279	response to alkaloid	GO_BP	1.86E-09	4.44E-09	7.62	8.00	[BCL2L1, CASP3, CCNA2, CDK5, CHEK1, HSP90AA1, NQO1, PPARG]
GO:0036473	cell death in response to oxidative stress	GO_BP	1.86E-09	4.44E-09	7.62	8.00	[AKT1, IL6, INS, MCL1, MMP3, NFE2L2, PARP1, TLR4]
GO:0010389	regulation of G2/M transition of mitotic cell cycle	GO_BP	1.86E-09	4.44E-09	7.62	8.00	[APP, AURKA, CCNB1, CCND1, CDK1, CDK2, CDK4, CHEK1]
KEGG:04150	mTOR signaling pathway	KEGG	2.02E-09	4.79E-09	5.77	9.00	[AKT1, GSK3B, IGF1, INS, IRS1, MAP2K1, MAPK1, MAPK3, MTOR]
GO:0071478	cellular response to radiation	GO_BP	2.03E-09	4.81E-09	4.63	10.00	[BCL2L1, CASP9, CHEK1, MAPK14, MMP2, MMP3, MMP9, PARP1, PTGS2, TP53]
GO:1904707	positive regulation of vascular associated smooth	GO_BP	2.11E-09	4.98E-09	16.22	6.00	[AGT, IGF1, JUN, MMP2, MMP9, SRC]

	muscle cell proliferation						
GO:0046326	positive regulation of glucose import	GO_BP	2.11E-09	4.98E-09	16.22	6.00	[AKT1, IGF1, INS, IRS1, MAPK14, NFE2L2]
GO:0050728	negative regulation of inflammatory response	GO_BP	2.13E-09	5.01E-09	5.73	9.00	[CYP19A1, GSTP1, IGF1, IL6, INS, MAPK14, NFKB1, PPARG, SRC]
GO:0046824	positive regulation of nucleocytoplasmic transport	GO_BP	2.18E-09	5.10E-09	10.45	7.00	[CDH1, CDK1, GSK3B, HSP90AA1, IL1B, MAPK14, PTGS2]
GO:0032388	positive regulation of intracellular transport	GO_BP	2.22E-09	5.17E-09	4.59	10.00	[CDH1, CDK1, CDK5, ERBB2, GSK3B, HSP90AA1, IL1B, MAPK14, MAPK8, PTGS2]
GO:2000377	regulation of reactive oxygen species metabolic process	GO_BP	2.26E-09	5.23E-09	5.70	9.00	[AGT, FOXO1, GSTP1, INS, MAPK14, MMP3, NFE2L2, TLR4, TP53]
KEGG:04664	Fc epsilon RI signaling pathway	KEGG	2.43E-09	5.60E-09	10.29	7.00	[AKT1, MAP2K1, MAPK1, MAPK14, MAPK3, MAPK8, MAPK9]
GO:0032885	regulation of polysaccharide biosynthetic process	GO_BP	2.50E-09	5.75E-09	15.79	6.00	[AKT1, GSK3B, IGF1, INS, IRS1, NFKB1]
KEGG:04024	cAMP signaling pathway	KEGG	2.54E-09	5.81E-09	4.52	10.00	[AKT1, FOS, JUN, MAP2K1, MAPK1, MAPK3, MAPK8, MAPK9, NFKB1, RELA]
GO:0035195	miRNA-mediated gene silencing	GO_BP	2.69E-09	6.15E-09	10.14	7.00	[EGFR, ESR1, IL6, MAP2K1, PPARG, STAT3, TP53]
GO:0001890	placenta development	GO_BP	2.81E-09	6.40E-09	5.56	9.00	[AKT1, CCNA2, EGFR, MAP2K1, MAPK1, MAPK14, PPARG, PTGS2, PTK2]
GO:0031663	lipopolysaccharide-mediated signaling pathway	GO_BP	2.99E-09	6.76E-09	10.00	7.00	[AKT1, IL1B, MAPK1, MAPK14, MAPK3, NOS3, TLR4]
GO:1901654	response to ketone	GO_BP	3.02E-09	6.80E-09	4.44	10.00	[AKT1, BCL2L1, CASP9, CCND1, CDK4, FOS, NQO1, PARP1, PPARG, SRC]
GO:0043500	muscle adaptation	GO_BP	3.12E-09	7.01E-09	7.14	8.00	[AGT, FOXO1, HMOX1, IGF1, IL1B, NOS3, PARP1, PPARG]
GO:0008585	female gonad development	GO_BP	3.35E-09	7.49E-09	7.08	8.00	[BCL2L1, CASP3, CYP19A1, ESR1, KDR, MMP2, NOS3, SRC]
GO:0007173	epidermal growth factor	GO_BP	3.35E-	7.49E-09	7.08	8.00	[AGT, AKT1, APP, EGFR, ERBB2, MMP9, PTK2,

	receptor signaling pathway		09				SRC]
GO:2001243	negative regulation of intrinsic apoptotic signaling pathway	GO_BP	3.59E-09	8.01E-09	7.02	8.00	[AKT1, BCL2L1, INS, MCL1, MMP9, NFE2L2, PTGS2, SRC]
KEGG:04137	Mitophagy	KEGG	3.65E-09	8.11E-09	9.72	7.00	[BCL2L1, JUN, MAPK8, MAPK9, RELA, SRC, TP53]
GO:0030235	nitric-oxide synthase regulator activity	GO_BP	3.67E-09	8.11E-09	57.14	4.00	[AKT1, EGFR, ESR1, HSP90AA1]
GO:1901989	positive regulation of cell cycle phase transition	GO_BP	3.85E-09	8.49E-09	6.96	8.00	[AKT1, APP, CCNB1, CCND1, CDK1, CDK4, CYP1A1, EGFR]
GO:0016241	regulation of macroautophagy	GO_BP	4.08E-09	8.97E-09	5.33	9.00	[AKT1, CASP3, CDK5, HMOX1, KDR, MAPK3, MAPK8, MTOR, TP53]
GO:0046545	development of primary female sexual characteristics	GO_BP	4.42E-09	9.66E-09	6.84	8.00	[BCL2L1, CASP3, CYP19A1, ESR1, KDR, MMP2, NOS3, SRC]
GO:0008637	apoptotic mitochondrial changes	GO_BP	4.42E-09	9.66E-09	6.84	8.00	[AKT1, BCL2L1, GSK3B, IGF1, IL6, MAPK8, MMP9, TP53]
GO:0010907	positive regulation of glucose metabolic process	GO_BP	4.71E-09	1.03E-08	14.29	6.00	[AKT1, FOXO1, IGF1, INS, IRS1, SRC]
GO:0046822	regulation of nucleocytoplasmic transport	GO_BP	4.73E-09	1.03E-08	6.78	8.00	[CDH1, CDK1, CDK5, GSK3B, HSP90AA1, IL1B, MAPK14, PTGS2]
GO:0010595	positive regulation of endothelial cell migration	GO_BP	4.73E-09	1.03E-08	6.78	8.00	[AGT, AKT1, HMOX1, KDR, MAPK14, NFE2L2, NOS3, PTGS2]
GO:0010657	muscle cell apoptotic process	GO_BP	5.36E-09	1.16E-08	9.21	7.00	[AGT, HMOX1, HSP90AA1, IGF1, NFE2L2, PPARG, TP53]
GO:0031669	cellular response to nutrient levels	GO_BP	5.39E-09	1.16E-08	4.18	10.00	[FOXO1, HMOX1, HSPA8, MAPK1, MAPK3, MAPK8, MTOR, NFE2L2, PPARG, TP53]
GO:0043542	endothelial cell migration	GO_BP	5.61E-09	1.20E-08	4.17	10.00	[AGT, AKT1, HMOX1, KDR, MAPK14, NFE2L2, NOS3, PPARG, PTGS2, PTK2]
GO:0031100	animal organ regeneration	GO_BP	5.88E-09	1.26E-08	9.09	7.00	[AURKA, CCNA2, CCND1, CDK1, HMOX1, IL6, PPARG]

GO:0045931	positive regulation of mitotic cell cycle	GO_BP	6.16E-09	1.31E-08	6.56	8.00	[AKT1, APP, CCNB1, CCND1, CDK1, CDK4, CYP1A1, EGFR]
GO:1904646	cellular response to amyloid-beta	GO_BP	6.30E-09	1.34E-08	13.64	6.00	[APP, CDK5, GSK3B, IGF1, PARP1, TLR4]
GO:0032881	regulation of polysaccharide metabolic process	GO_BP	6.30E-09	1.34E-08	13.64	6.00	[AKT1, GSK3B, IGF1, INS, IRS1, NFKB1]
GO:2000628	regulation of miRNA metabolic process	GO_BP	6.45E-09	1.36E-08	8.97	7.00	[FOS, JUN, NFKB1, PPARG, RELA, STAT3, TP53]
GO:0035194	post-transcriptional gene silencing by RNA	GO_BP	6.45E-09	1.36E-08	8.97	7.00	[EGFR, ESR1, IL6, MAP2K1, PPARG, STAT3, TP53]
GO:0033157	regulation of intracellular protein transport	GO_BP	6.83E-09	1.44E-08	4.08	10.00	[CDH1, CDK1, CDK5, ERBB2, GSK3B, HSP90AA1, IL1B, MAPK14, MAPK8, PTGS2]
GO:0006606	protein import into nucleus	GO_BP	7.10E-09	1.49E-08	5.00	9.00	[AGT, AKT1, CDH1, CDK1, HSP90AA1, MAPK14, PTGS2, STAT3, TP53]
GO:0043534	blood vessel endothelial cell migration	GO_BP	7.46E-09	1.56E-08	6.40	8.00	[AKT1, HMOX1, KDR, MAPK14, NFE2L2, NOS3, PPARG, PTGS2]
GO:0009408	response to heat	GO_BP	7.95E-09	1.66E-08	6.35	8.00	[AKT1, GSK3B, HMOX1, HSP90AA1, IGF1, MTOR, NOS3, PTGS2]
GO:0045429	positive regulation of nitric oxide biosynthetic process	GO_BP	8.32E-09	1.73E-08	13.04	6.00	[AGT, AKT1, HSP90AA1, IL1B, PTGS2, TLR4]
GO:0010828	positive regulation of glucose transmembrane transport	GO_BP	8.32E-09	1.73E-08	13.04	6.00	[AKT1, IGF1, INS, IRS1, MAPK14, NFE2L2]
GO:0010827	regulation of glucose transmembrane transport	GO_BP	8.42E-09	1.74E-08	8.64	7.00	[AKT1, IGF1, IL1B, INS, IRS1, MAPK14, NFE2L2]
GO:0051170	import into nucleus	GO_BP	8.60E-09	1.78E-08	4.89	9.00	[AGT, AKT1, CDH1, CDK1, HSP90AA1, MAPK14, PTGS2, STAT3, TP53]
GO:0060965	negative regulation of miRNA-mediated gene silencing	GO_BP	1.05E-08	2.15E-08	21.74	5.00	[ESR1, IL6, PPARG, STAT3, TP53]
GO:1904407	positive regulation of nitric	GO_BP	1.08E-	2.21E-08	12.50	6.00	[AGT, AKT1, HSP90AA1, IL1B, PTGS2, TLR4]

	oxide metabolic process		08				
GO:0016441	post-transcriptional gene silencing	GO_BP	1.09E-08	2.22E-08	8.33	7.00	[EGFR, ESR1, IL6, MAP2K1, PPARG, STAT3, TP53]
GO:0031047	gene silencing by RNA	GO_BP	1.08E-08	2.22E-08	6.11	8.00	[EGFR, ESR1, IL6, MAP2K1, PPARG, SMAD2, STAT3, TP53]
GO:0046660	female sex differentiation	GO_BP	1.22E-08	2.47E-08	6.02	8.00	[BCL2L1, CASP3, CYP19A1, ESR1, KDR, MMP2, NOS3, SRC]
GO:1902895	positive regulation of miRNA transcription	GO_BP	1.23E-08	2.49E-08	12.24	6.00	[FOS, JUN, PPARG, RELA, STAT3, TP53]
GO:1904019	epithelial cell apoptotic process	GO_BP	1.29E-08	2.60E-08	5.97	8.00	[BCL2L1, CASP3, CASP9, ESR1, HMOX1, IL6, KDR, NFE2L2]
GO:1900369	negative regulation of post-transcriptional gene silencing by RNA	GO_BP	1.32E-08	2.64E-08	20.83	5.00	[ESR1, IL6, PPARG, STAT3, TP53]
GO:0070412	R-SMAD binding	GO_MF	1.32E-08	2.64E-08	20.83	5.00	[FOS, JUN, PARP1, PPARG, SMAD2]
GO:0060967	negative regulation of gene silencing by RNA	GO_BP	1.32E-08	2.64E-08	20.83	5.00	[ESR1, IL6, PPARG, STAT3, TP53]
GO:0060149	negative regulation of post-transcriptional gene silencing	GO_BP	1.32E-08	2.64E-08	20.83	5.00	[ESR1, IL6, PPARG, STAT3, TP53]
GO:2000278	regulation of DNA biosynthetic process	GO_BP	1.63E-08	3.26E-08	5.80	8.00	[CHEK1, HSP90AA1, IL6, MAPK1, MAPK3, PPARG, SRC, TP53]
GO:0002673	regulation of acute inflammatory response	GO_BP	1.78E-08	3.55E-08	11.54	6.00	[GSTP1, IL1B, IL6, INS, PPARG, PTGS2]
GO:0071482	cellular response to light stimulus	GO_BP	1.82E-08	3.62E-08	5.71	8.00	[CASP9, CHEK1, MMP2, MMP3, MMP9, PARP1, PTGS2, TP53]
GO:0090199	regulation of release of cytochrome c from mitochondria	GO_BP	2.00E-08	3.97E-08	11.32	6.00	[AKT1, BCL2L1, IGF1, IL6, MMP9, TP53]

GO:0010586	miRNA metabolic process	GO_BP	2.06E-08	4.07E-08	7.61	7.00	[FOS, JUN, NFKB1, PPARG, RELA, STAT3, TP53]
GO:0070555	response to interleukin-1	GO_BP	2.27E-08	4.47E-08	5.56	8.00	[APP, IL1B, IL6, MAPK3, MMP2, NFKB1, RELA, SRC]
GO:2000637	positive regulation of miRNA-mediated gene silencing	GO_BP	2.48E-08	4.86E-08	18.52	5.00	[EGFR, IL6, MAP2K1, STAT3, TP53]
GO:0031334	positive regulation of protein-containing complex assembly	GO_BP	2.50E-08	4.88E-08	4.33	9.00	[ESR1, GSK3B, HSP90AA1, MAPK9, MMP3, MTOR, SRC, TLR4, TP53]
GO:0051972	regulation of telomerase activity	GO_BP	2.52E-08	4.90E-08	10.91	6.00	[HSP90AA1, MAPK1, MAPK3, PPARG, SRC, TP53]
GO:0043620	regulation of DNA-templated transcription in response to stress	GO_BP	2.52E-08	4.90E-08	10.91	6.00	[CHEK1, HMOX1, JUN, NFE2L2, RELA, TP53]
GO:0043393	regulation of protein binding	GO_BP	2.83E-08	5.45E-08	4.27	9.00	[AKT1, APP, AURKA, CDK5, GSK3B, MAPK3, MAPK8, MMP9, SRC]
KEGG:04072	Phospholipase D signaling pathway	KEGG	2.82E-08	5.45E-08	5.41	8.00	[AGT, AKT1, EGFR, INS, MAP2K1, MAPK1, MAPK3, MTOR]
GO:0043525	positive regulation of neuron apoptotic process	GO_BP	2.81E-08	5.45E-08	10.71	6.00	[CASP3, CASP9, CDK5, MCL1, NQO1, TP53]
GO:0010507	negative regulation of autophagy	GO_BP	2.99E-08	5.74E-08	7.22	7.00	[AKT1, HMOX1, IL6, MCL1, MTOR, STAT3, TP53]
GO:1900370	positive regulation of post-transcriptional gene silencing by RNA	GO_BP	3.01E-08	5.77E-08	17.86	5.00	[EGFR, IL6, MAP2K1, STAT3, TP53]
GO:0060148	positive regulation of post-transcriptional gene silencing	GO_BP	3.01E-08	5.77E-08	17.86	5.00	[EGFR, IL6, MAP2K1, STAT3, TP53]

GO:0035094	response to nicotine	GO_BP	3.13E-08	5.98E-08	10.53	6.00	[CASP3, HMOX1, MAPK1, MMP2, NFKB1, RELA]
GO:0043470	regulation of carbohydrate catabolic process	GO_BP	3.49E-08	6.63E-08	10.34	6.00	[APP, IGF1, INS, MTOR, STAT3, TP53]
GO:0010823	negative regulation of mitochondrion organization	GO_BP	3.49E-08	6.63E-08	10.34	6.00	[AKT1, BCL2L1, IGF1, IL6, PPARG, TP53]
GO:1904385	cellular response to angiotensin	GO_BP	3.63E-08	6.89E-08	17.24	5.00	[AGT, NFE2L2, NFKB1, RELA, SRC]
GO:0010971	positive regulation of G2/M transition of mitotic cell cycle	GO_BP	3.63E-08	6.89E-08	17.24	5.00	[APP, CCNB1, CCND1, CDK1, CDK4]
GO:1900542	regulation of purine nucleotide metabolic process	GO_BP	3.69E-08	6.98E-08	7.00	7.00	[APP, IGF1, INS, MTOR, NOS3, PARP1, STAT3]
GO:0010676	positive regulation of cellular carbohydrate metabolic process	GO_BP	3.87E-08	7.29E-08	10.17	6.00	[AKT1, FOXO1, IGF1, INS, IRS1, SRC]
GO:0003720	telomerase activity	GO_BP	3.87E-08	7.29E-08	10.17	6.00	[HSP90AA1, MAPK1, MAPK3, PPARG, SRC, TP53]
GO:0007179	transforming growth factor beta receptor signaling pathway	GO_BP	4.05E-08	7.61E-08	4.09	9.00	[FOS, JUN, PARP1, PPARG, PTK2, SMAD2, SRC, STAT3, TP53]
GO:0006140	regulation of nucleotide metabolic process	GO_BP	4.24E-08	7.93E-08	6.86	7.00	[APP, IGF1, INS, MTOR, NOS3, PARP1, STAT3]
GO:0046324	regulation of glucose import	GO_BP	4.29E-08	8.01E-08	10.00	6.00	[AKT1, IGF1, INS, IRS1, MAPK14, NFE2L2]
GO:0010962	regulation of glucan biosynthetic process	GO_BP	4.35E-08	8.09E-08	16.67	5.00	[AKT1, GSK3B, IGF1, INS, IRS1]
GO:0005979	regulation of glycogen biosynthetic process	GO_BP	4.35E-08	8.09E-08	16.67	5.00	[AKT1, GSK3B, IGF1, INS, IRS1]
GO:0045732	positive regulation of protein catabolic process	GO_BP	4.38E-08	8.12E-08	4.05	9.00	[AKT1, AURKA, FOXO1, GSK3B, HSP90AA1, IL1B, KEAP1, MAPK9, NFE2L2]

GO:0090398	cellular senescence	GO_BP	4.54E-08	8.38E-08	6.80	7.00	[CDK2, CDK6, MAP2K1, MAPK14, MAPK8, MAPK9, TP53]
GO:0042594	response to starvation	GO_BP	4.73E-08	8.71E-08	4.02	9.00	[FOXO1, HSPA8, MAPK1, MAPK3, MAPK8, MTOR, NFE2L2, PPARG, TP53]
KEGG:04064	NF-kappa B signaling pathway	KEGG	4.85E-08	8.91E-08	6.73	7.00	[BCL2L1, IL1B, NFKB1, PARP1, PTGS2, RELA, TLR4]
GO:0044703	multi-organism reproductive process	GO_BP	4.91E-08	9.00E-08	4.00	9.00	[AGT, AKT1, CYP1A1, ESR1, FOS, IL1B, MMP2, MMP9, PTGS2]
GO:1903800	positive regulation of miRNA maturation	GO_BP	5.12E-08	9.35E-08	33.33	4.00	[EGFR, IL6, MAP2K1, TP53]
GO:0008353	RNA polymerase II CTD heptapeptide repeat kinase activity	GO_MF	5.12E-08	9.35E-08	33.33	4.00	[CDK1, CDK4, CDK6, MAPK1]
KEGG:04630	JAK-STAT signaling pathway	KEGG	5.70E-08	1.04E-07	4.94	8.00	[AKT1, BCL2L1, CCND1, EGFR, IL6, MCL1, MTOR, STAT3]
KEGG:04215	Apoptosis	KEGG	6.11E-08	1.11E-07	15.63	5.00	[BCL2L1, CASP3, CASP9, MAPK8, MAPK9]
GO:1902751	positive regulation of cell cycle G2/M phase transition	GO_BP	6.11E-08	1.11E-07	15.63	5.00	[APP, CCNB1, CCND1, CDK1, CDK4]
GO:0001836	release of cytochrome c from mitochondria	GO_BP	6.36E-08	1.15E-07	9.38	6.00	[AKT1, BCL2L1, IGF1, IL6, MMP9, TP53]
GO:0051092	positive regulation of NF-kappaB transcription factor activity	GO_BP	6.57E-08	1.18E-07	4.85	8.00	[AGT, APP, IL1B, IL6, INS, RELA, STAT3, TLR4]
GO:0045428	regulation of nitric oxide biosynthetic process	GO_BP	6.99E-08	1.26E-07	9.23	6.00	[AGT, AKT1, HSP90AA1, IL1B, PTGS2, TLR4]
GO:0043255	regulation of carbohydrate biosynthetic process	GO_BP	7.15E-08	1.28E-07	6.36	7.00	[AKT1, FOXO1, GSK3B, IGF1, INS, IRS1, NFKB1]
GO:1902175	regulation of oxidative stress-induced intrinsic apoptotic signaling pathway	GO_BP	7.19E-08	1.28E-07	15.15	5.00	[AKT1, INS, MCL1, NFE2L2, PARP1]

GO:1902042	negative regulation of extrinsic apoptotic signaling pathway via death domain receptors	GO_BP	7.19E-08	1.28E-07	15.15	5.00	[BCL2L1, GSK3B, HMOX1, IL6, NOS3]
GO:1902893	regulation of miRNA transcription	GO_BP	8.40E-08	1.50E-07	8.96	6.00	[FOS, JUN, PPARG, RELA, STAT3, TP53]
GO:0061614	miRNA transcription	GO_BP	8.40E-08	1.50E-07	8.96	6.00	[FOS, JUN, PPARG, RELA, STAT3, TP53]
GO:0046626	regulation of insulin receptor signaling pathway	GO_BP	8.40E-08	1.50E-07	8.96	6.00	[AGT, CDK4, IL1B, INS, IRS1, SRC]
GO:0080164	regulation of nitric oxide metabolic process	GO_BP	9.19E-08	1.63E-07	8.82	6.00	[AGT, AKT1, HSP90AA1, IL1B, PTGS2, TLR4]
GO:0003964	RNA-directed DNA polymerase activity	GO_BP	9.19E-08	1.63E-07	8.82	6.00	[HSP90AA1, MAPK1, MAPK3, PPARG, SRC, TP53]
GO:0010906	regulation of glucose metabolic process	GO_BP	9.73E-08	1.72E-07	6.09	7.00	[AKT1, FOXO1, IGF1, INS, IRS1, SRC, TP53]
GO:0071354	cellular response to interleukin-6	GO_BP	9.78E-08	1.73E-07	14.29	5.00	[IL6, NFKB1, RELA, SRC, STAT3]
GO:2000060	positive regulation of ubiquitin-dependent protein catabolic process	GO_BP	1.03E-07	1.82E-07	6.03	7.00	[AKT1, AURKA, GSK3B, KEAP1, MAPK9, NFE2L2, PTK2]
GO:1900076	regulation of cellular response to insulin stimulus	GO_BP	1.10E-07	1.92E-07	8.57	6.00	[AGT, IL1B, INS, IRS1, PPARG, SRC]
GO:0070873	regulation of glycogen metabolic process	GO_BP	1.13E-07	1.98E-07	13.89	5.00	[AKT1, GSK3B, IGF1, INS, IRS1]
GO:0070665	positive regulation of leukocyte proliferation	GO_BP	1.13E-07	1.98E-07	4.52	8.00	[BCL2L1, IGF1, IL1B, IL6, MAPK1, MAPK3, PTK2, TLR4]
GO:0009267	cellular response to starvation	GO_BP	1.13E-07	1.98E-07	4.52	8.00	[FOXO1, HSPA8, MAPK1, MAPK3, MAPK8, MTOR, NFE2L2, TP53]
GO:1904659	glucose transmembrane transport	GO_BP	1.16E-07	2.02E-07	5.93	7.00	[AKT1, IGF1, IL1B, INS, IRS1, MAPK14, NFE2L2]

KEGG:04520	Adherens junction	KEGG	1.19E-07	2.07E-07	8.45	6.00	[CDH1, EGFR, ERBB2, MAPK1, MAPK3, SRC]
GO:0010660	regulation of muscle cell apoptotic process	GO_BP	1.19E-07	2.07E-07	8.45	6.00	[AGT, HMOX1, IGF1, NFE2L2, PPARG, TP53]
KEGG:04960	Aldosterone-regulated sodium reabsorption	KEGG	1.31E-07	2.26E-07	13.51	5.00	[IGF1, INS, IRS1, MAPK1, MAPK3]
GO:0097718	disordered domain specific binding	GO_MF	1.31E-07	2.26E-07	13.51	5.00	[FN1, HSP90AA1, KEAP1, SMAD2, TP53]
GO:0043276	anoikis	GO_BP	1.31E-07	2.26E-07	13.51	5.00	[AKT1, MCL1, MTOR, PTK2, SRC]
GO:0090170	regulation of Golgi inheritance	GO_BP	1.35E-07	2.31E-07	75.00	3.00	[MAP2K1, MAPK1, MAPK3]
GO:2000058	regulation of ubiquitin-dependent protein catabolic process	GO_BP	1.34E-07	2.31E-07	4.42	8.00	[AKT1, AURKA, CDK2, GSK3B, KEAP1, MAPK9, NFE2L2, PTK2]
GO:0035265	organ growth	GO_BP	1.34E-07	2.31E-07	4.42	8.00	[AGT, AKT1, CDK1, CYP19A1, ESR1, IGF1, MAPK14, SMAD2]
GO:0008645	hexose transmembrane transport	GO_BP	1.38E-07	2.36E-07	5.79	7.00	[AKT1, IGF1, IL1B, INS, IRS1, MAPK14, NFE2L2]
GO:0000079	regulation of cyclin-dependent protein serine/threonine kinase activity	GO_BP	1.38E-07	2.36E-07	5.79	7.00	[AKT1, CASP3, CCNA2, CCNB1, CCND1, EGFR, SRC]
GO:0070741	response to interleukin-6	GO_BP	1.50E-07	2.56E-07	13.16	5.00	[IL6, NFKB1, RELA, SRC, STAT3]
GO:1903578	regulation of ATP metabolic process	GO_BP	1.53E-07	2.61E-07	8.11	6.00	[APP, IGF1, INS, MTOR, PARP1, STAT3]
GO:0043200	response to amino acid	GO_BP	1.54E-07	2.62E-07	5.69	7.00	[BCL2L1, CASP3, EGFR, INS, MMP2, MTOR, NQO1]
GO:0042752	regulation of circadian rhythm	GO_BP	1.54E-07	2.62E-07	5.69	7.00	[CDK1, GSK3B, MAPK8, MAPK9, MTOR, PPARG, TP53]

GO:1904029	regulation of cyclin-dependent protein kinase activity	GO_BP	1.63E-07	2.76E-07	5.65	7.00	[AKT1, CASP3, CCNA2, CCNB1, CCND1, EGFR, SRC]
GO:0015749	monosaccharide transmembrane transport	GO_BP	1.63E-07	2.76E-07	5.65	7.00	[AKT1, IGF1, IL1B, INS, IRS1, MAPK14, NFE2L2]
GO:0046890	regulation of lipid biosynthetic process	GO_BP	1.66E-07	2.79E-07	4.30	8.00	[AKT1, CCNA2, CDK4, IL1B, INS, MTOR, NFKB1, PTGS2]
GO:2000379	positive regulation of reactive oxygen species metabolic process	GO_BP	1.66E-07	2.80E-07	8.00	6.00	[AGT, GSTP1, MAPK14, NFE2L2, TLR4, TP53]
GO:0021782	glial cell development	GO_BP	1.82E-07	3.06E-07	5.56	7.00	[AKT1, APP, CDK5, CDK6, IL1B, IL6, TLR4]
GO:0048313	Golgi inheritance	GO_BP	1.87E-07	3.12E-07	25.00	4.00	[CDK1, MAP2K1, MAPK1, MAPK3]
GO:0048308	organelle inheritance	GO_BP	1.87E-07	3.12E-07	25.00	4.00	[CDK1, MAP2K1, MAPK1, MAPK3]
GO:0046323	glucose import	GO_BP	1.95E-07	3.25E-07	7.79	6.00	[AKT1, IGF1, INS, IRS1, MAPK14, NFE2L2]
GO:0051101	regulation of DNA binding	GO_BP	2.03E-07	3.37E-07	5.47	7.00	[HMOX1, IGF1, JUN, MAPK8, MMP9, PARP1, PPARG]
GO:0031960	response to corticosteroid	GO_BP	2.03E-07	3.38E-07	4.19	8.00	[CASP3, CASP9, CCND1, FOS, IL6, PARP1, PTGS2, SRC]
GO:0010822	positive regulation of mitochondrion organization	GO_BP	2.10E-07	3.48E-07	7.69	6.00	[AURKA, GSK3B, KDR, MAPK8, MMP9, TP53]
GO:0000271	polysaccharide biosynthetic process	GO_BP	2.10E-07	3.48E-07	7.69	6.00	[AKT1, GSK3B, IGF1, INS, IRS1, NFKB1]
GO:1903799	negative regulation of miRNA maturation	GO_BP	2.43E-07	4.01E-07	23.53	4.00	[ESR1, IL6, STAT3, TP53]
GO:0045725	positive regulation of glycogen biosynthetic process	GO_BP	2.43E-07	4.01E-07	23.53	4.00	[AKT1, IGF1, INS, IRS1]

GO:0034349	glial cell apoptotic process	GO_BP	2.43E-07	4.01E-07	23.53	4.00	[CASP3, CASP9, CDK5, TP53]
GO:0042307	positive regulation of protein import into nucleus	GO_BP	2.84E-07	4.68E-07	11.63	5.00	[CDH1, CDK1, HSP90AA1, MAPK14, PTGS2]
GO:0070875	positive regulation of glycogen metabolic process	GO_BP	3.12E-07	5.12E-07	22.22	4.00	[AKT1, IGF1, INS, IRS1]
GO:0035173	histone kinase activity	GO_BP	3.12E-07	5.12E-07	22.22	4.00	[AURKA, CDK1, CDK2, CHEK1]
GO:0120041	positive regulation of macrophage proliferation	GO_BP	3.36E-07	5.49E-07	60.00	3.00	[MAPK1, MAPK3, PTK2]
GO:0014805	smooth muscle adaptation	GO_BP	3.36E-07	5.49E-07	60.00	3.00	[HMOX1, IL1B, NOS3]
GO:0002526	acute inflammatory response	GO_BP	3.39E-07	5.53E-07	5.07	7.00	[FN1, GSTP1, IL1B, IL6, INS, PPARG, PTGS2]
GO:0043627	response to estrogen	GO_BP	3.52E-07	5.72E-07	7.06	6.00	[CCND1, ESR1, HMOX1, HSP90AA1, MMP2, PPARG]
GO:0071392	cellular response to estradiol stimulus	GO_BP	3.59E-07	5.82E-07	11.11	5.00	[CCNA2, EGFR, ESR1, ESR2, MMP2]
GO:0034219	carbohydrate transmembrane transport	GO_BP	3.74E-07	6.05E-07	5.00	7.00	[AKT1, IGF1, IL1B, INS, IRS1, MAPK14, NFE2L2]
GO:0001101	response to acid chemical	GO_BP	3.74E-07	6.05E-07	5.00	7.00	[BCL2L1, CASP3, EGFR, INS, MMP2, MTOR, NQO1]
GO:0043502	regulation of muscle adaptation	GO_BP	3.77E-07	6.08E-07	6.98	6.00	[AGT, FOXO1, IGF1, NOS3, PARP1, PPARG]
GO:0071732	cellular response to nitric oxide	GO_BP	3.94E-07	6.34E-07	21.05	4.00	[CCNA2, CDK2, FOXO1, MMP3]
GO:0004708	MAP kinase kinase activity	GO_BP	3.94E-07	6.34E-07	21.05	4.00	[MAP2K1, MAPK1, MAPK14, MAPK3]
GO:0014002	astrocyte development	GO_BP	4.02E-07	6.44E-07	10.87	5.00	[APP, CDK6, IL1B, IL6, TLR4]

GO:0009250	glucan biosynthetic process	GO_BP	4.02E-07	6.44E-07	10.87	5.00	[AKT1, GSK3B, IGF1, INS, IRS1]
GO:0005978	glycogen biosynthetic process	GO_BP	4.02E-07	6.44E-07	10.87	5.00	[AKT1, GSK3B, IGF1, INS, IRS1]
KEGG:04540	Gap junction	KEGG	4.32E-07	6.90E-07	6.82	6.00	[CDK1, EGFR, MAP2K1, MAPK1, MAPK3, SRC]
GO:0045598	regulation of fat cell differentiation	GO_BP	4.32E-07	6.91E-07	4.90	7.00	[AKT1, FOXO1, IL6, INS, MAPK14, PPARG, PTGS2]
GO:1903052	positive regulation of proteolysis involved in protein catabolic process	GO_BP	4.53E-07	7.20E-07	4.86	7.00	[AKT1, AURKA, GSK3B, KEAP1, MAPK9, NFE2L2, PTK2]
GO:2000641	regulation of early endosome to late endosome transport	GO_BP	4.92E-07	7.80E-07	20.00	4.00	[MAP2K1, MAPK1, MAPK3, SRC]
GO:0071498	cellular response to fluid shear stress	GO_BP	4.92E-07	7.80E-07	20.00	4.00	[MMP2, NFE2L2, PTGS2, SRC]
GO:0006112	energy reserve metabolic process	GO_BP	5.28E-07	8.35E-07	6.59	6.00	[AKT1, GSK3B, IGF1, INS, IRS1, MTOR]
GO:0032434	regulation of proteasomal ubiquitin-dependent protein catabolic process	GO_BP	5.45E-07	8.60E-07	4.73	7.00	[AKT1, AURKA, CDK2, GSK3B, KEAP1, MAPK9, NFE2L2]
GO:0070849	response to epidermal growth factor	GO_BP	5.55E-07	8.73E-07	10.20	5.00	[AKT1, EGFR, ERBB2, MAPK1, MAPK3]
GO:0043618	regulation of transcription from RNA polymerase II promoter in response to stress	GO_BP	5.55E-07	8.73E-07	10.20	5.00	[CHEK1, HMOX1, JUN, NFE2L2, TP53]
KEGG:05323	Rheumatoid arthritis	KEGG	6.01E-07	9.43E-07	6.45	6.00	[FOS, IL1B, IL6, JUN, MMP3, TLR4]
GO:1902170	cellular response to reactive nitrogen species	GO_BP	6.06E-07	9.49E-07	19.05	4.00	[CCNA2, CDK2, FOXO1, MMP3]
GO:0008631	intrinsic apoptotic signaling pathway in response to	GO_BP	6.15E-	9.60E-07	10.00	5.00	[AKT1, INS, MCL1, NFE2L2, PARP1]

	oxidative stress		07				
GO:0006110	regulation of glycolytic process	GO_BP	6.15E-07	9.60E-07	10.00	5.00	[APP, IGF1, INS, MTOR, STAT3]
GO:0120040	regulation of macrophage proliferation	GO_BP	6.70E-07	1.04E-06	50.00	3.00	[MAPK1, MAPK3, PTK2]
GO:0035051	cardiocyte differentiation	GO_BP	6.82E-07	1.05E-06	4.58	7.00	[AGT, CDK1, EGFR, IGF1, MAPK1, MAPK3, MTOR]
GO:0046889	positive regulation of lipid biosynthetic process	GO_BP	6.81E-07	1.06E-06	6.32	6.00	[AKT1, CCNA2, IL1B, INS, MTOR, PTGS2]
KEGG:04913	Ovarian steroidogenesis	KEGG	6.80E-07	1.06E-06	9.80	5.00	[CYP19A1, CYP1A1, IGF1, INS, PTGS2]
GO:0045821	positive regulation of glycolytic process	GO_BP	7.39E-07	1.14E-06	18.18	4.00	[APP, IGF1, INS, MTOR]
GO:0038083	peptidyl-tyrosine autophosphorylation	GO_BP	7.39E-07	1.14E-06	18.18	4.00	[EGFR, KDR, MAPK3, SRC]
GO:0005158	insulin receptor binding	GO_MF	7.39E-07	1.14E-06	18.18	4.00	[IGF1, INS, IRS1, SRC]
GO:1900544	positive regulation of purine nucleotide metabolic process	GO_BP	7.50E-07	1.15E-06	9.62	5.00	[APP, IGF1, INS, MTOR, NOS3]
GO:0046677	response to antibiotic	GO_BP	7.50E-07	1.15E-06	9.62	5.00	[CASP3, CASP9, CYP1A1, HSP90AA1, TP53]
GO:0045981	positive regulation of nucleotide metabolic process	GO_BP	7.50E-07	1.15E-06	9.62	5.00	[APP, IGF1, INS, MTOR, NOS3]
GO:0045737	positive regulation of cyclin-dependent protein serine/threonine kinase activity	GO_BP	7.50E-07	1.15E-06	9.62	5.00	[AKT1, CCNB1, CCND1, EGFR, SRC]
GO:0016538	cyclin-dependent protein serine/threonine kinase regulator activity	GO_BP	7.50E-07	1.15E-06	9.62	5.00	[CASP3, CCNA2, CCNB1, CCND1, CDK4]

GO:0019217	regulation of fatty acid metabolic process	GO_BP	7.71E-07	1.18E-06	6.19	6.00	[AKT1, IL1B, INS, IRS1, PPARG, PTGS2]
KEGG:04750	Inflammatory mediator regulation of TRP channels	KEGG	8.19E-07	1.25E-06	6.12	6.00	[IGF1, IL1B, MAPK14, MAPK8, MAPK9, SRC]
GO:0070301	cellular response to hydrogen peroxide	GO_BP	8.69E-07	1.33E-06	6.06	6.00	[CDK1, IL6, NFE2L2, NQO1, RELA, SRC]
GO:0032436	positive regulation of proteasomal ubiquitin-dependent protein catabolic process	GO_BP	8.69E-07	1.33E-06	6.06	6.00	[AKT1, AURKA, GSK3B, KEAP1, MAPK9, NFE2L2]
KEGG:04217	Necroptosis	KEGG	8.84E-07	1.34E-06	4.40	7.00	[HSP90AA1, IL1B, MAPK8, MAPK9, PARP1, STAT3, TLR4]
GO:0030879	mammary gland development	GO_BP	8.84E-07	1.34E-06	4.40	7.00	[AKT1, CCND1, CYP19A1, ESR1, MAPK1, NFKB1, SRC]
GO:0046628	positive regulation of insulin receptor signaling pathway	GO_BP	8.92E-07	1.35E-06	17.39	4.00	[AGT, INS, IRS1, SRC]
GO:0020037	heme binding	GO_MF	9.22E-07	1.40E-06	4.38	7.00	[CYP19A1, CYP1A1, HMOX1, NFE2L2, NOS3, PTGS2, SRC]
GO:0097194	execution phase of apoptosis	GO_BP	9.78E-07	1.48E-06	5.94	6.00	[AKT1, BCL2L1, CASP3, CASP9, IL6, TP53]
GO:0032642	regulation of chemokine production	GO_BP	9.78E-07	1.48E-06	5.94	6.00	[APP, GSTP1, HMOX1, IL1B, IL6, TLR4]
GO:0032602	chemokine production	GO_BP	9.78E-07	1.48E-06	5.94	6.00	[APP, GSTP1, HMOX1, IL1B, IL6, TLR4]
GO:0001889	liver development	GO_BP	1.00E-06	1.51E-06	4.32	7.00	[AURKA, CCND1, CYP1A1, HMOX1, IL6, JUN, RELA]
GO:0071731	response to nitric oxide	GO_BP	1.07E-06	1.60E-06	16.67	4.00	[CCNA2, CDK2, FOXO1, MMP3]
GO:0008643	carbohydrate transport	GO_BP	1.09E-06	1.63E-06	4.27	7.00	[AKT1, IGF1, IL1B, INS, IRS1, MAPK14, NFE2L2]
GO:1904031	positive regulation of cyclin-dependent protein kinase	GO_BP	1.09E-	1.63E-06	8.93	5.00	[AKT1, CCNB1, CCND1, EGFR, SRC]

	activity		06				
GO:0032651	regulation of interleukin-1 beta production	GO_BP	1.10E-06	1.64E-06	5.83	6.00	[APP, GSTP1, IGF1, IL6, STAT3, TLR4]
GO:0032611	interleukin-1 beta production	GO_BP	1.10E-06	1.64E-06	5.83	6.00	[APP, GSTP1, IGF1, IL6, STAT3, TLR4]
GO:0061008	hepaticobiliary system development	GO_BP	1.13E-06	1.69E-06	4.24	7.00	[AURKA, CCND1, CYP1A1, HMOX1, IL6, JUN, RELA]
GO:0060440	trachea formation	GO_BP	1.17E-06	1.73E-06	42.86	3.00	[MAP2K1, MAPK1, MAPK3]
GO:1902041	regulation of extrinsic apoptotic signaling pathway via death domain receptors	GO_BP	1.19E-06	1.76E-06	8.77	5.00	[BCL2L1, GSK3B, HMOX1, IL6, NOS3]
GO:0048010	vascular endothelial growth factor receptor signaling pathway	GO_BP	1.19E-06	1.76E-06	8.77	5.00	[IL1B, KDR, MAPK14, PTK2, SRC]
KEGG:04022	cGMP-PKG signaling pathway	KEGG	1.23E-06	1.81E-06	4.19	7.00	[AKT1, INS, IRS1, MAP2K1, MAPK1, MAPK3, NOS3]
GO:0005178	integrin binding	GO_MF	1.28E-06	1.88E-06	4.17	7.00	[CCNA2, FN1, IGF1, IL1B, KDR, PTK2, SRC]
GO:0032768	regulation of monooxygenase activity	GO_BP	1.30E-06	1.91E-06	8.62	5.00	[AKT1, EGFR, IL1B, INS, NFKB1]
GO:0022602	ovulation cycle process	GO_BP	1.30E-06	1.91E-06	8.62	5.00	[CASP3, ESR1, MMP2, NOS3, SRC]
GO:0010611	regulation of cardiac muscle hypertrophy	GO_BP	1.30E-06	1.91E-06	8.62	5.00	[AGT, FOXO1, IGF1, PARP1, PPARG]
GO:0006278	RNA-templated DNA biosynthetic process	GO_BP	1.30E-06	1.91E-06	5.66	6.00	[HSP90AA1, MAPK1, MAPK3, PPARG, SRC, TP53]
KEGG:04530	Tight junction	KEGG	1.33E-06	1.94E-06	4.14	7.00	[CCND1, CDK4, ERBB2, JUN, MAPK8, MAPK9, SRC]
GO:0046906	tetrapyrrole binding	GO_MF	1.38E-06	2.02E-06	4.12	7.00	[CYP19A1, CYP1A1, HMOX1, NFE2L2, NOS3, PTGS2, SRC]

GO:0140296	general transcription initiation factor binding	GO_MF	1.42E-06	2.06E-06	8.47	5.00	[ESR1, JUN, MTOR, RELA, TP53]
GO:0051100	negative regulation of binding	GO_BP	1.44E-06	2.09E-06	4.09	7.00	[AKT1, AURKA, GSK3B, HMOX1, JUN, MAPK3, MAPK8]
GO:0062014	negative regulation of small molecule metabolic process	GO_BP	1.45E-06	2.10E-06	5.56	6.00	[AKT1, INS, NFKB1, PARP1, STAT3, TP53]
GO:0034061	DNA polymerase activity	GO_BP	1.45E-06	2.10E-06	5.56	6.00	[HSP90AA1, MAPK1, MAPK3, PPARG, SRC, TP53]
GO:0090201	negative regulation of release of cytochrome c from mitochondria	GO_BP	1.50E-06	2.16E-06	15.38	4.00	[AKT1, BCL2L1, IGF1, IL6]
GO:0030307	positive regulation of cell growth	GO_BP	1.62E-06	2.33E-06	4.02	7.00	[AKT1, EGFR, ERBB2, FN1, IGF1, INS, MTOR]
GO:0014743	regulation of muscle hypertrophy	GO_BP	1.68E-06	2.41E-06	8.20	5.00	[AGT, FOXO1, IGF1, PARP1, PPARG]
GO:0051341	regulation of oxidoreductase activity	GO_BP	1.70E-06	2.44E-06	5.41	6.00	[AGT, AKT1, EGFR, IL1B, INS, NFKB1]
GO:1900078	positive regulation of cellular response to insulin stimulus	GO_BP	1.75E-06	2.50E-06	14.81	4.00	[AGT, INS, IRS1, SRC]
GO:0001541	ovarian follicle development	GO_BP	1.82E-06	2.59E-06	8.06	5.00	[BCL2L1, ESR1, KDR, MMP2, SRC]
GO:0098531	ligand-activated transcription factor activity	GO_BP	1.97E-06	2.80E-06	7.94	5.00	[CYP1A1, ESR1, ESR2, PPARG, STAT3]
GO:0004879	nuclear receptor activity	GO_BP	1.97E-06	2.80E-06	7.94	5.00	[CYP1A1, ESR1, ESR2, PPARG, STAT3]
GO:0001706	endoderm formation	GO_BP	1.97E-06	2.80E-06	7.94	5.00	[FN1, MAP2K1, MMP2, MMP9, SMAD2]
GO:0070498	interleukin-1-mediated signaling pathway	GO_BP	2.04E-06	2.89E-06	14.29	4.00	[IL1B, IL6, MAPK3, RELA]
GO:0055093	response to hyperoxia	GO_BP	2.04E-	2.89E-06	14.29	4.00	[CYP1A1, FOXO1, MMP2, PPARG]

			06				
KEGG:04726	Serotonergic synapse	KEGG	2.10E-06	2.96E-06	5.22	6.00	[APP, CASP3, MAP2K1, MAPK1, MAPK3, PTGS2]
GO:0071347	cellular response to interleukin-1	GO_BP	2.10E-06	2.96E-06	5.22	6.00	[IL1B, IL6, MAPK3, MMP2, NFKB1, RELA]
KEGG:04929	GnRH secretion	KEGG	2.14E-06	3.01E-06	7.81	5.00	[AKT1, ESR2, MAP2K1, MAPK1, MAPK3]
GO:0005976	polysaccharide metabolic process	GO_BP	2.32E-06	3.26E-06	5.13	6.00	[AKT1, GSK3B, IGF1, INS, IRS1, NFKB1]
GO:1901522	positive regulation of transcription from RNA polymerase II promoter involved in cellular response to chemical stimulus	GO_BP	2.36E-06	3.31E-06	13.79	4.00	[JUN, NFE2L2, RELA, TP53]
GO:0042306	regulation of protein import into nucleus	GO_BP	2.49E-06	3.49E-06	7.58	5.00	[CDH1, CDK1, HSP90AA1, MAPK14, PTGS2]
GO:0006953	acute-phase response	GO_BP	2.49E-06	3.49E-06	7.58	5.00	[FN1, IL1B, IL6, INS, PTGS2]
GO:0071901	negative regulation of protein serine/threonine kinase activity	GO_BP	2.69E-06	3.74E-06	5.00	6.00	[AGT, AKT1, CASP3, GSTP1, IL1B, PPARG]
GO:0050994	regulation of lipid catabolic process	GO_BP	2.68E-06	3.75E-06	7.46	5.00	[AKT1, CDK4, IL1B, INS, IRS1]
GO:0097421	liver regeneration	GO_BP	2.71E-06	3.77E-06	13.33	4.00	[AURKA, CCND1, HMOX1, IL6]
GO:0002246	wound healing involved in inflammatory response	GO_BP	2.79E-06	3.88E-06	33.33	3.00	[HMOX1, PPARG, TLR4]
GO:1901224	positive regulation of NIK/NF-kappaB signaling	GO_BP	2.89E-06	4.00E-06	7.35	5.00	[APP, EGFR, IL1B, RELA, TLR4]
GO:0097192	extrinsic apoptotic signaling pathway in absence of ligand	GO_BP	2.89E-06	4.00E-06	7.35	5.00	[AKT1, BCL2L1, GSK3B, IL1B, MCL1]

GO:0038034	signal transduction in absence of ligand	GO_BP	2.89E-06	4.00E-06	7.35	5.00	[AKT1, BCL2L1, GSK3B, IL1B, MCL1]
GO:1903649	regulation of cytoplasmic transport	GO_BP	3.11E-06	4.27E-06	12.90	4.00	[MAP2K1, MAPK1, MAPK3, SRC]
GO:0002360	T cell lineage commitment	GO_BP	3.11E-06	4.27E-06	12.90	4.00	[IL6, MTOR, STAT3, TP53]
KEGG:05211	Renal cell carcinoma	KEGG	3.11E-06	4.28E-06	7.25	5.00	[AKT1, JUN, MAP2K1, MAPK1, MAPK3]
GO:0014910	regulation of smooth muscle cell migration	GO_BP	3.11E-06	4.28E-06	7.25	5.00	[AGT, IGF1, NFE2L2, SRC, TLR4]
GO:0033138	positive regulation of peptidyl-serine phosphorylation	GO_BP	3.10E-06	4.28E-06	4.88	6.00	[AKT1, APP, EGFR, HSP90AA1, IL6, PTGS2]
GO:0032652	regulation of interleukin-1 production	GO_BP	3.10E-06	4.28E-06	4.88	6.00	[APP, GSTP1, IGF1, IL6, STAT3, TLR4]
GO:0032612	interleukin-1 production	GO_BP	3.10E-06	4.28E-06	4.88	6.00	[APP, GSTP1, IGF1, IL6, STAT3, TLR4]
GO:0007088	regulation of mitotic nuclear division	GO_BP	3.10E-06	4.28E-06	4.88	6.00	[AURKA, CCNB1, CHEK1, IGF1, IL1B, INS]
KEGG:04611	Platelet activation	KEGG	3.25E-06	4.46E-06	4.84	6.00	[AKT1, MAPK1, MAPK14, MAPK3, NOS3, SRC]
KEGG:04622	RIG-I-like receptor signaling pathway	KEGG	3.34E-06	4.57E-06	7.14	5.00	[MAPK14, MAPK8, MAPK9, NFKB1, RELA]
GO:0070542	response to fatty acid	GO_BP	3.34E-06	4.57E-06	7.14	5.00	[CDK4, FOXO1, IRS1, PTGS2, SRC]
GO:1901800	positive regulation of proteasomal protein catabolic process	GO_BP	3.41E-06	4.65E-06	4.80	6.00	[AKT1, AURKA, GSK3B, KEAP1, MAPK9, NFE2L2]
GO:0071887	leukocyte apoptotic process	GO_BP	3.41E-06	4.65E-06	4.80	6.00	[AKT1, BCL2L1, CASP3, CASP9, IL6, TP53]
GO:0051881	regulation of mitochondrial	GO_BP	3.58E-	4.88E-06	7.04	5.00	[AKT1, BCL2L1, KDR, PARP1, SRC]

	membrane potential		06				
GO:0033692	cellular polysaccharide biosynthetic process	GO_BP	3.58E-06	4.88E-06	7.04	5.00	[AKT1, GSK3B, IGF1, INS, IRS1]
GO:0032757	positive regulation of interleukin-8 production	GO_BP	3.84E-06	5.22E-06	6.94	5.00	[IL1B, IL6, RELA, STAT3, TLR4]
GO:0045600	positive regulation of fat cell differentiation	GO_BP	4.11E-06	5.57E-06	6.85	5.00	[AKT1, INS, MAPK14, PPARG, PTGS2]
GO:0034605	cellular response to heat	GO_BP	4.11E-06	5.57E-06	6.85	5.00	[GSK3B, HMOX1, HSP90AA1, MTOR, PTGS2]
GO:0030332	cyclin binding	GO_MF	4.55E-06	6.15E-06	11.76	4.00	[CDK1, CDK2, CDK4, CDK6]
KEGG:04728	Dopaminergic synapse	KEGG	4.67E-06	6.30E-06	4.55	6.00	[AKT1, FOS, GSK3B, MAPK14, MAPK8, MAPK9]
GO:0045667	regulation of osteoblast differentiation	GO_BP	4.67E-06	6.30E-06	4.55	6.00	[CCNA2, CDK6, IGF1, IL6, PPARG, PTK2]
GO:0042826	histone deacetylase binding	GO_MF	4.67E-06	6.30E-06	4.55	6.00	[CCND1, HSP90AA1, MAPK8, PARP1, RELA, TP53]
GO:0090559	regulation of membrane permeability	GO_BP	5.02E-06	6.75E-06	6.58	5.00	[BCL2L1, GSK3B, MAPK8, MTOR, TP53]
GO:0000077	DNA damage checkpoint signaling	GO_BP	5.09E-06	6.84E-06	4.48	6.00	[CCND1, CDK1, CDK2, CHEK1, MAPK14, TP53]
GO:2001240	negative regulation of extrinsic apoptotic signaling pathway in absence of ligand	GO_BP	5.12E-06	6.86E-06	11.43	4.00	[AKT1, BCL2L1, IL1B, MCL1]
GO:1901099	negative regulation of signal transduction in absence of ligand	GO_BP	5.12E-06	6.86E-06	11.43	4.00	[AKT1, BCL2L1, IL1B, MCL1]
GO:0050321	tau-protein kinase activity	GO_MF	5.12E-06	6.86E-06	11.43	4.00	[CDK5, GSK3B, HSP90AA1, IL6]
GO:0036296	response to increased oxygen levels	GO_BP	5.12E-06	6.86E-06	11.43	4.00	[CYP1A1, FOXO1, MMP2, PPARG]

GO:0010743	regulation of macrophage derived foam cell differentiation	GO_BP	5.12E-06	6.86E-06	11.43	4.00	[AGT, MAPK9, NFKB1, PPARG]
GO:0007263	nitric oxide mediated signal transduction	GO_BP	5.12E-06	6.86E-06	11.43	4.00	[AGT, EGFR, INS, NOS3]
GO:0032722	positive regulation of chemokine production	GO_BP	5.35E-06	7.16E-06	6.49	5.00	[APP, HMOX1, IL1B, IL6, TLR4]
GO:0014909	smooth muscle cell migration	GO_BP	5.35E-06	7.16E-06	6.49	5.00	[AGT, IGF1, NFE2L2, SRC, TLR4]
GO:0060439	trachea morphogenesis	GO_BP	5.46E-06	7.28E-06	27.27	3.00	[MAP2K1, MAPK1, MAPK3]
GO:0060020	Bergmann glial cell differentiation	GO_BP	5.46E-06	7.28E-06	27.27	3.00	[MAP2K1, MAPK1, MAPK3]
GO:0014745	negative regulation of muscle adaptation	GO_BP	5.46E-06	7.28E-06	27.27	3.00	[FOXO1, NOS3, PPARG]
GO:0038061	NIK/NF-kappaB signaling	GO_BP	5.55E-06	7.38E-06	4.41	6.00	[AKT1, APP, EGFR, IL1B, RELA, TLR4]
GO:0005977	glycogen metabolic process	GO_BP	5.71E-06	7.57E-06	6.41	5.00	[AKT1, GSK3B, IGF1, INS, IRS1]
GO:0016242	negative regulation of macroautophagy	GO_BP	5.75E-06	7.61E-06	11.11	4.00	[AKT1, HMOX1, MTOR, TP53]
GO:0010758	regulation of macrophage chemotaxis	GO_BP	5.75E-06	7.61E-06	11.11	4.00	[CYP19A1, MAPK1, MAPK3, PTK2]
GO:0046332	SMAD binding	GO_MF	6.08E-06	8.03E-06	6.33	5.00	[FOS, JUN, PARP1, PPARG, SMAD2]
GO:0044042	glucan metabolic process	GO_BP	6.08E-06	8.03E-06	6.33	5.00	[AKT1, GSK3B, IGF1, INS, IRS1]
GO:0006073	cellular glucan metabolic process	GO_BP	6.08E-06	8.03E-06	6.33	5.00	[AKT1, GSK3B, IGF1, INS, IRS1]
GO:0010565	regulation of cellular ketone	GO_BP	6.29E-	8.29E-06	4.32	6.00	[AKT1, IL1B, INS, IRS1, PPARG, PTGS2]

	metabolic process		06				
GO:1903580	positive regulation of ATP metabolic process	GO_BP	6.43E-06	8.46E-06	10.81	4.00	[APP, IGF1, INS, MTOR]
GO:0071312	cellular response to alkaloid	GO_BP	6.43E-06	8.46E-06	10.81	4.00	[BCL2L1, CASP3, CCNA2, CHEK1]
GO:0060674	placenta blood vessel development	GO_BP	6.43E-06	8.46E-06	10.81	4.00	[AKT1, CCNA2, MAP2K1, MAPK1]
GO:0051385	response to mineralocorticoid	GO_BP	6.43E-06	8.46E-06	10.81	4.00	[CCND1, FOS, PARP1, SRC]
GO:0061180	mammary gland epithelium development	GO_BP	6.47E-06	8.49E-06	6.25	5.00	[AKT1, CCND1, ESR1, MAPK1, SRC]
GO:0022612	gland morphogenesis	GO_BP	6.56E-06	8.58E-06	4.29	6.00	[EGFR, ESR1, IL6, MMP2, NFKB1, SRC]
GO:0072584	caveolin-mediated endocytosis	GO_BP	7.27E-06	9.49E-06	25.00	3.00	[MAPK1, MAPK3, SRC]
GO:0061517	macrophage proliferation	GO_BP	7.27E-06	9.49E-06	25.00	3.00	[MAPK1, MAPK3, PTK2]
GO:2000573	positive regulation of DNA biosynthetic process	GO_BP	7.30E-06	9.52E-06	6.10	5.00	[HSP90AA1, IL6, MAPK1, MAPK3, SRC]
GO:0042698	ovulation cycle	GO_BP	7.30E-06	9.52E-06	6.10	5.00	[CASP3, ESR1, MMP2, NOS3, SRC]
GO:0006801	superoxide metabolic process	GO_BP	7.30E-06	9.52E-06	6.10	5.00	[AGT, GSTP1, NFE2L2, NOS3, NQO1]
GO:0031570	DNA integrity checkpoint signaling	GO_BP	7.41E-06	9.63E-06	4.20	6.00	[CCND1, CDK1, CDK2, CHEK1, MAPK14, TP53]
GO:0045923	positive regulation of fatty acid metabolic process	GO_BP	7.97E-06	1.03E-05	10.26	4.00	[IL1B, IRS1, PPARG, PTGS2]
GO:0014911	positive regulation of smooth muscle cell migration	GO_BP	7.97E-06	1.03E-05	10.26	4.00	[AGT, IGF1, SRC, TLR4]
GO:0034103	regulation of tissue	GO_BP	8.22E-	1.06E-05	5.95	5.00	[AGT, IL6, PPARG, SRC, TP53]

	remodeling		06				
GO:0030168	platelet activation	GO_BP	8.68E-06	1.12E-05	4.08	6.00	[FN1, IL6, MAPK14, NOS3, SRC, TLR4]
KEGG:04723	Retrograde endocannabinoid signaling	KEGG	9.02E-06	1.16E-05	4.05	6.00	[MAPK1, MAPK14, MAPK3, MAPK8, MAPK9, PTGS2]
GO:0032368	regulation of lipid transport	GO_BP	9.02E-06	1.16E-05	4.05	6.00	[AGT, AKT1, CYP19A1, IL1B, NFKB1, PPARG]
GO:0071492	cellular response to UV-A	GO_BP	9.43E-06	1.21E-05	23.08	3.00	[MMP2, MMP3, MMP9]
GO:0071223	cellular response to lipoteichoic acid	GO_BP	9.43E-06	1.21E-05	23.08	3.00	[MAPK14, RELA, TLR4]
GO:0070391	response to lipoteichoic acid	GO_BP	9.43E-06	1.21E-05	23.08	3.00	[MAPK14, RELA, TLR4]
GO:0002674	negative regulation of acute inflammatory response	GO_BP	9.43E-06	1.21E-05	23.08	3.00	[GSTP1, INS, PPARG]
GO:0044773	mitotic DNA damage checkpoint signaling	GO_BP	9.76E-06	1.25E-05	5.75	5.00	[CCND1, CDK1, CDK2, CHEK1, TP53]
GO:0030331	nuclear estrogen receptor binding	GO_MF	9.76E-06	1.25E-05	9.76	4.00	[ESR1, PARP1, PPARG, SRC]
GO:0010742	macrophage derived foam cell differentiation	GO_BP	9.76E-06	1.25E-05	9.76	4.00	[AGT, MAPK9, NFKB1, PPARG]
GO:0043154	negative regulation of cysteine-type endopeptidase activity involved in apoptotic process	GO_BP	1.03E-05	1.32E-05	5.68	5.00	[AKT1, IL6, MMP9, PTGS2, SRC]
GO:0034637	cellular carbohydrate biosynthetic process	GO_BP	1.03E-05	1.32E-05	5.68	5.00	[AKT1, GSK3B, IGF1, INS, IRS1]
GO:0003300	cardiac muscle hypertrophy	GO_BP	1.03E-05	1.32E-05	5.68	5.00	[AGT, FOXO1, IGF1, PARP1, PPARG]

GO:0090077	foam cell differentiation	GO_BP	1.08E-05	1.37E-05	9.52	4.00	[AGT, MAPK9, NFKB1, PPARG]
GO:0031016	pancreas development	GO_BP	1.09E-05	1.39E-05	5.62	5.00	[AKT1, CDK6, GSK3B, IL6, SMAD2]
GO:0010833	telomere maintenance via telomere lengthening	GO_BP	1.09E-05	1.39E-05	5.62	5.00	[HSP90AA1, MAPK1, MAPK3, PARP1, SRC]
GO:0002532	production of molecular mediator involved in inflammatory response	GO_BP	1.15E-05	1.46E-05	5.56	5.00	[IL6, INS, MAPK14, STAT3, TLR4]
GO:2000279	negative regulation of DNA biosynthetic process	GO_BP	1.18E-05	1.50E-05	9.30	4.00	[CHEK1, PPARG, SRC, TP53]
GO:0050999	regulation of nitric-oxide synthase activity	GO_BP	1.18E-05	1.50E-05	9.30	4.00	[AKT1, EGFR, IL1B, INS]
GO:0010659	cardiac muscle cell apoptotic process	GO_BP	1.18E-05	1.50E-05	9.30	4.00	[AGT, HSP90AA1, NFE2L2, TP53]
GO:0043471	regulation of cellular carbohydrate catabolic process	GO_BP	1.20E-05	1.51E-05	21.43	3.00	[INS, MTOR, TP53]
GO:0014897	striated muscle hypertrophy	GO_BP	1.22E-05	1.53E-05	5.49	5.00	[AGT, FOXO1, IGF1, PARP1, PPARG]
GO:0044774	mitotic DNA integrity checkpoint signaling	GO_BP	1.28E-05	1.62E-05	5.43	5.00	[CCND1, CDK1, CDK2, CHEK1, TP53]
GO:0008625	extrinsic apoptotic signaling pathway via death domain receptors	GO_BP	1.28E-05	1.62E-05	5.43	5.00	[BCL2L1, GSK3B, HMOX1, IL6, NOS3]
GO:0007492	endoderm development	GO_BP	1.28E-05	1.62E-05	5.43	5.00	[FN1, MAP2K1, MMP2, MMP9, SMAD2]
GO:0046688	response to copper ion	GO_BP	1.30E-05	1.63E-05	9.09	4.00	[APP, CDK1, CYP1A1, NFE2L2]
GO:0045840	positive regulation of mitotic nuclear division	GO_BP	1.30E-05	1.63E-05	9.09	4.00	[AURKA, IGF1, IL1B, INS]

GO:0060395	SMAD protein signal transduction	GO_BP	1.35E-05	1.70E-05	5.38	5.00	[FOS, JUN, PARP1, PPARG, SMAD2]
GO:0014896	muscle hypertrophy	GO_BP	1.35E-05	1.70E-05	5.38	5.00	[AGT, FOXO1, IGF1, PARP1, PPARG]
GO:2001239	regulation of extrinsic apoptotic signaling pathway in absence of ligand	GO_BP	1.42E-05	1.78E-05	8.89	4.00	[AKT1, BCL2L1, IL1B, MCL1]
GO:1900087	positive regulation of G1/S transition of mitotic cell cycle	GO_BP	1.42E-05	1.78E-05	8.89	4.00	[AKT1, CCND1, CYP1A1, EGFR]
GO:0045022	early endosome to late endosome transport	GO_BP	1.42E-05	1.78E-05	8.89	4.00	[MAP2K1, MAPK1, MAPK3, SRC]
GO:0032677	regulation of interleukin-8 production	GO_BP	1.43E-05	1.78E-05	5.32	5.00	[IL1B, IL6, RELA, STAT3, TLR4]
GO:0032637	interleukin-8 production	GO_BP	1.43E-05	1.78E-05	5.32	5.00	[IL1B, IL6, RELA, STAT3, TLR4]
GO:0006970	response to osmotic stress	GO_BP	1.43E-05	1.78E-05	5.32	5.00	[CASP3, HSP90AA1, MTOR, PTGS2, TP53]
GO:0051974	negative regulation of telomerase activity	GO_BP	1.49E-05	1.86E-05	20.00	3.00	[PPARG, SRC, TP53]
GO:0014812	muscle cell migration	GO_BP	1.50E-05	1.86E-05	5.26	5.00	[AGT, IGF1, NFE2L2, SRC, TLR4]
GO:0046622	positive regulation of organ growth	GO_BP	1.55E-05	1.93E-05	8.70	4.00	[AKT1, CDK1, IGF1, MAPK14]
GO:0010658	striated muscle cell apoptotic process	GO_BP	1.55E-05	1.93E-05	8.70	4.00	[AGT, HSP90AA1, NFE2L2, TP53]
GO:0006096	glycolytic process	GO_BP	1.58E-05	1.95E-05	5.21	5.00	[APP, IGF1, INS, MTOR, STAT3]
GO:0006757	ATP generation from ADP	GO_BP	1.66E-05	2.05E-05	5.15	5.00	[APP, IGF1, INS, MTOR, STAT3]
GO:0051219	phosphoprotein binding	GO_MF	1.83E-	2.26E-05	5.05	5.00	[IRS1, MAPK1, MAPK3, MTOR, SRC]

			05				
GO:0032091	negative regulation of protein binding	GO_BP	1.83E-05	2.26E-05	5.05	5.00	[AKT1, AURKA, GSK3B, MAPK3, MAPK8]
GO:0001892	embryonic placenta development	GO_BP	1.83E-05	2.26E-05	5.05	5.00	[AKT1, CCNA2, EGFR, MAP2K1, MAPK1]
GO:1905953	negative regulation of lipid localization	GO_BP	1.84E-05	2.27E-05	8.33	4.00	[AKT1, IL6, NFKB1, PPARG]
GO:0098927	vesicle-mediated transport between endosomal compartments	GO_BP	1.84E-05	2.27E-05	8.33	4.00	[MAP2K1, MAPK1, MAPK3, SRC]
GO:0048156	tau protein binding	GO_MF	1.84E-05	2.27E-05	8.33	4.00	[CDK5, GSK3B, HSP90AA1, SMAD2]
KEGG:05030	Cocaine addiction	KEGG	2.00E-05	2.46E-05	8.16	4.00	[CDK5, JUN, NFKB1, RELA]
GO:0048246	macrophage chemotaxis	GO_BP	2.00E-05	2.46E-05	8.16	4.00	[CYP19A1, MAPK1, MAPK3, PTK2]
GO:0050886	endocrine process	GO_BP	2.02E-05	2.47E-05	4.95	5.00	[AGT, CYP19A1, IL1B, NOS3, PPARG]
GO:0090594	inflammatory response to wounding	GO_BP	2.22E-05	2.71E-05	17.65	3.00	[HMOX1, PPARG, TLR4]
GO:0090336	positive regulation of brown fat cell differentiation	GO_BP	2.22E-05	2.71E-05	17.65	3.00	[INS, MAPK14, PTGS2]
GO:0031053	primary miRNA processing	GO_BP	2.22E-05	2.71E-05	17.65	3.00	[IL6, SMAD2, STAT3]
GO:0005159	insulin-like growth factor receptor binding	GO_MF	2.22E-05	2.71E-05	17.65	3.00	[IGF1, INS, IRS1]
GO:2000117	negative regulation of cysteine-type endopeptidase activity	GO_BP	2.33E-05	2.84E-05	4.81	5.00	[AKT1, IL6, MMP9, PTGS2, SRC]
GO:0032755	positive regulation of interleukin-6 production	GO_BP	2.33E-05	2.84E-05	4.81	5.00	[APP, IL1B, IL6, STAT3, TLR4]

GO:1905521	regulation of macrophage migration	GO_BP	2.35E-05	2.86E-05	7.84	4.00	[CYP19A1, MAPK1, MAPK3, PTK2]
GO:0046031	ADP metabolic process	GO_BP	2.44E-05	2.96E-05	4.76	5.00	[APP, IGF1, INS, MTOR, STAT3]
GO:0060711	labyrinthine layer development	GO_BP	2.54E-05	3.08E-05	7.69	4.00	[AKT1, CCNA2, MAP2K1, MAPK1]
GO:0010718	positive regulation of epithelial to mesenchymal transition	GO_BP	2.54E-05	3.08E-05	7.69	4.00	[IL1B, IL6, MTOR, SMAD2]
GO:0006775	fat-soluble vitamin metabolic process	GO_BP	2.54E-05	3.08E-05	7.69	4.00	[CYP1A1, IL1B, NFKB1, NQO1]
KEGG:04928	Parathyroid hormone synthesis, secretion and action	KEGG	2.55E-05	3.09E-05	4.72	5.00	[EGFR, FOS, MAP2K1, MAPK1, MAPK3]
GO:1904035	regulation of epithelial cell apoptotic process	GO_BP	2.55E-05	3.09E-05	4.72	5.00	[ESR1, HMOX1, IL6, KDR, NFE2L2]
GO:1901222	regulation of NIK/NF-kappaB signaling	GO_BP	2.55E-05	3.09E-05	4.72	5.00	[APP, EGFR, IL1B, RELA, TLR4]
GO:0044264	cellular polysaccharide metabolic process	GO_BP	2.55E-05	3.09E-05	4.72	5.00	[AKT1, GSK3B, IGF1, INS, IRS1]
GO:2000811	negative regulation of anoikis	GO_BP	2.66E-05	3.21E-05	16.67	3.00	[MCL1, PTK2, SRC]
GO:0070102	interleukin-6-mediated signaling pathway	GO_BP	2.66E-05	3.21E-05	16.67	3.00	[IL6, SRC, STAT3]
GO:0051900	regulation of mitochondrial depolarization	GO_BP	2.66E-05	3.21E-05	16.67	3.00	[KDR, PARP1, SRC]
GO:0038128	ERBB2 signaling pathway	GO_BP	2.66E-05	3.21E-05	16.67	3.00	[EGFR, ERBB2, SRC]
GO:0060324	face development	GO_BP	2.74E-05	3.30E-05	7.55	4.00	[MAP2K1, MAPK1, MAPK3, MMP2]

GO:0035987	endodermal cell differentiation	GO_BP	2.74E-05	3.30E-05	7.55	4.00	[FN1, MAP2K1, MMP2, MMP9]
GO:0002066	columnar/cuboidal epithelial cell development	GO_BP	2.74E-05	3.30E-05	7.55	4.00	[AKT1, CDK6, GSK3B, SRC]
GO:1905477	positive regulation of protein localization to membrane	GO_BP	2.79E-05	3.36E-05	4.63	5.00	[AKT1, CDK5, EGFR, ERBB2, MAPK8]
GO:0014013	regulation of gliogenesis	GO_BP	2.79E-05	3.36E-05	4.63	5.00	[IL1B, IL6, MTOR, PPARG, TP53]
GO:1903076	regulation of protein localization to plasma membrane	GO_BP	2.92E-05	3.50E-05	4.59	5.00	[AKT1, BCL2L1, CDK5, EGFR, INS]
GO:0030316	osteoclast differentiation	GO_BP	2.92E-05	3.50E-05	4.59	5.00	[FOS, MAPK14, MTOR, SRC, TLR4]
GO:0048013	ephrin receptor signaling pathway	GO_BP	2.95E-05	3.53E-05	7.41	4.00	[MMP2, MMP9, PTK2, SRC]
GO:0034198	cellular response to amino acid starvation	GO_BP	2.95E-05	3.53E-05	7.41	4.00	[MAPK1, MAPK3, MAPK8, MTOR]
GO:0031018	endocrine pancreas development	GO_BP	2.95E-05	3.53E-05	7.41	4.00	[AKT1, CDK6, GSK3B, IL6]
GO:1903358	regulation of Golgi organization	GO_BP	3.15E-05	3.76E-05	15.79	3.00	[MAP2K1, MAPK1, MAPK3]
GO:0060438	trachea development	GO_BP	3.15E-05	3.76E-05	15.79	3.00	[MAP2K1, MAPK1, MAPK3]
GO:0002295	T-helper cell lineage commitment	GO_BP	3.15E-05	3.76E-05	15.79	3.00	[IL6, MTOR, STAT3]
GO:0140353	lipid export from cell	GO_BP	3.18E-05	3.79E-05	7.27	4.00	[AGT, CYP19A1, IL1B, PTGS2]
GO:1901655	cellular response to ketone	GO_BP	3.19E-05	3.79E-05	4.50	5.00	[AKT1, CASP9, CDK4, PPARG, SRC]
GO:0001938	positive regulation of endothelial cell proliferation	GO_BP	3.19E-05	3.79E-05	4.50	5.00	[AKT1, HMOX1, JUN, KDR, STAT3]

KEGG:04923	Regulation of lipolysis in adipocytes	KEGG	3.41E-05	4.05E-05	7.14	4.00	[AKT1, INS, IRS1, PTGS2]
GO:1990928	response to amino acid starvation	GO_BP	3.41E-05	4.05E-05	7.14	4.00	[MAPK1, MAPK3, MAPK8, MTOR]
GO:1903202	negative regulation of oxidative stress-induced cell death	GO_BP	3.41E-05	4.05E-05	7.14	4.00	[AKT1, IL6, INS, NFE2L2]
GO:0061900	glial cell activation	GO_BP	3.41E-05	4.05E-05	7.14	4.00	[APP, IL1B, IL6, JUN]
KEGG:04725	Cholinergic synapse	KEGG	3.47E-05	4.12E-05	4.42	5.00	[AKT1, FOS, MAP2K1, MAPK1, MAPK3]
GO:0008630	intrinsic apoptotic signaling pathway in response to DNA damage	GO_BP	3.47E-05	4.12E-05	4.42	5.00	[BCL2L1, CASP9, HMOX1, MCL1, TP53]
GO:0002042	cell migration involved in sprouting angiogenesis	GO_BP	3.66E-05	4.33E-05	7.02	4.00	[AKT1, HMOX1, KDR, PTGS2]
GO:0051882	mitochondrial depolarization	GO_BP	3.70E-05	4.36E-05	15.00	3.00	[KDR, PARP1, SRC]
GO:0010759	positive regulation of macrophage chemotaxis	GO_BP	3.70E-05	4.36E-05	15.00	3.00	[MAPK1, MAPK3, PTK2]
GO:0010744	positive regulation of macrophage derived foam cell differentiation	GO_BP	3.70E-05	4.36E-05	15.00	3.00	[AGT, MAPK9, NFKB1]
GO:0098869	cellular oxidant detoxification	GO_BP	3.78E-05	4.45E-05	4.35	5.00	[GSTP1, NFE2L2, NOS3, NQO1, PTGS2]
GO:0006165	nucleoside diphosphate phosphorylation	GO_BP	3.78E-05	4.45E-05	4.35	5.00	[APP, IGF1, INS, MTOR, STAT3]
GO:0045912	negative regulation of carbohydrate metabolic process	GO_BP	3.92E-05	4.61E-05	6.90	4.00	[GSK3B, INS, STAT3, TP53]
GO:0009409	response to cold	GO_BP	3.92E-05	4.61E-05	6.90	4.00	[FOS, FOXO1, HSP90AA1, PPARG]

GO:0046939	nucleotide phosphorylation	GO_BP	4.11E-05	4.81E-05	4.27	5.00	[APP, IGF1, INS, MTOR, STAT3]
GO:1902808	positive regulation of cell cycle G1/S phase transition	GO_BP	4.20E-05	4.91E-05	6.78	4.00	[AKT1, CCND1, CYP1A1, EGFR]
GO:0051785	positive regulation of nuclear division	GO_BP	4.20E-05	4.91E-05	6.78	4.00	[AURKA, IGF1, IL1B, INS]
GO:0007631	feeding behavior	GO_BP	4.28E-05	5.00E-05	4.24	5.00	[AGT, APP, FOS, INS, STAT3]
GO:0051769	regulation of nitric-oxide synthase biosynthetic process	GO_BP	4.30E-05	5.01E-05	14.29	3.00	[GSTP1, KDR, TLR4]
GO:0051767	nitric-oxide synthase biosynthetic process	GO_BP	4.30E-05	5.01E-05	14.29	3.00	[GSTP1, KDR, TLR4]
KEGG:04730	Long-term depression	KEGG	4.49E-05	5.22E-05	6.67	4.00	[IGF1, MAP2K1, MAPK1, MAPK3]
GO:0007566	embryo implantation	GO_BP	4.49E-05	5.22E-05	6.67	4.00	[IL1B, MMP2, MMP9, PTGS2]
GO:1901796	regulation of signal transduction by p53 class mediator	GO_BP	4.63E-05	5.38E-05	4.17	5.00	[AKT1, AURKA, CHEK1, MTOR, TP53]
GO:0051897	positive regulation of protein kinase B signaling	GO_BP	4.63E-05	5.38E-05	4.17	5.00	[EGFR, HSP90AA1, INS, PTK2, SRC]
GO:0009179	purine ribonucleoside diphosphate metabolic process	GO_BP	4.63E-05	5.38E-05	4.17	5.00	[APP, IGF1, INS, MTOR, STAT3]
GO:0009135	purine nucleoside diphosphate metabolic process	GO_BP	4.63E-05	5.38E-05	4.17	5.00	[APP, IGF1, INS, MTOR, STAT3]
GO:0060986	endocrine hormone secretion	GO_BP	4.79E-05	5.55E-05	6.56	4.00	[AGT, CYP19A1, IL1B, PPARG]

GO:0043388	positive regulation of DNA binding	GO_BP	4.79E-05	5.55E-05	6.56	4.00	[IGF1, MMP9, PARP1, PPARG]
GO:0038093	Fc receptor signaling pathway	GO_BP	4.79E-05	5.55E-05	6.56	4.00	[MAPK8, MAPK9, PTK2, SRC]
GO:0031281	positive regulation of cyclase activity	GO_BP	4.79E-05	5.55E-05	6.56	4.00	[MAPK14, MAPK3, MAPK8, NOS3]
GO:0030520	intracellular estrogen receptor signaling pathway	GO_BP	4.79E-05	5.55E-05	6.56	4.00	[ESR1, ESR2, PARP1, SRC]
GO:1903209	positive regulation of oxidative stress-induced cell death	GO_BP	4.97E-05	5.75E-05	13.64	3.00	[MCL1, MMP3, TLR4]
GO:1902176	negative regulation of oxidative stress-induced intrinsic apoptotic signaling pathway	GO_BP	4.97E-05	5.75E-05	13.64	3.00	[AKT1, INS, NFE2L2]
GO:0043373	CD4-positive, alpha-beta T cell lineage commitment	GO_BP	4.97E-05	5.75E-05	13.64	3.00	[IL6, MTOR, STAT3]
GO:0051224	negative regulation of protein transport	GO_BP	5.02E-05	5.79E-05	4.10	5.00	[CDK5, FOXO1, IL1B, INS, IRS1]
GO:0001221	transcription coregulator binding	GO_MF	5.02E-05	5.79E-05	4.10	5.00	[ESR1, FOS, NFE2L2, PPARG, RELA]
GO:1900015	regulation of cytokine production involved in inflammatory response	GO_BP	5.11E-05	5.88E-05	6.45	4.00	[IL6, MAPK14, STAT3, TLR4]
GO:0002534	cytokine production involved in inflammatory response	GO_BP	5.11E-05	5.88E-05	6.45	4.00	[IL6, MAPK14, STAT3, TLR4]
GO:0090100	positive regulation of transmembrane receptor protein serine/threonine kinase signaling pathway	GO_BP	5.22E-05	6.00E-05	4.07	5.00	[CCNA2, KDR, PARP1, PPARG, SMAD2]
GO:0007006	mitochondrial membrane	GO_BP	5.22E-	6.00E-05	4.07	5.00	[BCL2L1, GSK3B, HSP90AA1, MAPK8, TP53]

	organization		05				
KEGG:04623	Cytosolic DNA-sensing pathway	KEGG	5.44E-05	6.24E-05	6.35	4.00	[IL1B, IL6, NFKB1, RELA]
GO:0050709	negative regulation of protein secretion	GO_BP	5.44E-05	6.24E-05	6.35	4.00	[FOXO1, IL1B, INS, IRS1]
GO:0046902	regulation of mitochondrial membrane permeability	GO_BP	5.44E-05	6.24E-05	6.35	4.00	[BCL2L1, GSK3B, MAPK8, TP53]
GO:0043407	negative regulation of MAP kinase activity	GO_BP	5.44E-05	6.24E-05	6.35	4.00	[AGT, GSTP1, IL1B, PPARG]
GO:0090050	positive regulation of cell migration involved in sprouting angiogenesis	GO_BP	5.70E-05	6.53E-05	13.04	3.00	[HMOX1, KDR, PTGS2]
GO:0048143	astrocyte activation	GO_BP	5.70E-05	6.53E-05	13.04	3.00	[APP, IL1B, IL6]
GO:0010518	positive regulation of phospholipase activity	GO_BP	5.79E-05	6.61E-05	6.25	4.00	[AGT, CCNA2, EGFR, ESR1]
GO:0032655	regulation of interleukin-12 production	GO_BP	6.15E-05	7.02E-05	6.15	4.00	[MAPK14, NFKB1, RELA, TLR4]
GO:0032615	interleukin-12 production	GO_BP	6.15E-05	7.02E-05	6.15	4.00	[MAPK14, NFKB1, RELA, TLR4]
GO:1900017	positive regulation of cytokine production involved in inflammatory response	GO_BP	6.50E-05	7.40E-05	12.50	3.00	[IL6, STAT3, TLR4]
GO:0090335	regulation of brown fat cell differentiation	GO_BP	6.50E-05	7.40E-05	12.50	3.00	[INS, MAPK14, PTGS2]
GO:0060065	uterus development	GO_BP	6.50E-05	7.40E-05	12.50	3.00	[CYP19A1, ESR1, SRC]
GO:0044321	response to leptin	GO_BP	6.50E-05	7.40E-05	12.50	3.00	[CCNA2, CCND1, STAT3]
GO:0042359	vitamin D metabolic process	GO_BP	6.50E-	7.40E-05	12.50	3.00	[CYP1A1, IL1B, NFKB1]

			05				
GO:0002363	alpha-beta T cell lineage commitment	GO_BP	6.50E-05	7.40E-05	12.50	3.00	[IL6, MTOR, STAT3]
GO:0031102	neuron projection regeneration	GO_BP	6.53E-05	7.42E-05	6.06	4.00	[IL6, JUN, MAP2K1, MMP2]
GO:1904356	regulation of telomere maintenance via telomere lengthening	GO_BP	6.93E-05	7.86E-05	5.97	4.00	[MAPK1, MAPK3, PARP1, SRC]
GO:0051353	positive regulation of oxidoreductase activity	GO_BP	6.93E-05	7.86E-05	5.97	4.00	[AGT, AKT1, IL1B, INS]
GO:0032731	positive regulation of interleukin-1 beta production	GO_BP	7.35E-05	8.31E-05	5.88	4.00	[APP, IL6, STAT3, TLR4]
GO:0060716	labyrinthine layer blood vessel development	GO_BP	7.37E-05	8.32E-05	12.00	3.00	[AKT1, CCNA2, MAPK1]
GO:0044346	fibroblast apoptotic process	GO_BP	7.37E-05	8.32E-05	12.00	3.00	[CASP3, CASP9, TP53]
GO:0043369	CD4-positive or CD8-positive, alpha-beta T cell lineage commitment	GO_BP	7.37E-05	8.32E-05	12.00	3.00	[IL6, MTOR, STAT3]
GO:0030728	ovulation	GO_BP	7.37E-05	8.32E-05	12.00	3.00	[MMP2, NOS3, PTGS2]
GO:1905517	macrophage migration	GO_BP	7.78E-05	8.77E-05	5.80	4.00	[CYP19A1, MAPK1, MAPK3, PTK2]
GO:0035924	cellular response to vascular endothelial growth factor stimulus	GO_BP	8.23E-05	9.26E-05	5.71	4.00	[AKT1, KDR, MAPK14, RELA]
GO:0022617	extracellular matrix disassembly	GO_BP	8.23E-05	9.26E-05	5.71	4.00	[IL6, MMP2, MMP3, MMP9]
GO:0014015	positive regulation of gliogenesis	GO_BP	8.23E-05	9.26E-05	5.71	4.00	[IL1B, IL6, MTOR, PPARG]
GO:0036120	cellular response to platelet-derived growth factor	GO_BP	8.31E-	9.33E-05	11.54	3.00	[CCNA2, SRC, TLR4]

	stimulus		05				
GO:0035902	response to immobilization stress	GO_BP	8.31E-05	9.33E-05	11.54	3.00	[CYP1A1, FOS, PPARG]
GO:0019430	removal of superoxide radicals	GO_BP	8.31E-05	9.33E-05	11.54	3.00	[NFE2L2, NOS3, NQO1]
GO:0010226	response to lithium ion	GO_BP	8.31E-05	9.33E-05	11.54	3.00	[CDH1, NFE2L2, PTGS2]
GO:0040014	regulation of multicellular organism growth	GO_BP	9.19E-05	1.03E-04	5.56	4.00	[APP, CDK4, IGF1, STAT3]
GO:2000209	regulation of anoikis	GO_BP	9.33E-05	1.04E-04	11.11	3.00	[MCL1, PTK2, SRC]
GO:1905523	positive regulation of macrophage migration	GO_BP	9.33E-05	1.04E-04	11.11	3.00	[MAPK1, MAPK3, PTK2]
GO:0034104	negative regulation of tissue remodeling	GO_BP	9.33E-05	1.04E-04	11.11	3.00	[AGT, IL6, PPARG]
GO:0010661	positive regulation of muscle cell apoptotic process	GO_BP	9.33E-05	1.04E-04	11.11	3.00	[AGT, PPARG, TP53]
GO:0003323	type B pancreatic cell development	GO_BP	9.33E-05	1.04E-04	11.11	3.00	[AKT1, CDK6, GSK3B]
GO:0097110	scaffold protein binding	GO_MF	1.02E-04	1.14E-04	5.41	4.00	[HSP90AA1, MAP2K1, NOS3, SRC]
GO:0097199	cysteine-type endopeptidase activity involved in apoptotic signaling pathway	GO_BP	1.04E-04	1.16E-04	10.71	3.00	[CASP3, CASP9, MMP9]
GO:0071451	cellular response to superoxide	GO_BP	1.04E-04	1.16E-04	10.71	3.00	[NFE2L2, NOS3, NQO1]
GO:0071450	cellular response to oxygen radical	GO_BP	1.04E-04	1.16E-04	10.71	3.00	[NFE2L2, NOS3, NQO1]
GO:0036119	response to platelet-derived growth factor	GO_BP	1.04E-04	1.16E-04	10.71	3.00	[CCNA2, SRC, TLR4]

GO:0010613	positive regulation of cardiac muscle hypertrophy	GO_BP	1.04E-04	1.16E-04	10.71	3.00	[AGT, IGF1, PARP1]
GO:0071230	cellular response to amino acid stimulus	GO_BP	1.08E-04	1.20E-04	5.33	4.00	[BCL2L1, EGFR, MMP2, MTOR]
GO:0060193	positive regulation of lipase activity	GO_BP	1.13E-04	1.26E-04	5.26	4.00	[AGT, CCNA2, EGFR, ESR1]
GO:0071280	cellular response to copper ion	GO_BP	1.16E-04	1.29E-04	10.34	3.00	[APP, CYP1A1, NFE2L2]
GO:0055023	positive regulation of cardiac muscle tissue growth	GO_BP	1.16E-04	1.29E-04	10.34	3.00	[CDK1, IGF1, MAPK14]
GO:0048011	neurotrophin TRK receptor signaling pathway	GO_BP	1.16E-04	1.29E-04	10.34	3.00	[AGT, CASP3, SRC]
GO:0014742	positive regulation of muscle hypertrophy	GO_BP	1.16E-04	1.29E-04	10.34	3.00	[AGT, IGF1, PARP1]
GO:0010575	positive regulation of vascular endothelial growth factor production	GO_BP	1.16E-04	1.29E-04	10.34	3.00	[IL1B, IL6, PTGS2]
KEGG:05100	Bacterial invasion of epithelial cells	KEGG	1.19E-04	1.32E-04	5.19	4.00	[CDH1, FN1, PTK2, SRC]
GO:2000142	regulation of DNA-templated transcription, initiation	GO_BP	1.26E-04	1.39E-04	5.13	4.00	[CDK4, ESR1, JUN, TP53]
GO:0010517	regulation of phospholipase activity	GO_BP	1.26E-04	1.39E-04	5.13	4.00	[AGT, CCNA2, EGFR, ESR1]
GO:0007004	telomere maintenance via telomerase	GO_BP	1.26E-04	1.39E-04	5.13	4.00	[HSP90AA1, MAPK1, MAPK3, SRC]
GO:2000737	negative regulation of stem cell differentiation	GO_BP	1.29E-04	1.42E-04	10.00	3.00	[GSK3B, NFE2L2, STAT3]
GO:0050995	negative regulation of lipid catabolic process	GO_BP	1.29E-04	1.42E-04	10.00	3.00	[AKT1, IL1B, INS]
GO:0033598	mammary gland epithelial	GO_BP	1.29E-	1.42E-04	10.00	3.00	[CCND1, ESR1, MAPK1]

	cell proliferation		04				
GO:0031571	mitotic G1 DNA damage checkpoint signaling	GO_BP	1.29E-04	1.42E-04	10.00	3.00	[CCND1, CDK2, TP53]
GO:0055017	cardiac muscle tissue growth	GO_BP	1.39E-04	1.52E-04	5.00	4.00	[AGT, CDK1, IGF1, MAPK14]
GO:0045685	regulation of glial cell differentiation	GO_BP	1.39E-04	1.52E-04	5.00	4.00	[CDK1, IL6, MTOR, PPARG]
GO:0090140	regulation of mitochondrial fission	GO_BP	1.42E-04	1.56E-04	9.68	3.00	[AURKA, KDR, PPARG]
GO:0055094	response to lipoprotein particle	GO_BP	1.42E-04	1.56E-04	9.68	3.00	[AKT1, PPARG, TLR4]
GO:0044819	mitotic G1/S transition checkpoint signaling	GO_BP	1.42E-04	1.56E-04	9.68	3.00	[CCND1, CDK2, TP53]
GO:0030878	thyroid gland development	GO_BP	1.42E-04	1.56E-04	9.68	3.00	[MAP2K1, MAPK1, MAPK3]
GO:0002675	positive regulation of acute inflammatory response	GO_BP	1.42E-04	1.56E-04	9.68	3.00	[IL1B, IL6, PTGS2]
GO:0000303	response to superoxide	GO_BP	1.42E-04	1.56E-04	9.68	3.00	[NFE2L2, NOS3, NQO1]
GO:0055013	cardiac muscle cell development	GO_BP	1.52E-04	1.67E-04	4.88	4.00	[AGT, CDK1, IGF1, MTOR]
GO:0032732	positive regulation of interleukin-1 production	GO_BP	1.52E-04	1.67E-04	4.88	4.00	[APP, IL6, STAT3, TLR4]
GO:0090322	regulation of superoxide metabolic process	GO_BP	1.56E-04	1.71E-04	9.38	3.00	[AGT, GSTP1, NFE2L2]
GO:0000305	response to oxygen radical	GO_BP	1.56E-04	1.71E-04	9.38	3.00	[NFE2L2, NOS3, NQO1]
GO:0071229	cellular response to acid chemical	GO_BP	1.67E-04	1.83E-04	4.76	4.00	[BCL2L1, EGFR, MMP2, MTOR]
GO:1903203	regulation of oxidative	GO_BP	1.72E-	1.87E-04	9.09	3.00	[MCL1, PARP1, TLR4]

	stress-induced neuron death		04				
GO:0060421	positive regulation of heart growth	GO_BP	1.72E-04	1.87E-04	9.09	3.00	[CDK1, IGF1, MAPK14]
GO:0010574	regulation of vascular endothelial growth factor production	GO_BP	1.72E-04	1.87E-04	9.09	3.00	[IL1B, IL6, PTGS2]
GO:0010165	response to X-ray	GO_BP	1.72E-04	1.87E-04	9.09	3.00	[CASP3, CCND1, TP53]
GO:0071402	cellular response to lipoprotein particle stimulus	GO_BP	1.88E-04	2.04E-04	8.82	3.00	[AKT1, PPARG, TLR4]
GO:0043552	positive regulation of phosphatidylinositol 3-kinase activity	GO_BP	1.88E-04	2.04E-04	8.82	3.00	[IRS1, PTK2, SRC]
GO:0010039	response to iron ion	GO_BP	1.88E-04	2.04E-04	8.82	3.00	[CCND1, CYP1A1, HMOX1]
GO:0055006	cardiac cell development	GO_BP	1.92E-04	2.08E-04	4.60	4.00	[AGT, CDK1, IGF1, MTOR]
GO:0019915	lipid storage	GO_BP	2.00E-04	2.17E-04	4.55	4.00	[IL1B, IL6, NFKB1, PPARG]
GO:0007422	peripheral nervous system development	GO_BP	2.00E-04	2.17E-04	4.55	4.00	[AKT1, CDK1, CDK5, ERBB2]
GO:0051973	positive regulation of telomerase activity	GO_BP	2.05E-04	2.22E-04	8.57	3.00	[HSP90AA1, MAPK1, MAPK3]
GO:0046825	regulation of protein export from nucleus	GO_BP	2.05E-04	2.22E-04	8.57	3.00	[CDK5, GSK3B, IL1B]
GO:0003309	type B pancreatic cell differentiation	GO_BP	2.05E-04	2.22E-04	8.57	3.00	[AKT1, CDK6, GSK3B]
GO:2000736	regulation of stem cell differentiation	GO_BP	2.09E-04	2.26E-04	4.49	4.00	[CDK6, GSK3B, NFE2L2, STAT3]
GO:0060419	heart growth	GO_BP	2.09E-04	2.26E-04	4.49	4.00	[AGT, CDK1, IGF1, MAPK14]

GO:0048147	negative regulation of fibroblast proliferation	GO_BP	2.23E-04	2.40E-04	8.33	3.00	[GSTP1, PPARG, TP53]
GO:0036475	neuron death in response to oxidative stress	GO_BP	2.23E-04	2.40E-04	8.33	3.00	[MCL1, PARP1, TLR4]
GO:0032770	positive regulation of monooxygenase activity	GO_BP	2.23E-04	2.40E-04	8.33	3.00	[AKT1, IL1B, INS]
GO:0031279	regulation of cyclase activity	GO_BP	2.28E-04	2.45E-04	4.40	4.00	[MAPK14, MAPK3, MAPK8, NOS3]
GO:0021766	hippocampus development	GO_BP	2.28E-04	2.45E-04	4.40	4.00	[CASP3, CDK5, CDK6, GSK3B]
GO:0033077	T cell differentiation in thymus	GO_BP	2.38E-04	2.55E-04	4.35	4.00	[CDK6, ERBB2, IL1B, TP53]
GO:0071398	cellular response to fatty acid	GO_BP	2.42E-04	2.59E-04	8.11	3.00	[CDK4, IRS1, SRC]
GO:0060251	regulation of glial cell proliferation	GO_BP	2.42E-04	2.59E-04	8.11	3.00	[IL1B, IL6, TP53]
GO:0030224	monocyte differentiation	GO_BP	2.42E-04	2.59E-04	8.11	3.00	[CDK6, JUN, PPARG]
GO:0010573	vascular endothelial growth factor production	GO_BP	2.42E-04	2.59E-04	8.11	3.00	[IL1B, IL6, PTGS2]
GO:1901983	regulation of protein acetylation	GO_BP	2.58E-04	2.76E-04	4.26	4.00	[CHEK1, GSK3B, IL1B, MAPK3]
GO:0032092	positive regulation of protein binding	GO_BP	2.58E-04	2.76E-04	4.26	4.00	[APP, CDK5, GSK3B, MMP9]
GO:0008306	associative learning	GO_BP	2.58E-04	2.76E-04	4.26	4.00	[AGT, APP, CDK5, FOS]
GO:0060218	hematopoietic stem cell differentiation	GO_BP	2.62E-04	2.80E-04	7.89	3.00	[CDK6, NFE2L2, TP53]
GO:0048678	response to axon injury	GO_BP	2.69E-04	2.86E-04	4.21	4.00	[CDK1, JUN, MAP2K1, MMP2]

GO:0060291	long-term synaptic potentiation	GO_BP	2.80E-04	2.98E-04	4.17	4.00	[APP, GSK3B, INS, MAPK1]
GO:0010717	regulation of epithelial to mesenchymal transition	GO_BP	2.80E-04	2.98E-04	4.17	4.00	[IL1B, IL6, MTOR, SMAD2]
GO:0090218	positive regulation of lipid kinase activity	GO_BP	2.83E-04	3.01E-04	7.69	3.00	[IRS1, PTK2, SRC]
GO:0060416	response to growth hormone	GO_BP	2.83E-04	3.01E-04	7.69	3.00	[AKT1, PTK2, STAT3]
GO:0048009	insulin-like growth factor receptor signaling pathway	GO_BP	2.83E-04	3.01E-04	7.69	3.00	[AKT1, IGF1, IRS1]
GO:0046320	regulation of fatty acid oxidation	GO_BP	2.83E-04	3.01E-04	7.69	3.00	[AKT1, IRS1, PPARG]
GO:0010665	regulation of cardiac muscle cell apoptotic process	GO_BP	2.83E-04	3.01E-04	7.69	3.00	[AGT, NFE2L2, TP53]
KEGG:04666	Fc gamma R-mediated phagocytosis	KEGG	2.91E-04	3.08E-04	4.12	4.00	[AKT1, MAP2K1, MAPK1, MAPK3]
GO:0046620	regulation of organ growth	GO_BP	2.91E-04	3.08E-04	4.12	4.00	[AKT1, CDK1, IGF1, MAPK14]
GO:0008593	regulation of Notch signaling pathway	GO_BP	2.91E-04	3.08E-04	4.12	4.00	[AKT1, NOS3, SRC, STAT3]
GO:1902745	positive regulation of lamellipodium organization	GO_BP	3.06E-04	3.23E-04	7.50	3.00	[HSP90AA1, MTOR, SRC]
GO:1901532	regulation of hematopoietic progenitor cell differentiation	GO_BP	3.06E-04	3.23E-04	7.50	3.00	[CDK6, KDR, NFE2L2]
GO:0090049	regulation of cell migration involved in sprouting angiogenesis	GO_BP	3.06E-04	3.23E-04	7.50	3.00	[HMOX1, KDR, PTGS2]
GO:0043368	positive T cell selection	GO_BP	3.06E-04	3.23E-04	7.50	3.00	[IL6, MTOR, STAT3]
GO:0002068	glandular epithelial cell	GO_BP	3.06E-	3.23E-04	7.50	3.00	[AKT1, CDK6, GSK3B]

	development		04				
GO:0106027	neuron projection organization	GO_BP	3.15E-04	3.32E-04	4.04	4.00	[APP, CDK5, GSK3B, INS]
GO:0042805	actinin binding	GO_MF	3.29E-04	3.47E-04	7.32	3.00	[NFKB1, PPARG, RELA]
GO:0010656	negative regulation of muscle cell apoptotic process	GO_BP	3.29E-04	3.47E-04	7.32	3.00	[HMOX1, IGF1, NFE2L2]
GO:1902003	regulation of amyloid-beta formation	GO_BP	3.53E-04	3.72E-04	7.14	3.00	[CASP3, IGF1, RELA]
GO:0070884	regulation of calcineurin-NFAT signaling cascade	GO_BP	3.53E-04	3.72E-04	7.14	3.00	[GSK3B, IGF1, MTOR]
GO:0035883	enteroendocrine cell differentiation	GO_BP	3.53E-04	3.72E-04	7.14	3.00	[AKT1, CDK6, GSK3B]
GO:0030513	positive regulation of BMP signaling pathway	GO_BP	3.53E-04	3.72E-04	7.14	3.00	[CCNA2, KDR, SMAD2]
GO:0014037	Schwann cell differentiation	GO_BP	3.53E-04	3.72E-04	7.14	3.00	[AKT1, CDK1, CDK5]
GO:0010662	regulation of striated muscle cell apoptotic process	GO_BP	3.53E-04	3.72E-04	7.14	3.00	[AGT, NFE2L2, TP53]
GO:0000266	mitochondrial fission	GO_BP	3.53E-04	3.72E-04	7.14	3.00	[AURKA, KDR, PPARG]
GO:0140467	integrated stress response signaling	GO_BP	3.79E-04	3.98E-04	6.98	3.00	[FOS, JUN, NFE2L2]
GO:0106056	regulation of calcineurin-mediated signaling	GO_BP	3.79E-04	3.98E-04	6.98	3.00	[GSK3B, IGF1, MTOR]
GO:0051155	positive regulation of striated muscle cell differentiation	GO_BP	3.79E-04	3.98E-04	6.98	3.00	[IGF1, MAPK14, MTOR]
GO:0045740	positive regulation of DNA replication	GO_BP	3.79E-04	3.98E-04	6.98	3.00	[CDK1, CDK2, EGFR]

GO:0032733	positive regulation of interleukin-10 production	GO_BP	3.79E-04	3.98E-04	6.98	3.00	[IL6, STAT3, TLR4]
GO:0016712	oxidoreductase activity, acting on paired donors, with incorporation or reduction of molecular oxygen, reduced flavin or flavoprotein as one donor, and incorporation of one atom of oxygen	GO_MF	3.79E-04	3.98E-04	6.98	3.00	[CYP19A1, CYP1A1, HMOX1]
GO:0038179	neurotrophin signaling pathway	GO_BP	4.06E-04	4.26E-04	6.82	3.00	[AGT, CASP3, SRC]
GO:0032735	positive regulation of interleukin-12 production	GO_BP	4.06E-04	4.26E-04	6.82	3.00	[MAPK14, RELA, TLR4]
GO:0003254	regulation of membrane depolarization	GO_BP	4.06E-04	4.26E-04	6.82	3.00	[KDR, PARP1, SRC]
GO:0071364	cellular response to epidermal growth factor stimulus	GO_BP	4.34E-04	4.54E-04	6.67	3.00	[AKT1, EGFR, ERBB2]
GO:0035794	positive regulation of mitochondrial membrane permeability	GO_BP	4.34E-04	4.54E-04	6.67	3.00	[GSK3B, MAPK8, TP53]
GO:0031952	regulation of protein autophosphorylation	GO_BP	4.34E-04	4.54E-04	6.67	3.00	[INS, JUN, SRC]
GO:0010863	positive regulation of phospholipase C activity	GO_BP	4.34E-04	4.54E-04	6.67	3.00	[AGT, EGFR, ESR1]
GO:1902108	regulation of mitochondrial membrane permeability involved in apoptotic process	GO_BP	4.63E-04	4.84E-04	6.52	3.00	[GSK3B, MAPK8, TP53]
GO:0045454	cell redox homeostasis	GO_BP	4.63E-04	4.84E-04	6.52	3.00	[NFE2L2, NOS3, NQO1]
GO:0042169	SH2 domain binding	GO_MF	4.63E-04	4.84E-04	6.52	3.00	[IRS1, PTK2, SRC]

GO:0051879	Hsp90 protein binding	GO_MF	4.93E-04	5.15E-04	6.38	3.00	[CDK5, CYP1A1, KDR]
GO:0033173	calcineurin-NFAT signaling cascade	GO_BP	4.93E-04	5.15E-04	6.38	3.00	[GSK3B, IGF1, MTOR]
GO:1902991	regulation of amyloid precursor protein catabolic process	GO_BP	5.25E-04	5.47E-04	6.25	3.00	[CASP3, IGF1, RELA]
GO:1900274	regulation of phospholipase C activity	GO_BP	5.25E-04	5.47E-04	6.25	3.00	[AGT, EGFR, ESR1]
GO:1900271	regulation of long-term synaptic potentiation	GO_BP	5.25E-04	5.47E-04	6.25	3.00	[APP, GSK3B, INS]
GO:0030574	collagen catabolic process	GO_BP	5.25E-04	5.47E-04	6.25	3.00	[MMP2, MMP3, MMP9]
GO:0001784	phosphotyrosine residue binding	GO_MF	5.25E-04	5.47E-04	6.25	3.00	[IRS1, MAPK1, MAPK3]
GO:0001774	microglial cell activation	GO_BP	5.25E-04	5.47E-04	6.25	3.00	[APP, IL6, JUN]
GO:1905710	positive regulation of membrane permeability	GO_BP	5.58E-04	5.80E-04	6.12	3.00	[GSK3B, MAPK8, TP53]
GO:0045747	positive regulation of Notch signaling pathway	GO_BP	5.58E-04	5.80E-04	6.12	3.00	[NOS3, SRC, STAT3]
GO:0045646	regulation of erythrocyte differentiation	GO_BP	5.58E-04	5.80E-04	6.12	3.00	[CDK6, MAPK14, STAT3]
GO:0042088	T-helper 1 type immune response	GO_BP	5.58E-04	5.80E-04	6.12	3.00	[IL1B, MTOR, TLR4]
GO:0006984	ER-nucleus signaling pathway	GO_BP	5.58E-04	5.80E-04	6.12	3.00	[GSK3B, NFE2L2, TP53]
KEGG:05144	Malaria	KEGG	5.92E-04	6.14E-04	6.00	3.00	[IL1B, IL6, TLR4]
GO:0051602	response to electrical	GO_BP	5.92E-	6.14E-04	6.00	3.00	[MMP2, NQO1, SRC]

	stimulus		04				
GO:0042220	response to cocaine	GO_BP	5.92E-04	6.14E-04	6.00	3.00	[CCNA2, CDK5, HSP90AA1]
GO:0034205	amyloid-beta formation	GO_BP	5.92E-04	6.14E-04	6.00	3.00	[CASP3, IGF1, RELA]
GO:0031641	regulation of myelination	GO_BP	6.28E-04	6.50E-04	5.88	3.00	[AKT1, IL6, MTOR]
GO:0002269	leukocyte activation involved in inflammatory response	GO_BP	6.28E-04	6.50E-04	5.88	3.00	[APP, IL6, JUN]
GO:0001046	core promoter sequence-specific DNA binding	GO_MF	6.28E-04	6.50E-04	5.88	3.00	[FOS, RELA, TP53]
GO:0097720	calcineurin-mediated signaling	GO_BP	6.65E-04	6.87E-04	5.77	3.00	[GSK3B, IGF1, MTOR]
GO:0071470	cellular response to osmotic stress	GO_BP	6.65E-04	6.87E-04	5.77	3.00	[CASP3, MTOR, PTGS2]
GO:0060425	lung morphogenesis	GO_BP	6.65E-04	6.87E-04	5.77	3.00	[MAP2K1, MAPK1, MAPK3]
GO:0048538	thymus development	GO_BP	6.65E-04	6.87E-04	5.77	3.00	[MAP2K1, MAPK1, MAPK3]
GO:0044275	cellular carbohydrate catabolic process	GO_BP	6.65E-04	6.87E-04	5.77	3.00	[INS, MTOR, TP53]
GO:0030225	macrophage differentiation	GO_BP	6.65E-04	6.87E-04	5.77	3.00	[APP, MMP9, PARP1]
GO:0010677	negative regulation of cellular carbohydrate metabolic process	GO_BP	6.65E-04	6.87E-04	5.77	3.00	[GSK3B, INS, STAT3]
GO:0009299	mRNA transcription	GO_BP	6.65E-04	6.87E-04	5.77	3.00	[PPARG, STAT3, TP53]
GO:2000378	negative regulation of reactive oxygen species	GO_BP	7.03E-	7.25E-04	5.66	3.00	[INS, MMP3, TP53]

	metabolic process		04				
GO:0045058	T cell selection	GO_BP	7.03E-04	7.25E-04	5.66	3.00	[IL6, MTOR, STAT3]
GO:0060443	mammary gland morphogenesis	GO_BP	7.43E-04	7.65E-04	5.56	3.00	[ESR1, NFKB1, SRC]
GO:0010883	regulation of lipid storage	GO_BP	7.43E-04	7.65E-04	5.56	3.00	[IL6, NFKB1, PPARG]
GO:0006693	prostaglandin metabolic process	GO_BP	7.43E-04	7.65E-04	5.56	3.00	[GSTP1, IL1B, PTGS2]
GO:0006692	prostanoid metabolic process	GO_BP	7.43E-04	7.65E-04	5.56	3.00	[GSTP1, IL1B, PTGS2]
GO:0050873	brown fat cell differentiation	GO_BP	8.70E-04	8.94E-04	5.26	3.00	[INS, MAPK14, PTGS2]
GO:0043331	response to dsRNA	GO_BP	8.70E-04	8.94E-04	5.26	3.00	[MAPK1, MAPK3, NFKB1]
GO:0042311	vasodilation	GO_BP	8.70E-04	8.94E-04	5.26	3.00	[AGT, INS, NOS3]
GO:0031103	axon regeneration	GO_BP	8.70E-04	8.94E-04	5.26	3.00	[JUN, MAP2K1, MMP2]
GO:0014009	glial cell proliferation	GO_BP	8.70E-04	8.94E-04	5.26	3.00	[IL1B, IL6, TP53]
GO:1902743	regulation of lamellipodium organization	GO_BP	9.15E-04	9.39E-04	5.17	3.00	[HSP90AA1, MTOR, SRC]
GO:0071622	regulation of granulocyte chemotaxis	GO_BP	9.15E-04	9.39E-04	5.17	3.00	[MAPK1, MAPK3, PTK2]
GO:0048662	negative regulation of smooth muscle cell proliferation	GO_BP	9.15E-04	9.39E-04	5.17	3.00	[HMOX1, NOS3, PPARG]
GO:0002931	response to ischemia	GO_BP	9.15E-04	9.39E-04	5.17	3.00	[CASP9, NQO1, TP53]

GO:0002686	negative regulation of leukocyte migration	GO_BP	9.15E-04	9.39E-04	5.17	3.00	[AKT1, CYP19A1, HMOX1]
GO:0055021	regulation of cardiac muscle tissue growth	GO_BP	9.62E-04	9.86E-04	5.08	3.00	[CDK1, IGF1, MAPK14]
GO:0043551	regulation of phosphatidylinositol 3-kinase activity	GO_BP	9.62E-04	9.86E-04	5.08	3.00	[IRS1, PTK2, SRC]

pAKT

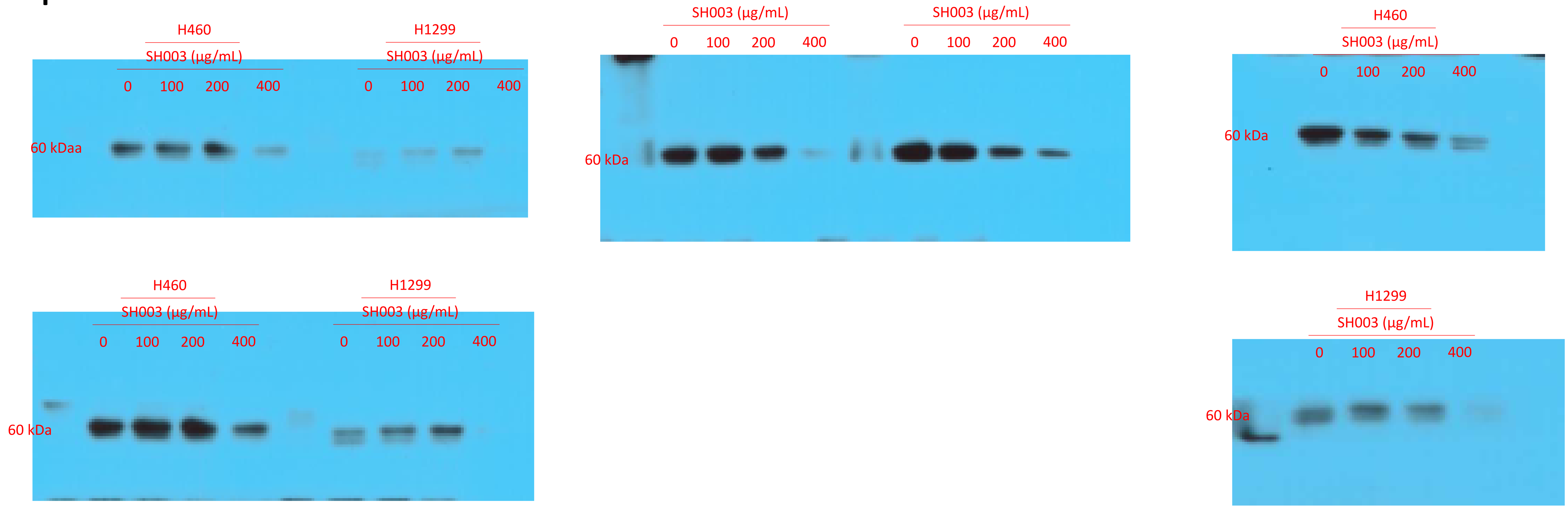

AKT

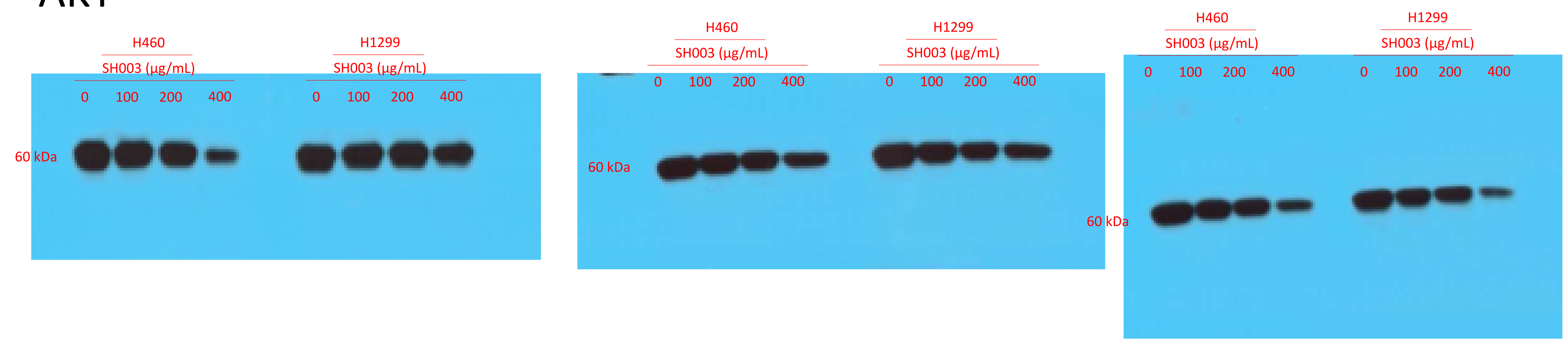

pERK

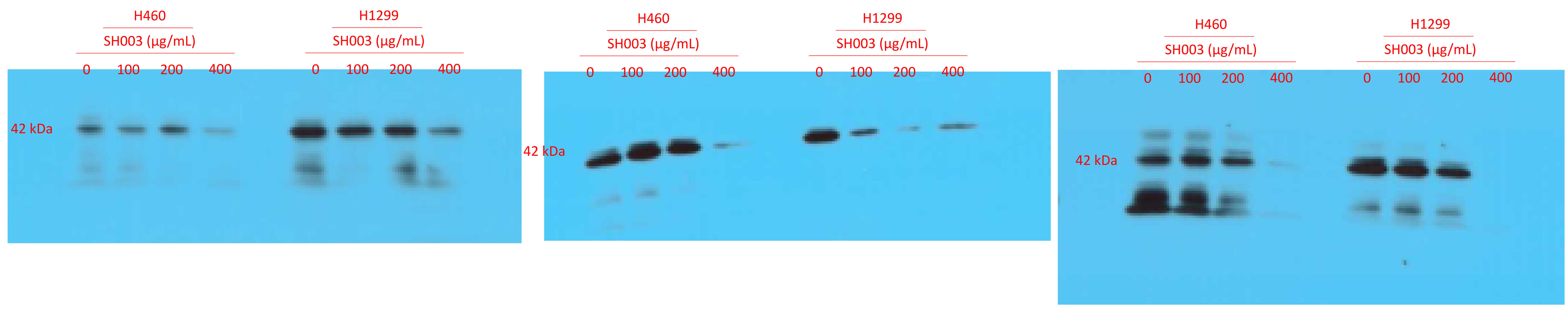

ERK

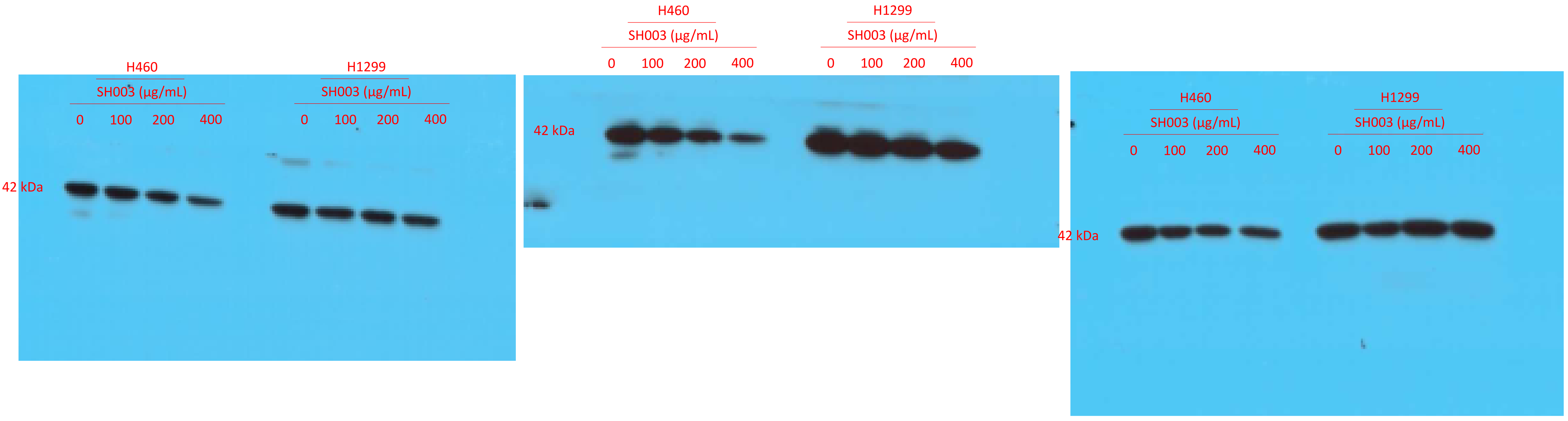

Due to pre-cutting the membranes after transfer, a 10~20 kDa margin was left around the band matching the specified protein size, making it impossible to display a full film image.

pSTAT3

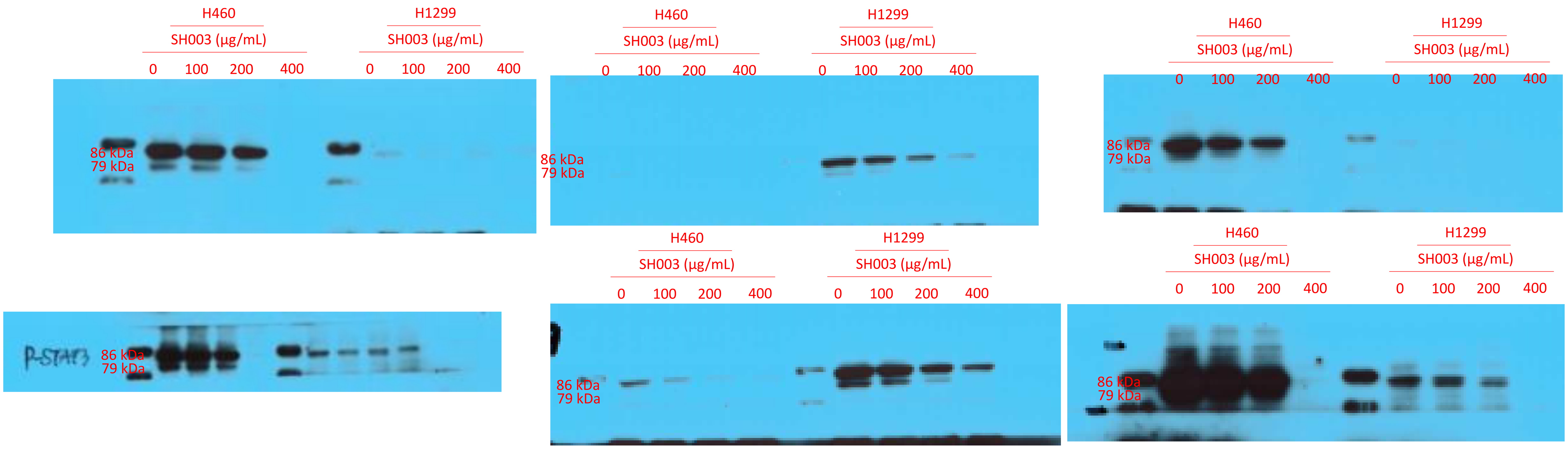

STAT3

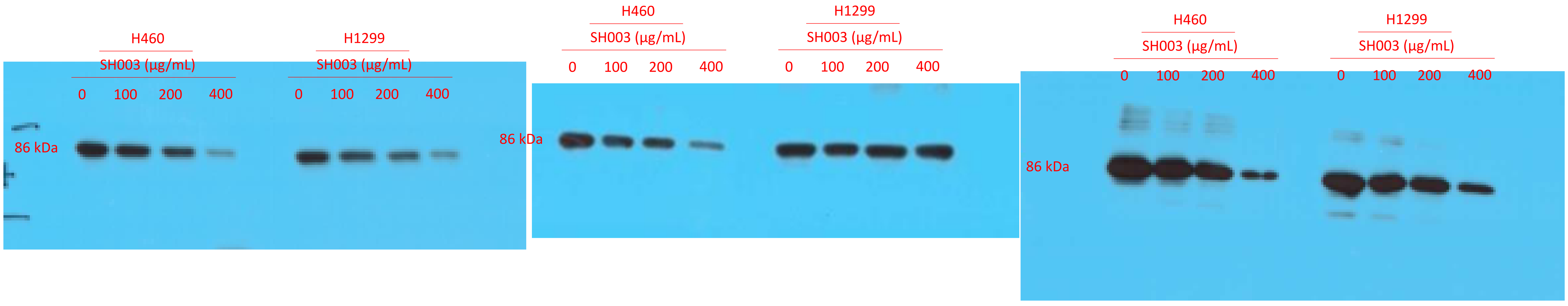

c-JUN

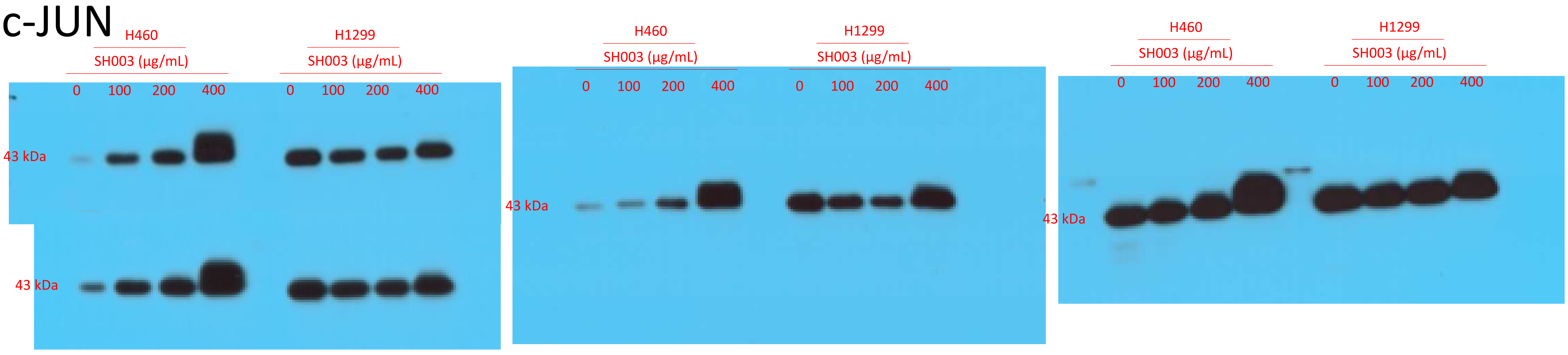

Due to pre-cutting the membranes after transfer, a 10~20 kDa margin was left around the band matching the specified protein size, making it impossible to display a full film image.

p53

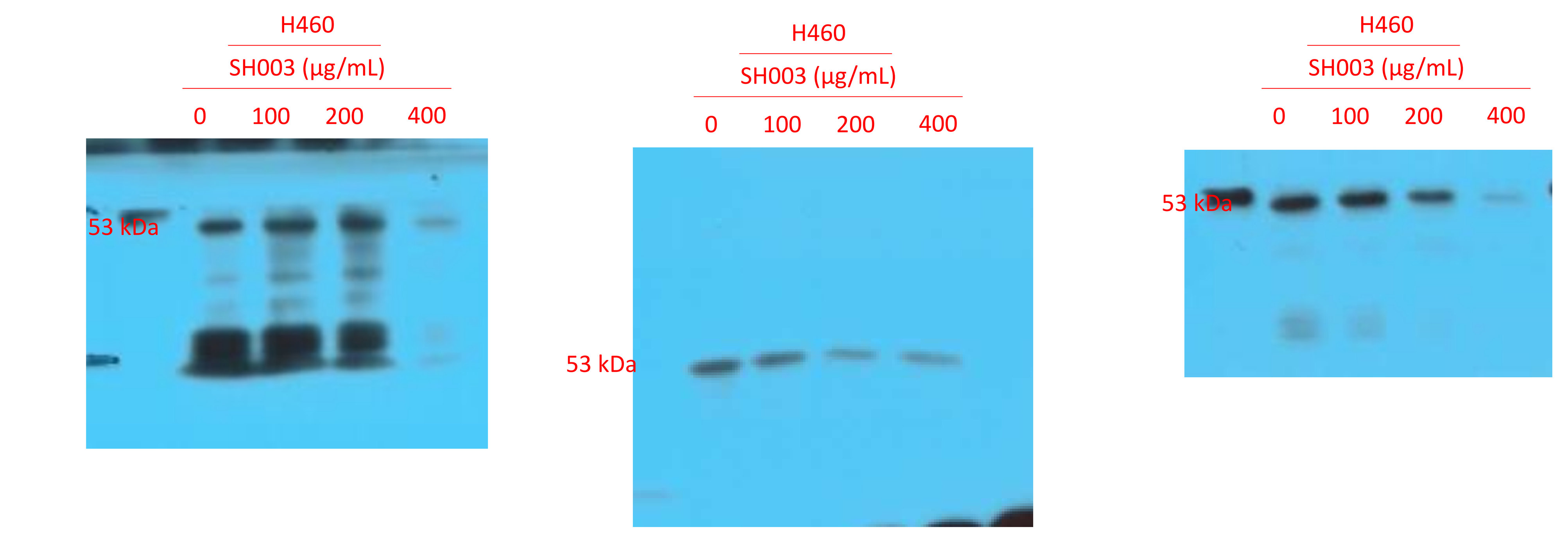

α-tubulin

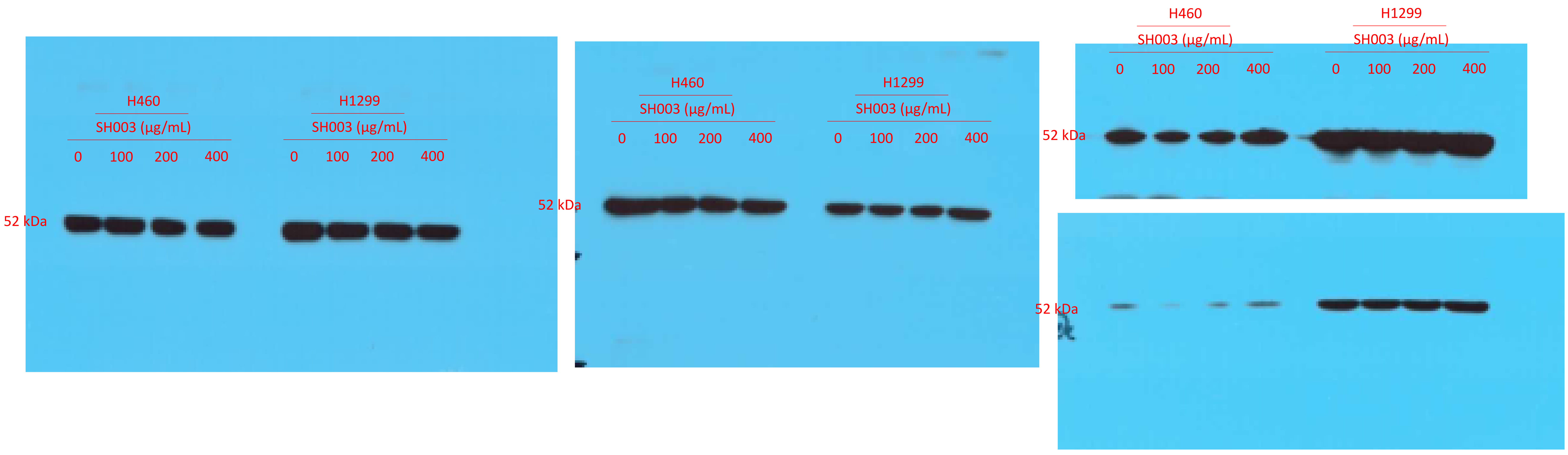

Due to pre-cutting the membranes after transfer, a 10~20 kDa margin was left around the band matching the specified protein size, making it impossible to display a full film image.
